# A Practical and Scalable Approach to Fluoro‐Substituted Bicyclo[1.1.1]pentanes

**DOI:** 10.1002/anie.202205103

**Published:** 2022-06-14

**Authors:** Roman Bychek, Pavel K. Mykhailiuk

**Affiliations:** ^1^ Enamine Ltd. Chervonotkatska 60 02094 Kyiv Ukraine

**Keywords:** Bicyclo[1.1.0]butane, Bicyclo[1.1.1]pentane, Bioisosteres, Fluorine

## Abstract

After more than 20 years of trials, a practical scalable approach to fluoro‐substituted bicyclo[1.1.1]pentanes (F‐BCPs) has been developed. The physicochemical properties of the F‐BCPs have been studied, and the core was incorporated into the structure of the anti‐inflammatory drug Flurbiprofen in place of the fluorophenyl ring.

## Graphical abstract

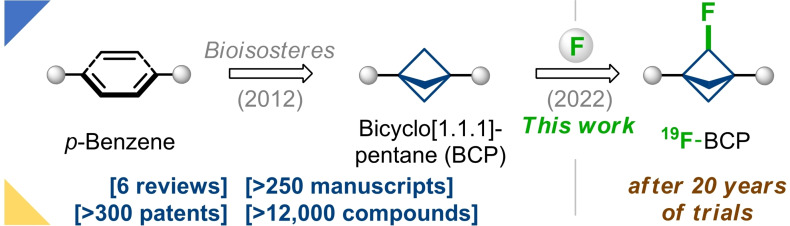

The phenyl ring is one of the most popular structural motifs in natural compounds and synthetic drugs.^[1]^ Ten years ago, Stepan and co‐workers replaced the phenyl ring in a γ‐secretase inhibitor with the bicyclo[1.1.1]pentyl (BCP) skeleton.^[2]^ The obtained analogue showed higher activity and improved physicochemical properties—that was a starting point for the sunrise of bicyclo[1.1.1]pentanes in various aspects of chemistry: from medicinal chemistry to supramolecular chemistry. Today, bicyclo[1.1.1]pentanes are highly popular in academic and industrial research.[^3^, ^4^, ^5^] In fact, at least 6 reviews,[^6^, ^7^] 250 research manuscripts, 300 patents, and 12 000 BCP‐containing compounds appeared during the past decade (Figure 1).^[8]^ Moreover, the number of BCP‐containing molecules is growing dramatically every year (Figure 2).

**Figure 1:**
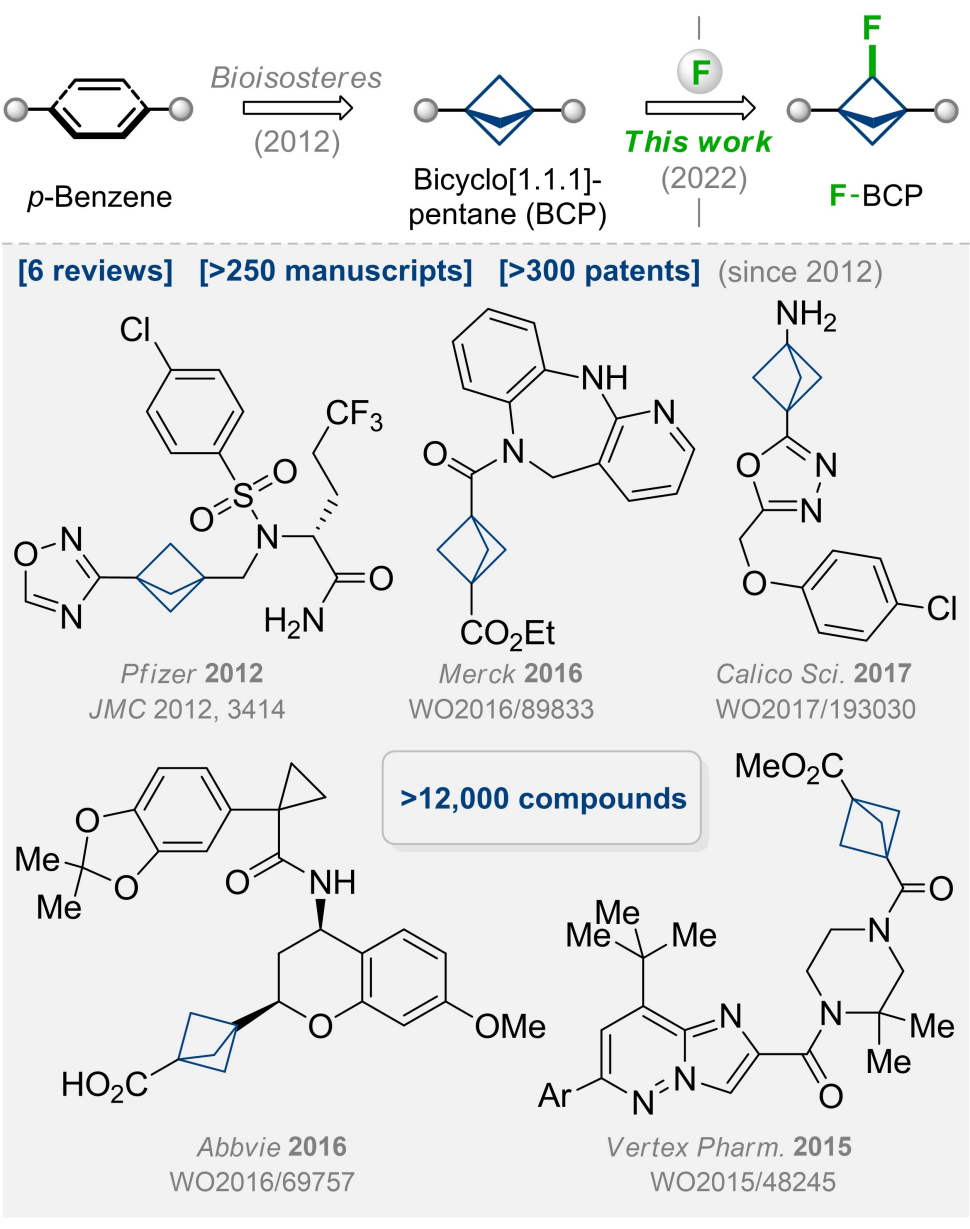
Bicyclo[1.1.1]pentanes (BCPs): state‐of‐the‐art. Aim of this work: fluoro‐bicyclo[1.1.1]pentanes (F‐BCPs).

**Figure 2:**
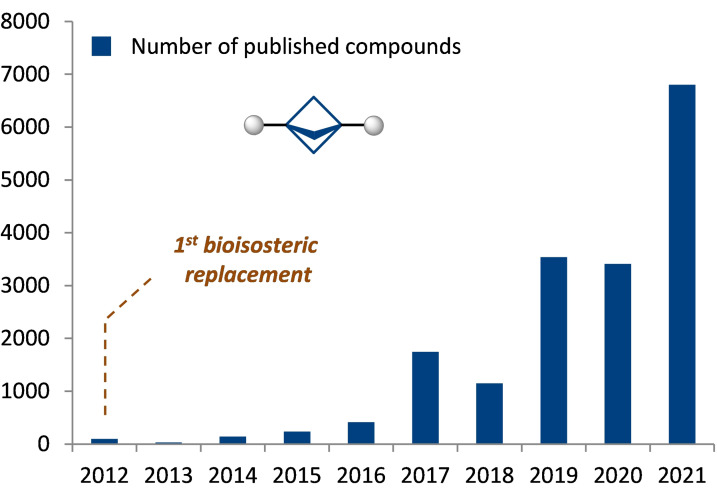
Number of bicyclo[1.1.1]pentanes (BCPs) published every year.

Chemists often incorporate a fluorine atom into organic molecules to fine‐tune their physicochemical properties,^[9]^ adjust the acidity/basicity of the neighboring functional groups,^[10]^ and control the conformation.^[11]^ Given the high popularity of bicyclo[1.1.1]pentanes, it is not surprising that chemists also tried to selectively decorate them with a fluorine atom. Indeed, previously, synthetic approaches to the corresponding bridgehead‐,[^12^, ^13^] gem‐^[14]^ and polyfluorinated^[15]^ derivatives had appeared, and these molecules possess significant value for medicinal chemistry purposes.[^14a^, ^14b^, ^14c^] At the same time, bicyclo[1.1.1]pentanes with a single fluorine atom in the bridge position have remained elusive to this day.

Previously, several attempts to synthesize monofluorinated bicyclo[1.1.1]pentanes (^19^F‐BCPs) were reported in the literature. In 2001, Michl and colleagues performed fluorination of diacid **1** by a mixture of fluorine gas with helium. Saponification and the subsequent esterification with diazomethane gave a mixture of 14 isomeric/homologous polyfluorinated compounds (Scheme 1). The needed diester **2** was present in 7 % according to ^19^F NMR. It was isolated by a silica gel column and additional preparative GC.^[15b]^ In 2019, our group also tried to synthesize ^19^F‐BCPs by directed‐group mediated CH‐activation of amides **3** and **4**. In both cases, the formation of the needed products was not observed.^[14b]^ In 2019, Baran and colleagues developed a practical electrochemical C(sp^3^)‐H fluorination.^[16]^ Being involved in the project at that time, we also tried to achieve electrochemical fluorination of diacid **1**.^[14b]^ Again, the formation of the needed product was not detected.

**Scheme 1:**
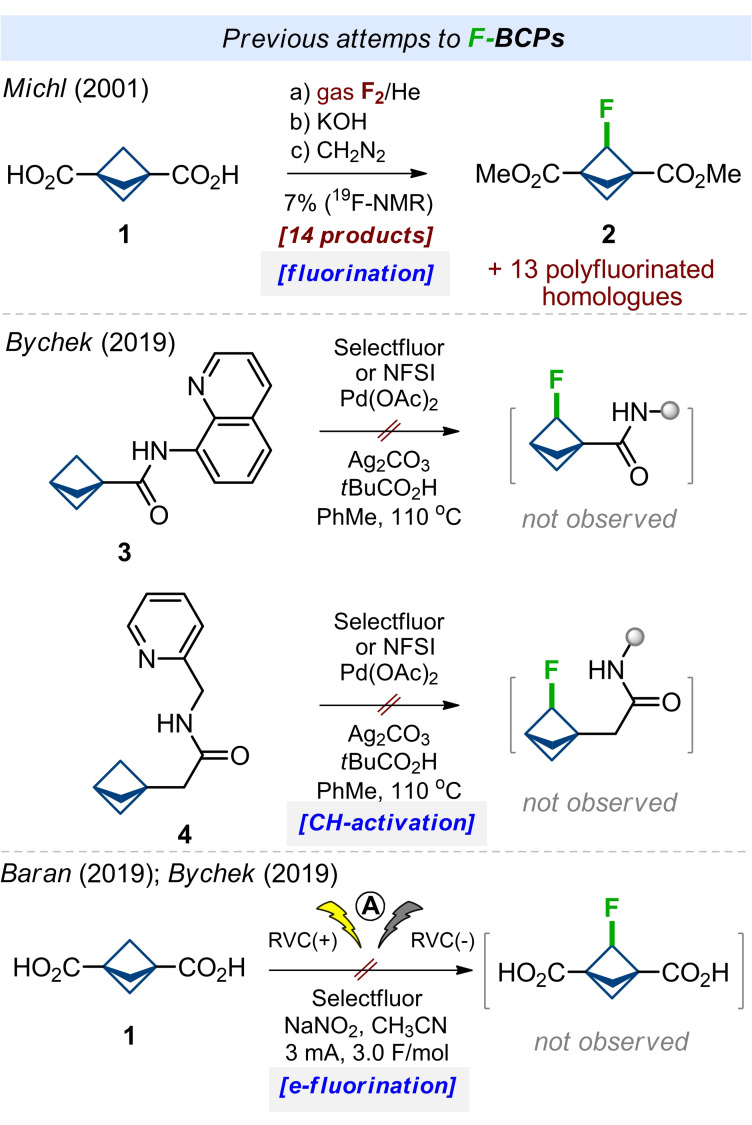
Previous attempts to fluoro‐bicyclo[1.1.1]pentanes (^19^F‐BCPs).

In this work, we finally solved this long‐standing problem and developed a practical scalable approach to fluoro‐bicyclo[1.1.1]pentanes (^19^F‐BCPs).

After unsuccessful attempts to access ^19^F‐BCPs by either protecting‐group directed CH‐activation of BCP‐scaffold or its electrochemical fluorination (Scheme 1), we decided to challenge a strain‐release addition of carbenes to bicyclo[1.1.0]butanes.^[17]^

The direct addition of fluorocarbene (:CHF) to bicyclo[1.1.0]butanes is unknown. However, previously practical protocols for the addition of fluorocarbene to activated alkenes were developed.[^18^, ^19^] These involved the use of freons CHF_2_I^[18]^ and CH_2_FI.^[19]^ We tried both literature protocols on the model substrate **5**, but the formation of the needed product **6** was not observed (Table 1, entries 1–4). Next, we tried an addition of chlorofluorocarbene (:CClF). Previously, we demonstrated one example of such a reaction between gaseous CHFCl_2_ and methyl analog of compound **5** in a low yield.^[14b]^ We did observe the formation of the needed product **7**, but despite all efforts, we could not increase the reaction yield significantly (entries 5–7). The addition of bromofluorocarbene (:CBrF) to bicyclo[1.1.0]butanes was unknown in the literature, but we also tried it. First, we did not observe the formation of the product (entries 8–10), but screening of solvents and phase‐transfer catalysts (entries 8–15) allowed us to identify the suitable conditions for an efficient process (entry 14). The reaction of substrate **5** with commercially available CHFBr_2_ and aq. NaOH in the presence of NEt_3_BnCl in toluene at room temperature gave the needed product **8** in 71 % yield. Formation of the undesired 1,4‐diene and its cyclopropanated derivatives was also observed, but we could isolate the pure product **8** by column chromatography. From the practical standpoint, the protocol was convenient, because CHFBr_2_ is a liquid at room temperature (b.p. 65 °C), in contrast to the previously attempted gaseous freons (CHF_2_I, CHFCl_2_).

**Table 1:**
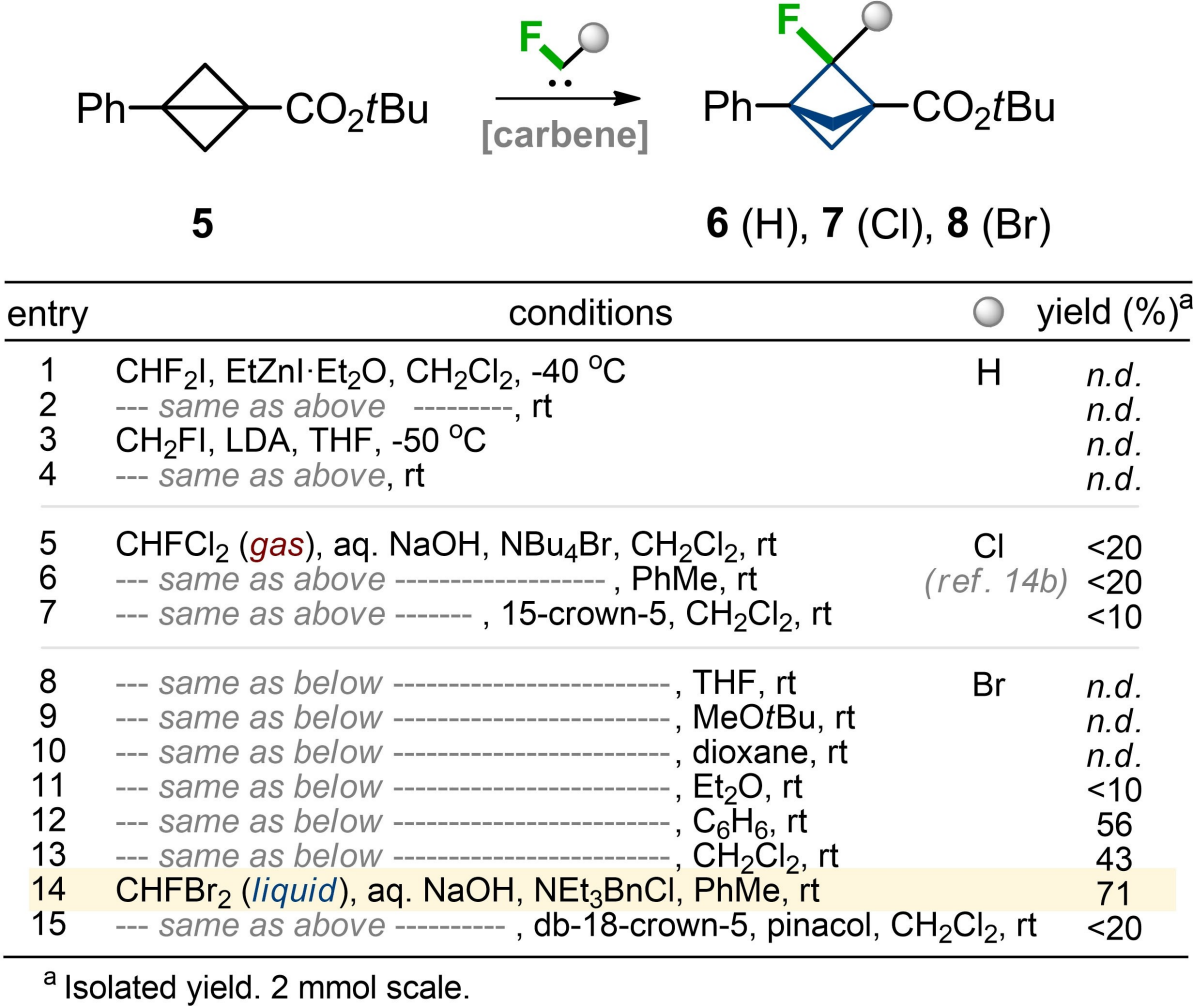
Optimization of synthesis of F‐BCP **8**.

Having an optimized procedure in hand, we studied its scalability. The synthesis commenced from the commercially available ketoacid **10** (ca. 1 €/g, Scheme 2). Chloride **11** was obtained from acid **10** in three steps following the literature protocol.^[14a]^ Cyclization of **11** was performed with NaHMDS in THF at room temperature to provide bicyclo[1.1.0]pentane **5** in 80 % yield. The scaled‐up reaction of **5** with CHFBr_2_ under the previously developed conditions gave bicyclo[1.1.1]pentane **8** in a lower yield of 52 %. However, 21 g of product **8** was obtained in one run. Cleavage of C−Br bond was developed next (Scheme 2). Reduction with either Zn/NH_4_Cl or Pd/H_2_ gave only traces of the product. However, the reaction of bromide **9** with the freshly prepared Raney nickel in the presence of ethylenediamine (EDA) in ethanol smoothly afforded the desired monofluoro‐bicyclo[1.1.1]pentane **9** in 96 % yield. Finally, cleavage of the *tert*‐butyl ester was performed with a catalytic amount of trifluoroacetic acid in dichloromethane at room temperature for one hour. The needed acid **12** was obtained as a crystalline solid in 90 % yield in an 11 g amount. The structure of product **12** was confirmed by X‐ray analysis.^[20]^

**Scheme 2:**
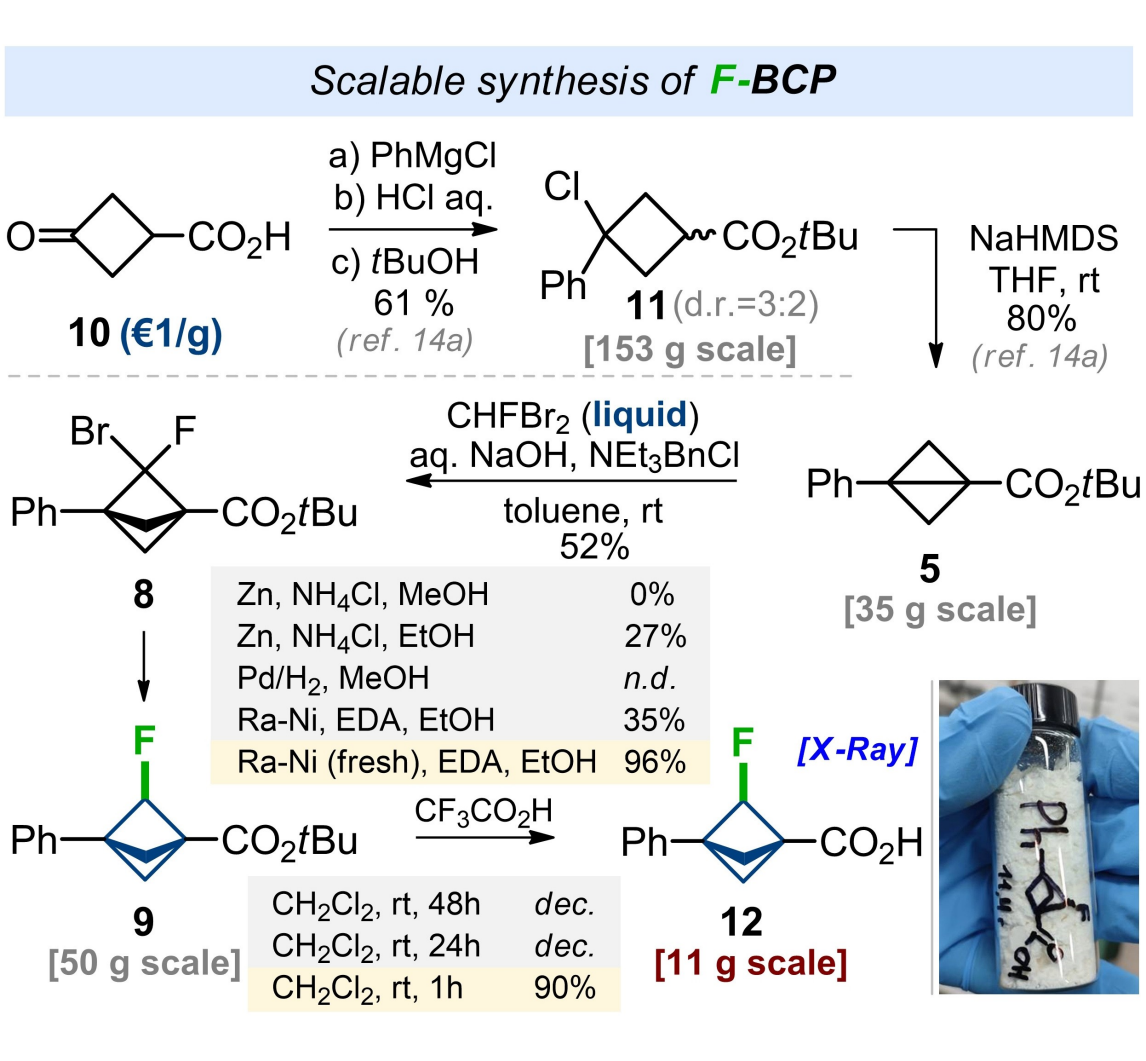
Scalable synthesis of F‐BCP carboxylic acid **12**.

Next, we studied the generality of the developed protocol. First, we synthesized representative starting compounds bearing simple methyl (**13**–**15**), fluorine (**16**, **17**), and trifluoromethyl (**18**, **19**) substituents on the phenyl ring (Scheme 3). All bicyclo[1.1.0]butanes were prepared from ketoacid **10** analogously to the initial substrate **5** (Scheme 2, see Supporting Information). Bicyclo[1.1.0]butanes **13**–**15** with methyl groups reacted with CHFBr_2_ in 56–69 % yield. Interestingly to note, the ortho‐substituted substrate **13** gave the highest yield of 69 %. Fluorine‐substituted substrates **16**, **17** gave the corresponding derivatives **16 a** and **17 a** in 50 % and 32 % yield, correspondingly. Trifluoromethyl‐substituted bicyclo[1.1.0]butanes **18**, **19** gave products **18 a** and **19 a** in 43 % and 52 % yield. All compounds **13 a**–**19 a** were next converted in two steps (via esters **13 b‐**‐**19 b**) into the desired acids **13 c**–**19 c** in high yields. Most of the final acids were obtained on a gram scale.

**Scheme 3:**
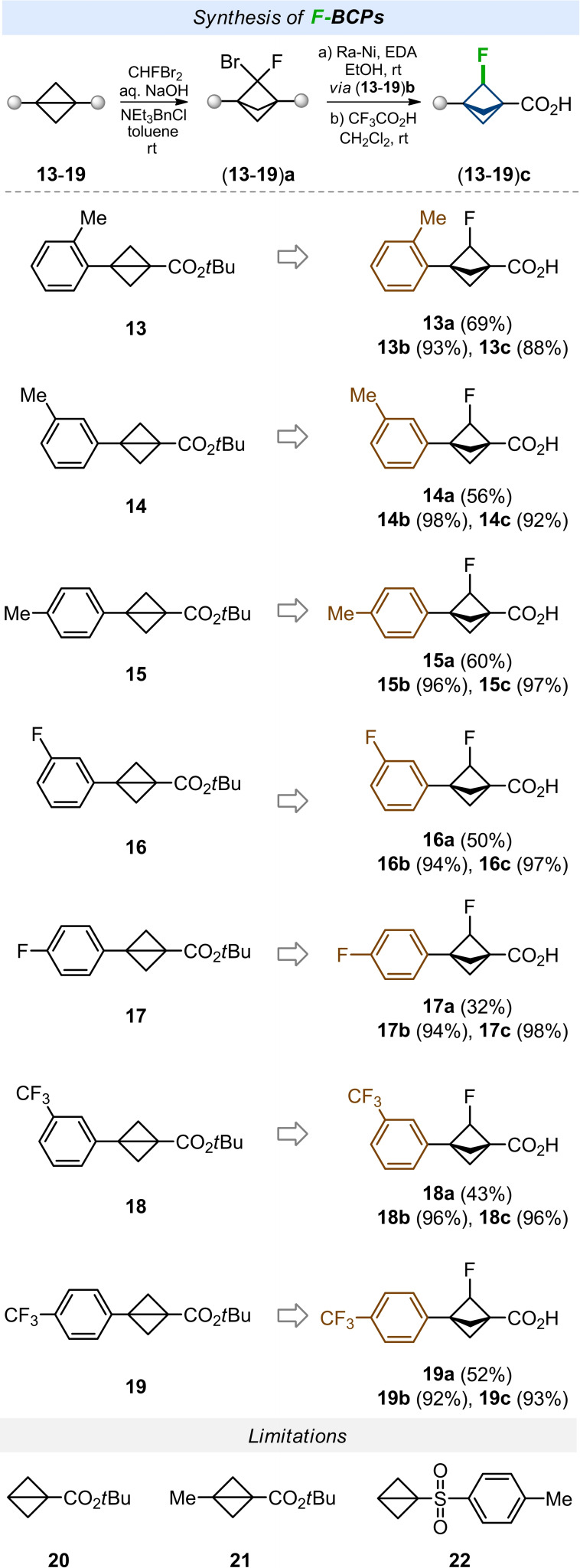
Synthesis of various F‐BCPs.

The developed approach was not without limitations, however. Substrates **20**–**22** having no phenyl substituents failed to react with CHFBr_2_ (Scheme 3).

Some key modifications of fluoro‐bicyclo[1.1.1]pentanes **5** and **12** were undertaken to show their high synthetic value (Scheme 4). Reduction of the ester group with LiAlH_4_ in tetrahydrofuran at room temperature gave alcohol **23** in 92 % yield. Reduction of C−F bond was not observed. Oxidation of the phenyl ring in **5** with NaIO_4_/RuCl_3_ (cat.) in acetonitrile‐water‐tetrachloromethane mixture gave monoacid **24** in 88 % yield. The product was obtained in 10 g amount in one run. Cleavage of the *tert*‐butyl ester with a catalytic amount of trifluoracetic acid in dichloromethane at room temperature gave diacid **25** in 84 % yield. Both compounds **24** and **25** open a way to synthesize various mono‐ and bifunctional derivatives fluoro‐bicyclo[1.1.1]pentanes by standard stepwise modifications of carboxylic groups: synthesis of amides, esters, heterocyclizations etc. This approach, for example, is commonly used to prepare bis‐substituted derivatives of bicyclo[1.1.1]pentanes from diacid **26** and its monoesters (Scheme 4).[^6b^, ^21^] Curtius reaction of acid **12** with (PhO)_2_P(O)N_3_/NEt_3_ in *tert*‐butanol gave, after acidic cleavage of *N*‐Boc group in the intermediate, the final fluoroamine **27** as a hydrochloride salt in 65 % yield. The structure of amine **27** was confirmed by X‐ray analysis.^[20]^

**Scheme 4:**
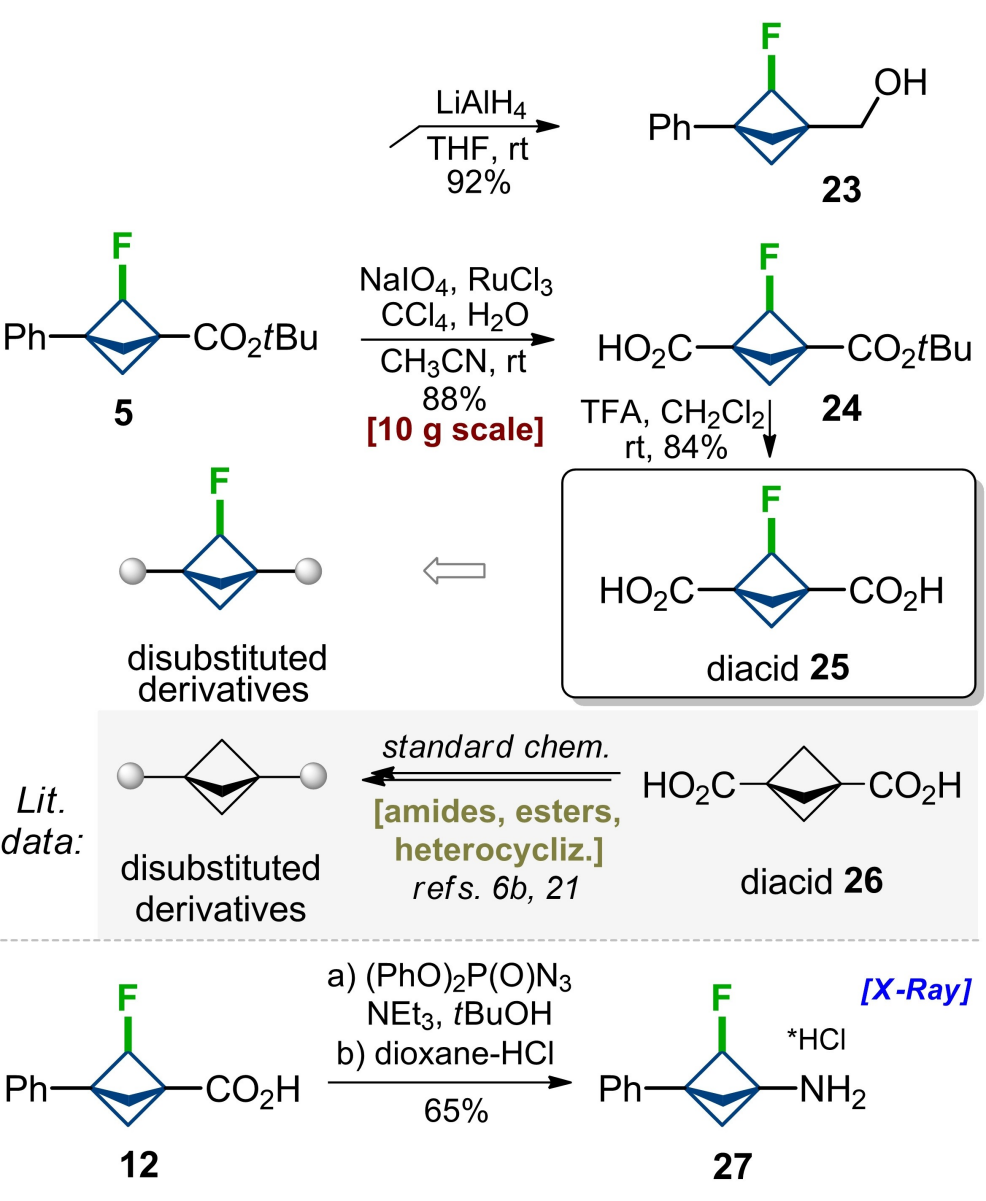
Chemical modifications of F‐BCPs.

All acids **12**, **13 c**–**19 c**, **25**, alcohol **23**, and amine **27** were obtained as crystalline solids. We stored them at room temperature in closed vials on the shelf and did not observe any detectable decomposition according to ^1^H NMR for at least three months.

The incorporation of a fluorine atom into organic compounds could dramatically alter the acidity/basicity of the neighboring functional groups.^[10]^ Therefore, to study the influence of the fluorine atom on the electronic properties of the bicyclo[1.1.1]pentane skeleton, we experimentally measured p*K*_a_ values of acids **28** and **12** and amine hydrochlorides **29** and **27** (Figure 3). Bridge‐fluorination of acid **28** significantly increased its acidity from p*K*_a_ (**28**)=4.2 to p*K*_a_ (**12**)=3.5. On the other hand, bridge‐fluorination of amine **29** reduced its basicity even more ‐ by more than one order of magnitude: p*K*_a_ (29*HCl)=8.2 vs. p*K*_a_ (27*HCl)=6.5. Because basic nitrogen atoms could cause toxicity of bioactive compounds,^[22]^ the incorporation of a fluorine atom at BCP‐containing amines could be a solution here.

**Figure 3:**
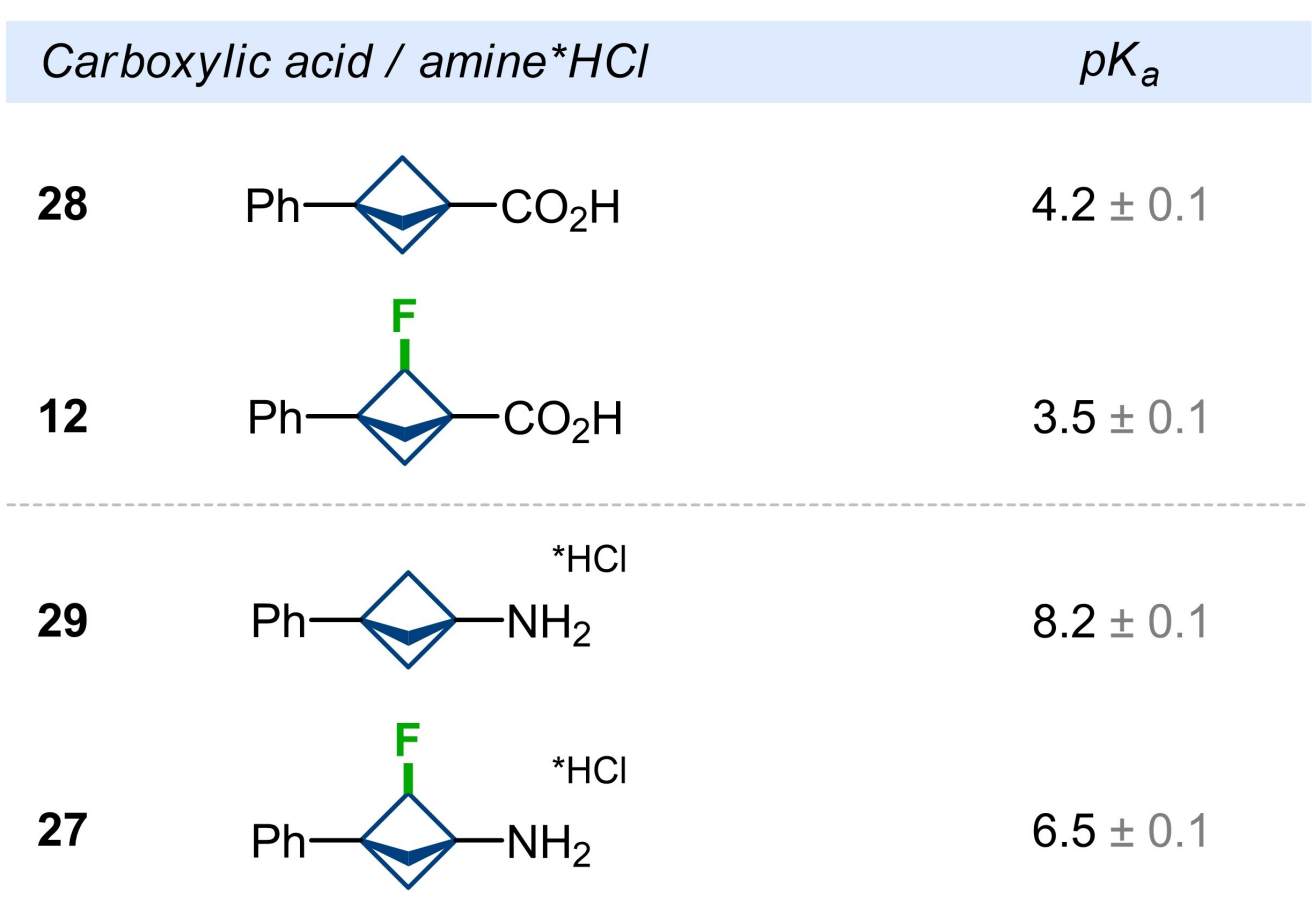
Experimental p*K*_a_ values of F‐BCP carboxylic acids and amines.

Fluorination is known also to affect the physicochemical properties of organic compounds, such as lipophilicity.^[9]^ Therefore, we next calculated lipophilicity as measured by log*P* index of model BCP‐compounds **9**, **30**, and isomeric aromatic compounds **31**, **32** (Figure 4).^[23]^ Incorporation of a fluorine atom into the bicyclo[1.1.1]pentane slightly decreased lipophilicity, *c*log*P*: 3.5 (**30**) vs. 3.3 (**9**) (Figure 4). On the other hand, the lipophilicity of F‐BCP scaffold was almost 2 orders of magnitude lower than that of F−Ph, *c*log*P*: 3.3 (**9**) vs 4.9 (**31**) vs 5.4 (**32**).

**Figure 4:**
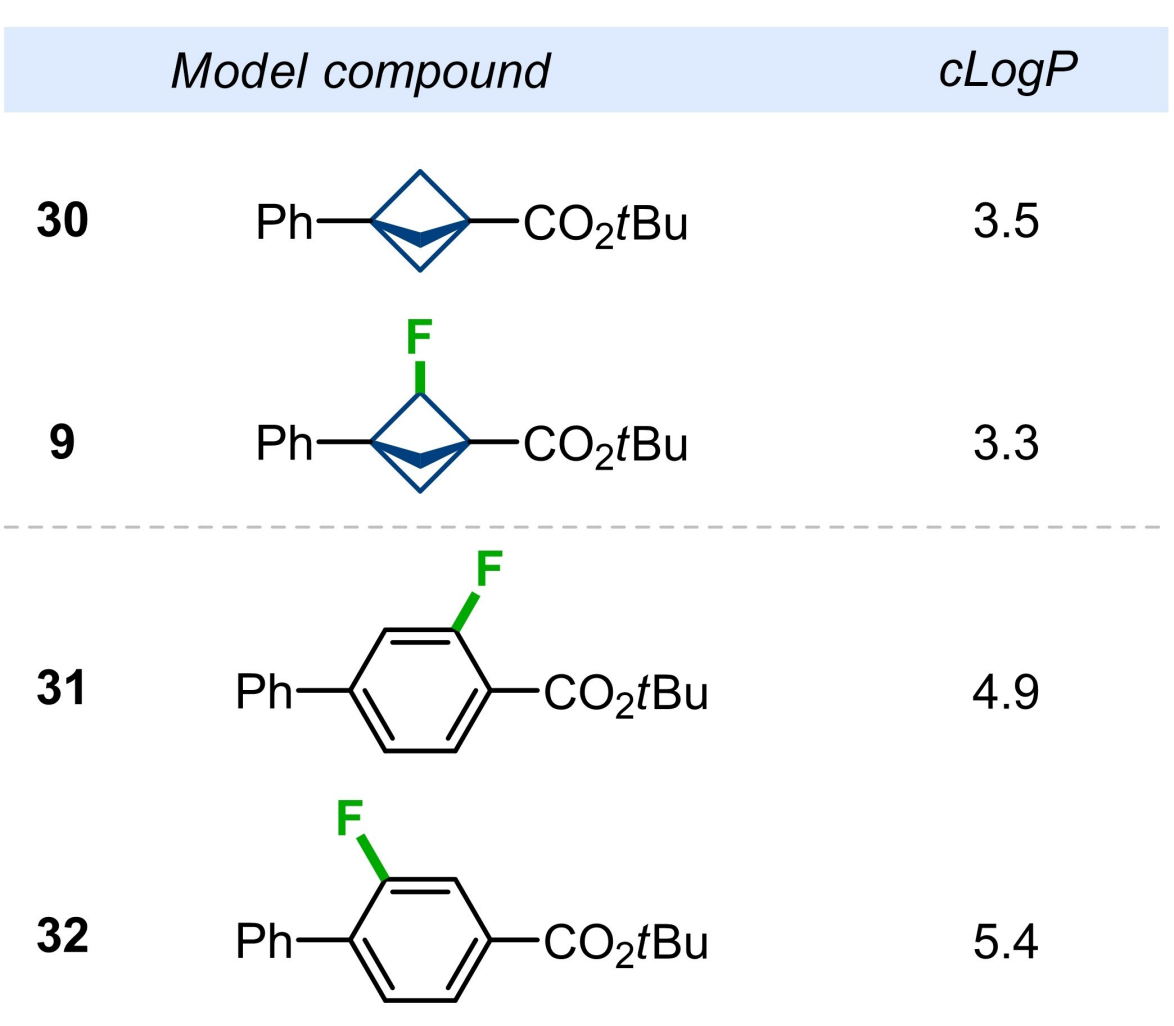
Calculated lipophilicity (*c*log*P*) for compounds **9**, **30**–**32**.

In a short summary, bridge‐fluorination of bicyclo[1.1.1]‐pentane seems to slightly decrease its lipophilicity (*c*log*P*). While the replacement of the fluorophenyl ring (**31**, **32**) with fluoro‐bicyclo[1.1.1]pentane (**9**) dramatically reduced lipophilicity (*c*log*P*) by almost two orders of magnitude, which might be beneficially used in medicinal chemistry programs.

To demonstrate the practical utility of the F‐BCP scaffold, we incorporated it into the structure of Flurbiprofen, a commercialized nonsteroidal anti‐inflammatory drug, instead of fluorophenyl core (Scheme 5). Worth noting, that Flurbiprofen is used in practice as a racemic mixture. The synthesis started from carboxylic acid **12**. Weinreb amide **33** was easily obtained in one step in a 92 % yield. Treatment of the latter with MeMgCl gave ketone **34** in 84 % yield. Wittig reaction of the keto group with (Ph_3_PCH_2_OMe)Cl in tetrahydrofuran in the presence of KO*t*Bu gave alkene **35**. Acidic hydrolysis of the vinyl ether moiety followed by oxidation of the intermediate aldehyde with CrO_3_ gave the needed acid **36** as a non‐separable mixture of two diastereomers. Compounds **36** can be viewed as a 3D‐shaped saturated analogue of Flurbiprofen.

**Scheme 5:**
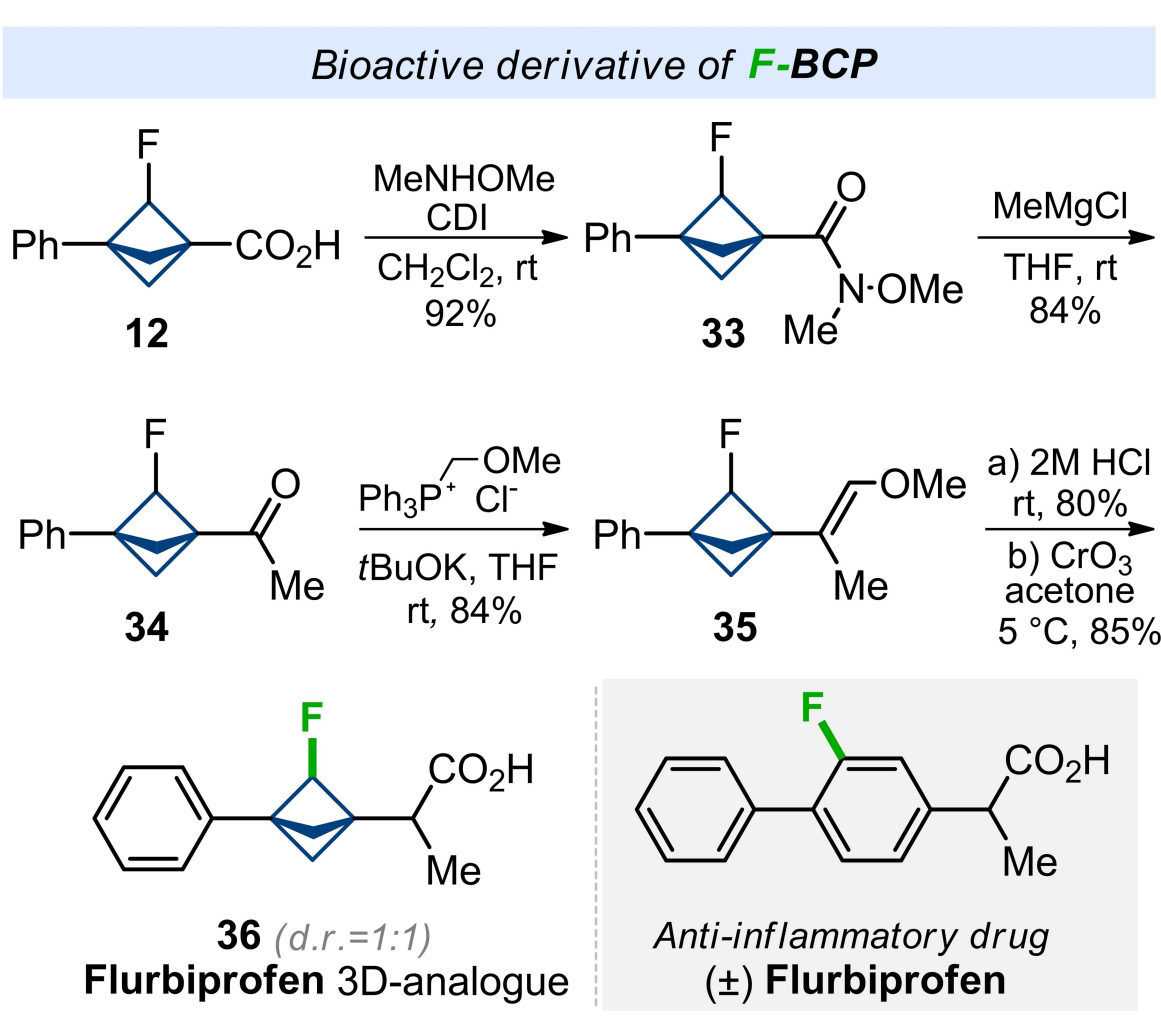
Synthesis of F‐BCP **36**—a saturated analogue of a nonsteroidal anti‐inflammatory drug Flurbiprofen.

Since the pioneering work of Stepan and colleagues ten years ago,^[2]^ bicyclo[1.1.1]pentanes (BCPs) have become extremely popular in chemistry (Figure 2). At least 6 reviews, 250 research manuscripts, and 300 patents are devoted to them.[^3^, ^4^, ^5^, ^6^, ^7^, ^8^] Different research groups, including ours, tried to selectively incorporate a single fluorine atom into the bridge‐position of bicyclo[1.1.1]pentane for a long time. Here, we finally solved this long‐standing problem: a practical scalable method to fluoro‐bicyclo[1.1.1]pentanes (^19^F‐BCP) has been developed. Crystallographic analysis of the obtained compounds was performed, and their physicochemical properties have been studied. Finally, ^19^F‐BCP core was incorporated into the structure of the nonsteroidal anti‐inflammatory drug Flurbiprofen, instead of the fluorophenyl ring.

We believe that with a practical scalable protocol, ^19^F‐BCPs will soon find a solid practical application in medicinal chemistry^[7a]^ and supramolecular chemistry.^[7b]^

## Conflict of interest

The authors declare no conflict of interest.

## Supporting information

As a service to our authors and readers, this journal provides supporting information supplied by the authors. Such materials are peer reviewed and may be re‐organized for online delivery, but are not copy‐edited or typeset. Technical support issues arising from supporting information (other than missing files) should be addressed to the authors.

Supporting Information

## Data Availability

The data that support the findings of this study are available in the Supporting Information of this article.
