# Supplementary material for: A Practical and Scalable Approach to Fluoro‐Substituted Bicyclo[1.1.1]pentanes
Source: Angew Chem Int Ed Engl. 2022 Jun 14;61(29):e202205103. doi: 10.1002/anie.202205103 (PMC9401599; doi:10.1002/anie.202205103)

Angewandte Chemie
Eine Zeitschrift der Gesellschaft Deutscher Chemiker

Supporting Information

# A Practical and Scalable Approach to Fluoro-Substituted Bicyclo[1.1.1]pentanes

*R. Bychek, P. K. Mykhailiuk**

# Supporting Information

## Contents

Experimental Section. Data description and procedures.....3
Experimental *p*Ka.....31
Crystallographic data (X-Ray).....36
Copies of $^{1}$H, $^{13}$C{$^{1}$H} and $^{19}$F{$^{1}$H} NMR spectra.....44
Compound 11.....84
Compound 5.....104
Compound 13.....106
Compound 14.....108
Compound 15.....110
Compound 16.....112
Compound 17.....115
Compound 18.....118
Compound 19.....121
Compound 8.....124
Compound 13a.....127
Compound 14a.....130
Compound 15a.....133
Compound 16a.....136
Compound 17a.....139
Compound 18a.....142
Compound 19a.....145
Compound 9.....148
Compound 13b.....151
Compound 14b.....154
Compound 15b.....157
Compound 16b.....160
Compound 17b.....163

Compound 18b ........................................................................................................166
Compound 19b ........................................................................................................169
Compound 12 ........................................................................................................172
Compound 13c ........................................................................................................175
Compound 14c ........................................................................................................178
Compound 15c ........................................................................................................181
Compound 16c ........................................................................................................184
Compound 17c ........................................................................................................187
Compound 18c ........................................................................................................190
Compound 19c ........................................................................................................193
Compound 23 ........................................................................................................196
Compound 24 ........................................................................................................199
Compound 25 ........................................................................................................202
Compound 27*HCl ........................................................................................................208
Compound 33 ........................................................................................................211
Compound 34 ........................................................................................................214
Compound 35 ........................................................................................................217
Compound 37 ........................................................................................................222

## Experimental Section. Data description and procedures

**General Considerations.** All chemicals were provided by Enamine Ltd. (www.enamine.net). All solvents were treated according to standard methods. All reactions were monitored by thin-layer chromatography (TLC) and were visualized using UV light. Product purification was performed using silica gel column chromatography. TLC-characterization was performed with pre-coated silica gel GF254 (0.2 mm), while column chromatography characterization was performed with silica gel (100-200 mesh). $^{1}$H-NMR spectra were recorded at 400, 500 or 600 MHz (Varian); $^{19}$F-NMR spectra were recorded at 376 MHz (Varian) and $^{13}$C NMR spectra were recorded at 100, 126 or 151 MHz (Varian). $^{1}$H-NMR chemical shifts are calibrated using residual undeuterated solvents $CHCl_3$ (δ = 7.26 ppm) or DMSO (δ = 2.50 ppm). $^{13}$C-NMR chemical shifts for $^{13}$C-NMR are reported relative to the central $CHCl_3$ (δ = 77.16 ppm) or DMSO (δ = 39.52 ppm). Coupling constants are given in Hz. High-resolution mass spectra (HRMS) were recorded on an Agilent LC/MSD TOF mass spectrometer by electrospray ionization time of flight reflectron experiments.

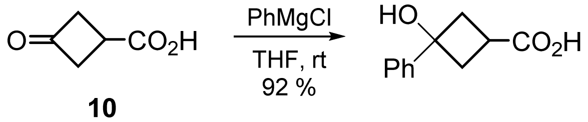


**3-Hydroxy-3-phenylcyclobutane-1-carboxylic acid**

To a solution of **10** (106.0 g, 0.93 mol, 1.0 equiv) in dry THF (1 L) was added PhMgCl (1.9 M, 1 L, 1.90 mol, 2.04 equiv) dropwise under argon at room temperature over 1 h. The mixture was stirred at room temperature for 1 h. A saturated aq. solution of $NH_4Cl$ (250 mL) and water (200 mL) were added dropwise to the solution. The reaction mixture was partially concentrated and acidified with con. HCl (150 mL) and a 2M solution of $NaHSO_4$ (200 mL). The residue was extracted with MTBE (1 × 1000 mL and 1 × 400 mL). The combined organic layers were washed with brine (2 × 400 mL), dried over $Na_2SO_4$, filtered and concentrated under reduced pressure to give the desired compound as a white solid. Yield: 165.12 g, 0.86 mol, 92%, m.p. = 134-135 °C. $^{1}$H NMR (500 MHz, DMSO-$d_6$): δ 12.13 (br s, 1H), 7.51 (d, *J* = 7.4 Hz, 2H), 7.35 (t, *J* = 7.4 Hz, 2H), 7.24 (t, *J* = 7.0 Hz, 1H), 5.68 (br s, 1H), 2.74 – 2.65 (m, 1H), 2.64 – 2.56 (m, 2H), 2.55 – 2.50 (m, 2H) ppm. $^{13}$C {$^{1}$H} NMR (126 MHz, DMSO-$d_6$): δ 176.0, 146.8, 128.1, 126.7, 125.0, 71.0, 41.0, 28.7 ppm. LCMS $(M-H)^-$: 191. HRMS (ESI-TOF) *m/z*: $[M - H]^-$ calcd for $C_{11}H_{11}O_3$, 191.0708; found 191.0704.

**General procedure A (3-hydroxy-3-(*p*-tolyl)cyclobutane-1-carboxylic acid as an example)**

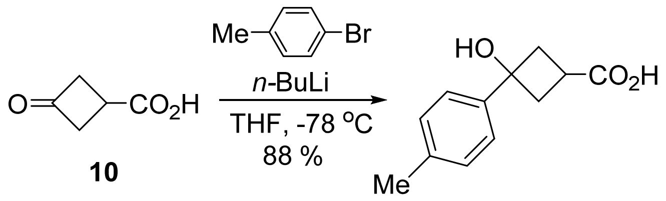


**3-Hydroxy-3-(*p*-tolyl)cyclobutane-1-carboxylic acid**

To a solution of 1-bromo-4-methylbenzene (47.80 g, 0.279 mol, 2.2 equiv) in dry THF (400 mL) was added *n*-BuLi (2.5 M, 112 mL, 0.279 mol, 2.2 equiv) dropwise at -78 °C under argon. The mixture was stirred for 1 h at -78 °C and a solution of 3-oxocyclobutane-1-carboxylic acid (14.50 g, 0.127 mol, 1.0 equiv) in dry THF (50 mL) was added in one portion (reaction temperature became -25 °C). The mixture was stirred for 1 h, and quenched with a sat. solution of $NH_4Cl$ (150 mL) and $H_2O$ (100 mL). The mixture was diluted with hexane (500 mL). The organic layer was separated and washed with water (100 mL). The combined aqueous layers were acidified with a 2M solution of $NaHSO_4$ and extracted with MTBE (300 mL). The organic layer was washed with brine (2 × 150 mL), dried over $Na_2SO_4$, filtered and concentrated under reduced pressure. Yield: 23.07 g, 0.112 mol, 88%, colorless oil. $^1$H NMR (500 MHz, DMSO-$d_6$): δ 12.11 (br s, 1H), 7.39 (d, $J$ = 8.0 Hz, 2H), 7.15 (d, $J$ = 7.9 Hz, 2H), 5.58 (br s, 1H), 2.68 – 2.56 (m, 3H), 2.49 – 2.44 (m, 2H), 2.29 (s, 3H) ppm. $^{13}$C{$^1$H} NMR (126 MHz, DMSO-$d_6$): δ 175.9, 143.7, 135.6, 128.5, 124.9, 70.7, 40.9, 28.6, 20.6 ppm. HRMS (ESI-TOF) *m/z*: [M - H]$^-$ calcd for $C_{12}H_{13}O_3$, 205.0865; found 205.0860.

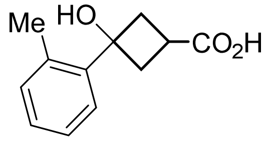


**3-Hydroxy-3-(*o*-tolyl)cyclobutane-1-carboxylic acid**

General procedure A. Scale 0.135 mol. Yield: 24.10 g, 0.117 mol, 87%, colorless oil. $^1$H NMR (500 MHz, DMSO-$d_6$): δ 12.08 (br s, 1H), 7.35 (d, $J$ = 6.9 Hz, 1H), 7.19 – 7.06 (m, 3H), 5.42 (br s, 1H), 2.84 – 2.70 (m, 2H), 2.49 – 2.44 (m, 3H), 2.34 (s, 3H) ppm. $^{13}$C{$^1$H} NMR (126 MHz, DMSO-$d_6$): δ 175.9, 142.1, 137.4, 131.5, 127.1, 125.0, 124.7, 72.6, 29.4, 20.2 ppm. HRMS (ESI-TOF) *m/z*: [M - H]$^-$ calcd for $C_{12}H_{13}O_3$, 205.0865; found 205.0862.

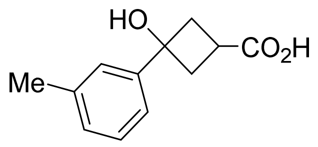


**3-Hydroxy-3-(*m*-tolyl)cyclobutane-1-carboxylic acid**

General procedure A. Scale 0.134 mol. Yield: 21.63 g, 0.105 mol, 78%, colorless oil. $^{1}H$ NMR (400 MHz, DMSO-d$_6$): δ 12.13 (br s, 1H), 7.35 – 7.20 (m, 3H), 7.05 (d, $J$ = 7.0 Hz, 1H), 5.61 (br s, 1H), 2.73 – 2.57 (m, 3H), 2.50 – 2.44 (m, 2H), 2.32 (s, 3H) ppm. $^{13}C\{^{1}H\}$ NMR (126 MHz, DMSO-d$_6$): δ 176.0, 146.7, 137.0, 127.9, 127.3, 125.7, 122.1, 70.9, 40.9, 28.7, 21.2 ppm. HRMS (ESI-TOF) *m/z*: [M - H]$^-$ calcd for $C_{12}H_{13}O_3$, 205.0865; found 205.0862.

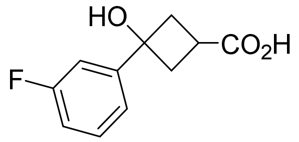


**3-(3-Fluorophenyl)-3-hydroxycyclobutane-1-carboxylic acid**

General procedure A. Scale 0.527 mol. Yield: 85.26 g, 0.406 mol, 77%, colorless oil. $^{1}H$ NMR (500 MHz, DMSO-d$_6$): δ 12.18 (br s, 1H), 7.40 – 7.31 (m, 2H), 7.28 (d, $J$ = 10.7 Hz, 1H), 7.06 (t, $J$ = 7.6 Hz, 1H), 5.82 (s, 1H), 2.83 – 2.69 (m, 1H), 2.65 – 2.50 (m, 4H) ppm. $^{13}C\{^{1}H\}$ NMR (126 MHz, DMSO-d$_6$): δ 175.9, 162.3 (d, $J$ = 243 Hz), 150.1 (d, $J$ = 7 Hz), 130.1 (d, $J$ = 8 Hz), 120.9 (d, $J$ = 2 Hz), 113.4 (d, $J$ = 21 Hz), 112.0 (d, $J$ = 22 Hz), 70.8, 41.0, 28.6 ppm. $^{19}F\{^{1}H\}$ NMR (376 MHz, DMSO-d$_6$): δ -113.8 (s) ppm. HRMS (ESI-TOF) *m/z*: [M - H]$^-$ calcd for $C_{11}H_{10}FO_3$, 209.0614; found 209.0611.

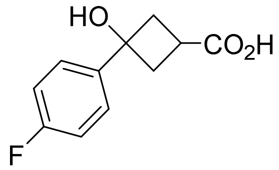


**3-(4-Fluorophenyl)-3-hydroxycyclobutane-1-carboxylic acid**

General procedure A. Scale 0.395 mol. Yield: 66.36 g, 0.316 mol, 80%, colorless oil. $^{1}H$ NMR (400 MHz, DMSO-d$_6$): δ 12.12 (br s, 1H), 7.53 (t, $J$ = 5.5 Hz, 2H), 7.15 (t, $J$ = 8.5 Hz, 2H), 5.74 (br s, 1H), 2.76 – 2.50 (m, 5H) ppm. $^{13}C\{^{1}H\}$ NMR (101 MHz, DMSO-d$_6$): δ 175.9, 161.1 (d, $J$ = 243 Hz), 143.0 (d, $J$ = 3 Hz), 127.1 (d, $J$ = 8 Hz), 114.7 (d, $J$ = 21 Hz), 70.7, 41.0, 28.6 ppm. $^{19}F\{^{1}H\}$ NMR (376 MHz, DMSO-d$_6$): δ -117.0 (s) ppm. HRMS (ESI-TOF) *m/z*: [M - H]$^-$ calcd for $C_{11}H_{10}FO_3$, 209.0614; found 209.0611.

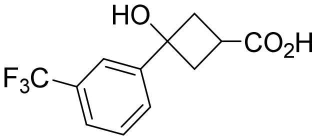


**3-Hydroxy-3-(3-(trifluoromethyl)phenyl)cyclobutane-1-carboxylic acid**

General procedure A. Scale 0.101 mol. Yield: 22.2 g, 0.085 mol, 85%, colorless oil. $^{1}$H NMR (500 MHz, DMSO-d$_6$): δ 12.19 (br s, 1H), 7.83 (d, *J* = 6.8 Hz, 1H), 7.80 (s, 1H), 7.66 – 7.55 (m, 2H), 5.95 (br s, 1H), 2.80 (p, *J* = 8.8 Hz, 1H), 2.69 – 2.59 (m, 2H), 2.58 – 2.51 (m, 2H) ppm. $^{13}$C{$^{1}$H} NMR (126 MHz, DMSO-d$_6$): δ 175.7, 148.4, 129.3, 129.0, 128.6 (q, *J* = 32 Hz), 125.9 – 118.0 (m), 70.8, 40.8, 28.5 ppm. $^{19}$F{$^{1}$H} NMR (376 MHz, DMSO-d$_6$): δ -61.5 (s) ppm. HRMS (ESI-TOF) *m/z*: [M - H]$^-$ calcd for $C_{12}H_{10}F_3O_3$, 259.0582; found 259.0575.

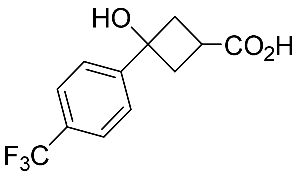


**3-Hydroxy-3-(4-(trifluoromethyl)phenyl)cyclobutane-1-carboxylic acid**

General procedure A. Scale 0.102 mol. Yield: 22.6 g, 0.087 mol, 85%, colorless oil. $^{1}$H NMR (500 MHz, DMSO-d$_6$): δ 12.20 (br s, 1H), 7.71 (dd, *J* = 20.8, 8.3 Hz, 4H), 5.93 (s, 1H), 2.80 (p, *J* = 8.7 Hz, 1H), 2.67 – 2.59 (m, 2H), 2.58 – 2.52 (m, 2H) ppm. $^{13}$C{$^{1}$H} NMR (126 MHz, DMSO-d$_6$): δ 175.8, 151.6, 127.4 (q, *J* = 32 Hz), 125.8, 125.0 (q, *J* = 4 Hz), 124.4 (q, *J* = 272 Hz), 70.8, 40.9, 28.5 ppm. $^{19}$F{$^{1}$H} NMR (376 MHz, DMSO-d$_6$): δ -61.3 (s) ppm. HRMS (ESI-TOF) *m/z*: [M - H]$^-$ calcd for $C_{12}H_{10}F_3O_3$, 259.0582; found 259.0575.

**General procedure B (3-chloro-3-phenylcyclobutane-1-carboxylic acid as an example)**

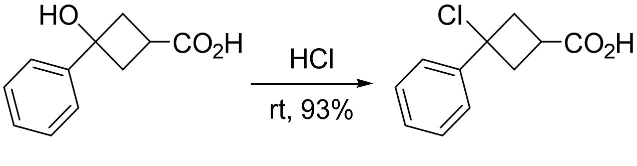


**3-Chloro-3-phenylcyclobutane-1-carboxylic acid**

To a solution of 3-hydroxy-3-phenylcyclobutane-1-carboxylic acid (164.93 g, 0.859 mol, 1.0 equiv) in toluene (1 L) was added con. HCl (1 L, 10.0 mol, 11.6 equiv) dropwise at room temperature. The resulted mixture was stirred at room temperature overnight. The organic phase was separated, washed with water (1 × 200 mL), brine (2 × 200 mL), dried over $Na_2SO_4$, filtered and concentrated under reduced pressure to give the title compound as a colorless oil. Yield 169.0 g, 0.80 mol, 93%. $^{1}$H NMR (500 MHz, DMSO-d$_6$): δ 12.40 (br s, 1H), 7.43 – 7.36 (m, 4H), 7.36 – 7.28 (m, 1H), 3.54

(p, $J$ = 8.7 Hz, 1H), 3.00 – 2.89 (m, 4H) ppm. $^{13}C\{^1H\}$ NMR (126 MHz, DMSO-$d_6$): δ 174.8, 144.9, 128.6, 128.0, 125.1, 69.4, 41.3, 32.2 ppm. HRMS (ESI-TOF) *m/z*: $[M - H]^-$ calcd for $C_{11}H_{10}ClO_2$, 209.0369; found 209.0385.

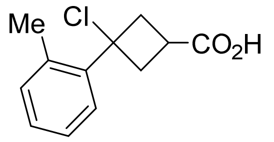


**3-Chloro-3-(*o*-tolyl)cyclobutane-1-carboxylic acid**

General procedure B. Scale 0.116 mol. Yield: 20.88 g, 0.093 mol, 80%, colorless oil. $^1H$ NMR (500 MHz, DMSO-$d_6$): δ 12.42 (br s, 1H), 7.29 – 7.17 (m, 4H), 3.61 – 3.47 (m, 1H), 3.03 – 2.91 (m, 4H), 2.34 (s, 3H) ppm. $^{13}C\{^1H\}$ NMR (151 MHz, DMSO-$d_6$): δ 174.7, 142.1, 135.7, 131.3, 128.4, 126.0, 125.4, 70.7, 42.0, 32.7, 19.5 ppm. HRMS (ESI-TOF) *m/z*: $[M - H]^-$ calcd for $C_{12}H_{12}ClO_2$, 223.0526; found 223.0522.

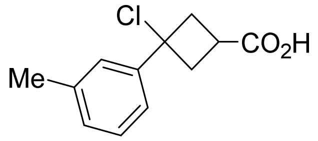


**(3-Chloro-3-(*o*-tolyl)cyclobutyl)(l1-oxidaneyl)methanone**

General procedure B. Scale 0.10 mol. Yield: 19.08 g, 0.085 mol, 85%, colorless oil. Mixture of diastereomers ~ 1:2. $^1H$ NMR (400 MHz, DMSO-$d_6$): δ 12.41 (br s, 1H), 7.49 – 6.99 (m, 4H), 3.53, 2.78 (2 × p, $J$ = 8.5 Hz, 1H), 3.27 – 3.18 (m), 3.01 – 2.88 (m, 4H), 2.34, 2.32 (2 × s, 3H) ppm. $^{13}C\{^1H\}$ NMR (126 MHz, DMSO-$d_6$): δ 174.7, 174.6, 144.8, 137.9, 137.8, 128.7, 128.6, 128.5, 128.4, 126.5, 125.6, 123.0, 122.1, 69.4, 42.2, 41.3, 32.2, 31.4, 21.0, 20.9 ppm. HRMS (ESI-TOF) *m/z*: $[M - H]^-$ calcd for $C_{12}H_{12}ClO_2$, 223.0526; found 223.0525.

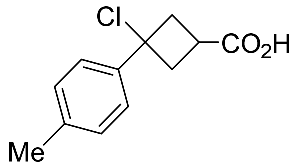


**3-Chloro-3-(*p*-tolyl)cyclobutane-1-carboxylic acid**

General procedure B. Scale 0.112 mol. Yield: 20.88 g, 0.093 mol, 83%, colorless oil. $^1H$ NMR (500 MHz, DMSO-$d_6$): δ 7.27 (d, $J$ = 8.1 Hz, 2H), 7.20 (d, $J$ = 7.9 Hz, 2H), 3.52 (p, $J$ = 8.7 Hz, 1H), 2.95 – 2.89 (m, 4H), 2.30 (s, 3H) ppm. $^{13}C\{^1H\}$ NMR (126 MHz, DMSO-$d_6$): δ 174.7, 142.0, 137.3, 129.0, 125.0, 69.5, 41.4, 32.2, 20.7 ppm. HRMS (ESI-TOF) *m/z*: $[M - H]^-$ calcd for $C_{12}H_{12}ClO_2$, 223.0526; found 223.0527.

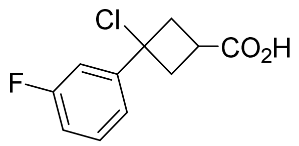


**3-Chloro-3-(3-fluorophenyl)cyclobutane-1-carboxylic acid**

General procedure B. Scale 0.412 mol, reaction time 48 h. Yield: 87.52 g, 0.383 mol, 93%, colorless oil. Mixture of diastereomers ~ 1:4. $^1$H NMR (400 MHz, DMSO-d$_6$): δ 12.47 (br s, 1H), 7.53 – 7.12 (m, 4H), 3.52, 2.82 (2 × p, *J* = 8.8 Hz, 1H), 3.30 – 3.16 (m, 2H), 3.00 – 2.90 (m, 2H) ppm. $^{13}$C{$^1$H} NMR (151 MHz, DMSO-d$_6$): δ 174.6, 174.5, 162.1 (d, *J* = 244 Hz), 162.1 (d, *J* = 245 Hz), 147.4 (d, *J* = 7 Hz), 146.2 (d, *J* = 7 Hz), 130.7 (d, *J* = 9 Hz), 130.7 (d, *J* = 8 Hz), 122.2 (d, *J* = 3 Hz), 121.3 (d, *J* = 3 Hz), 115.0 (d, *J* = 21 Hz), 114.9 (d, *J* = 21 Hz), 113.2 (d, *J* = 23 Hz), 112.3 (d, *J* = 22 Hz), 68.3, 65.1, 42.1, 41.2, 32.1, 31.3 ppm. $^{19}$F{$^1$H} NMR (376 MHz, DMSO-d$_6$): δ -112.8 (s), -112.9 (s) ppm. HRMS (ESI-TOF) *m/z*: [M - H]$^-$ calcd for $C_{11}H_9ClFO_2$, 227.0275; found 227.0270.

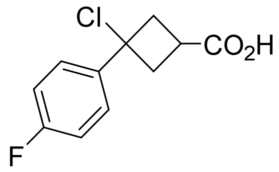


**3-Chloro-3-(4-fluorophenyl)cyclobutane-1-carboxylic acid**

General procedure B. Scale 0.45 mol. Yield: 95.51 g, 0.418 mol, 92%, colorless oil. Mixture of diastereomers ~ 1:2. $^1$H NMR (500 MHz, DMSO-d$_6$): δ 12.23 (br s, 1H), 7.63 – 7.14 (m, 4H), 3.57 – 3.45 (m), 2.81 – 2.71 (m, 1H), 3.26 – 3.15 (m), 3.03 – 2.83 (m, 4H) ppm. $^{13}$C{$^1$H} NMR (151 MHz, DMSO-d$_6$): δ 174.7, 174.6, 161.6 (d, *J* = 245 Hz), 161.5 (d, *J* = 245 Hz), 141.3 (d, *J* = 3 Hz), 139.8 (d, *J* = 3 Hz), 128.4 (d, *J* = 9 Hz), 127.5 (d, *J* = 9 Hz), 115.4 (d, *J* = 22 Hz), 115.4 (d, *J* = 22 Hz), 68.7, 65.4, 42.3, 41.4, 32.1, 31.3 ppm. $^{19}$F{$^1$H} NMR (376 MHz, DMSO-d$_6$): δ -114.3 (s), -114.4 (s) ppm. HRMS (ESI-TOF) *m/z*: [M - H]$^-$ calcd for $C_{11}H_9ClFO_2$, 227.0275; found 227.0271.

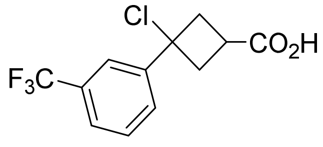


**3-Chloro-3-(3-(trifluoromethyl)phenyl)cyclobutane-1-carboxylic acid**

To a solution of 3-hydroxy-3-(3-(trifluoromethyl)phenyl)cyclobutane-1-carboxylic acid (22.10 g, 0.085 mol, 1.0 equiv) in toluene (150 mL) was added con. HCl (200 mL) dropwise at room temperature. The resulted mixture was stirred at room temperature for 48 h and additional portion of con HCl (150 mL) was added. The organic phase was separated, washed with water (1 × 70 mL), brine (2 × 70 mL), dried over $Na_2SO_4$, filtered and concentrated under reduced pressure to give the

title compound as a colorless oil. Yield: 20.05 g, 0.072 mol, 85%. $^{1}$H NMR (400 MHz, DMSO-d$_6$): δ 12.49 (br s, 1H), 7.90 (d, *J* = 7.7 Hz, 1H), 7.87 (s, 1H), 7.77 – 7.62 (m, 2H), 3.32 – 3.26 (m, 2H), 3.08 – 2.94 (m, 2H), 2.85 (p, *J* = 8.5 Hz, 1H) ppm. $^{13}$C{$^{1}$H} NMR (126 MHz, DMSO-d$_6$): δ 174.6, 144.8, 130.3, 129.9, 129.5 (q, *J* = 32 Hz), 125.0 (d, *J* = 11 Hz), 124.0 (q, *J* = 273 Hz), 122.5 (q, *J* = 4 Hz), 65.2, 41.9, 31.2 ppm. $^{19}$F{$^{1}$H} NMR (376 MHz DMSO-d$_6$): δ -61.5 (s) ppm. HRMS (ESI-TOF) *m/z*: [M - H]$^-$ calcd for $C_{12}H_9ClF_3O_2$, 277.0243; found 277.0237.

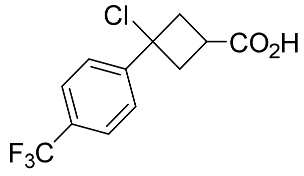


**3-Chloro-3-(4-(trifluoromethyl)phenyl)cyclobutane-1-carboxylic acid**

General procedure B. Scale 0.087 mol. Yield: 21.68 g, 0.078 mol, 90%, colorless oil. Mixture of diastereomers ~ 1:2. $^{1}$H NMR (500 MHz, DMSO-d$_6$): δ 12.47 (br s, 1H), 7.83 – 7.58 (m, 4H), 3.55 (p, *J* = 8.7 Hz), 2.86 (p, *J* = 8.5 Hz, 1H), 3.31 – 3.21 (m), 3.07 – 2.90 (m, 4H) ppm. $^{13}$C{$^{1}$H} NMR (151 MHz, DMSO-d$_6$): δ 174.6, 174.5, 148.9, 147.8, 128.5 (q, *J* = 32 Hz), 127.0, 126.2, 125.7 – 125.5 (m), 124.0 (q, *J* = 272 Hz), 67.9, 65.0, 42.0, 41.1, 32.1, 31.2 ppm. $^{19}$F{$^{1}$H} NMR (376 MHz, DMSO-d$_6$): δ -61.7 (s), -61.7 (s) ppm. HRMS (ESI-TOF) *m/z*: [M - H]$^-$ calcd for $C_{12}H_9ClF_3O_2$, 277.0243; found 277.0234.

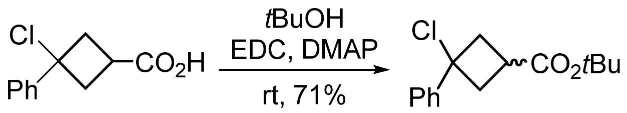


**Tert-butyl 3-chloro-3-phenylcyclobutane-1-carboxylate (11)**

To a solution of 3-chloro-3-phenylcyclobutane-1-carboxylic acid (169.0 g, 0.80 mol, 1.0 equiv) in DMF (500 mL) were added *t*BuOH (355.20 g, 4.80 mol, 6.0 equiv), EDC (229.80 g, 1.20 mol, 1.5 equiv) and DMAP (100.04 g, 0.82 mol, 1.02 equiv). The mixture was stirred at room temperature for 72 h. The reaction mixture was diluted with a mixture of 1M $NaHSO_4$ (1 L), MTBE (1 L) and hexane (500 mL). The organic layer was separated and washed with 1M $NaHSO_4$ (1 × 800 mL), water (1 × 200 mL), 1M $NaHCO_3$ (2 × 400 mL) and brine (1 × 500 mL). The organic layer was dried over $Na_2SO_4$, filtered over $SiO_2$ and concentrated under reduced pressure. Yield: 152.97 g, 0.574 mol, 71%, colorless oil. Mixture of diastereomers (~ 3:2). $^{1}$H NMR (400 MHz, $CDCl_3$): δ 7.54 – 7.27 (m, 5H), 3.60 (p, *J* = 8.8 Hz), 2.78 (p, *J* = 8.6 Hz, 1H), 3.22 – 2.90 (m, 4H), 1.48, 1.42 (2×s, 9H) ppm. $^{13}$C {$^{1}$H} NMR (101 MHz, $CDCl_3$): δ 173.4, 145.2, 128.7, 128.7, 128.1, 128.0, 126.0, 125.2, 81.0, 80.8, 68.6, 42.7, 41.8, 34.0, 33.2, 28.2, 28.1 ppm. HRMS (ESI-TOF) *m/z*: [M + Na]$^+$ calcd for $C_{15}H_{19}ClNaO_2$, 289.0971; found 289.1000.

**General procedure C (tert-butyl 3-chloro-3-(*o*-tolyl)cyclobutane-1-carboxylate as an example)**

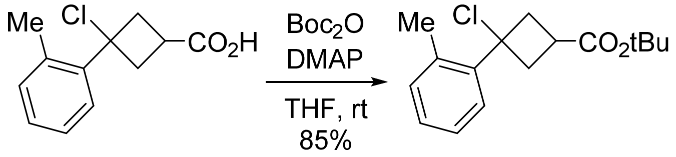


**Tert-butyl 3-chloro-3-(*o*-tolyl)cyclobutane-1-carboxylate**

To a solution of 3-chloro-3-(*p*-tolyl)cyclobutane-1-carboxylic acid (2.25 g, 0.01 mol, 1.0 equiv) in THF (30 mL) were added $Boc_2O$ (2.60 g, 0.012 mol, 1.2 equiv) and DMAP (60 mg). The mixture was stirred at room temperature overnight and concentrated under reduced pressure. The residue was dissolved in a mixture of MTBE and hexane (30 mL, 1:1). The solution was washed with 1M $NaHSO_4$ (10 mL), 1M $NaHCO_3$ (10 mL), brine (10 mL), filtered through $SiO_2$ and concentrated under reduced pressure. Yield: 2.40 g, 0.0085 mol, 85%, colorless oil. $^1H$ NMR (500 MHz, $CDCl_3$): δ 7.26 – 7.05 (m, 5H), 3.61 (p, $J$ = 9.0 Hz, 1H), 3.05 – 2.99 (m, 4H), 2.41 (s, 3H), 1.44 (s, 9H) ppm. $^{13}C\{^1H\}$ NMR (151 MHz, $CDCl_3$): δ 173.4, 142.5, 136.2, 131.7, 128.5, 126.1, 125.5, 80.8, 69.8, 42.5, 34.4, 28.2, 20.1 ppm. HRMS (ESI-TOF) *m/z*: $[M + Na]^+$ calcd for $C_{16}H_{21}ClNaO_2$, 303.1128; found 303.1136.

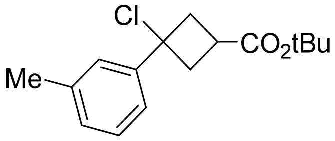


**Tert-butyl 3-chloro-3-(*m*-tolyl)cyclobutane-1-carboxylate**

General procedure C. Scale 0.086 mol. Yield: 22.44 g, 0.080 mol, 93%, colorless oil. Mixture of diastereomers ~ 1:2. $^1H$ NMR (500 MHz, $CDCl_3$): δ 7.34 – 7.06 (m, 4H), 3.59 (p, $J$ = 8.8 Hz), 2.75 (p, $J$ = 8.5 Hz) 1H, 3.20 – 3.05 (m), 3.02 – 2.90 (m) 4H, 2.37 (s), 2.36 (s, 3H), 1.47 (s), 1.41 (s) 9H ppm. $^{13}C\{^1H\}$ NMR (126 MHz, $CDCl_3$): δ 173.4, 173.0, 145.2, 143.7, 138.4, 138.4, 128.9, 128.8, 128.6, 126.8, 125.9, 123.0, 122.2, 81.0, 80.8, 68.7, 64.9, 42.7, 41.8, 34.1, 33.2, 28.1, 27.5, 21.6, 21.5 ppm. HRMS (ESI-TOF) *m/z*: $[M + Na]^+$ calcd for $C_{16}H_{21}ClNaO_2$, 303.1128; found 303.1136.

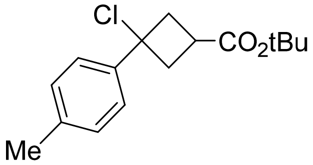


**Tert-butyl 3-chloro-3-(*p*-tolyl)cyclobutane-1-carboxylate**

General procedure C. Scale: 0.01 mol. Yield: 2.70 g, 0.0096 mol, 96%, colorless oil. Mixture of diastereomers ~ 1:4. $^1H$ NMR (500 MHz, $CDCl_3$): δ 7.40 (d, $J$ = 8.0 Hz), 7.19 (d, $J$ = 8.3 Hz), 7.25

(d, $J$ = 7.9 Hz,), 7.16 (d, $J$ = 7.9 Hz) 4H, 3.58 (p, $J$ = 8.8 Hz), 2.75 (p, $J$ = 8.5 Hz) 1H, 3.29 – 2.84 (m, 4H), 2.36 (s), 2.34 (s) 3H, 1.47 (s), 1.41 (s) 9H ppm. $^{13}C\{^1H\}$ NMR (126 MHz, $CDCl_3$): δ 173.4, 173.0, 142.4, 140.9, 138.0, 137.8, 129.4, 129.3, 125.9, 125.1, 80.9, 80.8, 68.8, 64.8, 42.7, 41.9, 34.0, 33.2, 28.1, 21.24, 21.19 ppm. HRMS (ESI-TOF) *m/z*: $[M + H]^+$ calcd for $C_{16}H_{22}ClO_2$, 281.1308; found 281.1314.

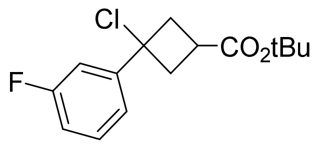


**Tert-butyl 3-chloro-3-(3-fluorophenyl)cyclobutane-1-carboxylate**

General procedure C. Scale 0.372 mol. Yield: 95.31 g, 0.335 mol, 90%, colorless oil. Mixture of diastereomers ~ 3:2. $^1H$ NMR (400 MHz, $CDCl_3$): δ 7.39 – 6.87 (m, 4H), 3.55 (p, $J$ = 8.7 Hz), 2.77 (p, $J$ = 8.5 Hz) 1H, 3.14 – 2.88 (m, 4H), 1.45 (s), 1.40 (s) 9H ppm. $^{13}C\{^1H\}$ NMR (151 MHz, $CDCl_3$): δ 173.3, 172.8, 162.9 (d, $J$ = 247 Hz), 162.9 (d, $J$ = 247 Hz), 147.6 (d, $J$ = 7 Hz), 146.4 (d, $J$ = 7 Hz), 130.3 (d, $J$ = 8 Hz), 130.3 (d, $J$ = 9 Hz), 121.6 (d, $J$ = 3 Hz), 120.9 (d, $J$ = 3 Hz), 115.1 (d, $J$ = 22 Hz), 115.0 (d, $J$ = 21 Hz), 113.4 (d, $J$ = 23 Hz), 112.6 (d, $J$ = 22 Hz), 81.2, 81.0, 67.5, 64.0, 42.7, 41.8, 33.9, 33.2, 28.2, 28.1 ppm. $^{19}F\{^1H\}$ NMR (376 MHz, $CDCl_3$): δ -112.7 (s), -112.8 (s) ppm. HRMS (ESI-TOF) *m/z*: $[M + Na]^+$ calcd for $C_{15}H_{18}ClFNaO_2$, 307.0877; found 307.0875.

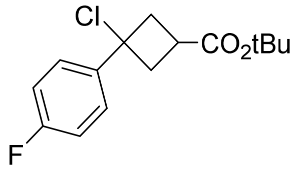


**Tert-butyl 3-chloro-3-(4-fluorophenyl)cyclobutane-1-carboxylate**

General procedure C. Scale 0.415 mol. Yield: 108.68 g, 0.382 mol, 92%, colorless oil. Mixture of diastereomers ~ 3:2. $^1H$ NMR (400 MHz, $CDCl_3$): δ 7.53 – 6.95 (m, 4H), 3.54 (p, $J$ = 8.8 Hz), 2.74 (p, $J$ = 8.5 Hz) 1H, 3.16 – 2.88 (m, 4H), 1.45 (s), 1.39 (s) 9H ppm. $^{13}C\{^1H\}$ NMR (151 MHz, $CDCl_3$): δ 173.4, 172.8, 162.3 (d, $J$ = 248 Hz), 162.3 (d, $J$ = 248 Hz), 141.2 (d, $J$ = 4 Hz), 139.8 (d, $J$ = 4 Hz), 128.0 (d, $J$ = 9 Hz), 127.2 (d, $J$ = 9 Hz), 115.6 (d, $J$ = 22 Hz), 115.6 (d, $J$ = 22 Hz), 81.1, 81.0, 68.0, 64.3, 42.8, 42.0, 33.9, 33.1, 28.2, 28.1 ppm. $^{19}F\{^1H\}$ NMR (376 MHz, $CDCl_3$): δ -114.30 (s), -114.33 (s) ppm. HRMS (ESI-TOF) *m/z*: $[M + Na]^+$ calcd for $C_{15}H_{18}ClFNaO_2$, 307.0877; found 307.0909.

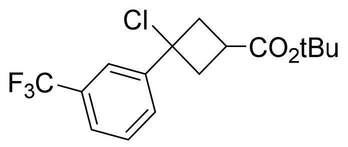

**Tert-butyl 3-chloro-3-(3-(trifluoromethyl)phenyl)cyclobutane-1-carboxylate**

General procedure C. Scale 0.072 mol. Yield: 22.41 g, 0.067 mol, 93%, colorless oil. Mixture of diastereomers ~ 3:2. $^{1}$H NMR (500 MHz, $CDCl_3$): δ 7.79 – 7.44 (m, 4H), 3.61 (p, *J* = 8.7 Hz), 2.79 (p, *J* = 8.5 Hz) 1H, 3.21 – 2.96 (m, 4H), 1.48 (s), 1.42 (s) 9H ppm. $^{13}$C{$^{1}$H} NMR (151 MHz, $CDCl_3$): δ 173.2, 172.6, 146.1, 145.0, 131.2 (q, *J* = 32 Hz), 131.2 (q, *J* = 33 Hz), 129.6, 129.4, 129.3, 128.8, 125.0 (q, *J* = 4 Hz), 124.9 (q, *J* = 4 Hz), 122.8 (q, *J* = 4 Hz), 122.2 (q, *J* = 272 Hz), 122.2 (q, *J* = 4 Hz), 81.3, 81.11 (s), 67.3, 63.9, 42.5, 41.7, 33.9, 33.1, 28.2, 28.1 ppm. $^{19}$F{$^{1}$H} NMR (376 MHz, $CDCl_3$): δ -63.1 (s), -63.2 (s) ppm. HRMS (ESI-TOF) *m/z*: $[M + Na]^+$ calcd for $C_{16}H_{18}ClF_3NaO_2$, 357.0845; found 357.0839.

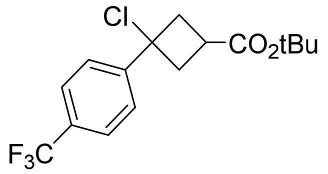

**Tert-butyl 3-chloro-3-(4-(trifluoromethyl)phenyl)cyclobutane-1-carboxylate**

General procedure C. Scale 0.0592 mol. Yield: 17.73 g, 0.053 mol, 90%, colorless oil. Mixture of diastereomers ~ 3:2. $^{1}$H NMR (500 MHz, $CDCl_3$): δ 7.69 – 7.44 (m, 4H), 3.60 (p, *J* = 8.7 Hz), 2.78 (p, *J* = 8.5 Hz) 1H, 3.14 (d, *J* = 8.5 Hz, 2H), 2.98 (d, *J* = 8.6 Hz, 2H), 1.48 (s), 1.42 (s) 9H ppm. $^{13}$C{$^{1}$H} NMR (151 MHz, $CDCl_3$): δ 173.2, 172.7, 148.8, 147.7, 130.3 (q, *J* = 33 Hz), 130.2 (q, *J* = 33 Hz), 126.5, 125.8, 125.8, 124.1 (q, *J* = 272 Hz), 124.0 (q, *J* = 272 Hz), 81.3, 81.1, 67.2, 63.8, 42.5, 41.7, 33.9, 33.1, 28.2, 28.1 ppm. $^{19}$F{$^{1}$H} NMR (376 MHz, $CDCl_3$): δ -63.18 (s), -63.21 (s) ppm. HRMS (ESI-TOF) *m/z*: $[M + Na]^+$ calcd for $C_{16}H_{18}ClF_3NaO_2$, 357.0845; found 357.0857.

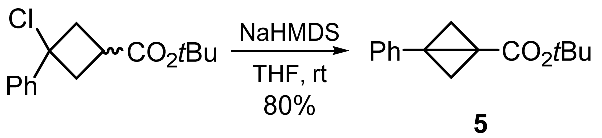

**5**

**Tert-butyl 3-phenylbicyclo[1.1.0]butane-1-carboxylate (5)**

To a solution of NaHMDS (115.0 mL, 0.23 mol, 1.2 eqiuv) in THF (200 mL) was added a solution of **11** (51.0 g, 0.191 mol, 1.0 equiv) under argon (reaction temperature < 35 °C). The resulted mixture was stirred for 2 h at room temperature, cold to 5 °C (an ice bath) and a sat. solution of $NH_4Cl$ (100 mL) and water (100 mL) were added dropwise. The mixture was diluted with hexane (300 mL). The organic layer was separated, washed with brine (2 × 200 mL), dried over $Na_2SO_4$,

filtered through $SiO_2$ and concentrated under reduced pressure. The crude product was immediately used for the next step without purification. Yield: 34.96 g, 0.152 mol, 80%, colorless oil. $^1H$ NMR (400 MHz, $CDCl_3$): δ 6.84 – 6.68 (m, 5H), 2.41 (s, 2H), 1.06 (s, 2H), 0.64 (s, 9H) ppm. $^{13}C$ {$^1H$} NMR (101 MHz, $CDCl_3$): δ 169.0, 134.1, 128.4, 126.8, 125.9, 80.5, 35.8, 31.9, 28.1, 23.9 ppm. HRMS (ESI-TOF) *m/z*: $[M + Na]^+$ calcd for $C_{15}H_{18}NaO_2$, 253.1204; found 253.1205.

**General procedure D (compound 15 as an example)**

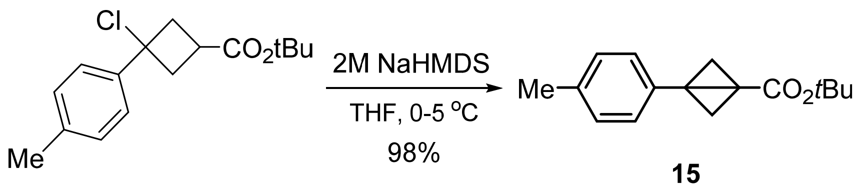


**15**

**Tert-butyl 3-(*p*-tolyl)bicyclo[1.1.0]butane-1-carboxylate (15)**

To a solution of tert-butyl 3-chloro-3-(*p*-tolyl)cyclobutane-1-carboxylate (2.70 g, 0.0096 mol, 1.0 equiv) in THF (25 mL) was added 2M NaHMDS (5.8mL, 0.0115 mol, 1.2 eqiuv) at 0-5 °C under argon. The resulted mixture was stirred for 1 h at 0-5 °C and a 25% solution of $NH_4Cl$ (10 mL) and water (10 mL) were added dropwise. The mixture was diluted with hexane (25 mL). The organic layer was separated and washed with brine (2 × 20 mL), dried over $Na_2SO_4$, filtered through $SiO_2$ and concentrated under reduced pressure. The crude product was immediately used for the next step without purification. Yield: 2.30 g, 0.0094 mol, 98%, colorless oil. $^1H$ NMR (400 MHz, $CDCl_3$): δ 7.19 (d, *J* = 7.9 Hz, 2H), 7.10 (d, *J* = 7.8 Hz, 2H), 2.87 (s, 2H), 2.31 (s, 3H), 1.53 (s, 2H), 1.15 (s, 9H) ppm. $^{13}C${$^1H$} NMR (101 MHz, $CDCl_3$): δ 169.2, 136.5, 130.8, 129.1, 125.8, 80.4, 35.8, 32.2, 28.1, 23.5, 21.2 ppm. HRMS (ESI-TOF) *m/z*: $[M + Na]^+$ calcd for $C_{16}H_{20}NaO_2$, 267.1361; found 267.1342.

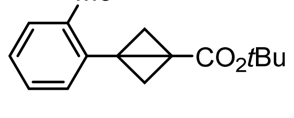


**Tert-butyl 3-(*o*-tolyl)bicyclo[1.1.0]butane-1-carboxylate (13)**

General procedure D. Scale 0.01 mol. Yield: 2.20 g, 0.009 mol, 90%, colorless oil. $^1H$ NMR (400 MHz, $CDCl_3$): δ 7.22 – 7.00 (m, 4H), 2.57 (s, 2H), 2.46 (s, 3H), 1.56 (s, 2H), 1.38 (s, 9H) ppm. $^{13}C${$^1H$} NMR (101 MHz, $CDCl_3$): δ 170.1, 139.0, 132.8, 130.7, 127.2, 125.7, 125.5, 80.9, 38.8, 30.1, 28.4, 21.7, 20.5 ppm. HRMS (ESI-TOF) *m/z*: $[M + H]^+$ calcd for $C_{16}H_{21}O_2$, 245.1542; found 245.1539.

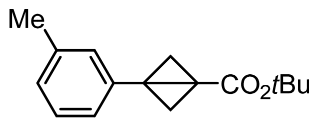

**Tert-butyl 3-(*m*-tolyl)bicyclo[1.1.0]butane-1-carboxylate (14)**

General procedure D. Scale 0.0118 mol. Yield: 2.62 g, 0.0107 mol, 91%, white solid, m.p. = 42-43 °C. $^1$H NMR (400 MHz, $CDCl_3$): δ 7.23 – 6.94 (m, 4H), 2.88 (s, 2H), 2.33 (s, 3H), 1.53 (s, 2H), 1.15 (s, 9H) ppm. $^{13}C\{^1H\}$ NMR (151 MHz, $CDCl_3$): δ 169.1, 137.9, 133.9, 128.3, 127.7, 126.5, 123.1, 80.4, 35.9, 32.0, 28.1, 23.7, 21.5 ppm. HRMS (ESI-TOF) *m/z*: $[M + Na]^+$ calcd for $C_{16}H_{20}NaO_2$, 267.1361; found 267.1358.

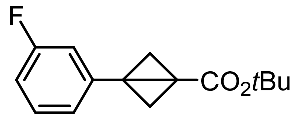

**Tert-butyl 3-(3-fluorophenyl)bicyclo[1.1.0]butane-1-carboxylate (16)**

General procedure D. Scale 0.0372 mol. Yield: 8.38 g, 0.0338 mol, 91%, yellow oil. $^1$H NMR (400 MHz, $CDCl_3$): δ 7.28 – 6.82 (m, 4H), 2.84 (s, 2H), 1.54 (s, 2H), 1.14 (s, 9H) ppm. $^{13}C\{^1H\}$ NMR (151 MHz, $CDCl_3$): δ 168.5, 163.0 (d, $J$ = 246 Hz), 137.1 (d, $J$ = 8 Hz), 129.9 (d, $J$ = 9 Hz), 121.6 (d, $J$ = 3 Hz), 113.7 (d, $J$ = 21 Hz), 112.9 (d, $J$ = 23 Hz), 80.8, 36.0, 31.1 (d, $J$ = 2 Hz), 28.1, 24.5 ppm. $^{19}F\{^1H\}$ NMR (376 MHz, $CDCl_3$): δ -114.0 (s) ppm. HRMS (ESI-TOF) *m/z*: $[M + Na]^+$ calcd for $C_{15}H_{17}FNaO_2$, 271.1110; found 271.1112.

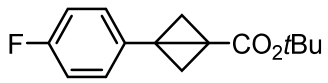

**Tert-butyl 3-(4-fluorophenyl)bicyclo[1.1.0]butane-1-carboxylate (17)**

General procedure D. Scale 0.054 mol. Yield: 12.4 g, 0.05 mol, 92%, colorless oil. $^1$H NMR (400 MHz, $CDCl_3$): δ 6.99 (dd, $J$ = 8.2, 5.4 Hz, 2H), 6.72 (t, $J$ = 8.6 Hz, 2H), 2.58 (s, 2H), 1.28 (s, 2H), 0.89 (s, 9H) ppm. $^{13}C\{^1H\}$ NMR (126 MHz, $CDCl_3$): δ 168.9, 162.1 (d, $J$ = 246 Hz), 130.0 (d, $J$ = 3 Hz), 127.5 (d, $J$ = 8 Hz), 115.4 (d, $J$ = 22 Hz), 80.7, 36.0 (d, $J$ = 3 Hz), 31.3, 28.1, 23.5 ppm. $^{19}F\{^1H\}$ NMR (376 MHz, $CDCl_3$): δ -116.2 (s) ppm. HRMS (ESI-TOF) *m/z*: $[M + Na]^+$ calcd for $C_{15}H_{17}FNaO_2$, 271.1110; found 271.1107.

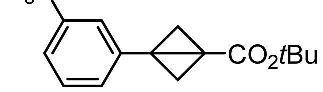

**Tert-butyl 3-(3-(trifluoromethyl)phenyl)bicyclo[1.1.0]butane-1-carboxylate (18)**

General procedure D. Scale 0.022 mol. Yield: 6.26 g, 0.021 mol, 97%, colorless oil. $^1$H NMR (400 MHz, $CDCl_3$): δ 7.59 – 7.36 (m, 4H), 2.92 (s, 2H), 1.61 (s, 2H), 1.13 (s, 9H) ppm. $^{13}C\{^1H\}$ NMR (126 MHz, $CDCl_3$): δ 168.3, 135.8, 130.9 (q, $J$ = 32 Hz), 129.2, 129.0, 124.2 (q, $J$ = 272 Hz), 123.4

(q, $J$ = 4 Hz), 122.5 (q, $J$ = 4 Hz), 81.0, 36.0, 30.7, 28.0, 24.7 ppm. $^{19}F\{^1H\}$ NMR (376 MHz, $CDCl_3$): δ -63.3 (s) ppm. HRMS (ESI-TOF) *m/z*: $[M + Na]^+$ calcd for $C_{16}H_{17}F_3NaO_2$, 321.1078; found 321.1068.

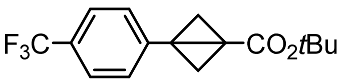

**Tert-butyl 3-(4-(trifluoromethyl)phenyl)bicyclo[1.1.0]butane-1-carboxylate (19)**

General procedure D. Scale 0.0089 mol. Yield: 2.50 g, 0.0084 mol, 94%, colorless oil. $^1H$ NMR (500 MHz, $CDCl_3$): δ 7.55 (d, $J$ = 8.2 Hz, 2H), 7.38 (d, $J$ = 8.2 Hz, 2H), 2.92 (s, 2H), 1.61 (s, 2H), 1.13 (s, 9H) ppm. $^{13}C\{^1H\}$ NMR (151 MHz, $CDCl_3$): δ 168.3, 138.9, 128.9 (q, $J$ = 32 Hz), 126.1, 125.3 (q, $J$ = 4 Hz), 124.2 (q, $J$ = 241 Hz), 81.0, 36.1, 30.7, 28.0, 25.2 ppm. $^{19}F\{^1H\}$ NMR (376 MHz, $CDCl_3$): δ -63.0 (s) ppm. HRMS (ESI-TOF) *m/z*: $[M + Na]^+$ calcd for $C_{16}H_{17}F_3NaO_2$, 321.1078; found 321.1079.

**General procedure E (compound 8 as an example)**

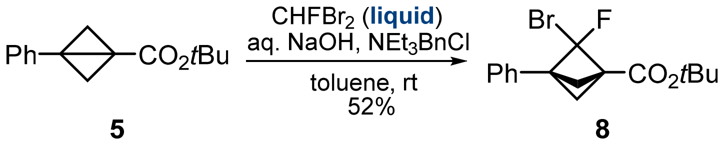


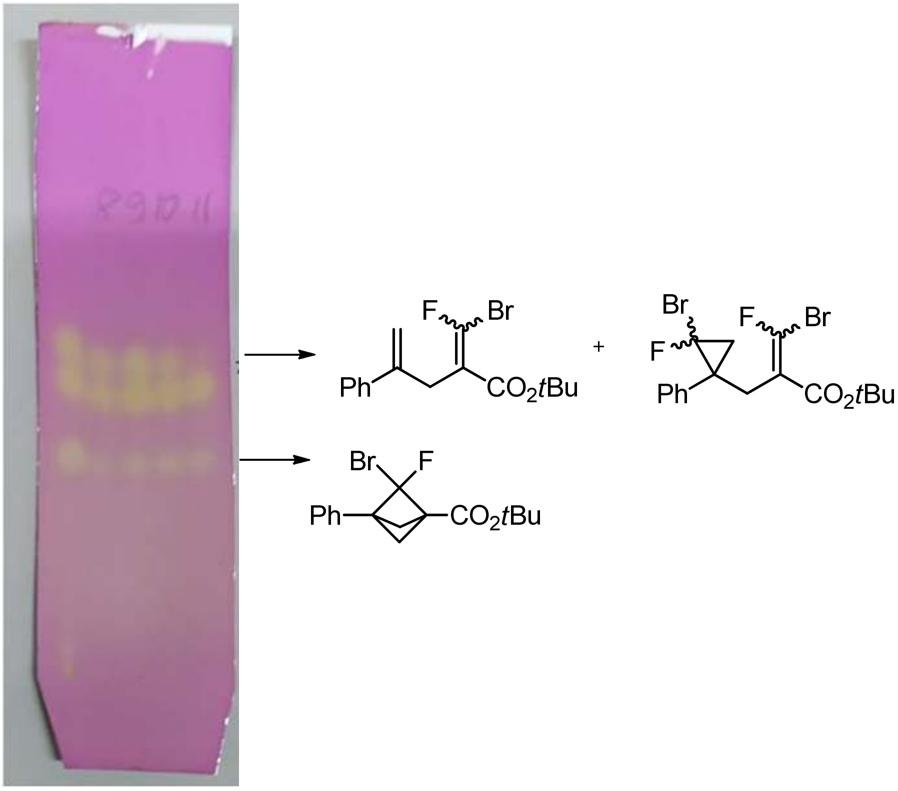

**Tert-butyl -2-bromo-2-fluoro-3-phenylbicyclo[1.1.1]pentane-1-carboxylate (8)**

To a solution of tert-butyl 3-phenylbicyclo[1.1.0]butane-1-carboxylate (28.0 g, 0.122 mol, 1.0 equiv) in toluene (400 mL) were added $CHFBr_2$ (70.0 g, 0.366 mol, 3.0 equiv) and TEBAC (0.61 g, 5 g per 1 mol) at room temperature. A 50% aqueous solution of NaOH (73.2 g, 1.83 mol, 15.0 equiv) was added to the mixture dropwise at 5-10 °C during ca. 30 min. The mixture was stirred at room temperature for 3 days. The solution was diluted with MTBE (300 mL), washed with $H_2O$ (200 mL), brine (20 × 200 mL), dried over $Na_2SO_4$, filtered through $SiO_2$ and concentrated under reduced pressure. The final product was purified by column chromatography (hexane:MTBE, 95:5). Yield: 21.48 g, 0.063 mol, 52%, colorless oil. $^1$H NMR (500 MHz, $CDCl_3$): δ 7.42 – 7.27 (m, 5H), 3.09 (d, $J$ = 10.4 Hz, 1H), 2.50 (s, 1H), 2.38 (dt, $J$ = 10.4, 4.0 Hz, 1H), 2.08 (dd, $J$ = 18.8, 3.3 Hz, 1H), 1.53 (s, 9H) ppm. $^{13}$C{$^1$H} NMR (126 MHz, $CDCl_3$): δ 164.8, 132.7, 128.6, 128.5, 127.2, 108.6 (d, $J$ = 311 Hz), 82.5, 56.6 (d, $J$ = 17 Hz), 52.8 (d, $J$ = 18 Hz), 46.5 (d, $J$ = 5 Hz), 46.4, 28.2 ppm. $^{19}$F{$^1$H} NMR (376 MHz, $CDCl_3$): δ -104.0 (s) ppm. HRMS (ESI-TOF) *m/z*: $[M + NH_4]^+$ calcd for $C_{16}H_{22}BrFNO_2$, 360.0797; found 360.0794.

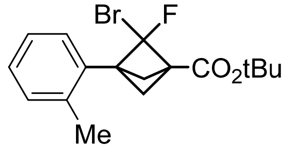


**Tert-butyl 2-bromo-2-fluoro-3-(*o*-tolyl)bicyclo[1.1.1]pentane-1-carboxylate (13a)**

General procedure E. Scale 0.082 mol. Yield: 20.0 g, 0.056 mol, 69%, colorless oil. $^1$H NMR (500 MHz, $CDCl_3$): δ 7.36 – 7.10 (m, 4H), 3.21 (d, $J$ = 10.5 Hz, 1H), 2.63 (d, $J$ = 2.3 Hz, 1H), 2.49 – 2.44 (m, 1H), 2.42 (s, 3H), 2.22 (dd, $J$ = 19.0, 3.4 Hz, 1H), 1.54 (s, 9H) ppm. $^{13}$C{$^1$H} NMR (126 MHz, $CDCl_3$): δ 164.7, 137.5, 131.2, 130.9, 128.9, 128.6, 126.0, 109.2 (d, $J$ = 312 Hz), 82.5, 57.5 (d, $J$ = 17 Hz), 53.3 (d, $J$ = 18 Hz), 47.3 (d, $J$ = 12 Hz), 46.9 (d, $J$ = 9 Hz), 28.2, 20.7 ppm. $^{19}$F{$^1$H} NMR (376 MHz, $CDCl_3$): δ -100.4 (s) ppm. HRMS (ESI-TOF) *m/z*: $[M + NH_4]^+$ calcd for $C_{17}H_{24}BrFNO_2$, 372.0974; found 372.0970.

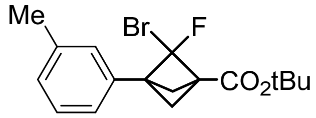


**Tert-butyl 2-bromo-2-fluoro-3-(*m*-tolyl)bicyclo[1.1.1]pentane-1-carboxylate (14a)**

General procedure E. Scale 0.10 mol. Yield: 20.0 g, 0.056 mol, 56%, colorless oil. $^1$H NMR (500 MHz, $CDCl_3$): δ 7.26 (t, $J$ = 7.8 Hz, 1H), 7.16 (d, $J$ = 7.6 Hz, 1H), 7.14 – 7.08 (m, 2H), 3.08 (d, $J$ = 10.4 Hz, 1H), 2.48 (t, $J$ = 2.4 Hz, 1H), 2.37 (s, 3H), 2.36 – 2.33 (m, 1H), 2.07 (dd, $J$ = 18.9, 3.4 Hz, 1H), 1.53 (s, 9H) ppm. $^{13}$C{$^1$H} NMR (126 MHz, $CDCl_3$): δ 164.9, 138.4, 132.7, 129.3, 128.6, 127.8, 124.3, 108.6 (d, $J$ = 311 Hz), 82.5, 56.6 (d, $J$ = 17 Hz), 52.8 (d, $J$ = 17.6 Hz), 46.5 (t, $J$ = 11 Hz), 28.2, 21.5 ppm. $^{19}$F{$^1$H} NMR (376 MHz, $CDCl_3$): δ -103.9 (s) ppm. HRMS (ESI-TOF) *m/z*: $[M + NH_4]^+$ calcd for $C_{17}H_{24}BrFNO_2$, 372.0974; found 372.0970.

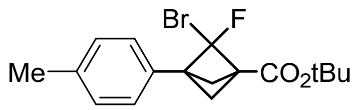

**Tert-butyl 2-bromo-2-fluoro-3-(*p*-tolyl)bicyclo[1.1.1]pentane-1-carboxylate (15a)**

General procedure E. Scale 0.0145 mol. Yield: 3.1 g, 0.0087 mol, 60%, colorless oil. $^{1}$H NMR (500 MHz, $CDCl_3$): δ 7.21 (d, *J* = 8.1 Hz, 2H), 7.18 (d, *J* = 8.0 Hz, 2H), 3.07 (d, *J* = 10.4 Hz, 1H), 2.47 (t, *J* = 2.5 Hz, 1H), 2.36 (s, 3H), 2.35 – 2.32 (m, 1H), 2.06 (dd, *J* = 18.9, 3.4 Hz, 1H), 1.52 (s, 9H) ppm. $^{13}$C{$^{1}$H} NMR (126 MHz, $CDCl_3$): δ 164.9, 138.4, 129.7, 129.3, 127.1, 108.7 (d, *J* = 311 Hz), 82.5, 56.5 (d, *J* = 17 Hz), 52.8 (d, *J* = 17 Hz), 46.5 (d, *J* = 8 Hz), 46.4 (d, *J* = 3 Hz), 28.2, 21.4 ppm. $^{19}$F{$^{1}$H} NMR (376 MHz, $CDCl_3$): δ -104.0 (s) ppm. HRMS (ESI-TOF) *m/z*: $[M + NH_4]^+$ calcd for $C_{17}H_{24}BrFNO_2$, 372.0974; found 372.0968.

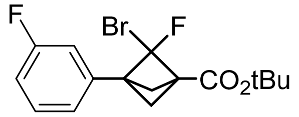

**Tert-butyl 2-bromo-2-fluoro-3-(3-fluorophenyl)bicyclo[1.1.1]pentane-1-carboxylate (16a)**

General procedure E. Scale 0.0332 mol. Yield: 7.78 g, 0.0161 mol, 50%, colorless oil. $^{1}$H NMR (500 MHz, $CDCl_3$): δ 7.37 – 7.29 (m, 1H), 7.09 (d, *J* = 7.6 Hz, 1H), 7.06 – 6.95 (m, 2H), 3.08 (d, *J* = 10.4 Hz, 1H), 2.50 (s, 1H), 2.37 (dt, *J* = 7.9, 3.7 Hz, 1H), 2.08 (dd, *J* = 18.7, 3.4 Hz, 1H), 1.52 (s, 9H) ppm. $^{13}$C{$^{1}$H} NMR (151 MHz, $CDCl_3$): δ 164.5, 162.9 (d, *J* = 247 Hz), 135.1 (d, *J* = 7 Hz), 130.3 (d, *J* = 8 Hz), 122.9 (d, *J* = 3 Hz), 115.6 (d, *J* = 21 Hz), 114.3 (d, *J* = 22 Hz), 108.2 (d, *J* = 311 Hz), 82.7, 56.0 (dd, *J* = 18, 2 Hz), 52.8 (d, *J* = 18 Hz), 46.6, 46.5 (d, *J* = 5 Hz), 28.2 ppm. $^{19}$F{$^{1}$H} NMR (376 MHz, $CDCl_3$): δ -104.2 (s, 1F), -112.9 (s, 1F), ppm. HRMS (ESI-TOF) *m/z*: $[M + NH_4]^+$ calcd for $C_{16}H_{21}BrF_2NO_2$, 376.0724; found 376.0720.

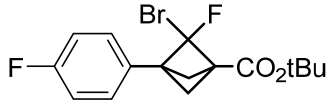

**Tert-butyl 2-bromo-2-fluoro-3-(4-fluorophenyl)bicyclo[1.1.1]pentane-1-carboxylate (17a)**

General procedure E. Scale 0.179 mol. Yield: 20.4 g, 0.057 mol, 32%, colorless oil. $^{1}$H NMR (400 MHz, $CDCl_3$): δ 7.26 (dd, *J* = 8.4, 5.4 Hz, 2H), 7.04 (t, *J* = 8.6 Hz, 2H), 3.05 (d, *J* = 10.4 Hz, 1H), 2.46 (s, 1H), 2.33 (dt, *J* = 7.9, 3.7 Hz, 1H), 2.05 (dd, *J* = 18.8, 3.4 Hz, 1H), 1.50 (s, 9H) ppm. $^{13}$C{$^{1}$H} NMR (126 MHz, $CDCl_3$): δ 164.6 (d, *J* = 1 Hz), 163.0 (d, *J* = 248 Hz), 129.0 (d, *J* = 8 Hz), 128.6 (d, *J* = 2 Hz), 115.7 (d, *J* = 22 Hz), 108.5 (d, *J* = 311 Hz), 82.6, 56.0 (d, *J* = 17 Hz), 52.8 (d, *J* = 17 Hz), 46.5, 46.5 (d, *J* = 7 Hz), 28.2 ppm. $^{19}$F{$^{1}$H} NMR (376 MHz, $CDCl_3$): δ -104.2 (s), -113.5 (s) ppm. HRMS (ESI-TOF) *m/z*: $[M + NH_4]^+$ calcd for $C_{16}H_{21}BrF_2NO_2$, 376.0724; found 376.07219.

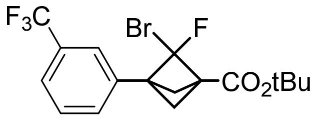

**Tert-butyl 2-bromo-2-fluoro-3-(3-(trifluoromethyl)phenyl)bicyclo[1.1.1]pentane-1-carboxylate (18a)**

General procedure E. Scale 0.0318 mol. Yield: 5.60 g, 0.0137 mol, 43%, colorless oil. $^{1}$H NMR (400 MHz, $CDCl_3$): δ 7.69 – 7.40 (m, 4H), 3.13 (d, *J* = 10.3 Hz, 1H), 2.54 (s, 1H), 2.47 – 2.37 (m, 1H), 2.12 (d, *J* = 18.7 Hz, 1H), 1.53 (s, 9H) ppm. $^{13}$C{$^{1}$H} NMR (101 MHz, $CDCl_3$): δ 164.5, 133.7, 131.2 (q, *J* = 33 Hz), 130.6, 129.3, 125.4 (q, *J* = 4 Hz), 124.1 (q, *J* = 4 Hz), 108.1 (d, *J* = 311 Hz), 82.8, 56.0 (d, *J* = 18 Hz), 52.9 (d, *J* = 18 Hz), 46.6, 46.5 (d, *J* = 4 Hz), 28.2 ppm. $^{19}$F{$^{1}$H} NMR (376 MHz, $CDCl_3$): δ -63.2 (s, 3F), -104.2 (s, 1F) ppm. HRMS (ESI-TOF) *m/z*: $[M + NH_4]^+$ calcd for $C_{17}H_{21}BrF_4NO_2$, 428.0671; found 428.0668.

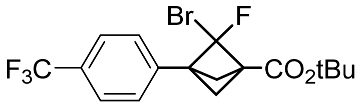

**Tert-butyl 2-bromo-2-fluoro-3-(4-(trifluoromethyl)phenyl)bicyclo[1.1.1]pentane-1-carboxylate (19a)**

General procedure E. Scale 0.021mol. Yield: 4.50 g, 0.011 mol, 52%, colorless oil. $^{1}$H NMR (400 MHz, $CDCl_3$): δ 7.63 (d, *J* = 8.1 Hz, 2H), 7.44 (d, *J* = 8.0 Hz, 2H), 3.12 (d, *J* = 10.4 Hz, 1H), 2.54 (t, *J* = 2.6 Hz, 1H), 2.41 (dt, *J* = 10.3, 3.9 Hz, 1H), 2.13 (dd, *J* = 18.6, 3.5 Hz, 1H), 1.53 (s, 9H) ppm. $^{13}$C{$^{1}$H} NMR (151 MHz, $CDCl_3$): δ 164.4, 136.6, 130.8 (q, *J* = 33 Hz), 127.7, 125.7 (q, *J* = 4 Hz), 124.1 (q, *J* = 272 Hz), 108.1 (d, *J* = 311 Hz), 82.8, 56.0 (d, *J* = 18 Hz), 52.9 (d, *J* = 18 Hz), 46.6 (d, *J* = 11 Hz), 46.5 (d, *J* = 6 Hz), 28.2 ppm. $^{19}$F{$^{1}$H} NMR (376 MHz, $CDCl_3$): δ -63.2 (s, 3F), -104.1 (s, 1F) ppm. HRMS (ESI-TOF) *m/z*: $[M + NH_4]^+$ calcd for $C_{17}H_{21}BrF_4NO_2$, 426.0692; found 426.0682.

**Preparation of Raney Ni[1]**

Ni-Al alloy (150 g) was added in small portions at a temperature of the reaction mixture not exceeding 50 ºC (careful, rapid evolution of hydrogen and exothermic reaction!) to a 3L glass flask with 20% aq. NaOH (1,5 L) under stirring. After the addition, the stirring was continued at 50 °C for 1 hour. The hot solution was decanted; the residue was washed with cold water (1L) and the solution was decanted again. 15% aq. NaOH (1.5 L) was added to the residue and the mixture was stirred for 2 hours at 90 °C. The hot solution was decanted. A new portion of aq. 15% NaOH (1.5 L) was added again, and the mixture was stirred for 2 hours at 90 °C. The hot solution was decanted. Raney Nickel was washed 10 times with cold water (0.5L each time; 5L in total) and

[1] A. A. Pavlic, H. Adkins. Preparation of a Raney Nickel Catalyst. *J. Am. Chem. Soc.* **1946**, *68*, 1471.

transferred to a glass vessel for storage. Raney Ni can be stored under water in a closed vessel at room temperature, and must be washed with ethanol (20 mL of EtOH for 1g of Raney Nickel) before using.

**General procedure F (compound 9 as an example)**

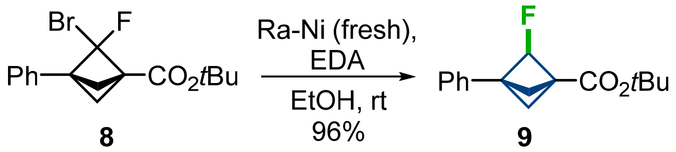


**Tert-butyl-2-fluoro-3-phenylbicyclo[1.1.1]pentane-1-carboxylate (9)**

To a solution of tert-butyl -2-bromo-2-fluoro-3-phenylbicyclo[1.1.1]pentane-1-carboxylate (68.0 g, 0.199 mol, 1.0 equiv) in EtOH (800 mL, ~ 4 mL per 0.001 mol) were added EDA (30.0 g, 0.50 mol, 2.5 equiv) and Raney Ni (69.65 g). The mixture was stirred under $H_2$ atmosphere at room temperature overnight. The mixture was filtered and concentrated under reduced pressure. The residue was dissolved in MTBE (500 mL), washed with water (100 mL), brine (100 mL), dried over $Na_2SO_4$, filtered and concentrated. The final product was purified by column chromatography (gradient, hexane, hexane:MTBE, 95:5). Yield: 50.05 g, 0.191 mol, 96%, colorless oil. $^1$H NMR (500 MHz, $CDCl_3$): δ 7.36 – 7.21 (m, 5H), 5.00 (dd, $J$ = 72.0, 6.5 Hz, 1H), 3.08 (d, $J$ = 9.6 Hz, 1H), 2.27 (dt, $J$ = 6.3, 2.3 Hz, 1H), 2.20 (dd, $J$ = 28.7, 3.0 Hz, 1H), 1.80 – 1.73 (m, 1H), 1.49 (s, 9H) ppm. $^{13}$C{$^1$H} NMR (126 MHz, $CDCl_3$): δ 167.2, 136.2, 128.5, 127.7, 126.7, 98.5 (d, $J$ = 240 Hz), 81.5, 47.7 (d, $J$ = 20 Hz), 45.6 (d, $J$ = 11 Hz), 44.7 (d, $J$ = 20 Hz), 41.3 (d, $J$ = 20 Hz), 28.2 ppm. $^{19}$F{$^1$H} NMR (376 MHz, $CDCl_3$): δ -183.6 (s) ppm. HRMS (ESI-TOF) *m/z*: $[M + NH_4]^+$ calcd for $C_{16}H_{23}FNO_2$, 280.1713; found 280.1713.

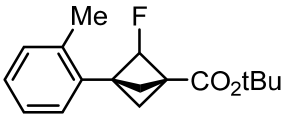


**Tert-butyl-2-fluoro-3-(*o*-tolyl)bicyclo[1.1.1]pentane-1-carboxylate (13b)**

General procedure F. Scale 0.0563 mol. Yield: 14.45 g, 0.0523 mol, 93%, colorless oil. $^1$H NMR (500 MHz, $CDCl_3$): δ 7.21 – 7.07 (m, 4H), 5.14 (dd, $J$ = 71.7, 6.5 Hz, 1H), 3.14 (d, $J$ = 9.6 Hz, 1H), 2.45 – 2.40 (m, 1H), 2.39 (s, 3H), 2.28 (dd, $J$ = 28.8, 3.1 Hz, 1H), 1.88 (d, $J$ = 9.6 Hz, 1H), 1.49 (s, 9H) ppm. $^{13}$C{$^1$H} NMR (126 MHz, $CDCl_3$): δ 167.2, 137.2, 134.0, 130.9, 128.3, 127.9, 126.0, 98.7 (d, $J$ = 241 Hz), 81.5, 48.6 (d, $J$ = 20 Hz), 45.9 (d, $J$ = 10 Hz), 45.6 (d, $J$ = 20 Hz), 41.8 (d, $J$ = 21 Hz), 28.2, 20.6 ppm. $^{19}$F{$^1$H} NMR (376 MHz, $CDCl_3$): δ -180.9 (s) ppm. HRMS (ESI-TOF) *m/z*: $[M + NH_4]^+$ calcd for $C_{17}H_{25}FNO_2$, 294.1869; found 294.1859.

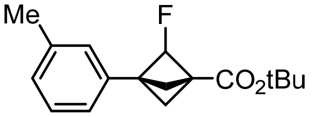


**Tert-butyl-2-fluoro-3-(*m*-tolyl)bicyclo[1.1.1]pentane-1-carboxylate (14b)**

General procedure F. Scale 0.0085 mol. Yield: 2.30 g, 0.0083 mol, 98%, colorless oil. $^{1}$H NMR (400 MHz, $CDCl_3$): δ 7.25 – 7.00 (m, 4H), 4.99 (dd, *J* = 72.1, 6.5 Hz, 1H), 3.07 (dd, *J* = 9.6, 2.1 Hz, 1H), 2.35 (s, 3H), 2.26 (dt, *J* = 5.6, 2.6 Hz, 1H), 2.19 (dd, *J* = 28.8, 3.1 Hz, 1H), 1.76 (dt, *J* = 12.1, 2.5 Hz, 1H), 1.49 (s, 9H) ppm. $^{13}$C{$^{1}$H} NMR (126 MHz, $CDCl_3$): δ 167.3, 138.2, 136.1, 128.5, 128.4, 127.3, 123.7, 98.5 (d, *J* = 240 Hz), 81.5, 47.7 (d, *J* = 20 Hz), 45.6 (d, *J* = 11 Hz), 44.7 (d, *J* = 20 Hz), 41.3 (d, *J* = 20 Hz), 28.2, 21.5 ppm. $^{19}$F{$^{1}$H} NMR (376 MHz, $CDCl_3$): δ -183.6 (s) ppm. HRMS (ESI-TOF) *m/z*: $[M + NH_4]^+$ calcd for $C_{17}H_{25}FNO_2$, 294.1869; found 294.1858.

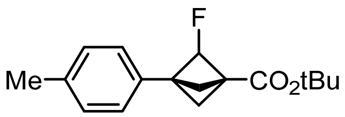


**Tert-butyl-2-fluoro-3-(*p*-tolyl)bicyclo[1.1.1]pentane-1-carboxylate (15b)**

General procedure F. Scale 0.0027 mol. Yield: 0.72 g, 0.00026 mol, 96%, colorless oil. $^{1}$H NMR (400 MHz, $CDCl_3$): δ 7.18 – 7.10 (m, 4H), 4.98 (dd, *J* = 72.1, 6.5 Hz, 1H), 3.06 (dd, *J* = 9.6, 2.1 Hz, 1H), 2.34 (s, 3H), 2.25 (dt, *J* = 5.4, 2.6 Hz, 1H), 2.18 (dd, *J* = 28.8, 3.1 Hz, 1H), 1.75 (dt, *J* = 12.2, 2.6 Hz, 1H), 1.49 (s, 9H) ppm. $^{13}$C{$^{1}$H} NMR (151 MHz, $CDCl_3$): δ 167.3, 137.4, 133.2, 129.2, 126.6, 98.6 (d, *J* = 240 Hz), 81.5, 46.6 (dd, *J* = 290, 16 Hz), 45.6 (d, *J* = 11 Hz), 44.7 (d, *J* = 20 Hz), 41.3 (d, *J* = 20 Hz), 28.2, 21.3 ppm. $^{19}$F{$^{1}$H} NMR (376 MHz, $CDCl_3$): δ -183.7 (s) ppm. HRMS (ESI-TOF) *m/z*: $[M + NH_4]^+$ calcd for $C_{17}H_{25}FNO_2$, 294.1869; found 294.1860.

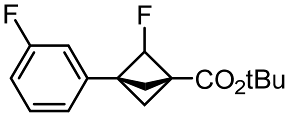


**Tert-butyl-2-fluoro-3-(3-fluorophenyl)bicyclo[1.1.1]pentane-1-carboxylate (16b)**

General procedure F. Scale 0.050 mol. Yield: 13.16 g, 0.047 mol, 94%, colorless oil. $^{1}$H NMR (400 MHz, $CDCl_3$): δ 7.22 (d, *J* = 7.3 Hz, 1H), 6.97 – 6.83 (m, 3H), 4.93 (dd, *J* = 71.9, 6.4 Hz, 1H), 3.01 (d, *J* = 9.3 Hz, 1H), 2.24 – 2.08 (m, 2H), 1.71 (d, *J* = 9.5 Hz, 1H), 1.43 (s, 9H) ppm. $^{13}$C{$^{1}$H} NMR (151 MHz, $CDCl_3$): δ 167.0, 163.0 (d, *J* = 247 Hz), 138.7 (d, *J* = 7 Hz), 130.2 (d, *J* = 8 Hz), 122.4 (d, *J* = 3 Hz), 114.7 (d, *J* = 21 Hz), 113.8 (d, *J* = 21 Hz), 98.4 (d, *J* = 241 Hz), 81.7, 47.3 (d, *J* = 20 Hz), 45.7 (d, *J* = 11 Hz), 44.7 (d, *J* = 20 Hz), 41.3 (d, *J* = 20 Hz), 28.2 ppm. $^{19}$F{$^{1}$H} NMR (376 MHz, $CDCl_3$): δ -113.4 (s, 1F), -183.6 (s, 1F) ppm. HRMS (ESI-TOF) *m/z*: $[M + NH_4]^+$ calcd for $C_{16}H_{22}F_2NO_2$, 298.1619; found 298.1617.

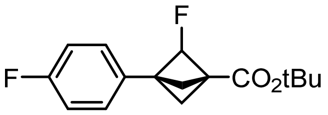


**Tert-butyl-2-fluoro-3-(4-fluorophenyl)bicyclo[1.1.1]pentane-1-carboxylate (17b)**

General procedure F. Scale 0.046 mol. Yield: 12.04 g, 0.043 mol, 94%, colorless oil. $^1$H NMR (400 MHz, $CDCl_3$): δ 7.18 (dd, *J* = 8.5, 5.4 Hz, 2H), 7.00 (t, *J* = 8.7 Hz, 2H), 4.97 (dd, *J* = 72.0, 6.5 Hz, 1H), 3.05 (dd, *J* = 9.6, 1.9 Hz, 1H), 2.27 – 2.23 (m, 1H), 2.19 (dd, *J* = 28.7, 3.1 Hz, 1H), 1.75 (dt, *J* = 12.1, 2.5 Hz, 1H), 1.48 (s, 9H) ppm. $^{13}$C{$^1$H} NMR (151 MHz, $CDCl_3$): δ 167.1 (d, *J* = 1 Hz), 162.4 (d, *J* = 246 Hz), 132.1 (d, *J* = 2 Hz), 128.3 (d, *J* = 8 Hz), 115.4 (d, *J* = 22 Hz), 98.5 (d, *J* = 240 Hz), 81.6, 47.1 (d, *J* = 20 Hz), 45.7 (d, *J* = 11 Hz), 44.6 (d, *J* = 20 Hz), 41.3 (d, *J* = 20 Hz), 28.2 ppm. $^{19}$F{$^1$H} NMR (376 MHz, $CDCl_3$): δ -115.0 (s, 1F), -183.7 (s, 1F) ppm. HRMS (ESI-TOF) *m/z*: $[M + NH_4]^+$ calcd for $C_{16}H_{22}F_2NO_2$, 298.1619; found 298.1618.

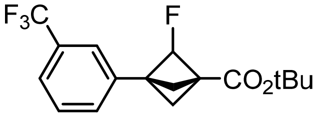


**Tert-butyl-2-fluoro-3-(3-(trifluoromethyl)phenyl)bicyclo[1.1.1]pentane-1-carboxylate (18b)**

General procedure F. Scale 0.0080 mol. Yield: 2.56 g, 0.0077 mol, 96%, colorless oil. $^1$H NMR (400 MHz, $CDCl_3$): δ 7.60 – 7.36 (m, 4H), 5.02 (dd, *J* = 71.8, 6.5 Hz, 1H), 3.11 (d, *J* = 9.6 Hz, 1H), 2.34 – 2.29 (m, 1H), 2.24 (dd, *J* = 28.5, 3.1 Hz, 1H), 1.81 (d, *J* = 9.6 Hz, 1H), 1.49 (s, 9H) ppm. $^{13}$C{$^1$H} NMR (126 MHz, $CDCl_3$): δ 166.8 (d, *J* = 1 Hz), 166.8 (d, *J* = 1 Hz), 137.2 (d, *J* = 1 Hz), 131.0 (q, *J* = 32 Hz), 130.1 (d, *J* = 1 Hz), 129.1, 124.5 (q, *J* = 4 Hz), 124.1 (q, *J* = 272 Hz), 123.5 (q, *J* = 4 Hz), 98.4 (d, *J* = 241 Hz), 81.8, 47.3 (d, *J* = 20 Hz), 45.7 (d, *J* = 11 Hz), 44.8 (d, *J* = 20 Hz), 41.3 (d, *J* = 20 Hz), 28.2 ppm. $^{19}$F{$^1$H} NMR (376 MHz, $CDCl_3$): δ -63.2 (s, 1F), -183.5 (s, 3F) ppm. HRMS (ESI-TOF) *m/z*: $[M + NH_4]^+$ calcd for $C_{17}H_{22}F_4NO_2$, 348.1587; found 348.1582.

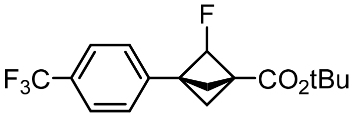


**Tert-butyl-2-fluoro-3-(4-(trifluoromethyl)phenyl)bicyclo[1.1.1]pentane-1-carboxylate (19b)**

General procedure F. Scale 0.0098 mol. Yield: 2.98 g, 0.009 mol, 92%, colorless oil. $^1$H NMR (400 MHz, $CDCl_3$): δ 7.58 (d, *J* = 8.0 Hz, 2H), 7.34 (d, *J* = 8.0 Hz, 2H), 5.02 (dd, *J* = 71.7, 6.5 Hz, 1H), 3.10 (dd, *J* = 9.6, 2.1 Hz, 1H), 2.34 – 2.29 (m, 1H), 2.24 (dd, *J* = 28.5, 3.2 Hz, 1H), 1.80 (dt, *J* = 12.1, 2.5 Hz, 1H), 1.49 (s, 9H) ppm. $^{13}$C{$^1$H} NMR (126 MHz, $CDCl_3$): δ 166.8, 140.1, 130.0 (d, *J* = 33 Hz), 127.1, 125.5 (q, *J* = 4 Hz), 124.2 (q, *J* = 272 Hz), 98.4 (d, *J* = 241 Hz), 81.8, 47.3 (d, *J* = 20 Hz), 45.8 (d, *J* = 11 Hz), 44.8 (q, *J* = 20 Hz), 41.3 (d, *J* = 20 Hz), 28.2 ppm. $^{19}$F{$^1$H} NMR (376

MHz, $CDCl_3$): δ -63.1 (s, 3F), -183.4 (s, 1F) ppm. HRMS (ESI-TOF) *m/z*: $[M + NH_4]^+$ calcd for $C_{17}H_{22}F_4NO_2$, 348.1587; found 348.1583.

**General procedure G (compound 12 as an example)**

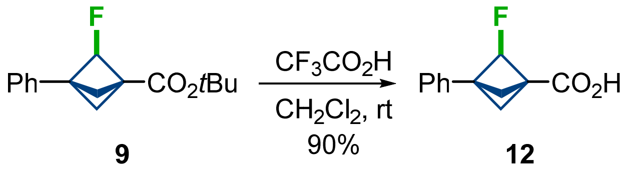


**2-Fluoro-3-phenylbicyclo[1.1.1]pentane-1-carboxylic acid (12)**

To a solution of tert-butyl-2-fluoro-3-phenylbicyclo[1.1.1]pentane-1-carboxylate (15.72 g, 0.06 mol, 1.0 equiv) in $CH_2Cl_2$ (70 mL) was added (27.36 g, 0.24 mol, 4.0 equiv). The mixture was stirred at room temperature for 2 h. The mixture was concentrated under reduced pressure. The solid residue was dissolved in $CH_2Cl_2$ (60 mL), washed with water (20 mL), dried over $Na_2SO_4$, filtered and concentrated. Yield: 11.12 g, 0.054 mol, 90%, white solid, m.p. = 151-152 °C. $^1$H NMR (500 MHz, $CDCl_3$): δ 7.36 – 7.26 (m, 3H), 7.23 (d, *J* = 7.0 Hz, 2H), 5.08 (dd, *J* = 71.5, 6.5 Hz, 1H), 3.17 (d, *J* = 9.6 Hz, 1H), 2.40 – 2.34 (m, 1H), 2.30 (dd, *J* = 28.4, 3.1 Hz, 1H), 1.86 (d, *J* = 9.5 Hz, 1H) ppm. $^{13}C\{^1H\}$ NMR (126 MHz, $CDCl_3$): δ 173.4, 135.6, 128.6, 127.9, 126.6, 98.4 (d, *J* = 242 Hz), 48.3 (d, *J* = 20 Hz), 45.9 (d, *J* = 11 Hz), 43.7 (d, *J* = 20 Hz), 41.4 (d, *J* = 19 Hz) ppm. $^{19}F\{^1H\}$ NMR (470 MHz, $CDCl_3$): δ -182.5 (dd, *J* = 72, 28. Hz) ppm. HRMS (ESI-TOF) *m/z*: $[M - H]^-$ calcd for $C_{12}H_{10}FO_2$, 205.0665; found 205.0673.

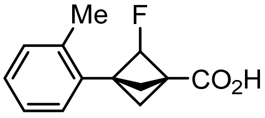


**2-Fluoro-3-(*o*-tolyl)bicyclo[1.1.1]pentane-1-carboxylic acid (13c)**

General procedure G. Scale 0.00148 mol. Yield: 0.28 g, 0.0013 mol, 88%, yellow solid, m.p. = 104-105 °C. $^1$H NMR (500 MHz, $CDCl_3$): δ 10.55 (br s, 1H), 7.25 – 7.12 (m, 4H), 5.23 (dd, *J* = 71.2, 6.5 Hz, 1H), 3.25 (dd, *J* = 9.6, 2.1 Hz, 1H), 2.56 – 2.50 (m, 1H), 2.41 (s, 3H), 2.40 (dd, *J* = 28.3, 3.2 Hz, 1H), 1.99 (dt, *J* = 7.2, 2.4 Hz, 1H) ppm. $^{13}C\{^1H\}$ NMR (126 MHz, $CDCl_3$): δ 173.5, 137.1, 133.4, 131.0, 128.3, 128.1, 126.1, 98.5 (d, *J* = 242 Hz), 49.2 (d, *J* = 20 Hz), 46.2 (d, *J* = 10 Hz), 44.5 (d, *J* = 21 Hz), 41.9 (d, *J* = 20 Hz), 20.6 ppm. $^{19}F\{^1H\}$ NMR (376 MHz, $CDCl_3$): δ -180.2 (s) ppm. LCMS $(M-H)^-$: 219. HRMS (ESI-TOF) *m/z*: $[M – H]^-$ calcd for $C_{13}H_{12}FO_2$, 219.0821; found 219.0832.

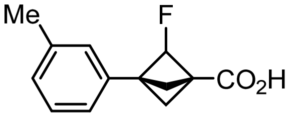


**2-Fluoro-3-(*m*-tolyl)bicyclo[1.1.1]pentane-1-carboxylic acid (14c)**

General procedure G. Scale 0.047 mol. Yield: 9.46 g, 0.043 mol, 92%, white solid, m.p. = 120-122 °C. $^1$H NMR (500 MHz, $CDCl_3$): δ 9.74 (br s, 1H), 7.24 (t, *J* = 7.5 Hz, 1H), 7.12 (d, *J* = 7.6 Hz, 1H), 7.08 – 7.02 (m, *J* = 8.6 Hz, 2H), 5.08 (dd, *J* = 71.6, 6.5 Hz, 1H), 3.17 (dd, *J* = 9.6, 2.3 Hz, 1H), 2.36 (s, 3H), 2.30 (dd, *J* = 28.4, 3.2 Hz, 1H), 1.86 (dt, *J* = 12.1, 2.4 Hz, 1H) ppm. $^{13}$C{$^1$H} NMR (126 MHz, $CDCl_3$): δ 173.7, 138.4, 135.6, 128.7, 128.5, 127.3, 123.7, 98.4 (d, *J* = 242 Hz), 48.3 (d, *J* = 20 Hz), 45.8 (d, *J* = 11 Hz), 43.7 (d, *J* = 20 Hz), 41.5 (d, *J* = 20 Hz), 21.5 ppm. $^{19}$F{$^1$H} NMR (376 MHz, $CDCl_3$): δ -182.9 (s) ppm. LCMS (M-H)$^-$: 219. HRMS (ESI-TOF) *m/z*: [M – H]$^-$ calcd for $C_{13}H_{12}FO_2$, 219.0821; found 219.0831.

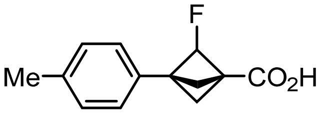


**2-Fluoro-3-(*p*-tolyl)bicyclo[1.1.1]pentane-1-carboxylic acid (15c)**

General procedure G. Scale 0.0122 mol. Yield: 2.60 g, 0.0118 mol, 97%, white solid. $^1$H NMR (500 MHz, $CDCl_3$): δ 10.17 (br s, 1H), 7.19 – 7.10 (m, 4H), 5.06 (dd, *J* = 71.6, 6.5 Hz, 1H), 3.15 (dd, *J* = 9.6, 2.2 Hz, 1H), 2.35 (s, 3H), 2.29 (dd, *J* = 28.4, 3.2 Hz, 1H), 1.84 (dt, *J* = 12.1, 2.5 Hz, 1H) ppm. $^{13}$C{$^1$H} NMR (126 MHz, $CDCl_3$): δ 173.6, 137.7, 132.6, 129.3, 126.5, 98.4 (d, *J* = 241 Hz), 48.2 (d, *J* = 20 Hz), 45.9 (d, *J* = 11 Hz), 43.7 (d, *J* = 21 Hz), 41.5 (d, *J* = 20 Hz), 21.3 ppm. $^{19}$F{$^1$H} NMR (376 MHz, $CDCl_3$): δ -183.1 (s) ppm. HRMS (ESI-TOF) *m/z*: [M + Na]$^+$ calcd for $C_{13}H_{13}FNaO_2$, 243.0797; found 243.0788.

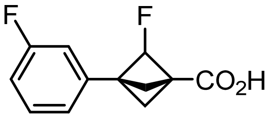


**2-Fluoro-3-(3-fluorophenyl)bicyclo[1.1.1]pentane-1-carboxylic acid (16c)**

General procedure G. Scale 0.071 mol. Yield: 15.46 g, 0.069 mol, 97%, beige solid, m.p. = 153-154 °C. $^1$H NMR (500 MHz, $CDCl_3$): δ 9.96 (br s, 1H), 7.36 – 7.27 (m, 1H), 7.09 – 6.81 (m, 3H), 5.07 (dd, *J* = 71.4, 6.4 Hz, 1H), 3.17 (d, *J* = 9.6 Hz, 1H), 2.40 – 2.35 (m, 1H), 2.32 (dd, *J* = 28.1, 3.0 Hz, 1H), 1.87 (d, *J* = 9.5 Hz, 1H) ppm. $^{13}$C{$^1$H} NMR (126 MHz, $CDCl_3$): δ 173.4, 163.0 (d, *J* = 247 Hz), 138.0 (d, *J* = 7 Hz), 130.3 (d, *J* = 8 Hz), 122.3 (d, *J* = 3 Hz), 114.9 (d, *J* = 21 Hz), 113.8 (d, *J* = 22 Hz), 98.3 (d, *J* = 242 Hz), 48.0 (d, *J* = 2 Hz), 47.8 (d, *J* = 2 Hz), 45.9 (d, *J* = 11 Hz), 43.7 (d, *J* = 20 Hz), 41.5 (d, *J* = 19 Hz) ppm. $^{19}$F{$^1$H} NMR (376 MHz, $CDCl_3$): δ -113.2 (s, 1F), -183.0 (s, 1F) ppm. LCMS (M-H)$^-$: 223. HRMS (ESI-TOF) *m/z*: [M – H]$^-$ calcd for $C_{12}H_9F_2O_2$, 223.0571; found 223.0576.

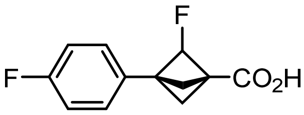

**2-Fluoro-3-(4-fluorophenyl)bicyclo[1.1.1]pentane-1-carboxylic acid (17c)**

General procedure G. Scale 0.0251 mol. Yield: 5.51 g, 0.0246 mol, 98%, white solid, m.p. = 167-168 °C. $^1$H NMR (400 MHz, $CDCl_3$): δ 9.59 (br s, 1H), 7.24 – 7.15 (m, 2H), 7.02 (t, *J* = 8.5 Hz, 2H), 5.06 (dd, *J* = 71.5, 6.4 Hz, 1H), 3.15 (d, *J* = 9.6 Hz, 1H), 2.38 – 2.25 (m, 2H), 1.85 (d, *J* = 9.5 Hz, 1H) ppm. $^{13}$C{$^1$H} NMR (126 MHz, $CDCl_3$): δ 173.5, 162.6 (d, *J* = 247 Hz), 131.5, 128.4 (d, *J* = 8 Hz), 115.6 (d, *J* = 22 Hz), 98.4 (d, *J* = 242 Hz), 47.8 (d, *J* = 20 Hz), 46.0 (d, *J* = 11 Hz), 43.6 (d, *J* = 20 Hz), 41.5 (d, *J* = 19 Hz) ppm. $^{19}$F{$^1$H} NMR (376 MHz, $CDCl_3$): δ -114.6 (s, 1F), -183.1 (s, 1F) ppm. LCMS $(M-H)^-$: 223. HRMS (ESI-TOF) *m/z*: $[M – H]^-$ calcd for $C_{12}H_9F_2O_2$, 223.0571; found 223.0577.

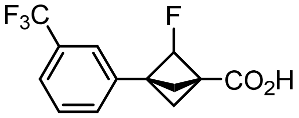

**2-Fluoro-3-(3-(trifluoromethyl)phenyl)bicyclo[1.1.1]pentane-1-carboxylic acid (18c)**

General procedure G. Scale 0.0092 mol. Yield: 2.41 g, 0.0088 mol, 96%, white solid, m.p. = 124-125 °C. $^1$H NMR (500 MHz, $CDCl_3$): 9.15 (br s, 1H), δ 7.57 – 7.43 (m, 4H), 5.11 (dd, *J* = 71.3, 6.5 Hz, 1H), 3.21 (d, *J* = 9.6 Hz, 1H), 2.43 – 2.40 (m, 1H), 2.36 (dd, *J* = 28.3, 2.9 Hz, 1H), 1.92 (d, *J* = 9.5 Hz, 1H) ppm. $^{13}$C{$^1$H} NMR (126 MHz, $CDCl_3$): δ 173.2, 136.6, 131.2 (q, *J* = 32 Hz), 130.1, 129.2, 124.8 (q, *J* = 4 Hz), 124.1 (q, *J* = 272 Hz), 123.5 (q, *J* = 4 Hz), 98.2 (d, *J* = 242 Hz), 47.9 (d, *J* = 20 Hz), 46.0 (d, *J* = 11 Hz), 43.8 (d, *J* = 20 Hz), 41.4 (d, *J* = 19 Hz) ppm. $^{19}$F{$^1$H} NMR (376 MHz, $CDCl_3$): δ -63.2 (s, 2F), -182.8 (s, 1F) ppm. LCMS $(M-H)^-$: 273. HRMS (ESI-TOF) *m/z*: $[M – H]^-$ calcd for $C_{13}H_9F_4O_2$, 273.0539; found 273.0541.

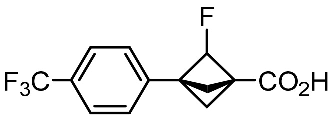

**2-Fluoro-3-(4-(trifluoromethyl)phenyl)bicyclo[1.1.1]pentane-1-carboxylic acid (19c)**

General procedure G. Scale 0.009 mol. Yield: 2.30 g, 0.0084 mol, 93%, white solid, m.p. = 179-180 °C. $^1$H NMR (400 MHz, DMSO-$d_6$): δ 12.87 (br s, 1H), 7.72 (d, *J* = 8.0 Hz, 2H), 7.48 (d, *J* = 7.9 Hz, 2H), 5.25 (dd, *J* = 72.7, 6.4 Hz, 1H), 2.93 (d, *J* = 9.4 Hz, 1H), 2.38 – 2.34 (m, 1H), 2.28 (dd, *J* = 29.7, 2.6 Hz, 1H), 1.87 (d, *J* = 9.6 Hz, 1H) ppm. $^{13}$C{$^1$H} NMR (151 MHz, DMSO-$d_6$): δ 168.5, 140.4, 128.2 (q, *J* = 32 Hz), 127.5, 125.3 (q, *J* = 4 Hz), 124.2 (q, *J* = 272 Hz), 97.9 (d, *J* = 238 Hz), 46.8 (d, *J* = 20 Hz), 44.5 (d, *J* = 11 Hz), 43.5 (d, *J* = 20 Hz), 40.8 (d, *J* = 20 Hz) ppm. $^{19}$F{$^1$H} NMR (376 MHz, DMSO-$d_6$): δ -61.5 (s, 1F), -182.6 (s, 3F) ppm. LCMS $(M-H)^-$: 273. HRMS (ESI-TOF) *m/z*: $[M – H]^-$ calcd for $C_{13}H_9F_4O_2$, 273.0539; found 273.0540.

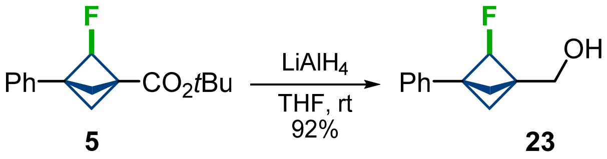


**2-Fluoro-3-phenylbicyclo[1.1.1]pentan-1-yl)methanol (23)**

To a solution of tert-butyl-2-fluoro-3-phenylbicyclo[1.1.1]pentane-1-carboxylate (2.60 g, 0.01 mol, 1.0 equiv) in dry THF (100 mL) was added $LiAlH_4$ (0.76 g, 0.02 mol, 2.0 equiv) in portions at 5 °C. The mixture was stirred for 5 h at room temperature and quenched with $H_2O$ (2 mL) and 2M $NaHSO_4$ (50 mL). The solution was concentrated under reduced pressure. The residue was extracted with $CHCl_3$ (2 × 50 mL). The combined organic layers were dried over $Na_2SO_4$, filtered and concentrated under reduced pressure. Yield: 1.77 g, 0.0092 mol, 92%, yellow oil. $^1H$ NMR (400 MHz, $CDCl_3$): δ 7.39 – 7.20 (m, 5H), 4.89 (dd, *J* = 73.4, 6.5 Hz, 1H), 3.80 (d, *J* = 3.3 Hz, 1H), 2.87 (d, *J* = 9.5 Hz, 1H), 2.08 – 2.00 (m, 1H), 1.95 (dd, *J* = 30.0, 2.8 Hz, 1H), 1.67 (br s, 1H), 1.53 (d, *J* = 9.6 Hz, 1H) ppm. $^{13}C\{^1H\}$ NMR (151 MHz, $CDCl_3$): δ 137.0, 128.4, 127.3, 126.6, 97.7 (d, *J* = 234 Hz), 60.0, 48.7 (d, *J* = 20 Hz), 45.7 (d, *J* = 20 Hz), 43.8 (d, *J* = 11 Hz), 38.4 (d, *J* = 22 Hz) ppm. $^{19}F\{^1H\}$ NMR (376 MHz, $CDCl_3$): δ -187.3 (s) ppm. GCMS (M): 192. HRMS (ESI-TOF) *m/z*: $[M + Na]^+$ calcd for $C_{12}H_{13}FNaO$, 215.0848; found 215.0846.

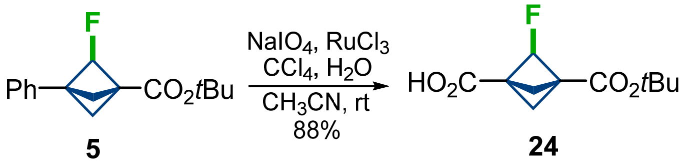


**3-(Tert-butoxycarbonyl)-2-fluorobicyclo[1.1.1]pentane-1-carboxylic acid (24)**

To a solution of tert-butyl-2-fluoro-3-phenylbicyclo[1.1.1]pentane-1-carboxylate (13.10 g, 0.05 mol, 1.0 equiv) in a mixture of 300 mL ($CH_3CN$), $CCl_4$ (300 mL) and $H_2O$ (500 mL) were added $NaIO_4$ (171.20 g, 0.80 mol, 16.0 equiv) and $RuCl_3$ (0.35 g). The mixture was stirred at room temperature for 96 h. The mixture was diluted with MTBE (500 mL) and 0.5M $NaHSO_4$ (1 L). The organic layer was separated and washed with $H_2O$ (3 × 300 mL), 0.5M $Na_2SO_3$ (3 × 200 mL), 2M $Na_2SO_3$ (50 mL), brine (2 × 200 mL), dried over $Na_2SO_4$, filtered and concentrated under reduced pressure. Yield: 10.12 g, 0.044 mol, 88%, white solid, m.p. = 132-133 °C. $^1H$ NMR (500 MHz, $CDCl_3$): δ 9.87 (br s, 1H), 5.02 (dd, *J* = 70.4, 6.5 Hz, 1H), 3.03 (dd, *J* = 9.5, 2.5 Hz, 1H), 2.34 – 2.30 (m, 1H), 2.25 (dd, *J* = 27.4, 3.2 Hz, 1H), 1.82 – 1.74 (m, 1H), 1.45 (s, 9H) ppm. $^{13}C\{^1H\}$ NMR (126 MHz, $CDCl_3$): δ 172.6, 166.1, 97.1 (d, *J* = 244 Hz), 82.3, 45.1 (d, *J* = 21 Hz), 44.9 (d, *J* = 10 Hz), 43.6 (d, *J* = 21 Hz), 41.4 (d, *J* = 18 Hz), 28.1 ppm. $^{19}F\{^1H\}$ NMR (376 MHz, $CDCl_3$): δ -181.6 (s) ppm. HRMS (ESI-TOF) *m/z*: $[M - H]^-$ calcd for $C_{11}H_{14}FO_4$, 229.0876; found 229.0882.

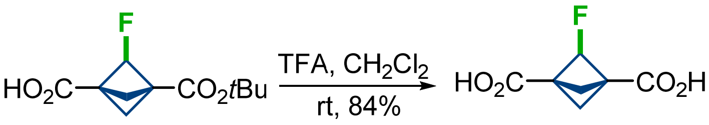


**24** **25**

**2-Fluorobicyclo[1.1.1]pentane-1,3-dicarboxylic acid (25)**

3-(Tert-butoxycarbonyl)-2-fluorobicyclo[1.1.1]pentane-1-carboxylic acid (3.30 g, 0.014 mol) was dissolved in TFA (30 mL). The solution was stirred at room temperature for 3 h and concentrated under reduced pressure. The residue was mashed in $CHCl_3$ (20 mL) and filtered. The final product was dried under reduced pressure. Yield: 2.10 g, 0.012 mol, 84%, beige solid, m.p. = 115-116 °C. $^1$H NMR (400 MHz, DMSO-d$_6$): δ 12.52 (br s, 2H), 5.09 (dd, $J$ = 71.9, 6.3 Hz, 1H), 2.76 (d, $J$ = 9.4 Hz, 1H), 2.28 – 2.10 (m, 2H), 1.70 (d, $J$ = 9.4 Hz, 1H) ppm. $^{13}$C{$^1$H} NMR (101 MHz, DMSO-d$_6$): δ 168.2, 97.0 (d, $J$ = 240 Hz), 44.0 (d, $J$ = 10 Hz), 43.6 (d, $J$ = 20 Hz) ppm. $^{19}$F{$^1$H} NMR (376 MHz, DMSO-d$_6$): δ -181.3 (s) ppm. LCMS (M-H)$^-$: 173. HRMS (ESI-TOF) *m/z*: [M + H]$^+$ calcd for $C_7H_8FO_4$, 175.0407; found 175.0404.

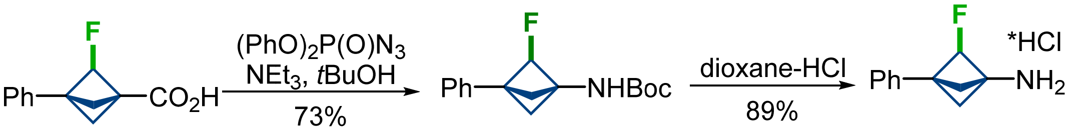


**12** **27**

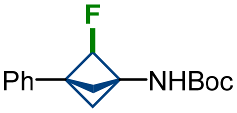


**Tert-butyl (2-fluoro-3-phenylbicyclo[1.1.1]pentan-1-yl)carbamate**

To a solution of 2-fluoro-3-phenylbicyclo[1.1.1]pentane-1-carboxylic acid (14.90 g, 0.0722 mol, 1.0 equiv) in *t*BuOH (250 mL) were added $Et_3N$ (8.80 g, 0.0867 mol, 1.2 equiv) and DPPA (22.0 g, 0.0795 mol, 1.1 equiv). The solution was heated under reflux for 24 h. The cold mixture was concentrated under reduced pressure. The residue was dissolved in MTBE (300 mL), washed with 1M $NaHCO_3$ (4 × 100 mL), brine (1 × 100 mL), dried over $Na_2SO_4$, filtered and concentrated under reduced pressure. The final product was purified by column chromatography (hexane:MTBE, 9:1). Yield: 14.68 g, 0.053 mol, 73%, yellow oil. $^1$H NMR (500 MHz, DMSO-d$_6$): δ 7.77 (br s, 1H), 7.34 – 7.22 (m, 5H), 5.04 (d, $J$ = 75.2 Hz, 1H), 2.78 (d, $J$ = 9.5 Hz, 2H), 2.21 (br s, 1H), 2.02 (dd, $J$ = 29.6, 2.4 Hz, 1H), 1.80 (s, 1H), 1.40 (s, 9H) ppm. $^{13}$C{$^1$H} NMR (151 MHz, DMSO-d$_6$): δ 154.4, 135.6, 128.3, 127.1, 126.6, 98.2 (d, $J$ = 237 Hz), 78.1, 50.3 (d, $J$ = 20 Hz), 45.9 (d, $J$ = 11 Hz), 45.0 (br s), 41.9 (d, $J$ = 21 Hz), 28.1 ppm. $^{19}$F{$^1$H} NMR (376 MHz, DMSO-d$_6$): δ -186.1 (s) ppm. HRMS (ESI-TOF) *m/z*: [M + Na]$^+$ calcd for $C_{16}H_{20}FNNaO_2$, 300.1376; found 300.1368.

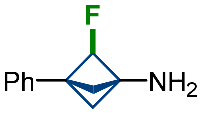


**2-Fluoro-3-phenylbicyclo[1.1.1]pentan-1-amine hydrochloride (27)**

A solution of tert-butyl (2-fluoro-3-phenylbicyclo[1.1.1]pentan-1-yl)carbamate (14.60 g, 0.0527 mol) in 4M HCl in dioxane (100 mL) was stirred for 5 h at room temperature. The mixture was diluted with $Et_2O$ (200 mL). The resulted precipitate was filtered, washed with $Et_2O$ (2 × 100 mL) and dried. Yield: 10.03 g, 0.047 mol, 89%, beige solid, m.p. = 174-175 °C. $^1$H NMR (500 MHz, DMSO-$d_6$): δ 9.38 (br s, 3H), 7.41 – 7.24 (m, 5H), 5.16 (dd, $J$ = 72.8, 6.5 Hz, 1H), 2.90 (dd, $J$ = 9.5, 1.4 Hz, 1H), 2.38 – 2.31 (m, 1H), 2.21 (dd, $J$ = 28.8, 2.9 Hz, 1H), 1.86 – 1.79 (m, 1H) ppm. $^{13}$C{$^1$H} NMR (126 MHz, DMSO-$d_6$): δ 133.9, 128.4, 127.6, 126.8, 97.5 (d, $J$ = 237 Hz), 47.6 (d, $J$ = 21 Hz), 45.6 (d, $J$ = 10 Hz), 44.9 (d, $J$ = 20 Hz), 40.6 (d, $J$ = 19 Hz) ppm. $^{19}$F{$^1$H} NMR (376 MHz, DMSO-$d_6$): δ -186.5 (s) ppm. HRMS (ESI-TOF) *m/z*: $[M + H]^+$ calcd for $C_{11}H_{13}FN$, 178.1032; found 178.1027.

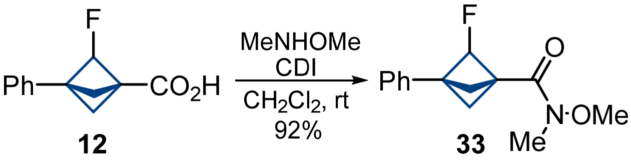


**2-Fluoro-*N*-methoxy-*N*-methyl-3-phenylbicyclo[1.1.1]pentane-1-carboxamide (33)**

To a solution of 2-fluoro-3-phenylbicyclo[1.1.1]pentane-1-carboxylic acid (0.50 g, 0.0024 mol, 1.0 equiv) in $CH_2Cl_2$ (10 mL) were added CDI (0.49 g, 0.003 mol, 1.25 equiv). The mixture was stirred for 2 h at room temperature. *N,O*-Dimethylhydroxylamine hydrochloride (0.26 g, 0.0027 mol, 1.1 equiv) was added in portions, and the solution was stirred at room temperature for 12 h. The mixture was washed with 1M $NaHSO_4$ (5 mL), 1M $NaHCO_3$ (5 mL), brine (5 mL), dried over $Na_2SO_4$, filtered and concentrated under reduced pressure. Yield: 0.55 g, 0.0022 mol, 92%, white solid. $^1$H NMR (400 MHz, $CDCl_3$): δ 7.36 – 7.18 (m, 5H), 5.09 (dd, $J$ = 72.1, 6.5 Hz, 1H), 3.72 (s, 3H), 3.21 (s, 3H), 3.16 (d, $J$ = 9.7 Hz, 1H), 2.43 – 2.35 (m, 1H), 2.26 (dd, $J$ = 28.9, 2.9 Hz, 1H), 1.88 – 1.81 (m, 1H) ppm. $^{13}$C{$^1$H} NMR (101 MHz, $CDCl_3$): δ 167.8, 167.8, 136.3, 128.5, 127.6, 126.6, 99.4 (d, $J$ = 240 Hz), 61.9, 48.5 (d, $J$ = 20 Hz), 46.6 (d, $J$ = 11 Hz), 44.8 (d, $J$ = 20 Hz), 41.7 (d, $J$ = 21 Hz), 32.4 ppm. $^{19}$F{$^1$H} NMR (376 MHz, $CDCl_3$): δ -181.6 (s) ppm. HRMS (ESI-TOF) *m/z*: $[M + H]^+$ calcd for $C_{14}H_{17}FNO_2$, 250.1243; found 250.1241.

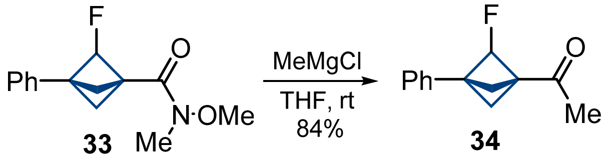


**1-(2-Fluoro-3-phenylbicyclo[1.1.1]pentan-1-yl)ethan-1-one (34)**

To a solution of 2-fluoro-*N*-methoxy-*N*-methyl-3-phenylbicyclo[1.1.1]pentane-1-carboxamide (0.40 g, 0.0016 mol, 1.0 equiv) in THF (10 mL) was added MeMgCl (3M, 1.1 mL, 0.0032 mol, 2.0 equiv) dropwise at 5 °C. The mixture was stirred for 2 h at room temperature. To the solution was added 2M $NaHSO_4$ (20 mL) dropwise, and the mixture was partially concentrated under reduced pressure. The residue was diluted with $CHCl_3$ (10 mL), and the organic layer was separated, dried over $Na_2SO_4$, filtered and concentrated under reduced pressure. Yield: 0.27 g, 0.0013 mol, 84%, colorless oil. $^1$H NMR (500 MHz, $CDCl_3$): δ 7.33 (t, *J* = 7.3 Hz, 2H), 7.28 (t, *J* = 7.2 Hz, 1H), 7.23 (d, *J* = 7.1 Hz, 2H), 5.03 (dd, *J* = 72.0, 6.5 Hz, 1H), 3.14 (dd, *J* = 9.4, 1.4 Hz, 1H), 2.31 – 2.26 (m, 1H), 2.22 (s, 3H), 2.22 – 2.15 (m, 1H), 1.79 – 1.74 (m, 1H) ppm. $^{13}$C{$^1$H} NMR (126 MHz, $CDCl_3$): δ 203.6, 135.9, 128.6, 127.8, 126.6, 98.7 (d, *J* = 240 Hz), 50.1 (d, *J* = 20 Hz), 47.6 (d, *J* = 20 Hz), 45.8 (d, *J* = 11 Hz), 40.5 (d, *J* = 20 Hz), 27.6 ppm. $^{19}$F{$^1$H} NMR (376 MHz, $CDCl_3$): δ -183.0 (s) ppm. HRMS (ESI-TOF) *m/z*: $[M + H]^+$ calcd for $C_{13}H_{14}FO$, 205.1029; found 205.1028.

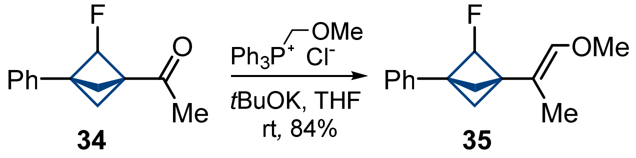


**2-Fluoro-1-(1-methoxyprop-1-en-2-yl)-3-phenylbicyclo[1.1.1]pentane (35)**

To a solution of (methoxymethyl)triphenylphosphonium chloride (20.00 g, 0.059 mol, 4.0 equiv) in THF (80 mL) was added *t*BuOK (6.60 g, 0.059 mol, 4.0 equiv) in portions at 0 °C. The mixture was stirred for 0.5 h, and a solution of 1-(2-fluoro-3-phenylbicyclo[1.1.1]pentan-1-yl)ethan-1-one (3.0 g, 0.0147 mol, 1.0 equiv) in THF (10 mL) was added dropwise. The mixture was stirred for 3 h at room temperature, and a sat. solution of $NH_4Cl$ (50 mL) was added dropwise. The solution was diluted with hexane (100 mL). The organic layer was separated, washed with brine (3 × 50 mL), dried over $Na_2SO_4$, filtered and concentrated under reduced pressure. Yield: 2.86 g, 0.0123 mol, 84%, white solid. Mixture of diastereomers (~ 1:2). $^1$H NMR (400 MHz, $CDCl_3$): δ 7.38 – 7.18 (m, 5H), 5.87 (s, 1H), 4.96 (dd, *J* = 73.3, 6.5 Hz), 4.81 (dd, *J* = 73.5, 6.5 Hz) 1H, 3.61 (s), 3.54 (s) 3H, 2.98 (d, *J* = 9.7 Hz), 2.86 (d, *J* = 9.6 Hz), 1H, 2.20 – 2.12 (m, 1H), 2.01 – 1.96 (m) 1H, 2.06 (dd, *J* = 30.3, 3.0 Hz), 1.89 (dd, *J* = 30.0, 2.9 Hz) 1H, 1.69 – 1.62 (m), 1.55 – 1.48 (m) 1H, 1.61 (s), 1.54 (s)

3H ppm. $^{13}C\{^1H\}$ NMR (101 MHz, $CDCl_3$): δ 145.0, 144.2, 137.7, 137.4, 128.4, 128.4, 127.2, 127.0, 126.6, 109.0, 108.1, 99.9 (d, $J$ = 237 Hz), 98.6 (d, $J$ = 237 Hz), 59.8, 59.7, 49.2 (d, $J$ = 20 Hz), 47.1 (d, $J$ = 20 Hz), 46.5 (d, $J$ = 11 Hz), 45.3 (d, $J$ = 20 Hz), 45.0 (d, $J$ = 11 Hz), 41.4 (d, $J$ = 23 Hz), 40.1 (d, $J$ = 23 Hz), 15.3, 10.4 ppm. $^{19}F\{^1H\}$ NMR (376 MHz, $CDCl_3$): δ -183.6 (s), -185.2 (s) ppm. HRMS (ESI-TOF) *m/z*: $[M + H]^+$ calcd for $C_{15}H_{18}FO$, 233.1342; found 233.1340.

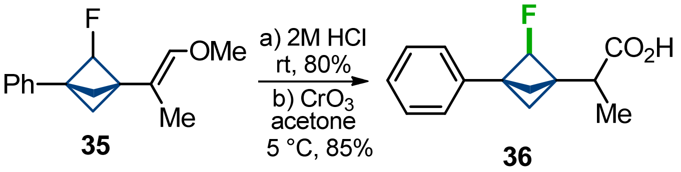


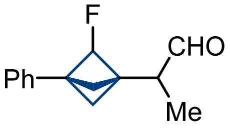


**2-(2-Fluoro-3-phenylbicyclo[1.1.1]pentan-1-yl)propanal**

Compound 2-fluoro-1-(1-methoxyprop-1-en-2-yl)-3-phenylbicyclo[1.1.1]pentane (1.0 g, 0.0043 mol) was dissolved in 2M HCl (20 mL). The mixture was stirred for 15 h at room temperature. The solution was diluted with 100 mL of $CHCl_3$. The organic layer was separated, dried over $Na_2SO_4$, filtered and concentrated under reduced pressure. The compound is unstable and was used for the next step without any purification. Yield: 0.75 g, 0.0034 mol, 80%, yellow oil. Mixture of diastereomers (~ 1:1). $^1H$ NMR (500 MHz, $CDCl_3$): δ 9.75 (s), 9.73 (s) 1H, 7.49 – 7.16 (m, 5H), 4.97 (t, $J$ = 6.9 Hz), 4.83 (t, $J$ = 6.9 Hz) 1H, 2.89 (t, $J$ = 7.4 Hz, 1H), 2.76 – 2.66 (m, 1H), 2.15 – 1.83 (m, 2H), 1.55 – 1.47 (m, 1H), 1.18 (d, $J$ = 7.1 Hz), 1.16 (d, $J$ = 7.1 Hz) 3H ppm. $^{19}F\{^1H\}$ NMR (376 MHz, $CDCl_3$): δ -185.1 (s), -185.3 (s) ppm. HRMS (ESI-TOF) *m/z*: $[M + Na]^+$ calcd for $C_{14}H_{15}FNaO$, 241.1005; found 241.1000.

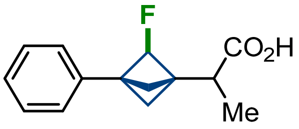


**2-(2-Fluoro-3-phenylbicyclo[1.1.1]pentan-1-yl)propanoic acid (36)**

To a solution of 2-(2-fluoro-3-phenylbicyclo[1.1.1]pentan-1-yl)propanal (1.0 g, 0.0046 mol) in acetone (100 mL) was added Jones reagent (30 mL) dropwise at 5 °C. The mixture was stirred for 0.5 h, and quenched with a sat. solution of $NaHSO_3$ (10 mL). The solution was filtered and concentrated under reduced pressure. The solid residue was dissolved in MTBE (20 mL) and extracted with 0.5M NaOH (2 × 10 mL). The combined aqueous layers were washed with $CH_2Cl_2$ (10 mL), acidified with 2M $NaHSO_4$, extracted with $CHCl_3$ (3 × 10 mL), dried over $Na_2SO_4$,

filtered and concentrated under reduced pressure. Yield: 0.91 g, 0.00391 mol, 85%, yellow solid. Mixture of diastereomers (~ 1:1). $^{1}H$ NMR (400 MHz, $CDCl_3$): δ 7.35 – 7.20 (m, 5H), 4.86 (dd, *J* = 73.1, 6.4 Hz, 1H), 2.93 – 2.76 (m, 2H), 2.09 – 1.83 (m, 2H), 1.50 (t, *J* = 8.7 Hz, 1H), 1.24 (d, *J* = 6.9 Hz), 1.23 (d, *J* = 7.0 Hz) 3H ppm. $^{13}C\{^{1}H\}$ NMR (126 MHz, $CDCl_3$): δ 180.2, 180.0, 136.8, 128.5, 127.3, 126.6, 99.1 (d, *J* = 65 Hz), 97.2 (d, *J* = 66 Hz), 47.9 (d, *J* = 4 Hz), 47.7 (d, *J* = 3 Hz), 45.8 (d, *J* = 11 Hz), 45.7 (d, *J* = 11 Hz), 44.6 (d, *J* = 11 Hz), 44.1 (d, *J* = 11 Hz), 38.9 (d, *J* = 12 Hz), 38.7 (d, *J* = 12 Hz), 38.3, 38.0, 13.5, 13.3 ppm. $^{19}F\{^{1}H\}$ NMR (376 MHz, $CDCl_3$): δ -186.68 (s), -186.74 (s) ppm. HRMS (ESI-TOF) *m/z*: $[M - H]^-$ calcd for $C_{14}H_{14}FO_2$, 233.0978; found 233.0985.

## Experimental *p*Ka

### Determination of $pK_a$

The $pK_a$ values of compounds were determined by a potentiometric titration method using the pH-meter (pH-340) with a ESL-43-07 glass electrode (*Gomel, Belarus*). In the applied pH-metric method, a solution of sample is titrated over a pH range in which it passes from being fully protonated to fully deprotonated. The negative *logarithm of dissociation constants* of carboxylic ($pK_{COOH}$) and amino groups ($pK_{NH3+}$) were calculated by analyzing the shape of the titration curve with using the graphical method (A. Albert, E. P. Sergeant. *Ionization constants of acids and bases*, Wiley, Inc., New York, **1962**) and a Hyperquad 2000 program. Theoretical *p*Ka values were predicted with using ACD/ChemSketch program.

A general titration procedure: the exact amounts of compounds were dissolved in an aqueous solution containing 0.1 M KCl to adjust an ionic strength. Then the prepared solutions were titrated with standardized base (0.06M KOH) at constant temperature (23 ºC).

## Acid-Base Titration

### Determination of assay

The assays of acids and amine hydrochlorides were determined by a potentiometric titration method using the automatic titrator (TitroLine 7000, SI Analytics SI Analytics GmbH) with pH combination electrode (A 7780, SI Analytics SI Analytics GmbH).

The assay of organic compound is determined as a function of the quantity of titrant (0.1 N potassium hydroxide solution) added until the endpoint is reached. The endpoint of the titration is determined by pH combination electrode immersed in the examined solution.

A general titration procedure:

The exact amounts of compounds were dissolved in a mixture of distilled water and isopropyl alcohol. These solutions were titrated with a 0.1 N potassium hydroxide solution using the automatic titrator. The assay of organic compound (X), in per cent, was calculated by the formula:

$$X = \frac{V \cdot c \cdot M}{m \cdot 1000} \cdot 100$$

where

V is the volume, in millilitres, of the 0.1 N potassium hydroxide solution used;

c is the concentration, in moles per litre, of potassium hydroxide solution;

M is the molar mass, in grams per moles, of the compound;

m is the mass, in grams, of the sample.

| | | | | | | | | |
|---|---|---|---|---|---|---|---|---|
| 17.12.2021 | | | | | K(NaOH) | | | |
| 272-173 | г/моль | | | | г | моль | | |
| *R2525573* | 188,222 | | | | 0,09411 | 0,0005 | | H2O |
| | | | | | 0,094 | 0,0005 | 47,5 | |

| Количество титранта (0.1 | pH | ΔpH | ΔV | ΔpH/ΔV | Стехиометричес [HA] | [A-] | pKa |
|---|---|---|---|---|---|---|---|
| 0 | 2,96 | 0 | 0 | | 0,01051 | 0 | |
| 0,5 | 3,29 | 0,33 | 0,5 | 0,66 | 0,00936 | 0,00115 | 4,19912 |
| 1 | 3,57 | 0,28 | 0,5 | 0,56 | 0,00824 | 0,00227 | 4,12916 |
| 1,5 | 3,79 | 0,22 | 0,5 | 0,44 | 0,00713 | 0,00338 | 4,11368 |
| 2 | 3,99 | 0,2 | 0,5 | 0,4 | 0,00605 | 0,00446 | 4,12204 |
| 2,5 | 4,19 | 0,2 | 0,5 | 0,4 | 0,00499 | 0,00552 | 4,14586 |
| 3 | 4,39 | 0,2 | 0,5 | 0,4 | 0,00395 | 0,00656 | 4,16944 |
| 3,5 | 4,62 | 0,23 | 0,5 | 0,46 | 0,00293 | 0,00758 | 4,20698 |
| 4 | 4,94 | 0,32 | 0,5 | 0,64 | 0,00193 | 0,00858 | 4,29187 |
| 4,5 | 5,55 | 0,61 | 0,5 | 1,22 | 0,00095 | 0,00956 | 4,54709 |
| 5 | 10,95 | 5,4 | 0,5 | 10,8 | -1E-05 | 0,01052 | ####### |
| 5,5 | 11,52 | 0,57 | 0,5 | 1,14 | -0,001 | 0,01146 | ####### |
| 6 | | -11,52 | 0,5 | -23,04 | -0,0019 | 0,01239 | ####### |
| 6,5 | | 0 | 0,5 | 0 | -0,0028 | 0,0133 | ####### |
| 7 | | 0 | 0,5 | 0 | -0,0037 | 0,01419 | ####### |
| 7,5 | | 0 | 0,5 | 0 | -0,0046 | 0,01507 | ####### |
| 8 | | 0 | 0,5 | 0 | -0,0054 | 0,01593 | ####### |
| 8,5 | | 0 | 0,5 | 0 | -0,0063 | 0,01677 | ####### |
| 9 | | 0 | 0,5 | 0 | -0,0071 | 0,0176 | ####### |
| 9,5 | | 0 | 0,5 | 0 | -0,0079 | 0,01842 | ####### |
| Среднее | | | | | | | 4,17227 |

**Результаты (расчеты)**

| | |
|---|---|
| 1 метод | |
| pKa | 4,17227 |
| 2 метод | |
| pHперехода | |
| pKa | 4,18 |

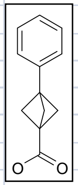


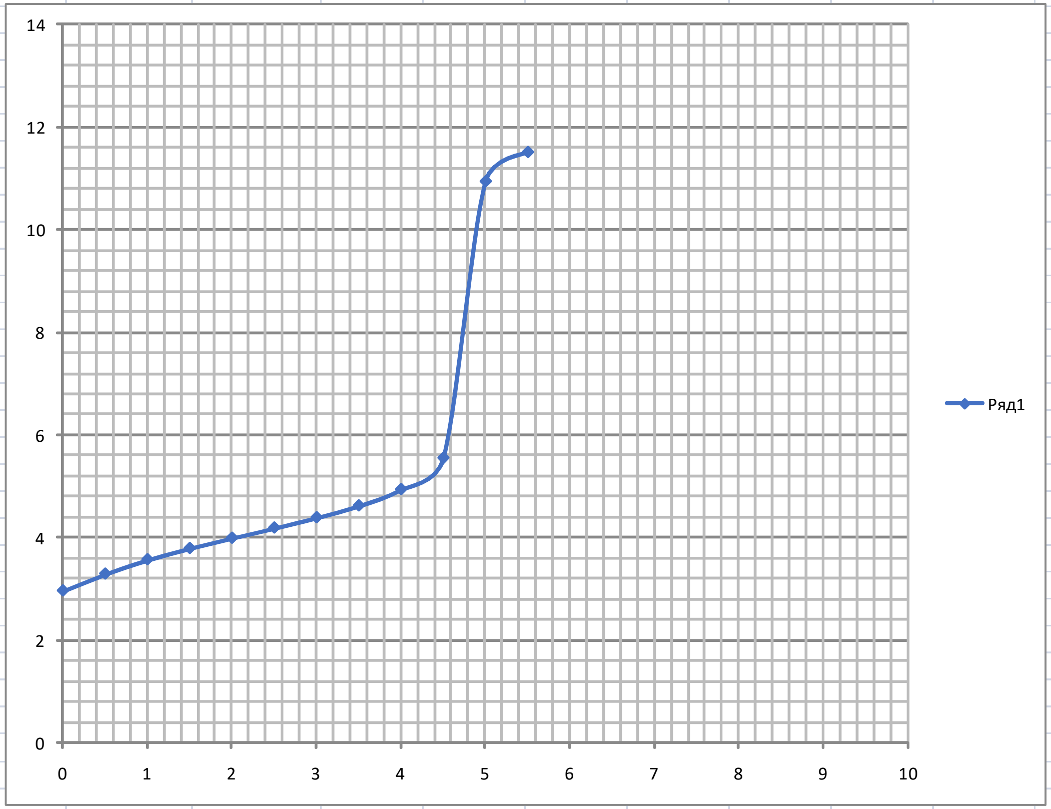

| | | | | | | | |
|---|---|---|---|---|---|---|---|
| 1.12.2021 | | | | | K(NaOH) | | |
| 0752-274 | г/моль | | | | г | моль | |
| R2598189 | 206,213 | | | | 0,10311 | 0,0005 | |
| | | | | | 0,1032 | 0,0005 | 47,5 |
| Количество титранта (0.1 | pH | ΔpH | ΔV | ΔpH/ΔV | Стехиометричес | | pKa |
| | | | | | [HA] | [A-] | |
| 0 | 2,58 | 0 | 0 | | 0,01054 | 0 | |
| 0,5 | 2,78 | 0,2 | 0,5 | 0,4 | 0,00938 | 0,00116 | 3,6893 |
| 1 | 2,98 | 0,2 | 0,5 | 0,4 | 0,00826 | 0,00228 | 3,53983 |
| 1,5 | 3,16 | 0,18 | 0,5 | 0,36 | 0,00715 | 0,00339 | 3,48464 |
| 2 | 3,35 | 0,19 | 0,5 | 0,38 | 0,00607 | 0,00447 | 3,48328 |
| 2,5 | 3,53 | 0,18 | 0,5 | 0,36 | 0,00501 | 0,00553 | 3,48744 |
| 3 | 3,69 | 0,16 | 0,5 | 0,32 | 0,00397 | 0,00657 | 3,4715 |
| 3,5 | 3,87 | 0,18 | 0,5 | 0,36 | 0,00295 | 0,00759 | 3,45982 |
| 4 | 4,1 | 0,23 | 0,5 | 0,46 | 0,00195 | 0,00859 | 3,45625 |
| 4,5 | 4,52 | 0,42 | 0,5 | 0,84 | 0,00097 | 0,00957 | 3,52605 |
| 5 | 10 | 5,48 | 0,5 | 10,96 | 0,00001 | 0,01053 | 6,97774 |
| 5,5 | 11,19 | 1,19 | 0,5 | 2,38 | -0,0009 | 0,01147 | ####### |
| 6 | 11,51 | 0,32 | 0,5 | 0,64 | -0,0019 | 0,0124 | ####### |
| 6,5 | 11,69 | 0,18 | 0,5 | 0,36 | -0,0028 | 0,01331 | ####### |
| 7 | 11,82 | 0,13 | 0,5 | 0,26 | -0,0037 | 0,0142 | ####### |
| 7,5 | 11,92 | 0,1 | 0,5 | 0,2 | -0,0045 | 0,01508 | ####### |
| 8 | | -11,92 | 0,5 | -23,84 | -0,0054 | 0,01594 | ####### |
| 8,5 | | 0 | 0,5 | 0 | -0,0062 | 0,01678 | ####### |
| 9 | | 0 | 0,5 | 0 | -0,0071 | 0,01761 | ####### |
| 9,5 | | 0 | 0,5 | 0 | -0,0079 | 0,01843 | ####### |
| Среднее | | | | | | | 3,5109 |

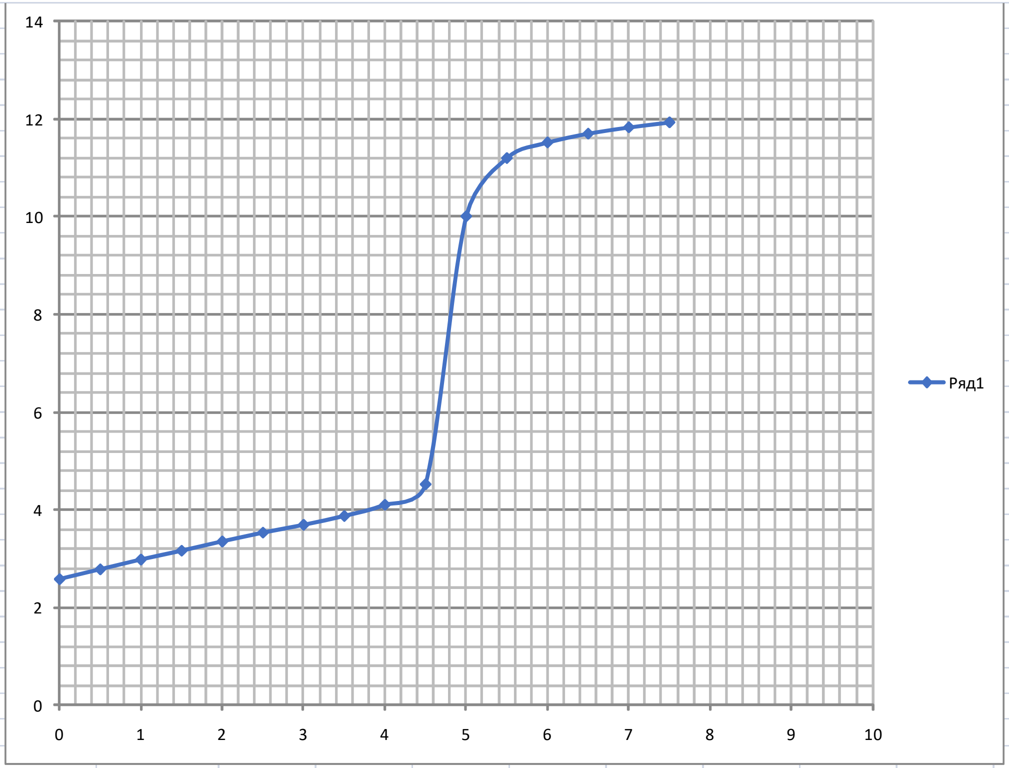


**Результаты (расчеты)**

| | |
|---|---|
| 1 метод | |
| pKa | 3,5109 |
| 2 метод | |
| pHперехода | |
| pKa | 3,6 |

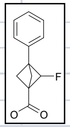

| | | | | | | | |
|---|---|---|---|---|---|---|---|
| 22.12.2021 | | | | | K(NaOH) | | |
| 663-109 | г/моль | | | | г | моль | |
| *R1323340* | 195,689 | | | | 0,09784 | 0,0005 | |
| | | | | | 0,098 | 0,0005 | 47,5 |
| Количество титранта (0.1 | pH | ΔpH | ΔV | ΔpH/ΔV | Стехиометричес | | pKa |
| | | | | | [HA] | [A-] | |
| 0 | 4,13 | 0 | 0 | | 0,01054 | 0 | |
| 0,5 | 7,11 | 2,98 | 0,5 | 5,96 | 0,00939 | 0,00115 | 8,02081 |
| 1 | 7,57 | 0,46 | 0,5 | 0,92 | 0,00826 | 0,00228 | 8,12846 |
| 1,5 | 7,85 | 0,28 | 0,5 | 0,56 | 0,00716 | 0,00338 | 8,1756 |
| 2 | 8,07 | 0,22 | 0,5 | 0,44 | 0,00608 | 0,00446 | 8,20427 |
| 2,5 | 8,26 | 0,19 | 0,5 | 0,38 | 0,00502 | 0,00552 | 8,21852 |
| 3 | 8,45 | 0,19 | 0,5 | 0,38 | 0,00398 | 0,00656 | 8,23278 |
| 3,5 | 8,65 | 0,2 | 0,5 | 0,4 | 0,00296 | 0,00758 | 8,24145 |
| 4 | 8,88 | 0,23 | 0,5 | 0,46 | 0,00196 | 0,00858 | 8,23861 |
| 4,5 | 9,19 | 0,31 | 0,5 | 0,62 | 0,00098 | 0,00956 | 8,20063 |
| 5 | 9,81 | 0,62 | 0,5 | 1,24 | 0,00002 | 0,01052 | 7,08889 |
| 5,5 | 10,99 | 1,18 | 0,5 | 2,36 | -0,0009 | 0,01147 | ####### |
| 6 | 11,42 | 0,43 | 0,5 | 0,86 | -0,0019 | 0,01239 | ####### |
| 6,5 | 11,64 | 0,22 | 0,5 | 0,44 | -0,0028 | 0,0133 | ####### |
| 7 | 11,78 | 0,14 | 0,5 | 0,28 | -0,0037 | 0,0142 | ####### |
| 7,5 | 11,89 | 0,11 | 0,5 | 0,22 | -0,0045 | 0,01507 | ####### |
| 8 | 11,98 | 0,09 | 0,5 | 0,18 | -0,0054 | 0,01593 | ####### |
| 8,5 | | -11,98 | 0,5 | -23,96 | -0,0062 | 0,01678 | ####### |
| 9 | | 0 | 0,5 | 0 | -0,0071 | 0,01761 | ####### |
| 9,5 | | 0 | 0,5 | 0 | -0,0079 | 0,01842 | ####### |
| Среднее | | | | | | | 8,18457 |

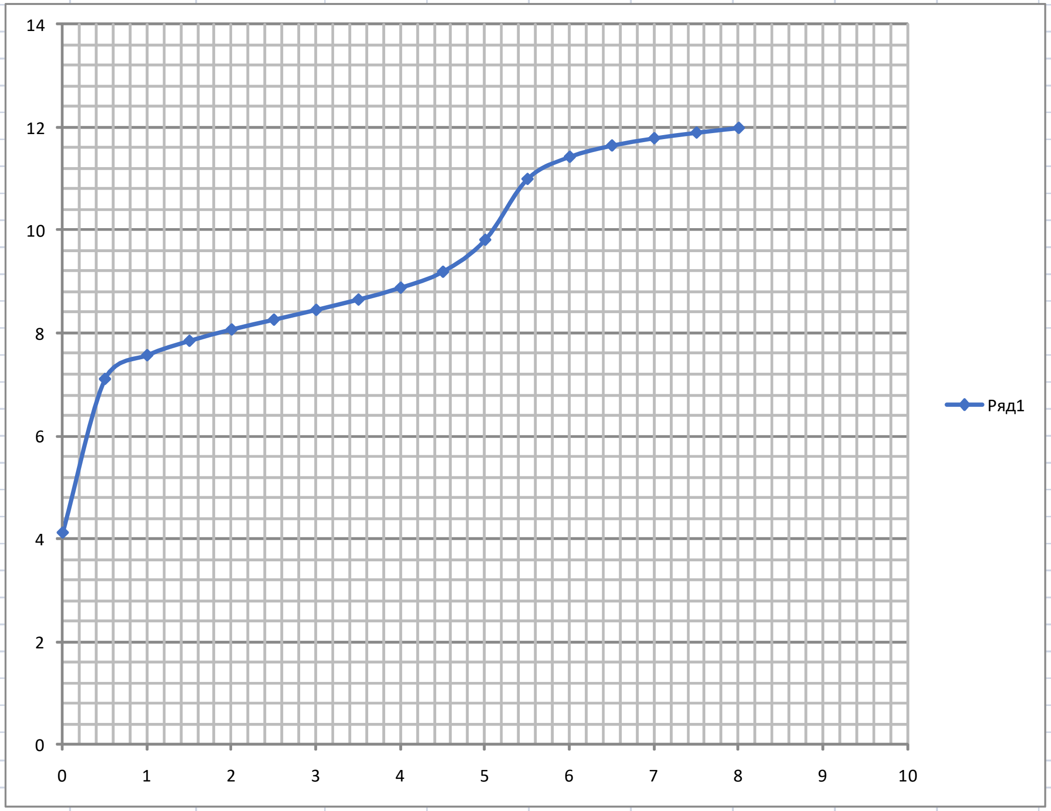


| | |
|---|---|
| **Результаты (расчеты)** | |
| 1 метод | |
| pKa | 8,18457 |
| 2 метод | |
| pHперехода | |
| pKa | 8,3 |

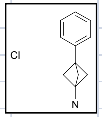

| | | | | | | | |
|---|---|---|---|---|---|---|---|
| 29.11.2021 | | | | | K(NaOH) | | |
| 2373-685 | г/моль | | | | г | моль | |
| R2711410 | 213,679 | | | | 0,10684 | 0,0005 | |
| | | | | | 0,1064 | 0,0005 | 47,5 |
| Количество титранта (0.1 | pH | ΔpH | ΔV | ΔpH/ΔV | Стехиометричес | | pKa |
| | | | | | [HA] | [A-] | |
| 0 | 4,14 | 0 | 0 | | 0,01048 | 0 | |
| 0,5 | 5,58 | 1,44 | 0,5 | 2,88 | 0,00933 | 0,00115 | 6,48805 |
| 1 | 5,93 | 0,35 | 0,5 | 0,7 | 0,00821 | 0,00227 | 6,48774 |
| 1,5 | 6,18 | 0,25 | 0,5 | 0,5 | 0,0071 | 0,00338 | 6,50195 |
| 2 | 6,39 | 0,21 | 0,5 | 0,42 | 0,00602 | 0,00446 | 6,51997 |
| 2,5 | 6,57 | 0,18 | 0,5 | 0,36 | 0,00496 | 0,00552 | 6,52331 |
| 3 | 6,76 | 0,19 | 0,5 | 0,38 | 0,00392 | 0,00656 | 6,53618 |
| 3,5 | 6,96 | 0,2 | 0,5 | 0,4 | 0,0029 | 0,00758 | 6,54256 |
| 4 | 7,21 | 0,25 | 0,5 | 0,5 | 0,0019 | 0,00858 | 6,55511 |
| 4,5 | 7,58 | 0,37 | 0,5 | 0,74 | 0,00092 | 0,00956 | 6,56319 |
| 5 | 9,04 | 1,46 | 0,5 | 2,92 | -4E-05 | 0,01052 | ####### |
| 5,5 | 10,98 | 1,94 | 0,5 | 3,88 | -0,001 | 0,01146 | ####### |
| 6 | 11,33 | 0,35 | 0,5 | 0,7 | -0,0019 | 0,01239 | ####### |
| 6,5 | 11,53 | 0,2 | 0,5 | 0,4 | -0,0028 | 0,0133 | ####### |
| 7 | 11,66 | 0,13 | 0,5 | 0,26 | -0,0037 | 0,01419 | ####### |
| 7,5 | 11,76 | 0,1 | 0,5 | 0,2 | -0,0046 | 0,01506 | ####### |
| 8 | | -11,76 | 0,5 | -23,52 | -0,0054 | 0,01592 | ####### |
| 8,5 | | 0 | 0,5 | 0 | -0,0063 | 0,01677 | ####### |
| 9 | | 0 | 0,5 | 0 | -0,0071 | 0,0176 | ####### |
| 9,5 | | 0 | 0,5 | 0 | -0,0079 | 0,01841 | ####### |
| Среднее | | | | | | | 6,52423 |

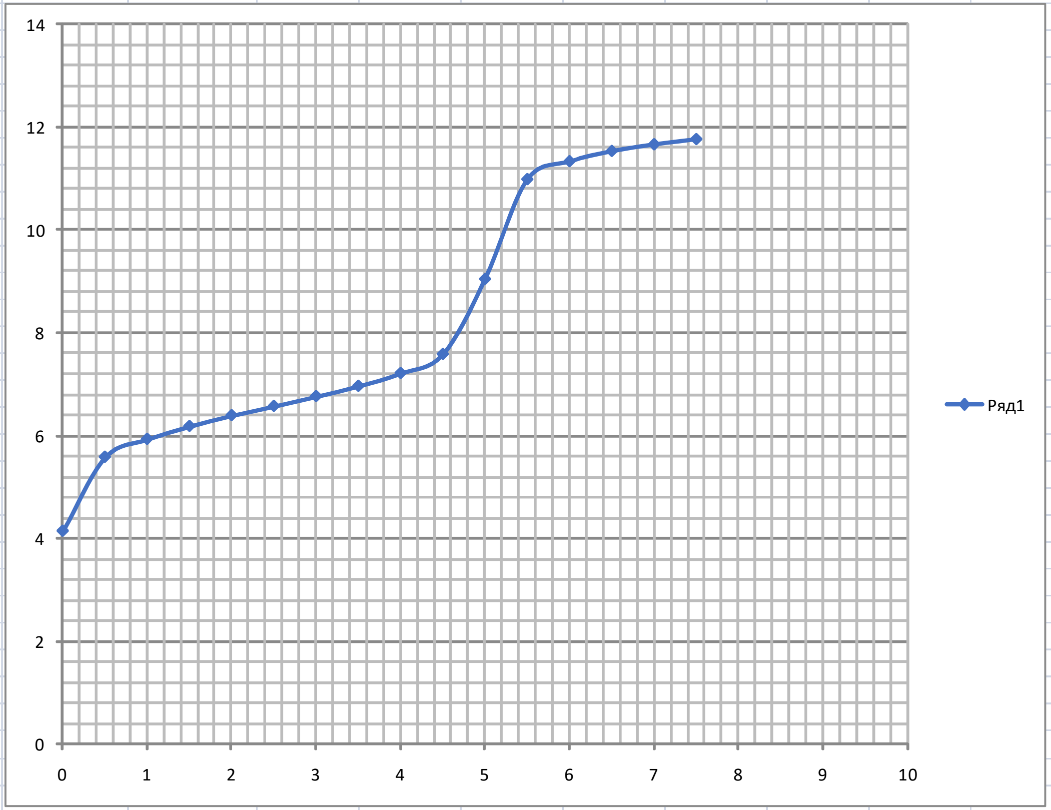


**Результаты (расчеты)**

| | |
|---|---|
| 1 метод | |
| pKa | 6,52423 |
| 2 метод | |
| pHперехода | |
| pKa | 6,6 |

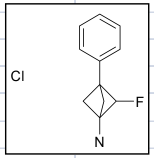

## Crystallographic data (X-Ray)

Crystals of compounds **12** and **27** suitable for X-Ray diffraction studies were obtained by a low evaporation of a solution of iPrOH-toluene (10:1). Diffraction data were collected at room temperature on an Xcallibur-3 diffractometer with graphite-monochromated Mo Kα radiation (λ = 0.71073 Å) operating in the w-scans mode. The structure was solved by direct methods and refined by the full-matrix least-squares technique in the anisotropic approximation for non-hydrogen atoms using the SHELXTL program package. Crystallographic data for all structures in this paper have been deposited at Cambridge Crystallographic Data Centre. CCDC numbers: 2161708 (**12**) and 2161709 (**27**). Copies of the data can be obtained, free of charge, on application to CCDC, 12 Union Road, Cambridge CB21EZ, UK, (fax: +44-(0)1223-336033 or e-mail: deposit@ccdc.cam.ac.uk).

Compound **12**

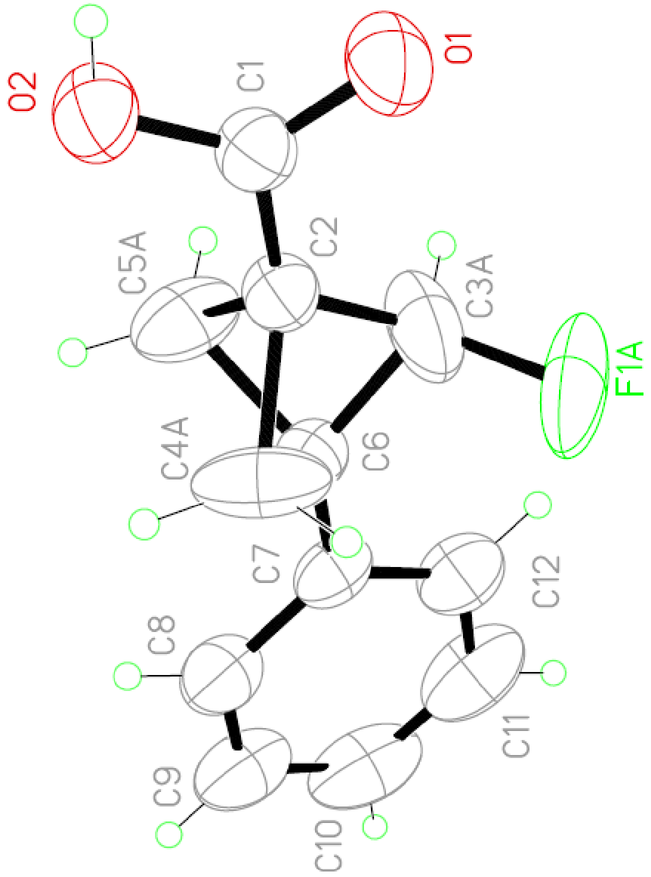


**Figure S1.** Molecular structure of **12** according to X-Ray diffraction data. Thermal ellipsoids are shown at 50% probability level.

**Crystal structure determination of 12**

**data_xr396**

_audit_creation_method 'SHELXL-2016/4'

_shelx_SHELXL_version_number '2016/4'

_chemical_formula_moiety 'C12 H11 F O2'

_chemical_formula_weight 206.21

_space_group_crystal_system monoclinic

_space_group_IT_number 14

_space_group_name_H-M_alt 'P 21/n'

_space_group_name_Hall '-P 2yn'

_cell_length_a 9.9265(7)

_cell_length_b 8.9981(6)

_cell_length_c 12.2213(10)

_cell_angle_alpha 90

_cell_angle_beta 107.313(5)

_cell_angle_gamma 90

_cell_volume 1042.15(14)

_cell_formula_units_Z 4

_cell_measurement_temperature 173(2)

_cell_measurement_reflns_used 1530

_cell_measurement_theta_min 2.86

_cell_measurement_theta_max 20.79

_exptl_crystal_description 'plate'

_exptl_crystal_colour 'colourless'

_exptl_crystal_density_diffrn 1.314

_exptl_crystal_F_000 432

_exptl_crystal_size_max 0.440

_exptl_crystal_size_mid 0.330

_exptl_crystal_size_min 0.080

_exptl_absorpt_coefficient_mu 0.100

_shelx_estimated_absorpt_T_min 0.957

_shelx_estimated_absorpt_T_max 0.992

_exptl_absorpt_correction_type multi-scan

_exptl_absorpt_correction_T_min 0.6530

_exptl_absorpt_correction_T_max 0.7452

_exptl_absorpt_process_details SADABS

_diffrn_ambient_temperature 173(2)

_diffrn_radiation_wavelength 0.71073

_diffrn_radiation_type MoK\a

_diffrn_source 'sealed tube'

_diffrn_measurement_device_type 'Bruker APEX-II CCD'

_diffrn_reflns_number 7457

_diffrn_reflns_av_unetI/netI 0.0368

_diffrn_reflns_av_R_equivalents 0.0350

_diffrn_reflns_limit_h_min -11

_diffrn_reflns_limit_h_max 11

_diffrn_reflns_limit_k_min -10

_diffrn_reflns_limit_k_max 10

_diffrn_reflns_limit_l_min -14

_diffrn_reflns_limit_l_max 14

_diffrn_reflns_theta_min 2.331

_diffrn_reflns_theta_max 25.318

_diffrn_reflns_theta_full 25.242

_diffrn_measured_fraction_theta_max 0.978

_diffrn_measured_fraction_theta_full 0.985

_diffrn_reflns_Laue_measured_fraction_max 0.978

_diffrn_reflns_Laue_measured_fraction_full 0.985

_diffrn_reflns_point_group_measured_fraction_max 0.978

_diffrn_reflns_point_group_measured_fraction_full 0.985

_reflns_number_total 1858

_reflns_number_gt 1212

_reflns_threshold_expression 'I > 2\s(I)'

_reflns_Friedel_coverage 0.000

Compound **27*HCl**

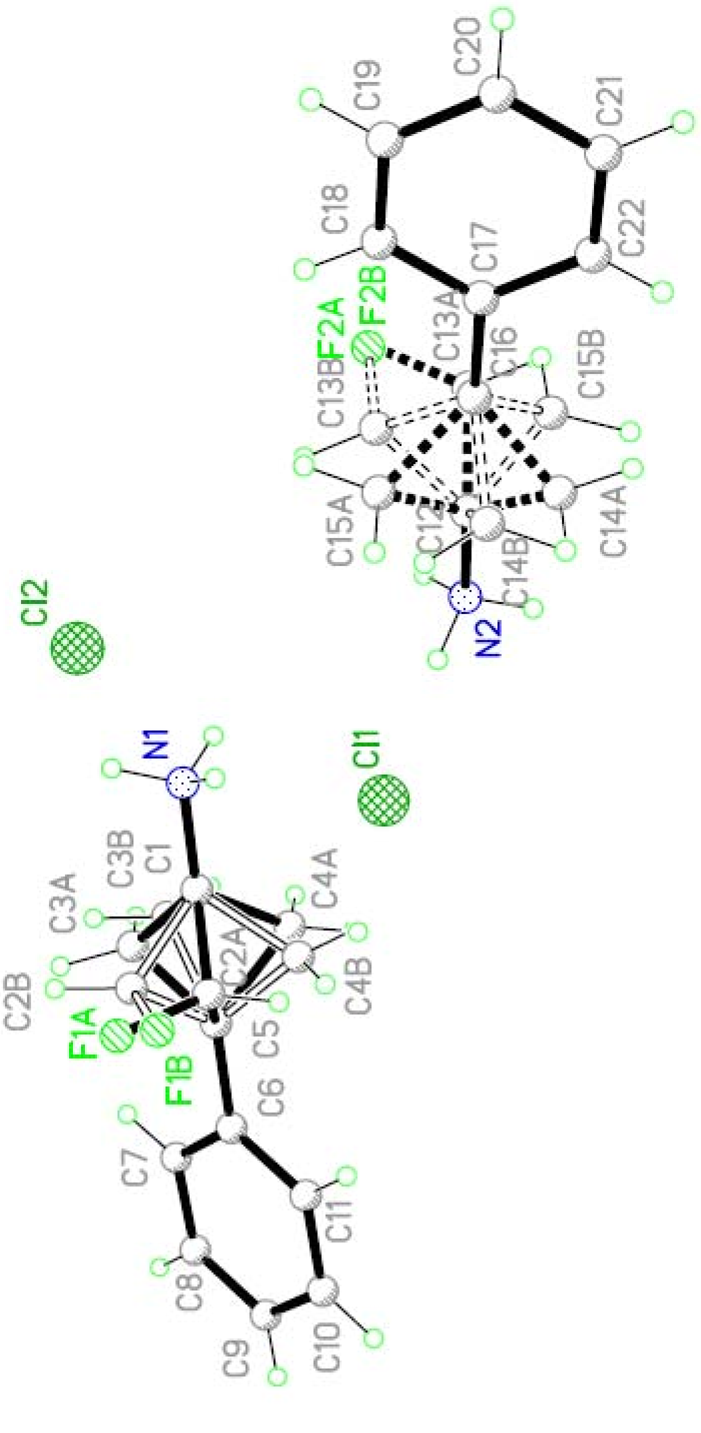


**Figure S2.** Molecular structure of **27*HCl** according to X-Ray diffraction data. Thermal ellipsoids are shown at 50% probability level.

**Crystal structure determination of 27*HCl**

**data_xr431**

_audit_creation_method 'SHELXL-2016/4'

_shelx_SHELXL_version_number '2016/4'

_chemical_formula_moiety 'C11 H13 F N, Cl'

_chemical_formula_sum 'C11 H13 Cl F N'

_chemical_formula_weight 213.67

_space_group_crystal_system orthorhombic

_space_group_IT_number 19

_space_group_name_H-M_alt 'P 21 21 21'

_space_group_name_Hall 'P 2ac 2ab'

_cell_length_a 6.0842(9)

_cell_length_b 10.7727(15)

_cell_length_c 32.893(4)

_cell_angle_alpha 90

_cell_angle_beta 90

_cell_angle_gamma 90

_cell_volume 2155.9(5)

_cell_formula_units_Z 8

_cell_measurement_temperature 173(2)

_cell_measurement_reflns_used 2466

_cell_measurement_theta_min 2.26

_cell_measurement_theta_max 20.56

_exptl_crystal_description block

_exptl_crystal_colour colourless

_exptl_crystal_density_diffrn 1.317

_exptl_crystal_F_000 896

_exptl_crystal_size_max 0.300

_exptl_crystal_size_mid 0.140

_exptl_crystal_size_min 0.100

_exptl_absorpt_coefficient_mu 0.328

_shelx_estimated_absorpt_T_min 0.908

_shelx_estimated_absorpt_T_max 0.968

_exptl_absorpt_correction_type multi-scan

_exptl_absorpt_correction_T_min 0.4918

_exptl_absorpt_correction_T_max 0.7454

_exptl_absorpt_process_details SADABS

_diffrn_ambient_temperature 173(2)

_diffrn_radiation_wavelength 0.71073

_diffrn_radiation_type MoK\a

_diffrn_source 'sealed tube'

_diffrn_measurement_device_type 'Bruker APEX-II CCD'

_diffrn_measurement_method '\f and \w scans'

_diffrn_reflns_number 17414

_diffrn_reflns_av_unetI/netI 0.0815

_diffrn_reflns_av_R_equivalents 0.0745

_diffrn_reflns_limit_h_min -7

_diffrn_reflns_limit_h_max 7

_diffrn_reflns_limit_k_min -13

_diffrn_reflns_limit_k_max 9

_diffrn_reflns_limit_l_min -40

_diffrn_reflns_limit_l_max 41

_diffrn_reflns_theta_min 2.260

_diffrn_reflns_theta_max 26.416

_diffrn_reflns_theta_full 25.242

_diffrn_measured_fraction_theta_max 0.997

_diffrn_measured_fraction_theta_full 0.998

_diffrn_reflns_Laue_measured_fraction_max 0.997

_diffrn_reflns_Laue_measured_fraction_full 0.998

_diffrn_reflns_point_group_measured_fraction_max 0.997

_diffrn_reflns_point_group_measured_fraction_full 0.999

_reflns_number_total 4434

_reflns_number_gt 3532

_reflns_threshold_expression 'I > 2\s(I)'

_reflns_Friedel_coverage 0.717

_reflns_Friedel_fraction_max 0.998

_reflns_Friedel_fraction_full 1.000

## Copies of $^1$H, $^{13}$C{$^1$H} and $^{19}$F{$^1$H} NMR spectra

### 3-Hydroxy-3-phenylcyclobutane-1-carboxylic acid

$^1$H NMR (500 MHz, DMSO-d$_6$)

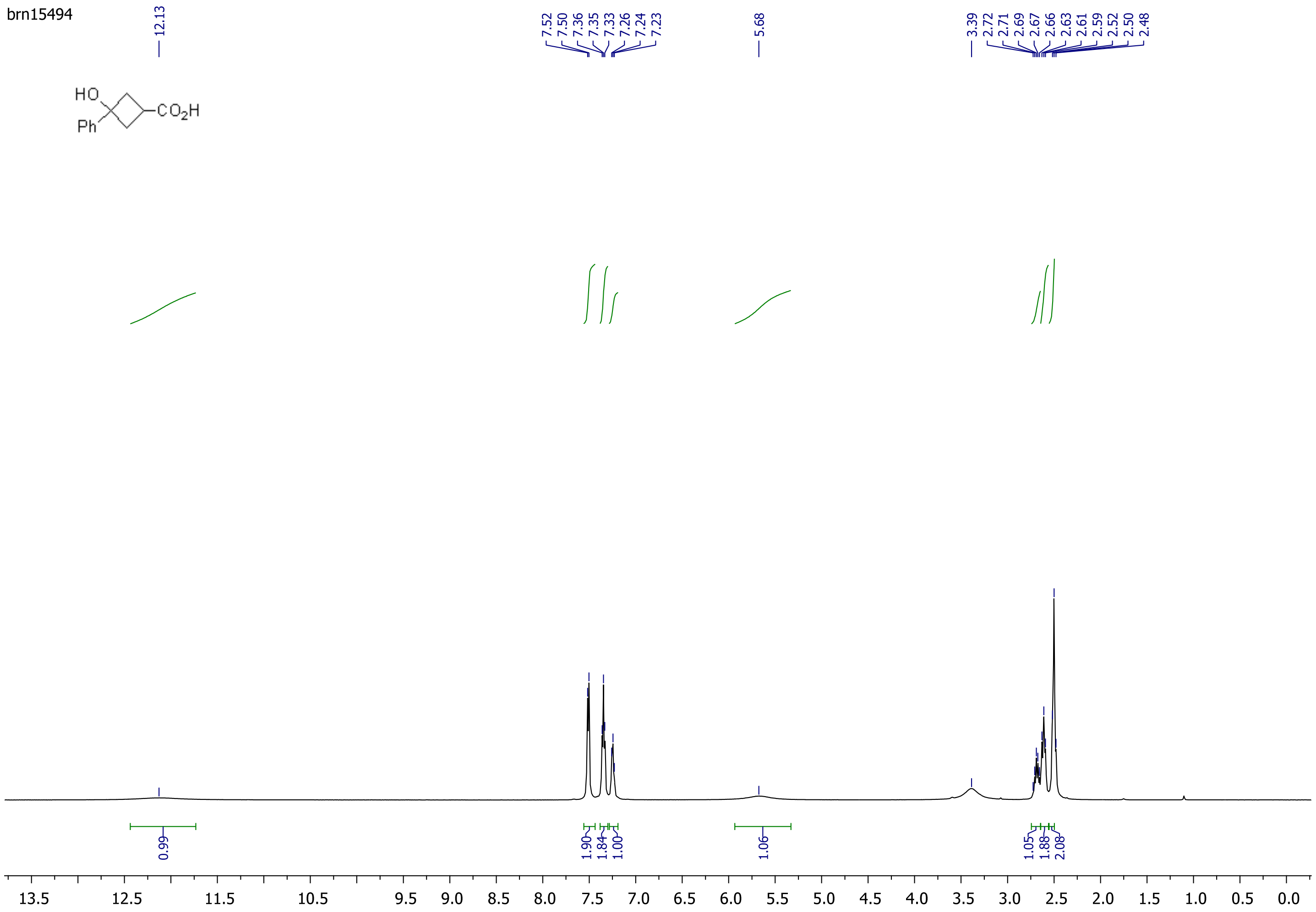

$^{13}C\ \{^{1}H\}$ NMR (126 MHz, DMSO-$d_6$)

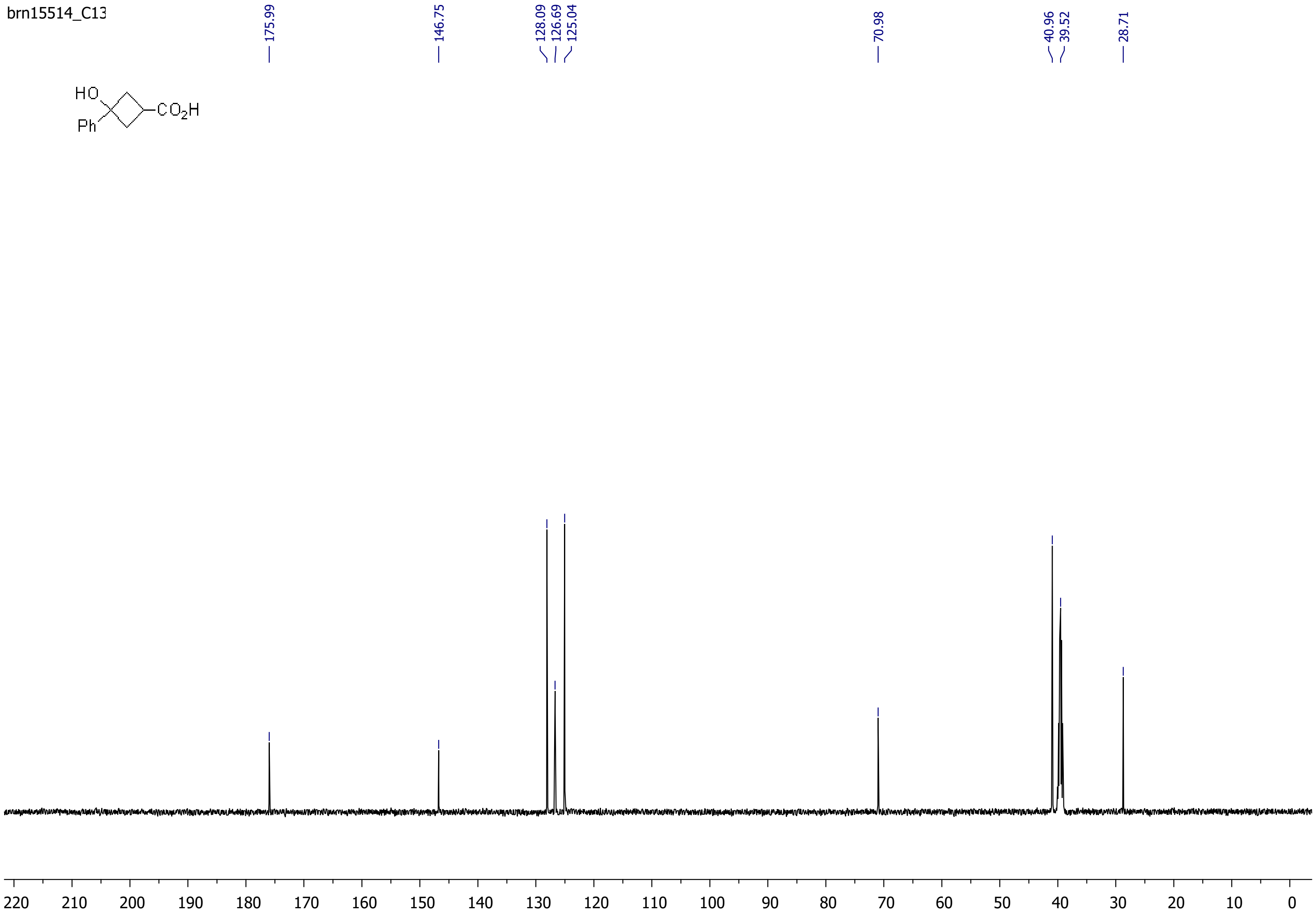

**3-Hydroxy-3-(o-tolyl)cyclobutane-1-carboxylic acid**

$^{1}H$ NMR (500 MHz, DMSO-$d_6$)

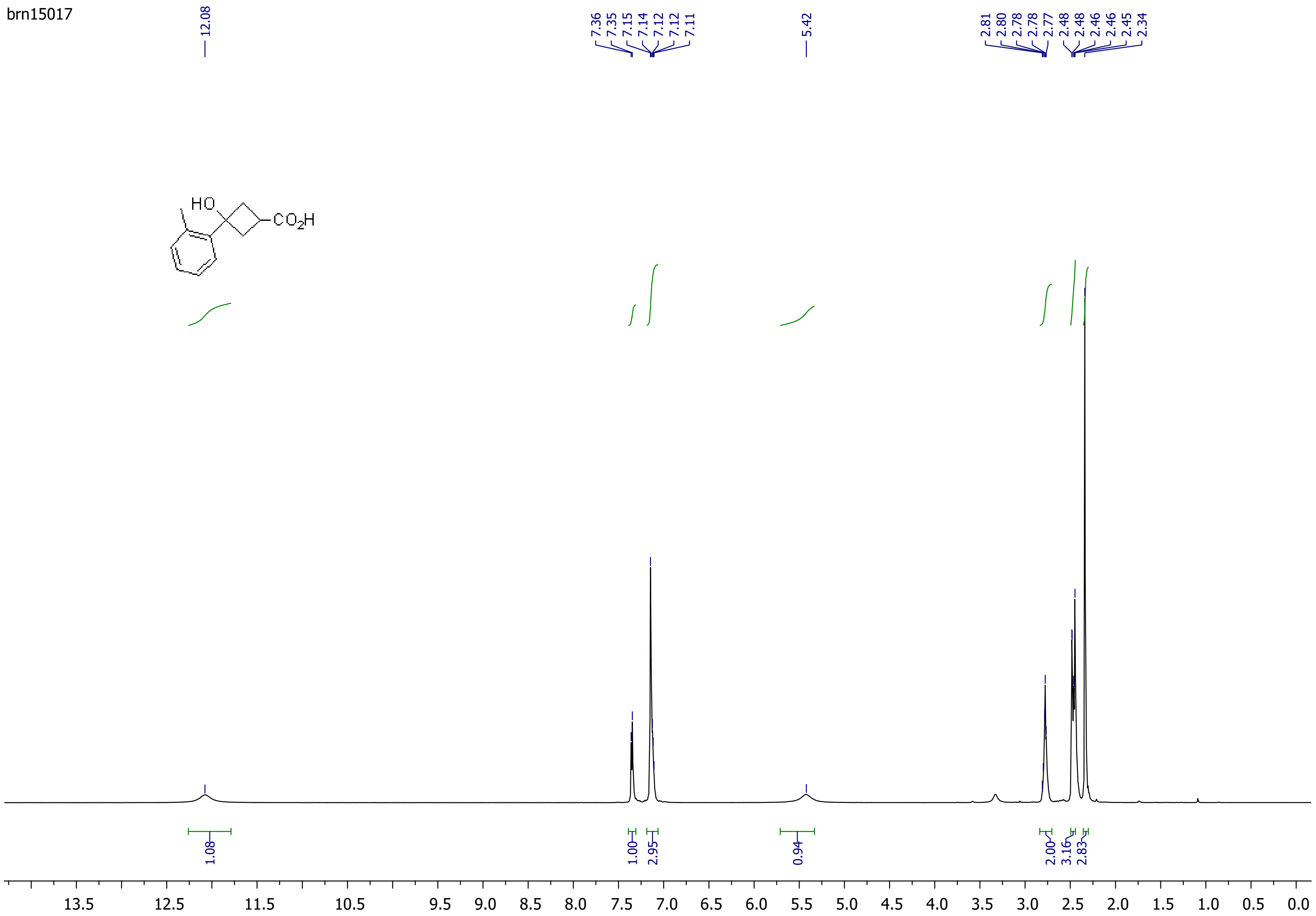

$^{13}C\{^{1}H\}$ NMR (126 MHz, DMSO-$d_6$)

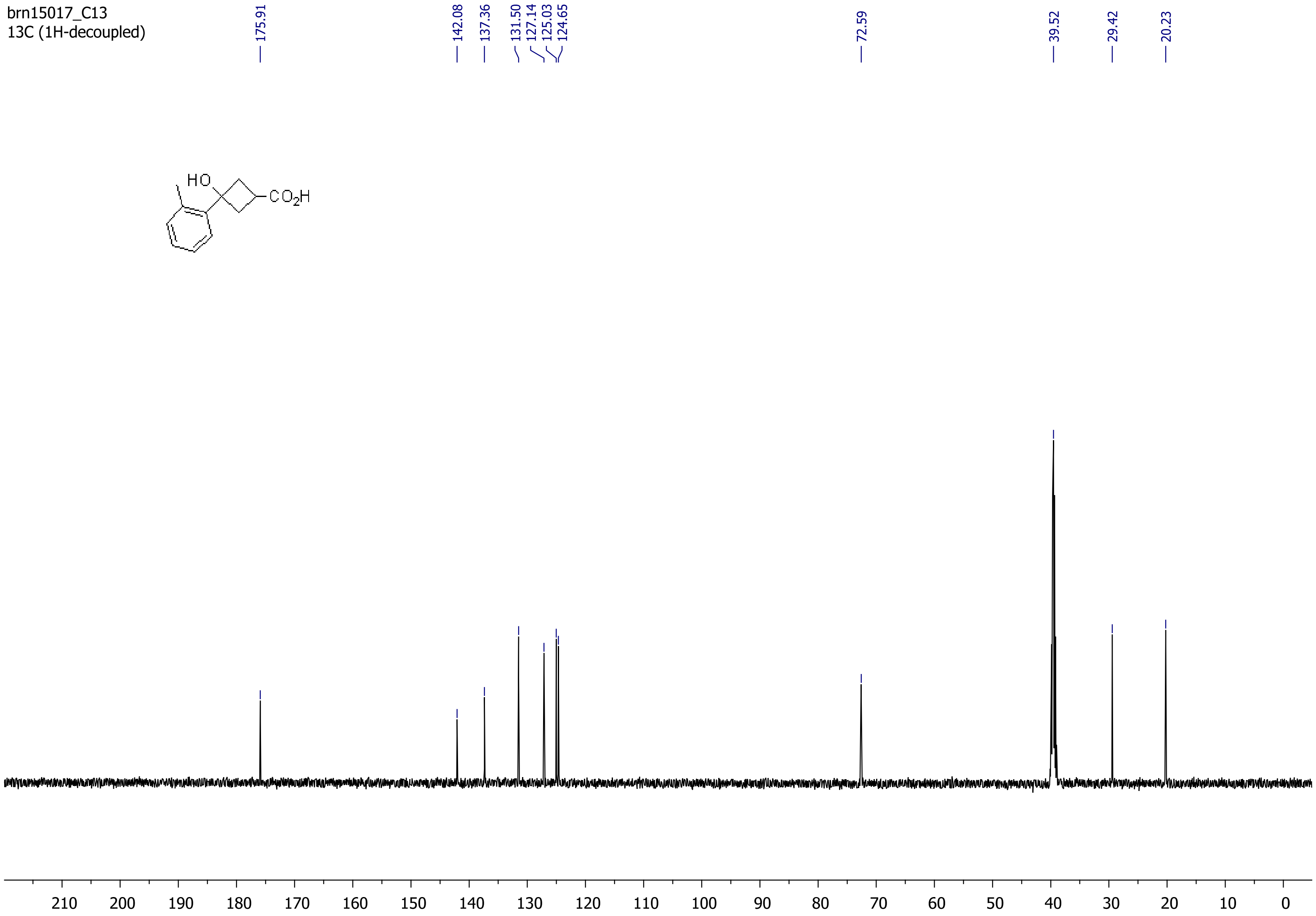

**3-Hydroxy-3-(*m*-tolyl)cyclobutane-1-carboxylic acid**

$^1H$ NMR (400 MHz, DMSO-d$_6$)

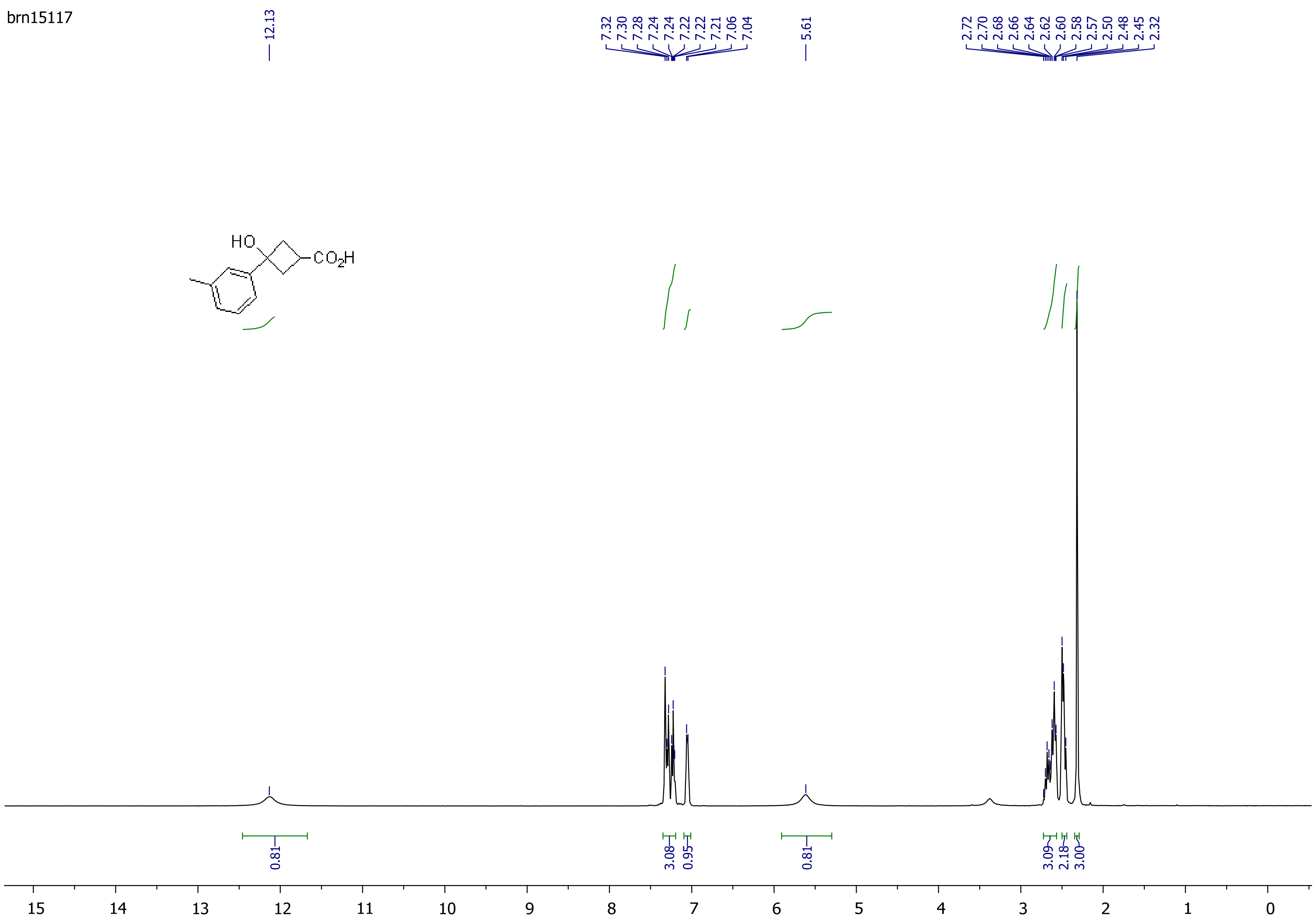

$^{13}C\{^{1}H\}$ NMR (126 MHz, DMSO-$d_6$)

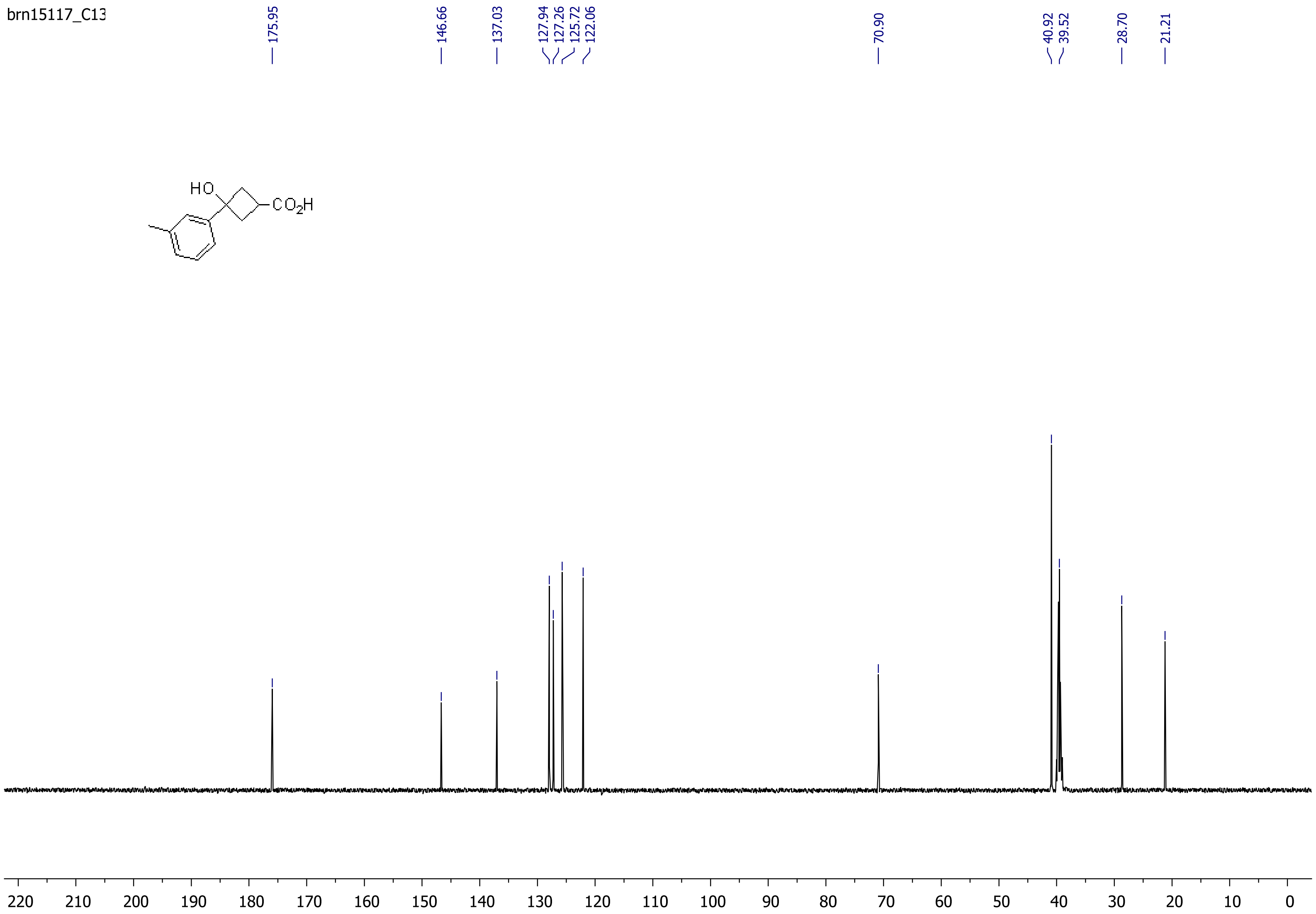

**3-Hydroxy-3-(*p*-tolyl)cyclobutane-1-carboxylic acid**

$^{1}$H NMR (500 MHz, DMSO-d$_6$)

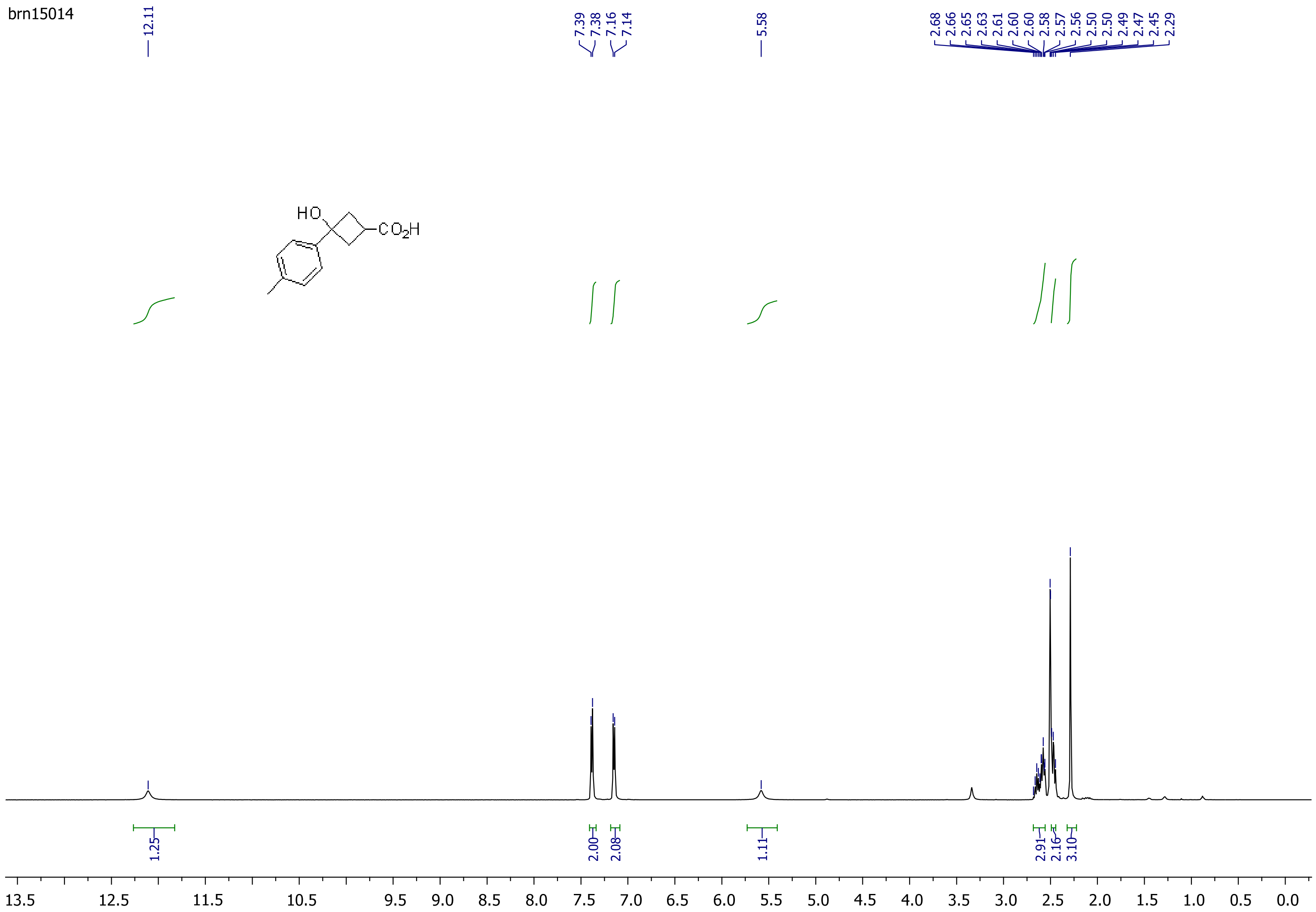

$^{13}C\{^1H\}$ NMR (126 MHz, DMSO-$d_6$)

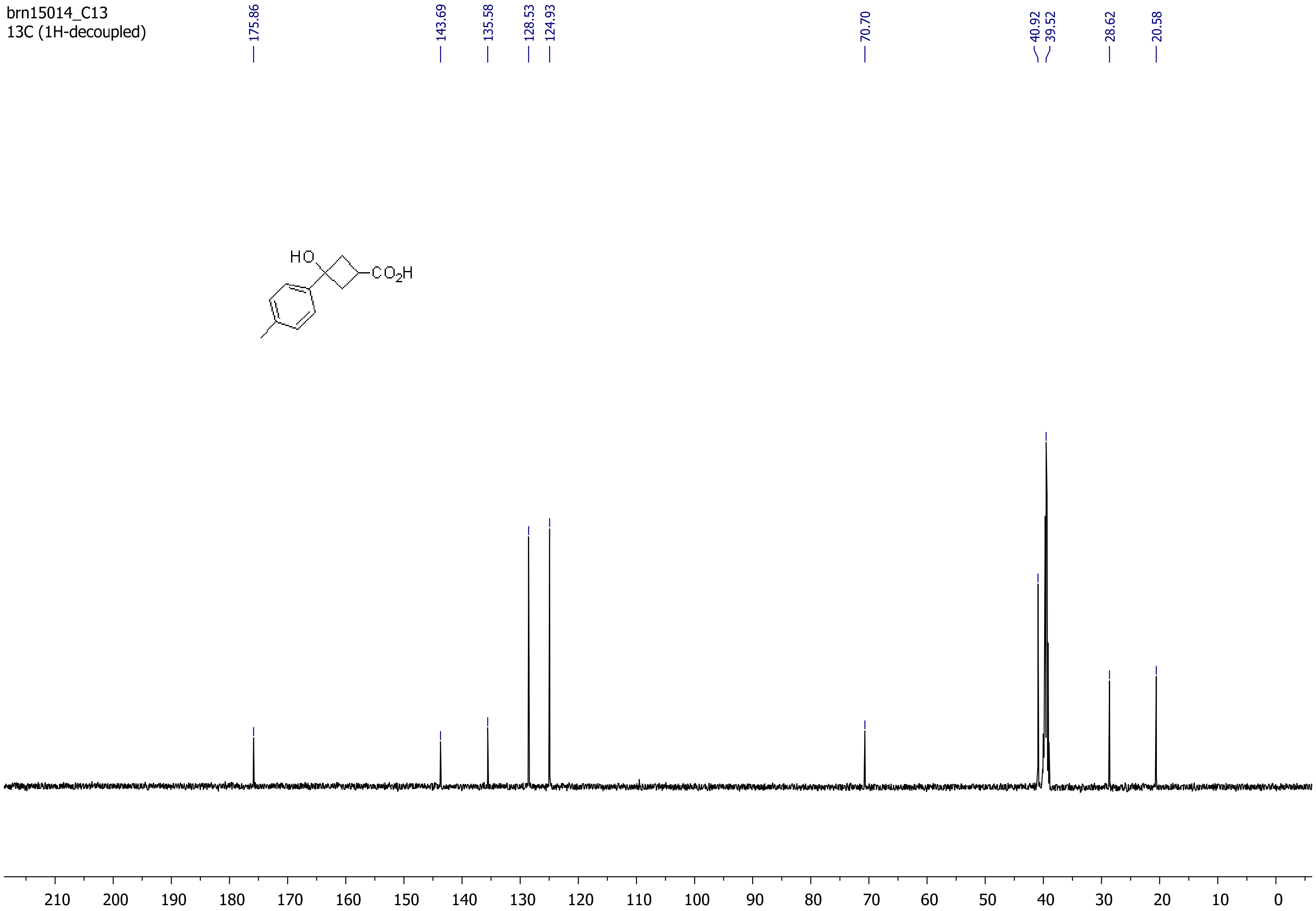

**3-(3-Fluorophenyl)-3-hydroxycyclobutane-1-carboxylic acid**

$^{1}$H NMR (500 MHz, DMSO-d$_6$)

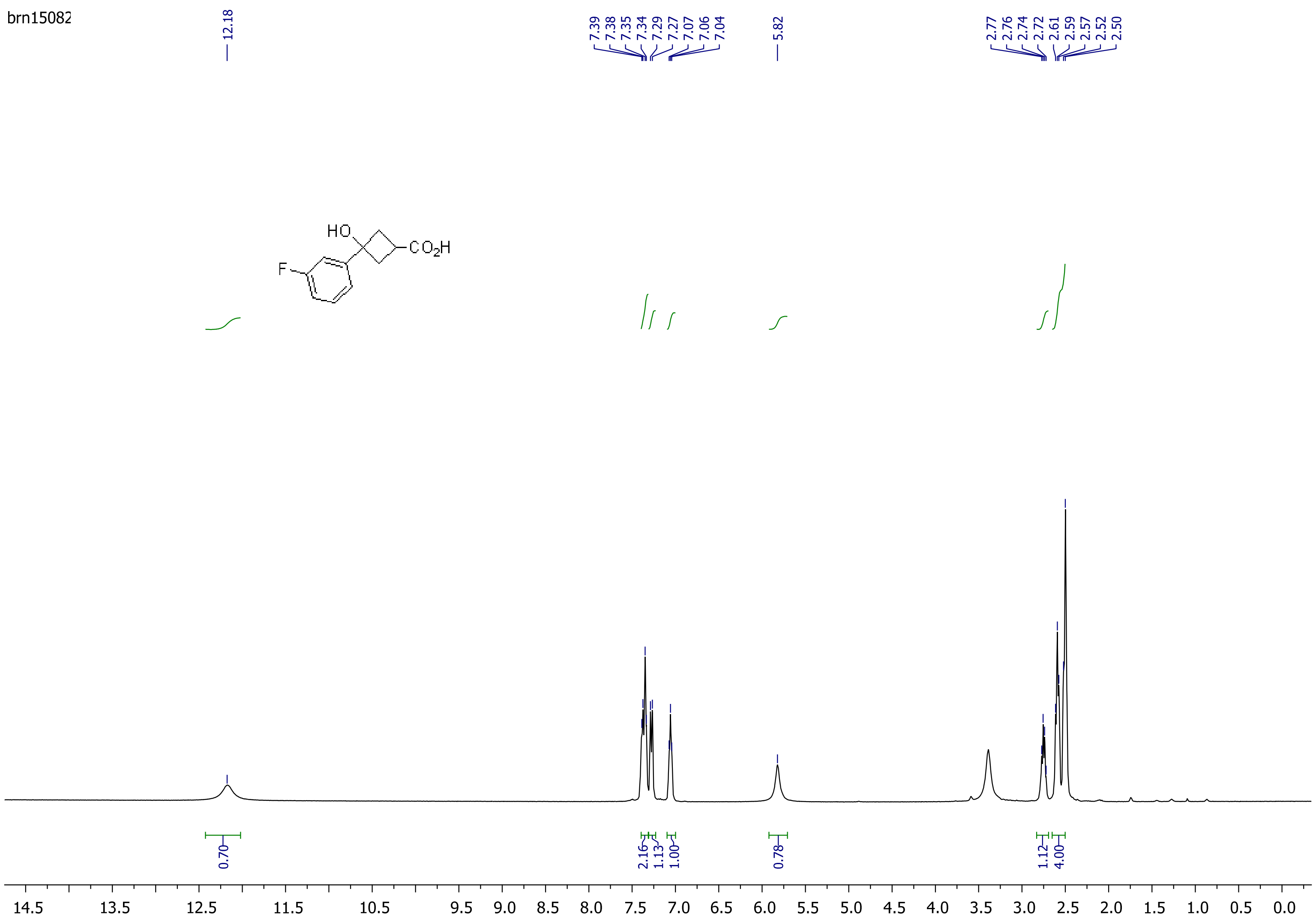

$^{13}C\{^1H\}$ NMR (126 MHz, DMSO-$d_6$)

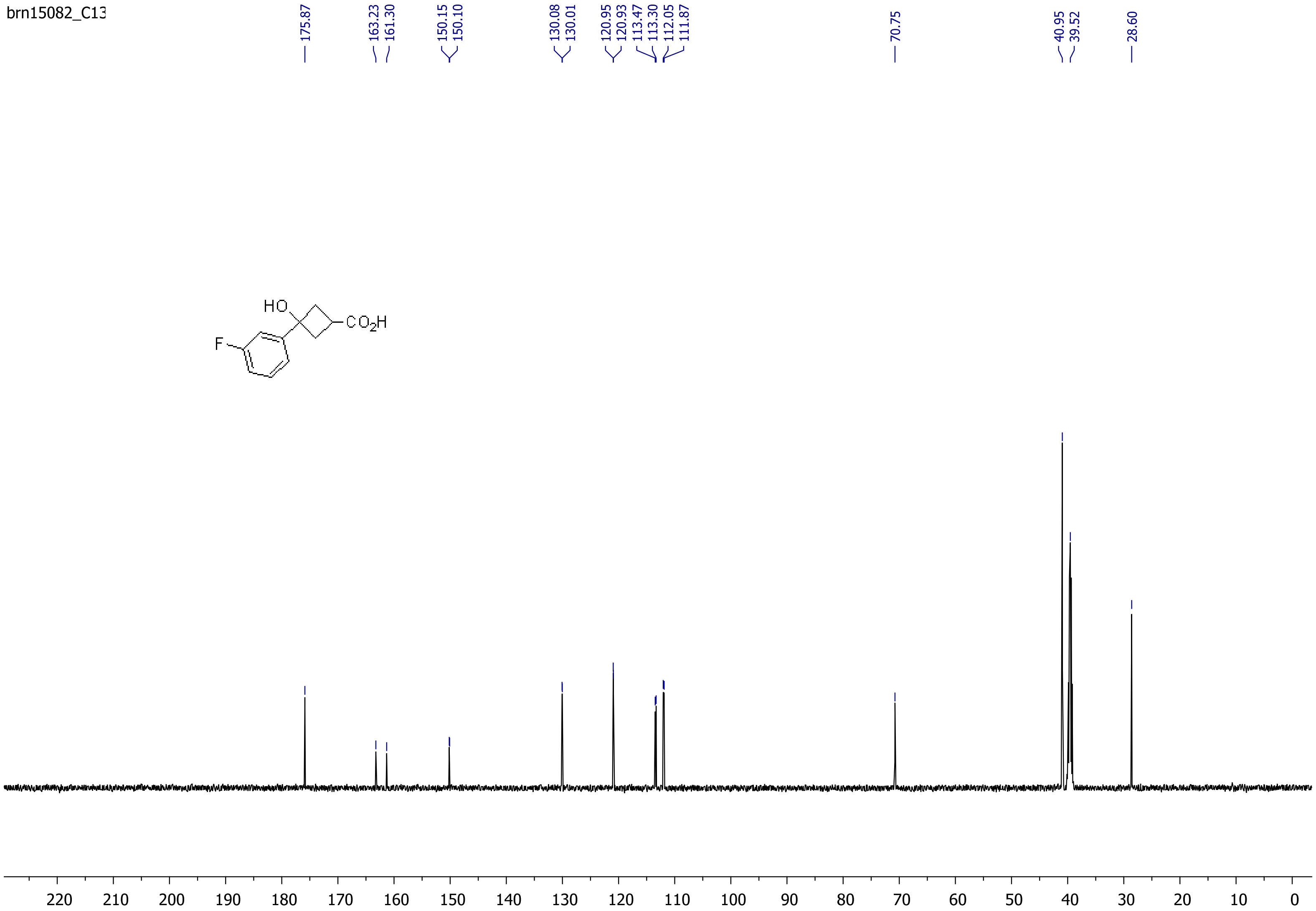

$^{19}F\{^{1}H\}$ NMR (376 MHz, DMSO-$d_6$)

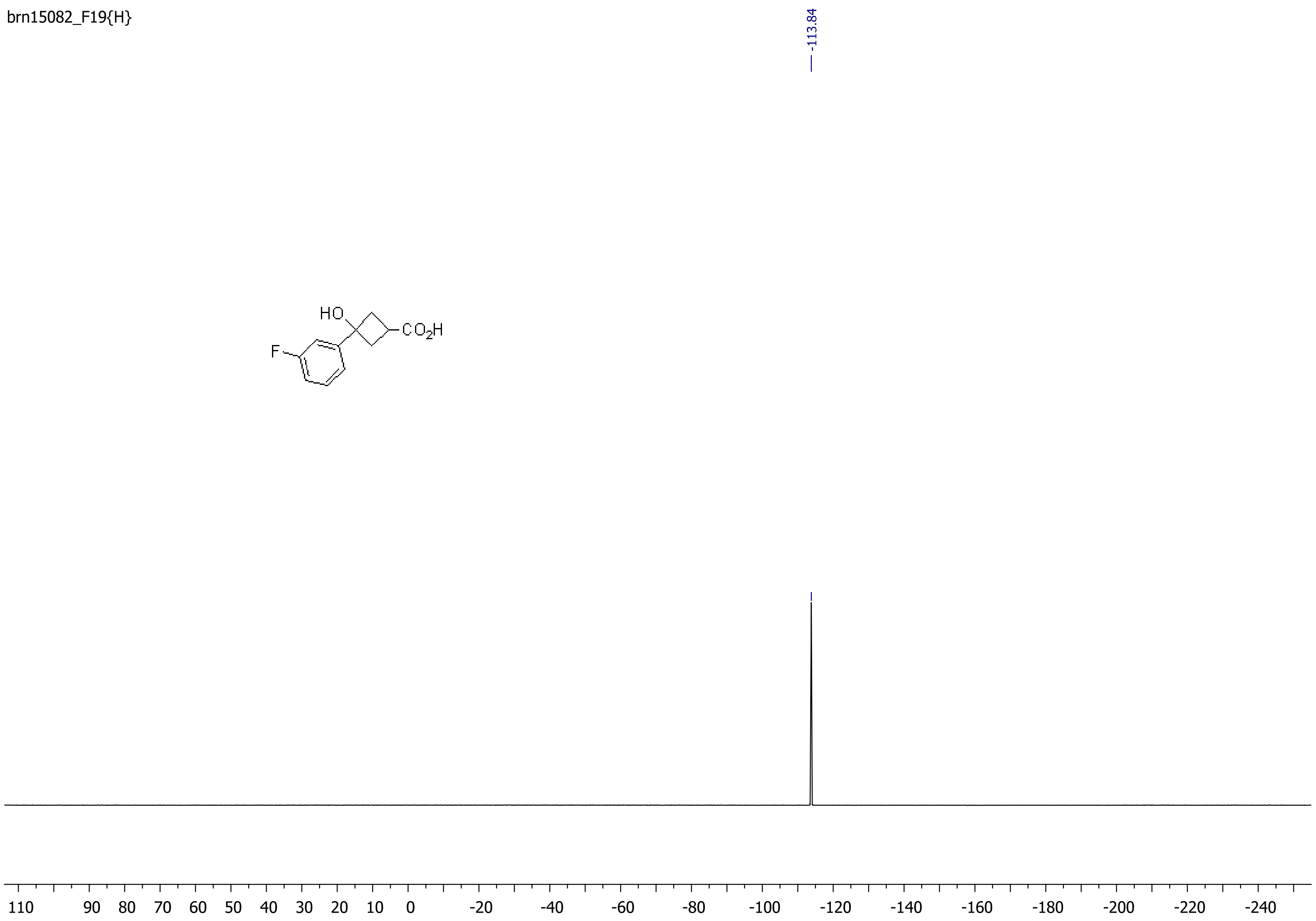

**3-(4-Fluorophenyl)-3-hydroxycyclobutane-1-carboxylic acid**

$^1H$ NMR (400 MHz, DMSO-$d_6$)

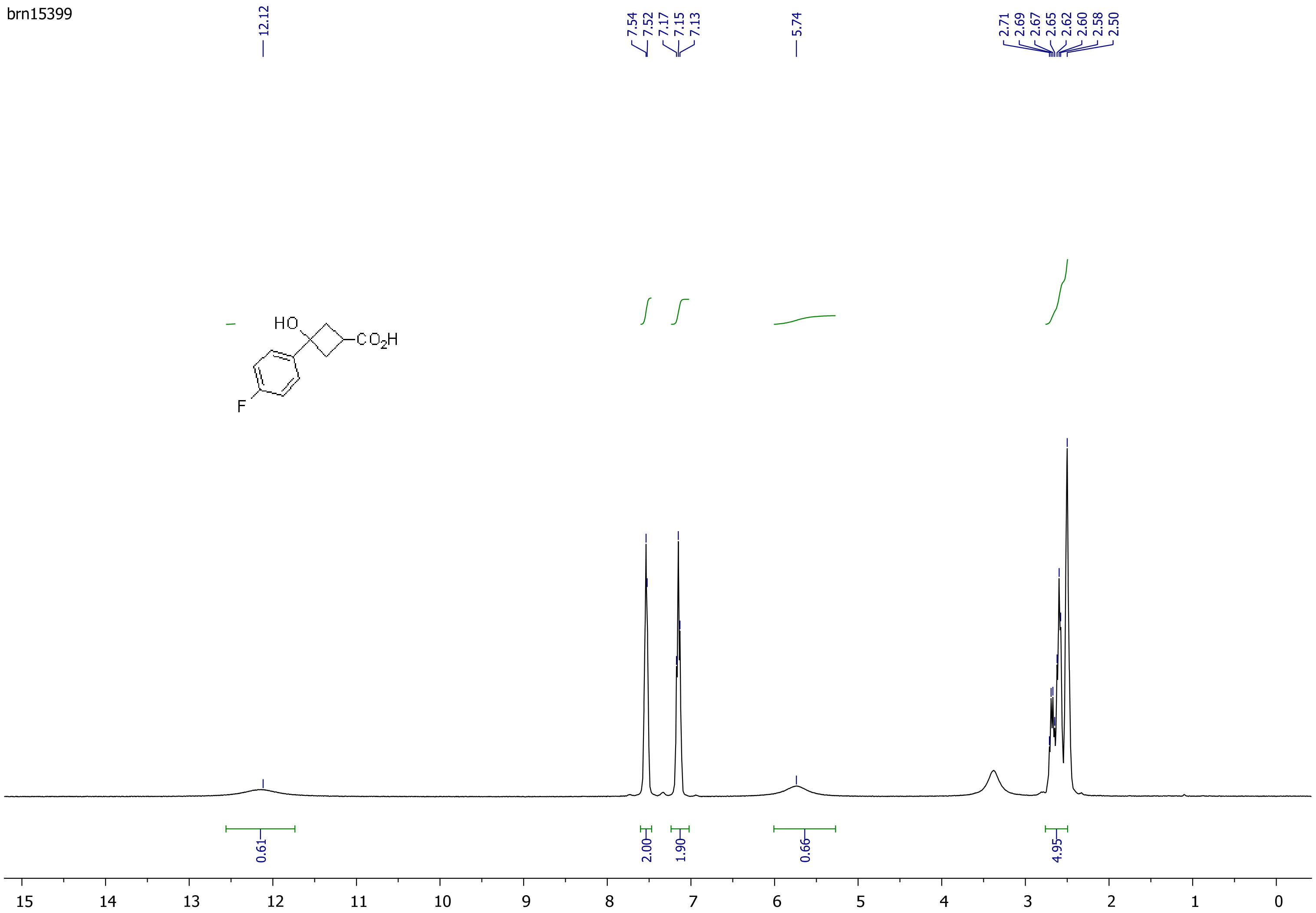

$^{13}C\{^{1}H\}$ NMR (101 MHz, DMSO-$d_6$)

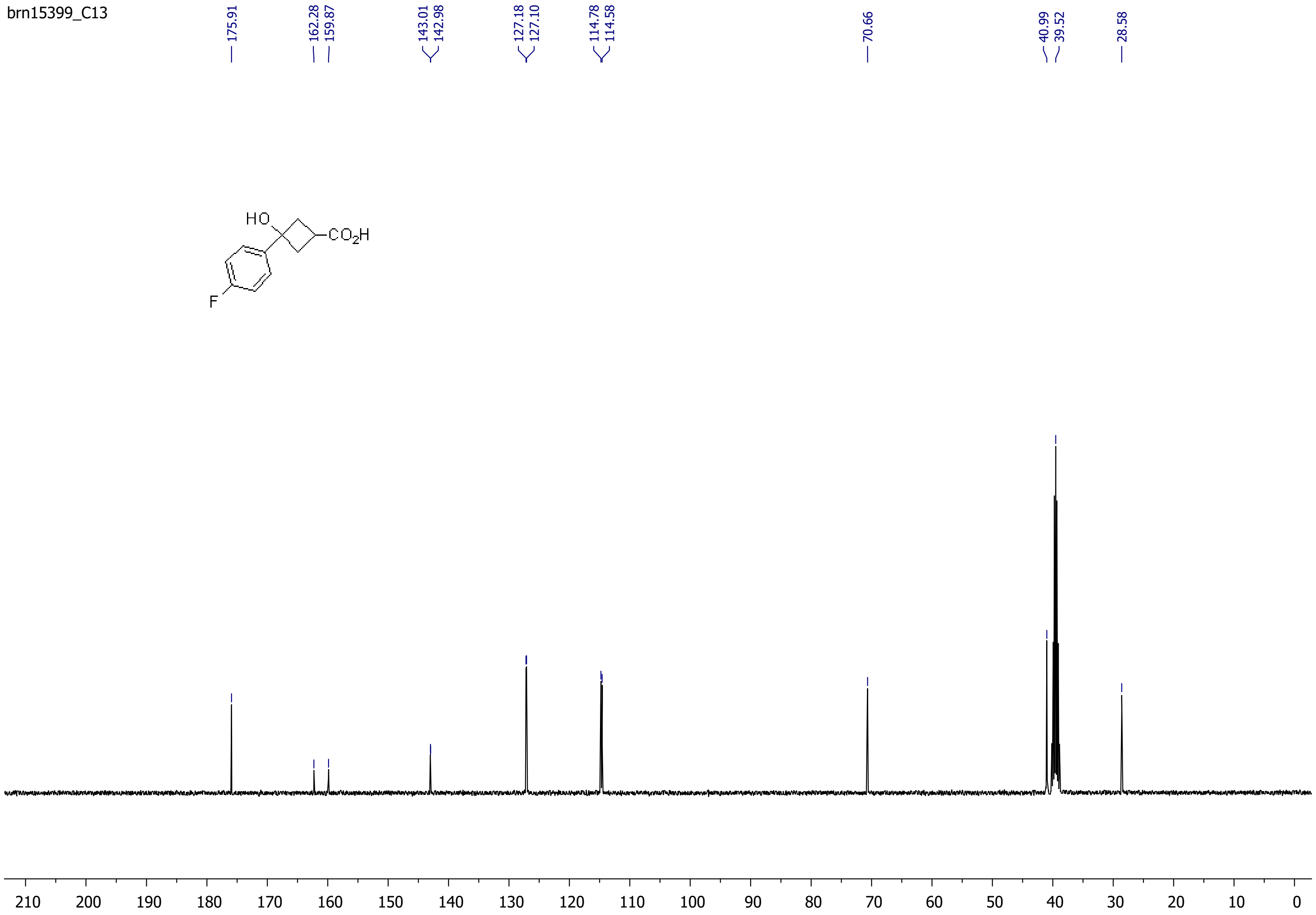

$^{19}F\{^{1}H\}$ NMR (376 MHz, DMSO-$d_6$)

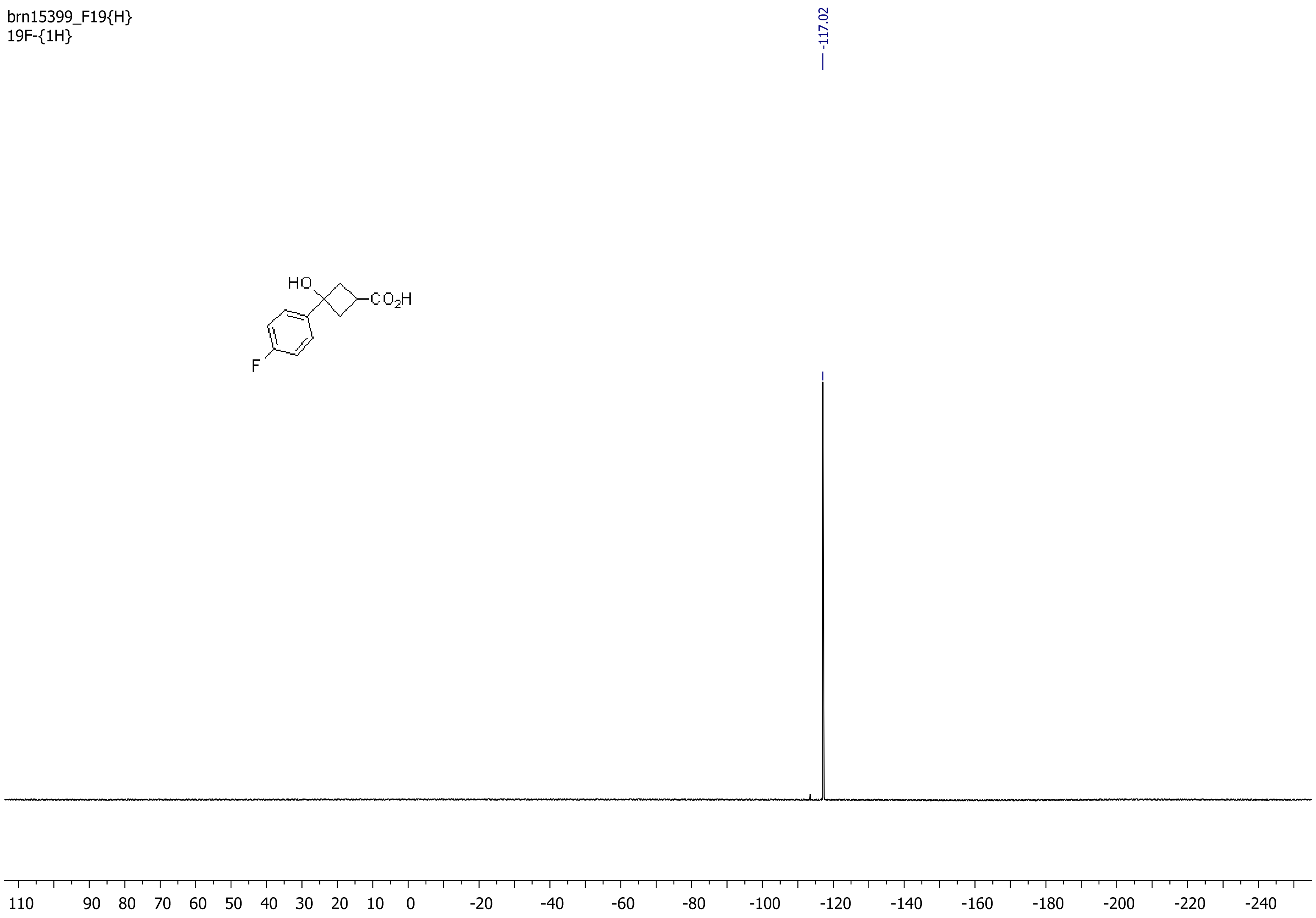

**3-Hydroxy-3-(3-(trifluoromethyl)phenyl)cyclobutane-1-carboxylic acid**

$^1$H NMR (500 MHz, DMSO-$d_6$)

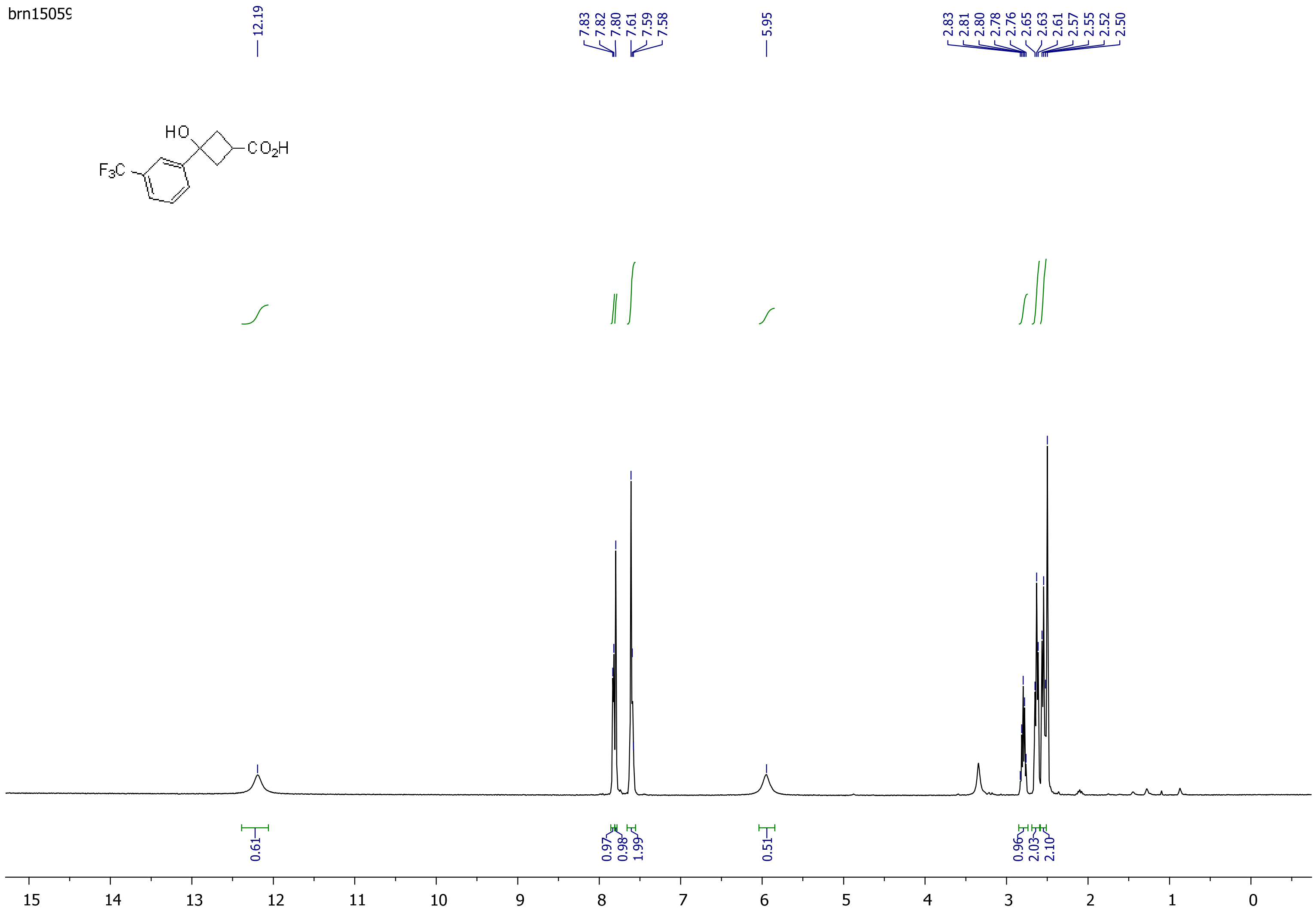

$^{13}C\{^1H\}$ NMR (126 MHz, DMSO-d$_6$)

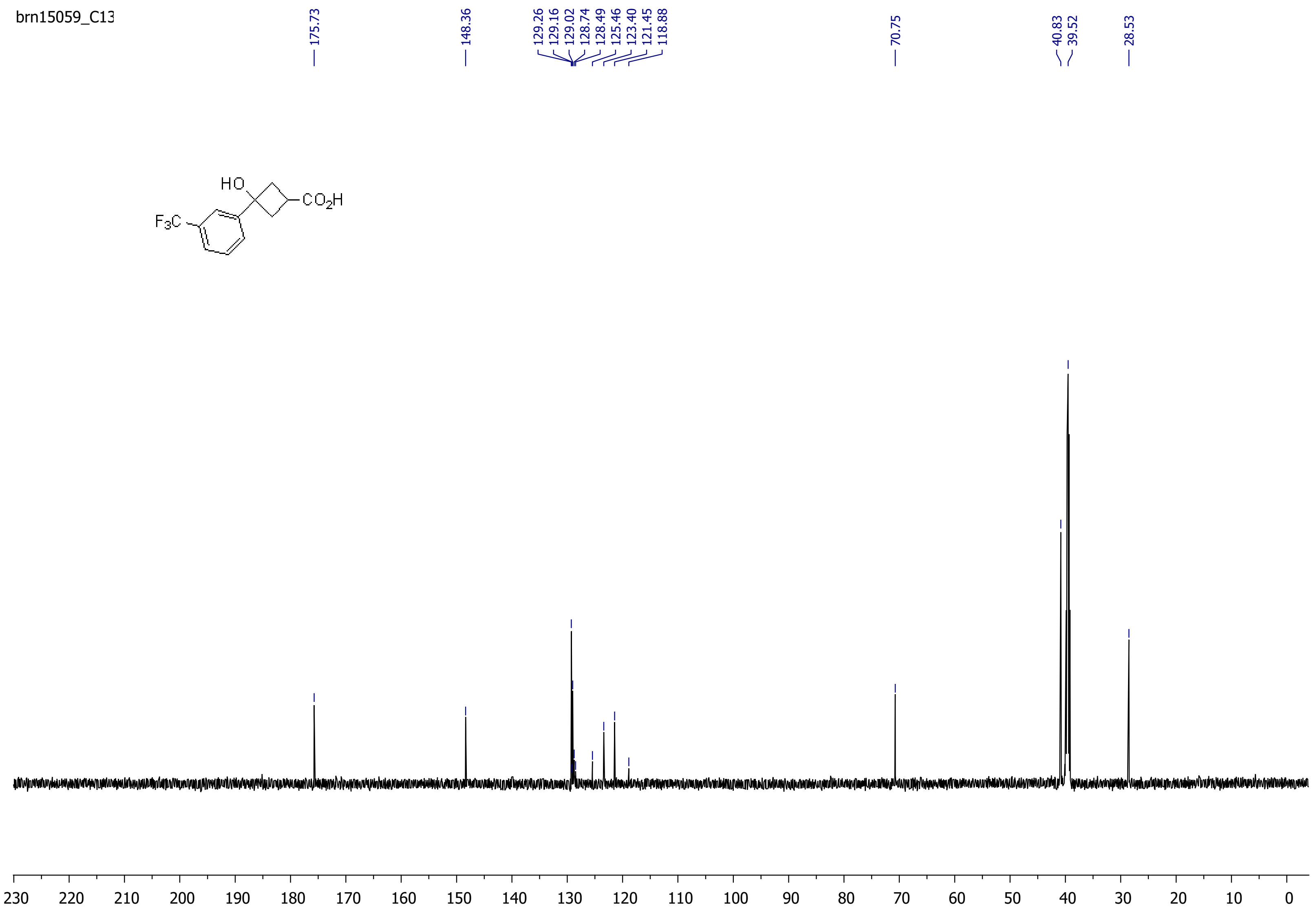

$^{19}F\{^{1}H\}$ NMR (376 MHz, DMSO-$d_6$)

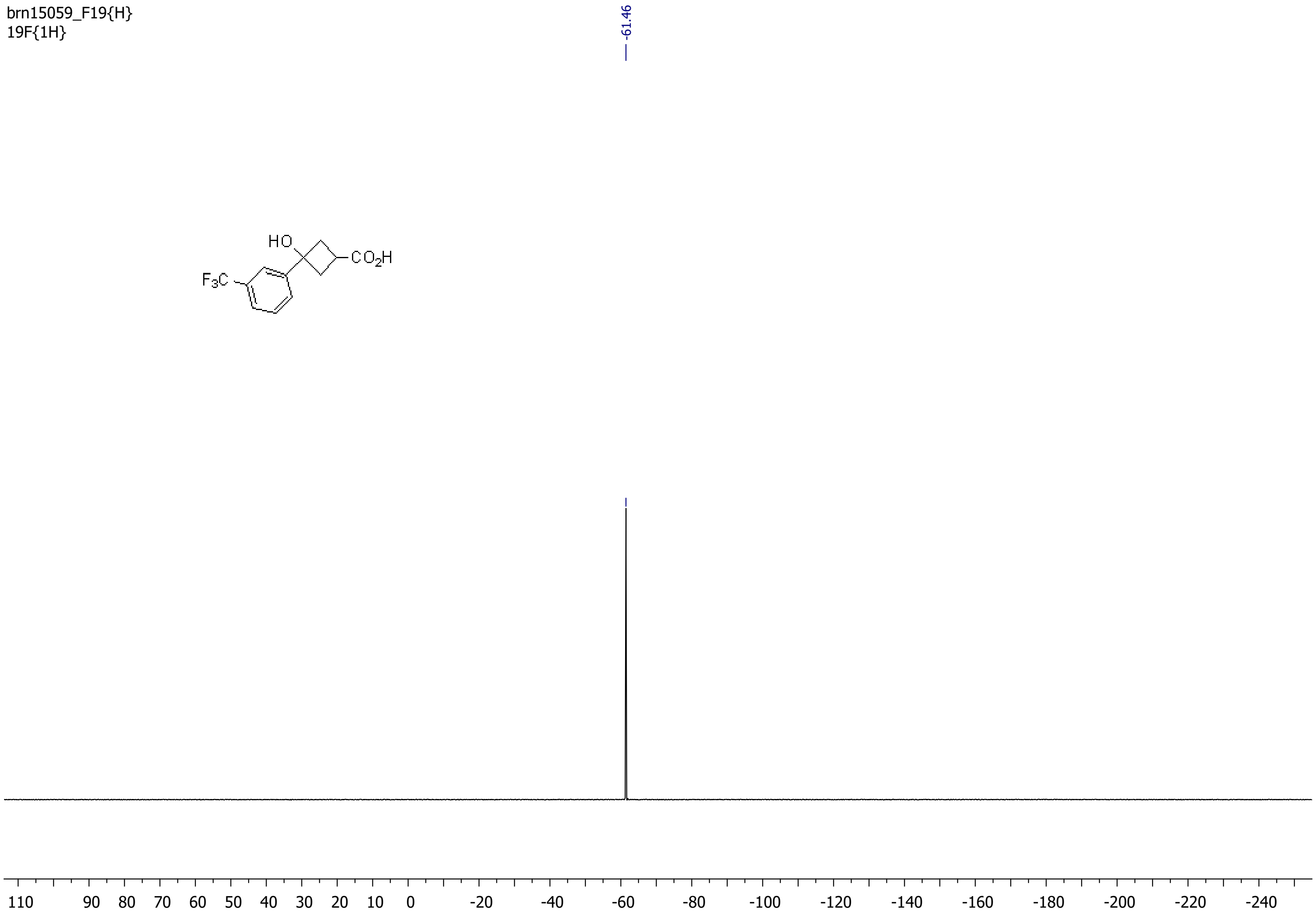

**3-Hydroxy-3-(4-(trifluoromethyl)phenyl)cyclobutane-1-carboxylic acid**

$^{1}$H NMR (500 MHz, DMSO-$d_6$)

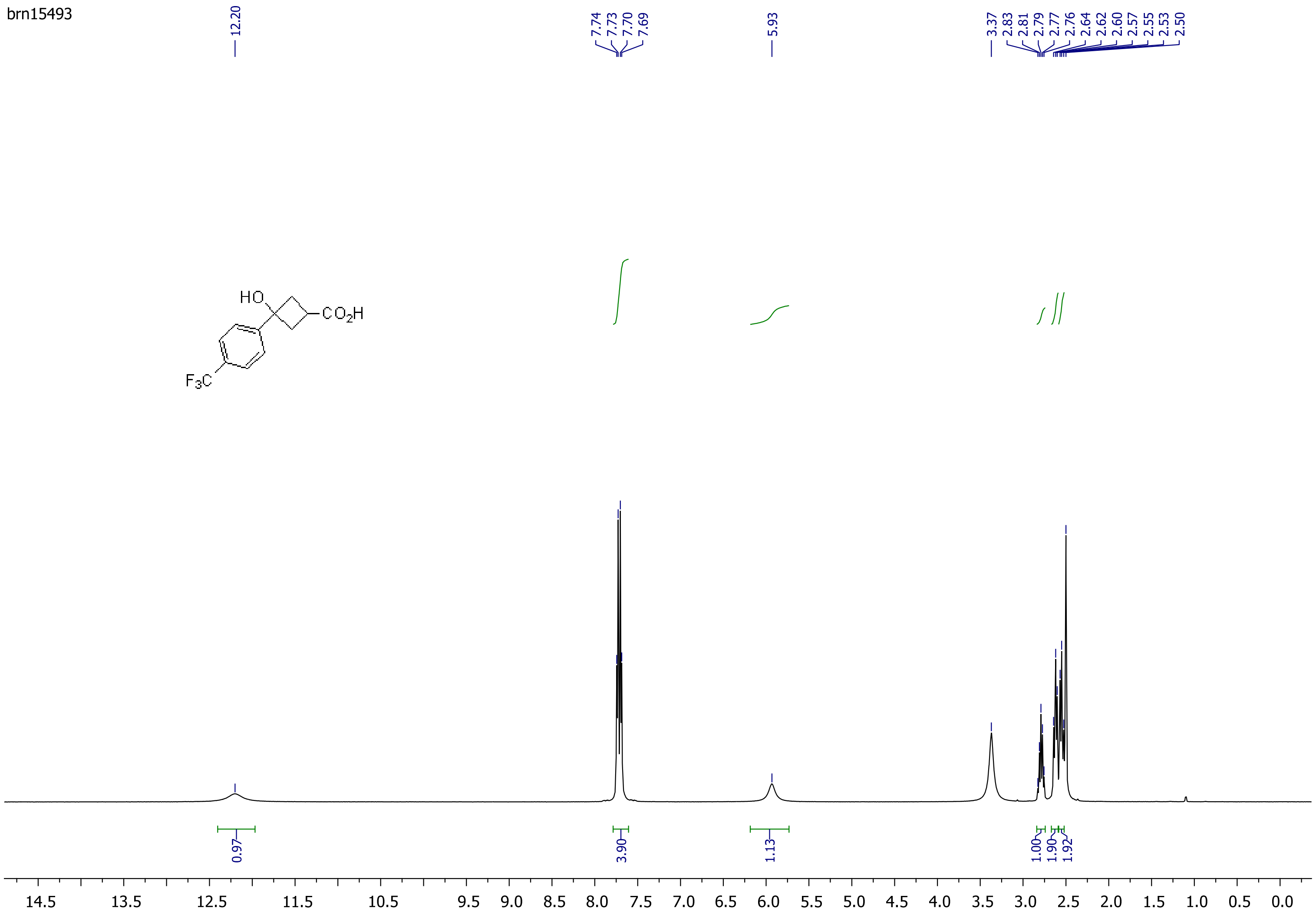

$^{13}C\{^{1}H\}$ NMR (126 MHz, DMSO-$d_6$)

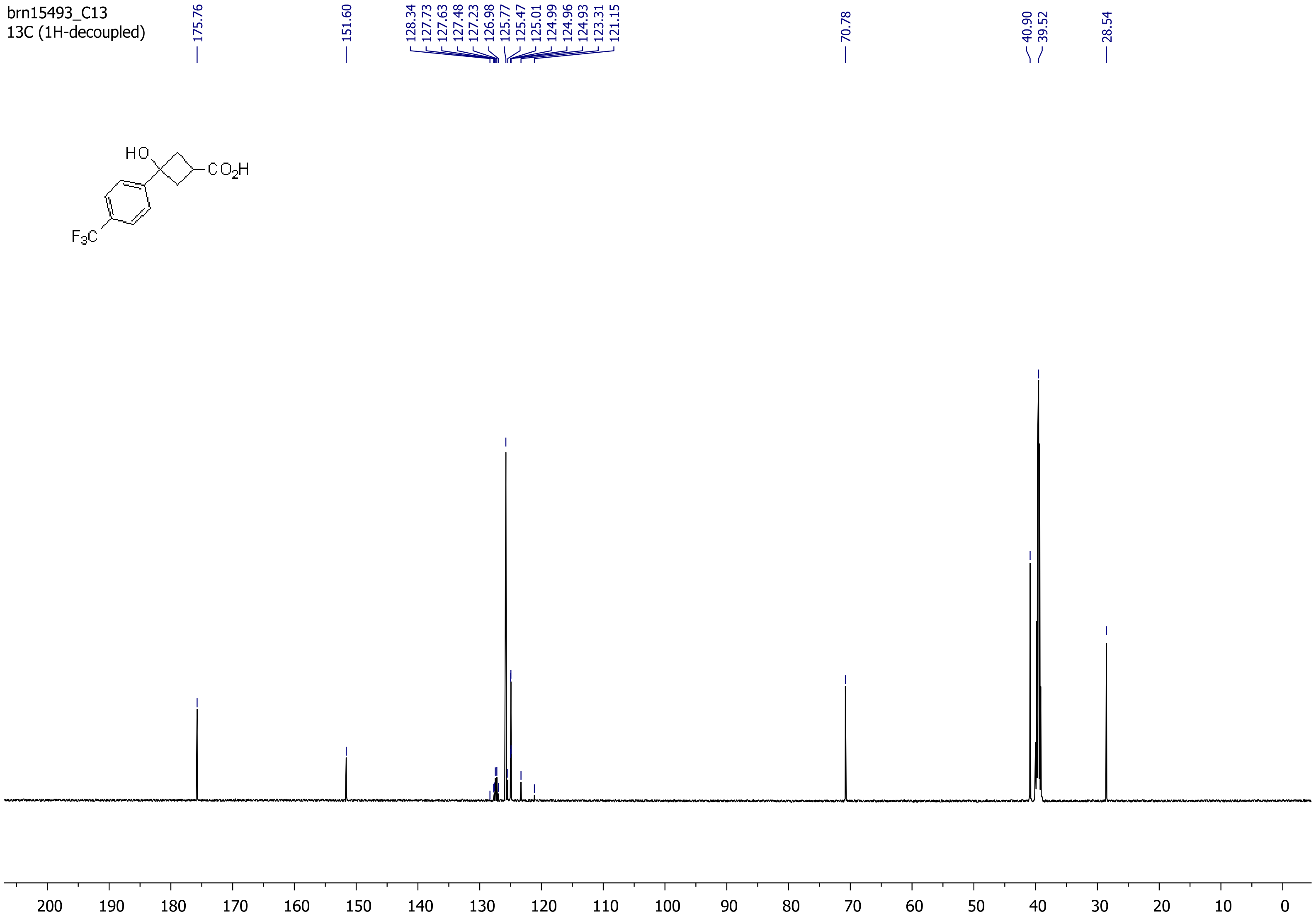

$^{19}F\{^{1}H\}$ NMR (376 MHz, DMSO-$d_6$)

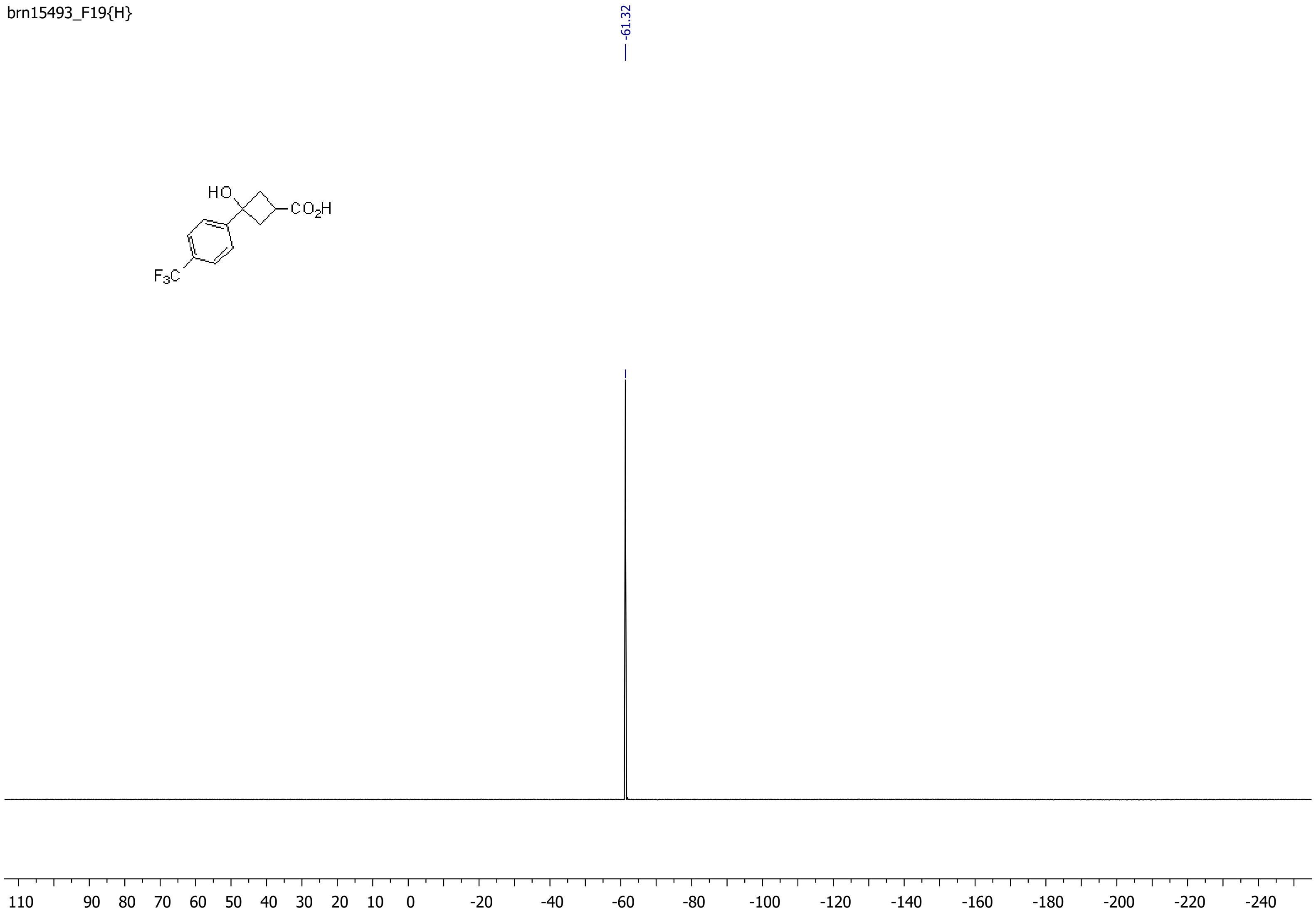

## 3-Chloro-3-phenylcyclobutane-1-carboxylic acid

$^1$H NMR (500 MHz, DMSO-$d_6$)

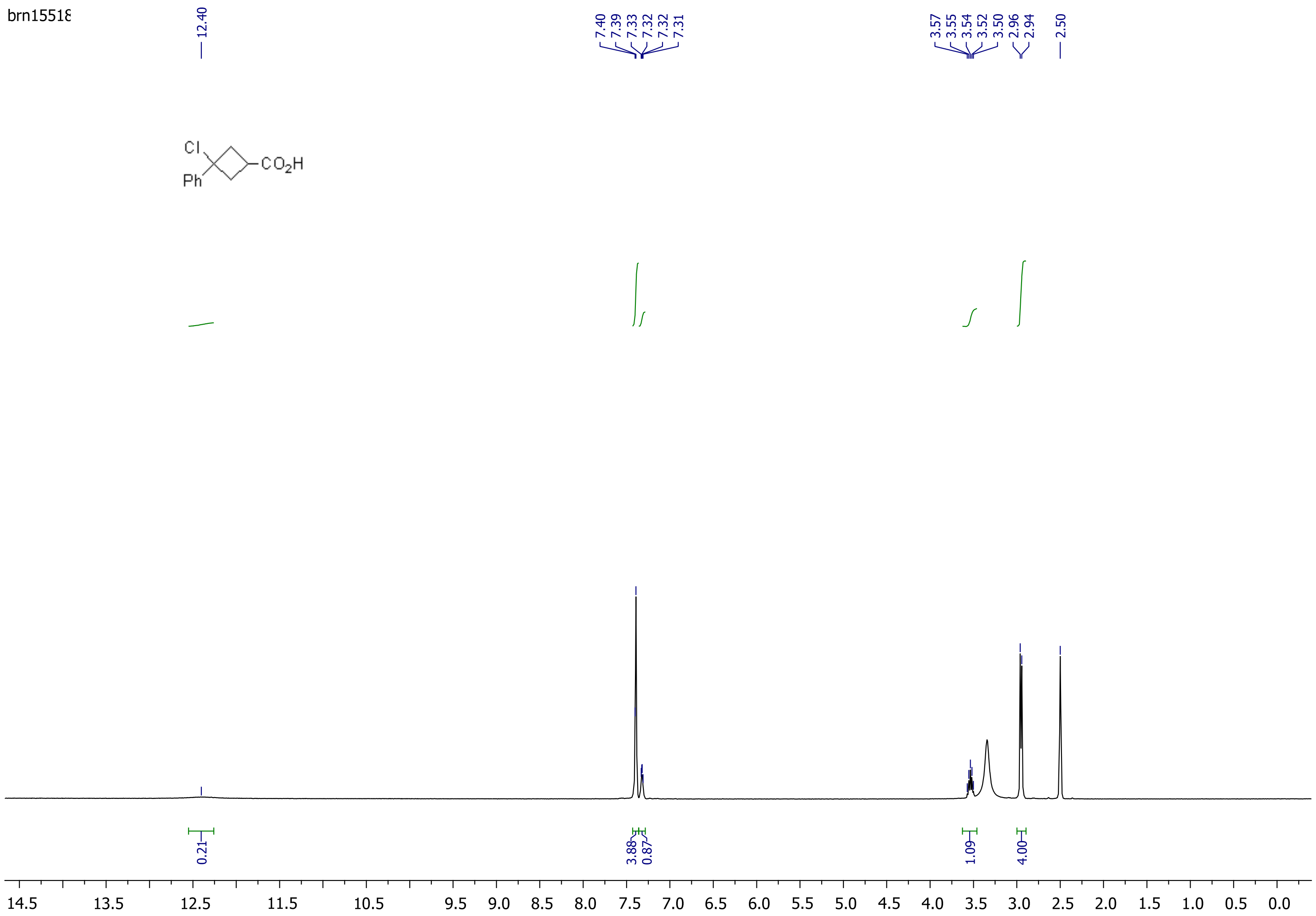

$^{13}C\ \{^1H\}$ NMR (126 MHz, DMSO-$d_6$)

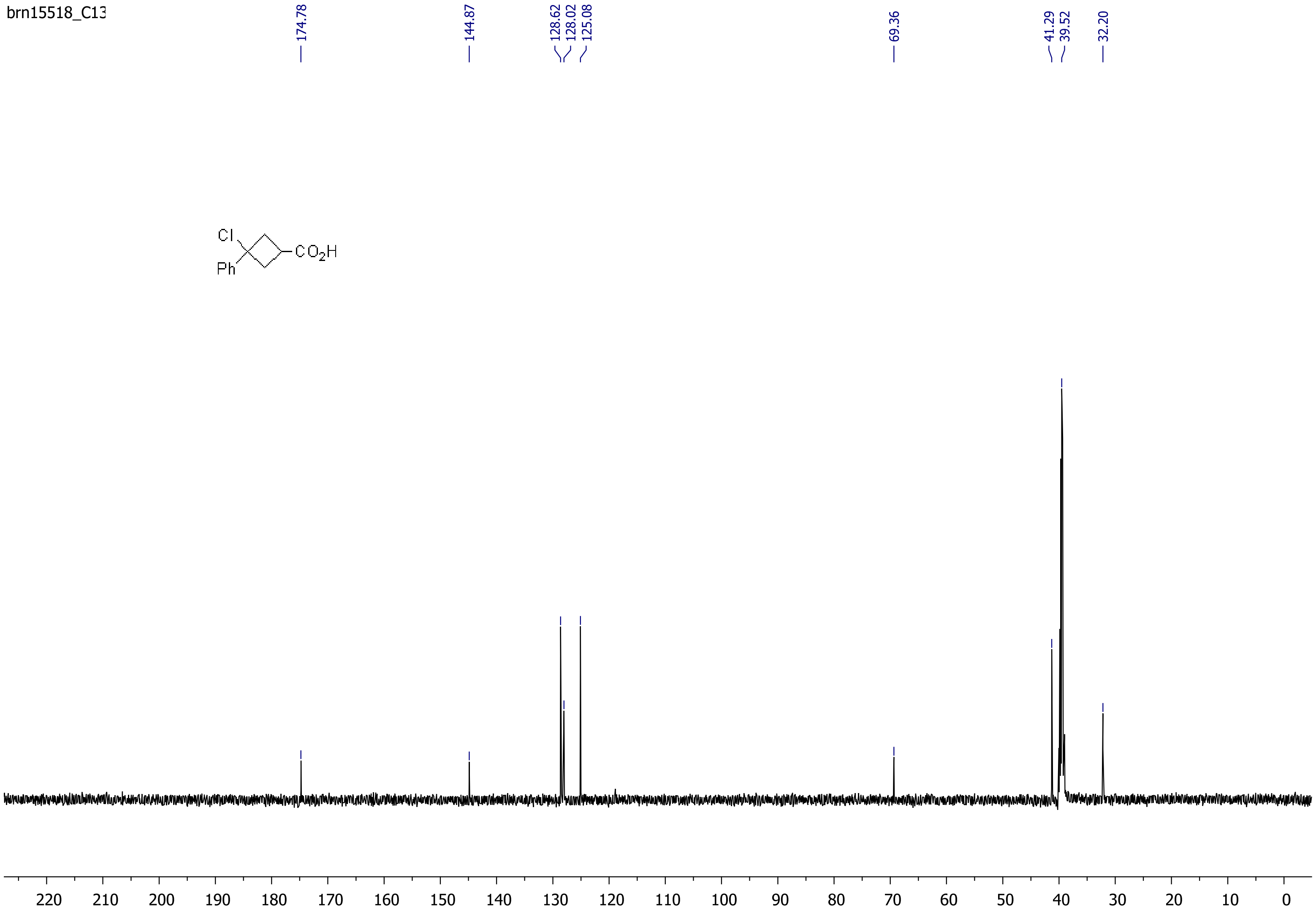

**3-Chloro-3-(*o*-tolyl)cyclobutane-1-carboxylic acid**

$^{1}H$ NMR (500 MHz, DMSO-$d_6$)

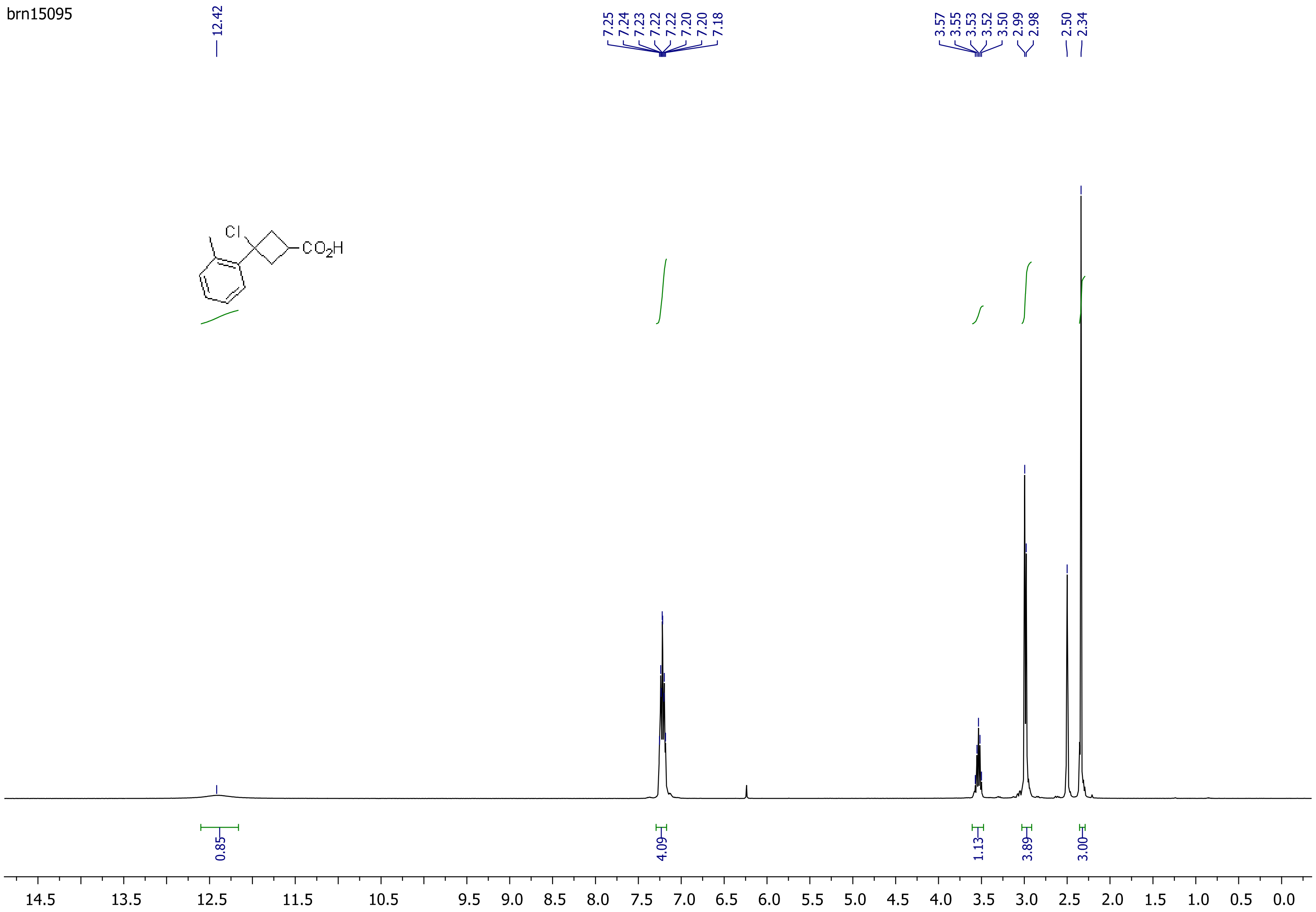

$^{13}C\{^1H\}$ NMR (151 MHz, DMSO-d$_6$) (mixture of cis-/trans-isomers)

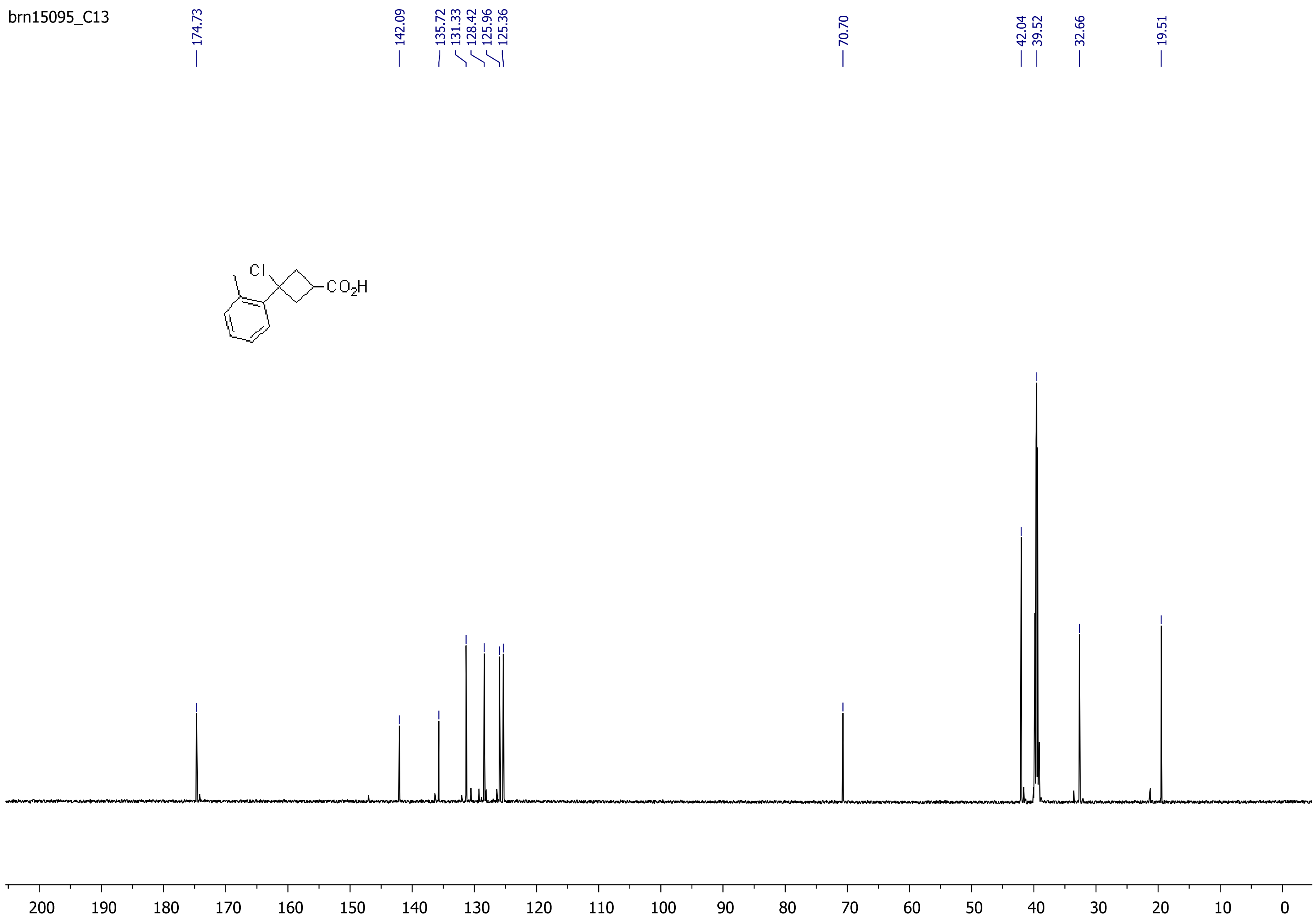

**(3-Chloro-3-(*o*-tolyl)cyclobutyl)(l1-oxidaneyl)methanone**

$^1$H NMR (400 MHz, DMSO-d$_6$)

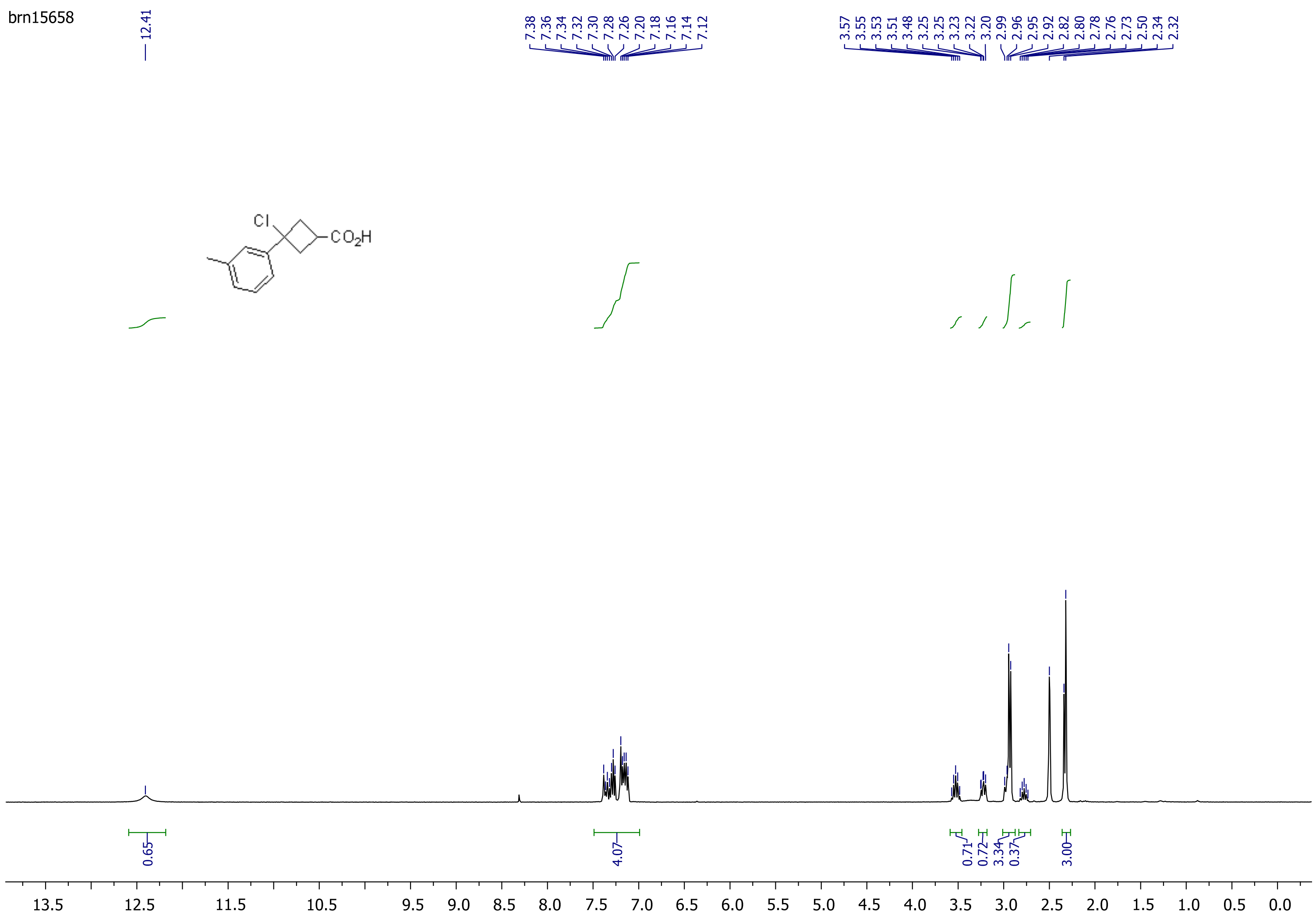

$^{13}C\{^1H\}$ NMR (126 MHz, DMSO-$d_6$)

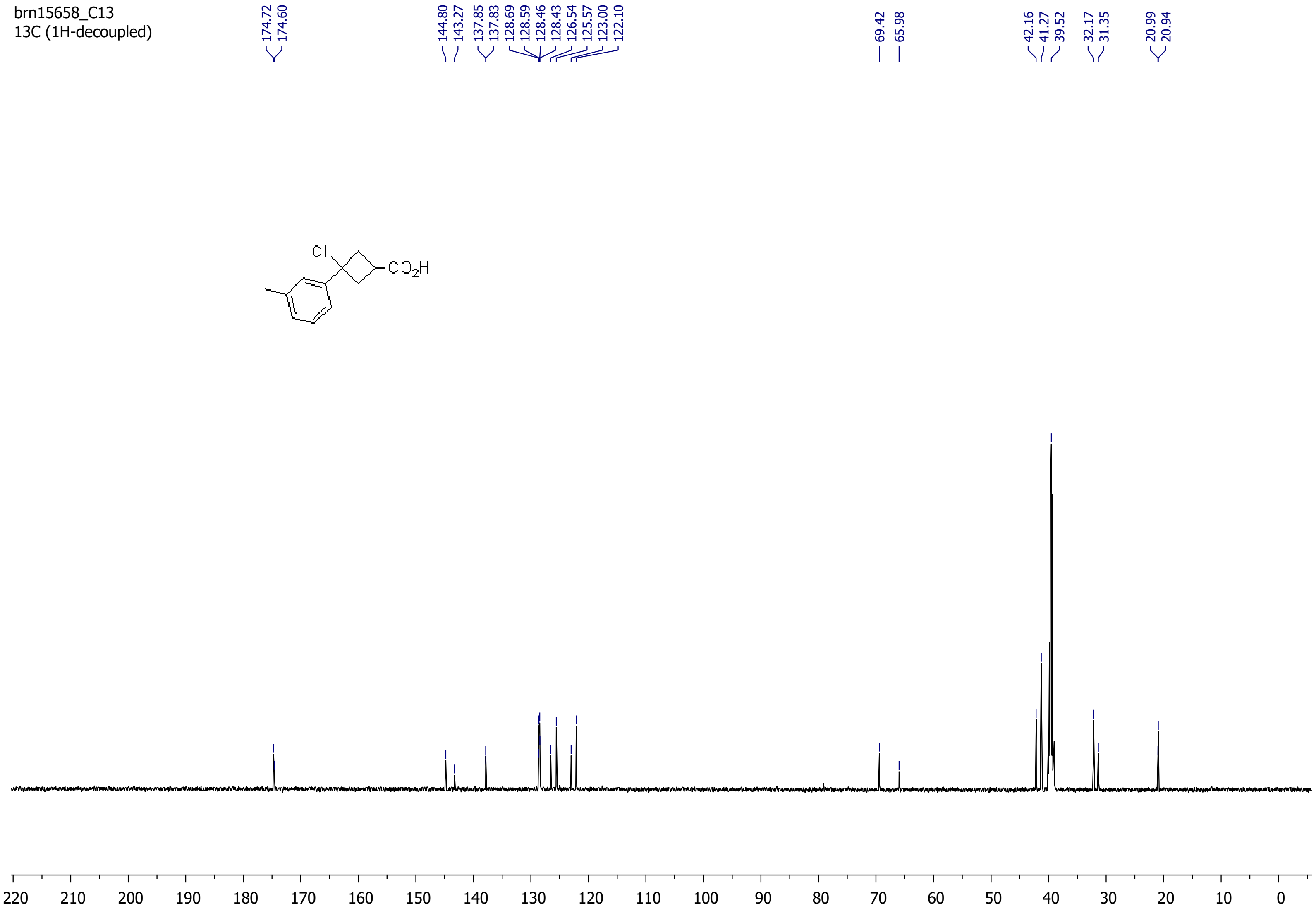

**3-Chloro-3-(*p*-tolyl)cyclobutane-1-carboxylic acid**

$^1$H NMR (500 MHz, DMSO-d$_6$)

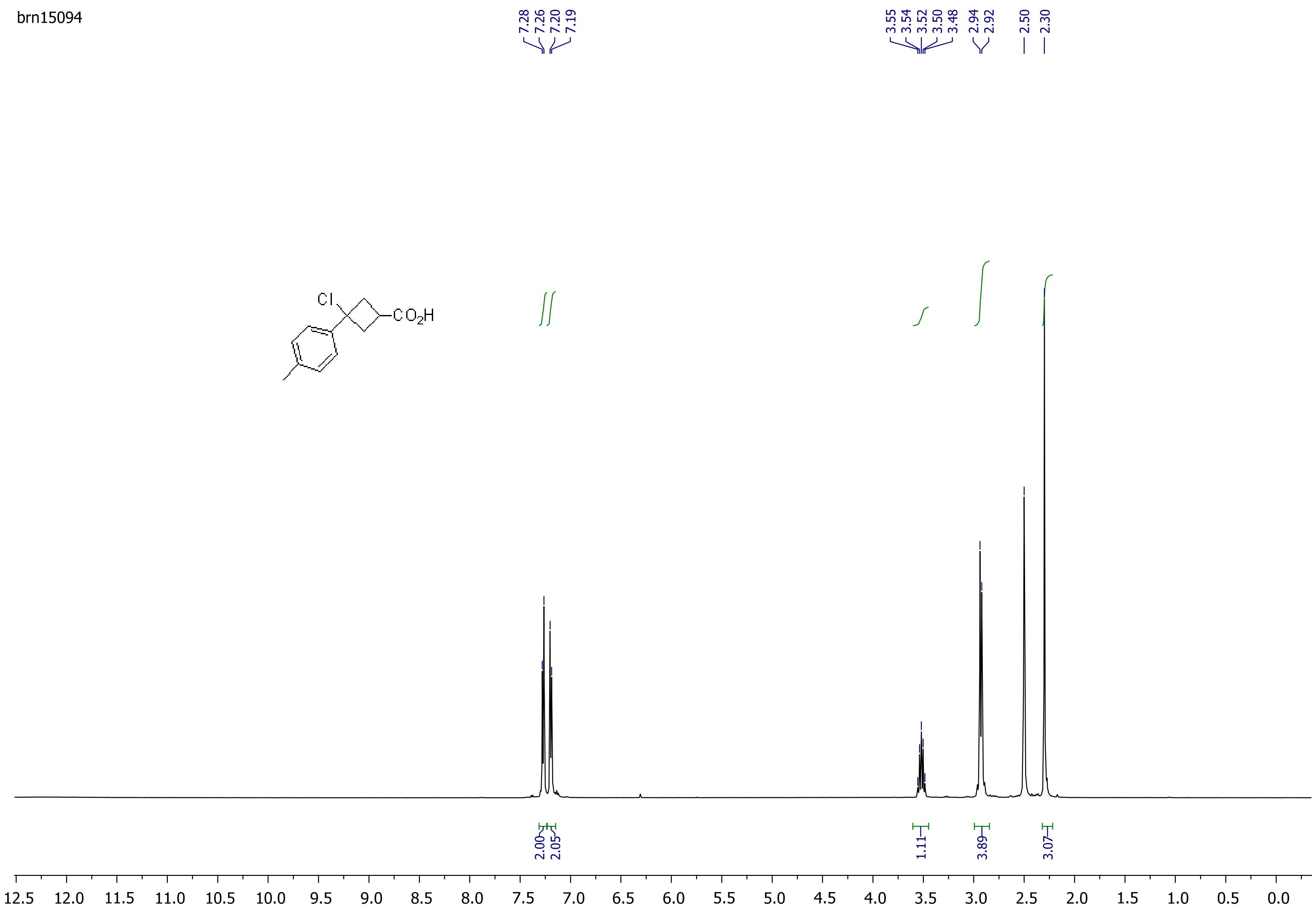

$^{13}C\{^{1}H\}$ NMR (126 MHz, DMSO-d$_6$)

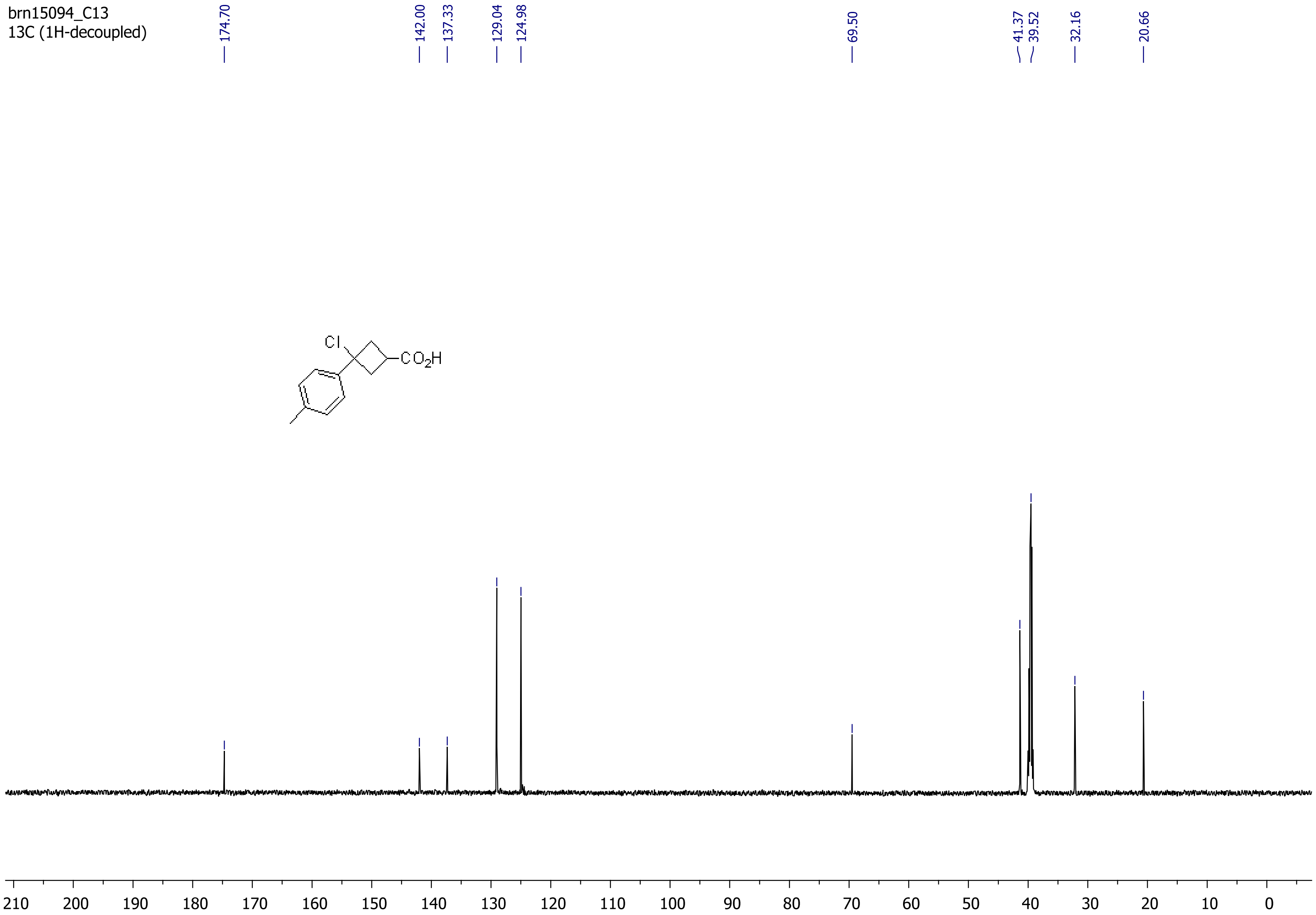

**3-Chloro-3-(3-fluorophenyl)cyclobutane-1-carboxylic acid**

$^{1}$H NMR (400 MHz, DMSO-d$_6$)

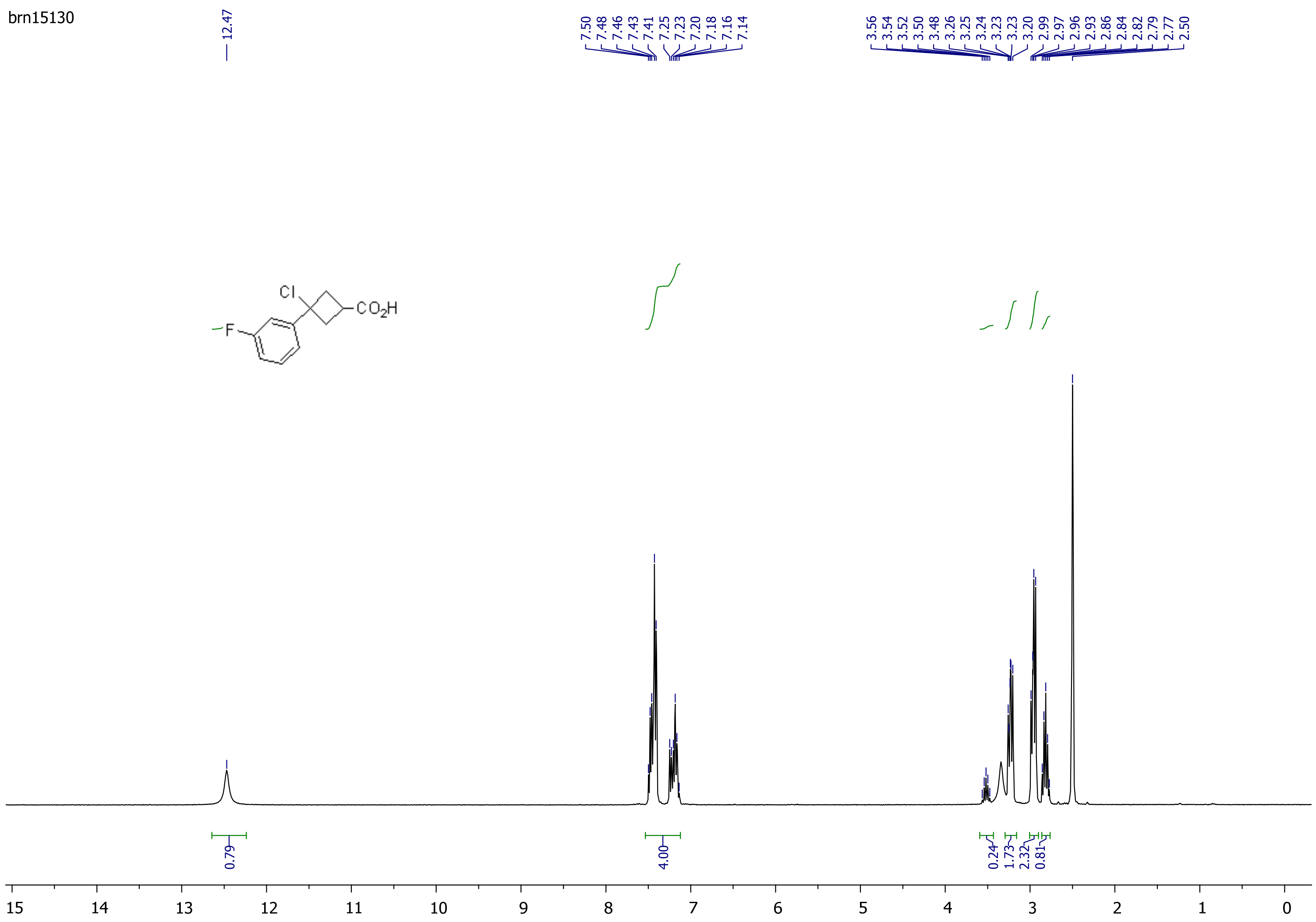

$^{13}C\{^1H\}$ NMR (151 MHz, DMSO-$d_6$)

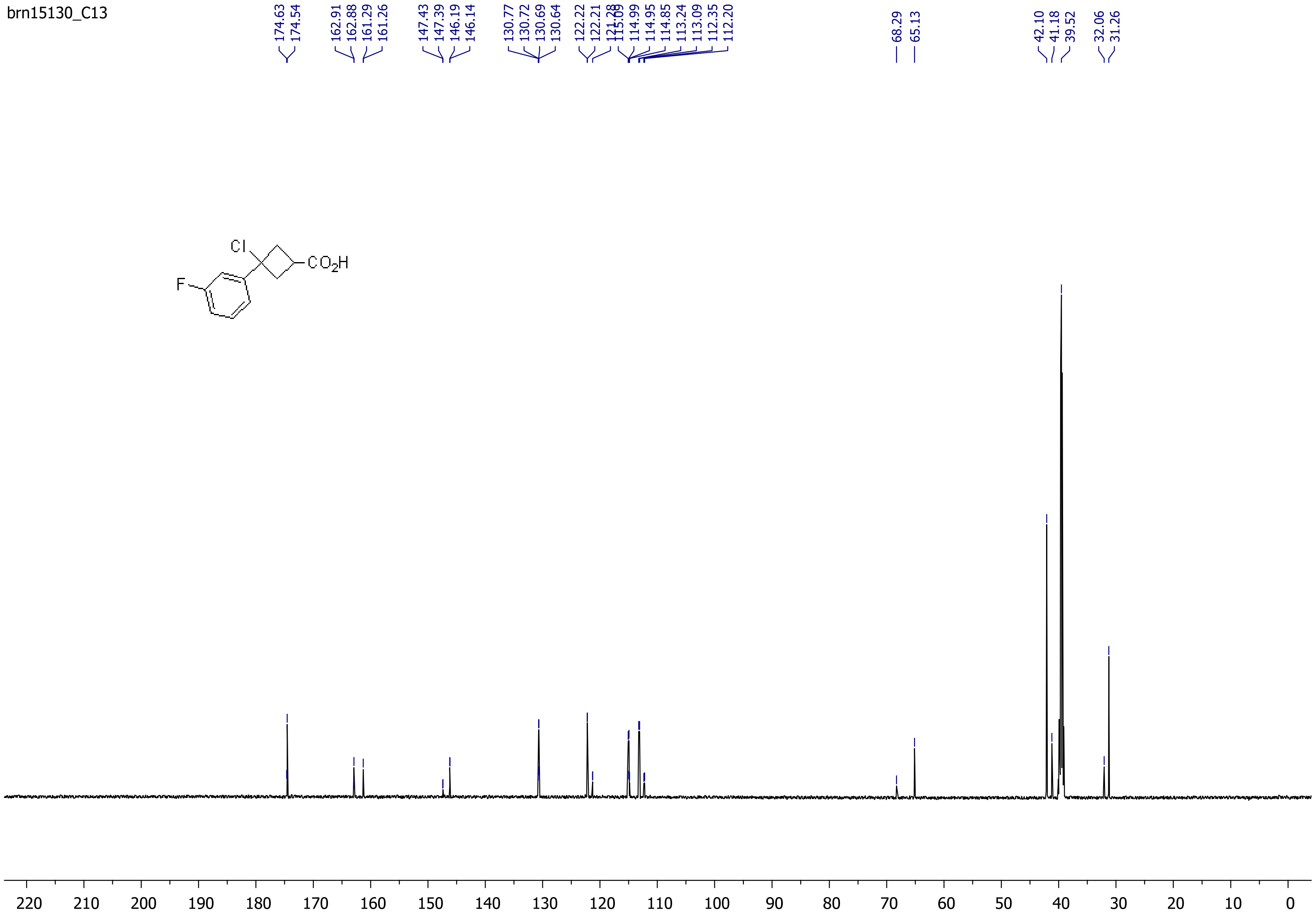

$^{19}F\{^{1}H\}$ NMR (376 MHz, DMSO-$d_6$)

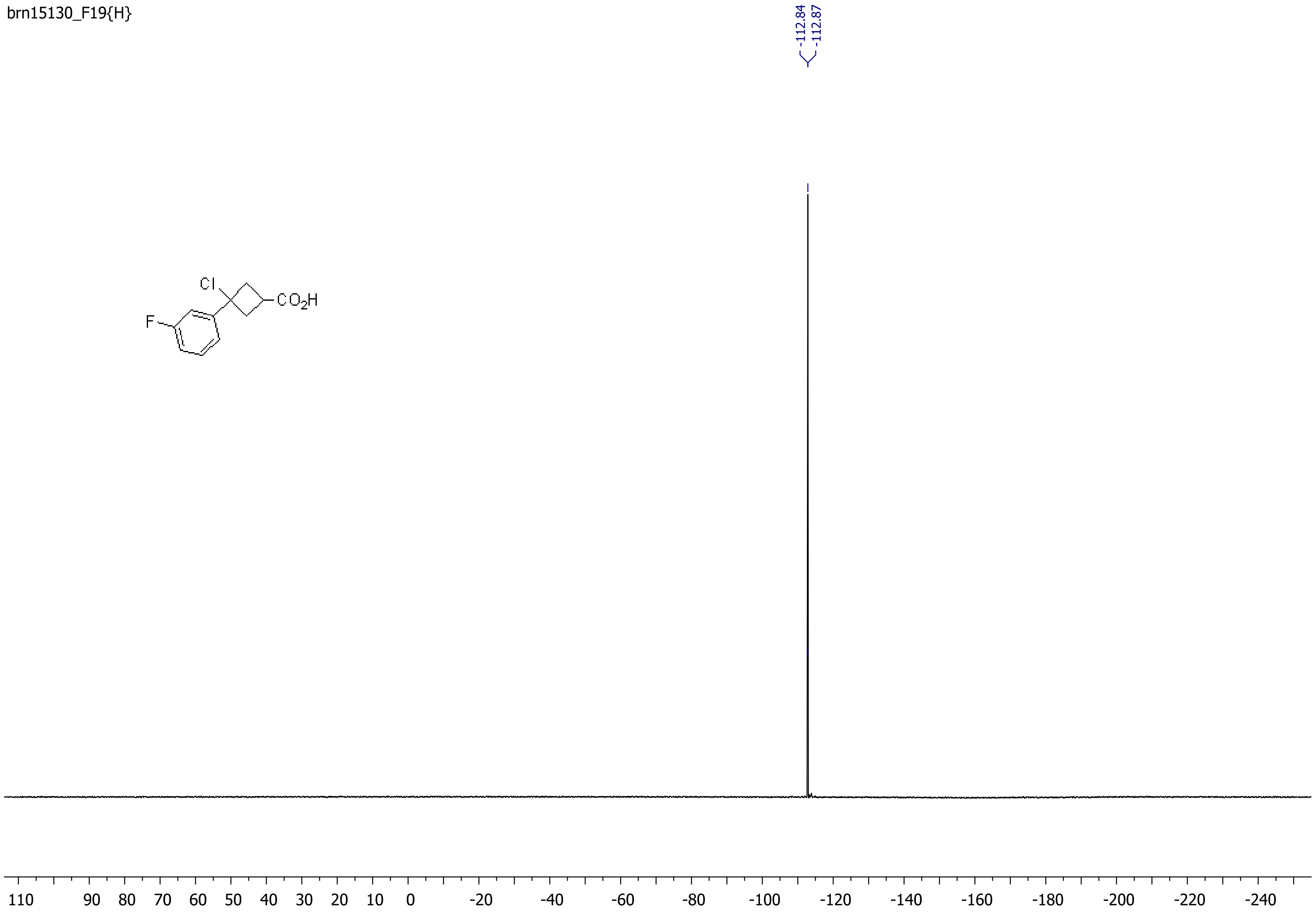

**3-Chloro-3-(4-fluorophenyl)cyclobutane-1-carboxylic acid**

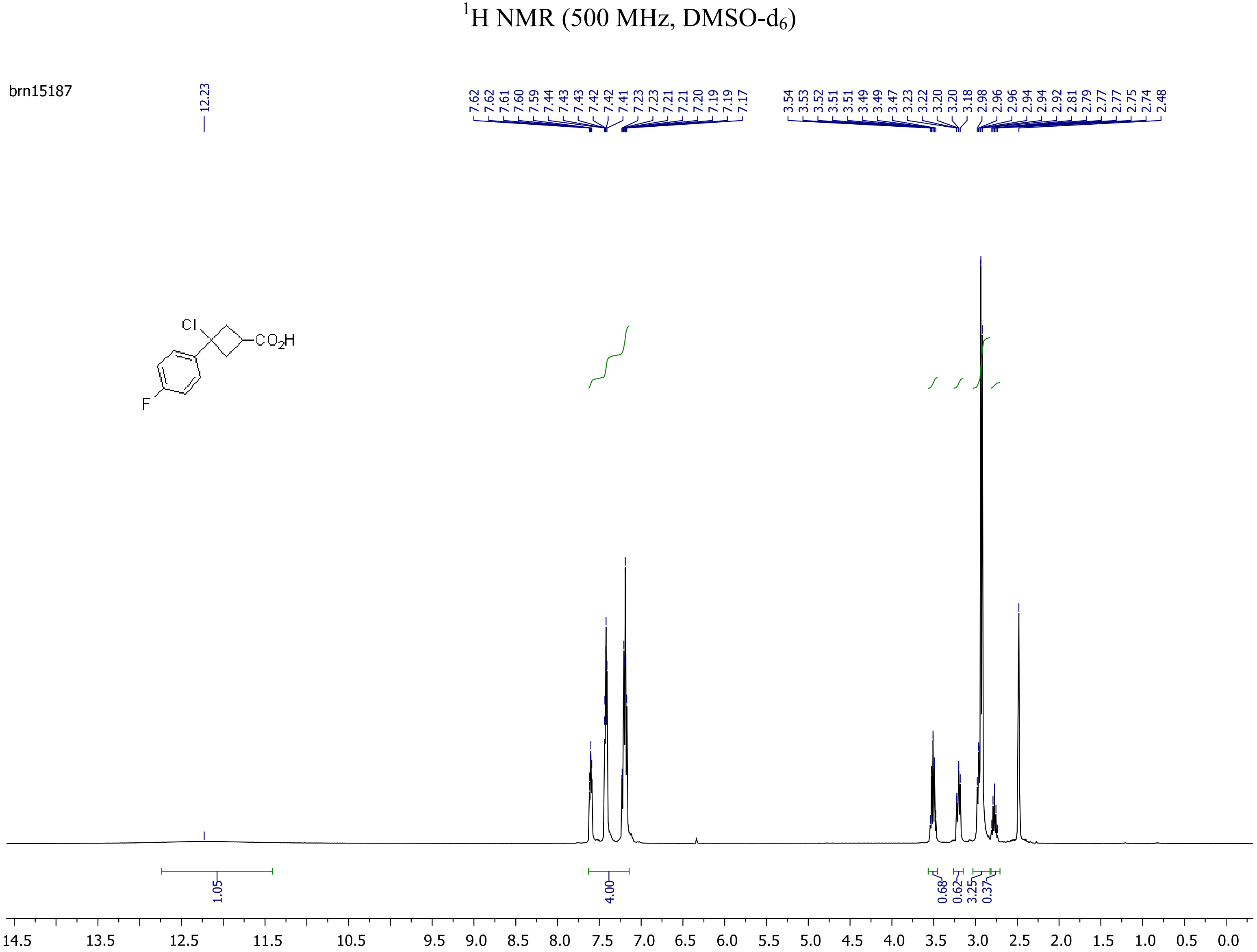

$^{13}C\{^1H\}$ NMR (151 MHz, DMSO-$d_6$)

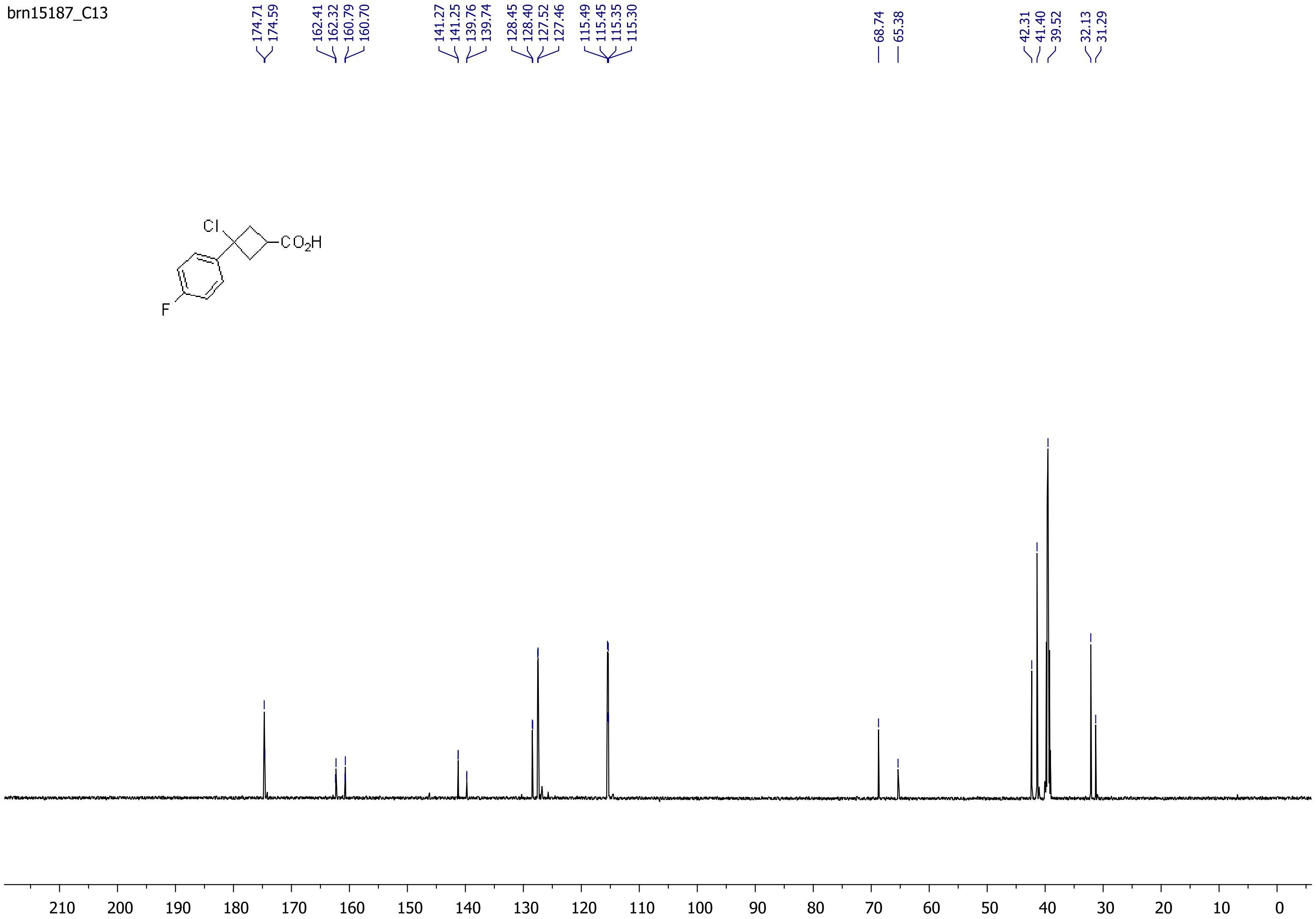

$^{19}F\{^{1}H\}$ NMR (376 MHz, DMSO-$d_6$)

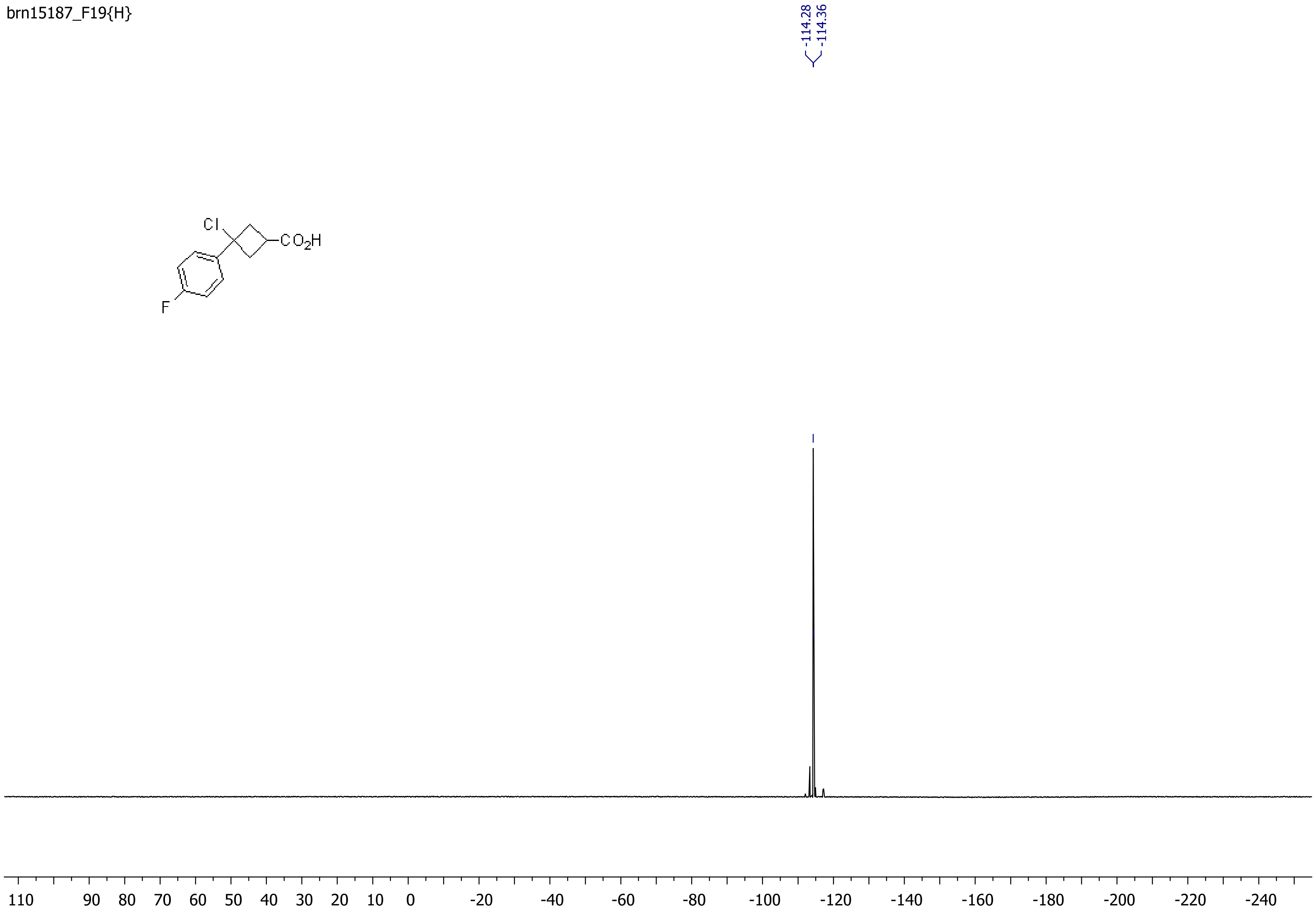

**3-Chloro-3-(3-(trifluoromethyl)phenyl)cyclobutane-1-carboxylic acid**

$^{1}$H NMR (400 MHz, DMSO-d$_6$)

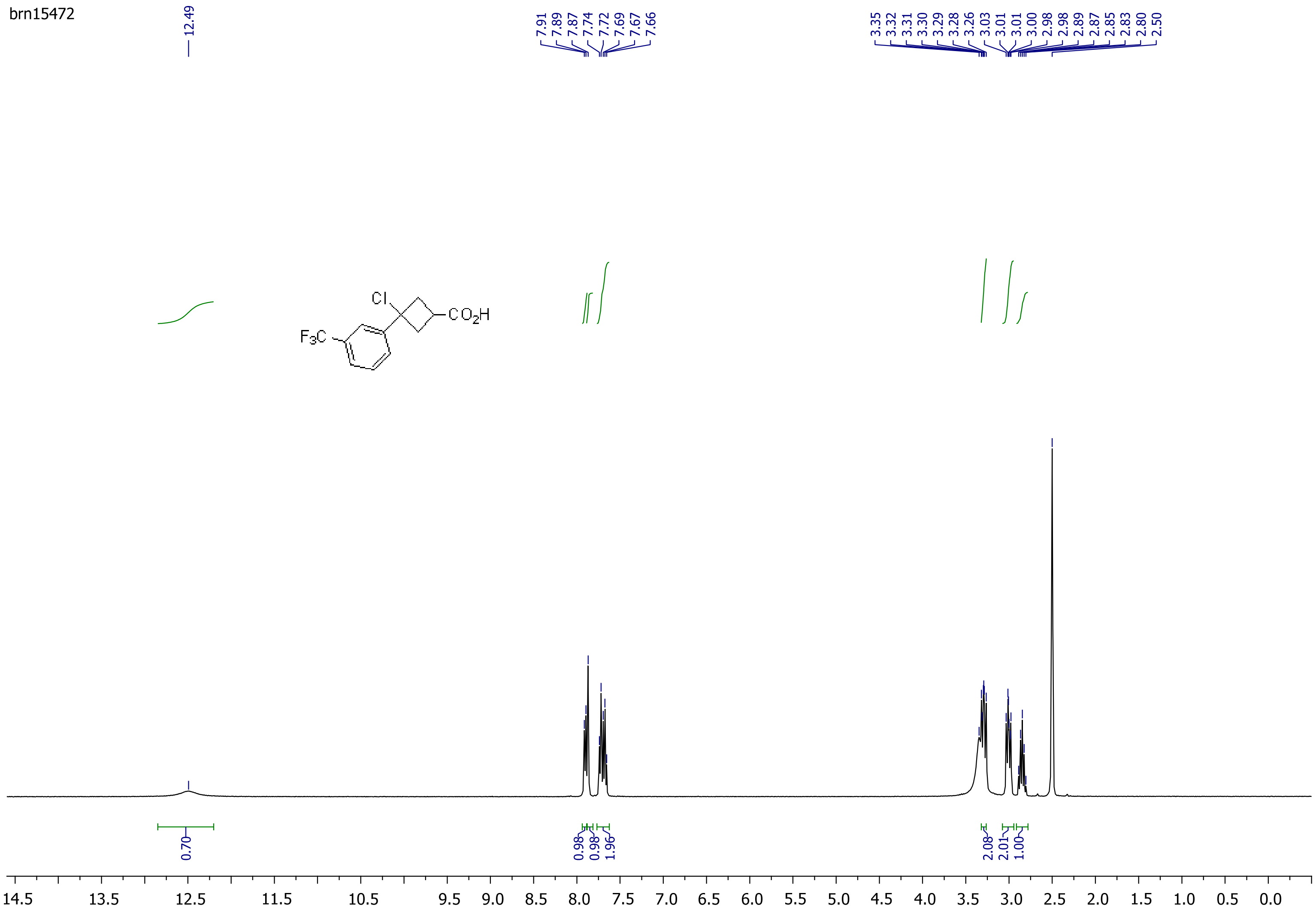

$^{13}C\{^{1}H\}$ NMR (126 MHz, DMSO-$d_6$)

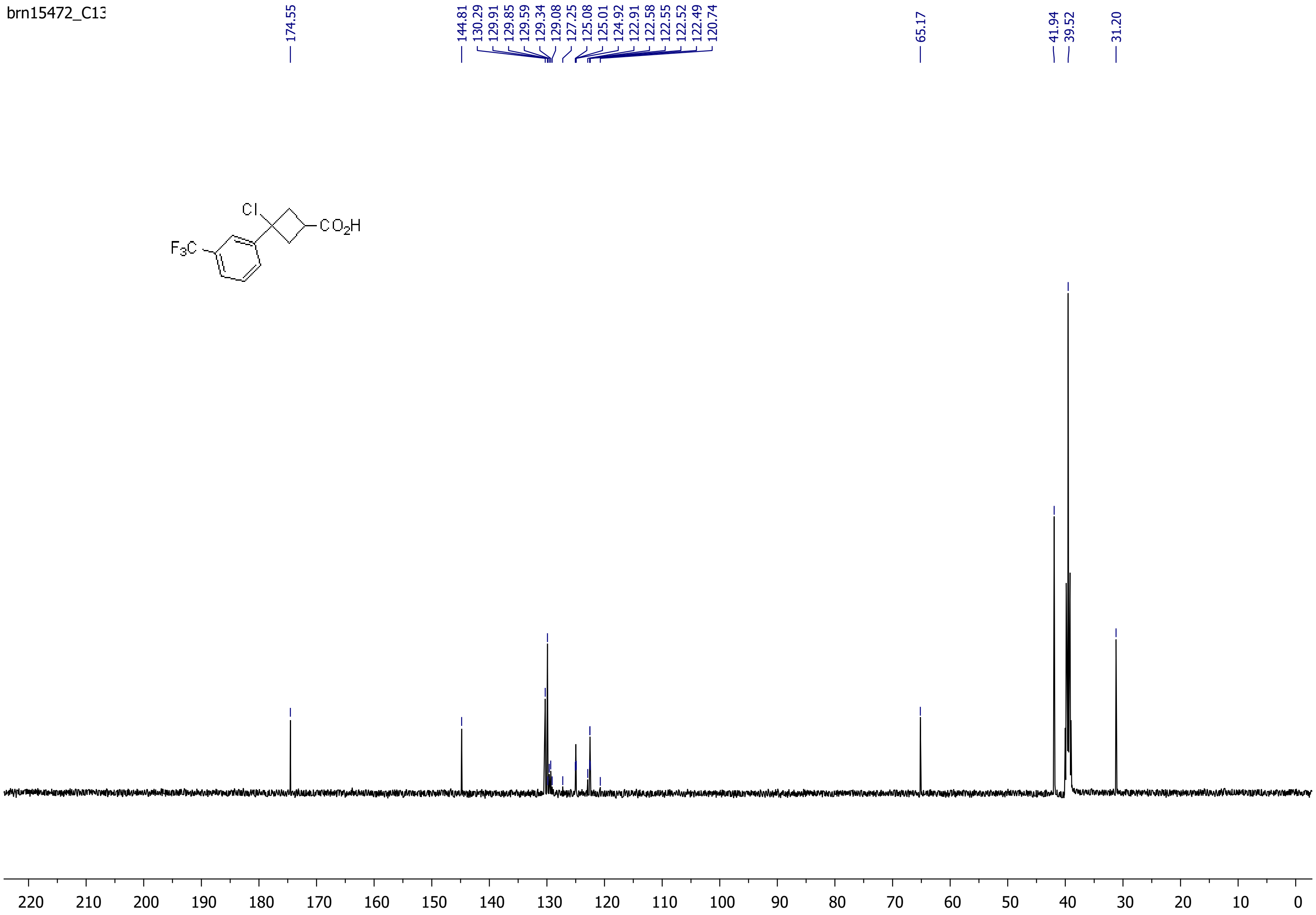

$^{19}F\{^1H\}$ NMR (376 MHz DMSO-$d_6$)

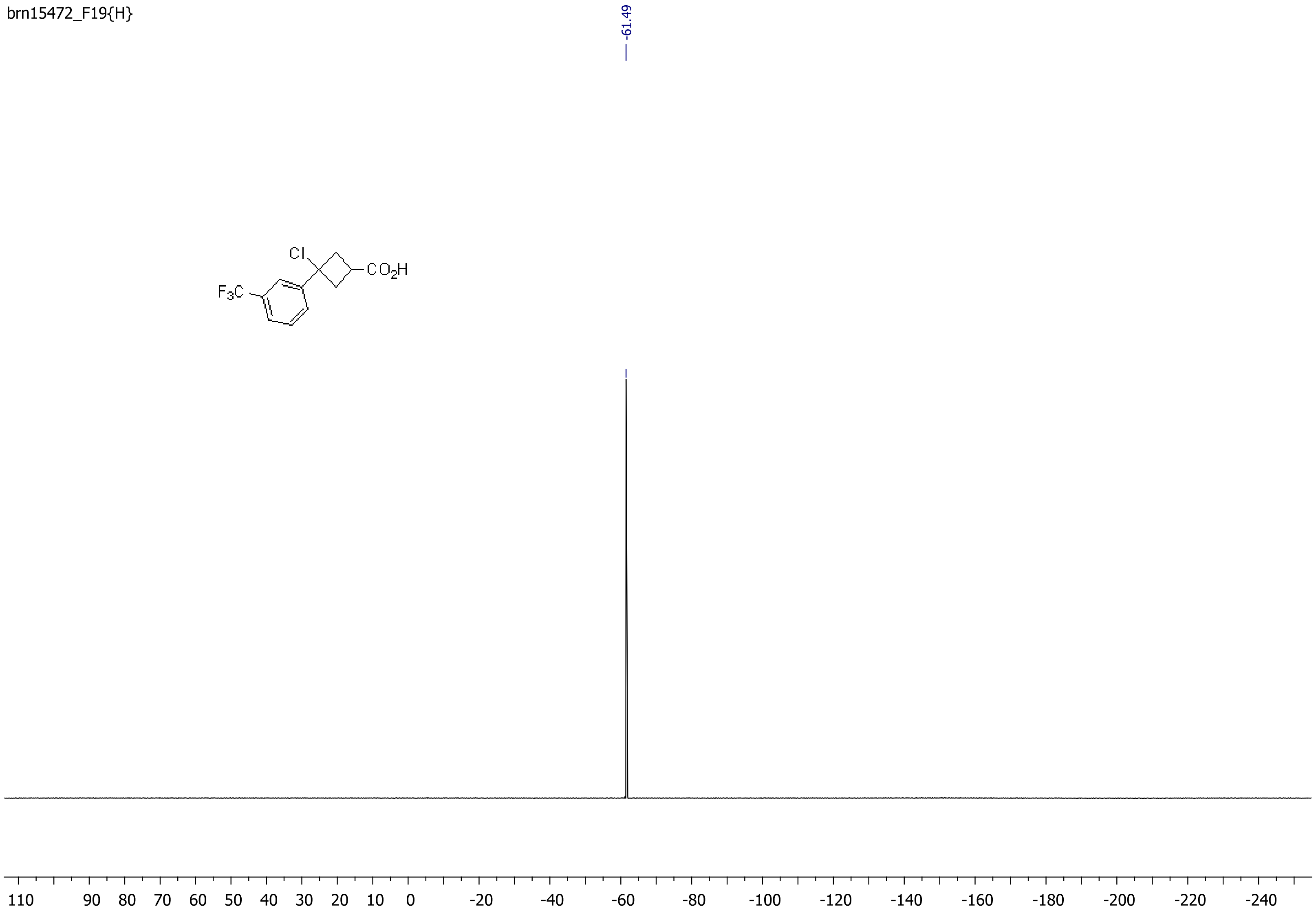

**3-Chloro-3-(4-(trifluoromethyl)phenyl)cyclobutane-1-carboxylic acid**

$^1H$ NMR (500 MHz, DMSO-$d_6$)

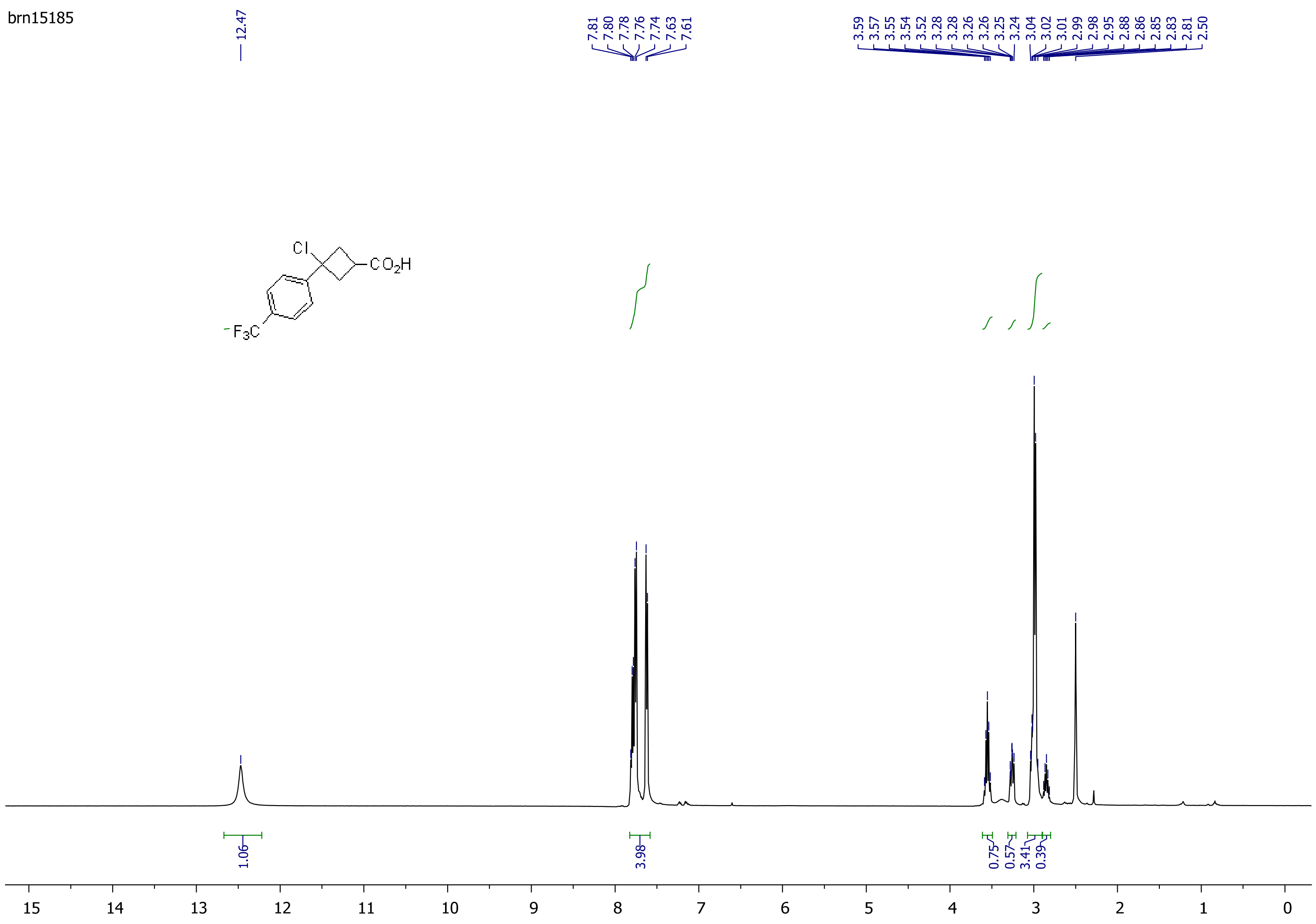

$^{13}C\{^1H\}$ NMR (151 MHz, DMSO-$d_6$)

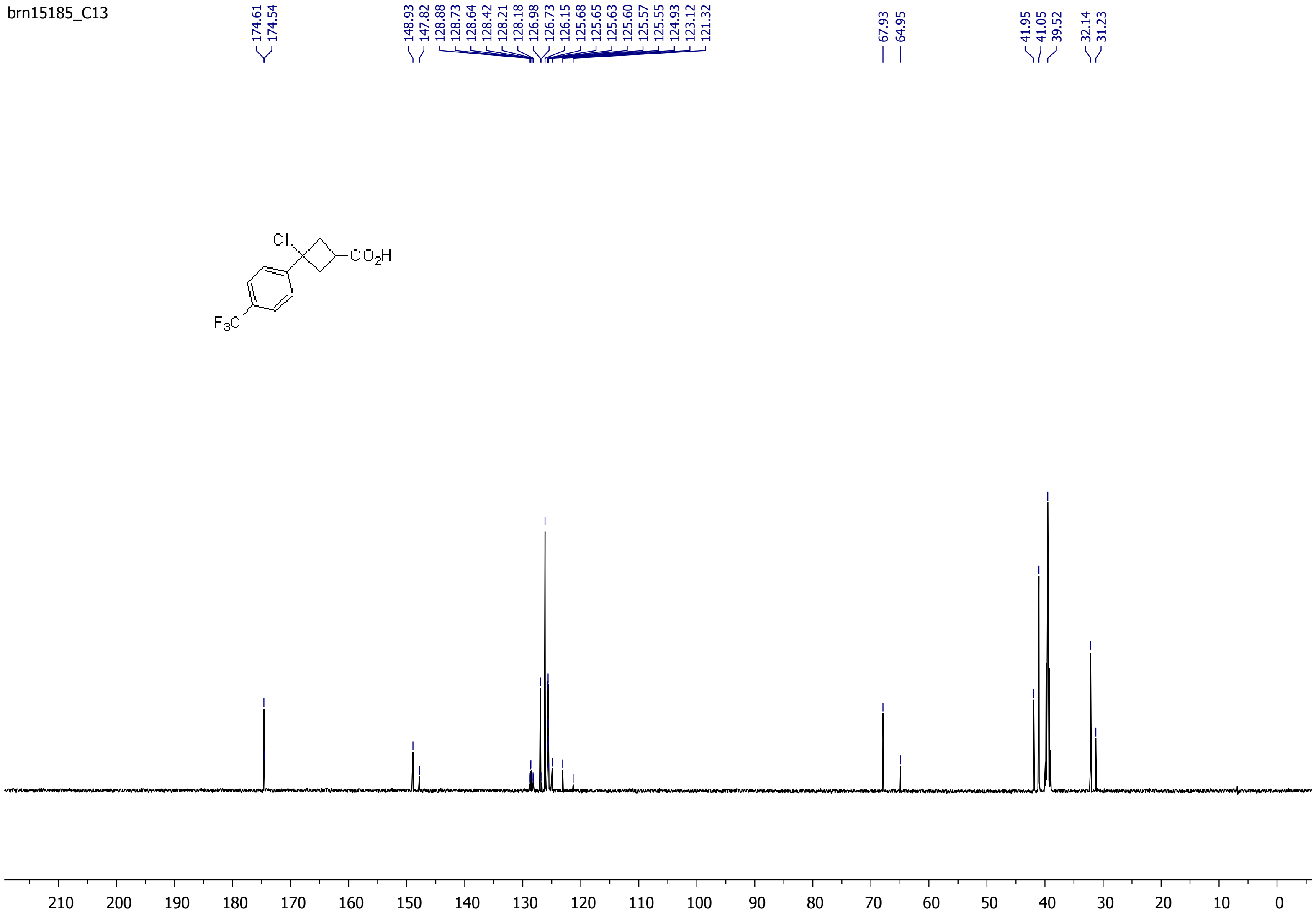

$^{19}F\{^{1}H\}$ NMR (376 MHz, DMSO-$d_6$)

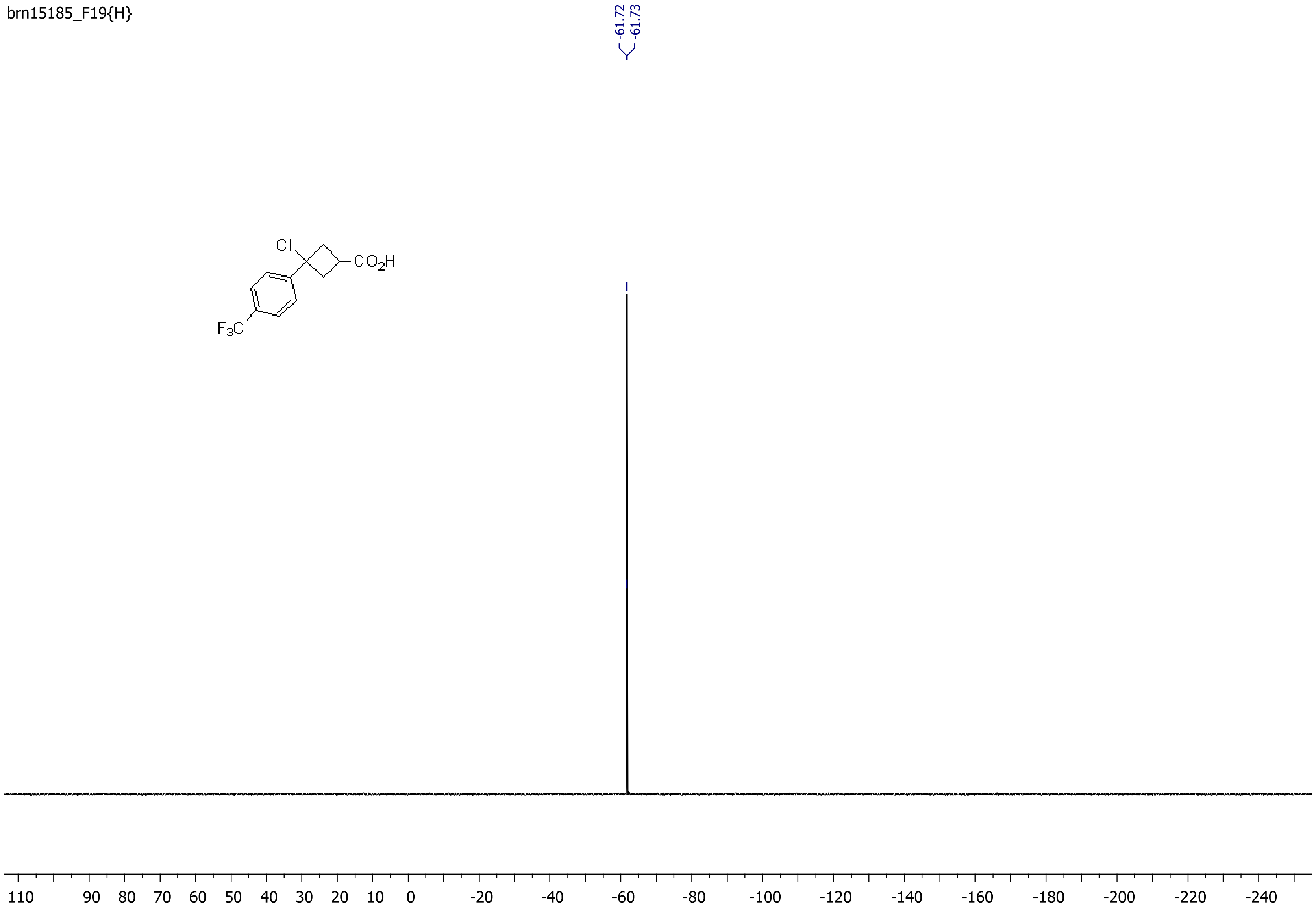

**Compound 11**

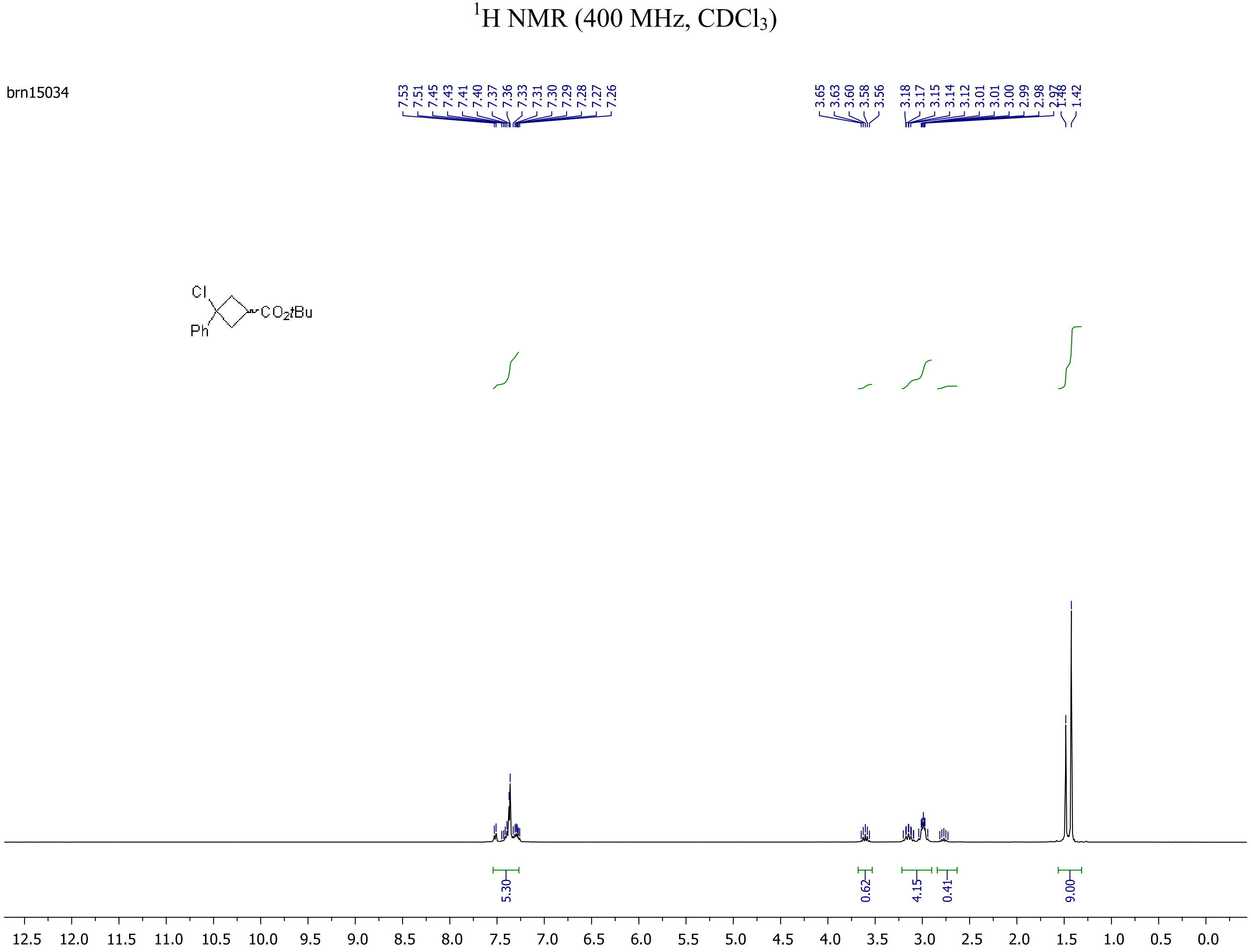

$^1$H NMR (400 MHz, CDCl$_3$)
brn15034
7.53 7.51 7.45 7.43 7.41 7.40 7.37 7.36 7.33 7.31 7.30 7.29 7.28 7.27 7.26
3.65 3.63 3.60 3.58 3.56 3.18 3.17 3.15 3.14 3.12 3.01 3.00 2.99 2.98 2.97 1.48 1.42
Cl
Ph
CO2tBu
5.30
0.62
4.15
0.41
9.00
12.5 12.0 11.5 11.0 10.5 10.0 9.5 9.0 8.5 8.0 7.5 7.0 6.5 6.0 5.5 5.0 4.5 4.0 3.5 3.0 2.5 2.0 1.5 1.0 0.5 0.0

$^{13}C\ \{^{1}H\}$ NMR (101 MHz, $CDCl_3$)

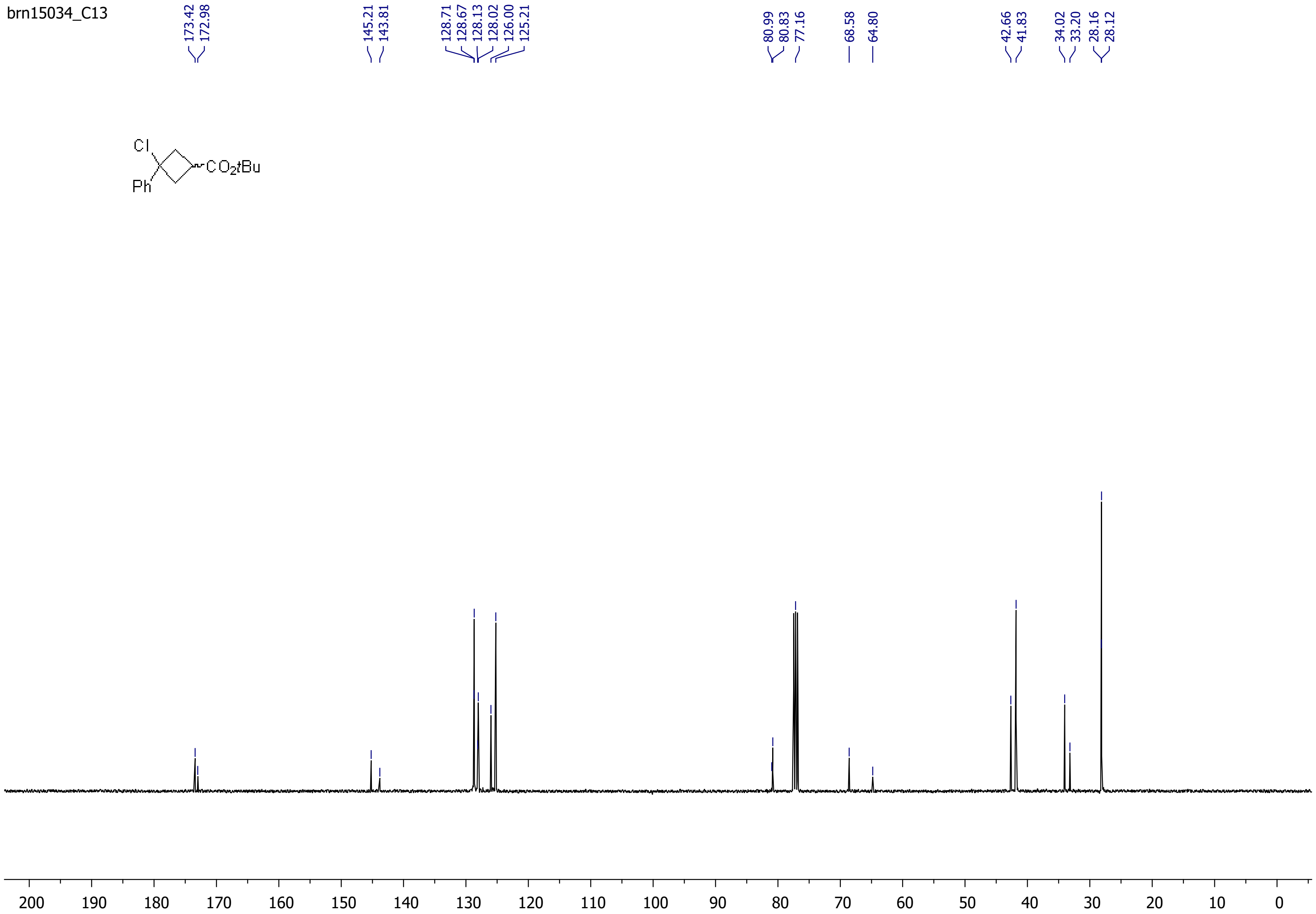

**Tert-butyl 3-chloro-3-(*o*-tolyl)cyclobutane-1-carboxylate**

$^1$H NMR (500 MHz, $CDCl_3$)

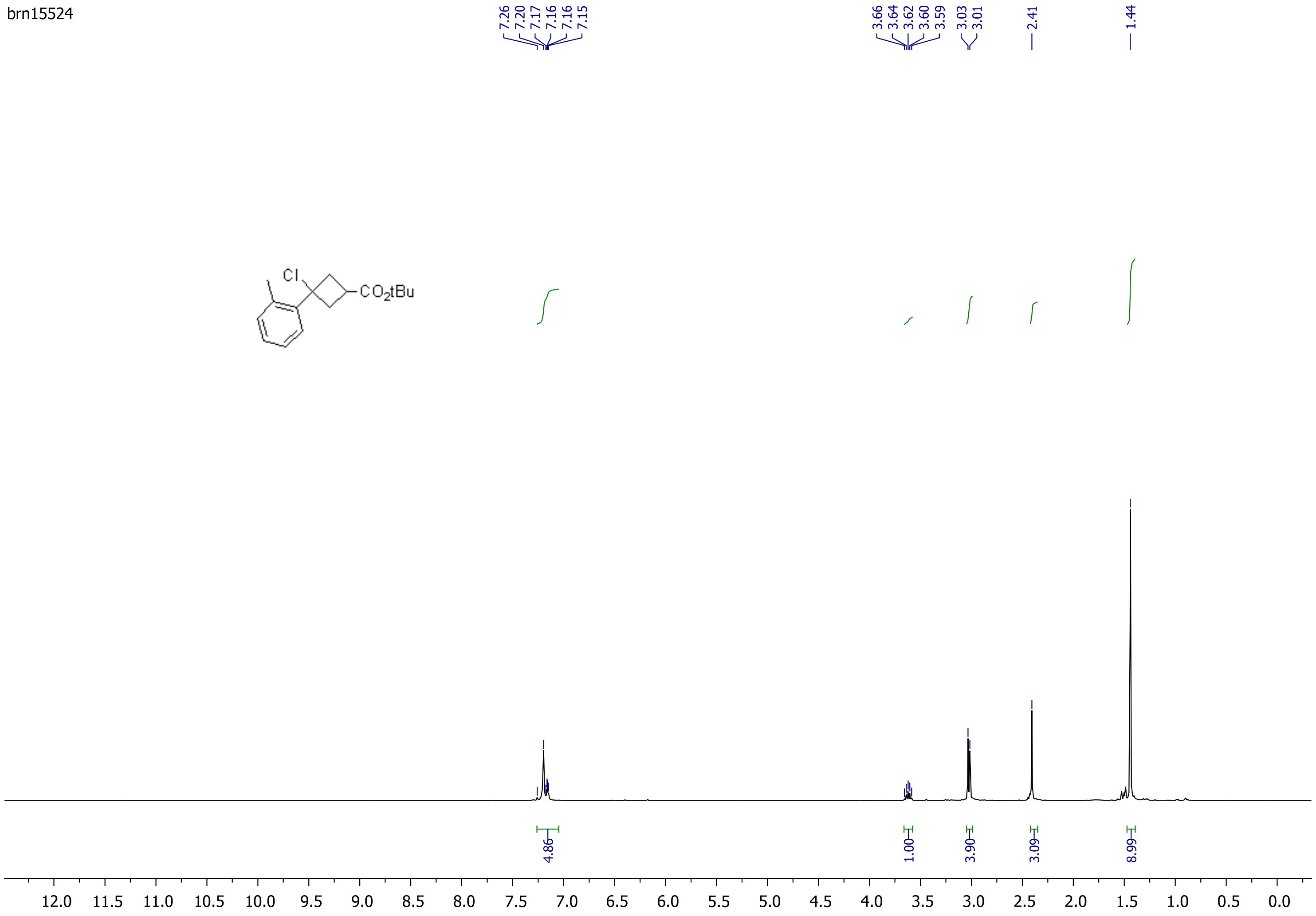

## $^{13}C\{^1H\}$ NMR (151 MHz, $CDCl_3$)

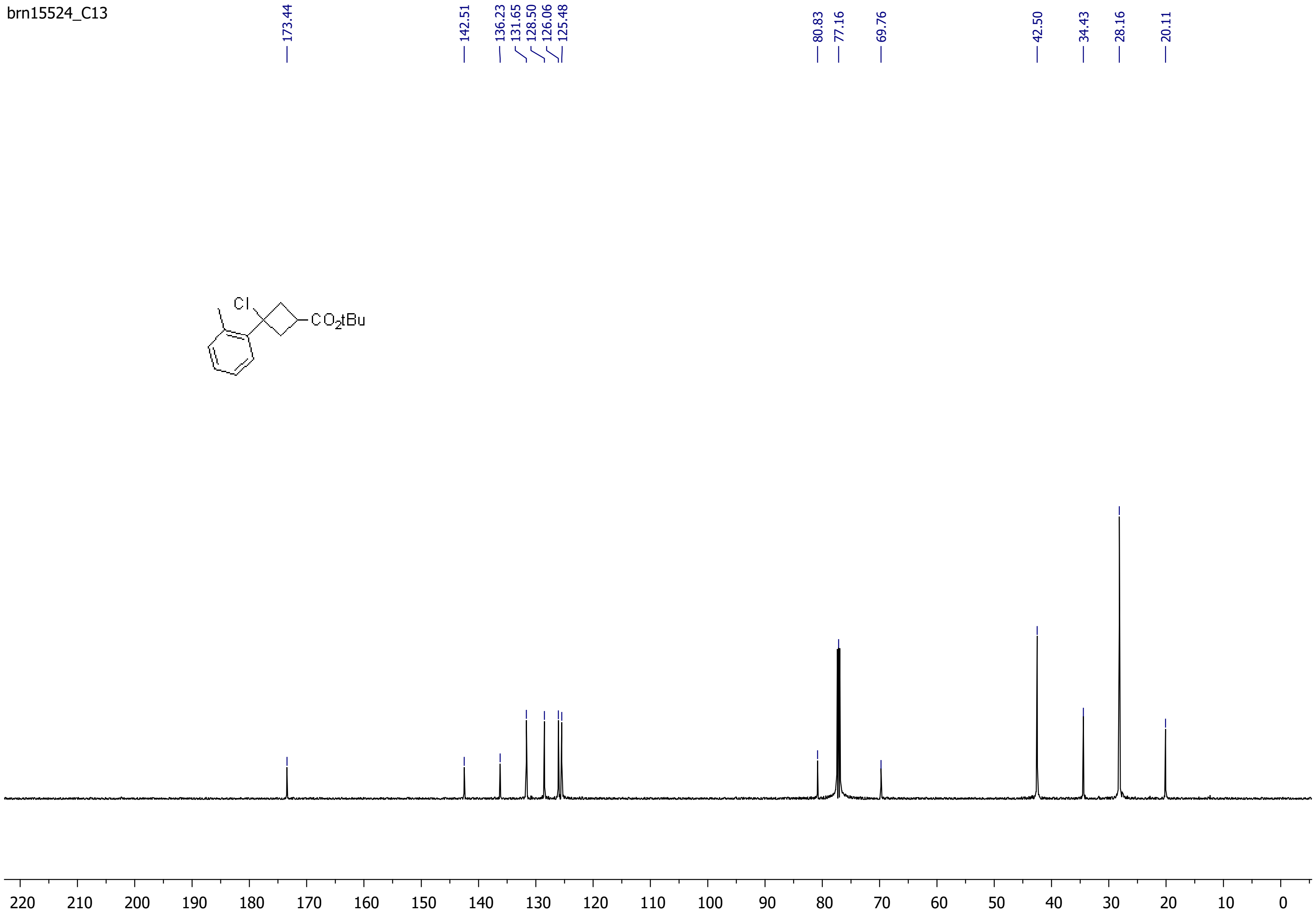

**Tert-butyl 3-chloro-3-(*m*-tolyl)cyclobutane-1-carboxylate**

$^1$H NMR (500 MHz, $CDCl_3$)

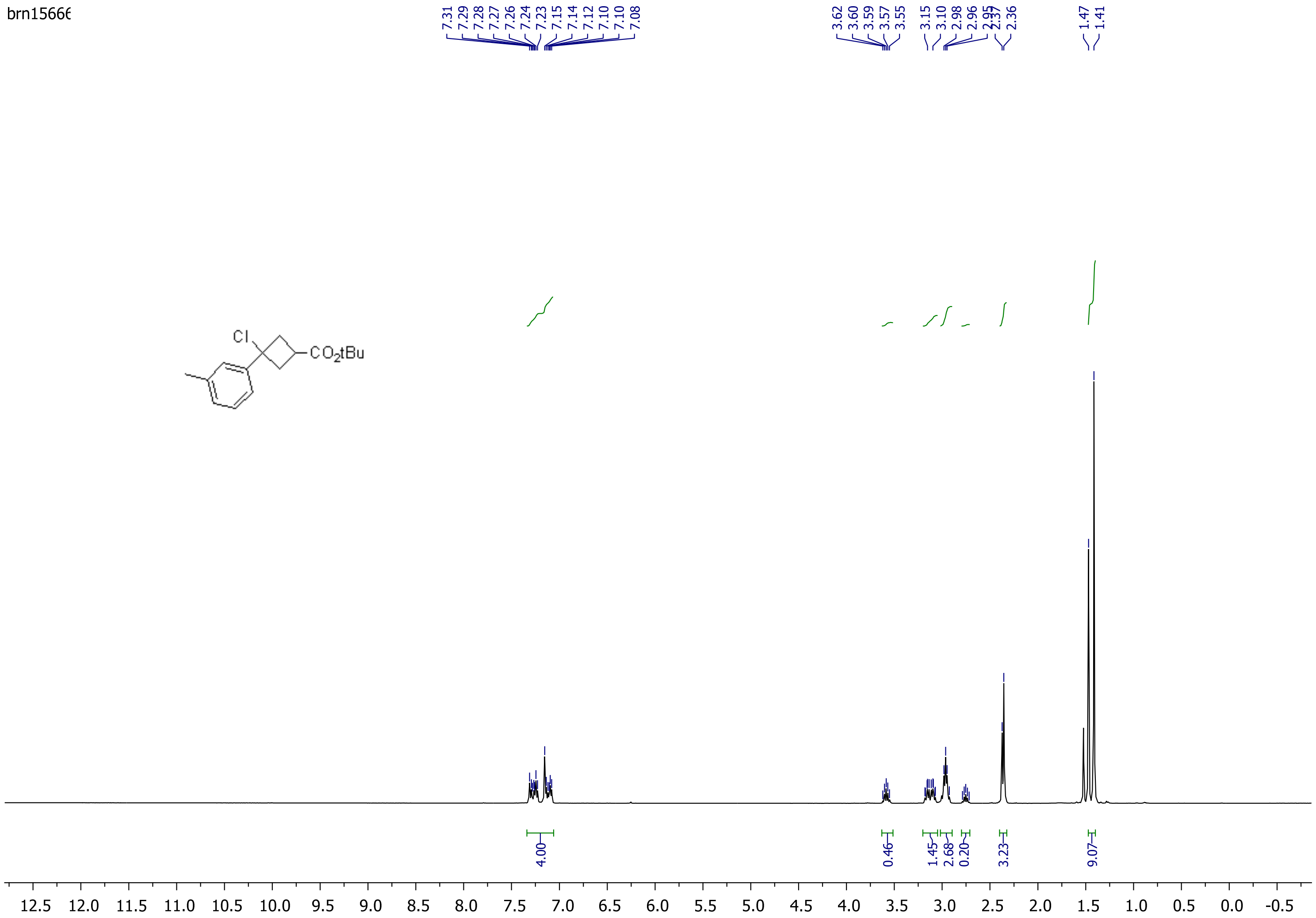

$^{13}C\{^1H\}$ NMR (126 MHz, $CDCl_3$)

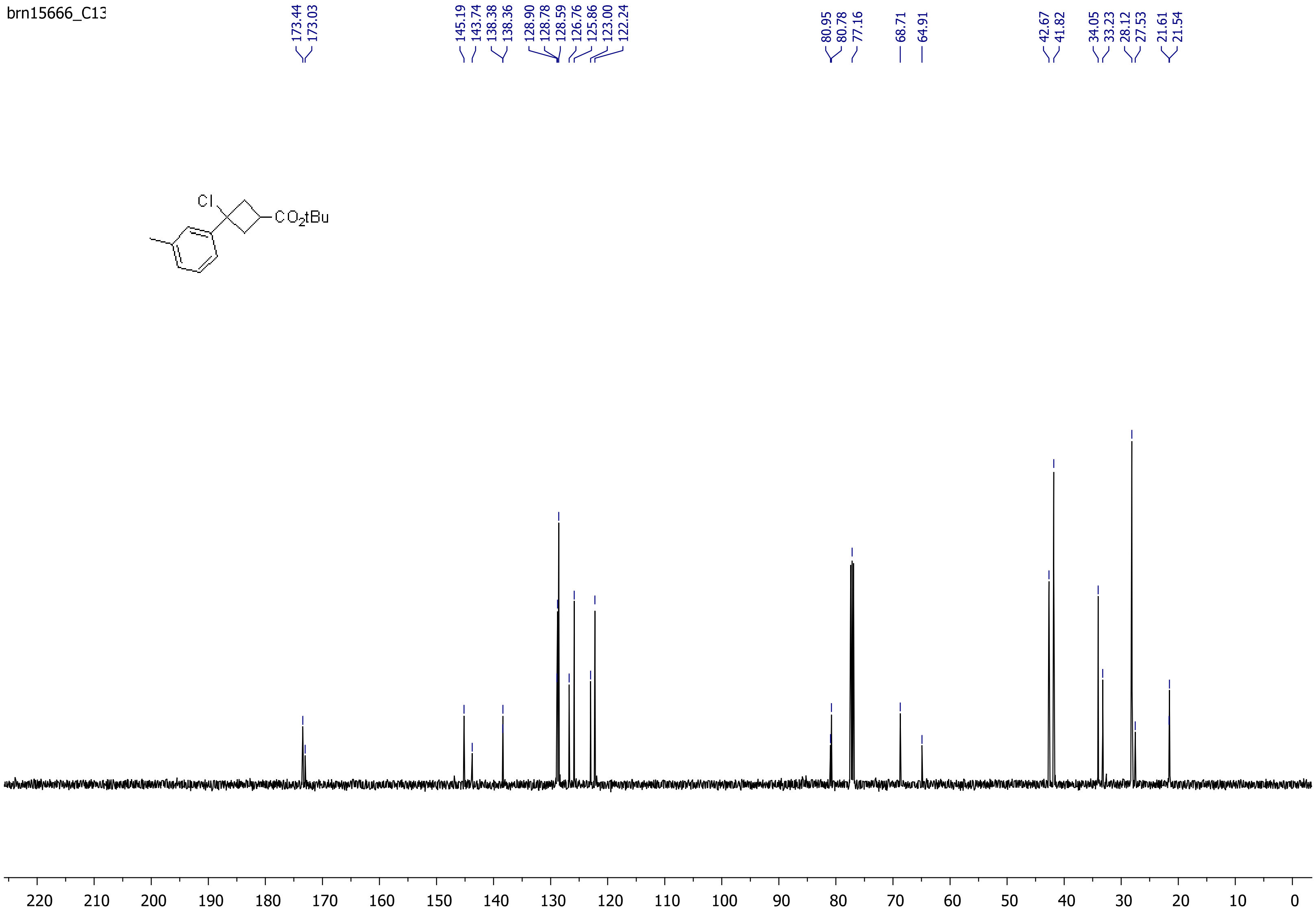

**Tert-butyl 3-chloro-3-(*p*-tolyl)cyclobutane-1-carboxylate**

$^{1}$H NMR (500 MHz, $CDCl_3$)

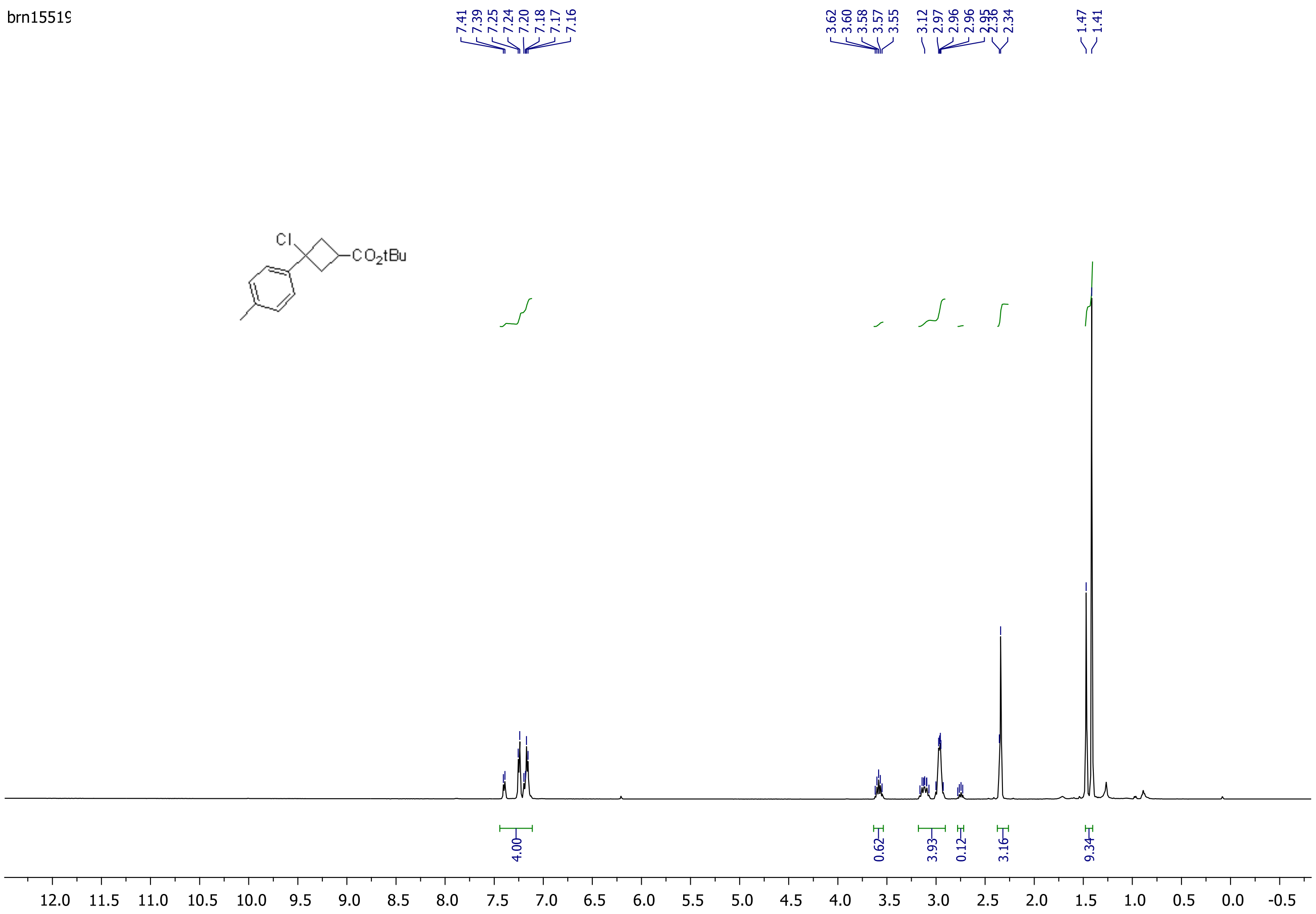

$^{13}C\{^1H\}$ NMR (126 MHz, $CDCl_3$)

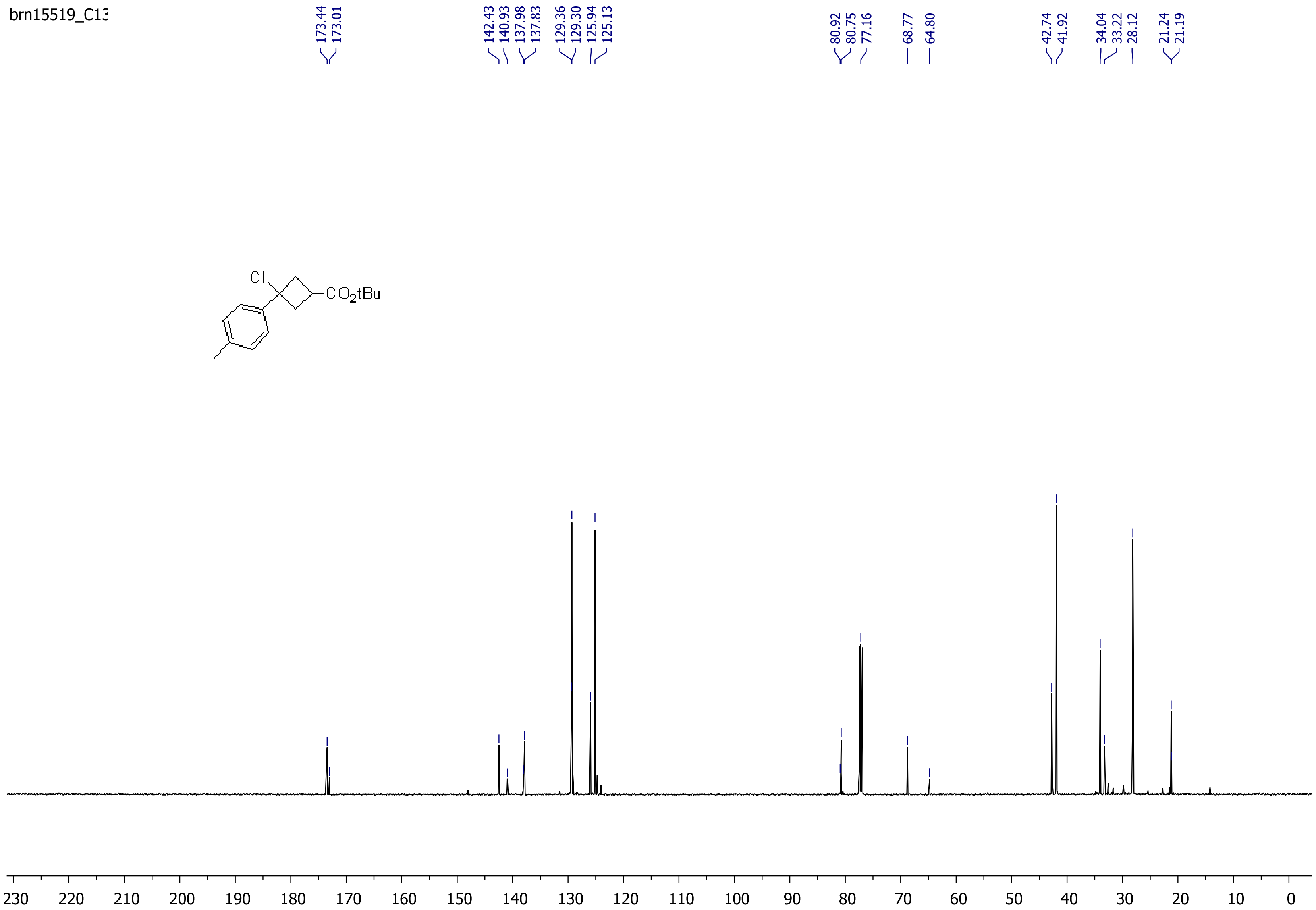

**Tert-butyl 3-chloro-3-(3-fluorophenyl)cyclobutane-1-carboxylate**

$^{1}$H NMR (400 MHz, $CDCl_3$)

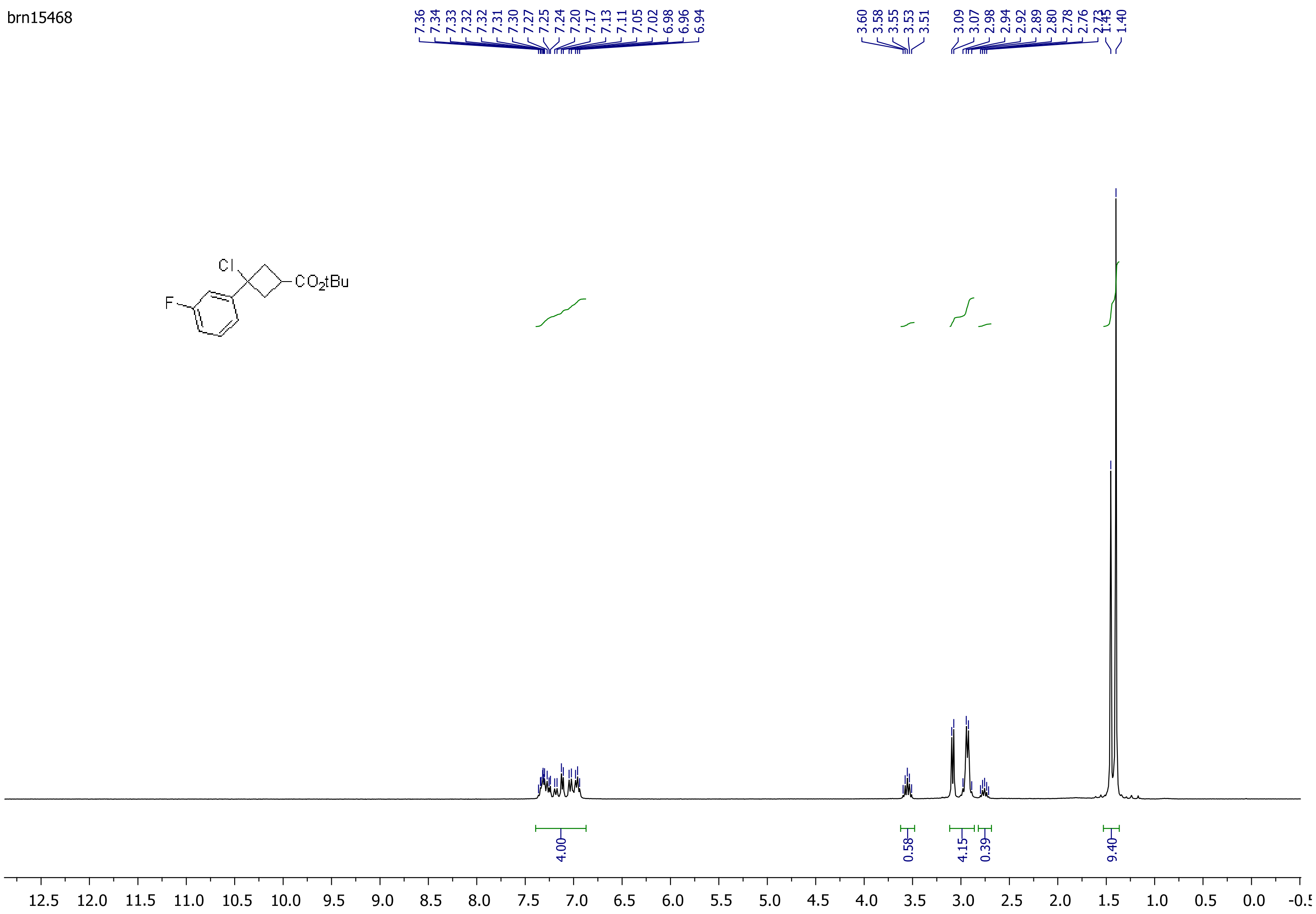

$^{13}C\{^{1}H\}$ NMR (151 MHz, $CDCl_3$)

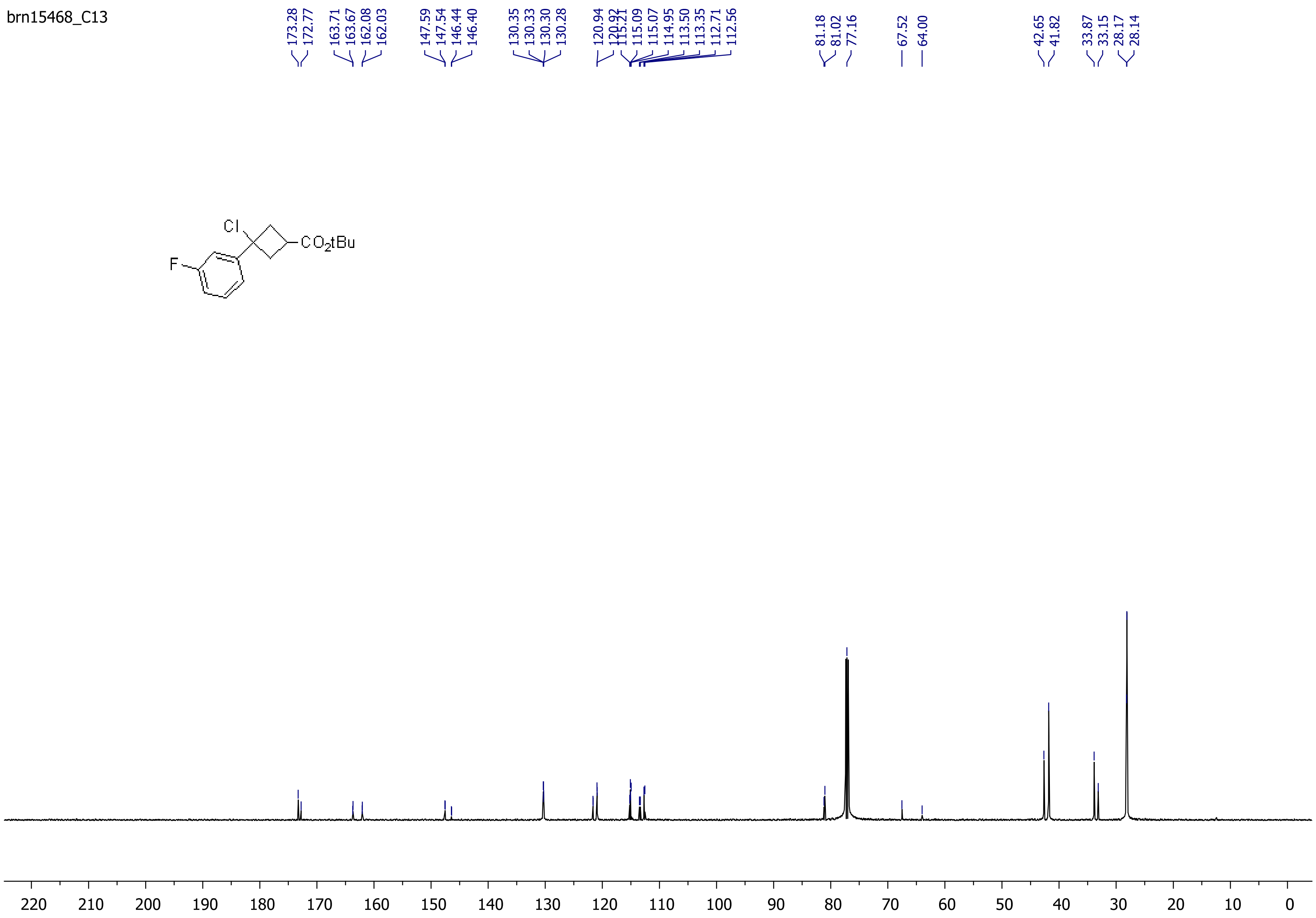

$^{19}F\{^1H\}$ NMR (376 MHz, $CDCl_3$)

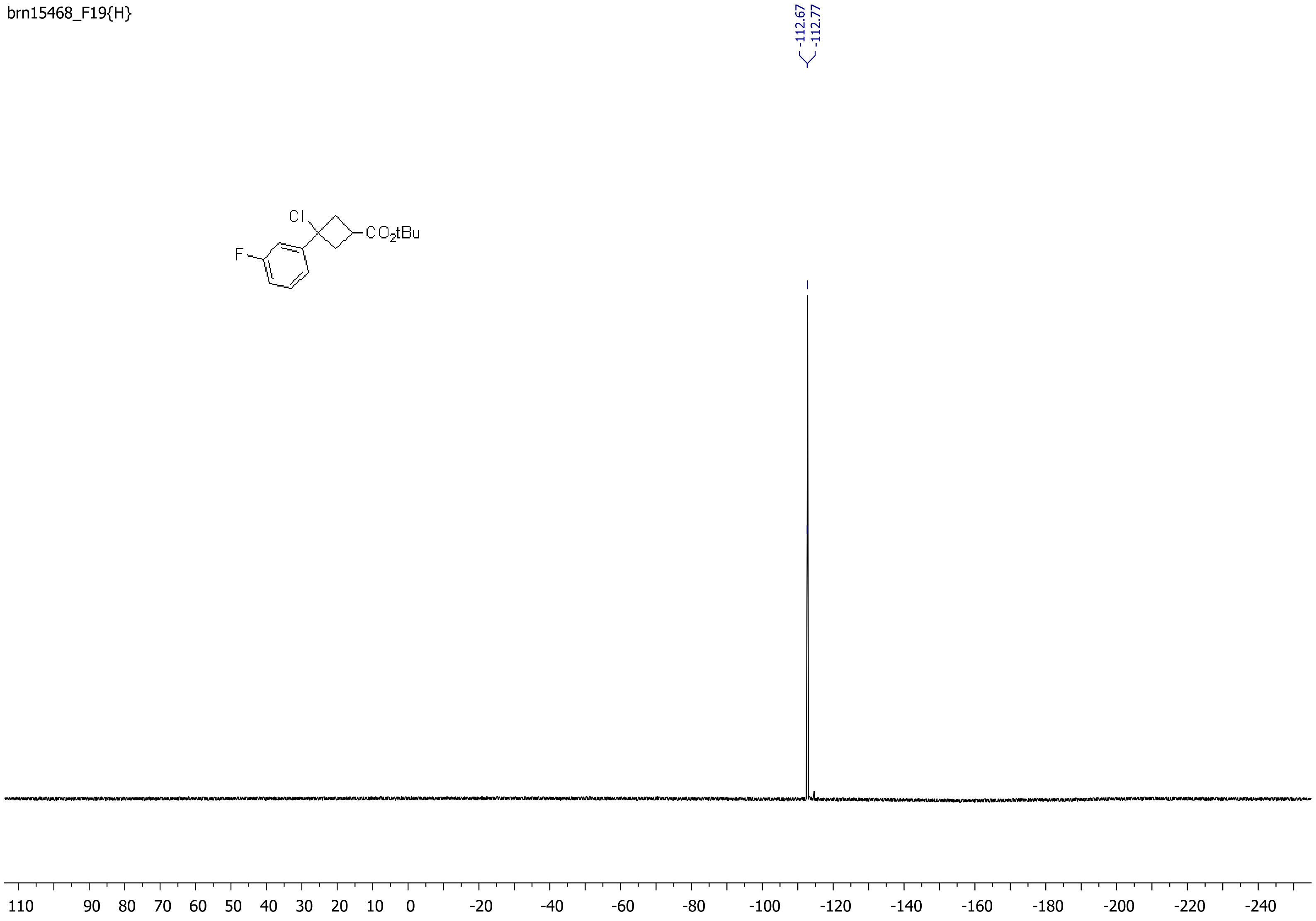

**Tert-butyl 3-chloro-3-(4-fluorophenyl)cyclobutane-1-carboxylate**

$^{1}$H NMR (400 MHz, $CDCl_3$)

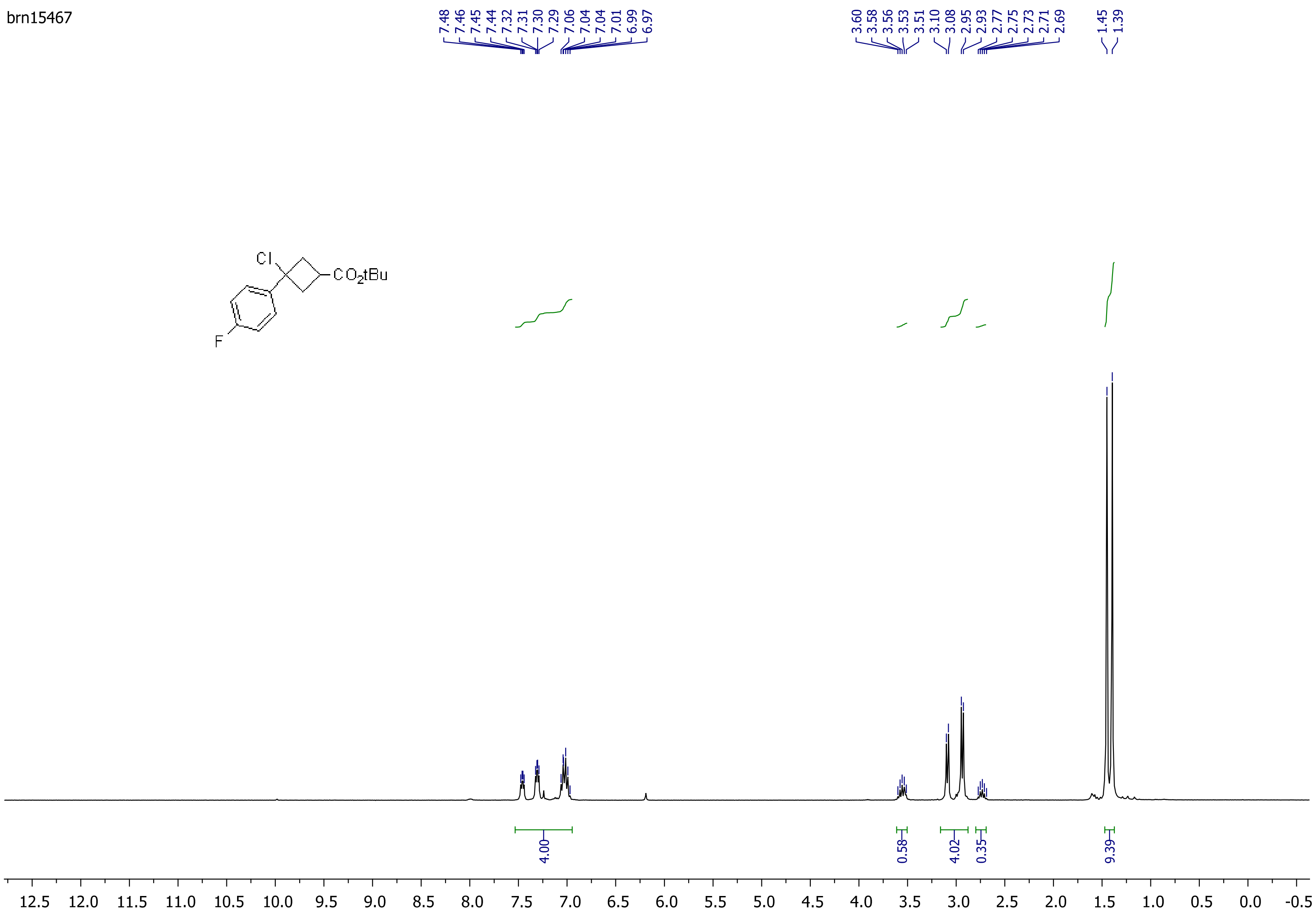

$^{13}C\{^{1}H\}$ NMR (151 MHz, $CDCl_3$)

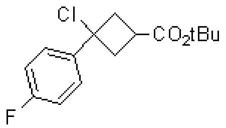


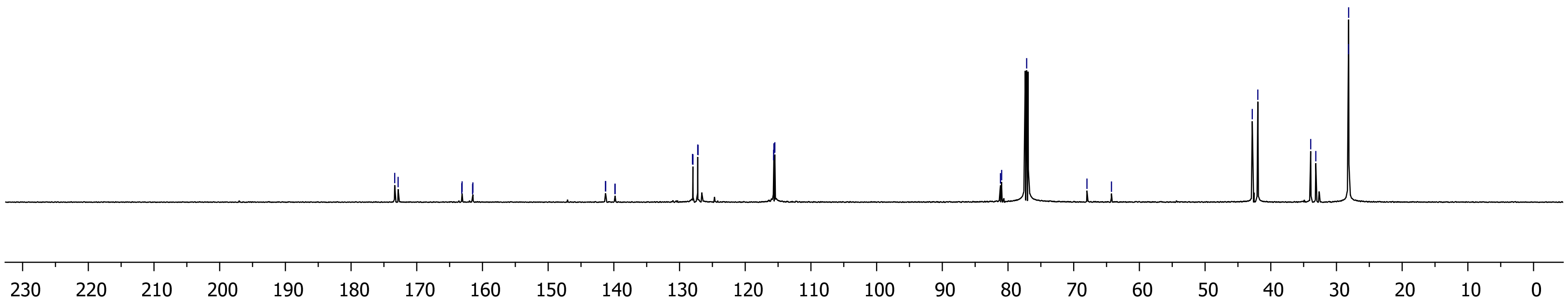

$^{19}F\{^{1}H\}$ NMR (376 MHz, $CDCl_3$)

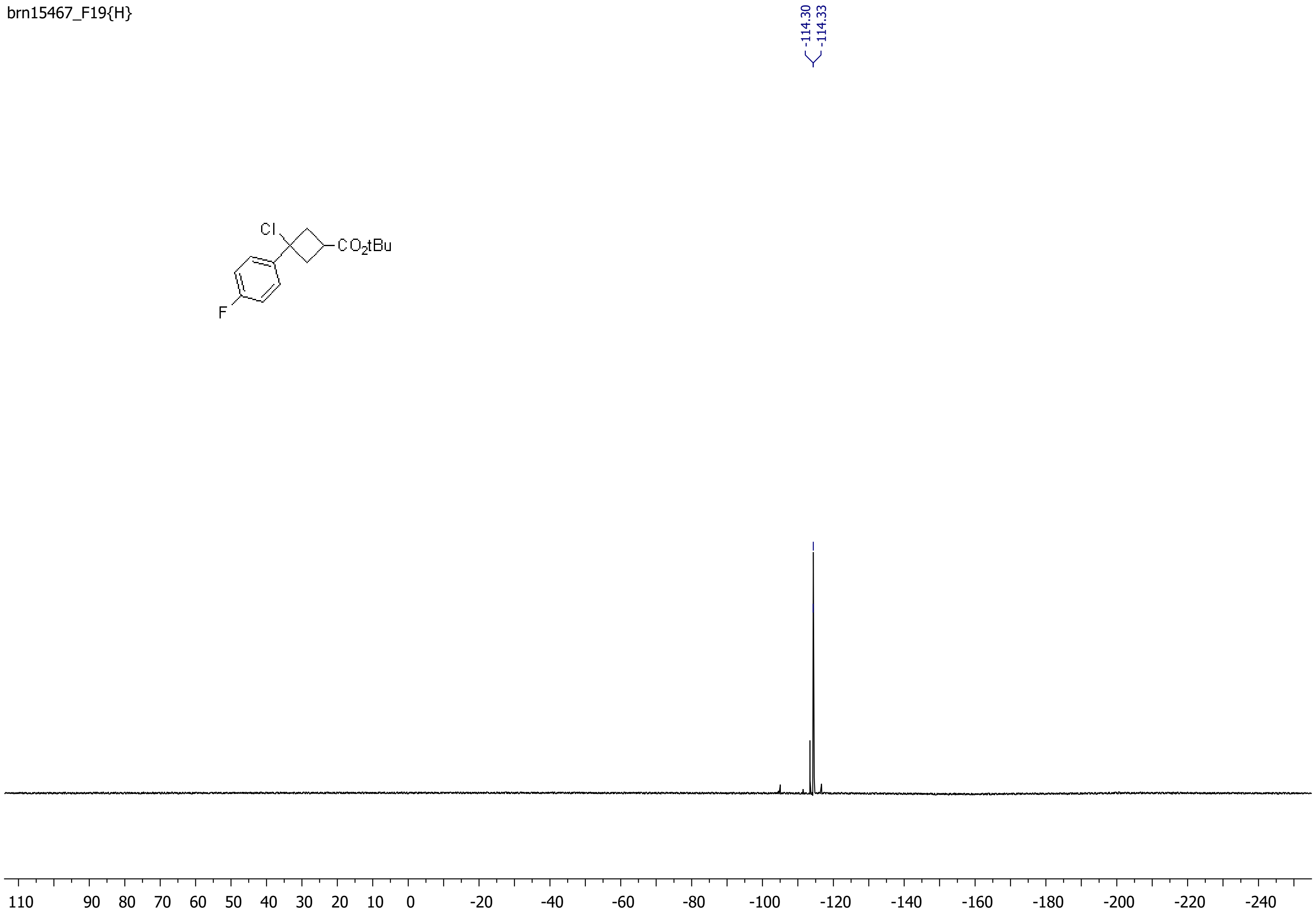

**Tert-butyl 3-chloro-3-(3-(trifluoromethyl)phenyl)cyclobutane-1-carboxylate**

$^1$H NMR (500 MHz, $CDCl_3$)

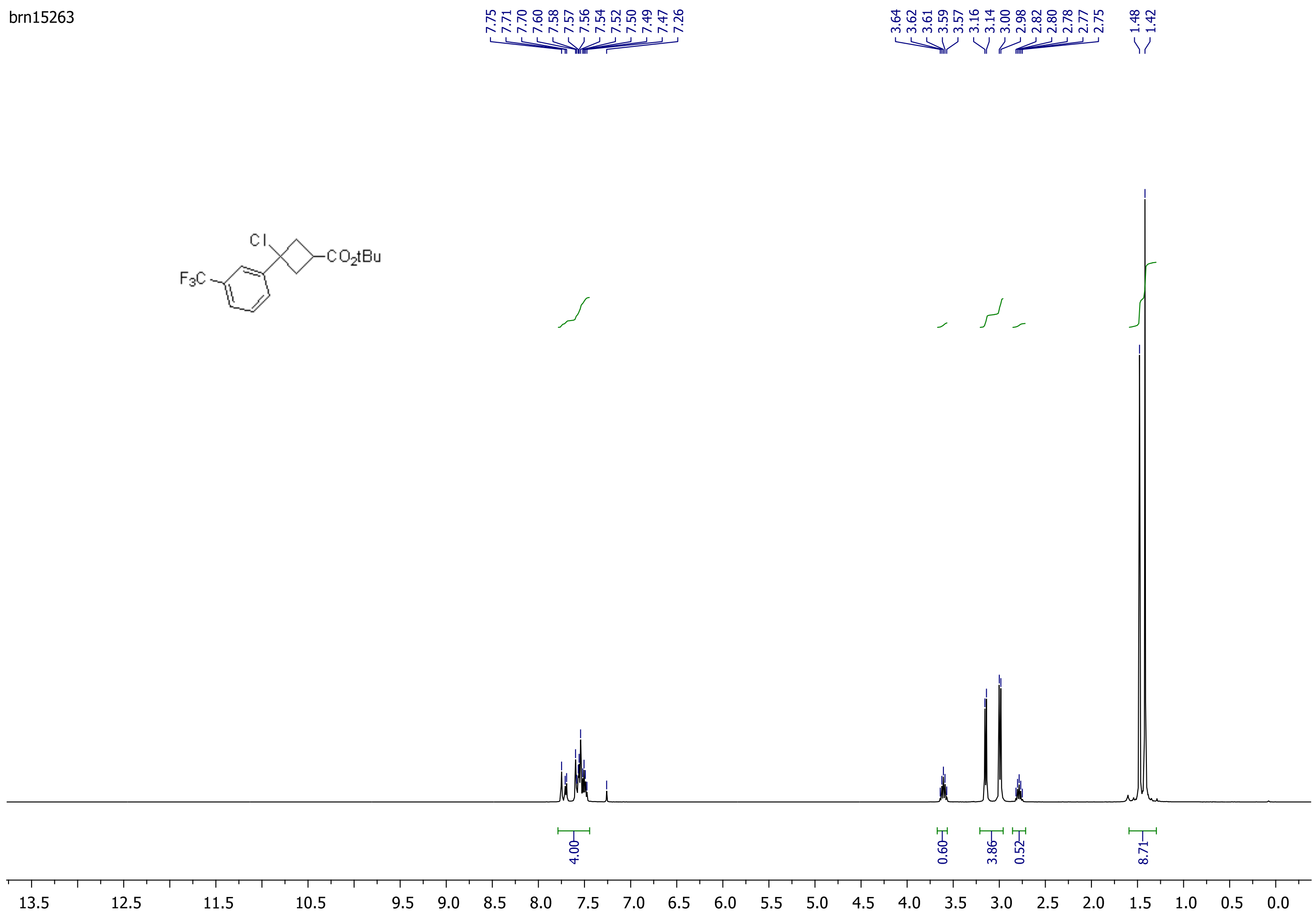

$^{13}C\{^{1}H\}$ NMR (151 MHz, $CDCl_3$)

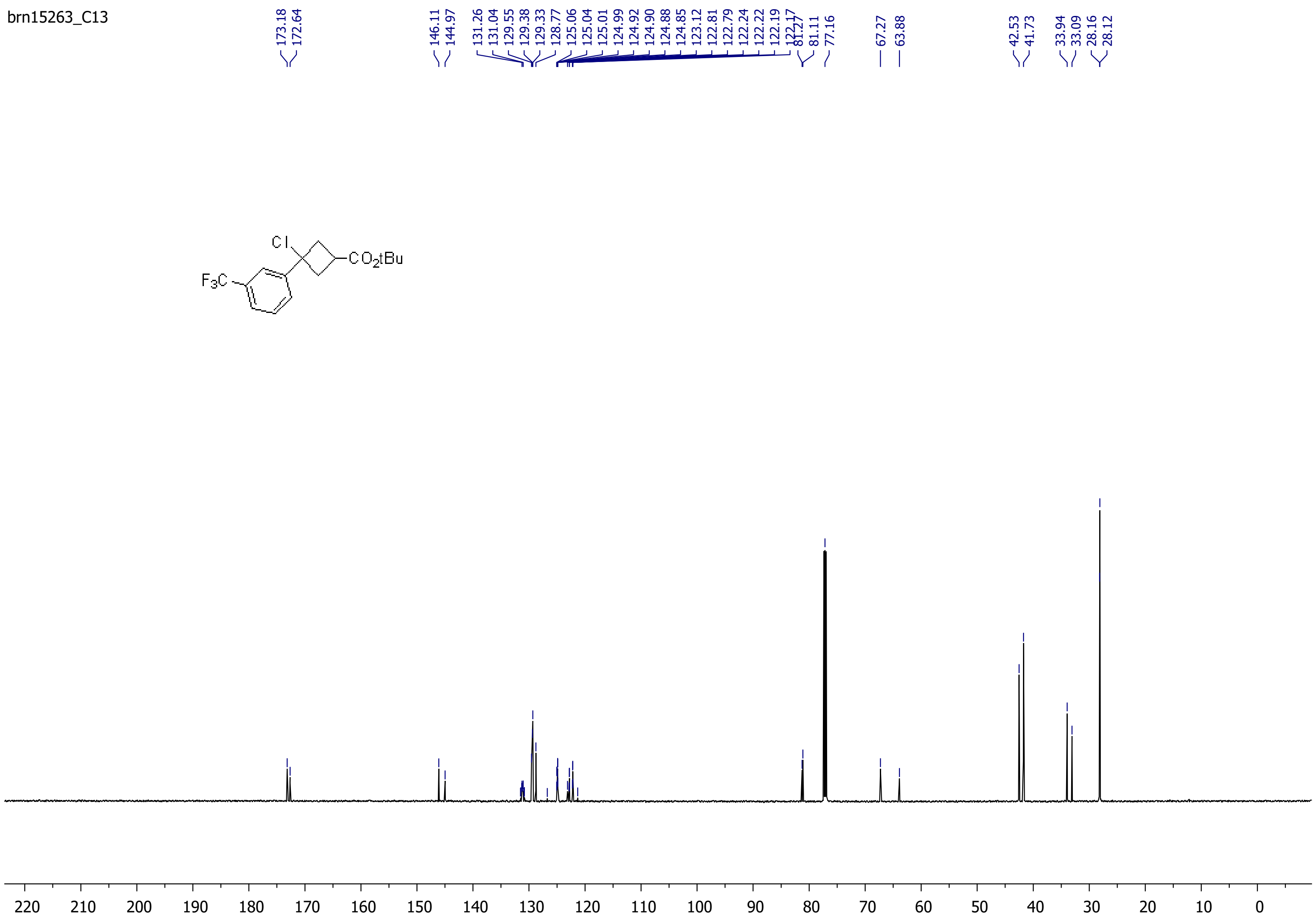

$^{19}F\{^{1}H\}$ NMR (376 MHz, $CDCl_3$)

brn15263_F19
19F-{1H}

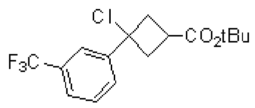


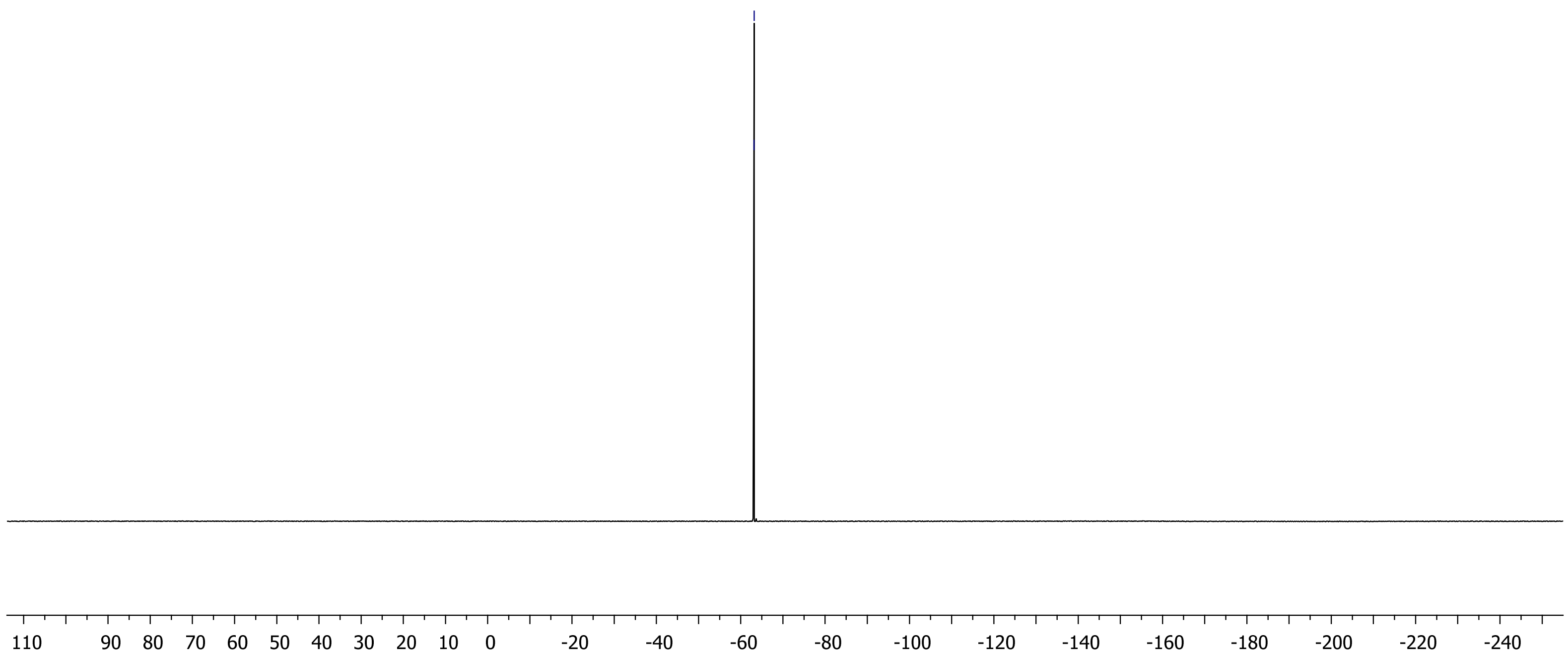

**Tert-butyl 3-chloro-3-(4-(trifluoromethyl)phenyl)cyclobutane-1-carboxylate**

$^{1}$H NMR (500 MHz, $CDCl_3$)

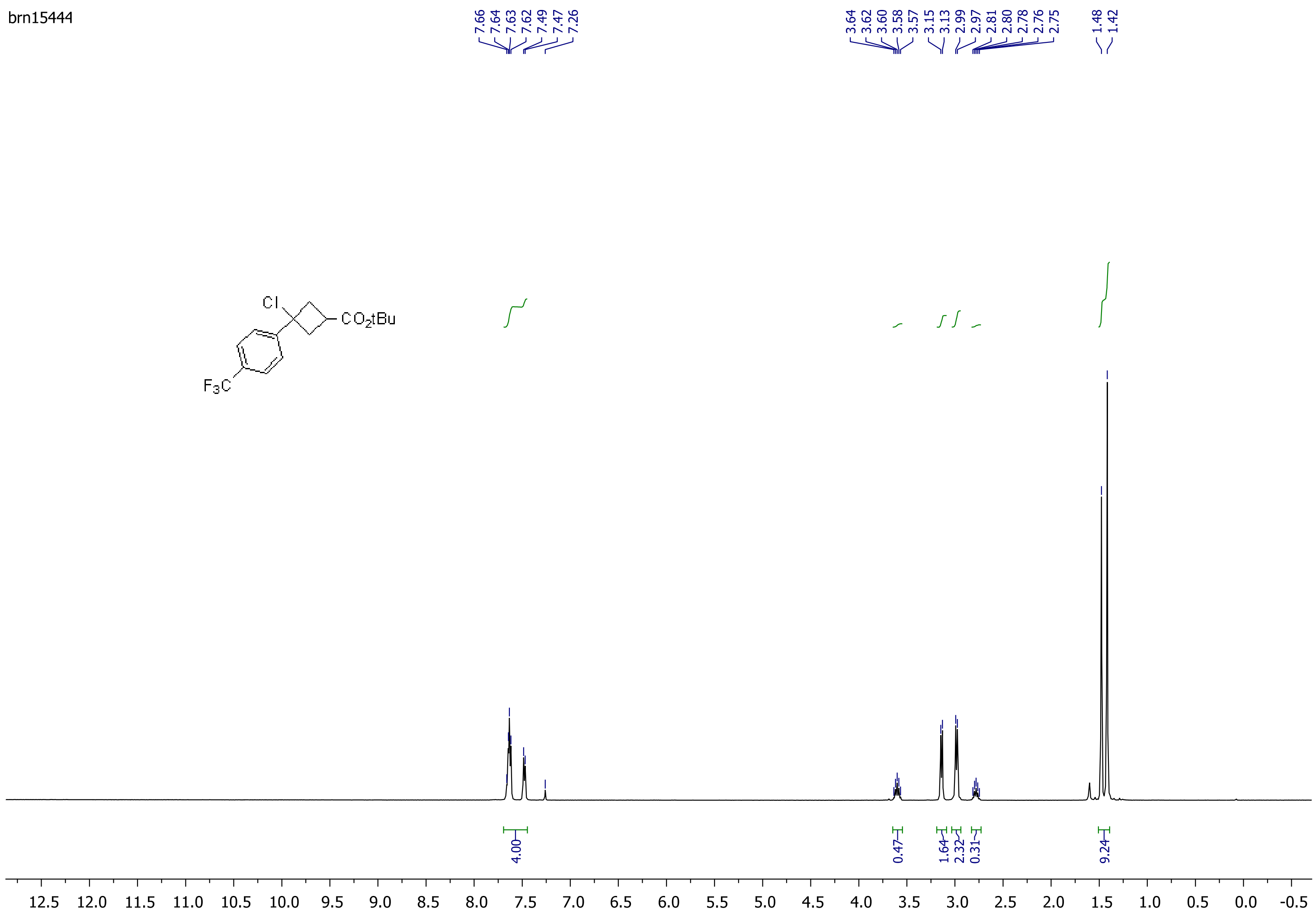

$^{13}C\{^1H\}$ NMR (151 MHz, $CDCl_3$)

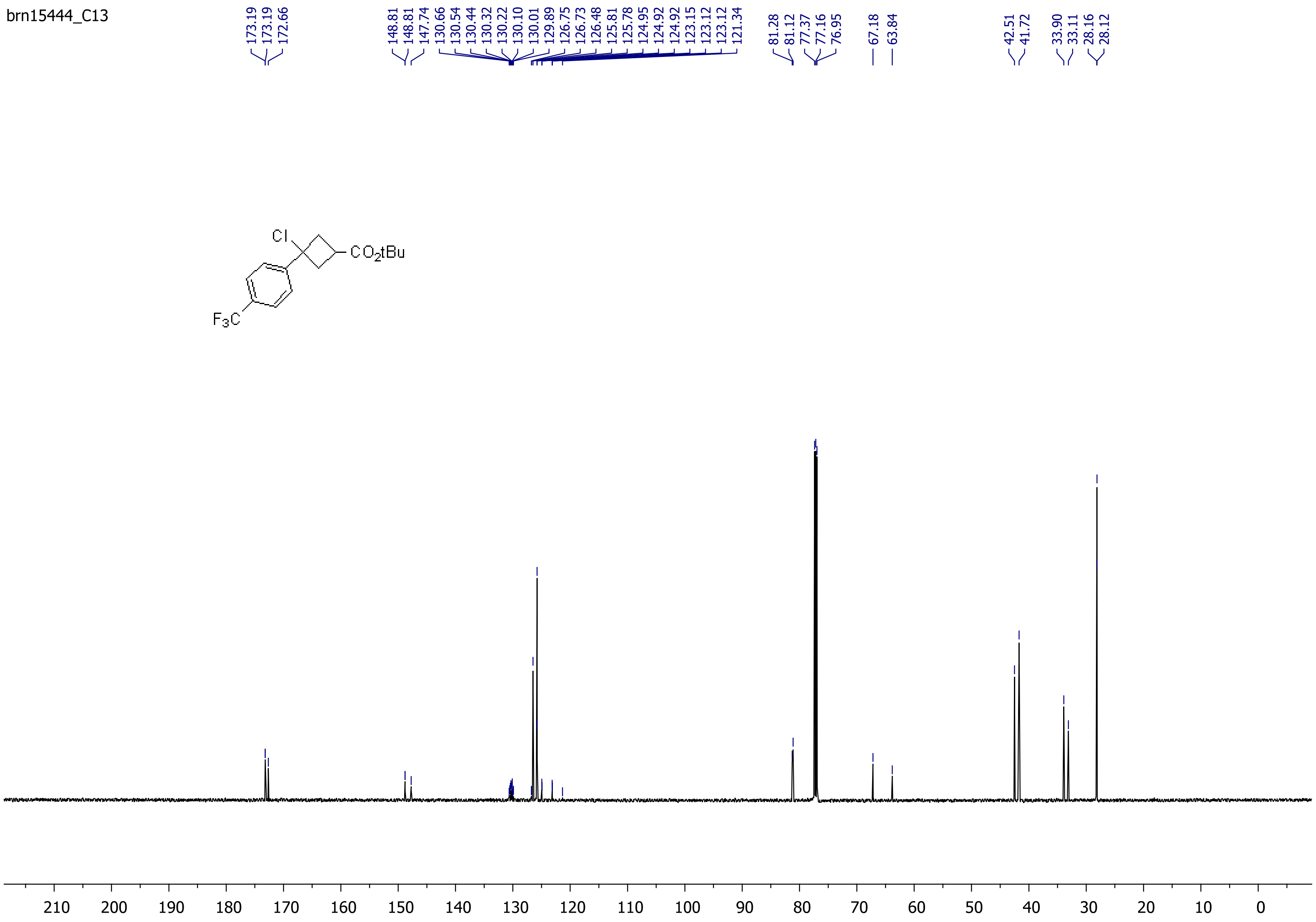

## $^{19}F\{^{1}H\}$ NMR (376 MHz, $CDCl_3$)

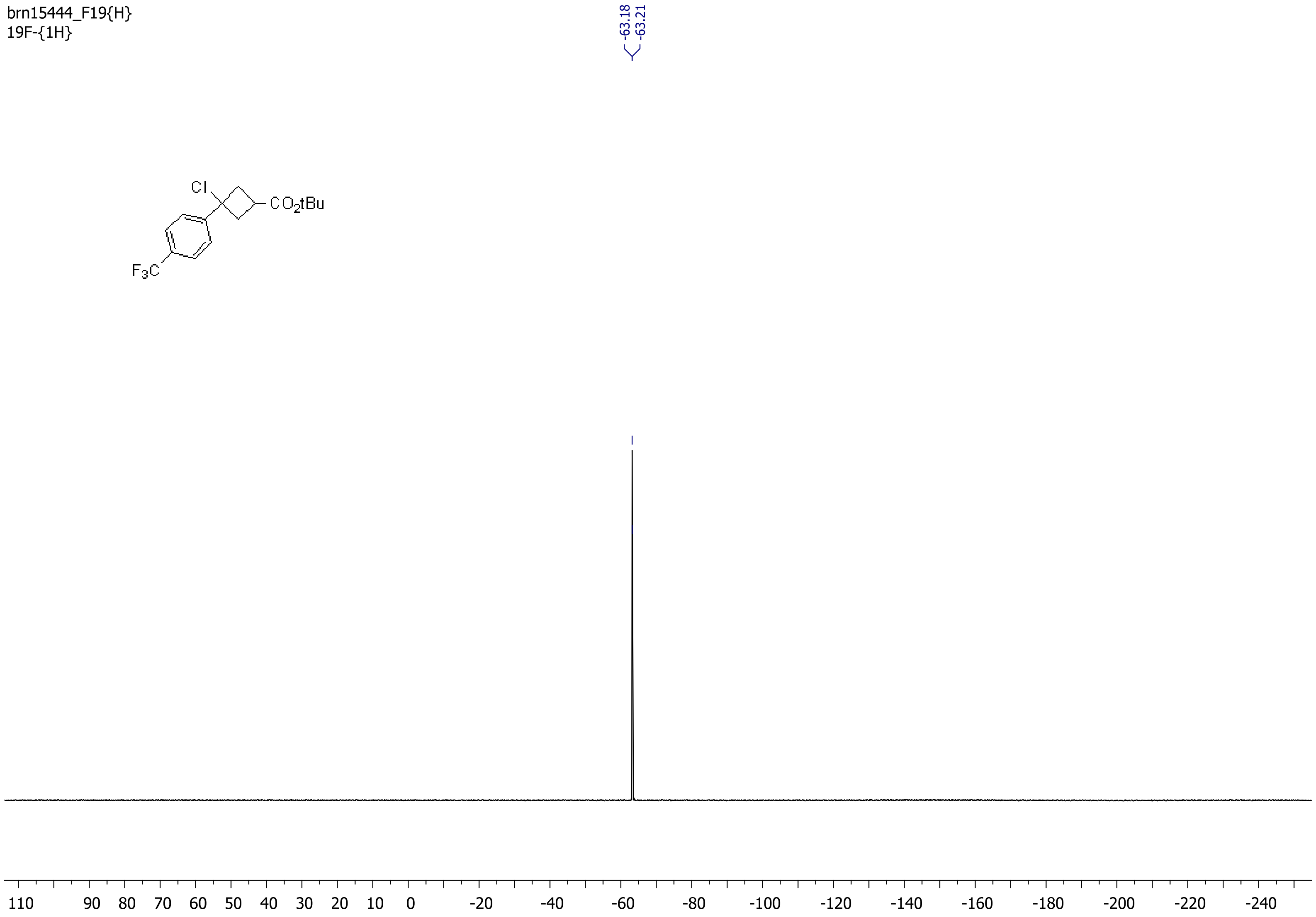

**Compound 5**

$^{1}$H NMR (400 MHz, $CDCl_3$)

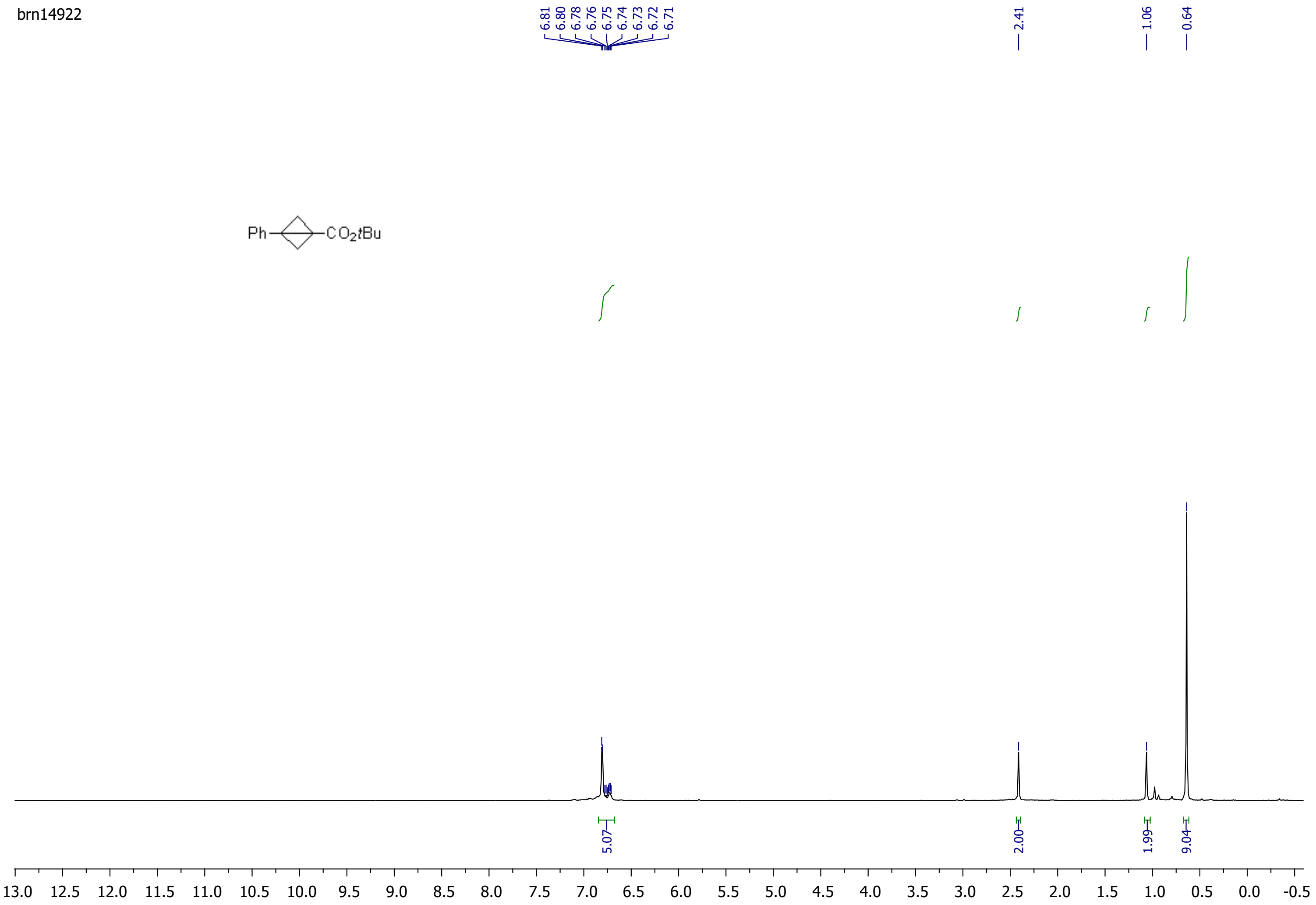

$^{13}C\ \{^{1}H\}$ NMR (101 MHz, $CDCl_3$)

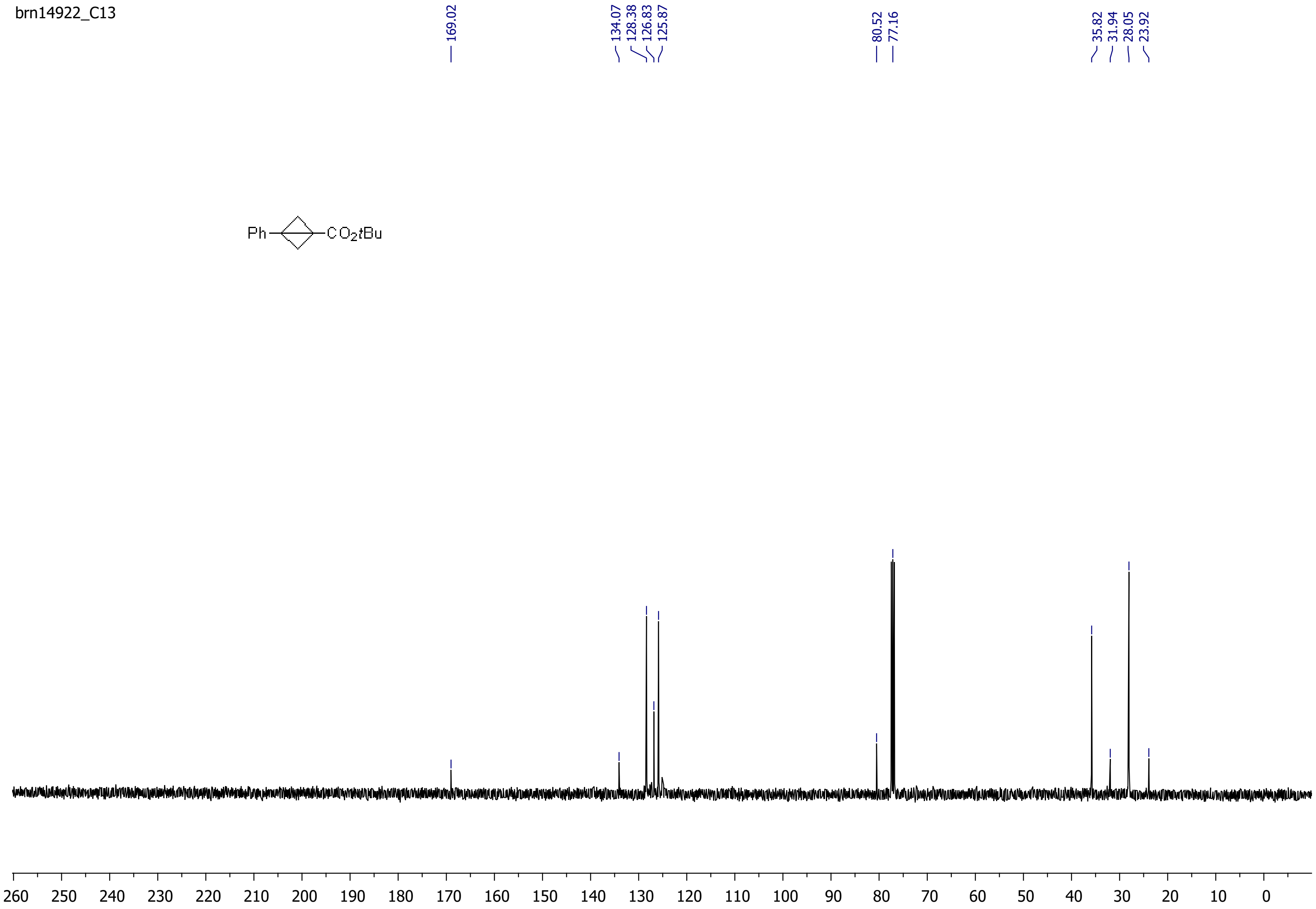

**Compound 13**

$^1$H NMR (400 MHz, $CDCl_3$)

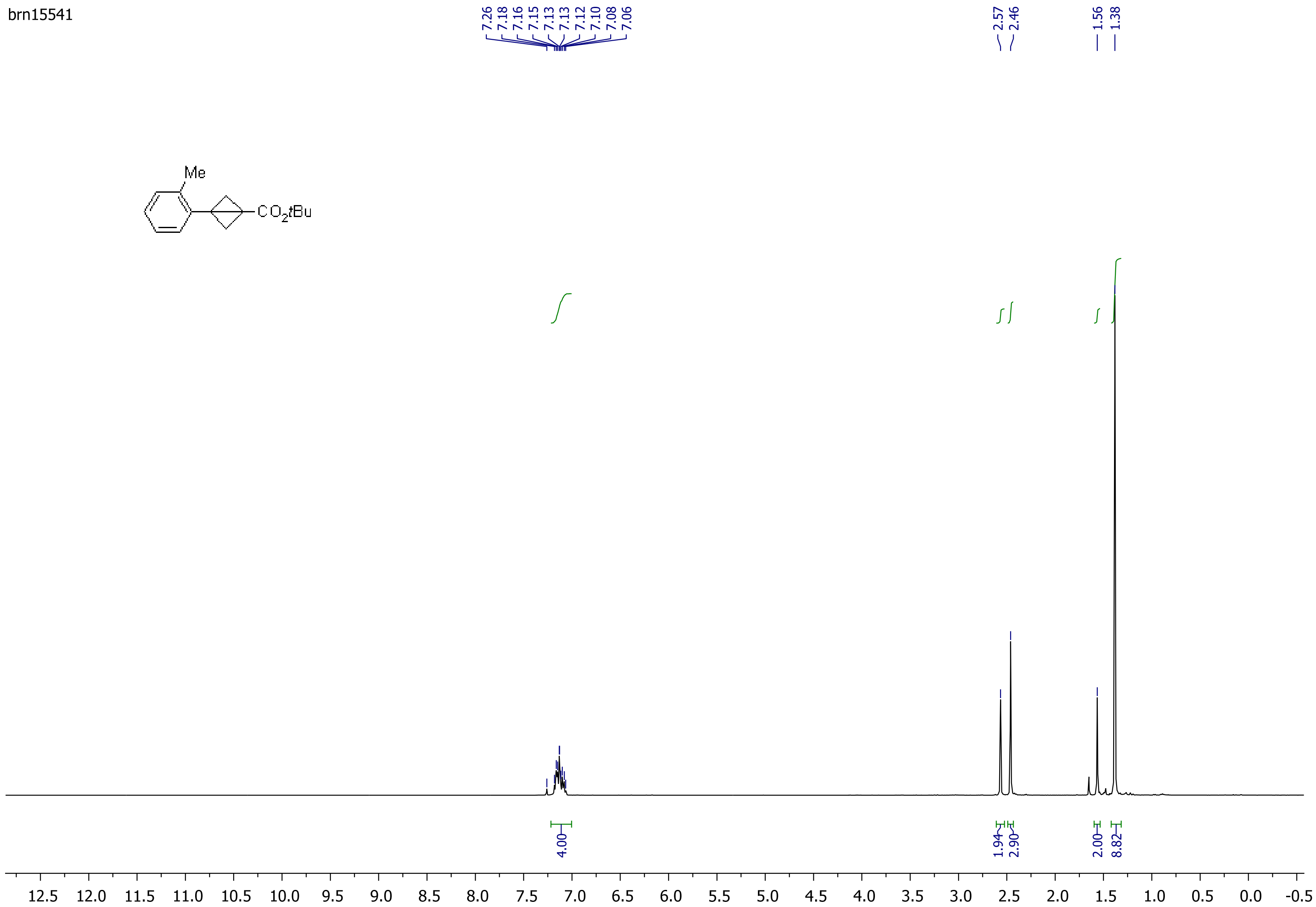

$^{13}C\{^1H\}$ NMR (101 MHz, $CDCl_3$)

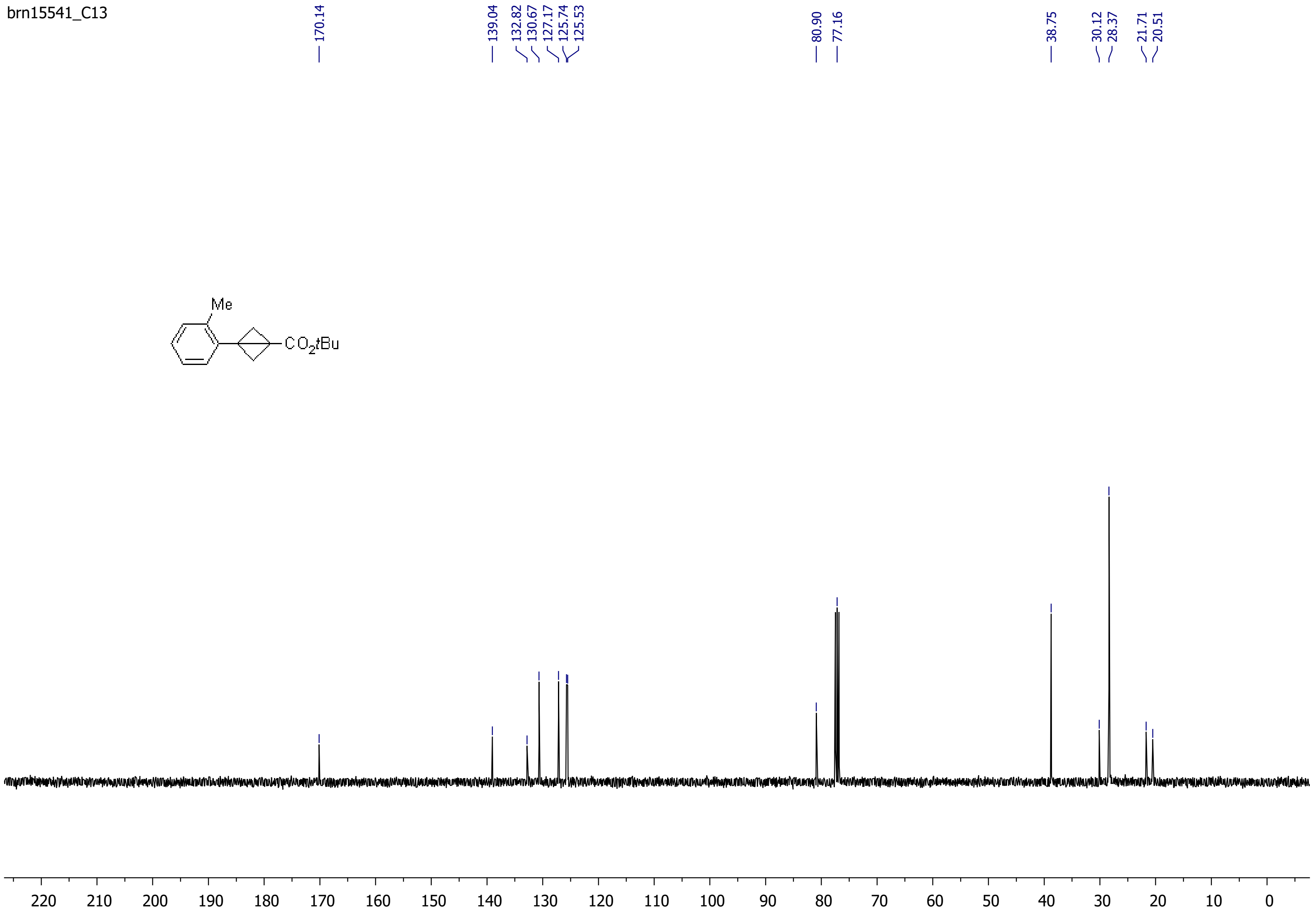

**Compound 14**

$^{1}$H NMR (400 MHz, $CDCl_3$)

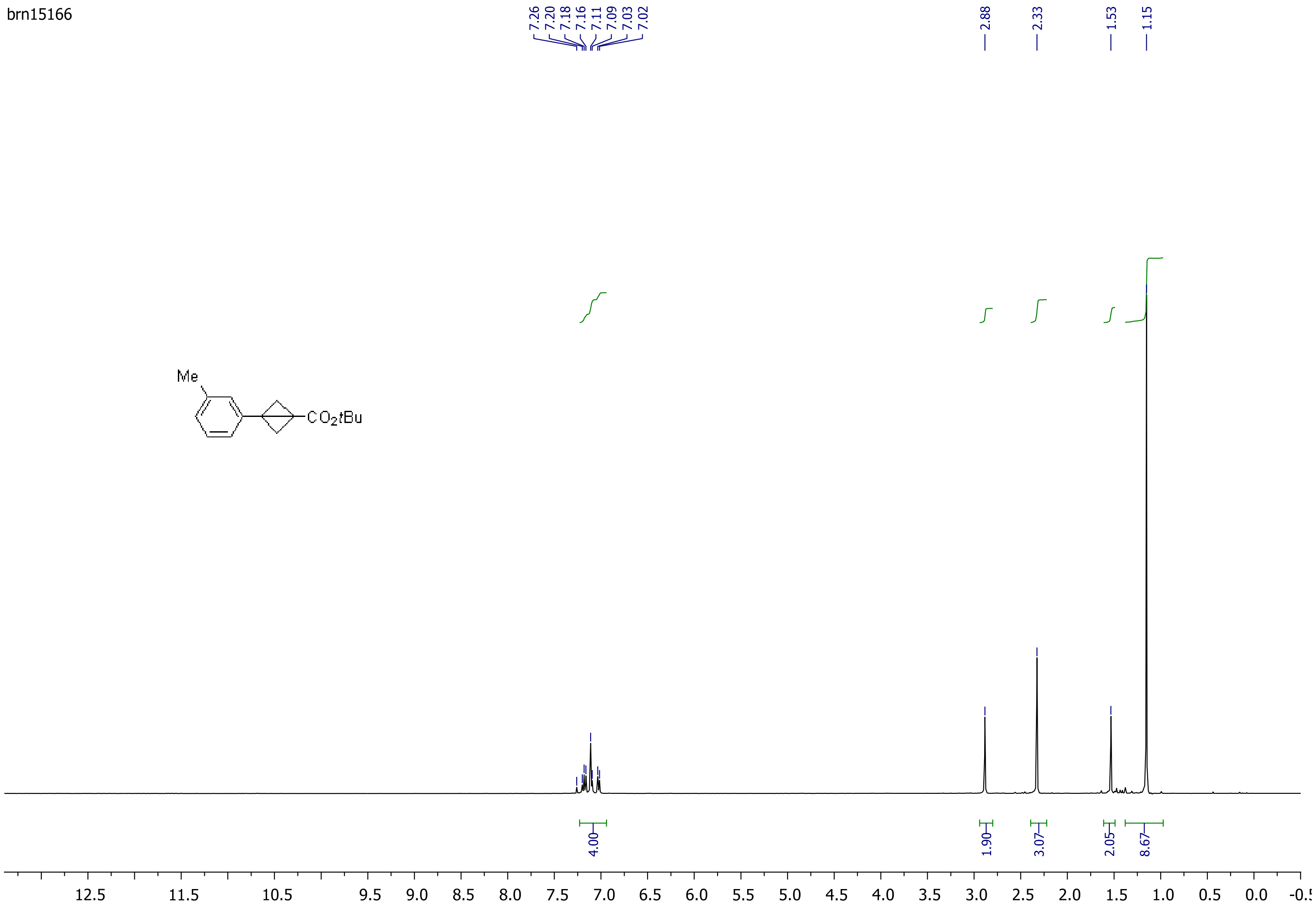

$^{13}C\{^1H\}$ NMR (151 MHz, $CDCl_3$)

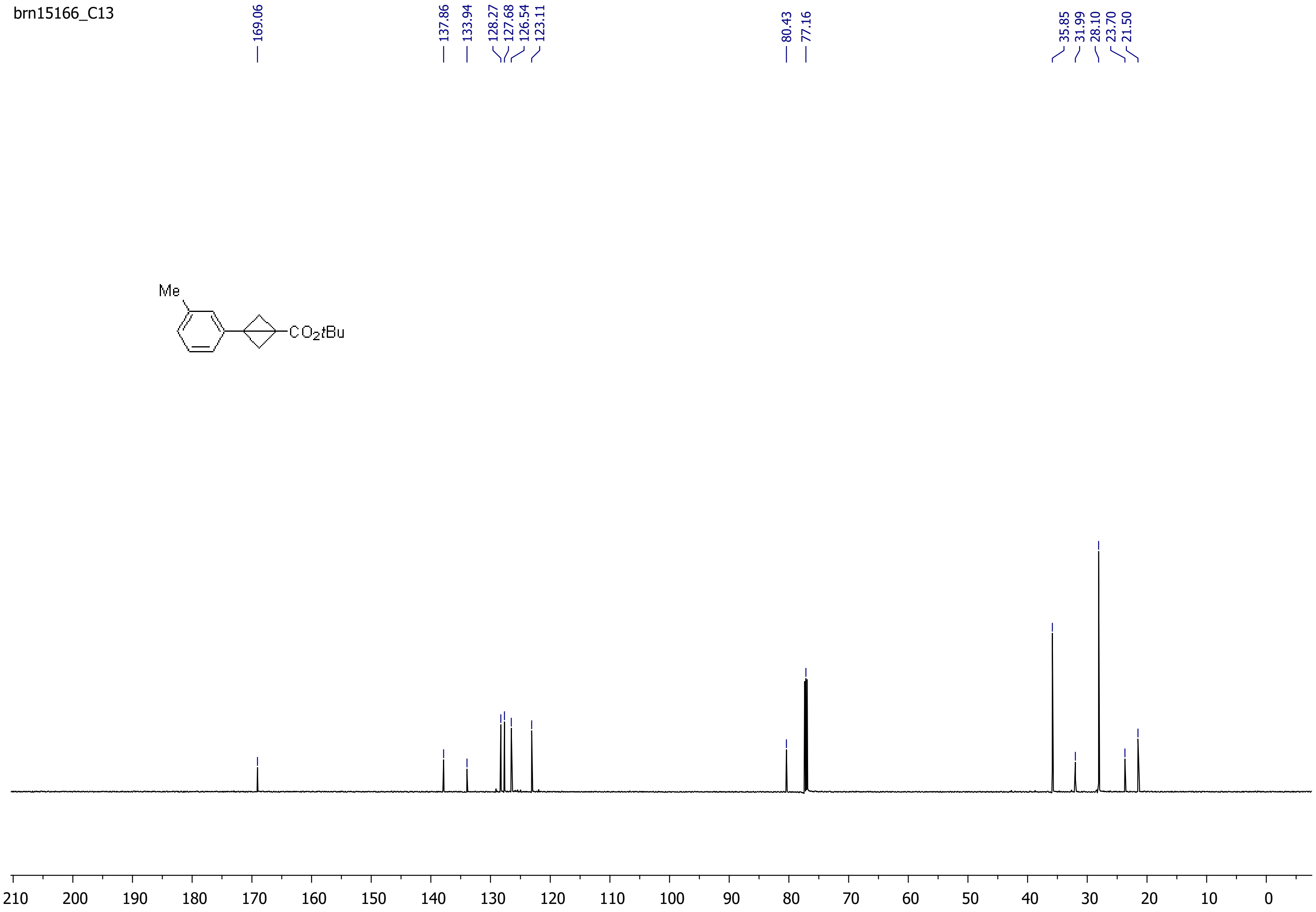

**Compound 15**

$^1H$ NMR (400 MHz, $CDCl_3$)

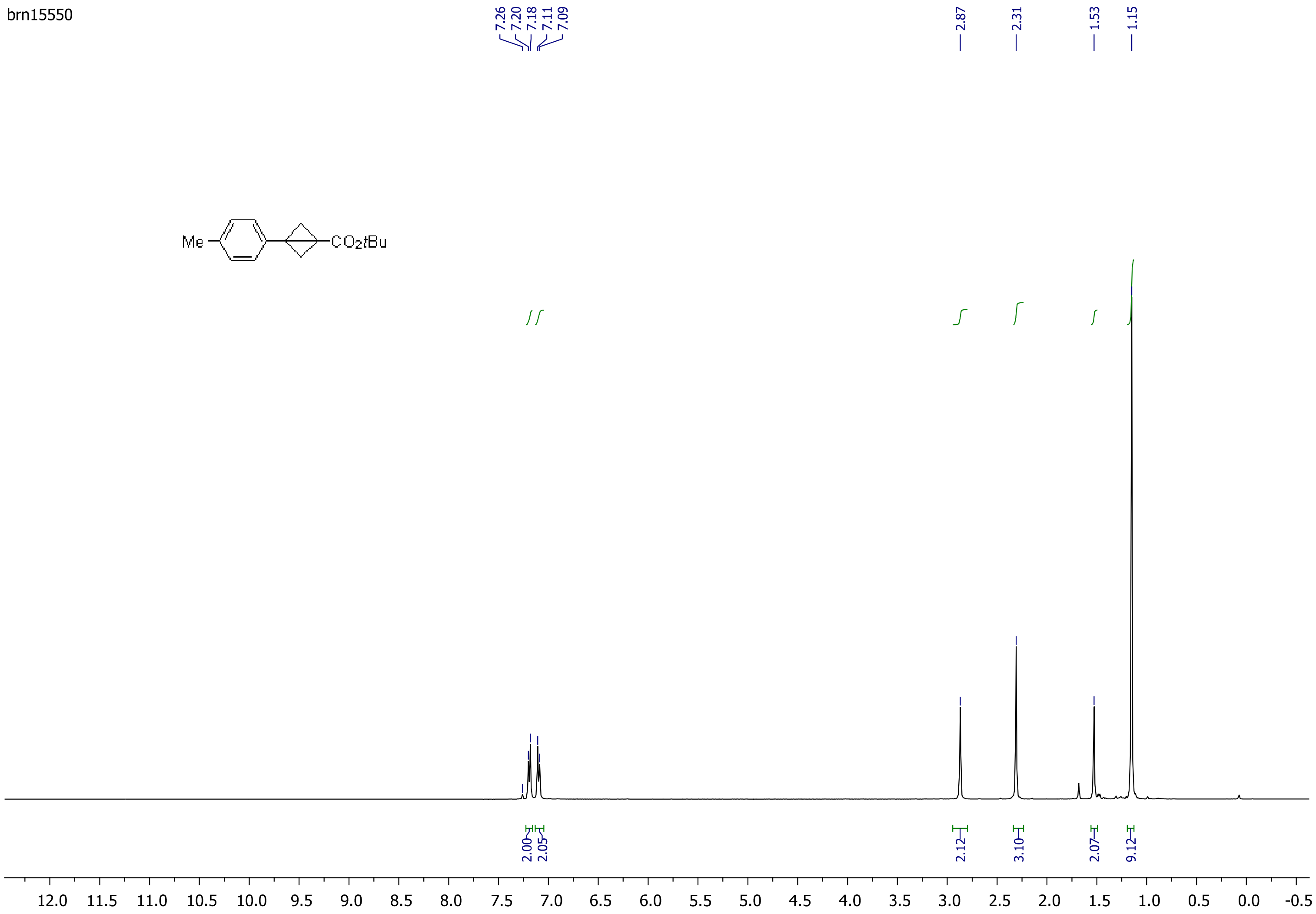

$^{13}C\{^1H\}$ NMR (101 MHz, $CDCl_3$)

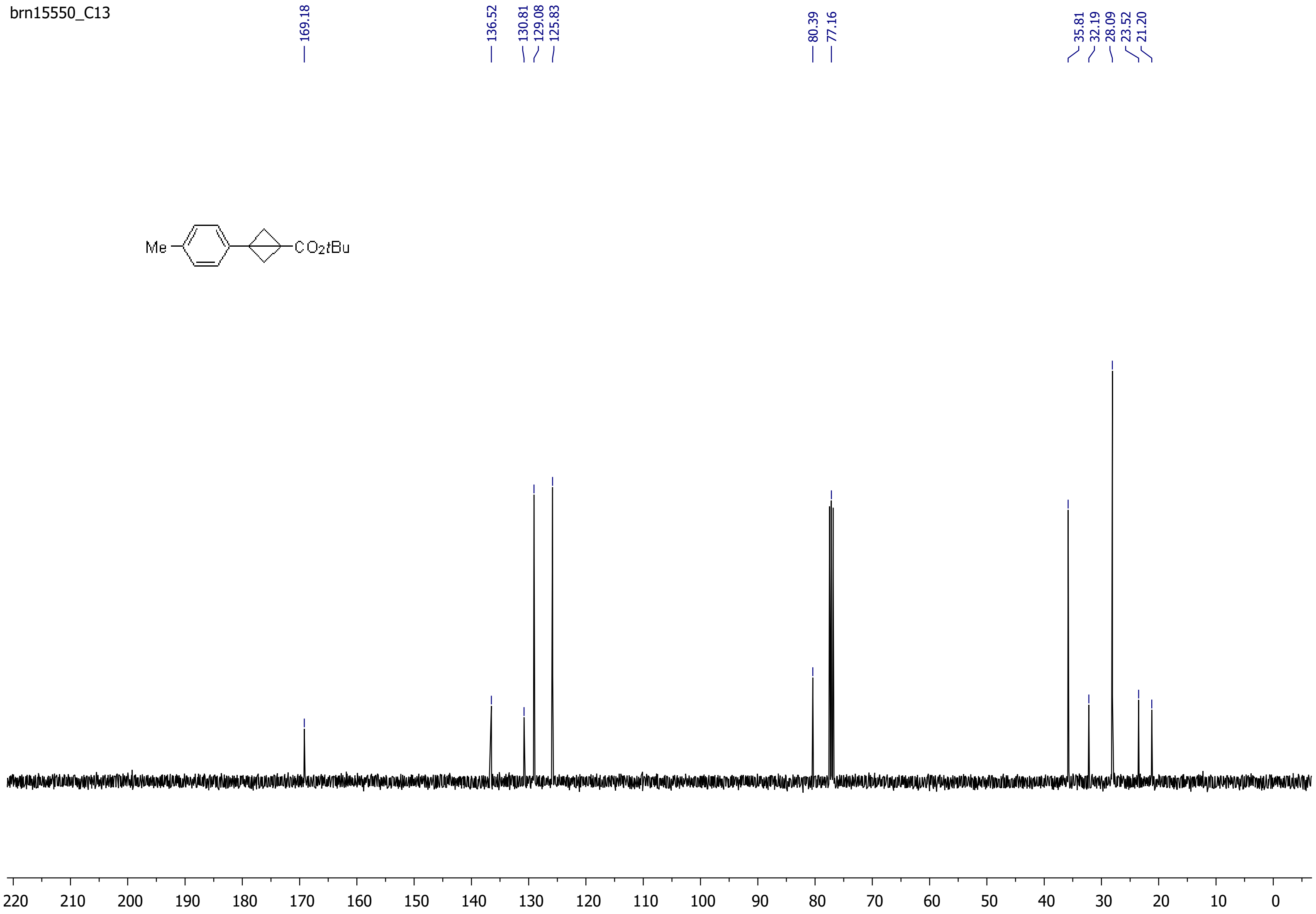

**Compound 16**

$^{1}$H NMR (400 MHz, $CDCl_3$)

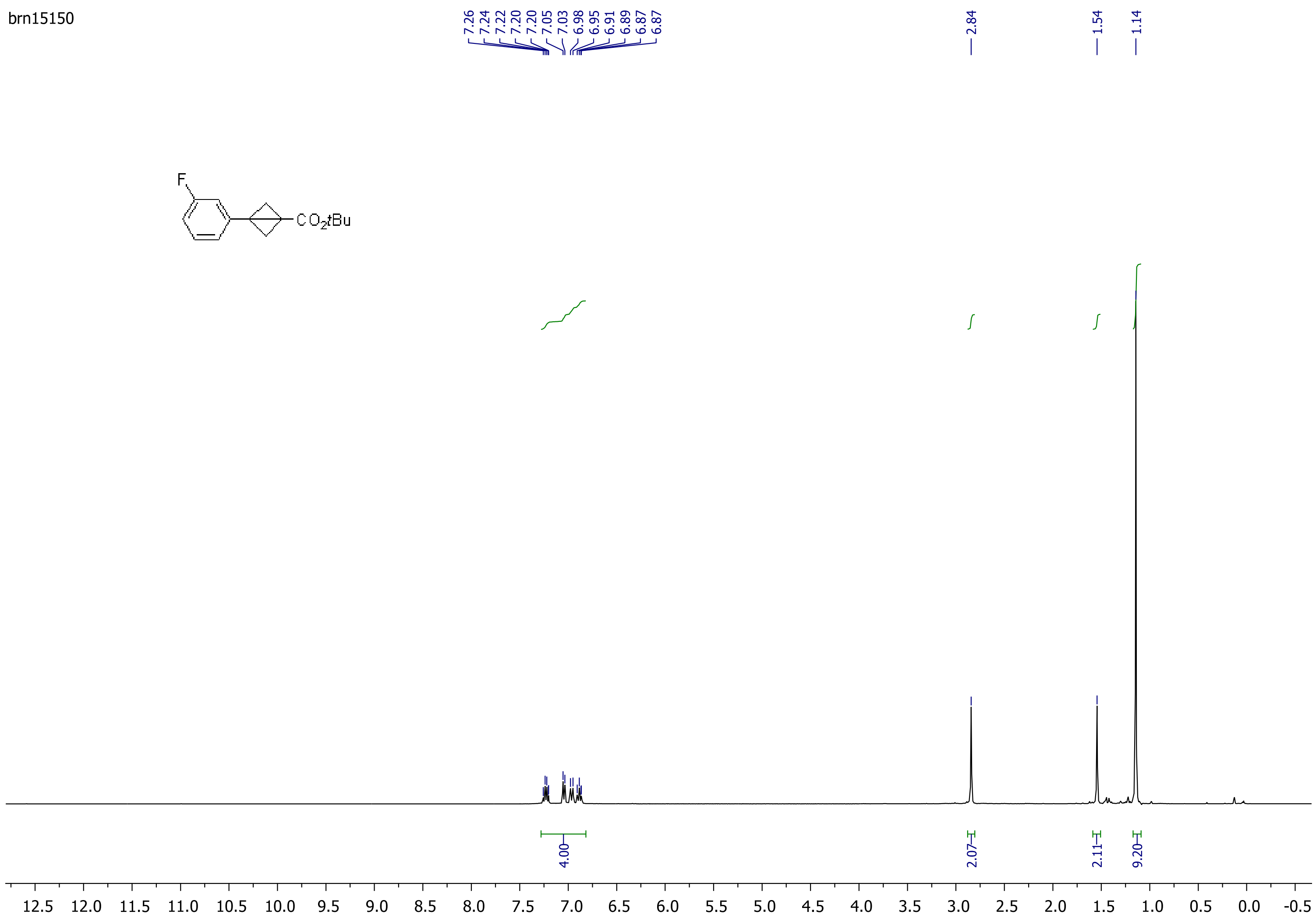

$^{13}C\{^1H\}$ NMR (151 MHz, $CDCl_3$)

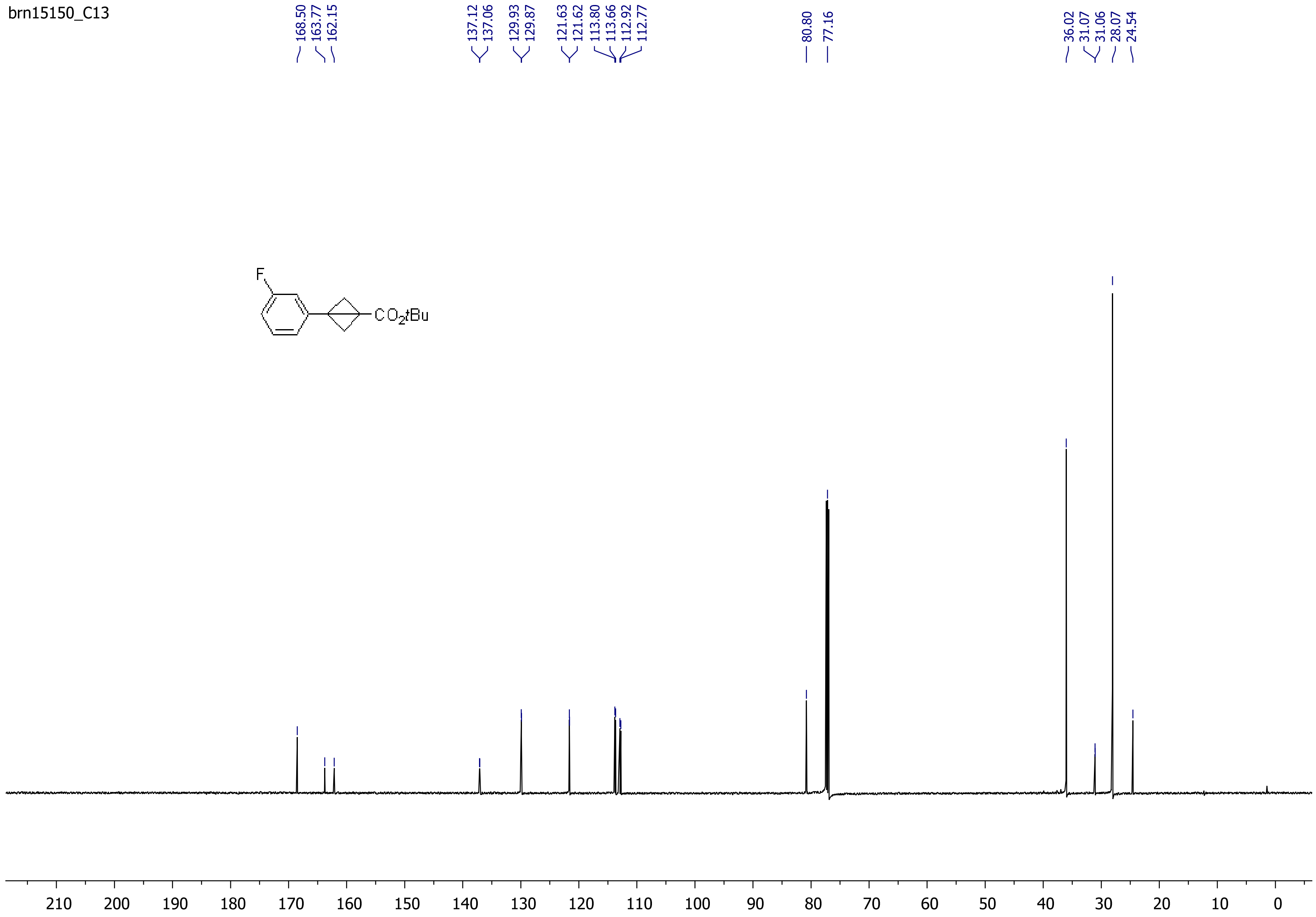

$^{19}F\{^1H\}$ NMR (376 MHz, $CDCl_3$)

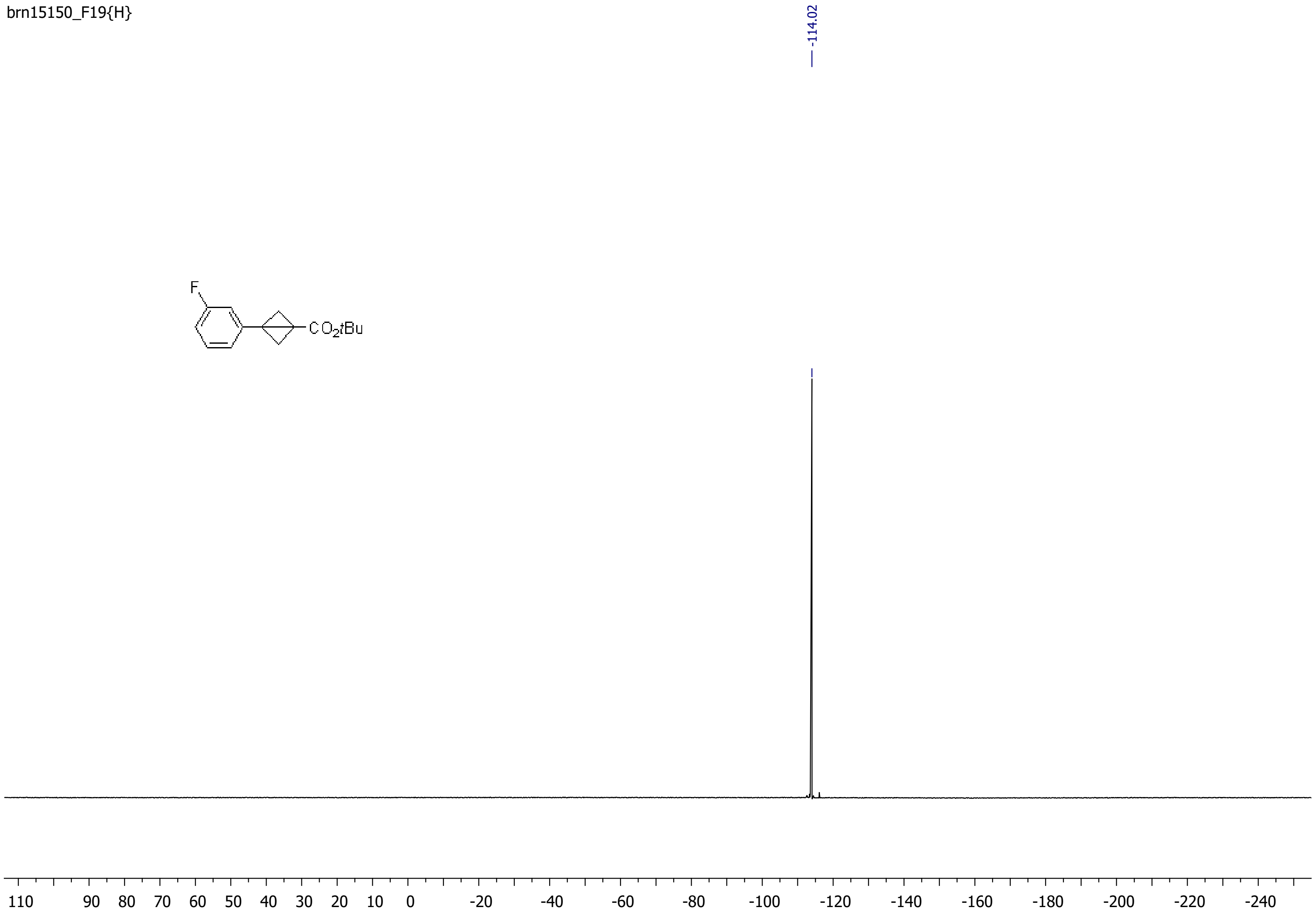

**Compound 17**

$^{1}H$ NMR (400 MHz, $CDCl_3$)

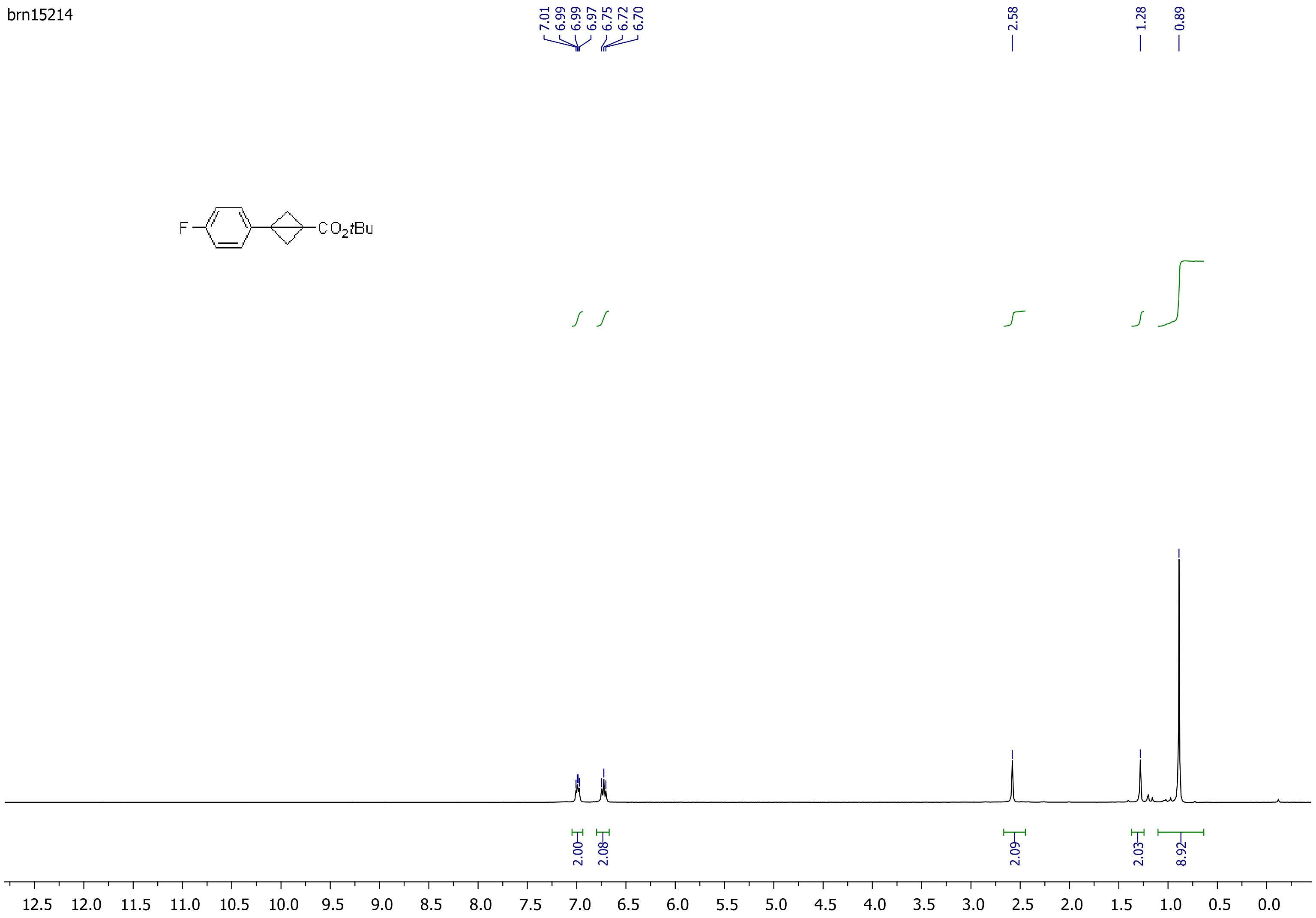

$^{13}C\{^{1}H\}$ NMR (126 MHz, $CDCl_3$)

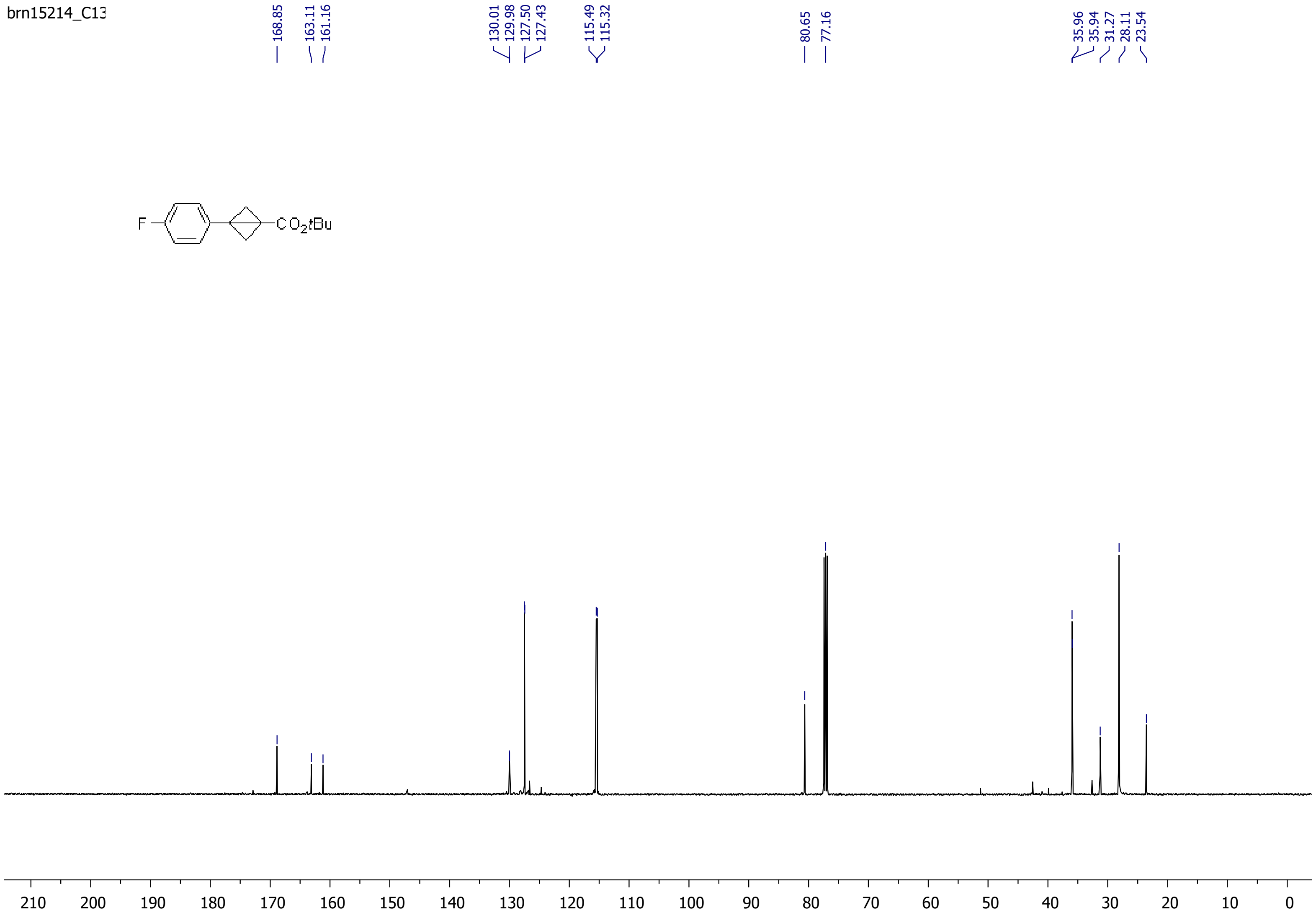

$^{19}F\{^{1}H\}$ NMR (376 MHz, $CDCl_3$)

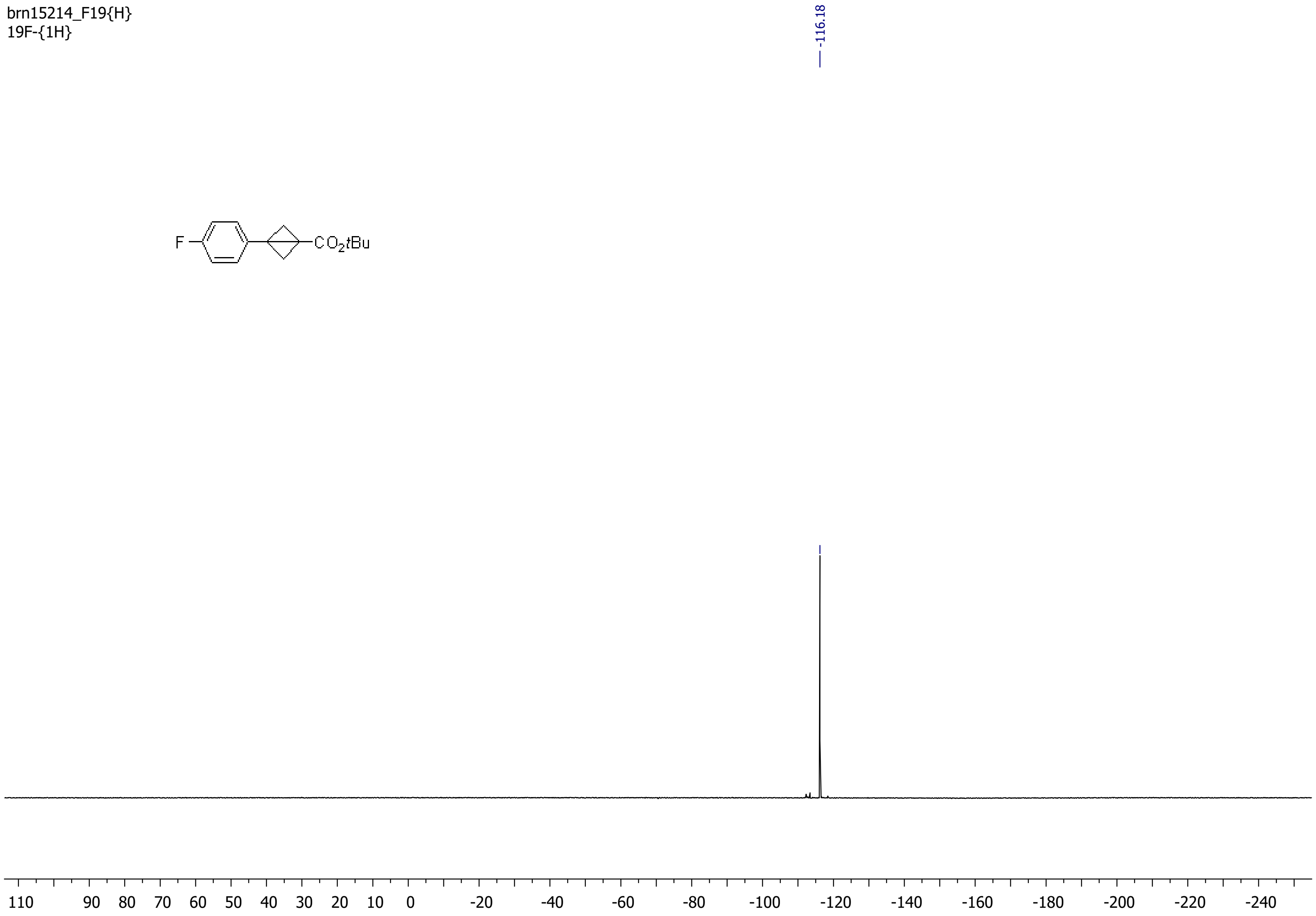

**Compound 18**

$^1$H NMR (400 MHz, $CDCl_3$)

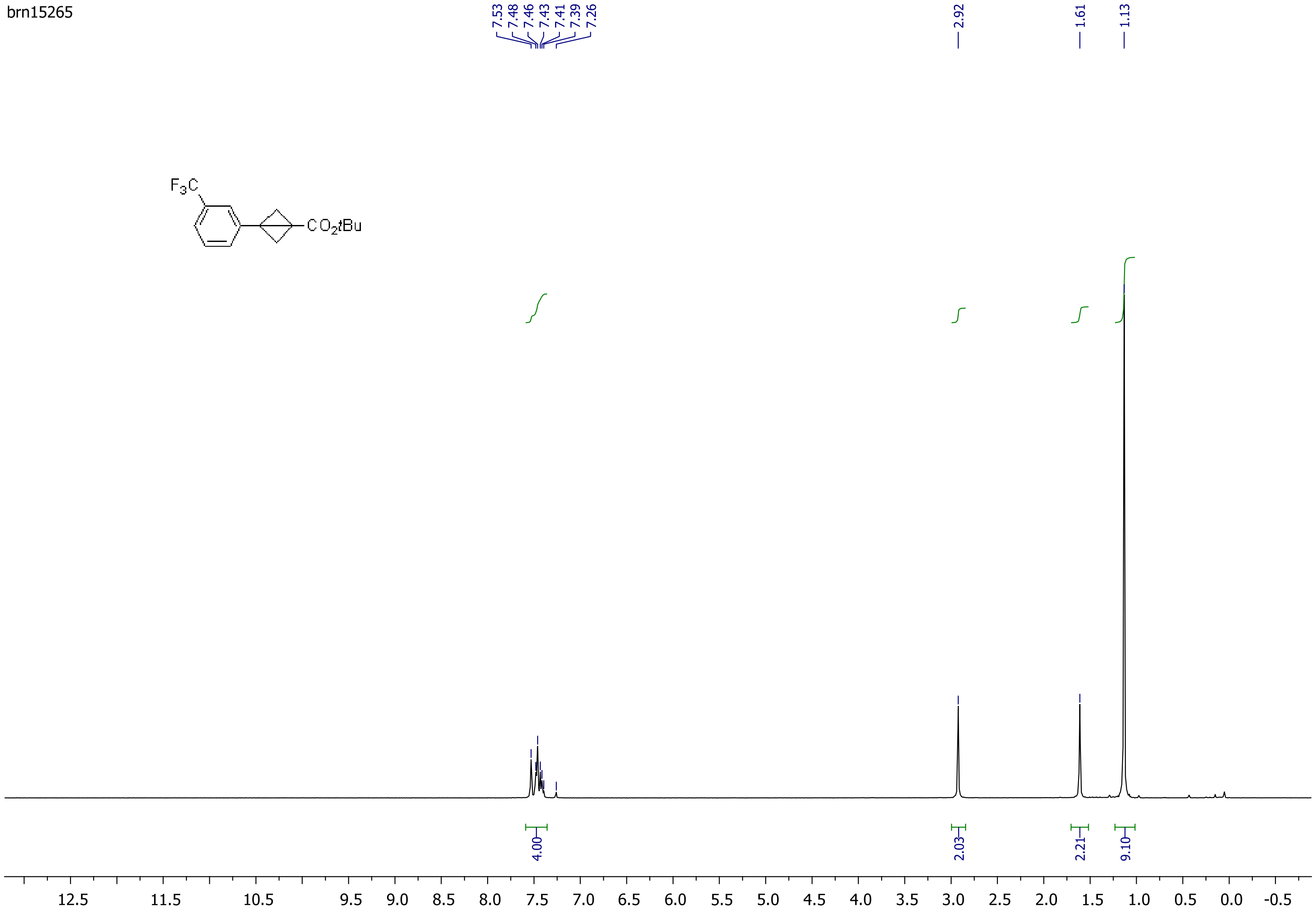

$^{13}C\{^1H\}$ NMR (126 MHz, $CDCl_3$)

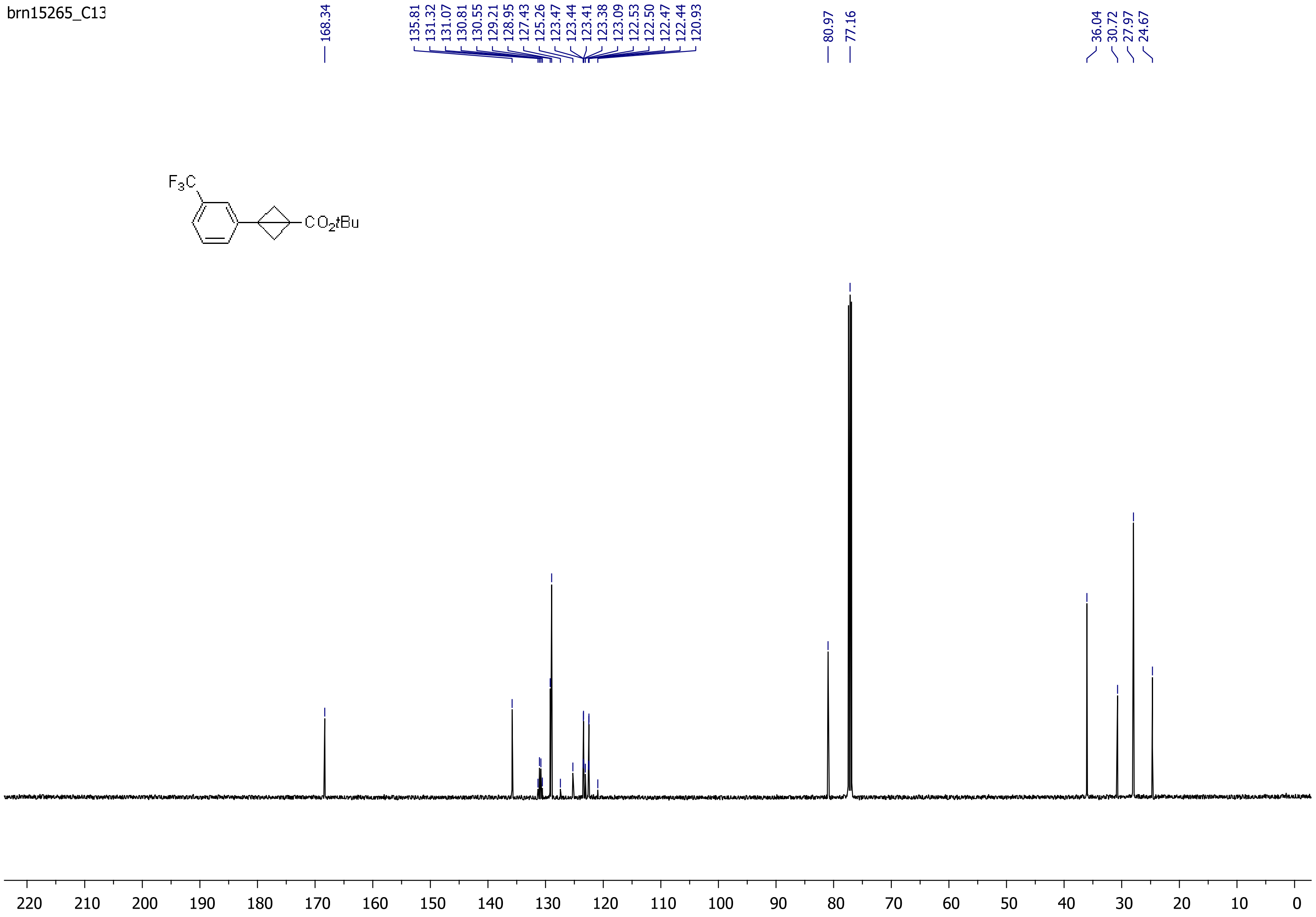

$^{19}F\{^1H\}$ NMR (376 MHz, $CDCl_3$)

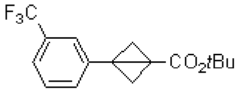


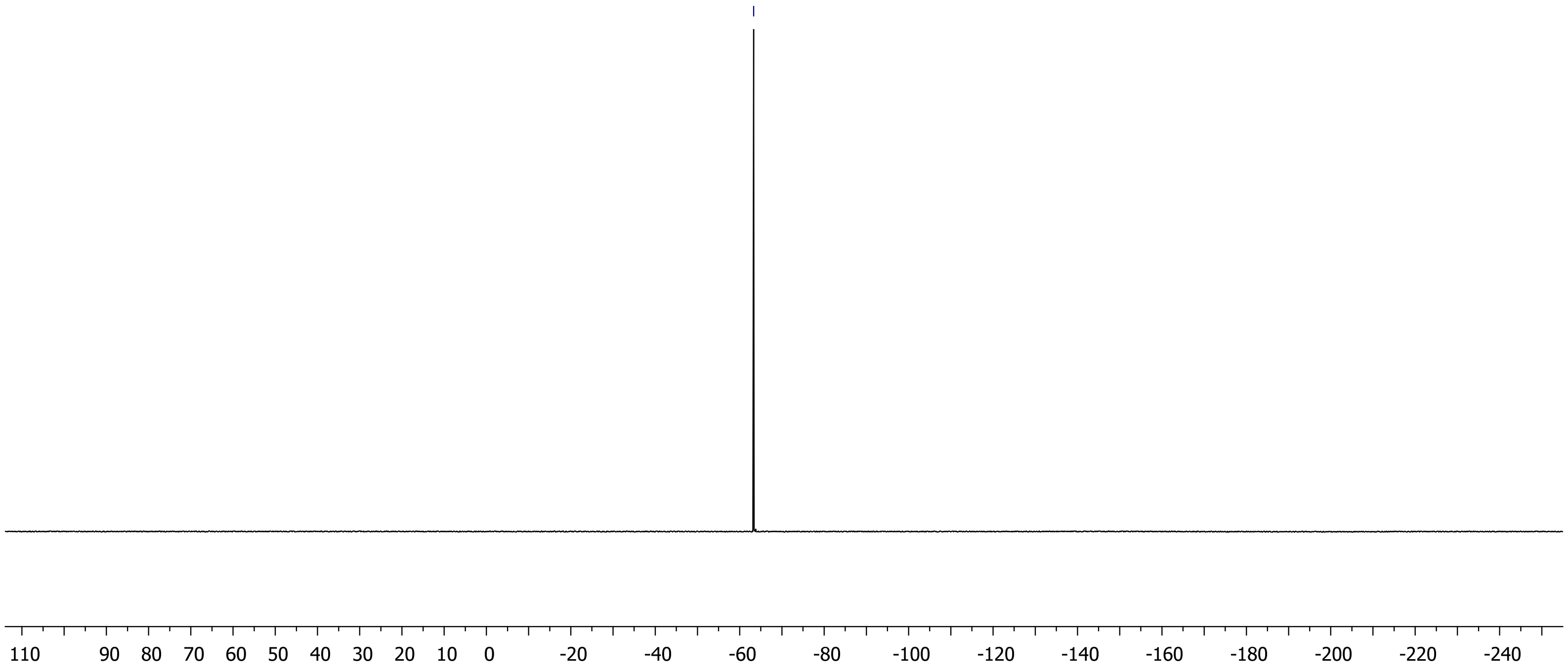

**Compound 19**

$^{1}$H NMR (500 MHz, $CDCl_3$)

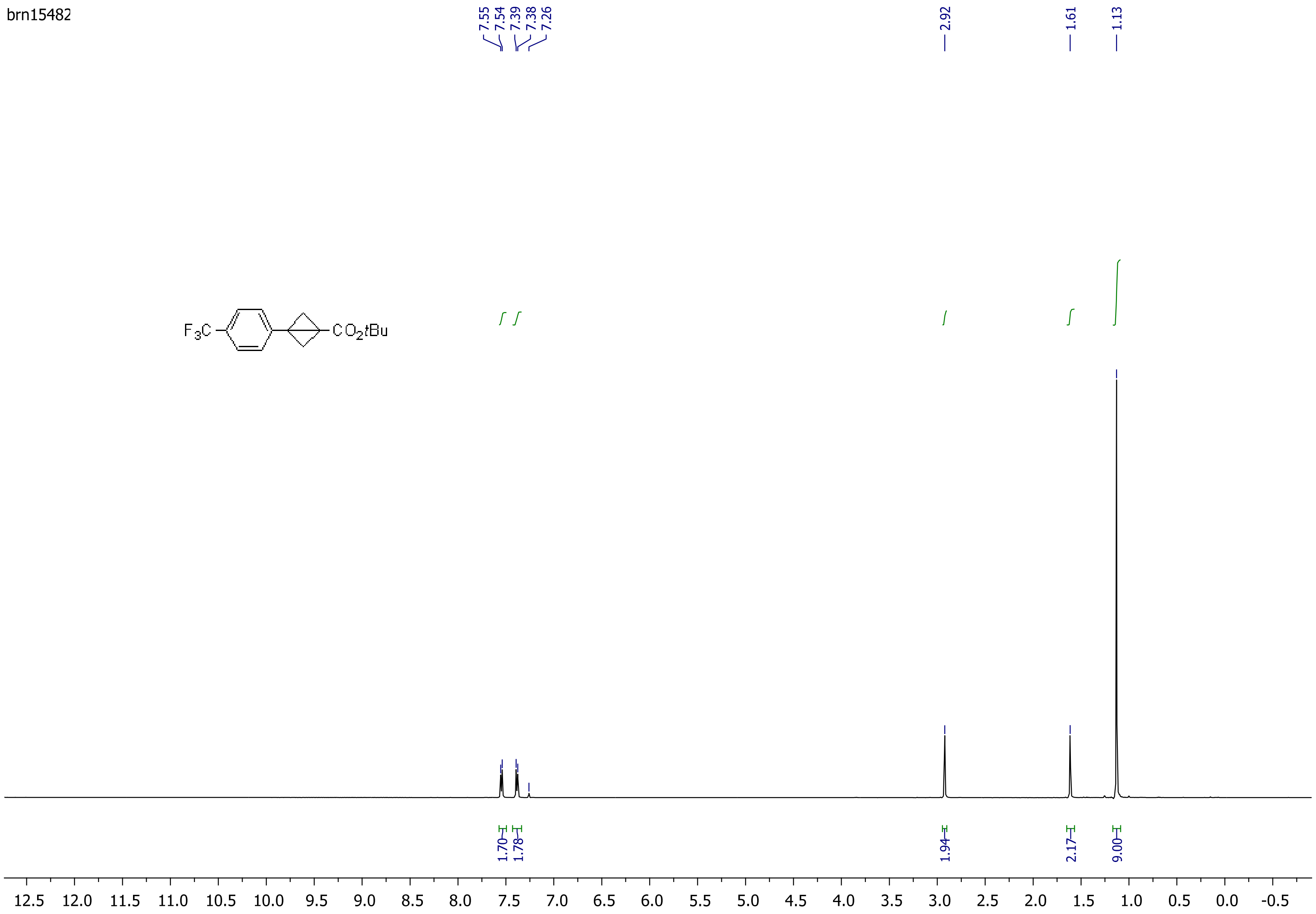

$^{13}C\{^1H\}$ NMR (151 MHz, $CDCl_3$)

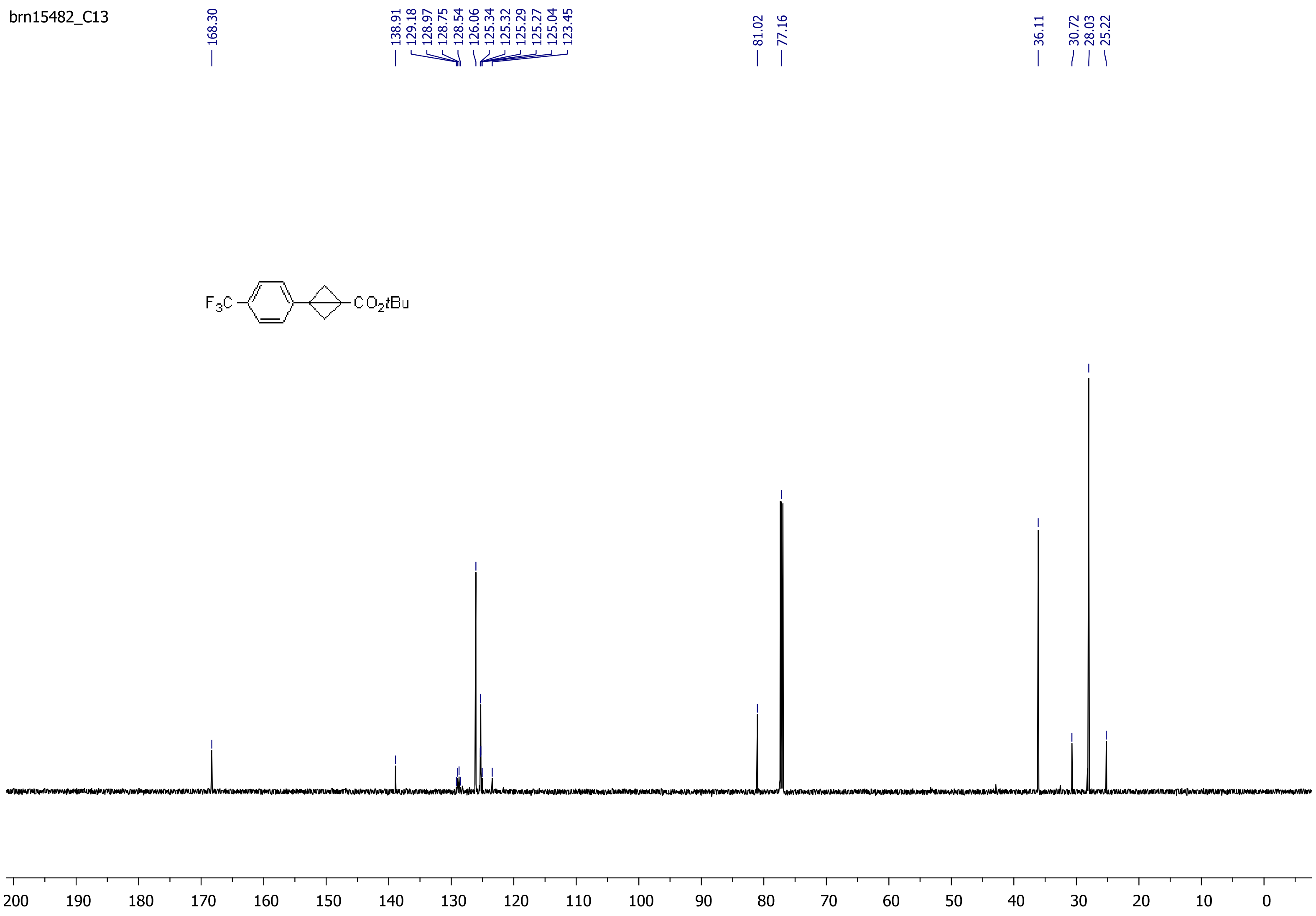

$^{19}F\{^{1}H\}$ NMR (376 MHz, $CDCl_3$)

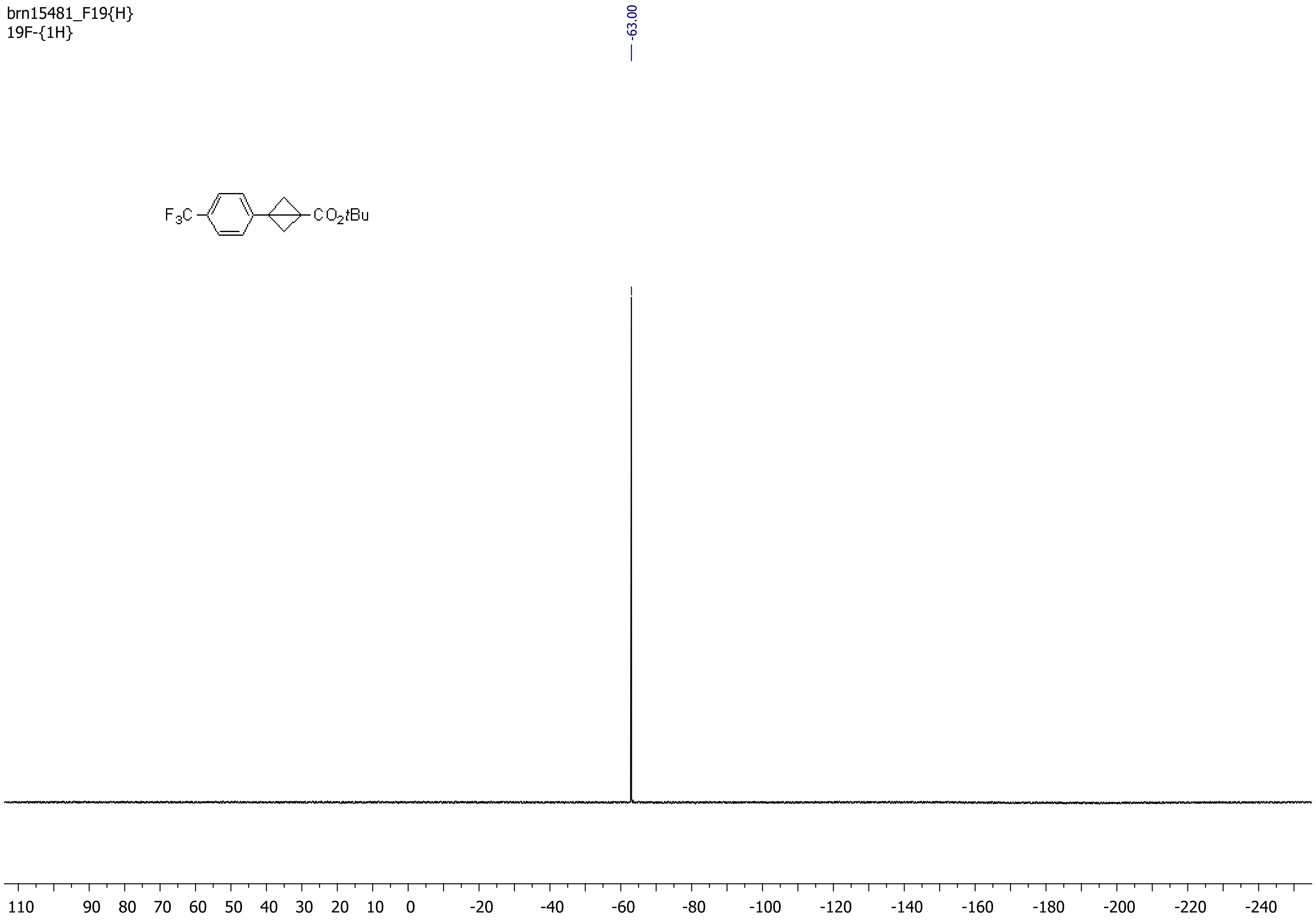

**Compound 8**

$^{1}H$ NMR (500 MHz, $CDCl_3$)

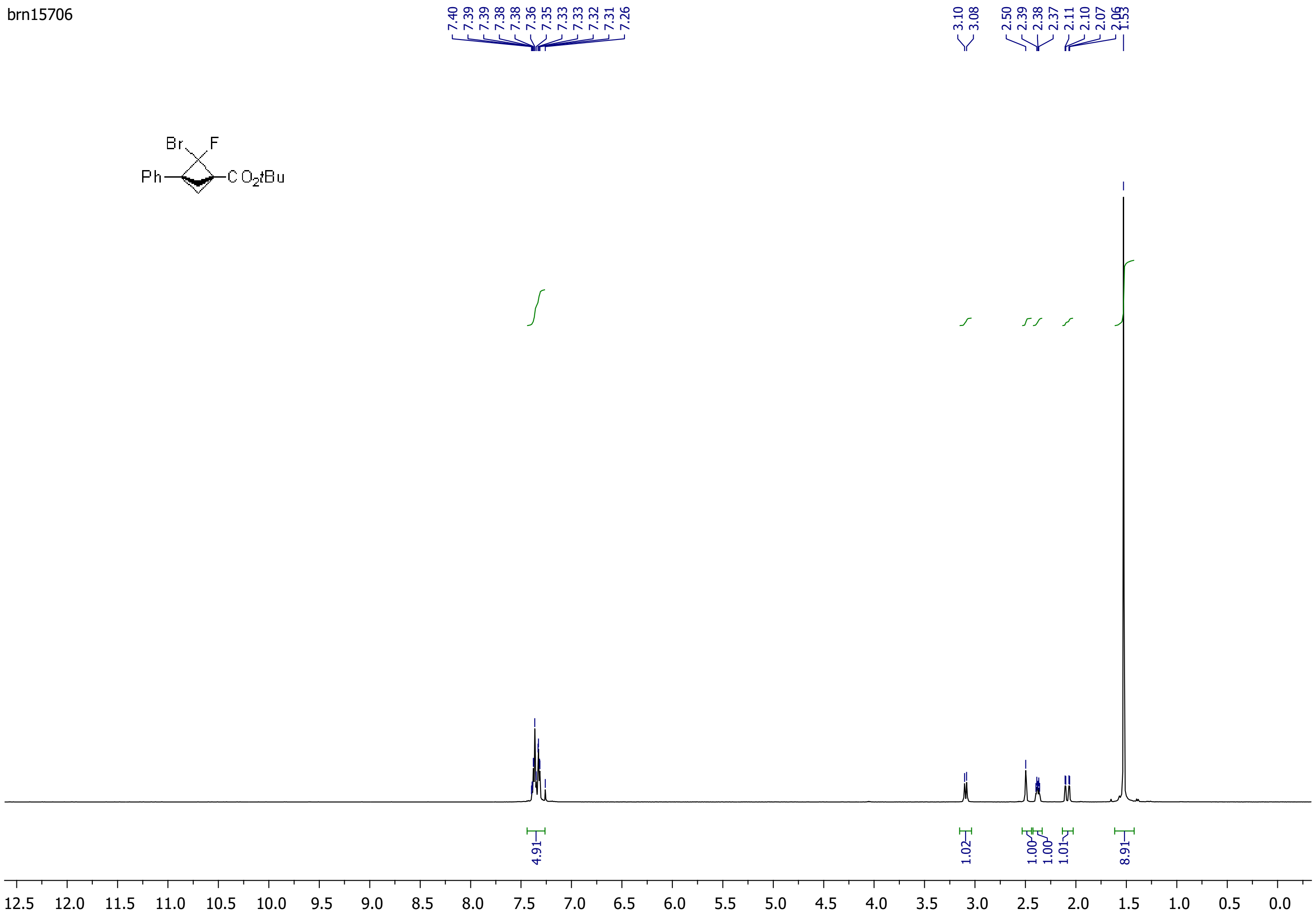

## $^{13}C\{^{1}H\}$ NMR (126 MHz, $CDCl_3$)

brn15706_C13
13C (1H-decoupled)

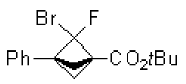


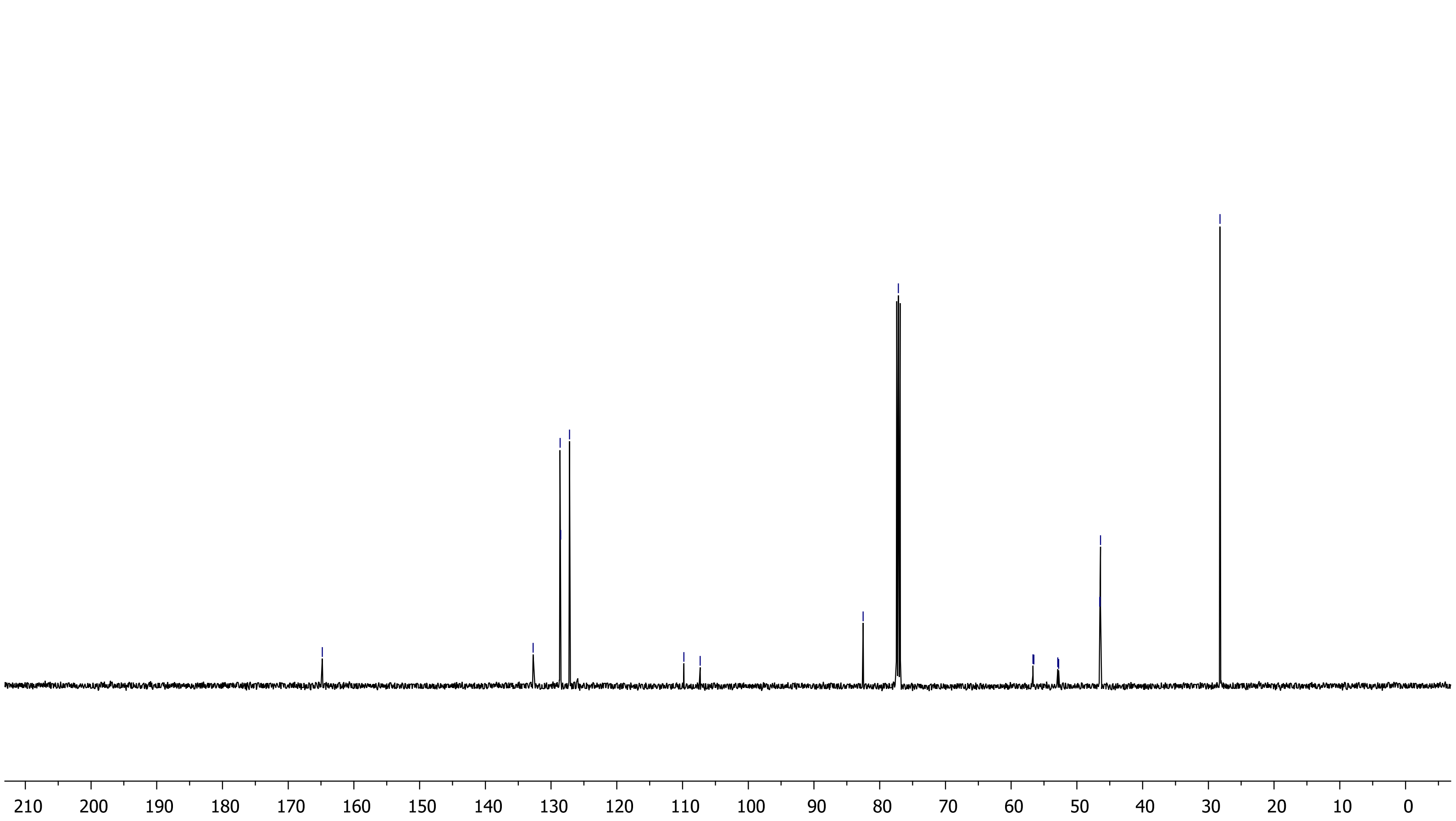

$^{19}F\{^1H\}$ NMR (376 MHz, $CDCl_3$)

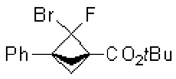


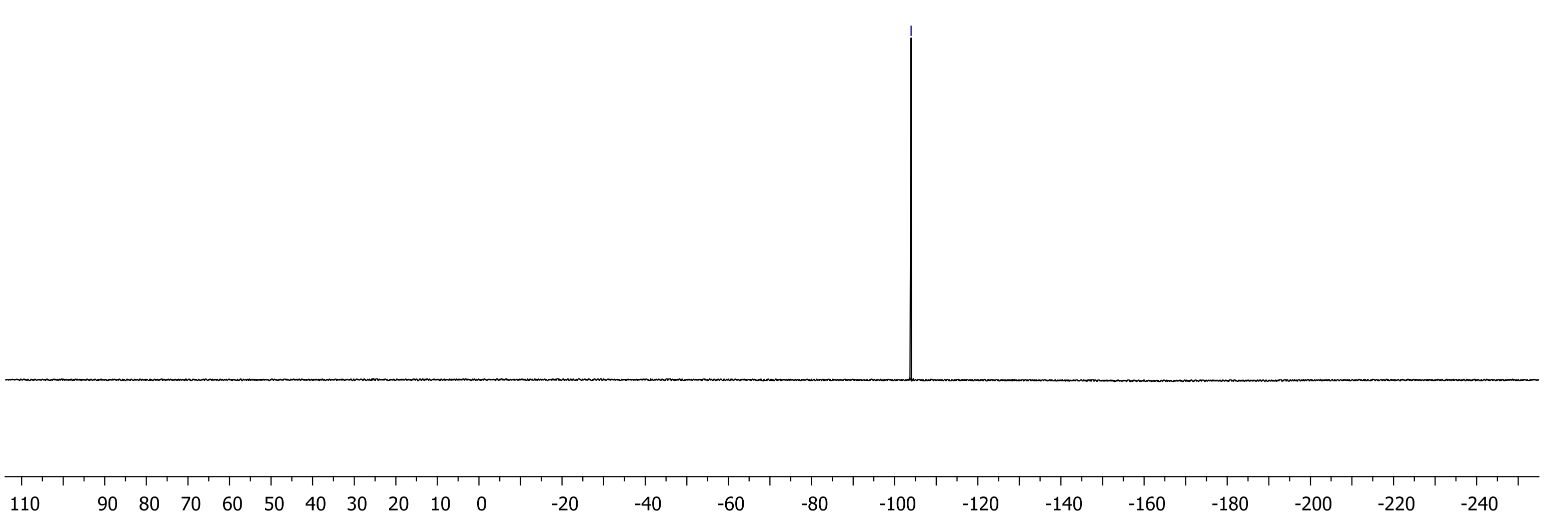

**Compound 13a**

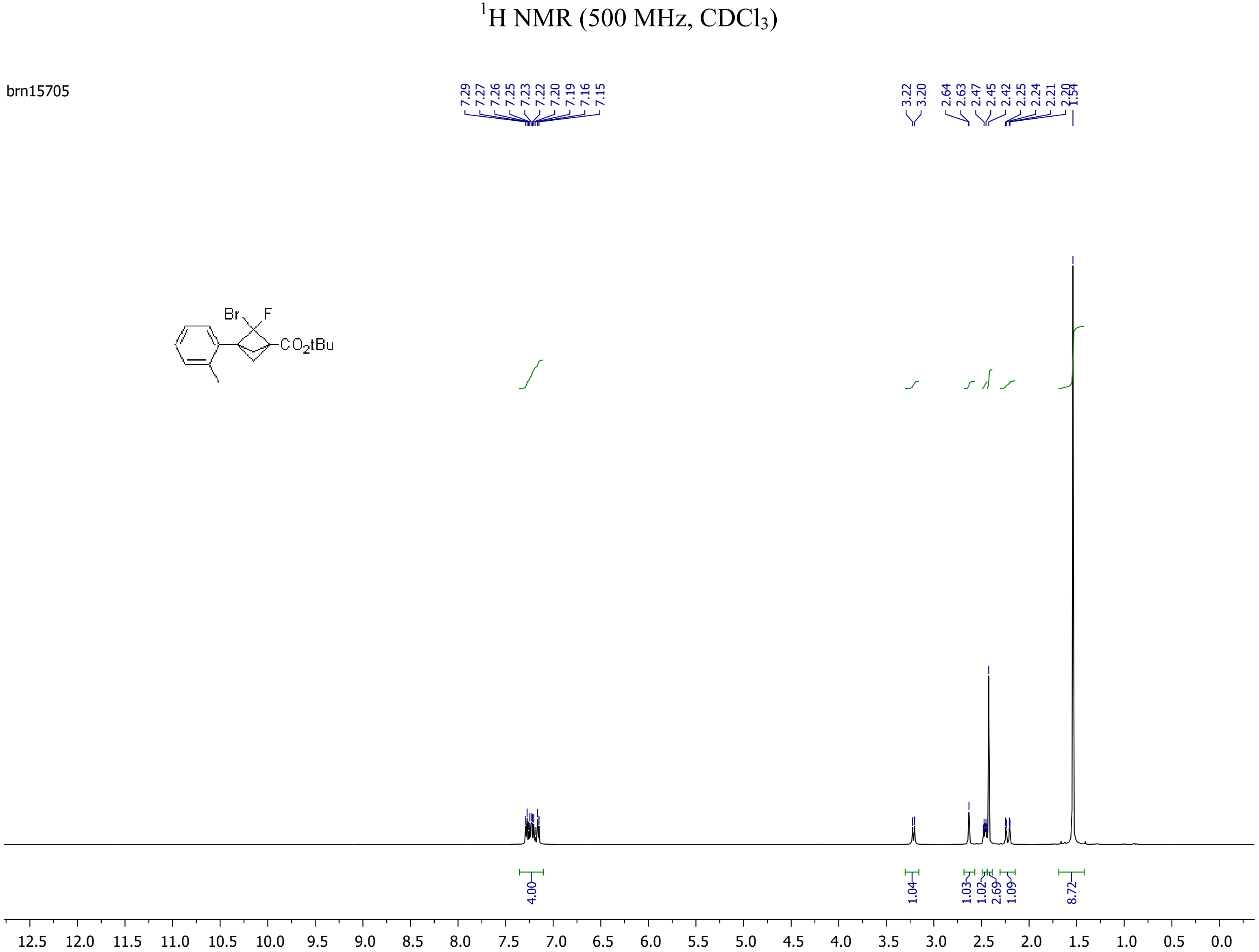

$^{13}C\{^1H\}$ NMR (126 MHz, $CDCl_3$)

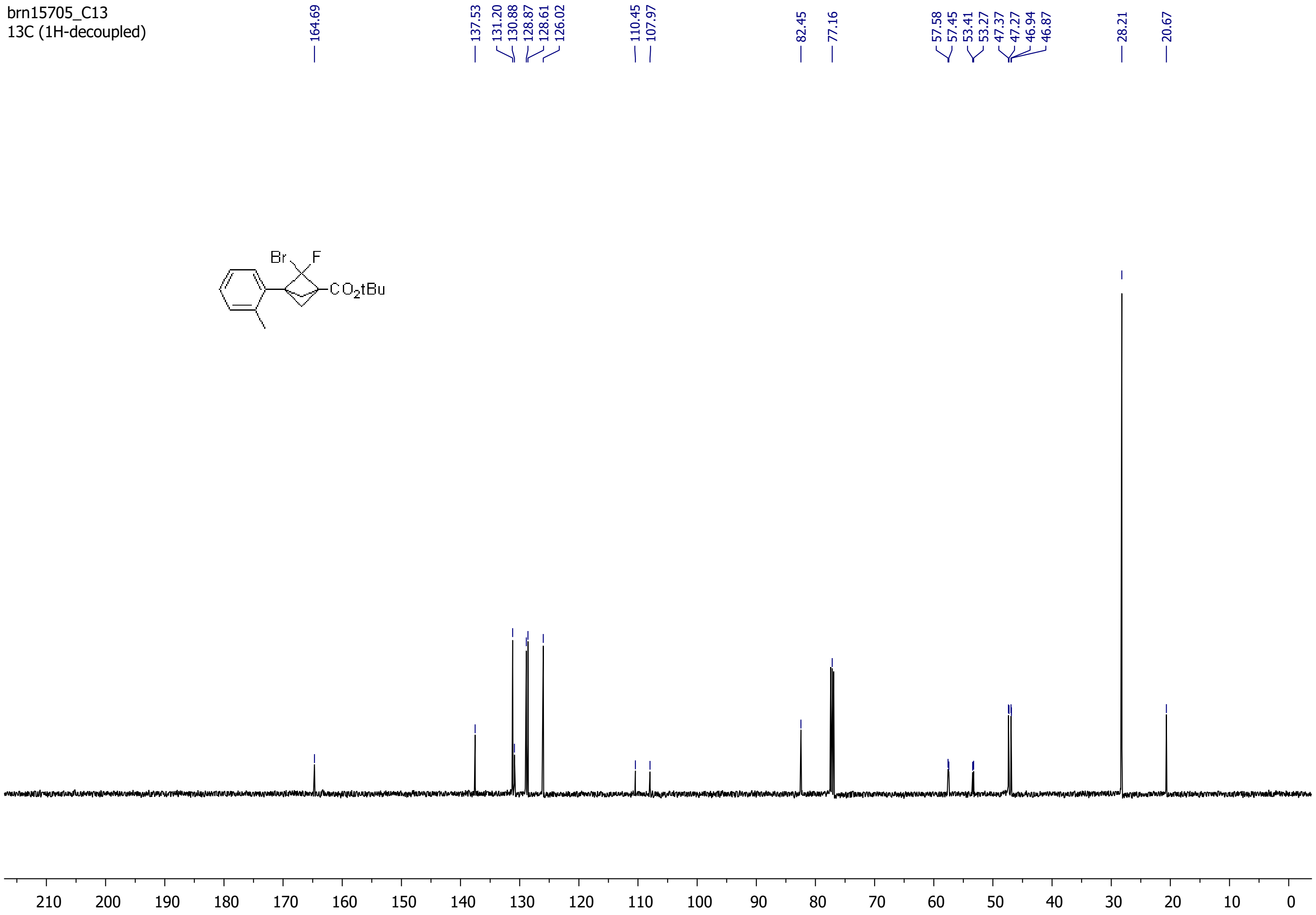

$^{19}F\{^{1}H\}$ NMR (376 MHz, $CDCl_3$)

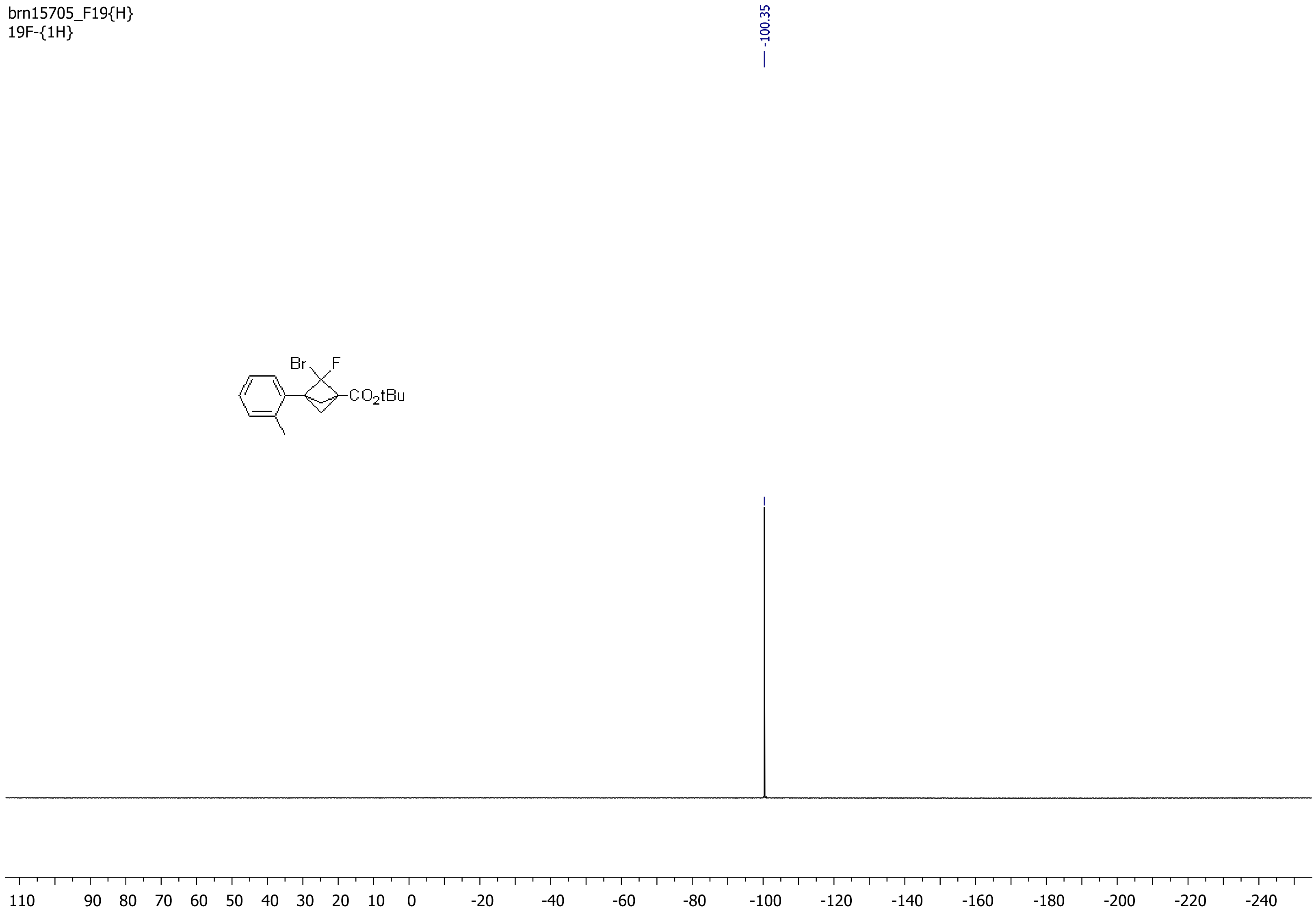

**Compound 14a**

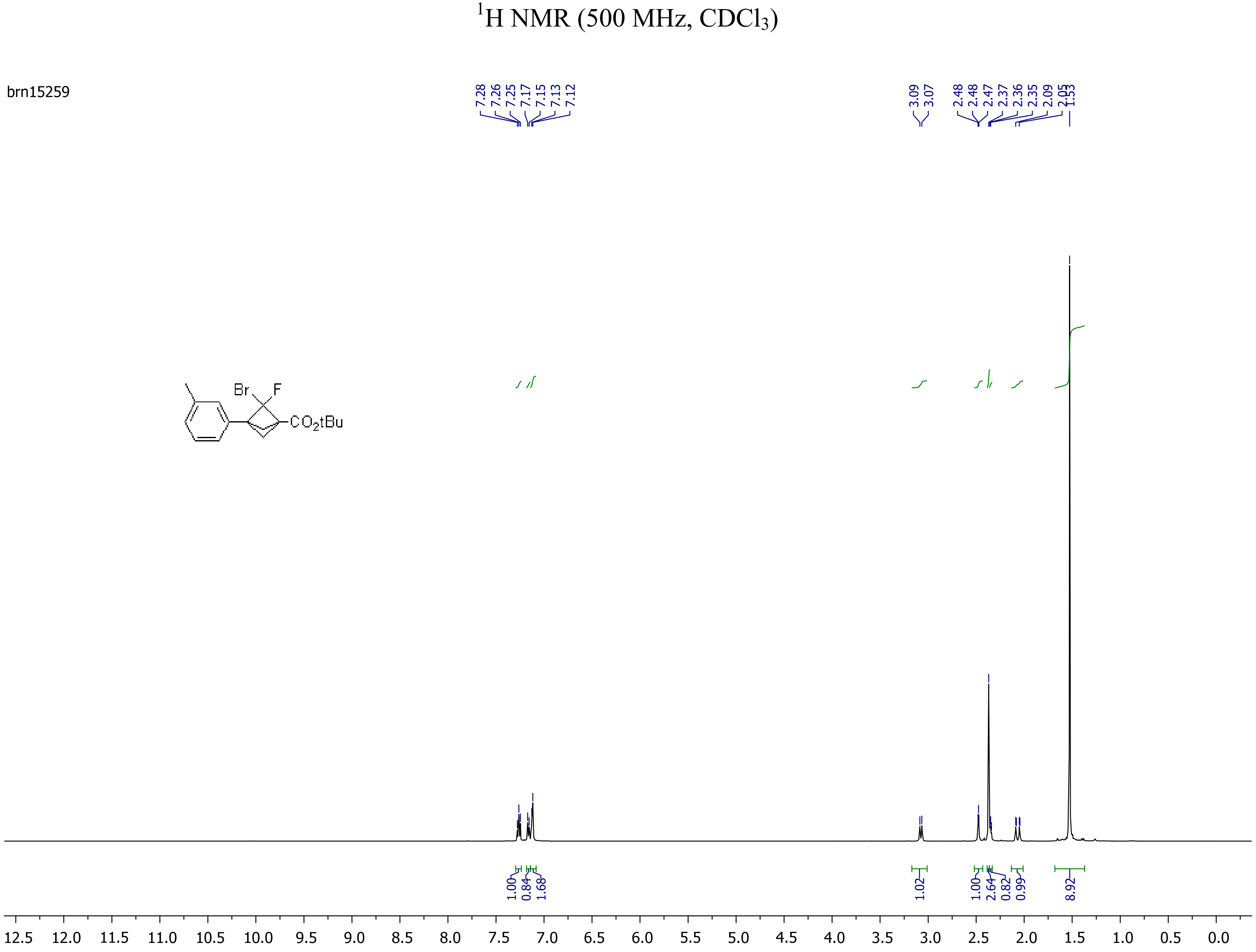

$^{13}C\{^{1}H\}$ NMR (126 MHz, $CDCl_3$)

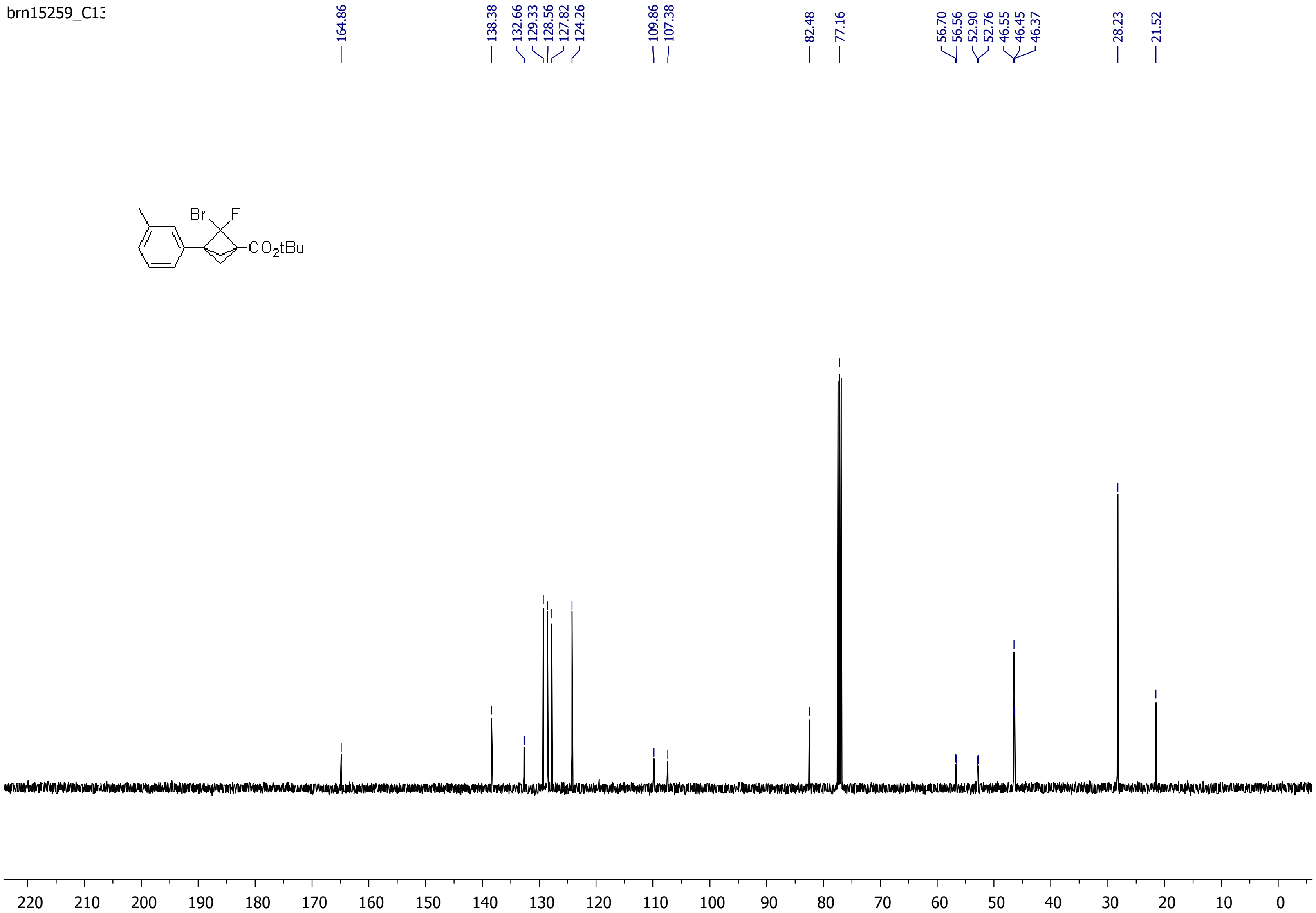

$^{19}F\{^{1}H\}$ NMR (376 MHz, $CDCl_3$)

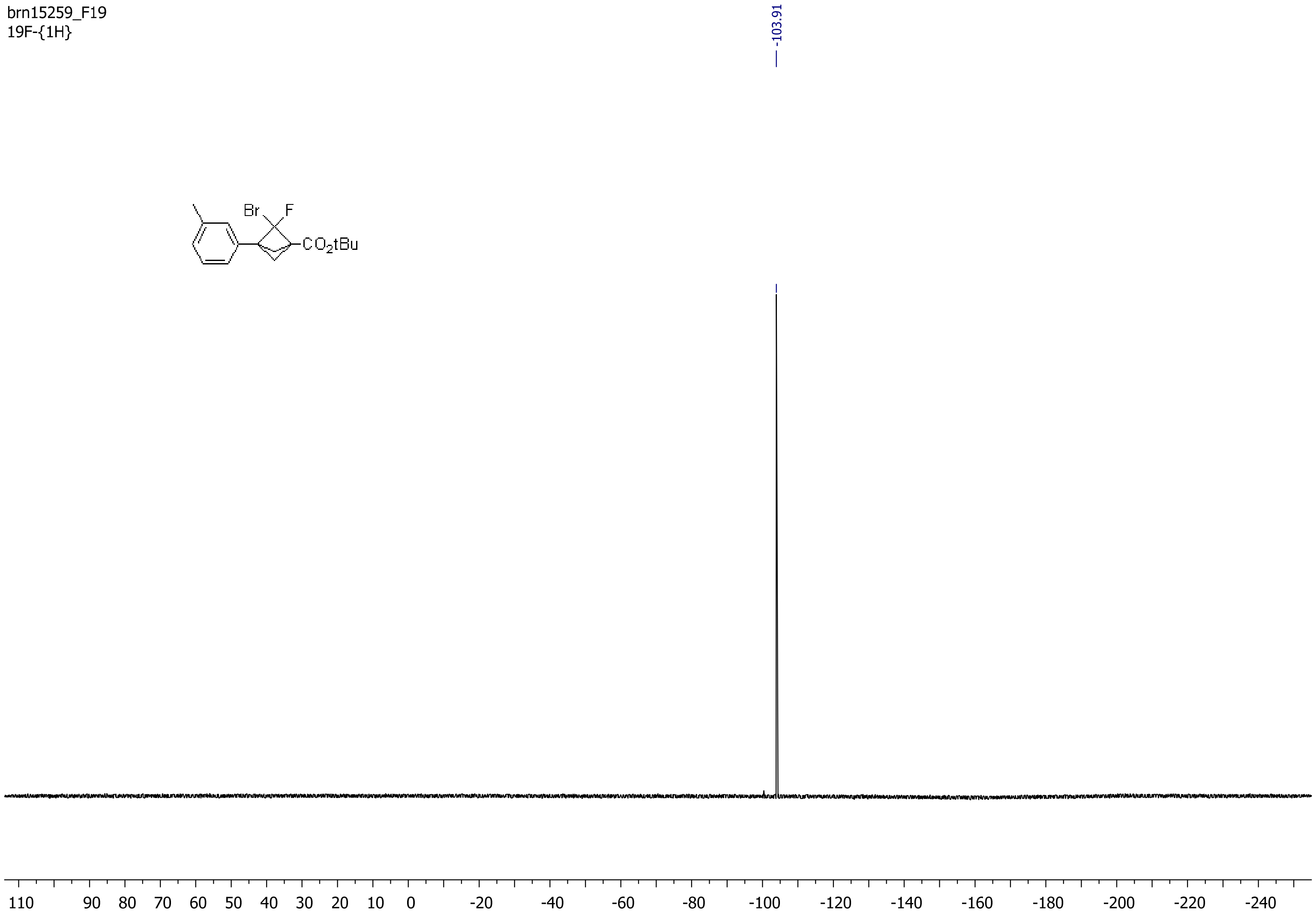

**Compound 15a**

$^1$H NMR (500 MHz, $CDCl_3$)

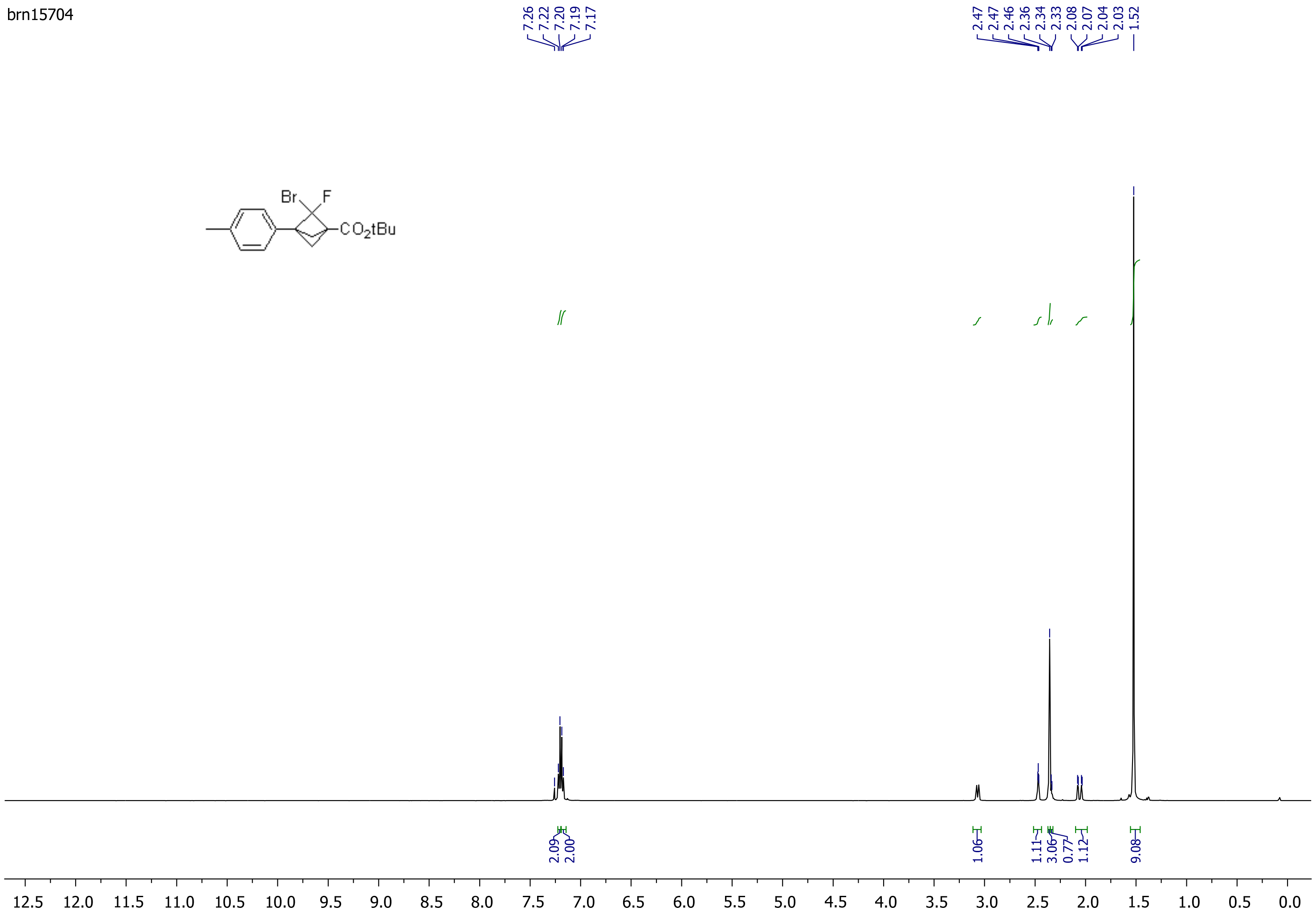

$^{13}C\{^1H\}$ NMR (126 MHz, $CDCl_3$)

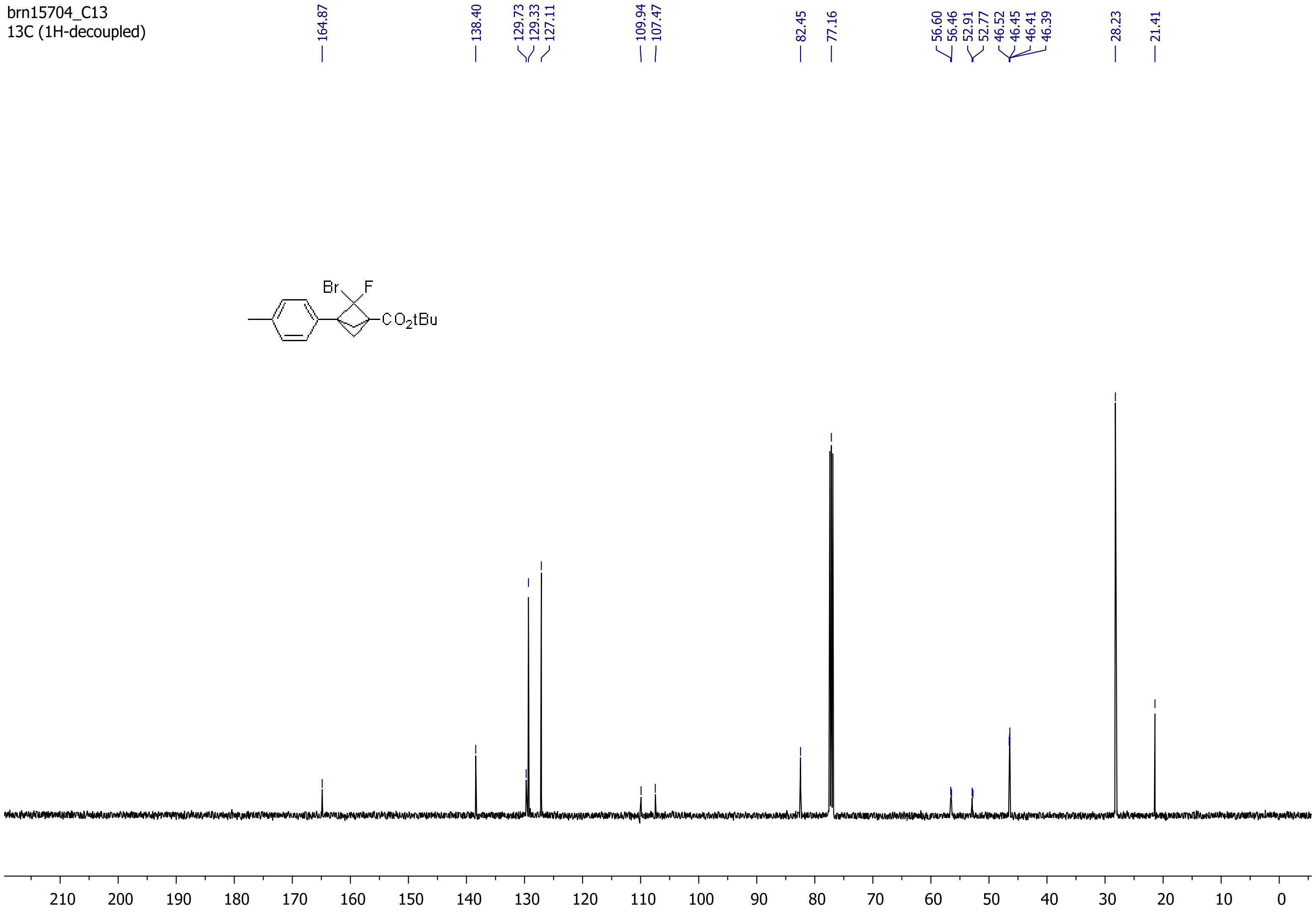

$^{19}F\{^1H\}$ NMR (376 MHz, $CDCl_3$)

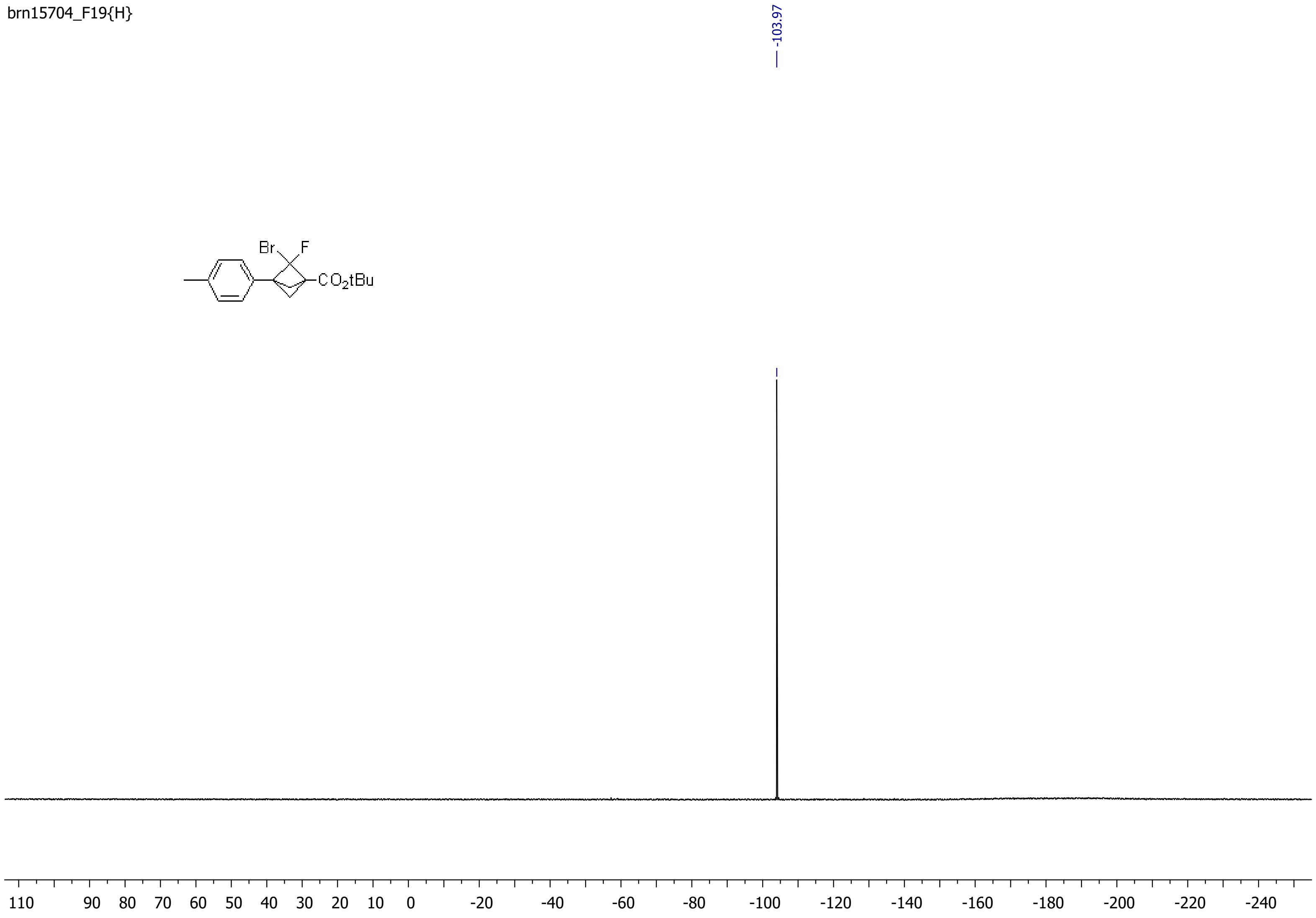

**Compound 16a**

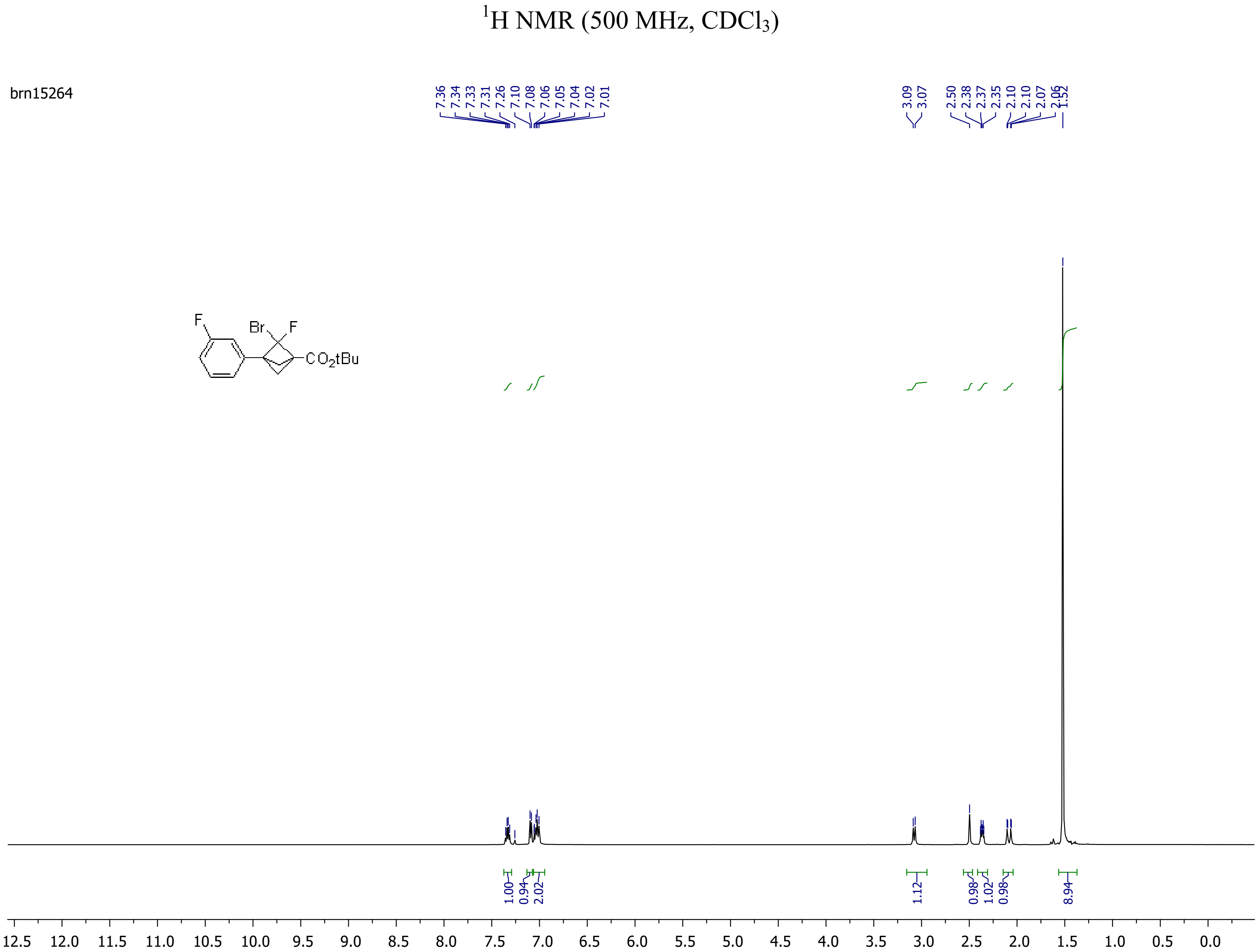

$^{13}C\{^1H\}$ NMR (151 MHz, $CDCl_3$)

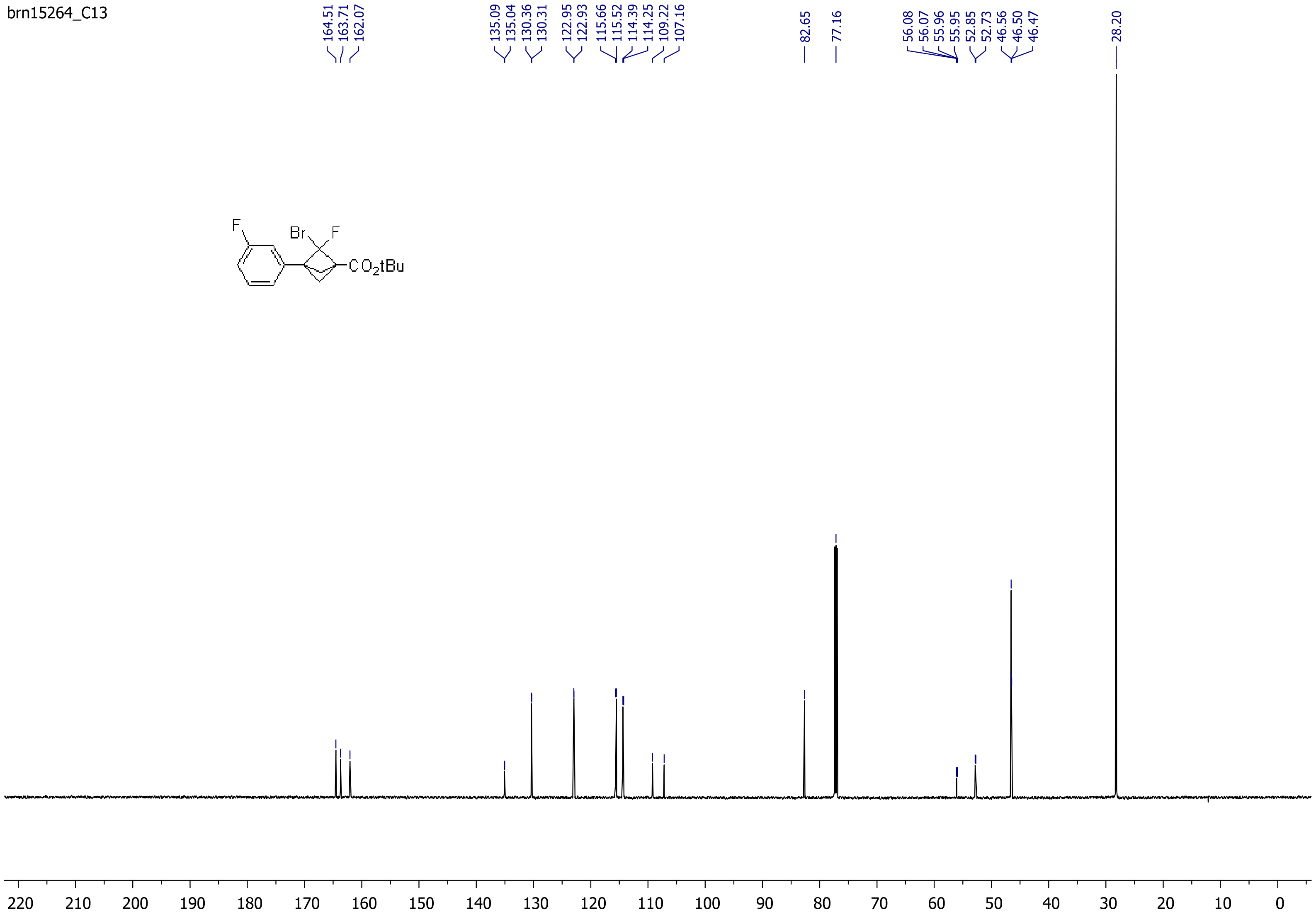

## $^{19}F\{^1H\}$ NMR (376 MHz, $CDCl_3$)

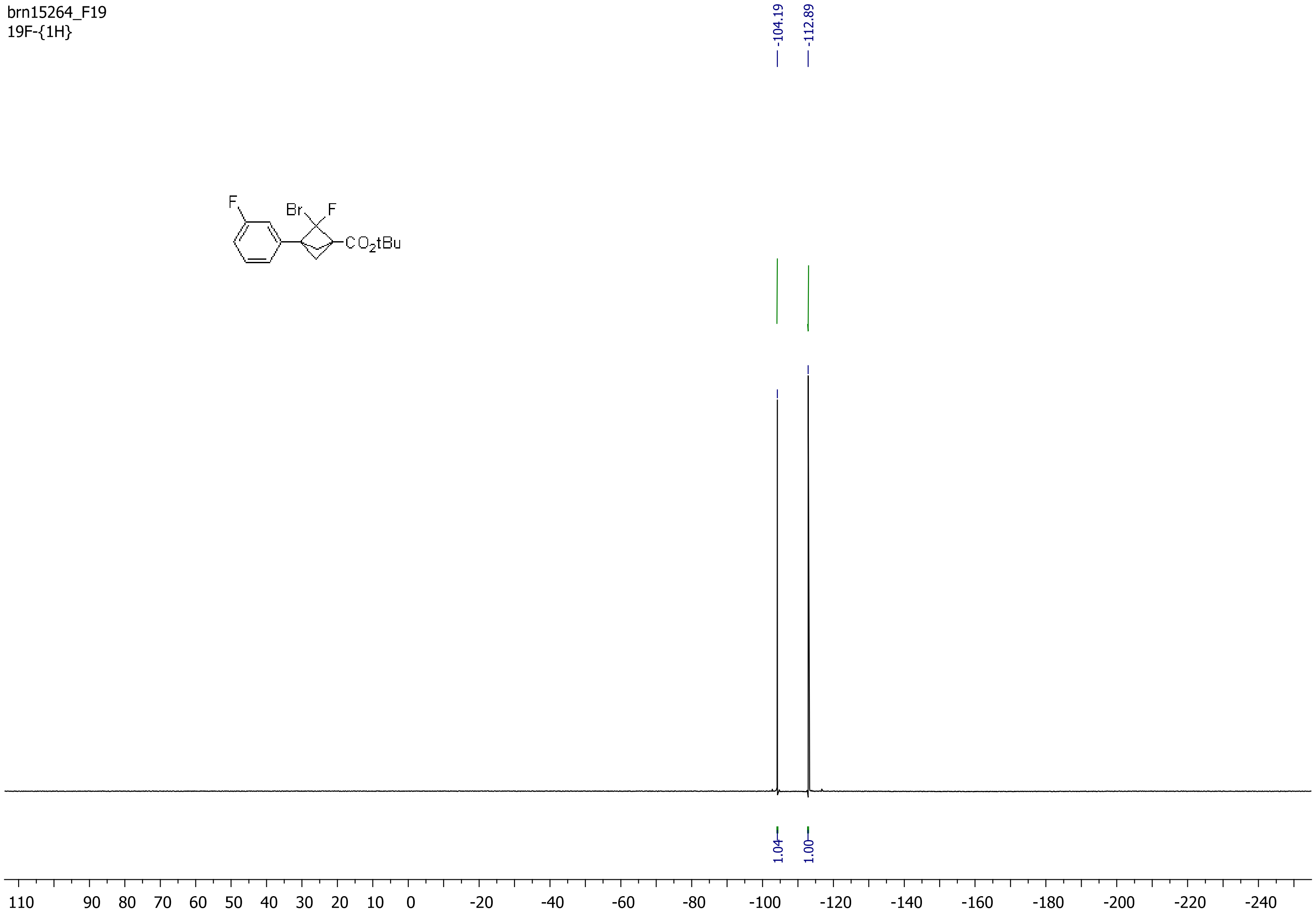

**Compound 17a**

$^1$H NMR (400 MHz, $CDCl_3$)

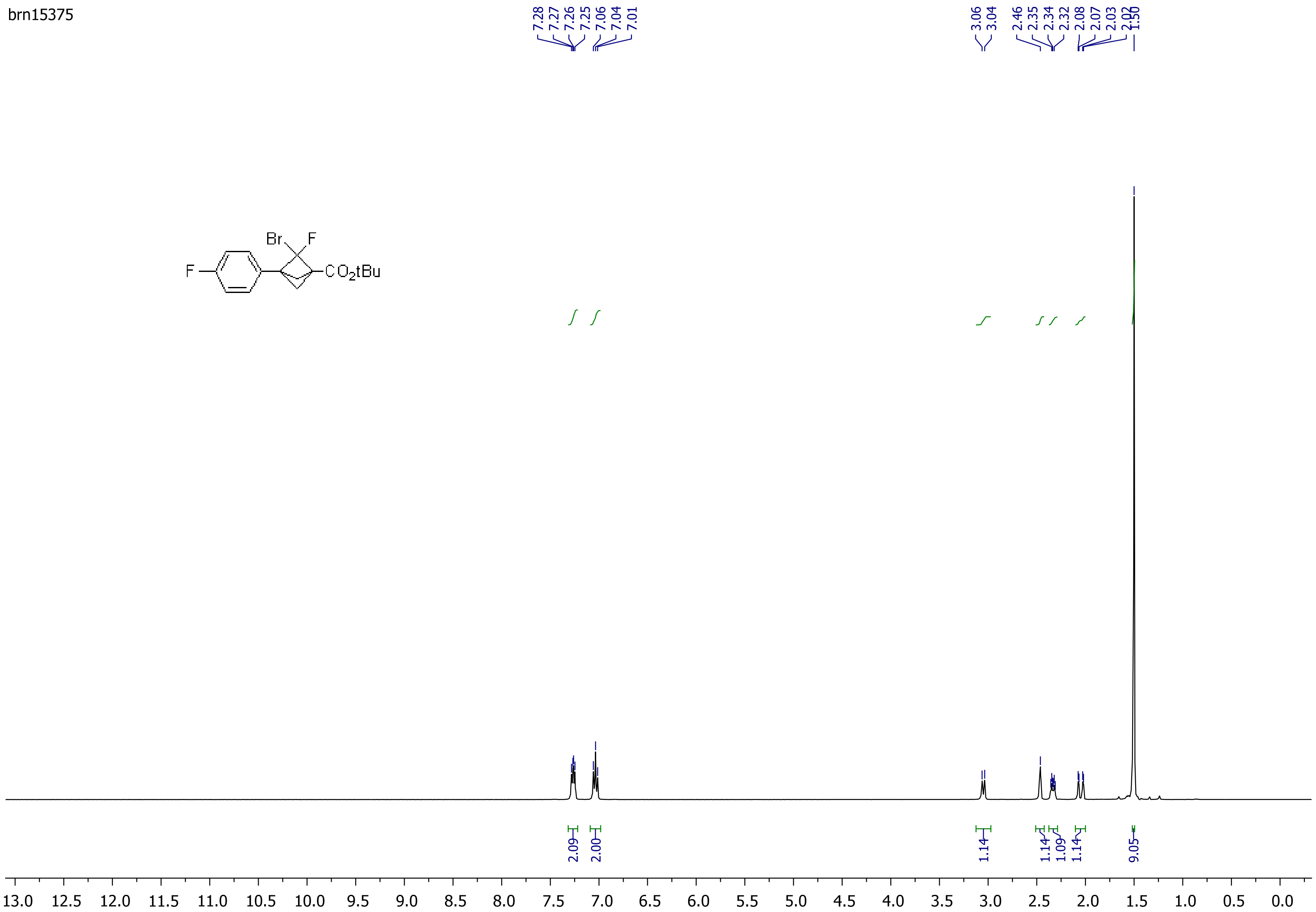

$^{13}C\{^{1}H\}$ NMR (126 MHz, $CDCl_3$)

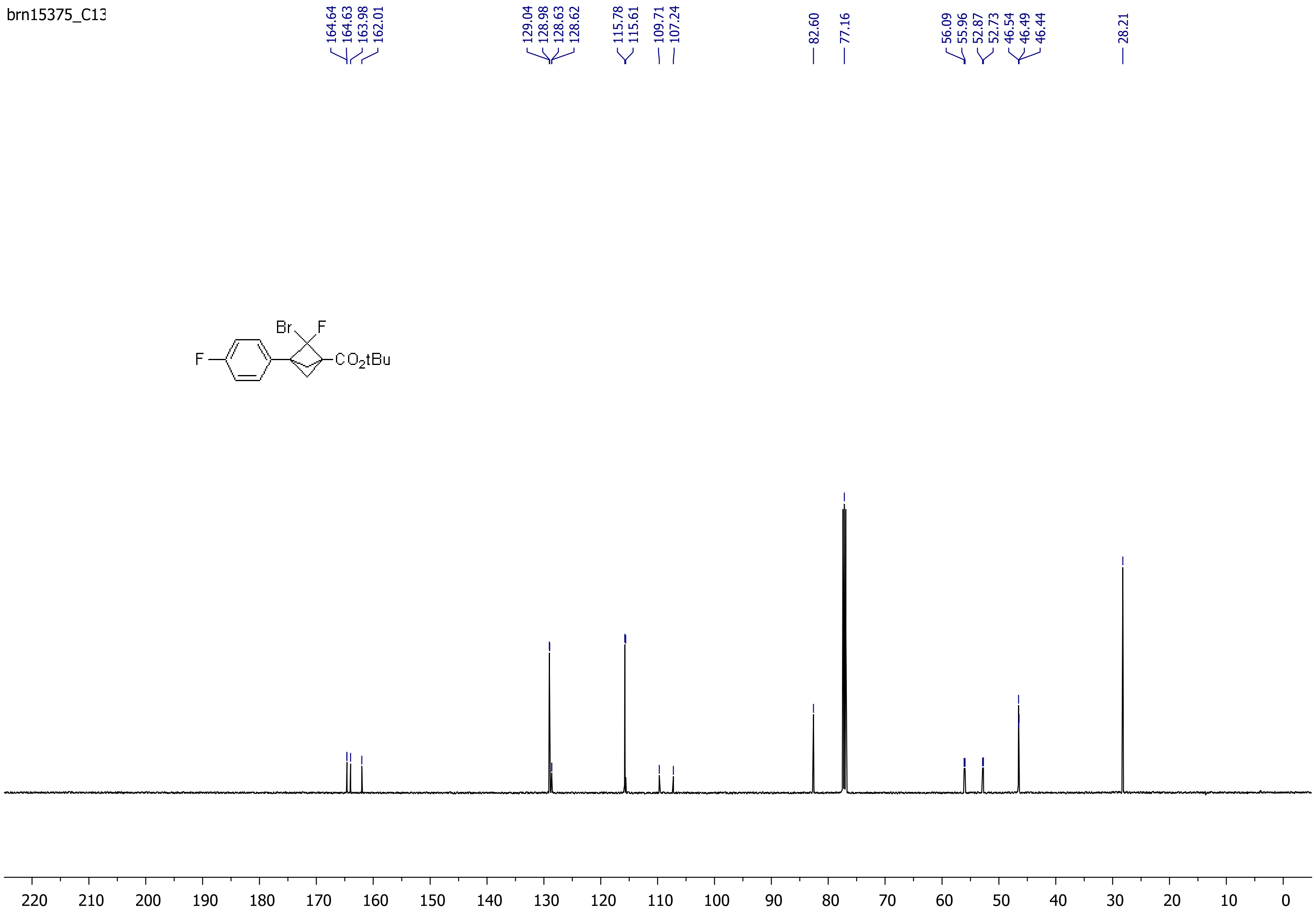

$^{19}F\{^{1}H\}$ NMR (376 MHz, $CDCl_3$)

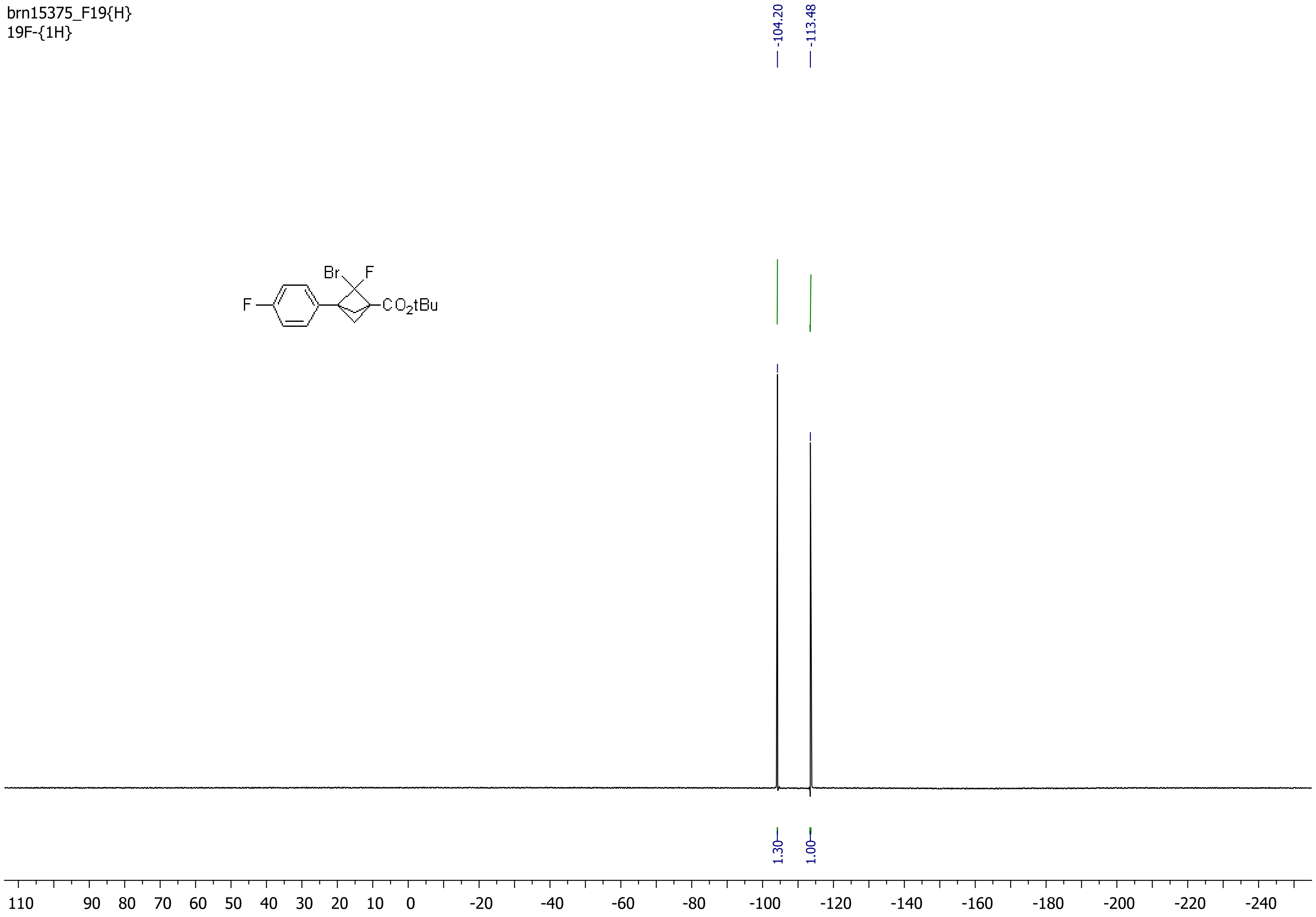

**Compound 18a**

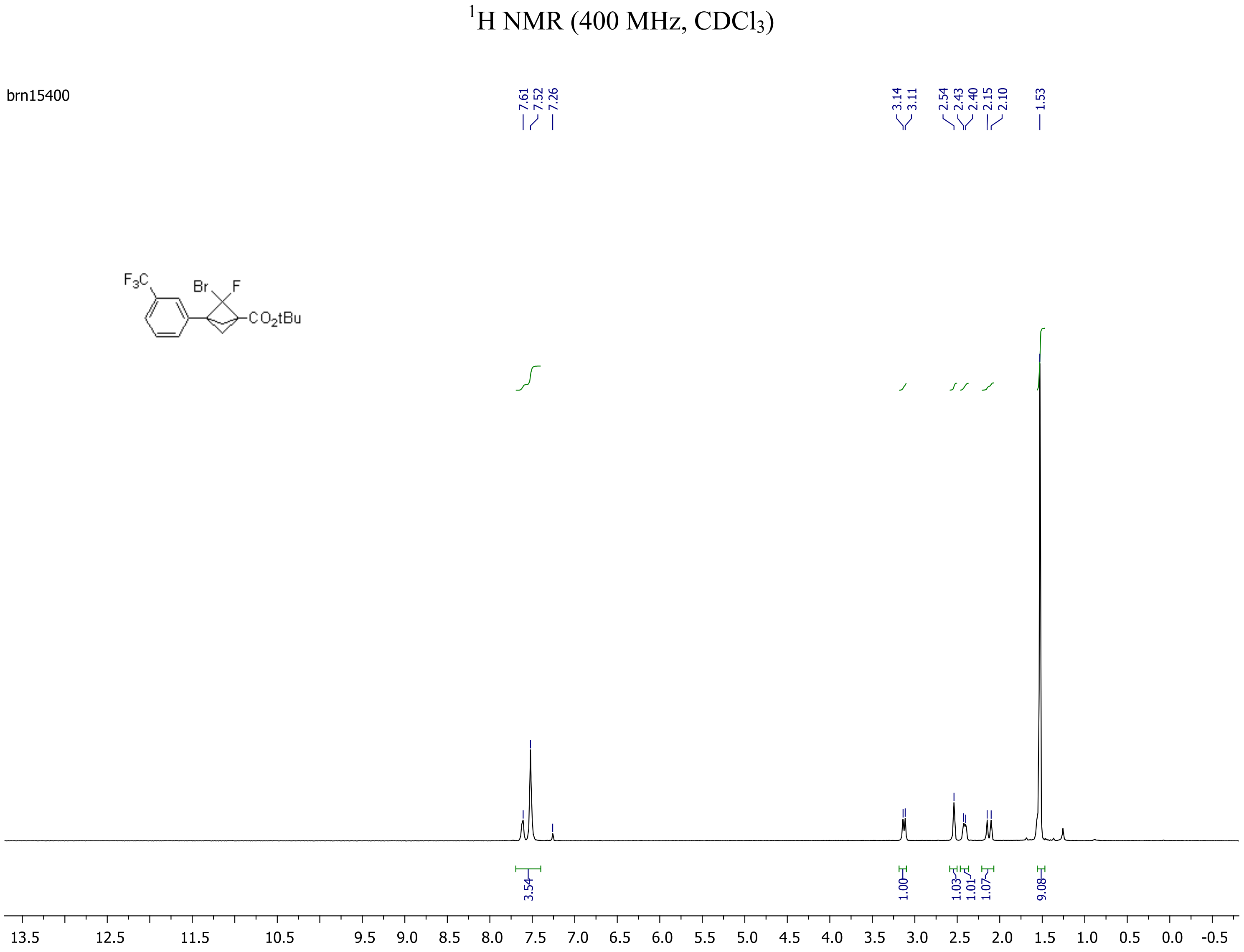

$^{13}C\{^1H\}$ NMR (101 MHz, $CDCl_3$)

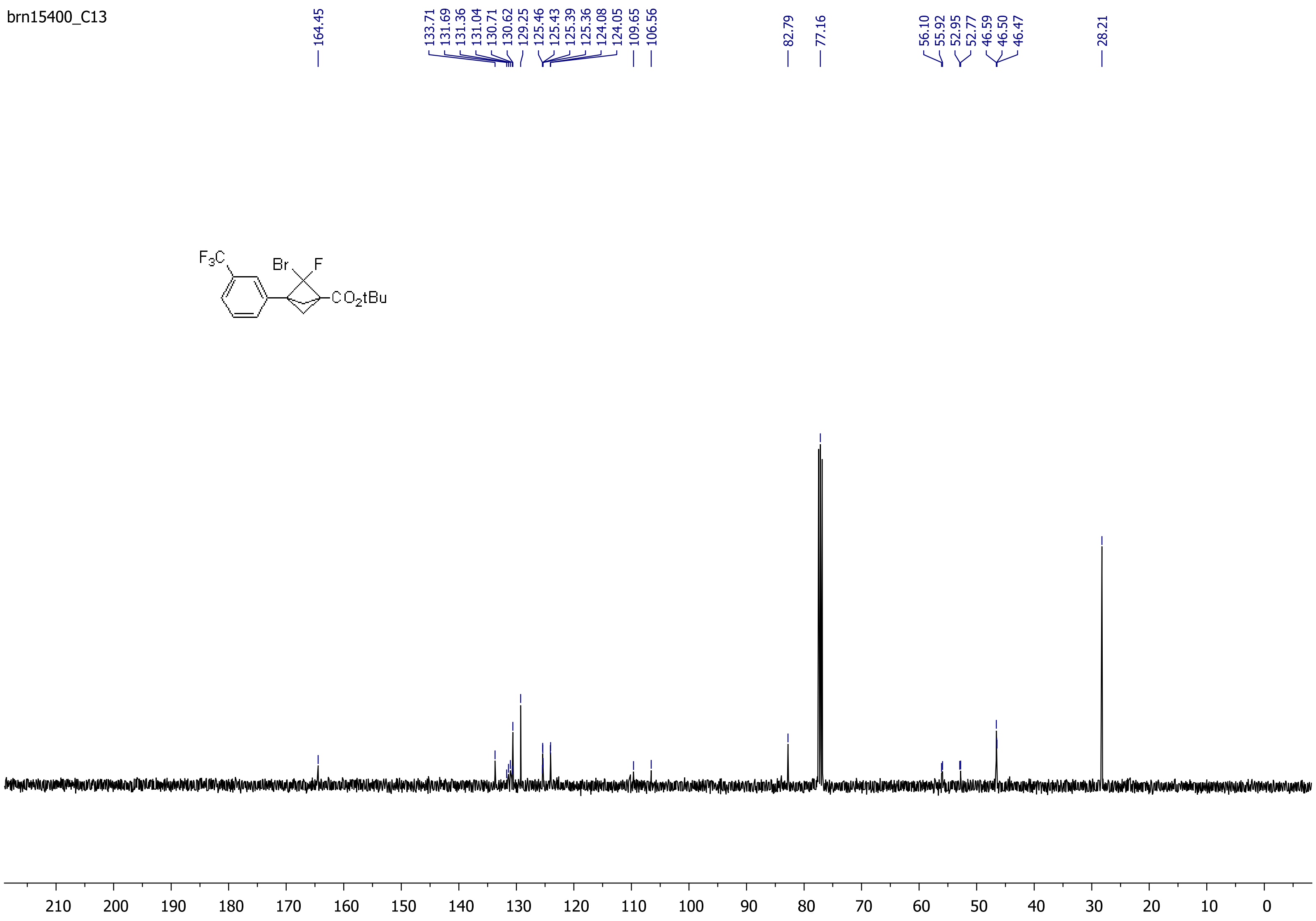

$^{19}F\{^1H\}$ NMR (376 MHz, $CDCl_3$)

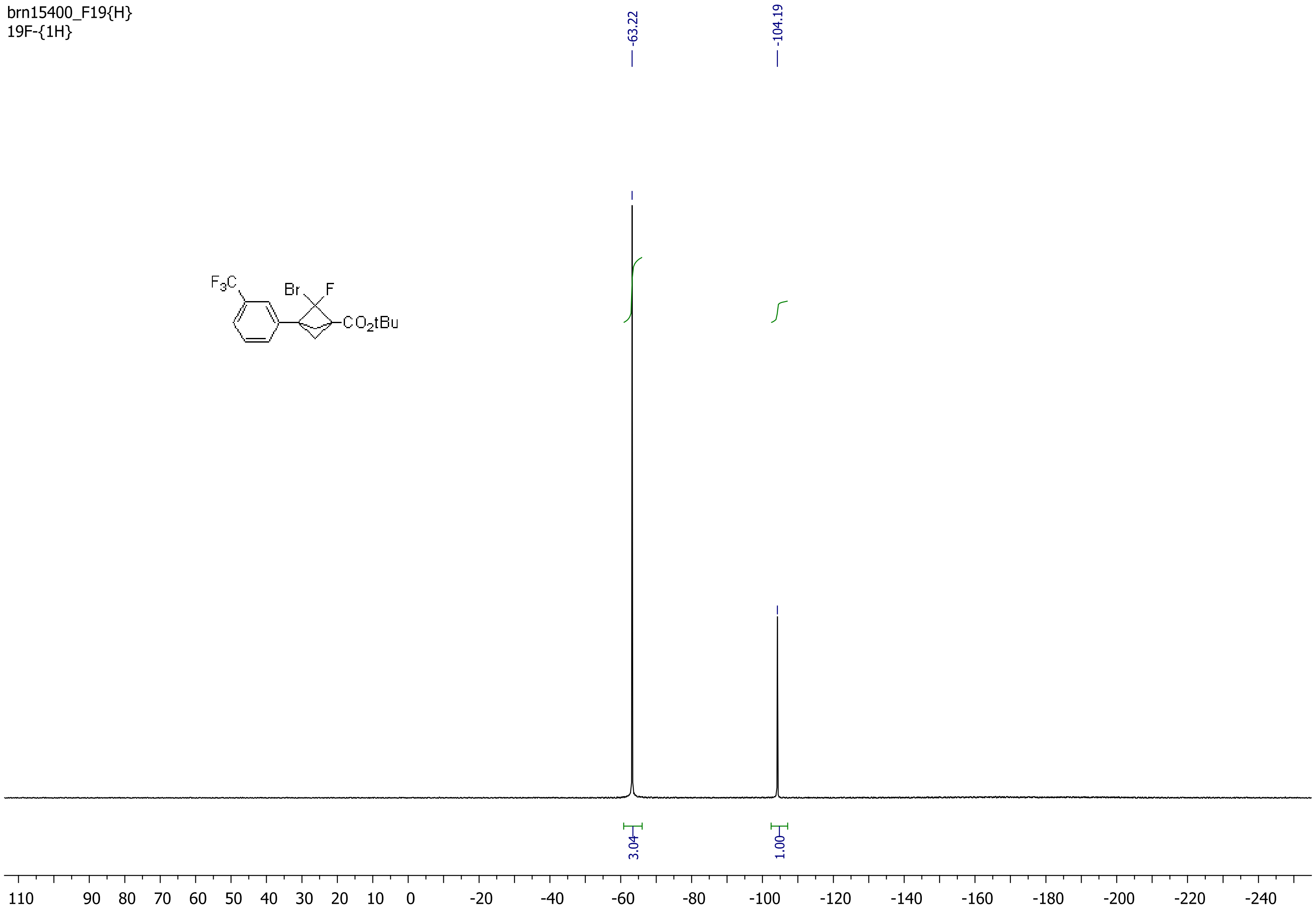

**Compound 19a**

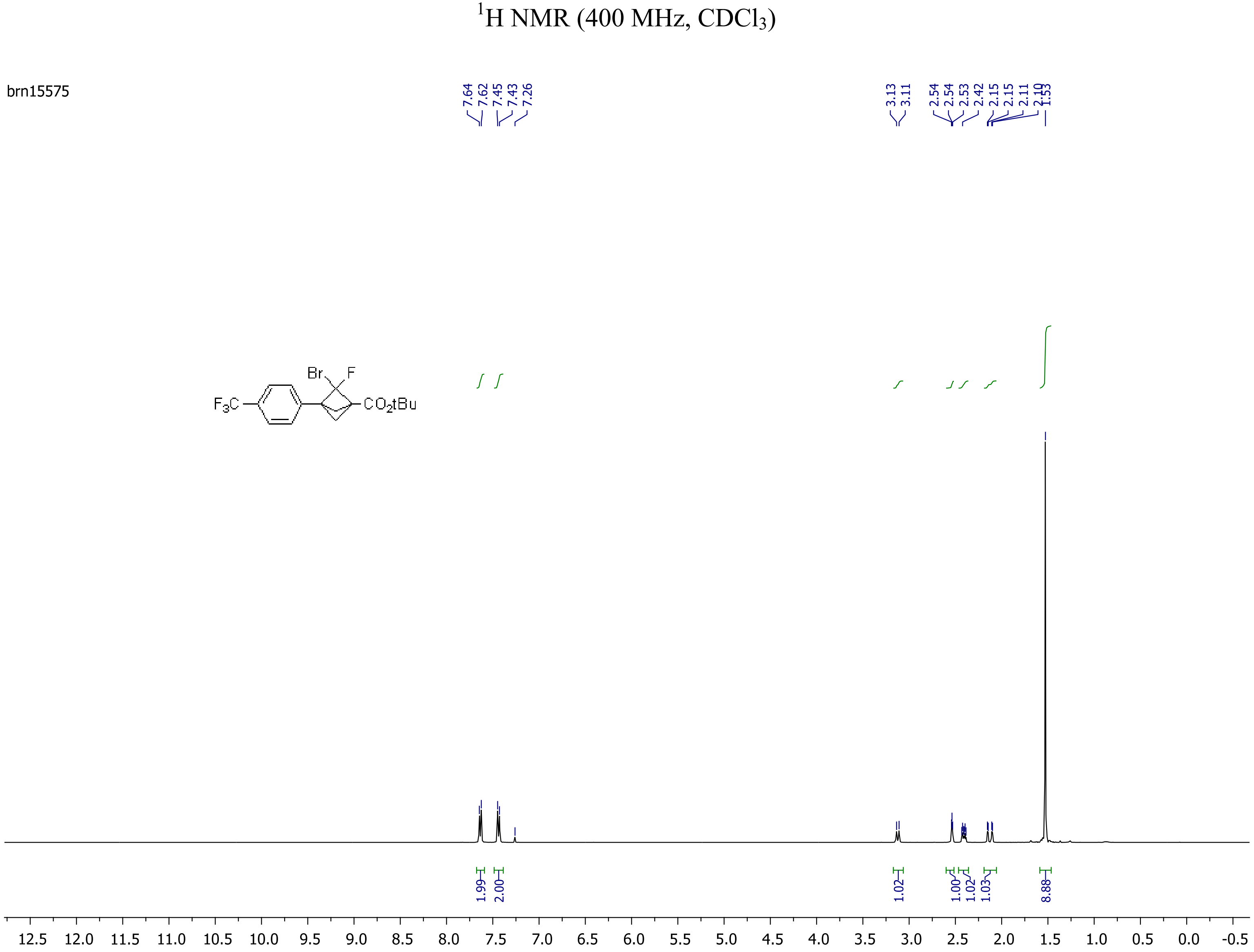

$^{13}C\{^{1}H\}$ NMR (151 MHz, $CDCl_3$)

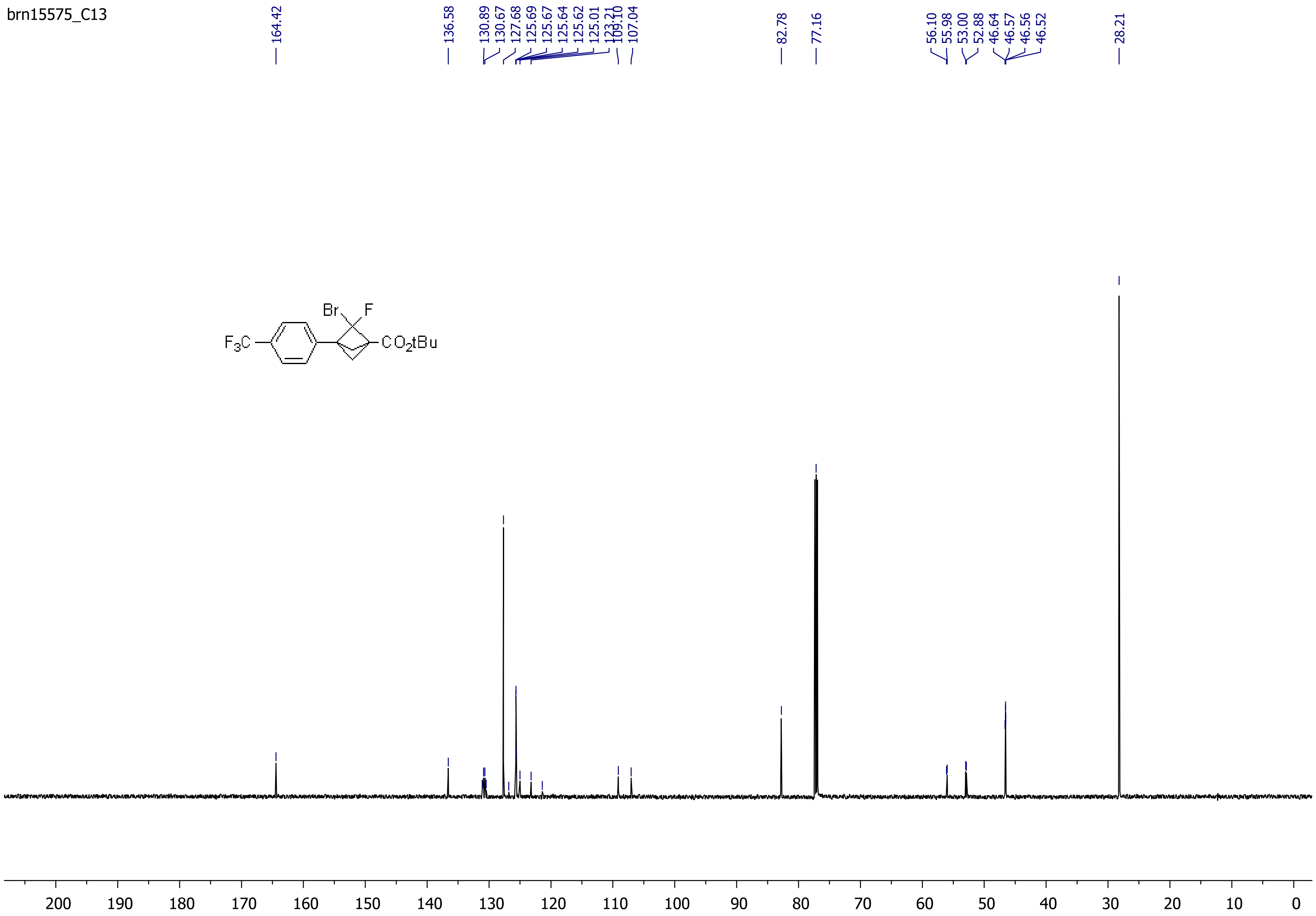

$^{19}F\{^{1}H\}$ NMR (376 MHz, $CDCl_3$)

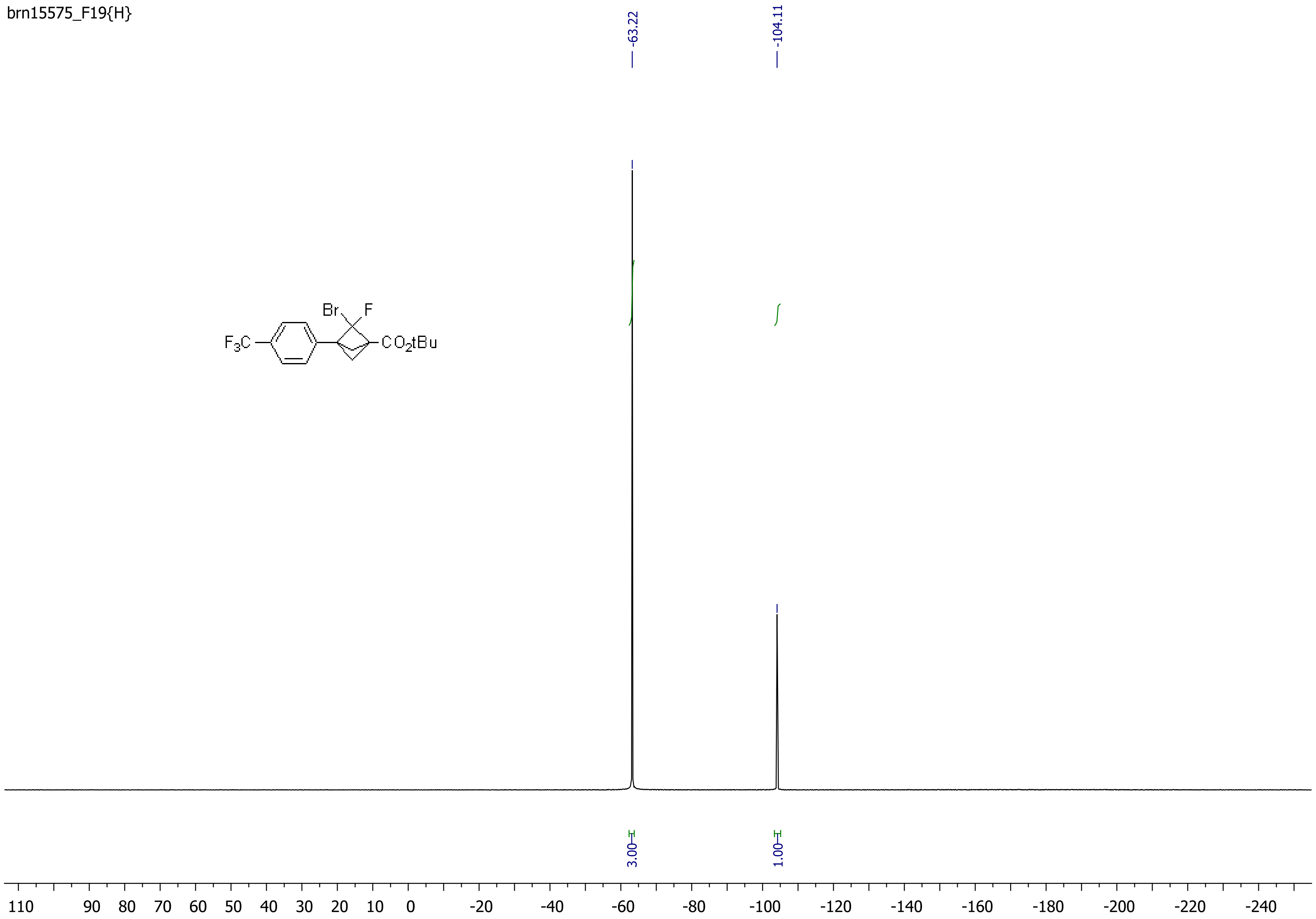

**Compound 9**

$^1$H NMR (500 MHz, $CDCl_3$)

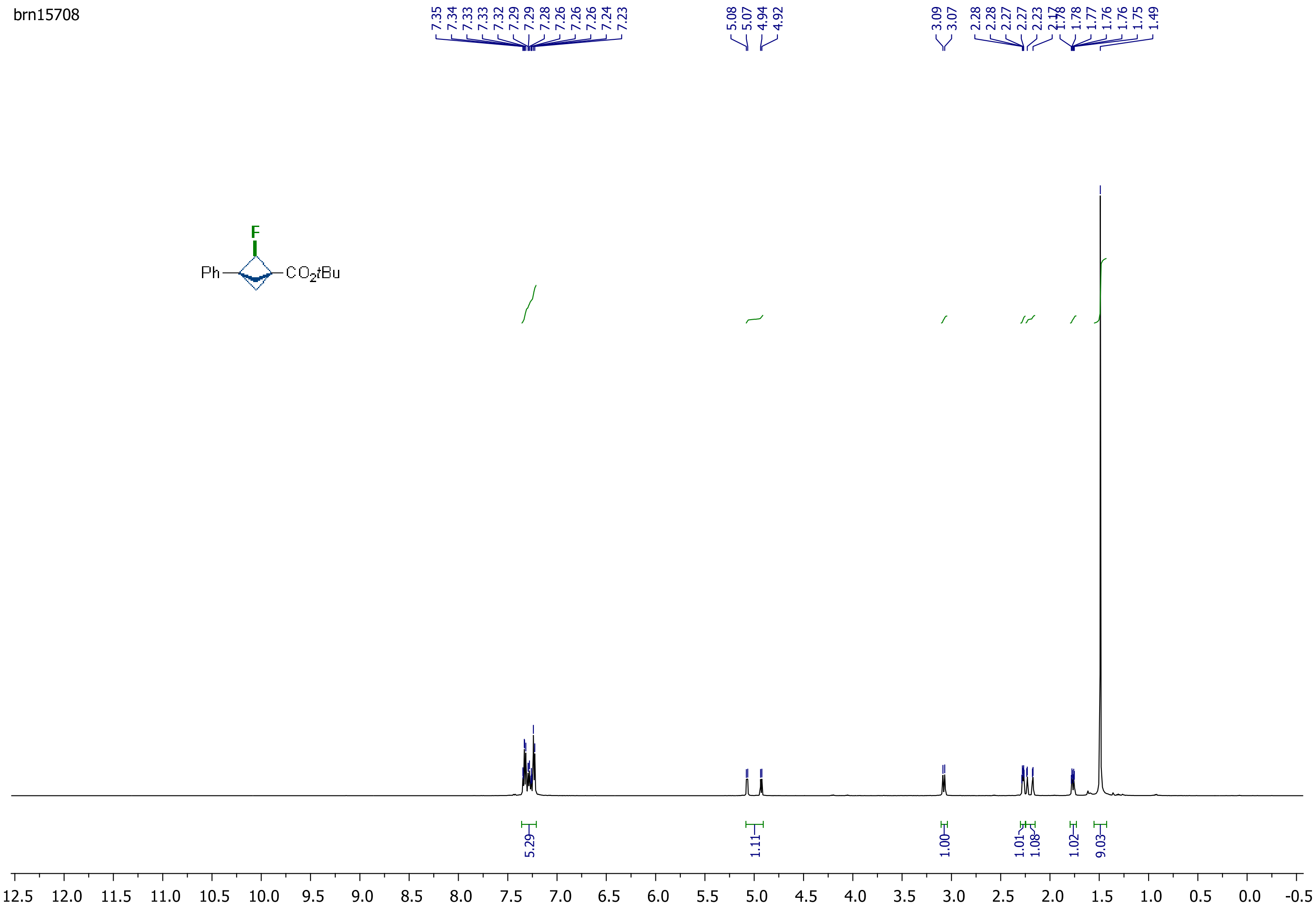

$^{13}C\{^1H\}$ NMR (126 MHz, $CDCl_3$)

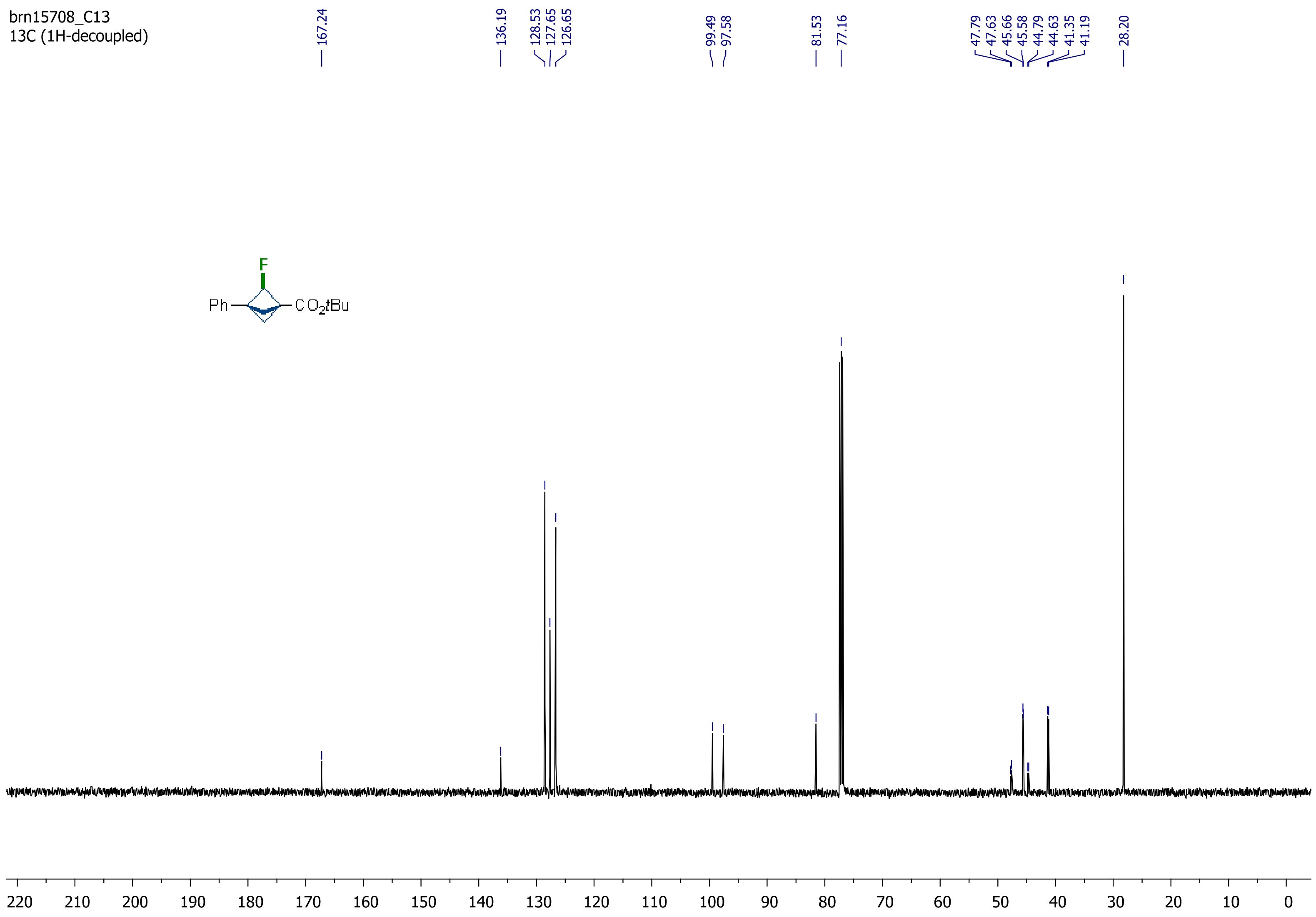

$^{19}F\{^{1}H\}$ NMR (376 MHz, $CDCl_3$)

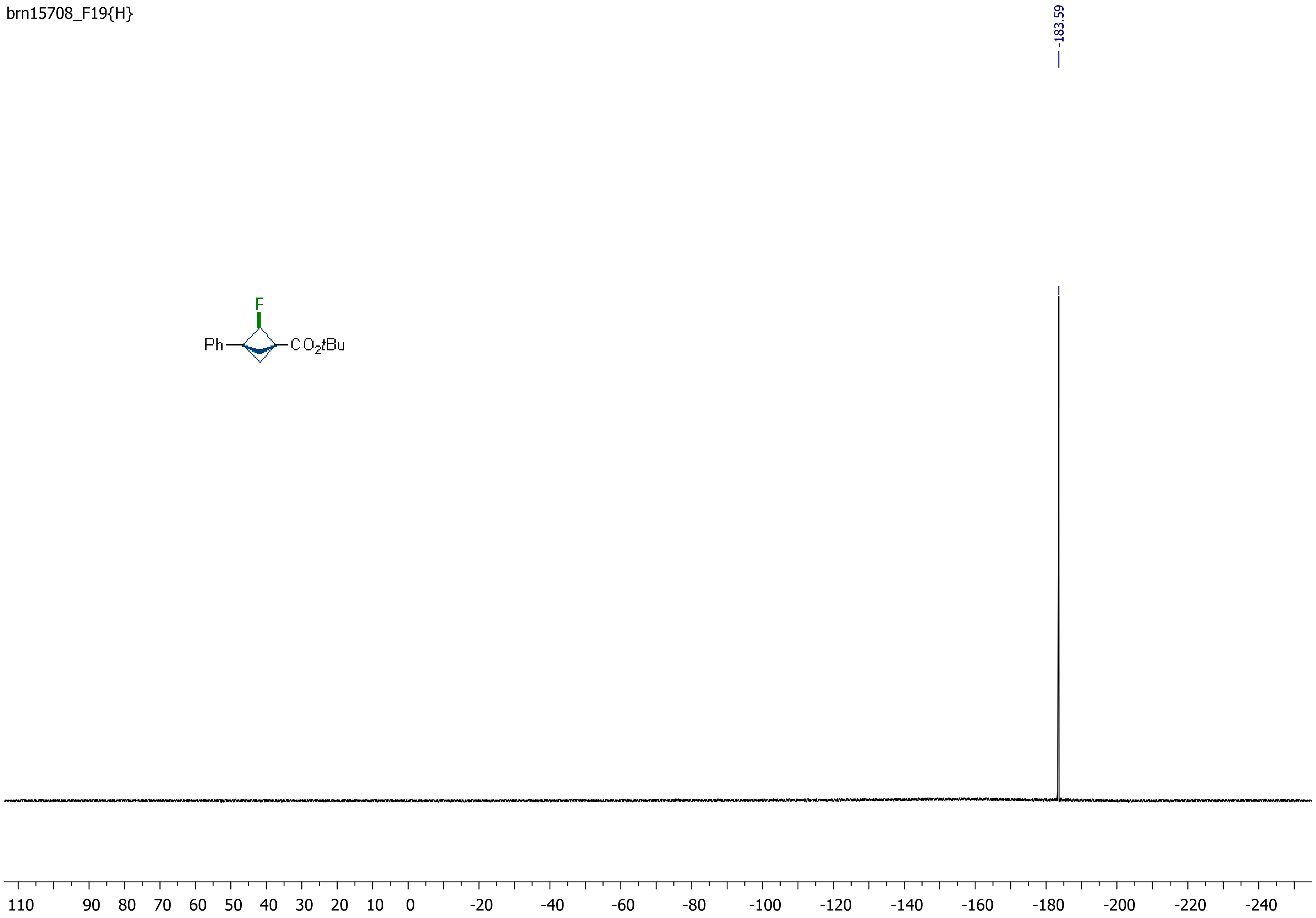

**Compound 13b**

$^{1}$H NMR (500 MHz, $CDCl_3$)

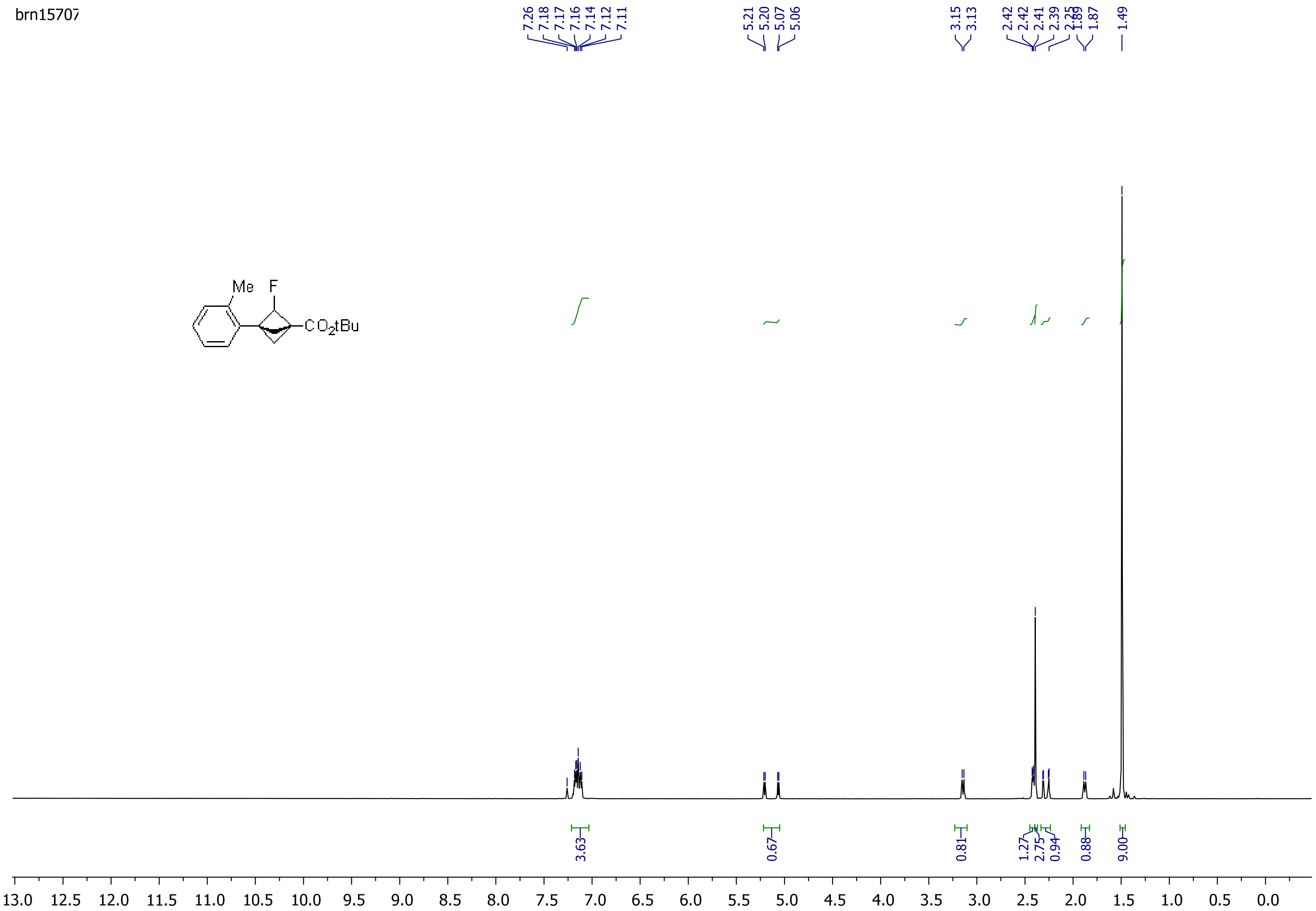

# $^{13}C\{^{1}H\}$ NMR (126 MHz, $CDCl_3$)

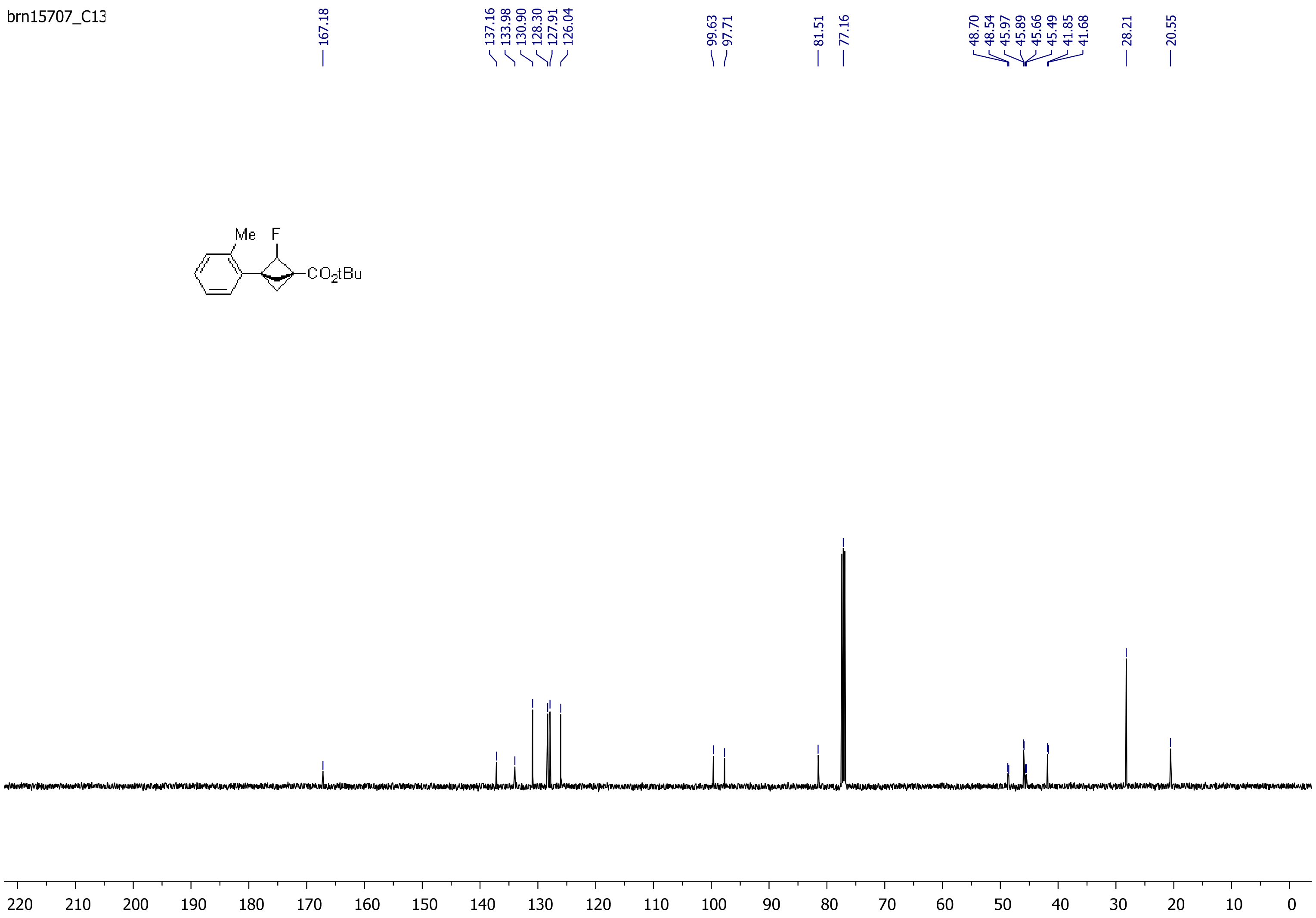

$^{19}F\{^1H\}$ NMR (376 MHz, $CDCl_3$)

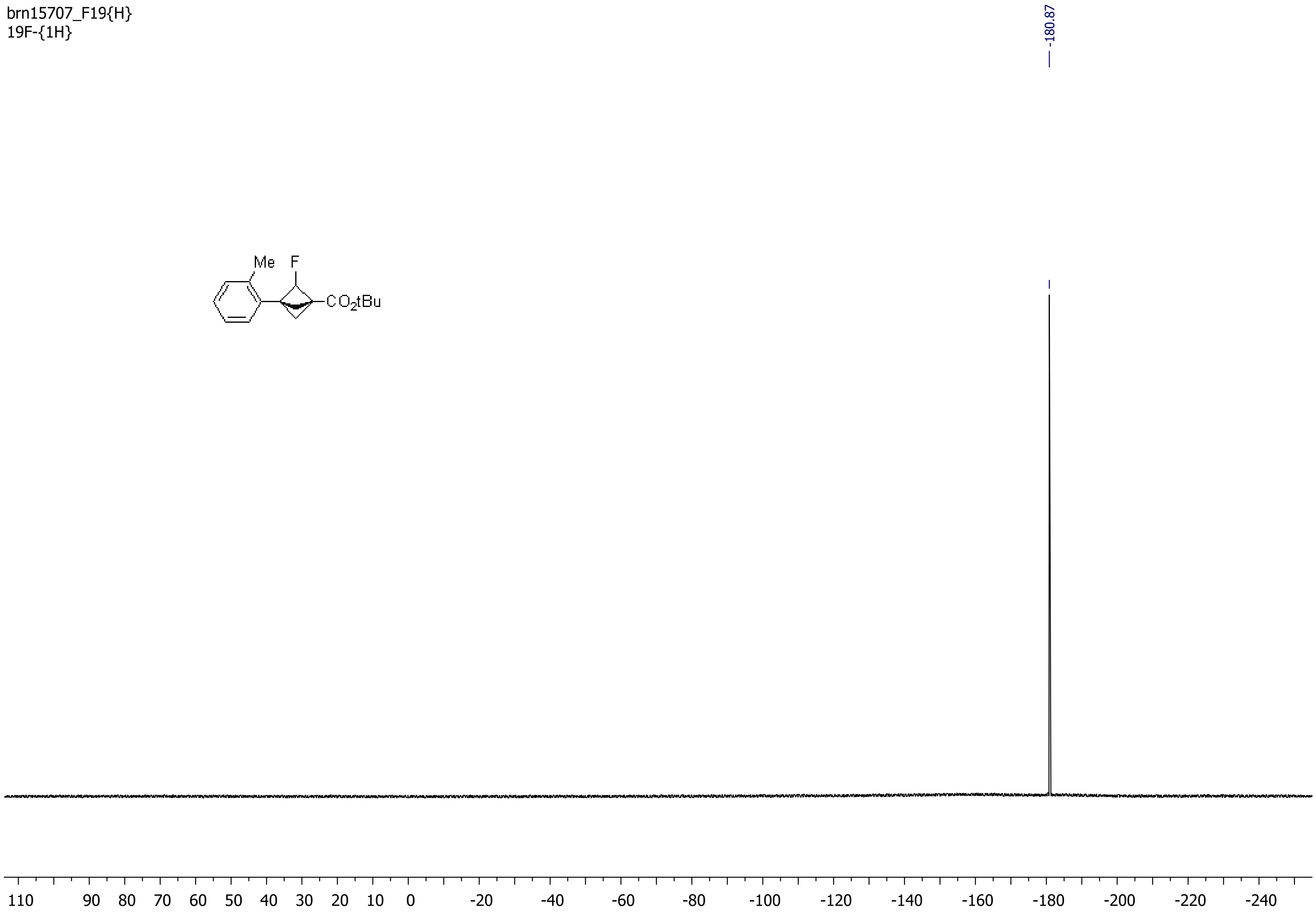

**Compound 14b**

$^{1}H$ NMR (400 MHz, $CDCl_3$)

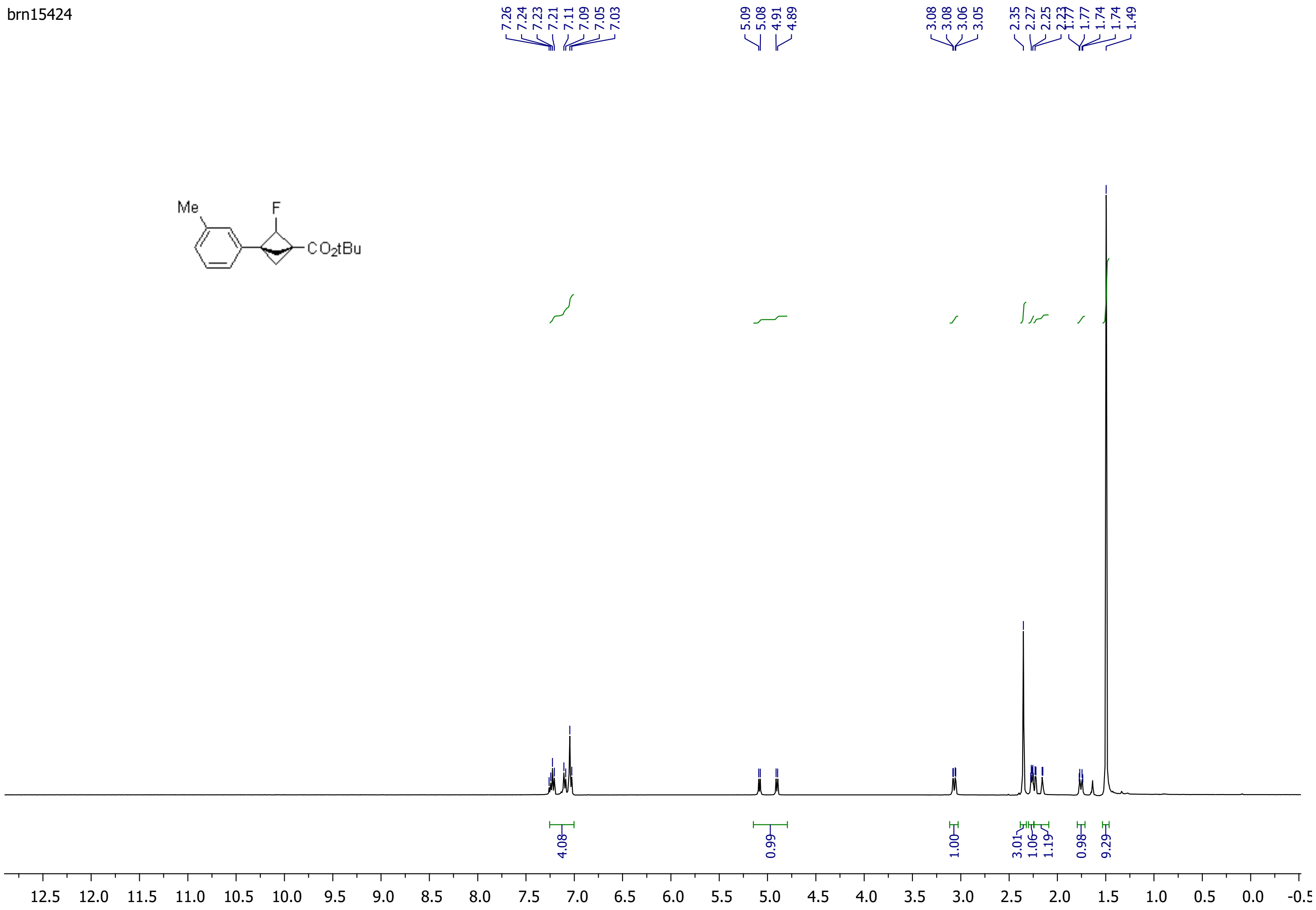

$^{13}C\{^{1}H\}$ NMR (126 MHz, $CDCl_3$)

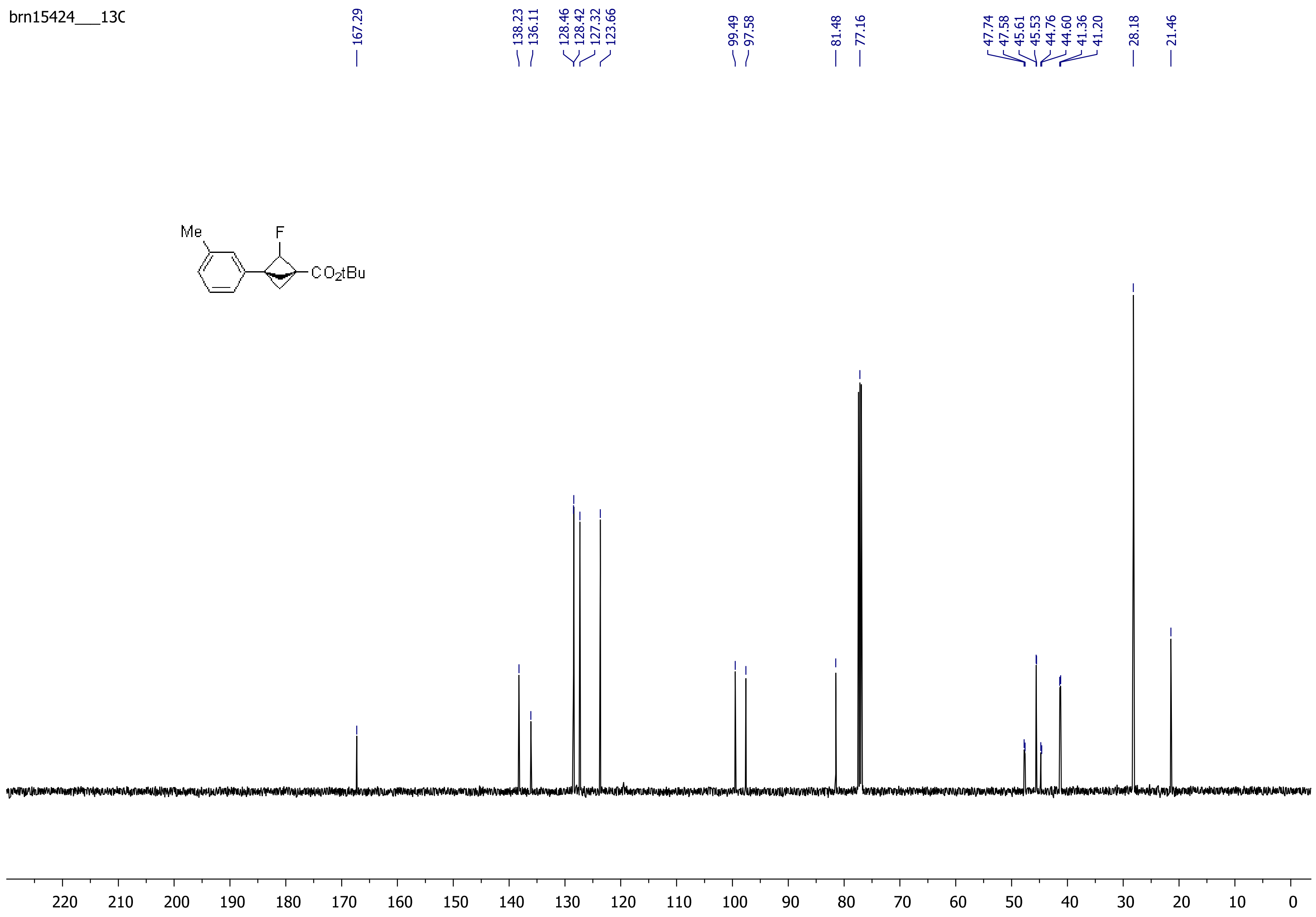

$^{19}F\{^1H\}$ NMR (376 MHz, $CDCl_3$)

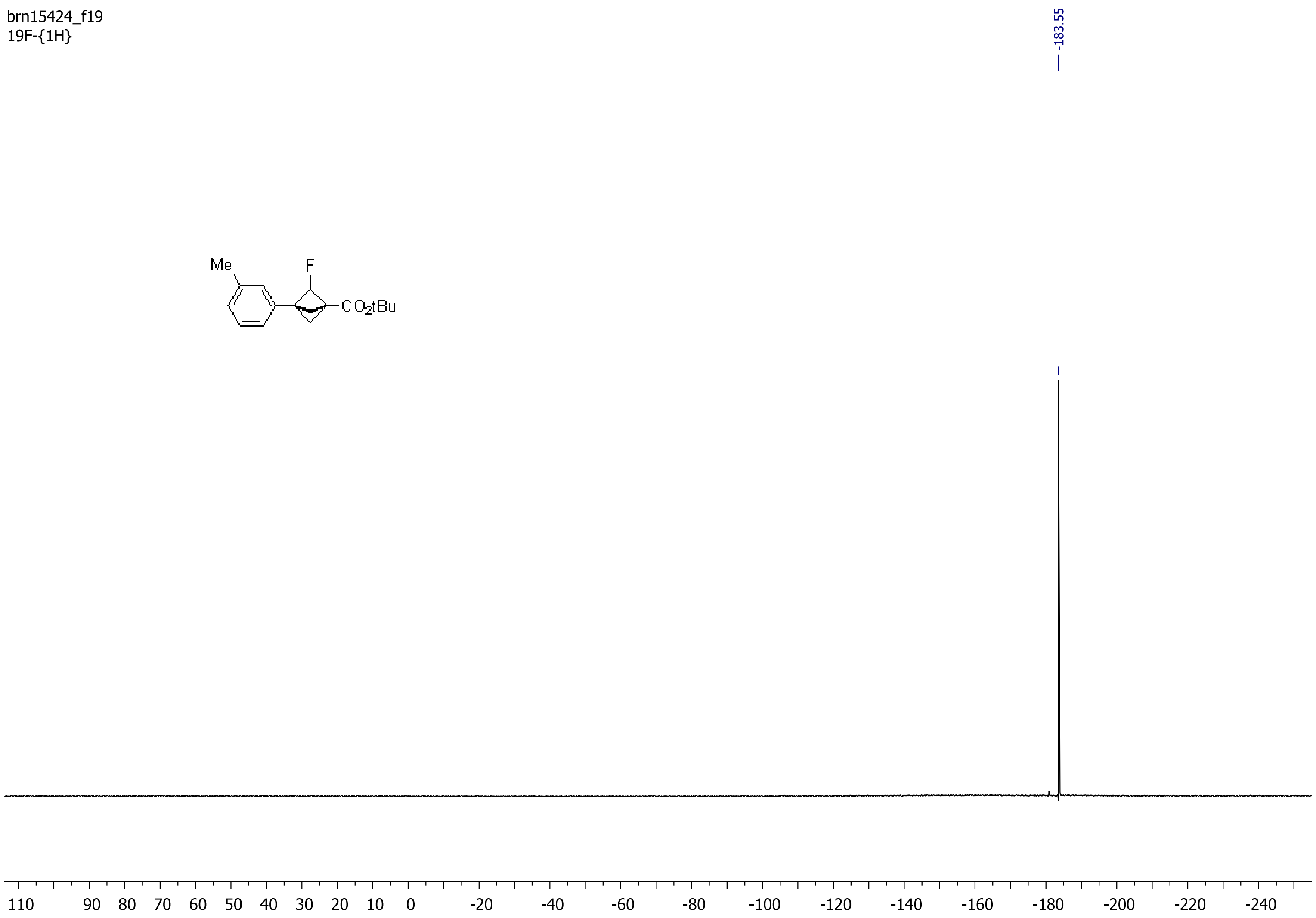

**Compound 15b**

$^{1}H$ NMR (400 MHz, $CDCl_3$)

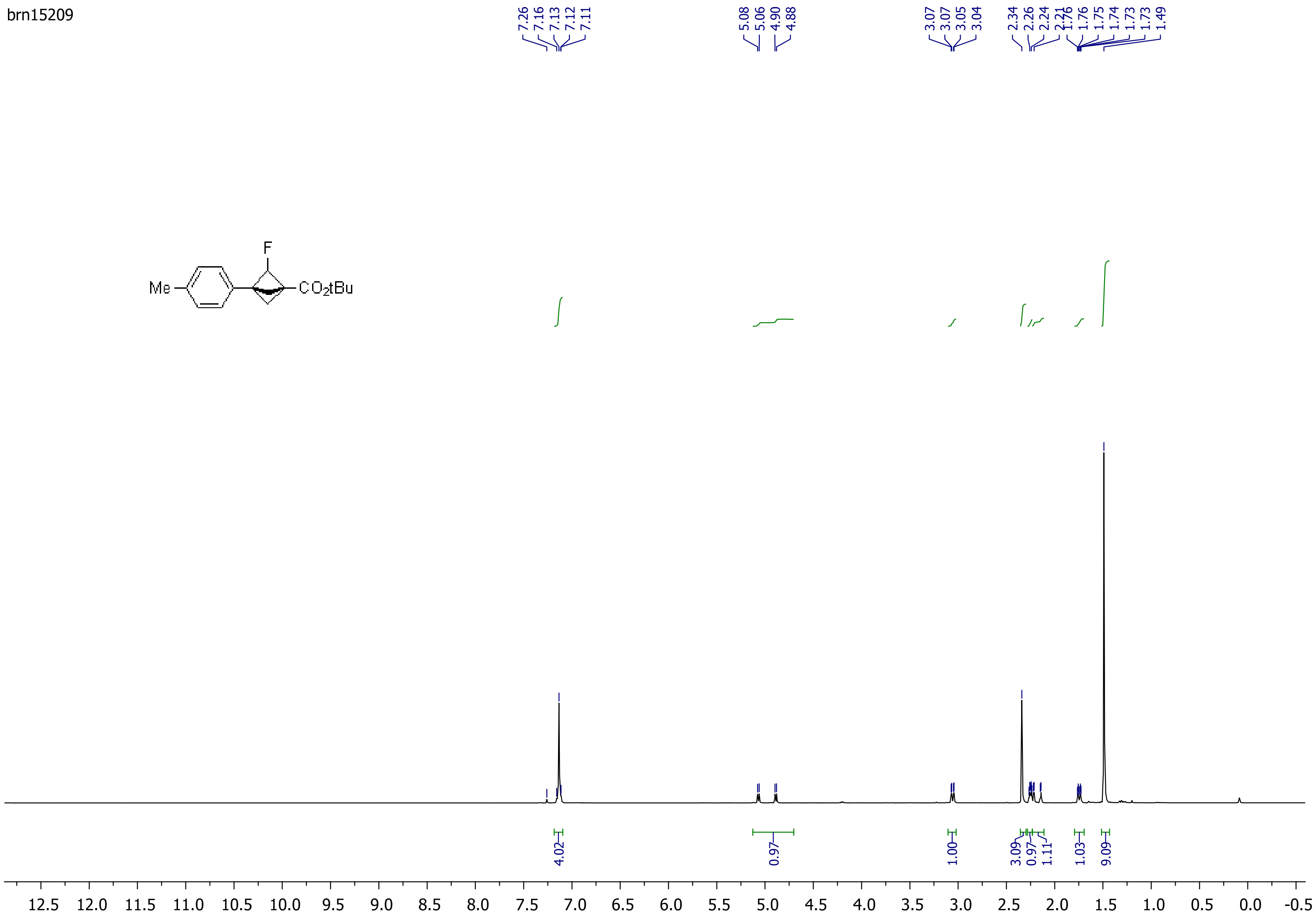

$^{13}C\{^{1}H\}$ NMR (151 MHz, $CDCl_3$)

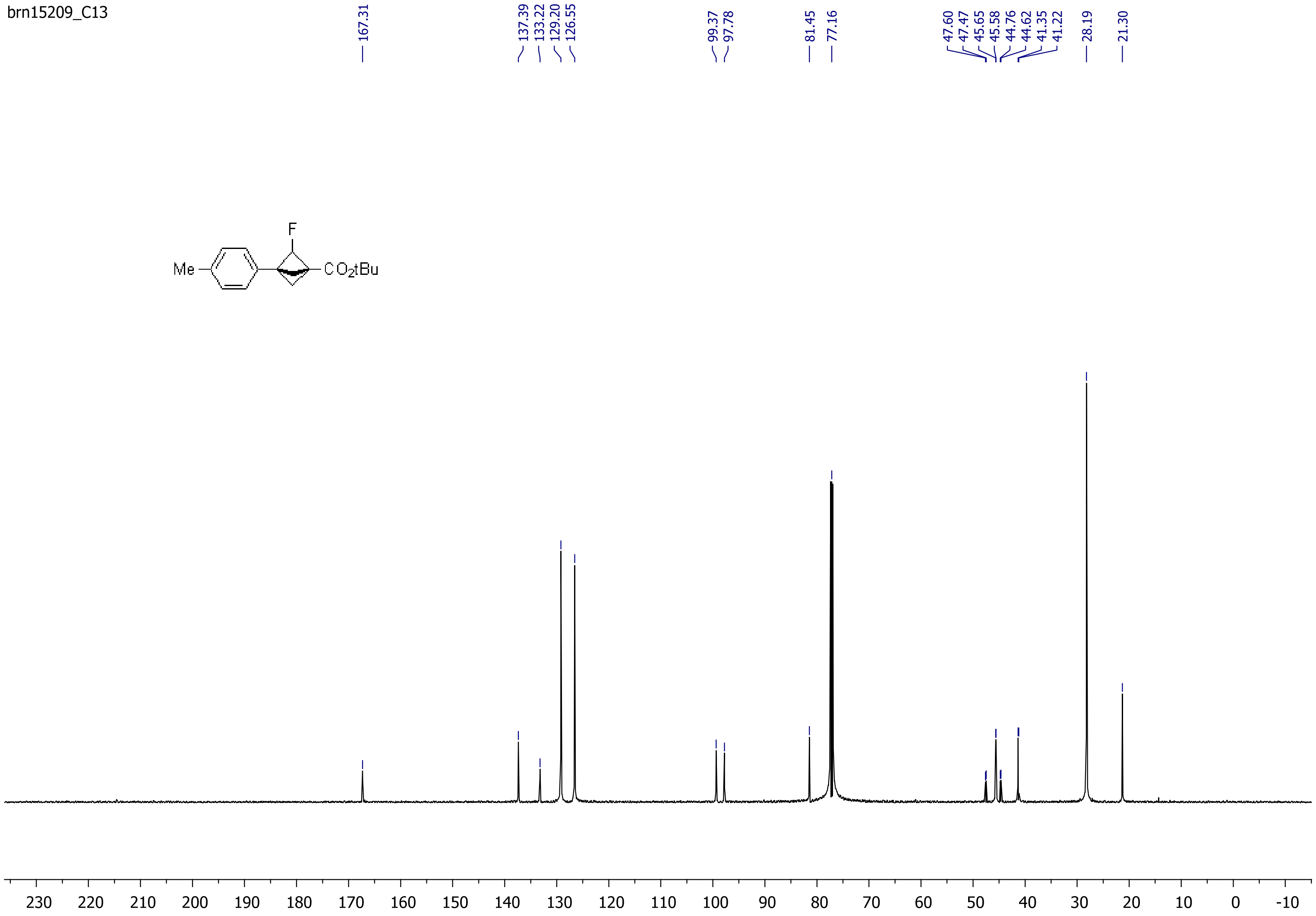

## $^{19}F\{^{1}H\}$ NMR (376 MHz, $CDCl_3$)

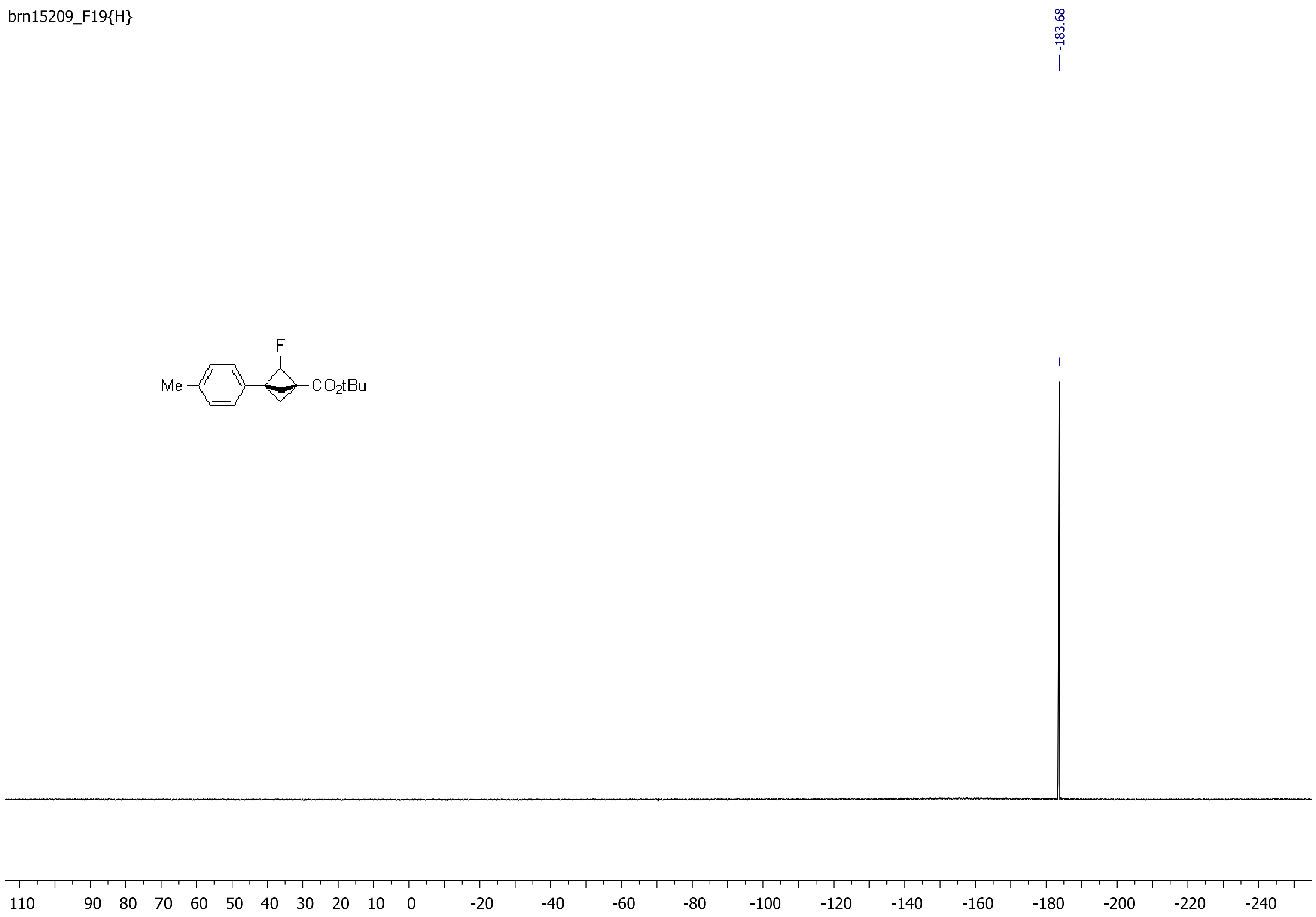

**Compound 16b**

$^{1}$H NMR (400 MHz, $CDCl_3$)

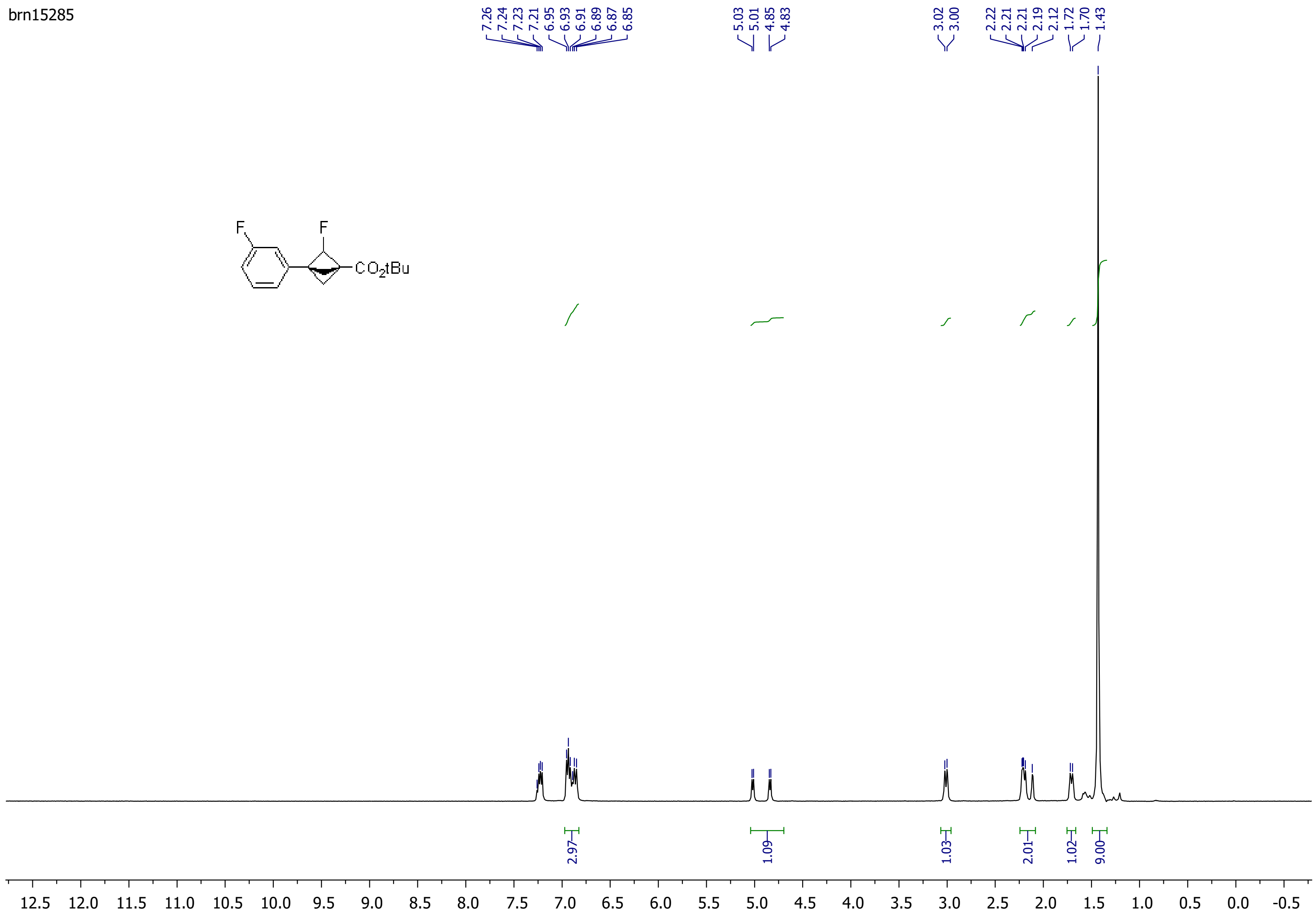

$^{13}C\{^{1}H\}$ NMR (151 MHz, $CDCl_3$)

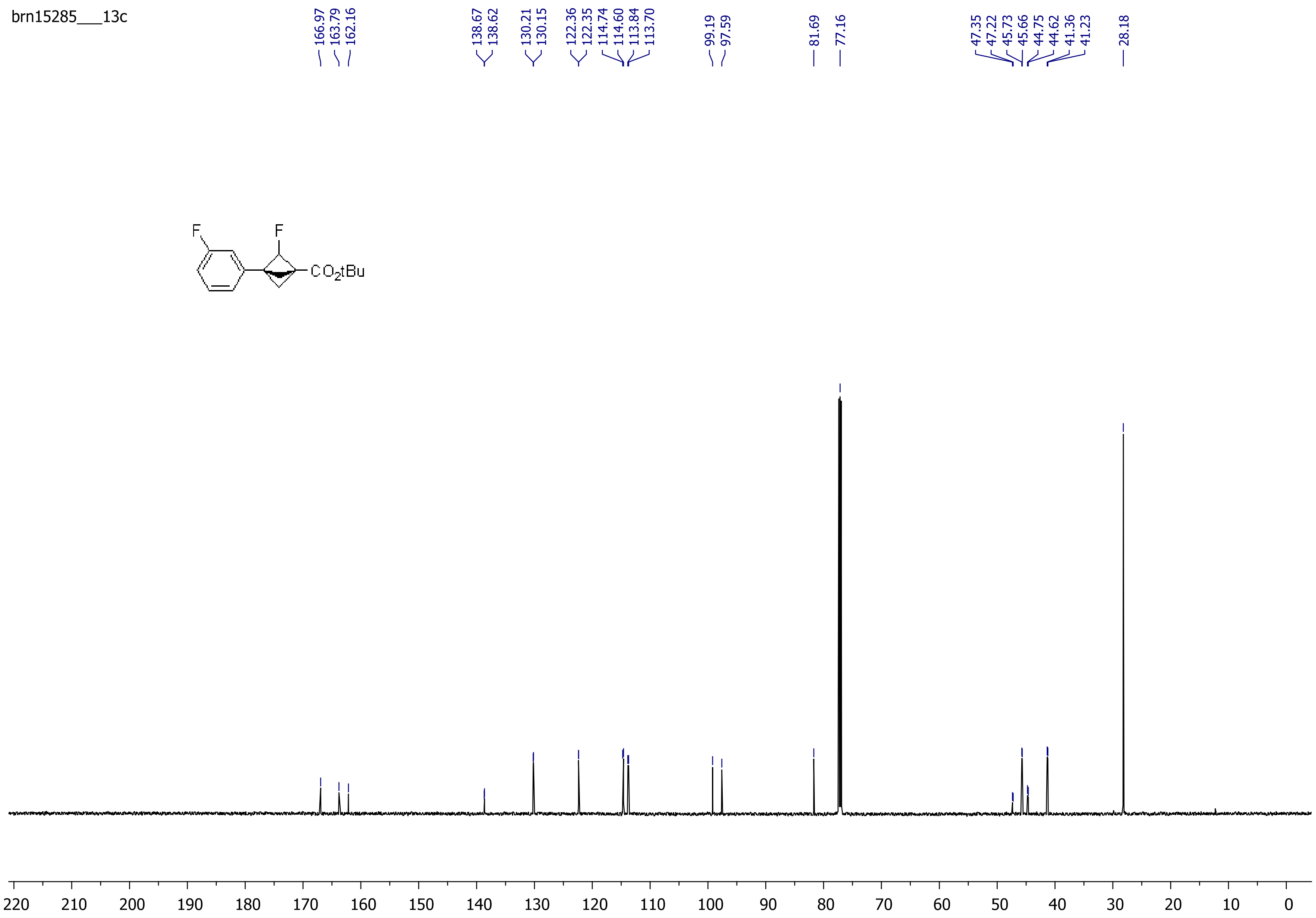

$^{19}F\{^1H\}$ NMR (376 MHz, $CDCl_3$)

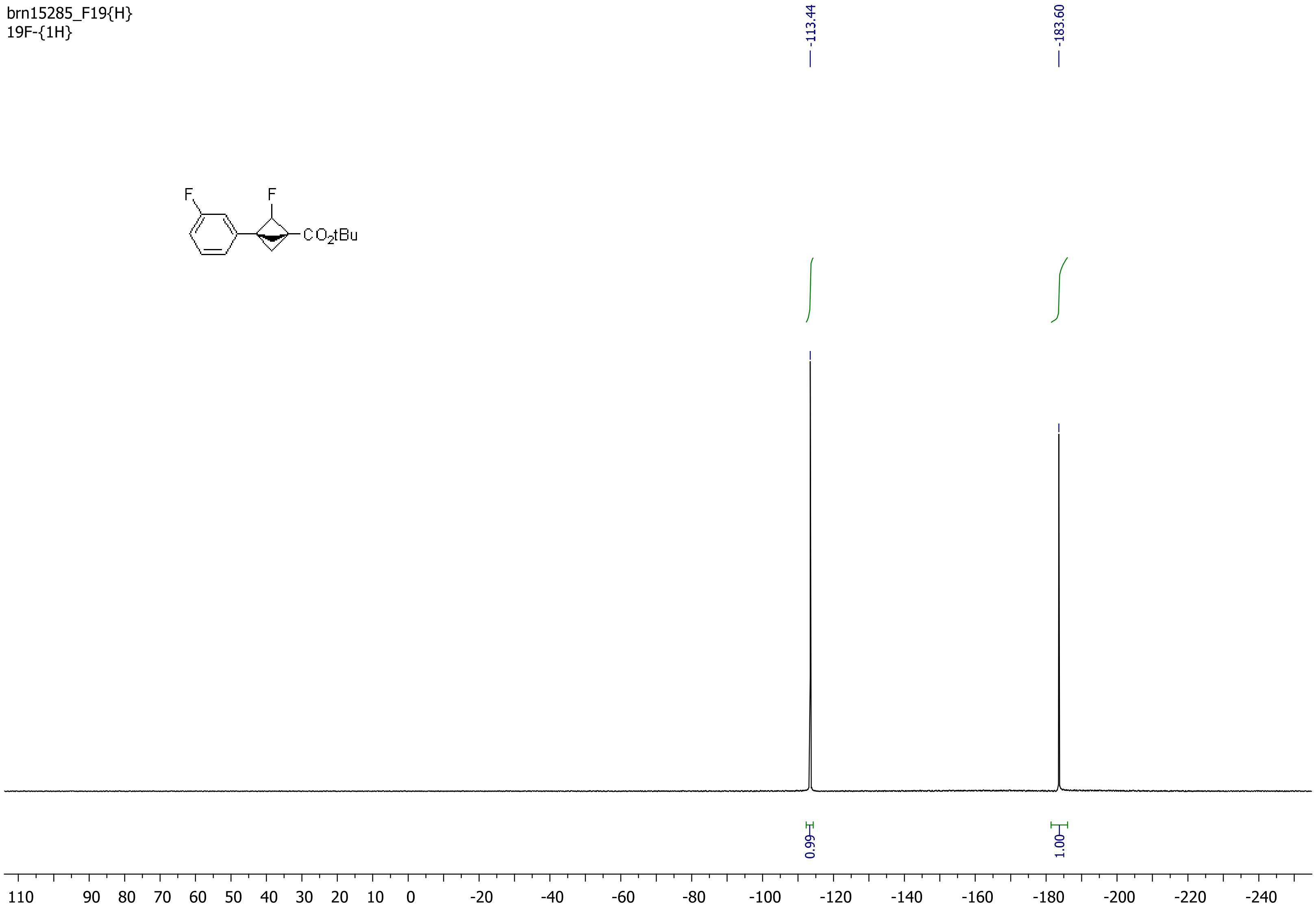

**Compound 17b**

$^1$H NMR (400 MHz, $CDCl_3$)

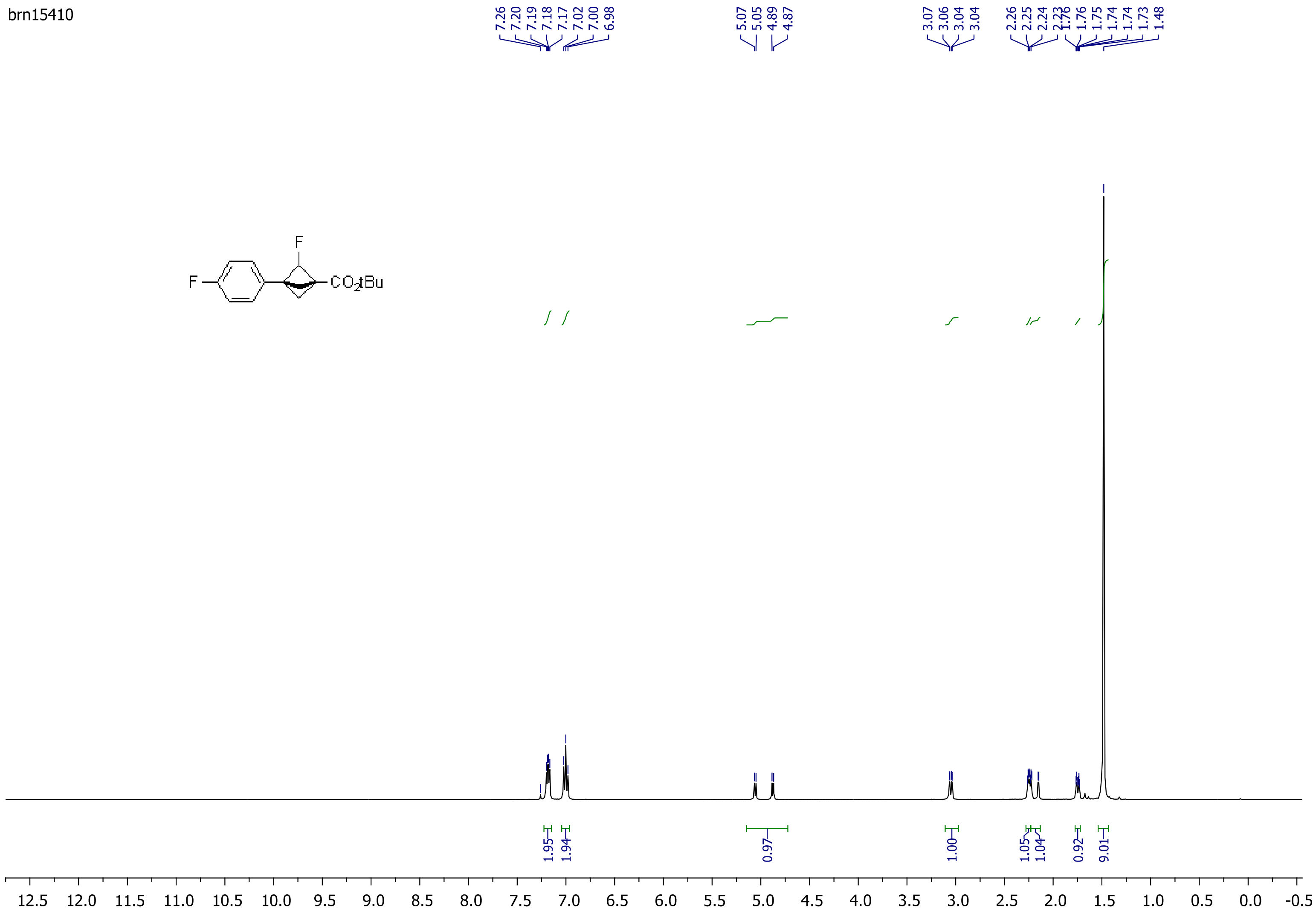

$^{13}C\{^{1}H\}$ NMR (151 MHz, $CDCl_3$)

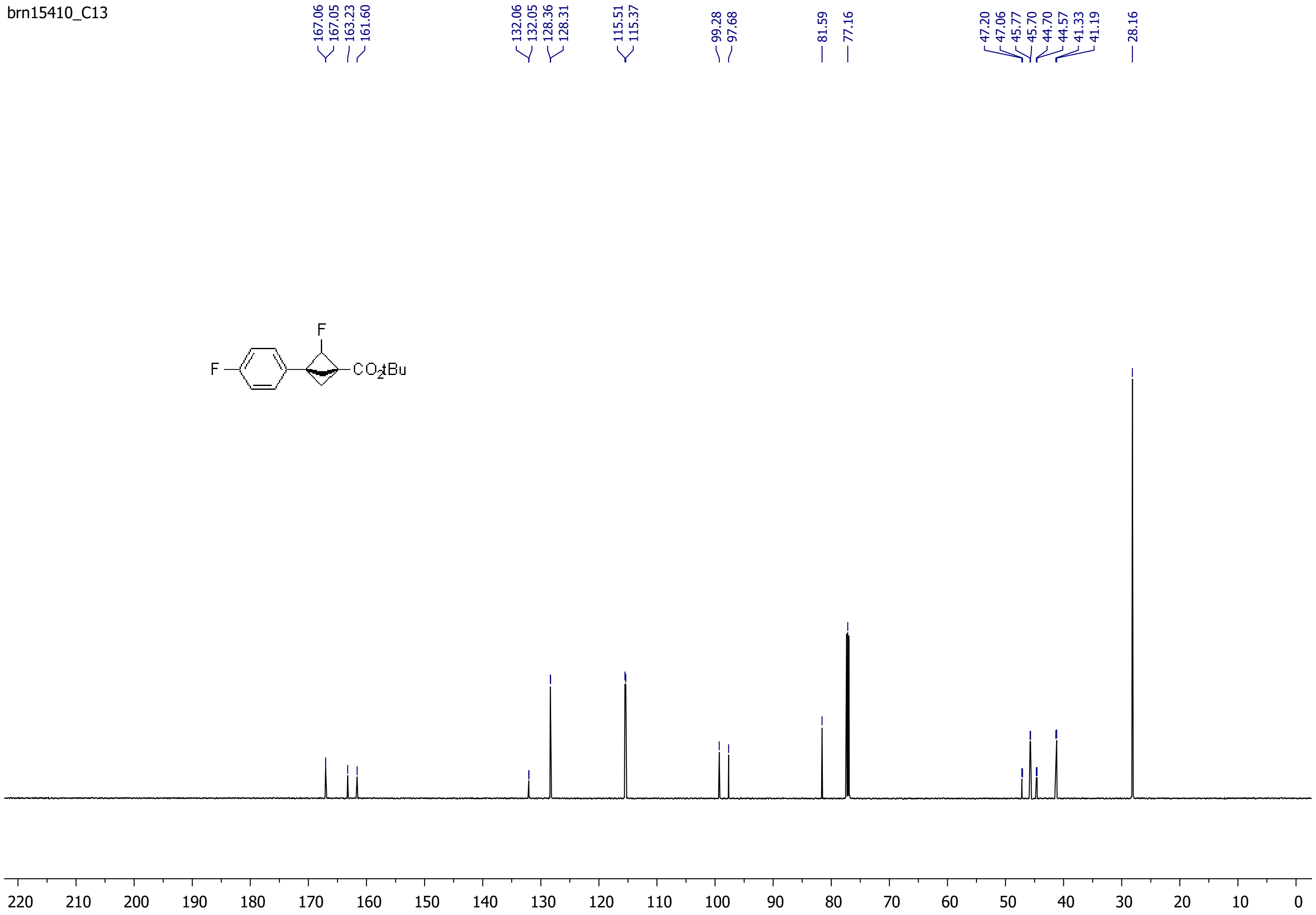

$^{19}F\{^{1}H\}$ NMR (376 MHz, $CDCl_3$)

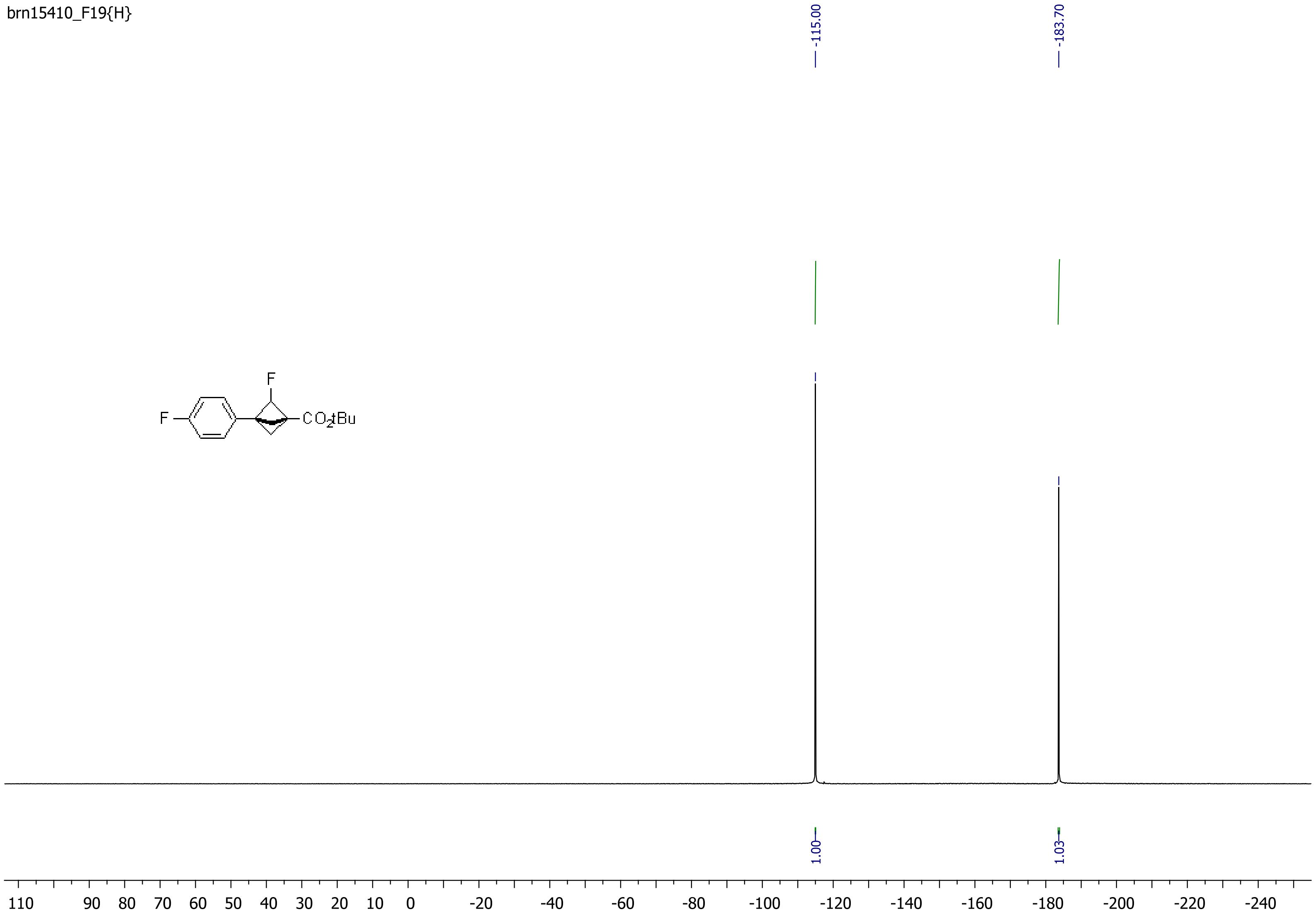

**Compound 18b**

$^{1}H$ NMR (400 MHz, $CDCl_3$)

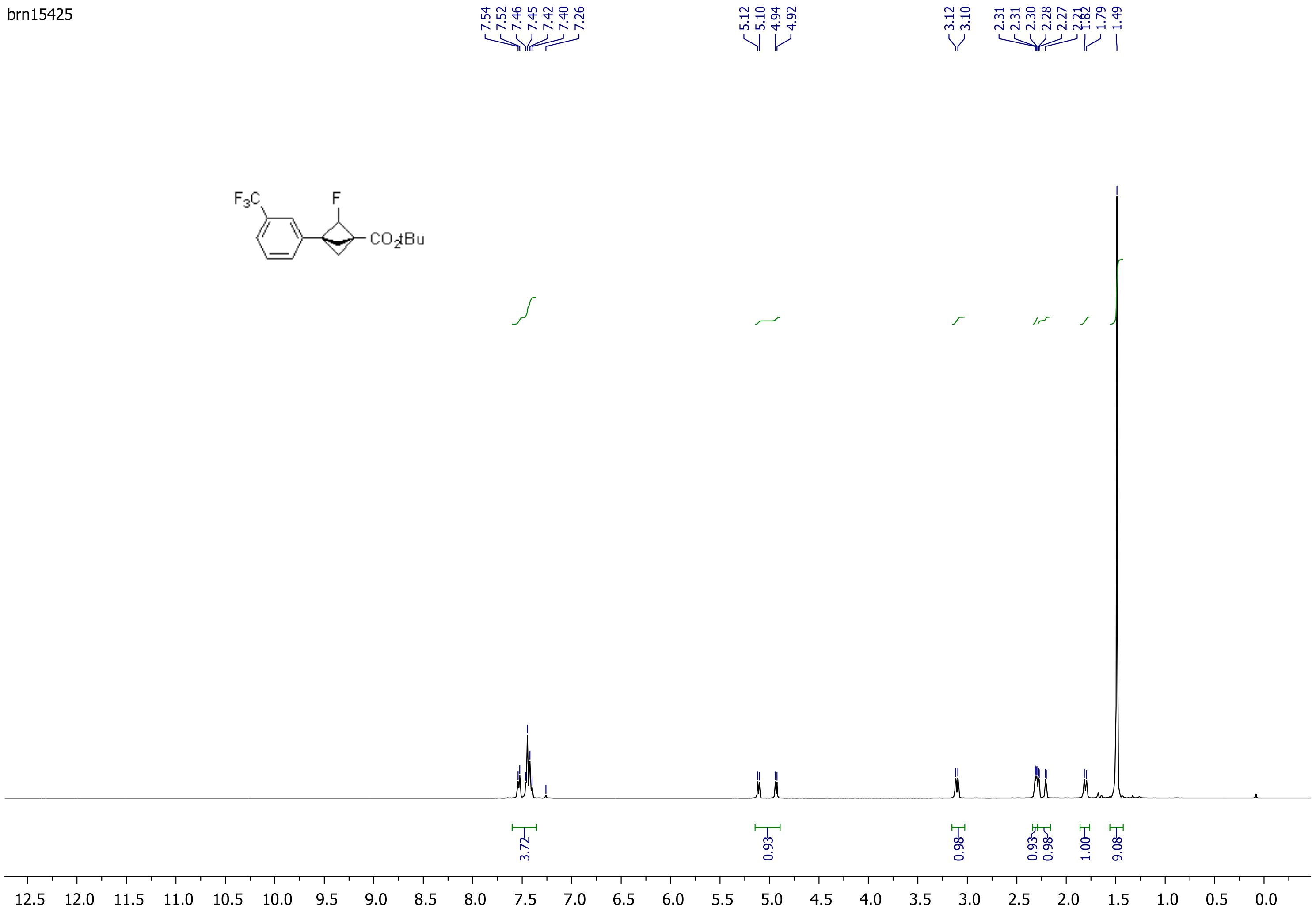

$^{13}C\{^{1}H\}$ NMR (126 MHz, $CDCl_3$)

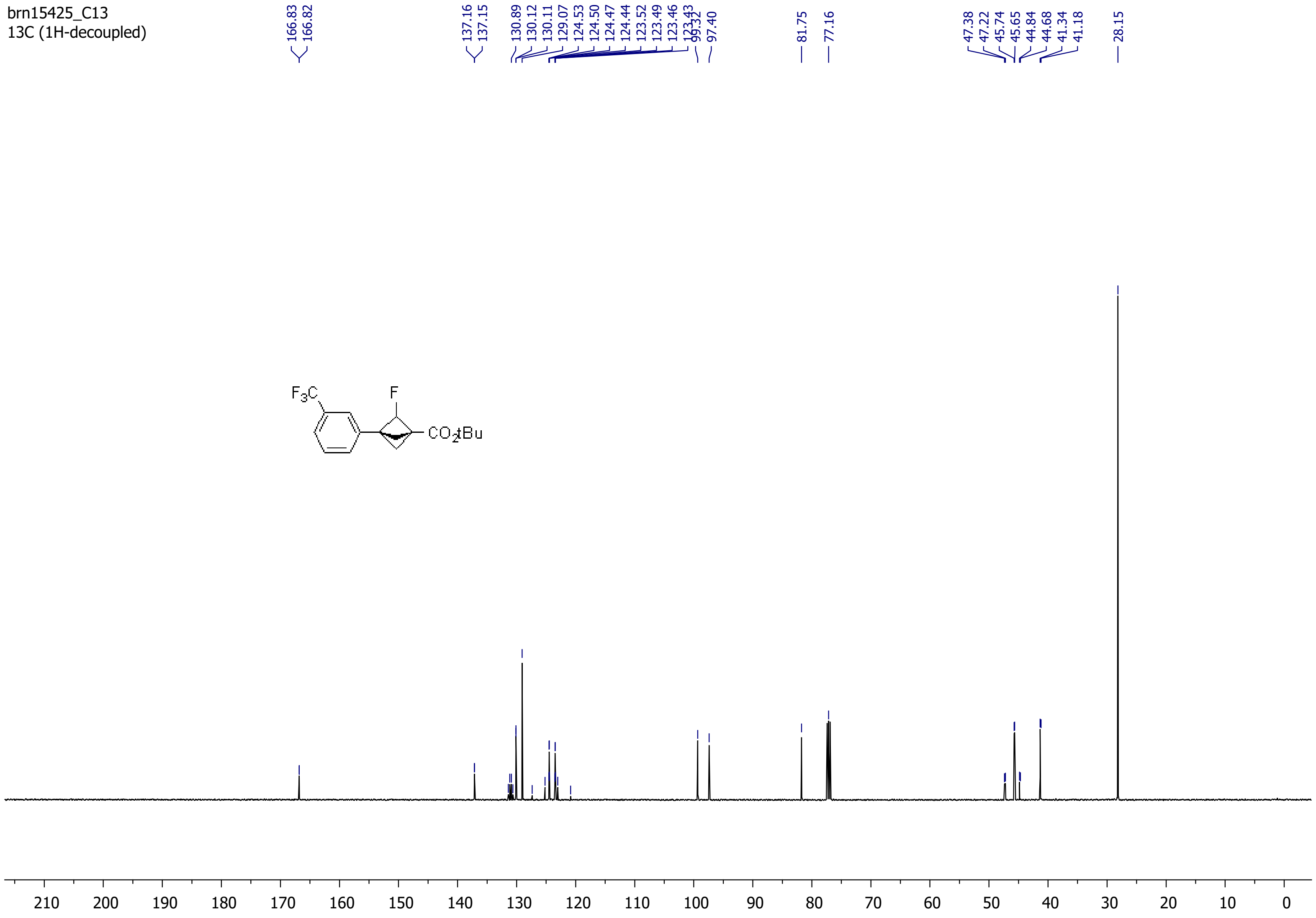

$^{19}F\{^1H\}$ NMR (376 MHz, $CDCl_3$)

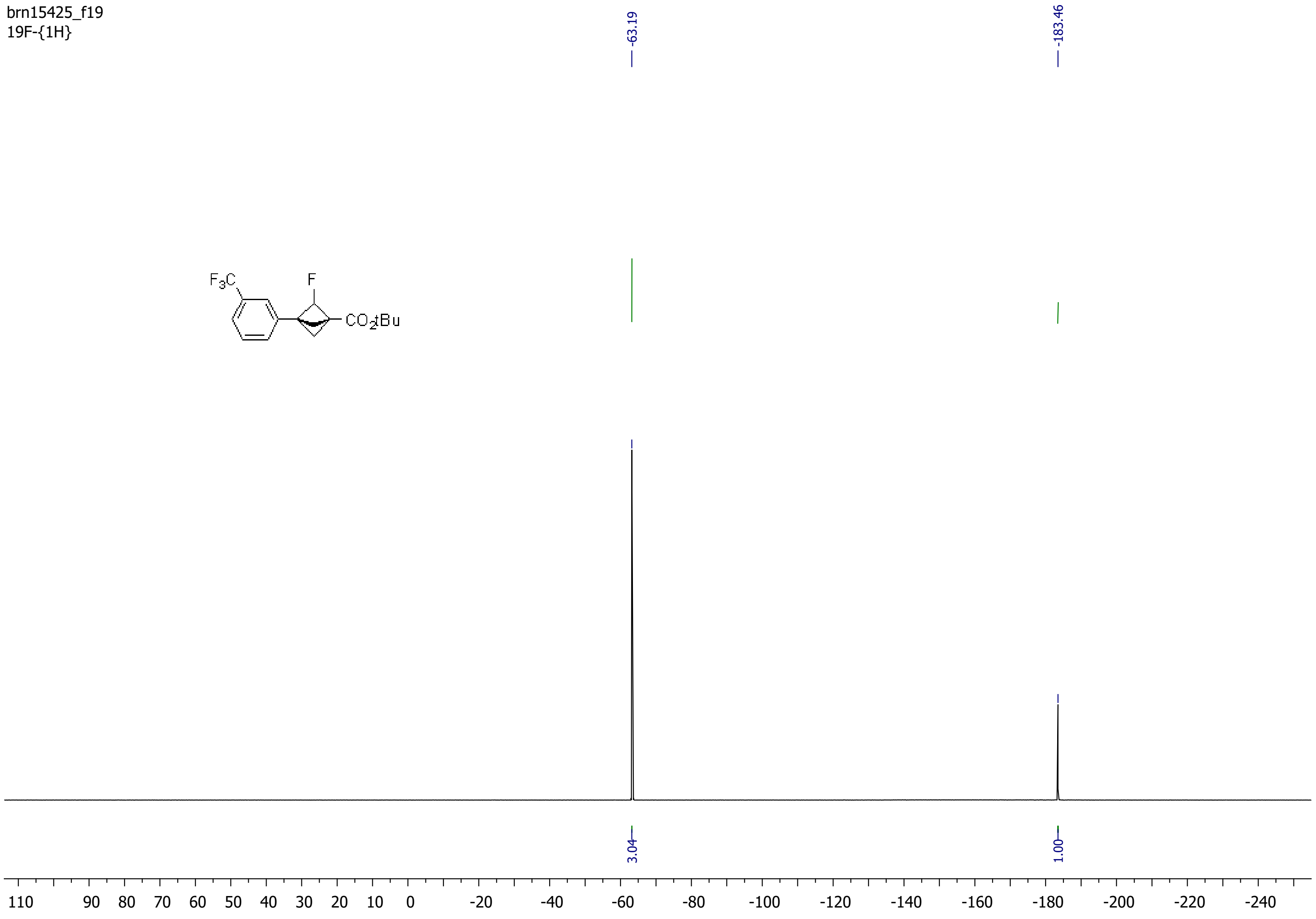

**Compound 19b**

$^{1}$H NMR (400 MHz, $CDCl_3$)

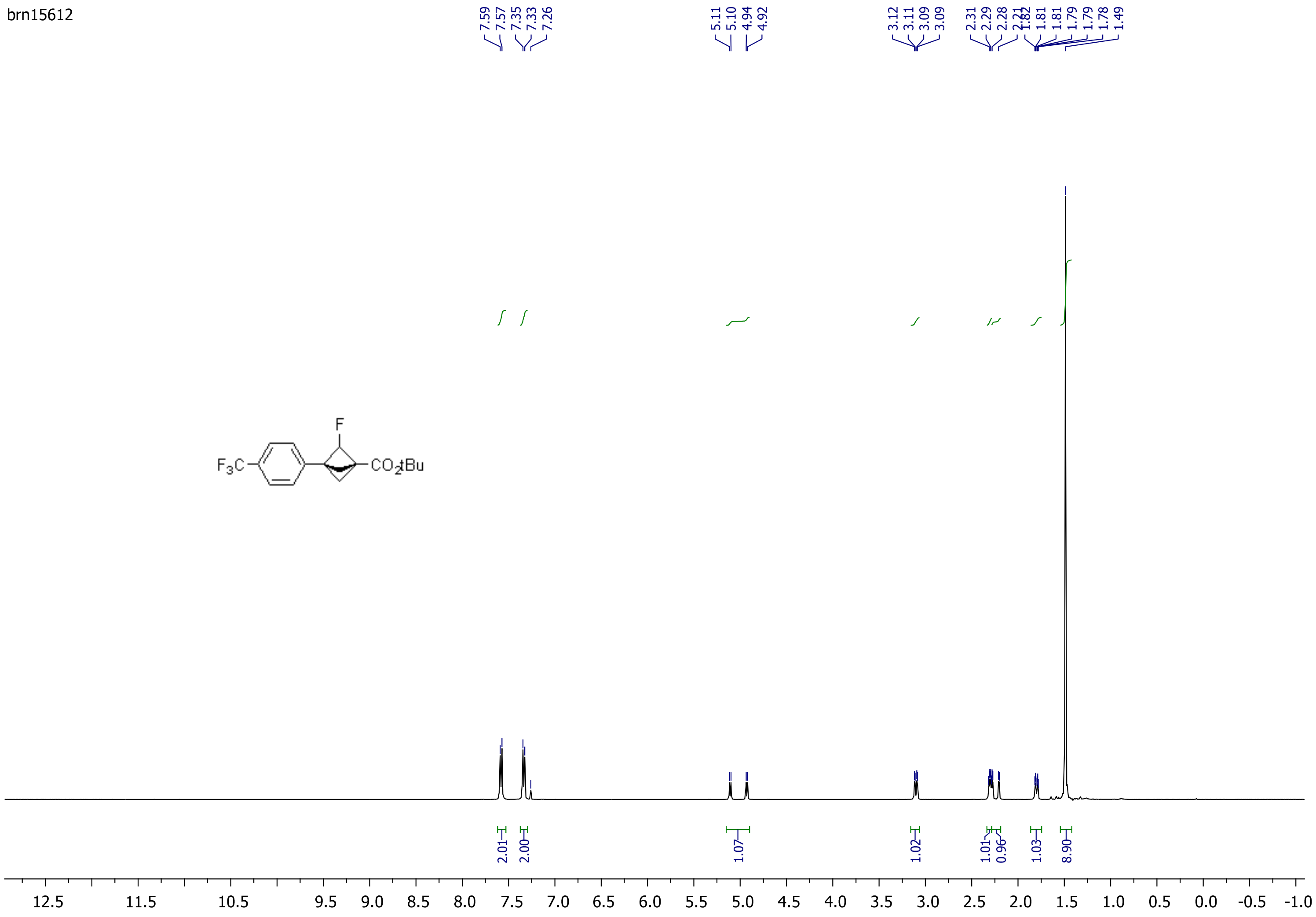

$^{13}C\{^{1}H\}$ NMR (126 MHz, $CDCl_3$)

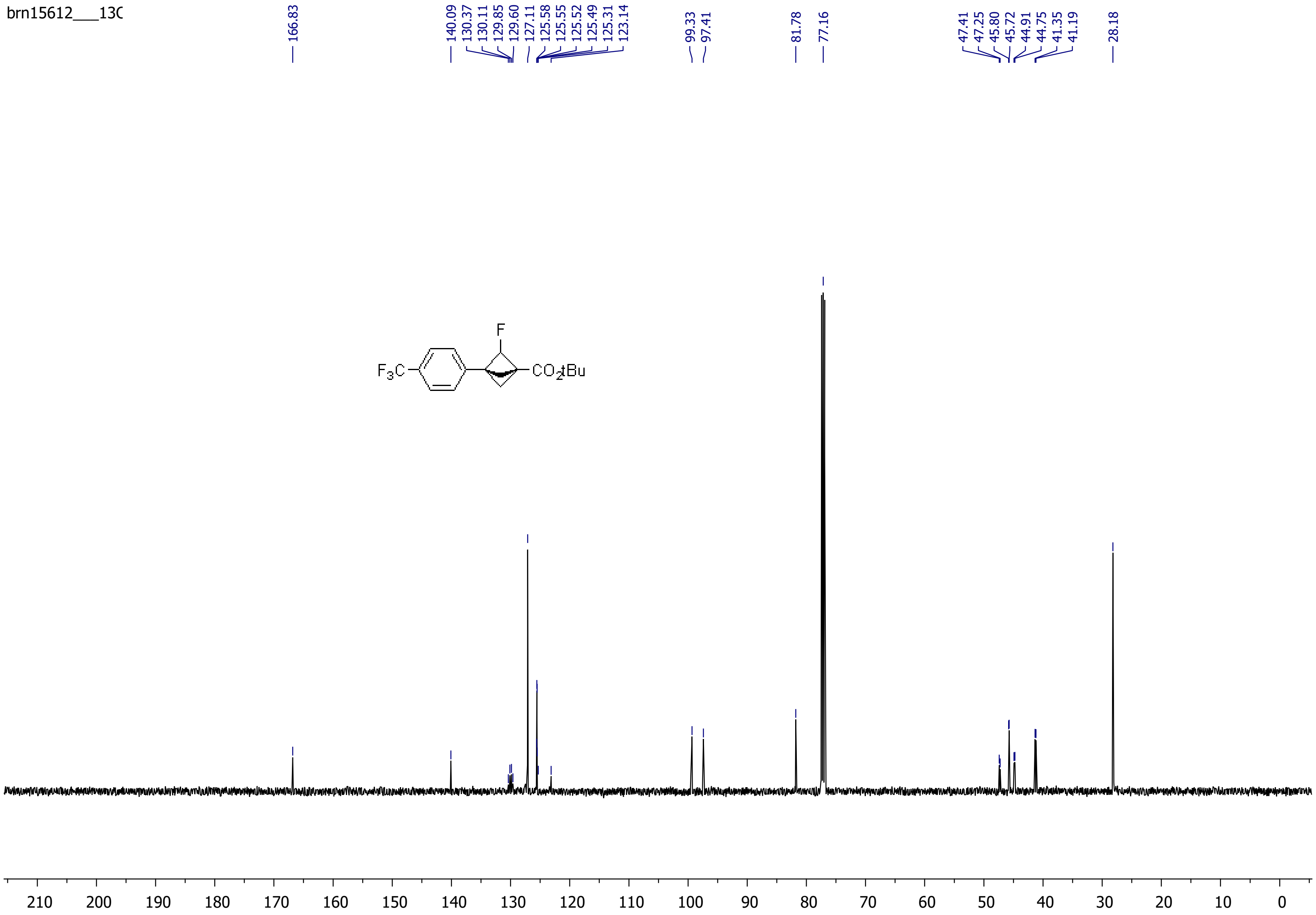

$^{19}F\{^{1}H\}$ NMR (376 MHz, $CDCl_3$)

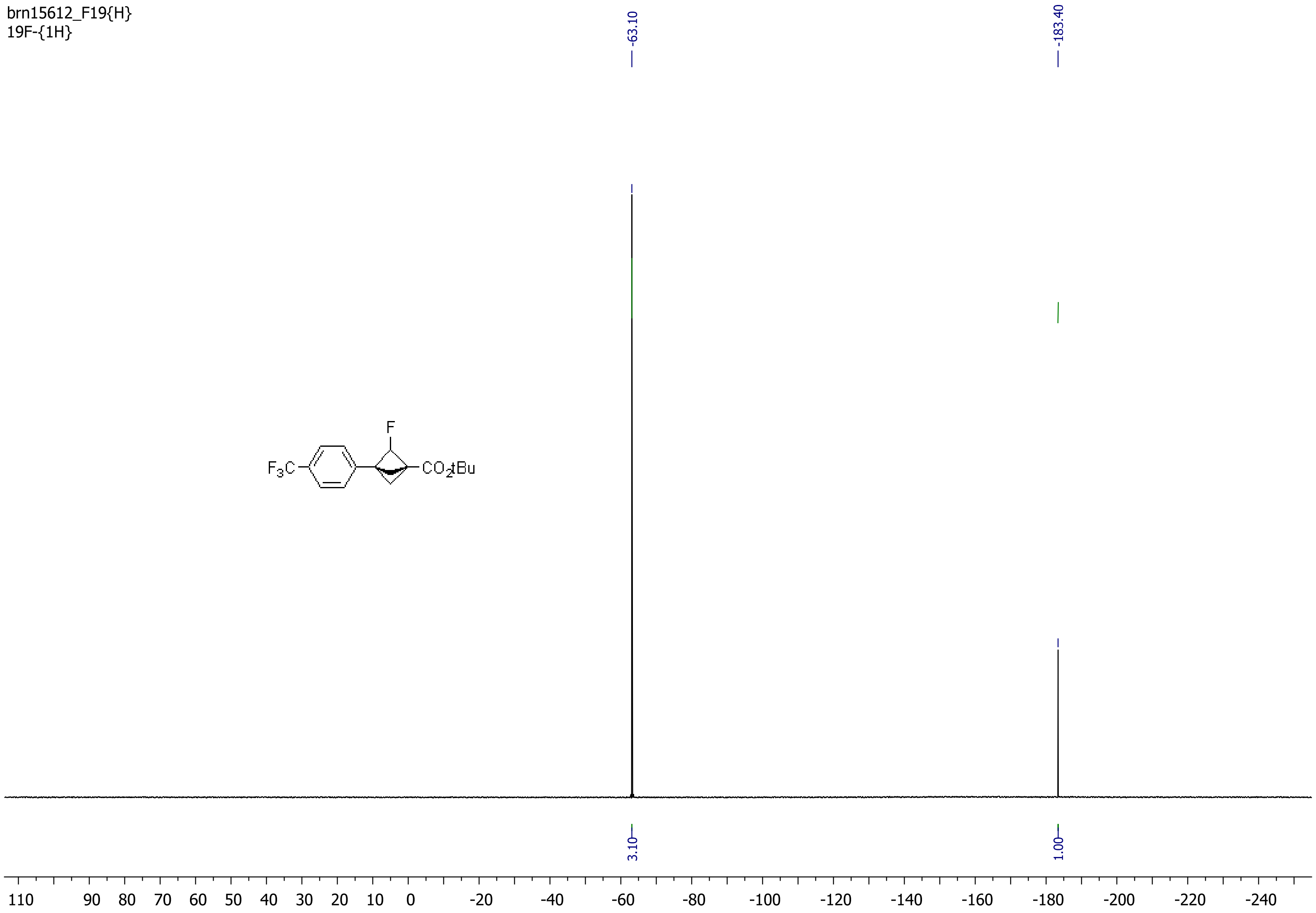

**Compound 12**

$^{1}H$ NMR (500 MHz, $CDCl_3$)

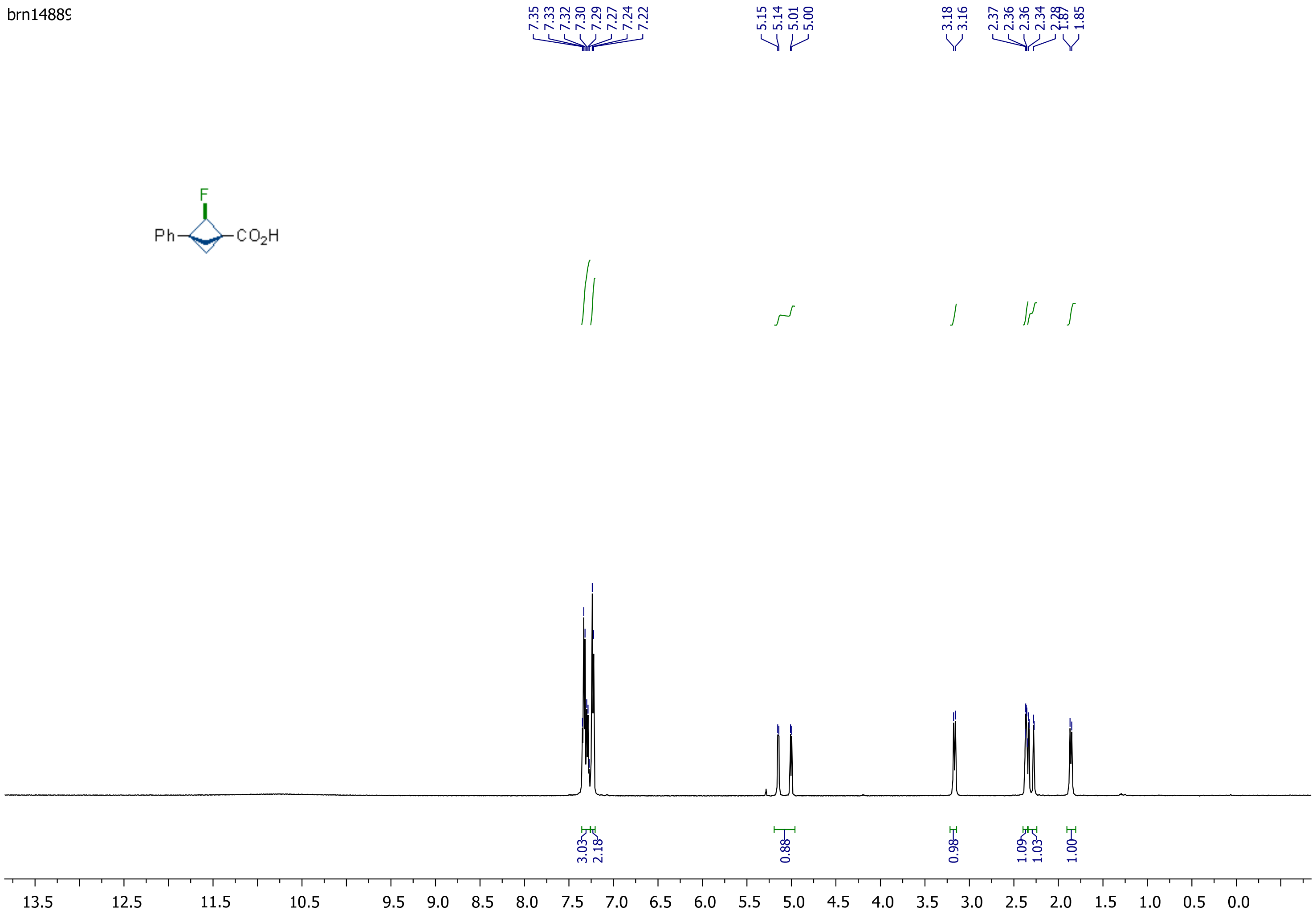

$^{13}C\{^1H\}$ NMR (126 MHz, $CDCl_3$)

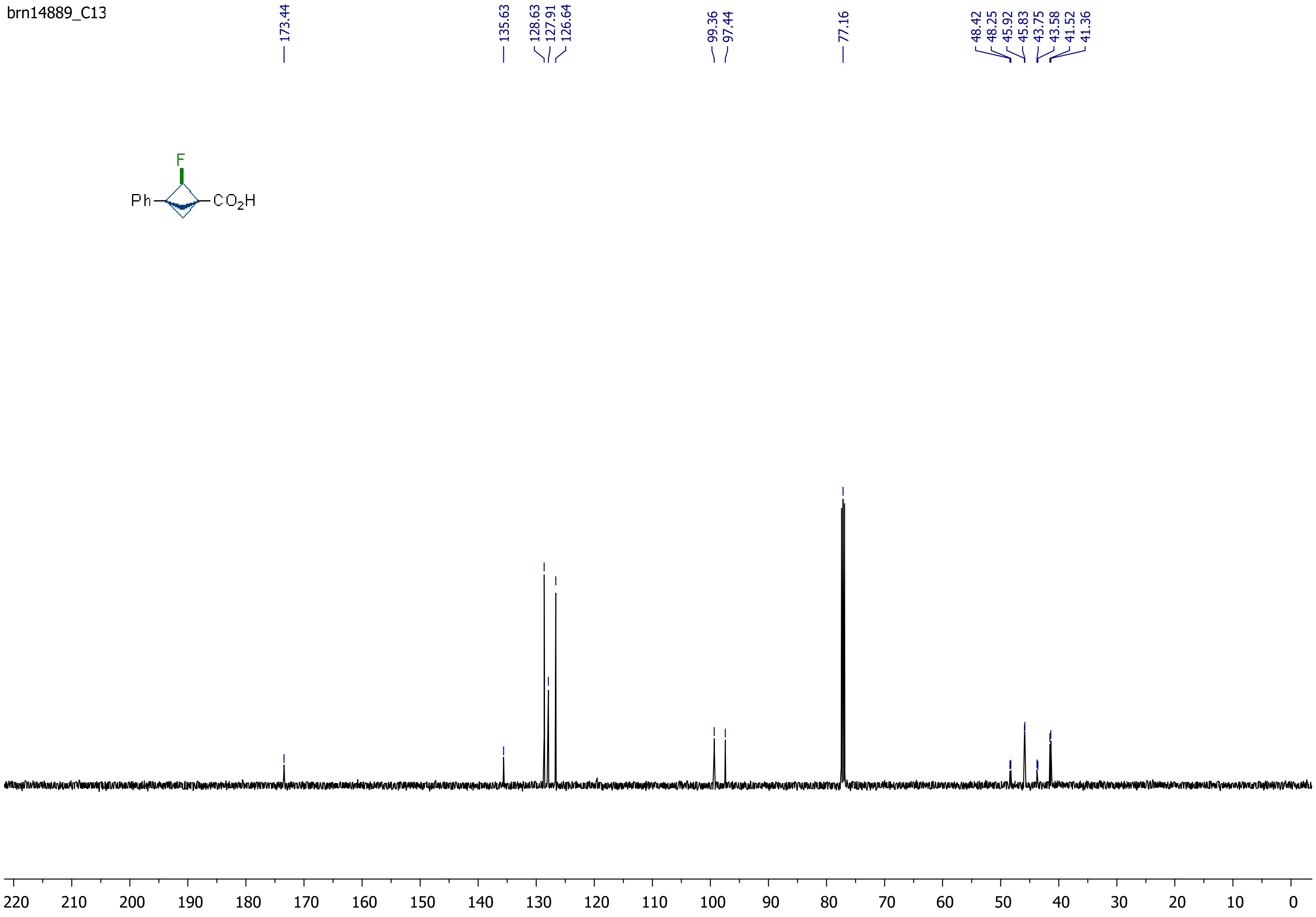

$^{19}F\{^{1}H\}$ NMR (470 MHz, $CDCl_3$)

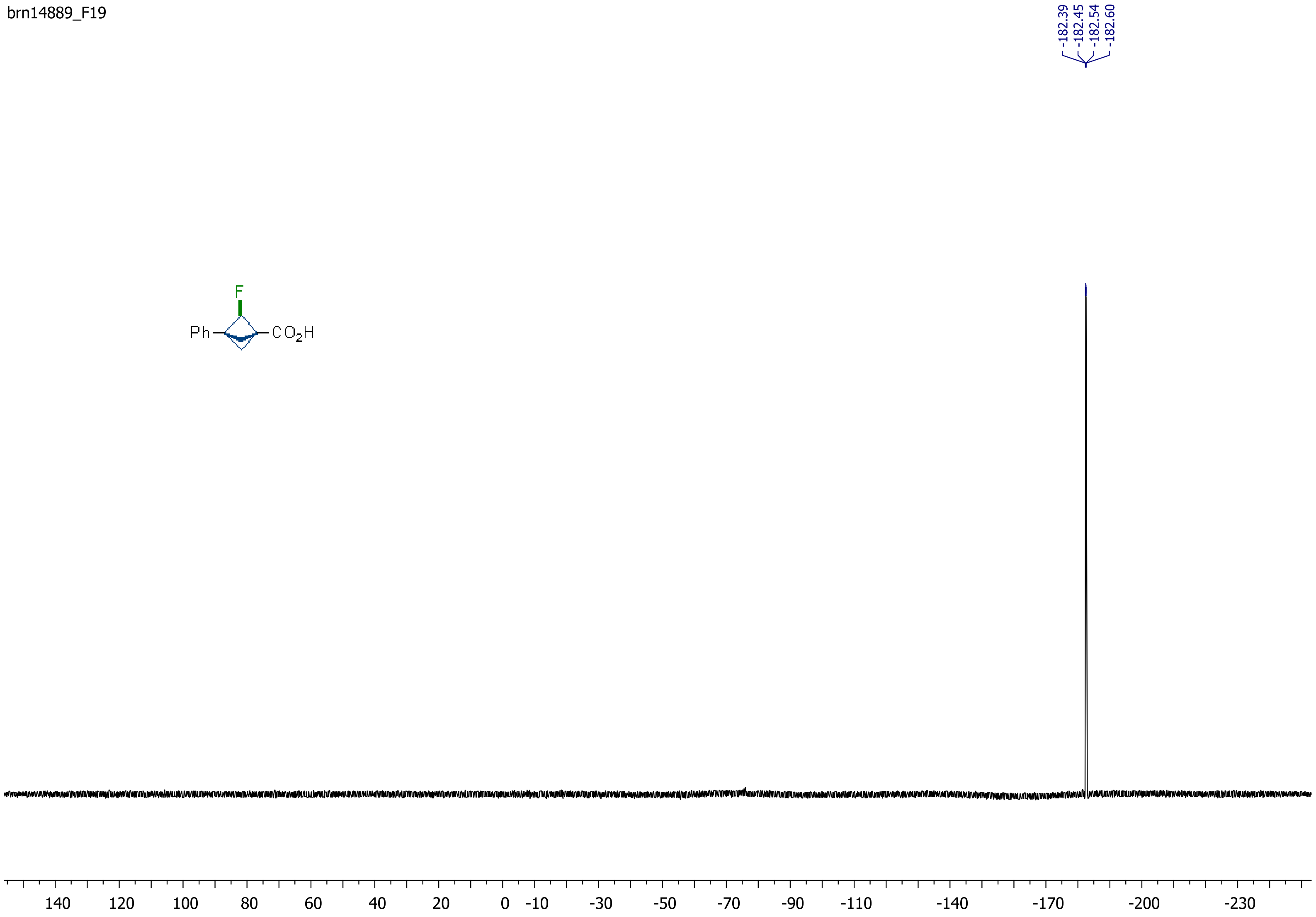

**Compound 13c**

$^{1}$H NMR (500 MHz, $CDCl_3$)

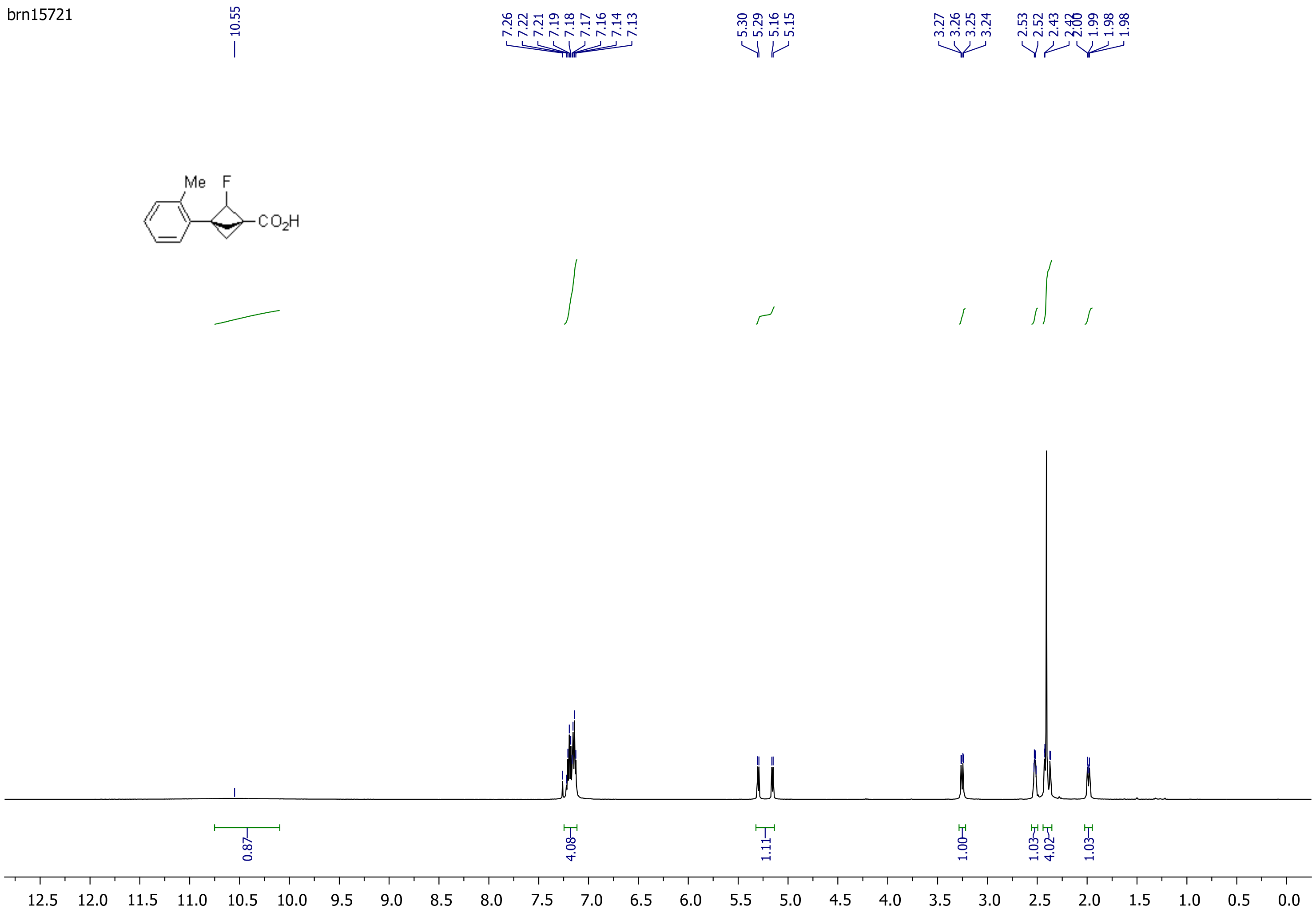

$^{13}C\{^{1}H\}$ NMR (126 MHz, $CDCl_3$)

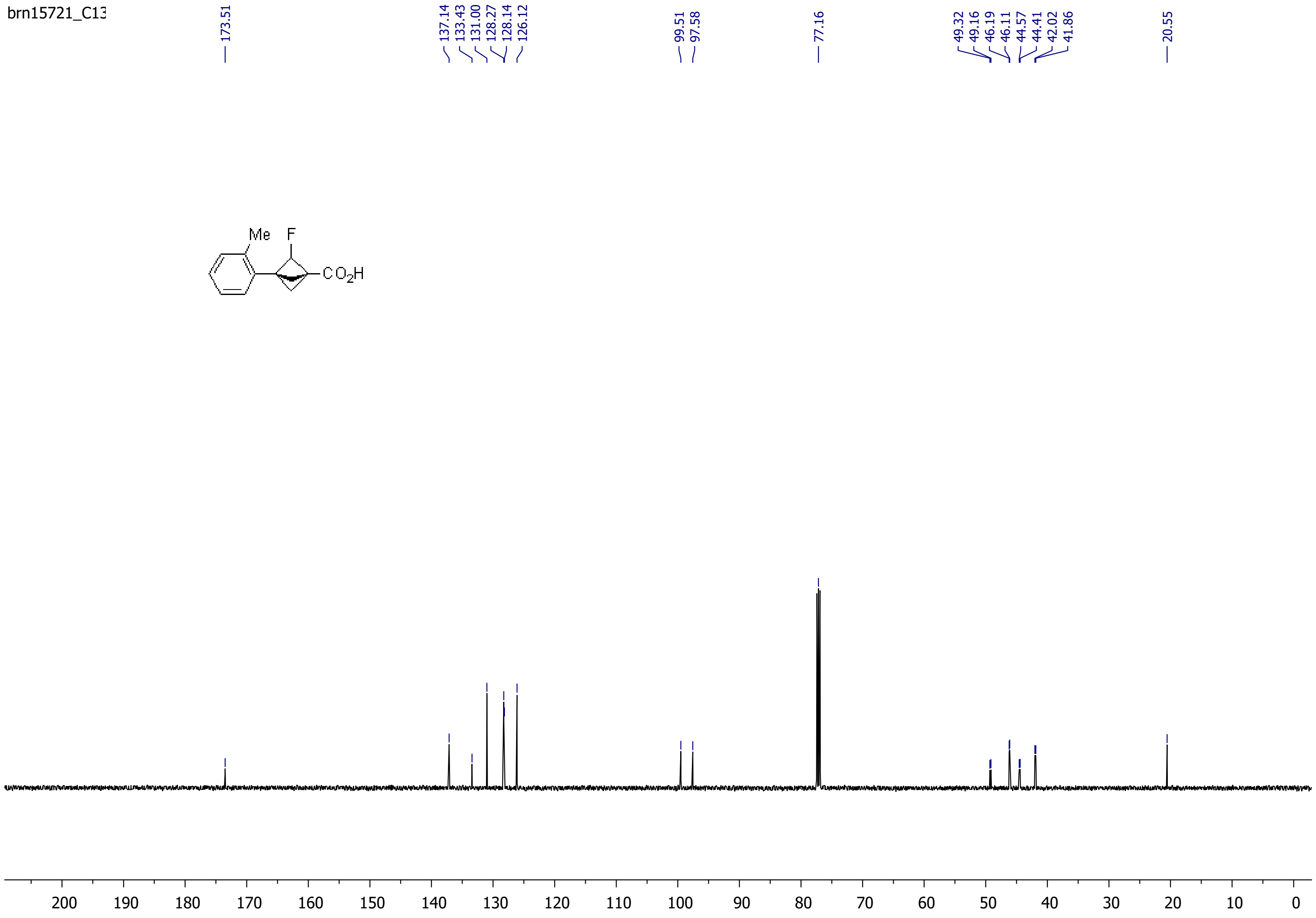

$^{19}F\{^1H\}$ NMR (376 MHz, $CDCl_3$)

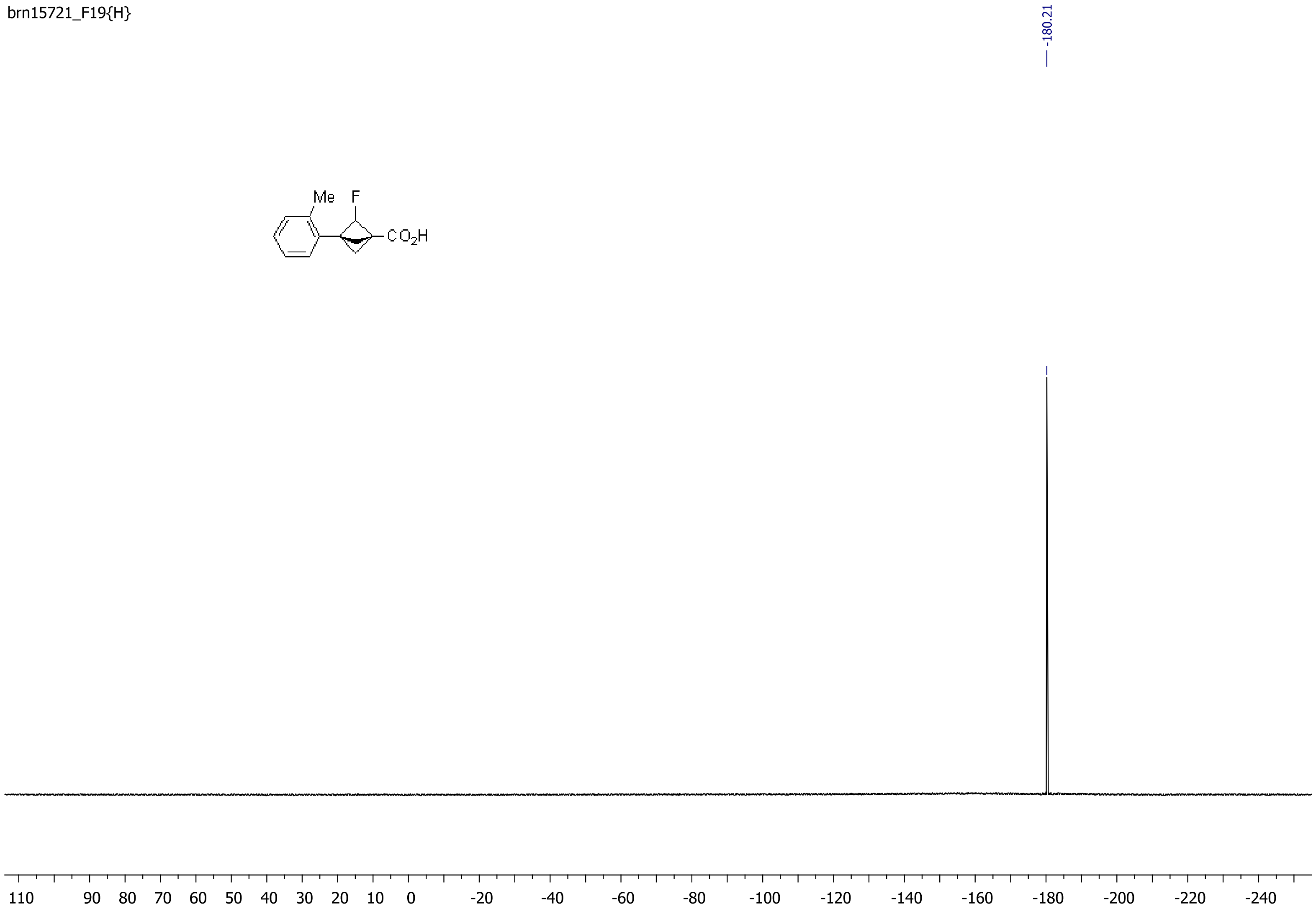

**Compound 14c**

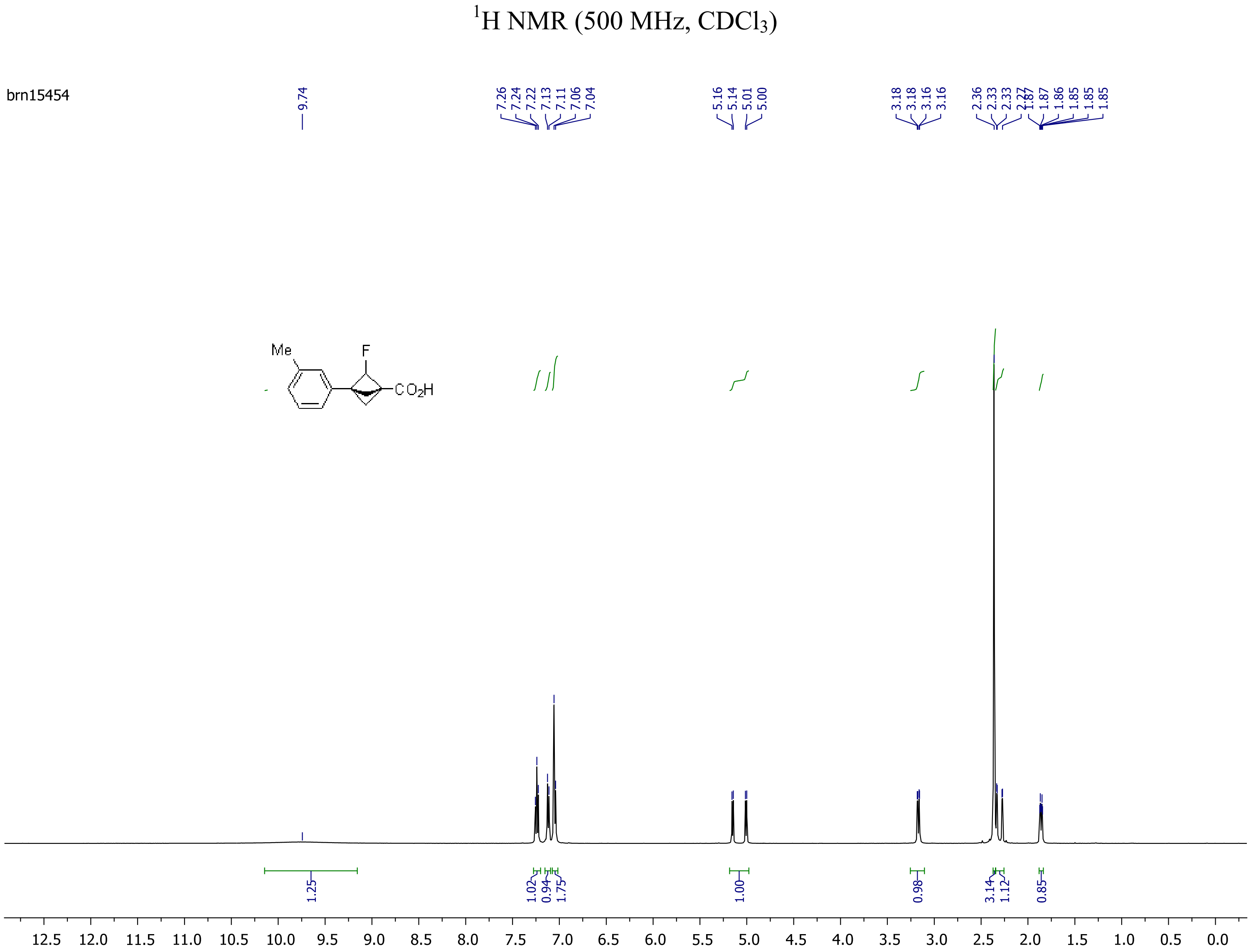

## $^{13}C\{^{1}H\}$ NMR (126 MHz, $CDCl_3$)

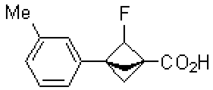

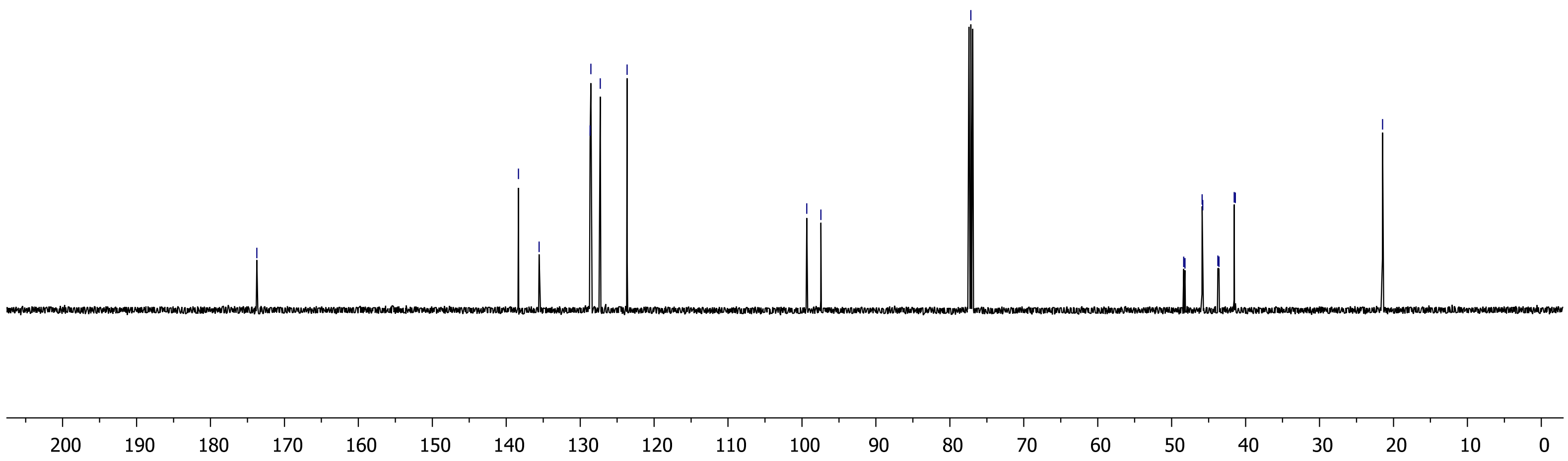

$^{19}F\{^{1}H\}$ NMR (376 MHz, $CDCl_3$)

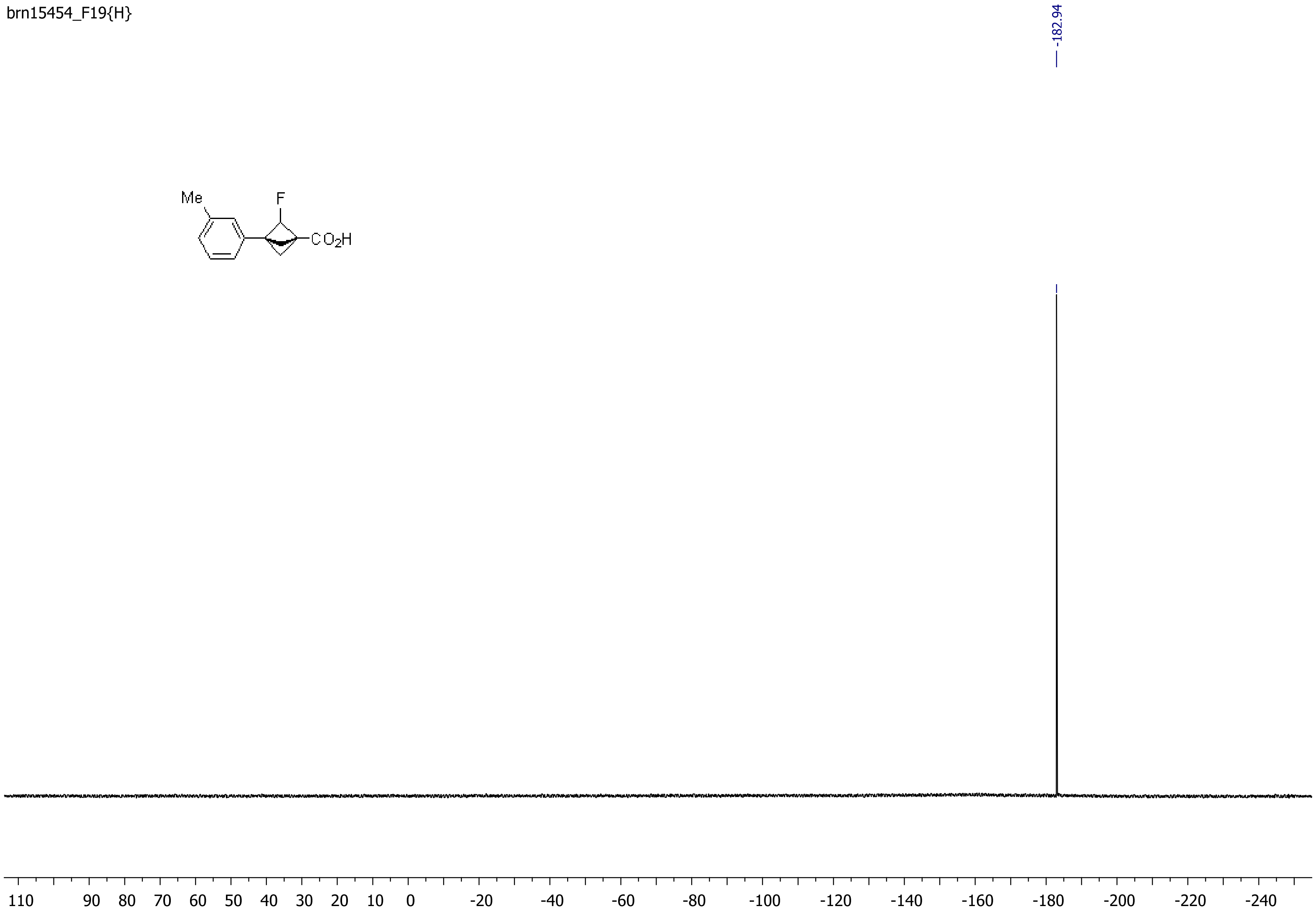

**Compound 15c**

$^{1}H$ NMR (500 MHz, $CDCl_3$)

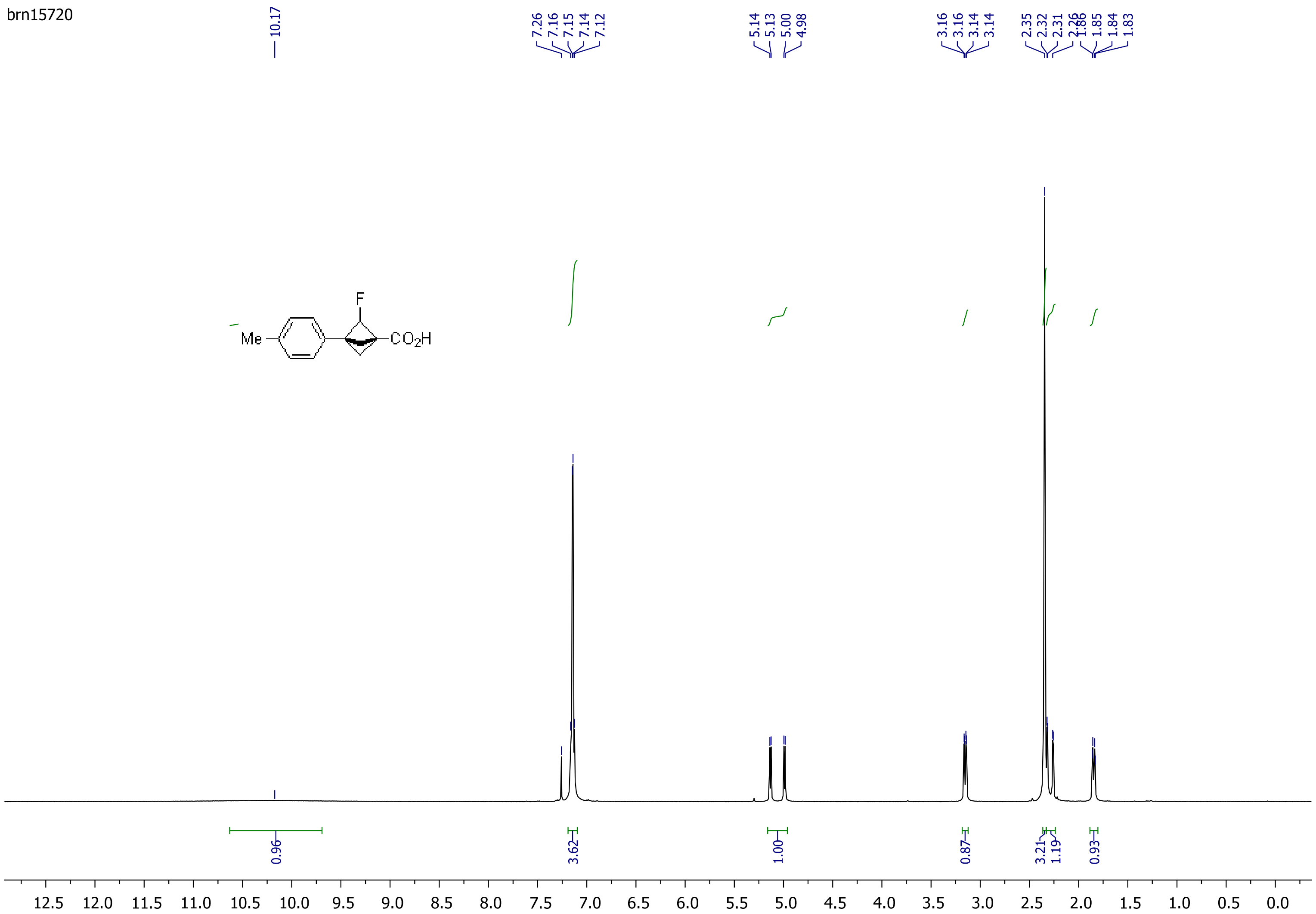

$^{13}C\{^{1}H\}$ NMR (126 MHz, $CDCl_3$)

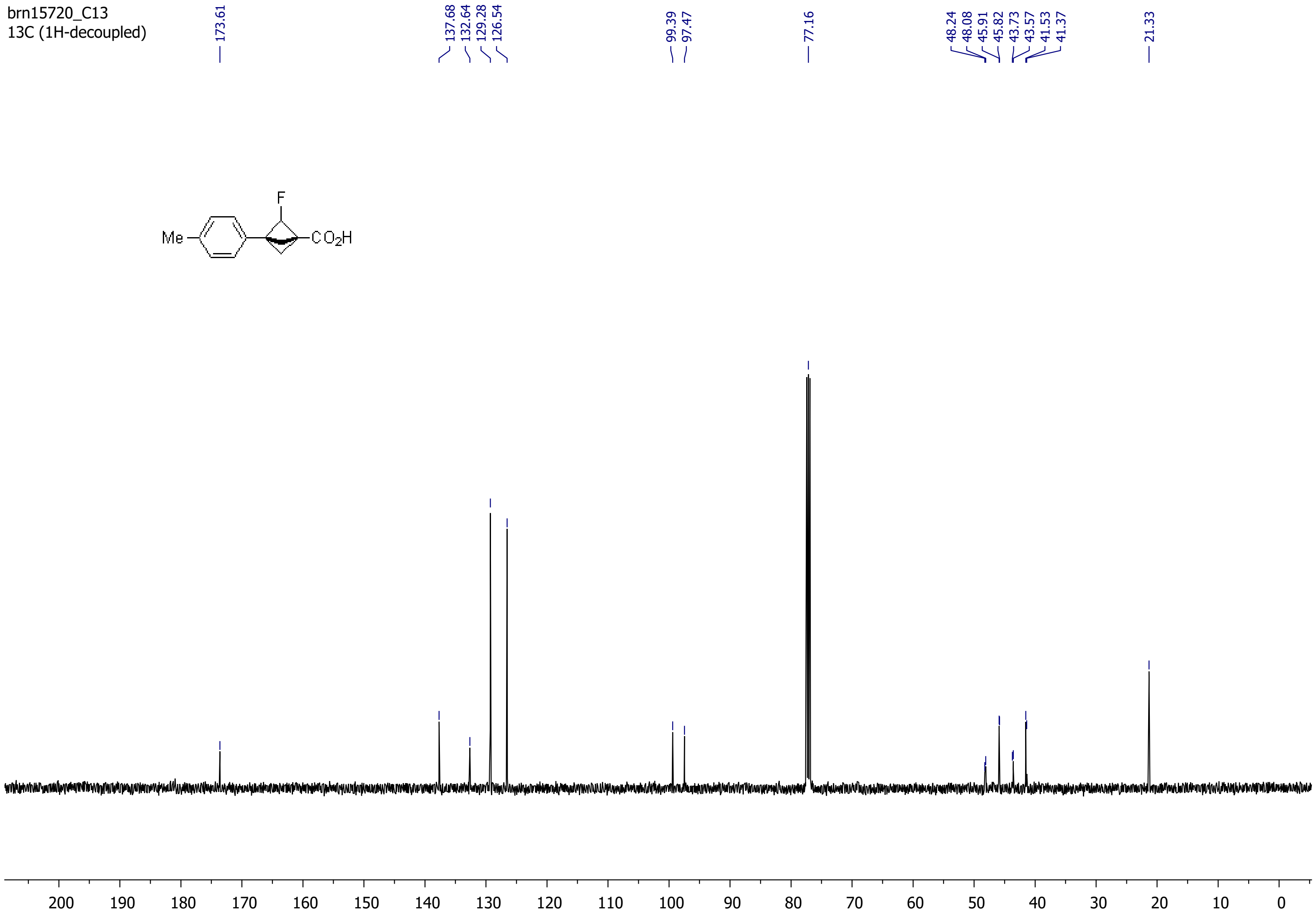

$^{19}F\{^1H\}$ NMR (376 MHz, $CDCl_3$)

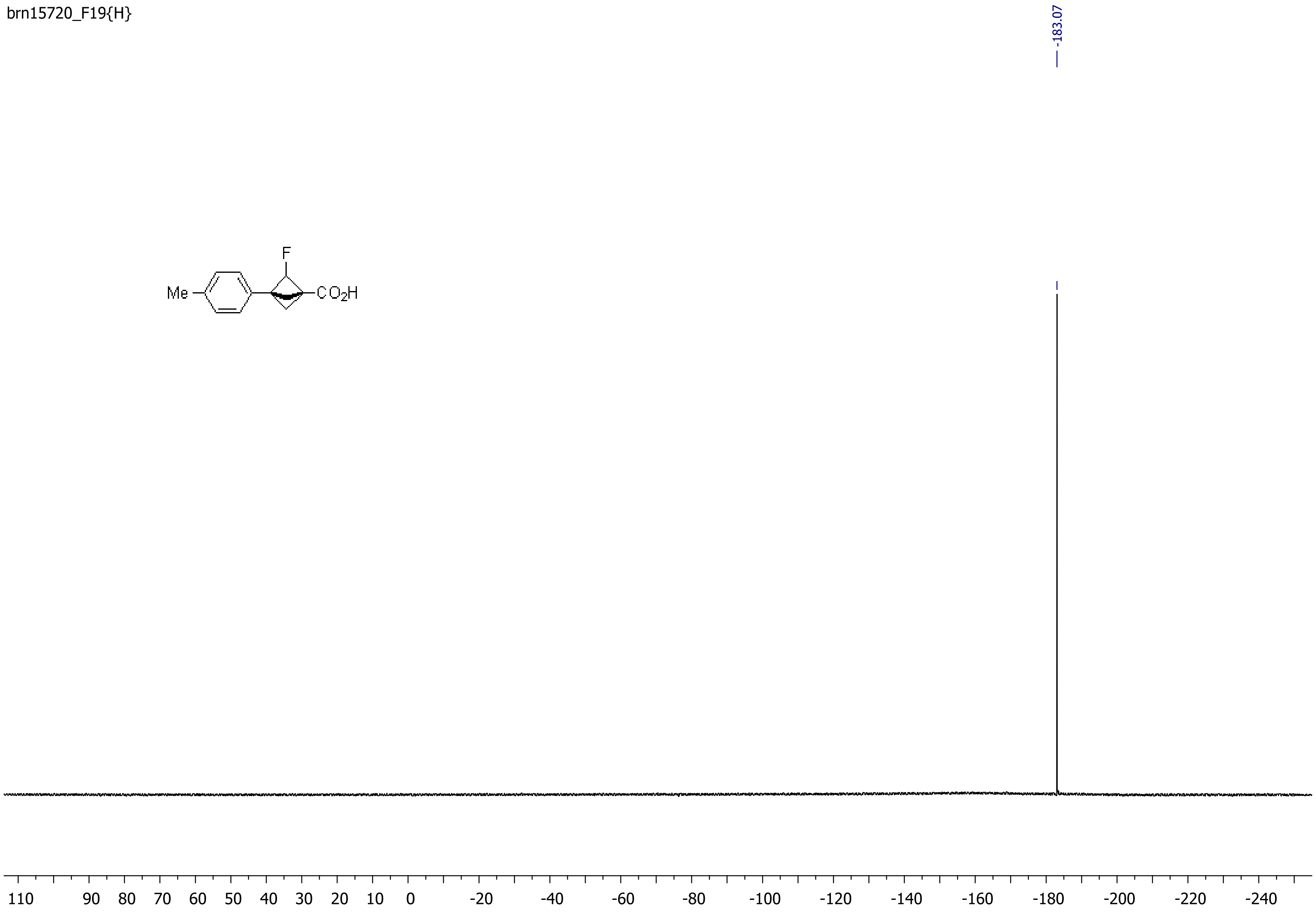

**Compound 16c**

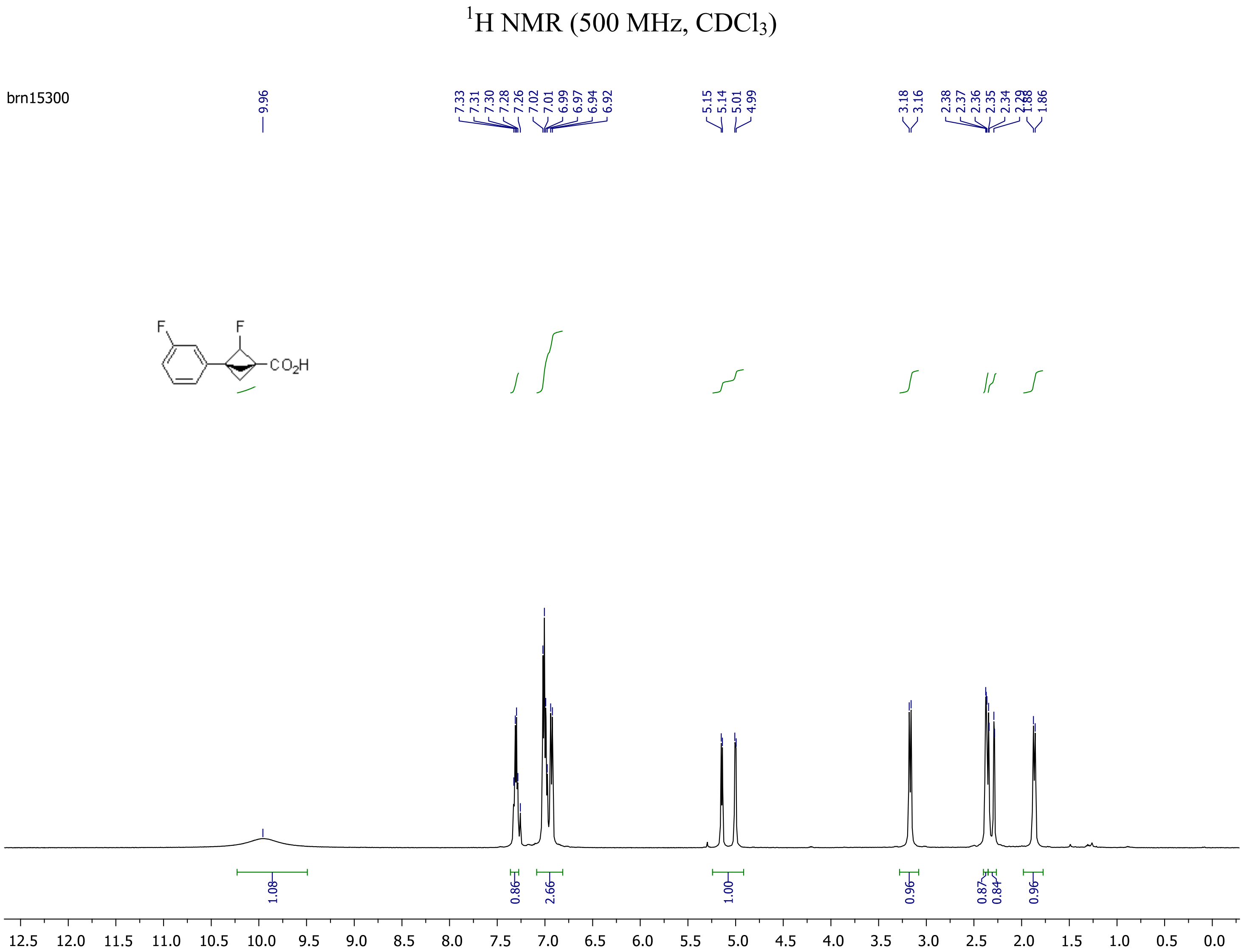

$^{13}C\{^1H\}$ NMR (126 MHz, $CDCl_3$)

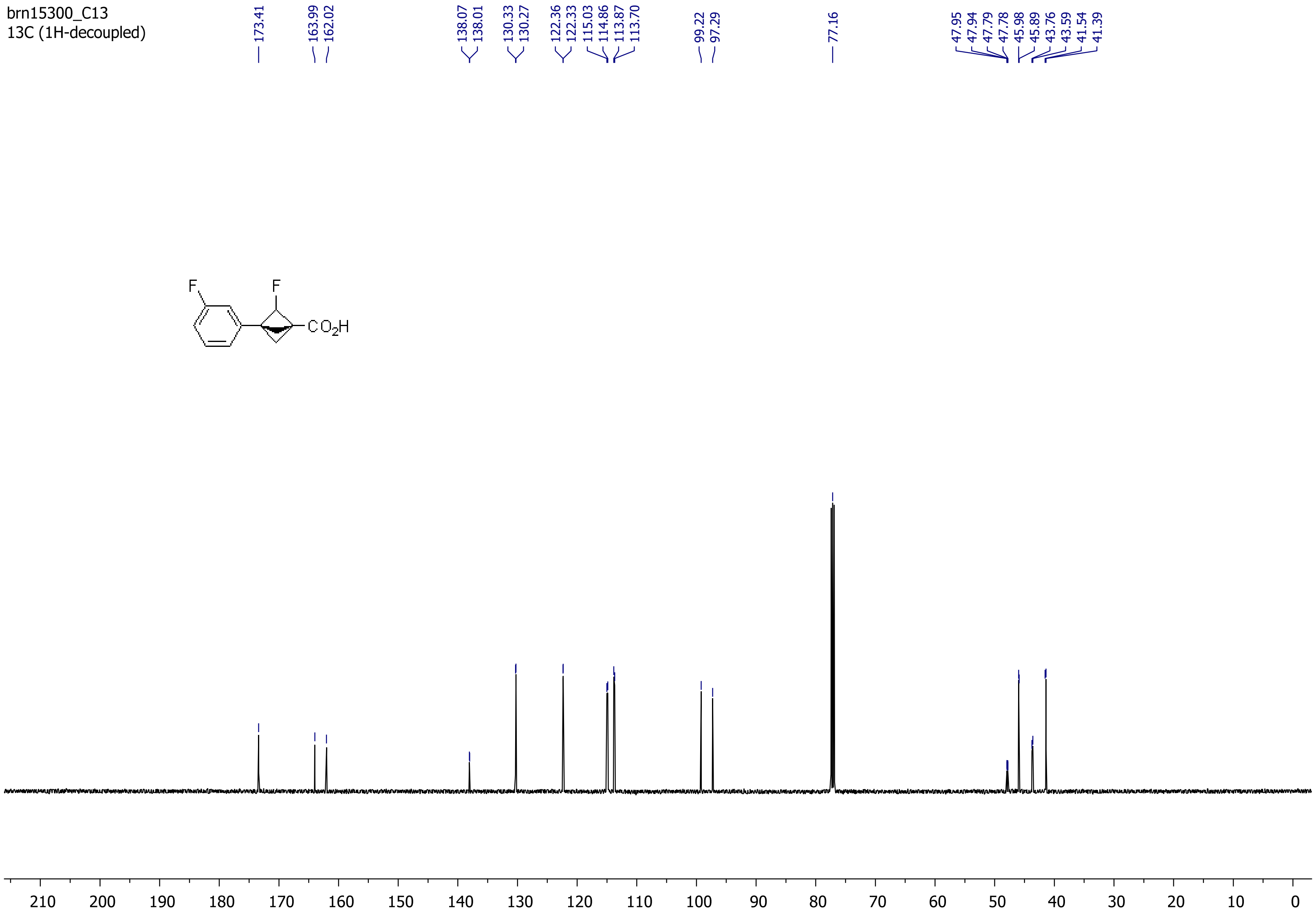

$^{19}F\{^1H\}$ NMR (376 MHz, $CDCl_3$)

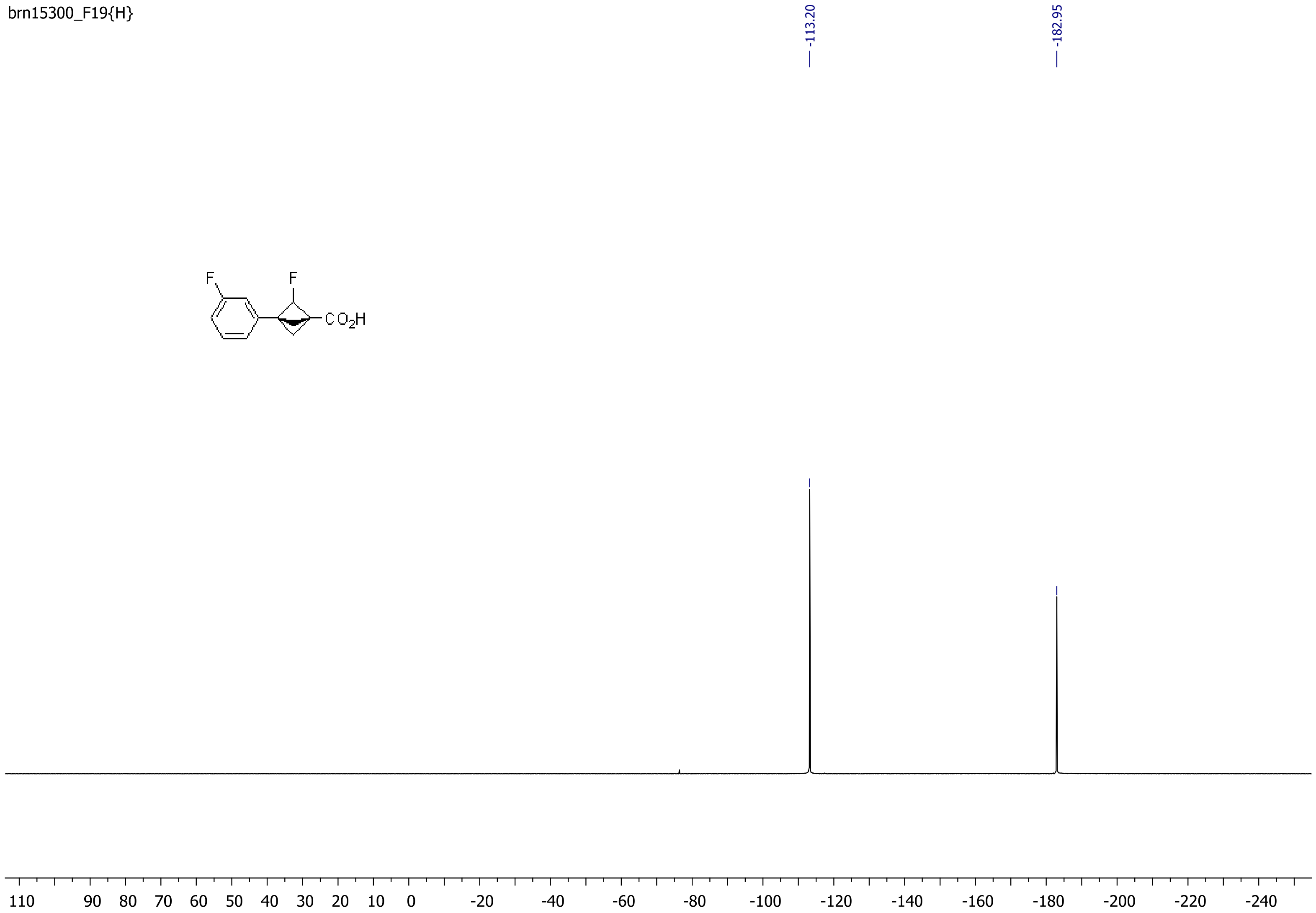

**Compound 17c**

$^1H$ NMR (400 MHz, $CDCl_3$)

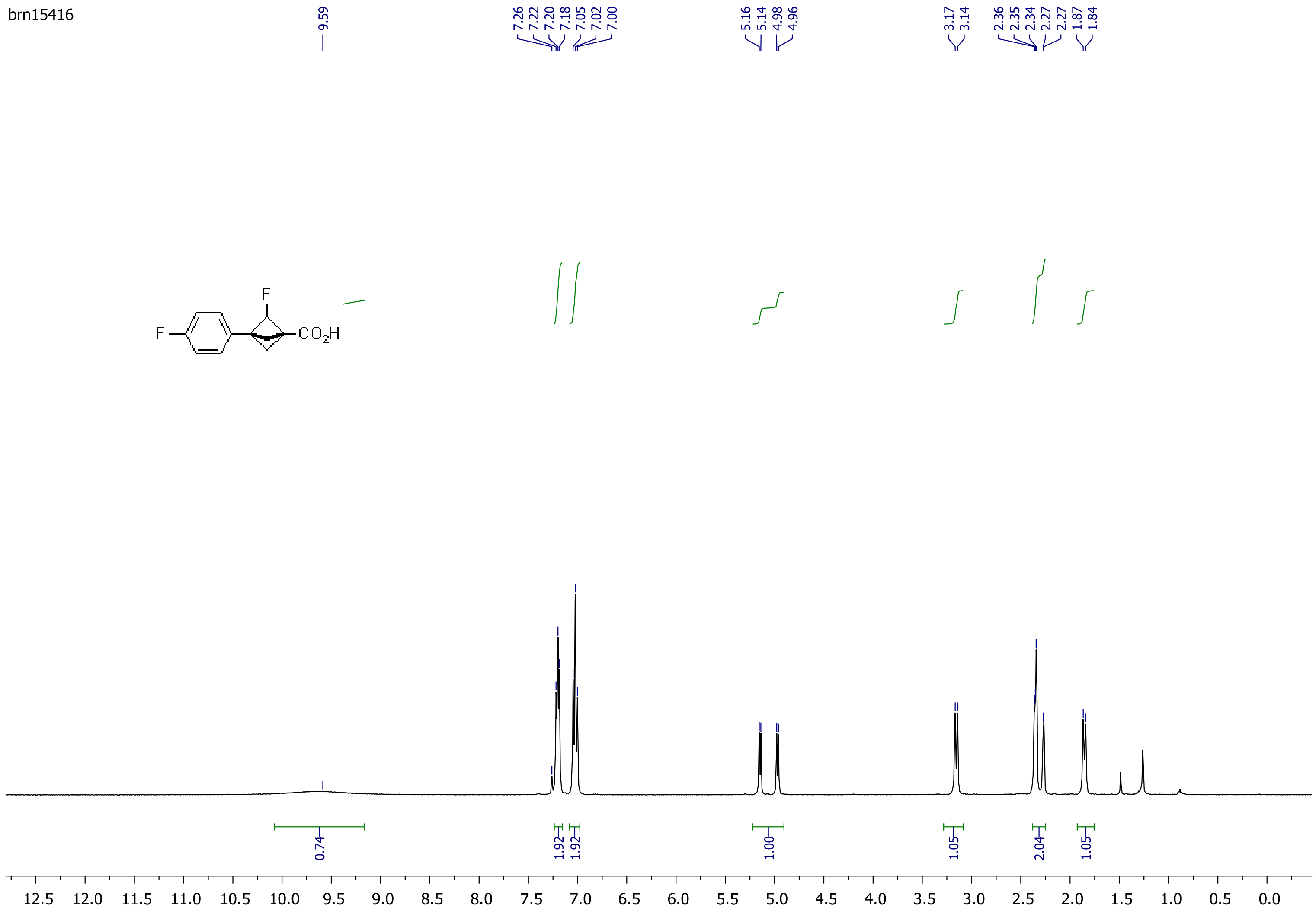

$^{13}C\{^1H\}$ NMR (126 MHz, $CDCl_3$)

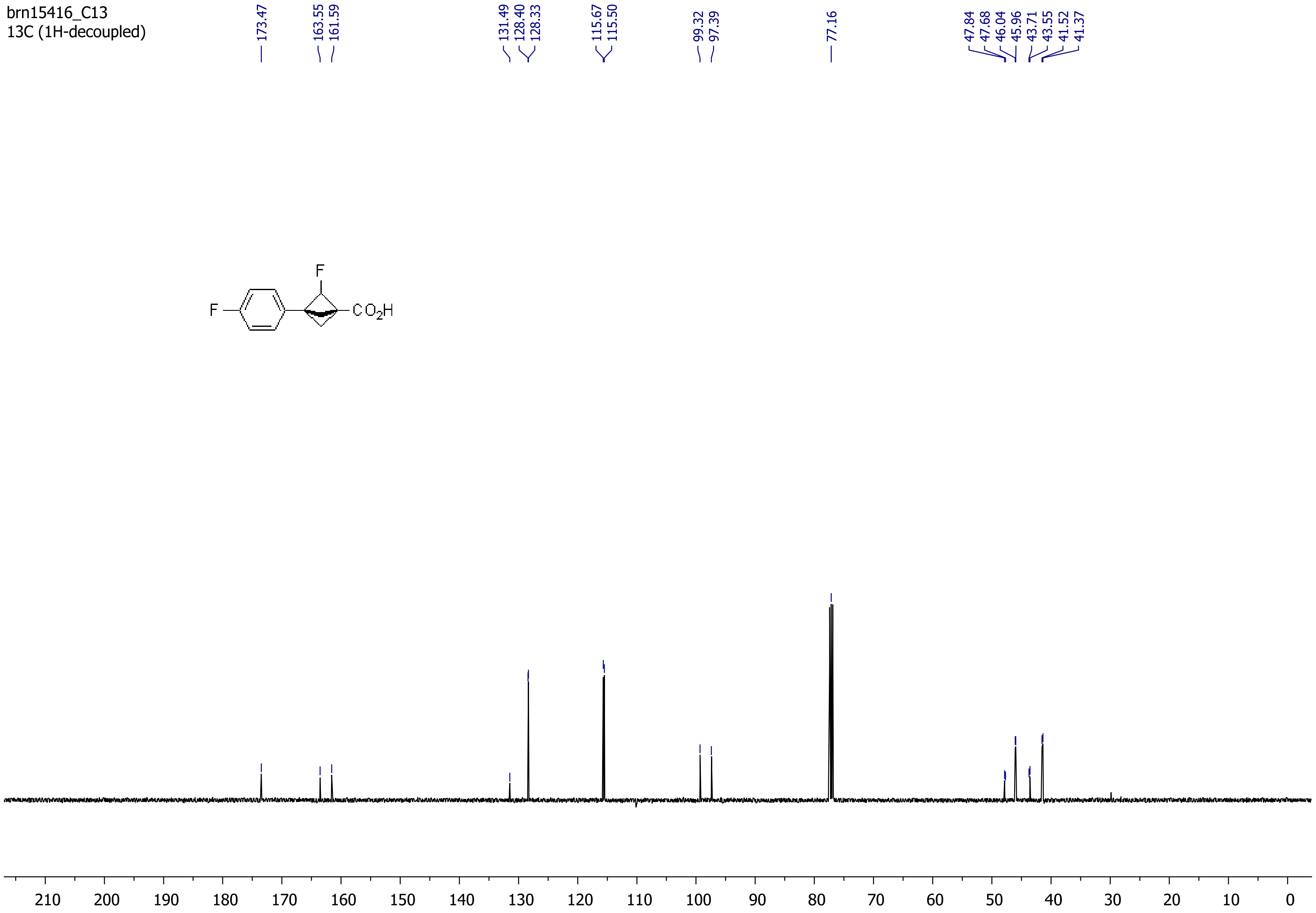

$^{19}F\{^1H\}$ NMR (376 MHz, $CDCl_3$)

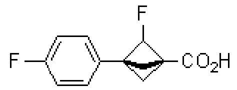

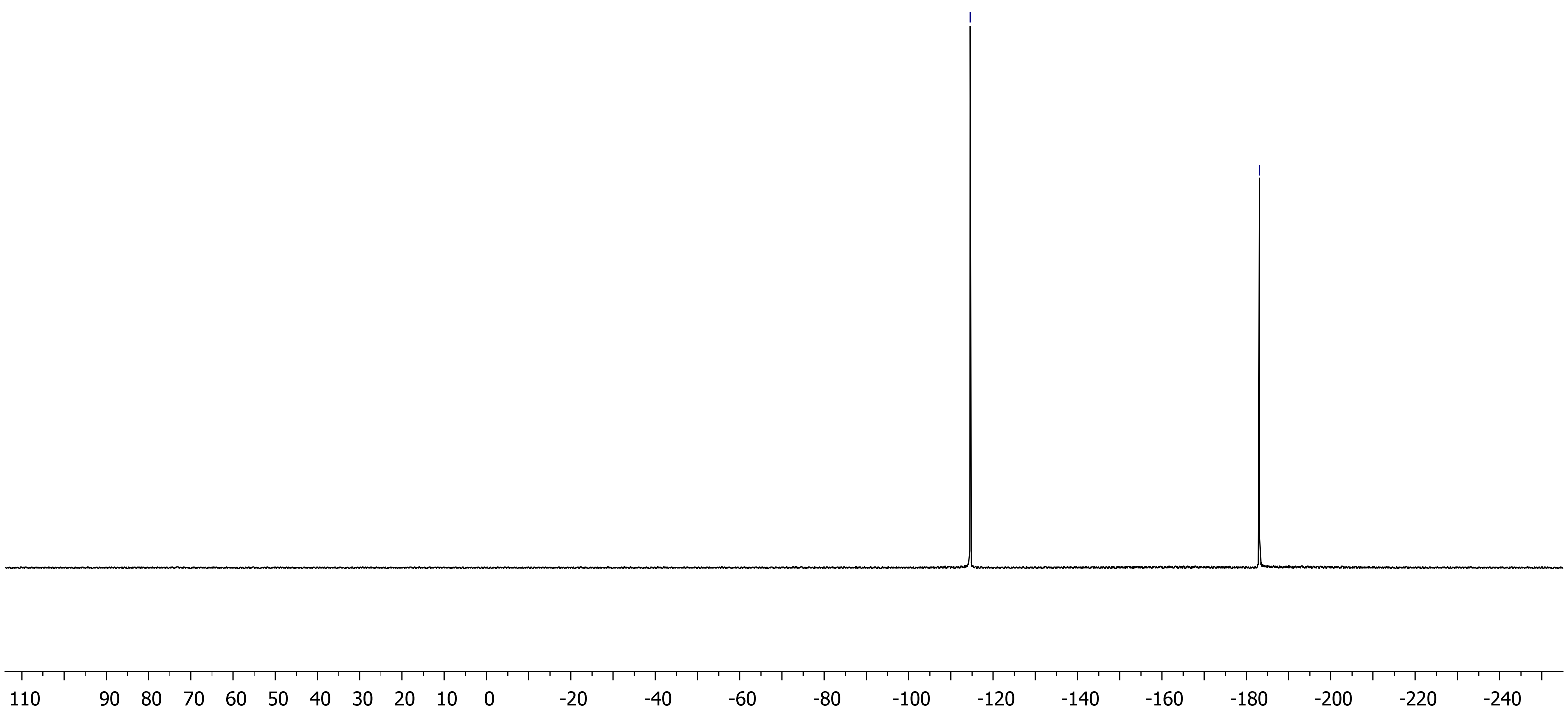

**Compound 18c**

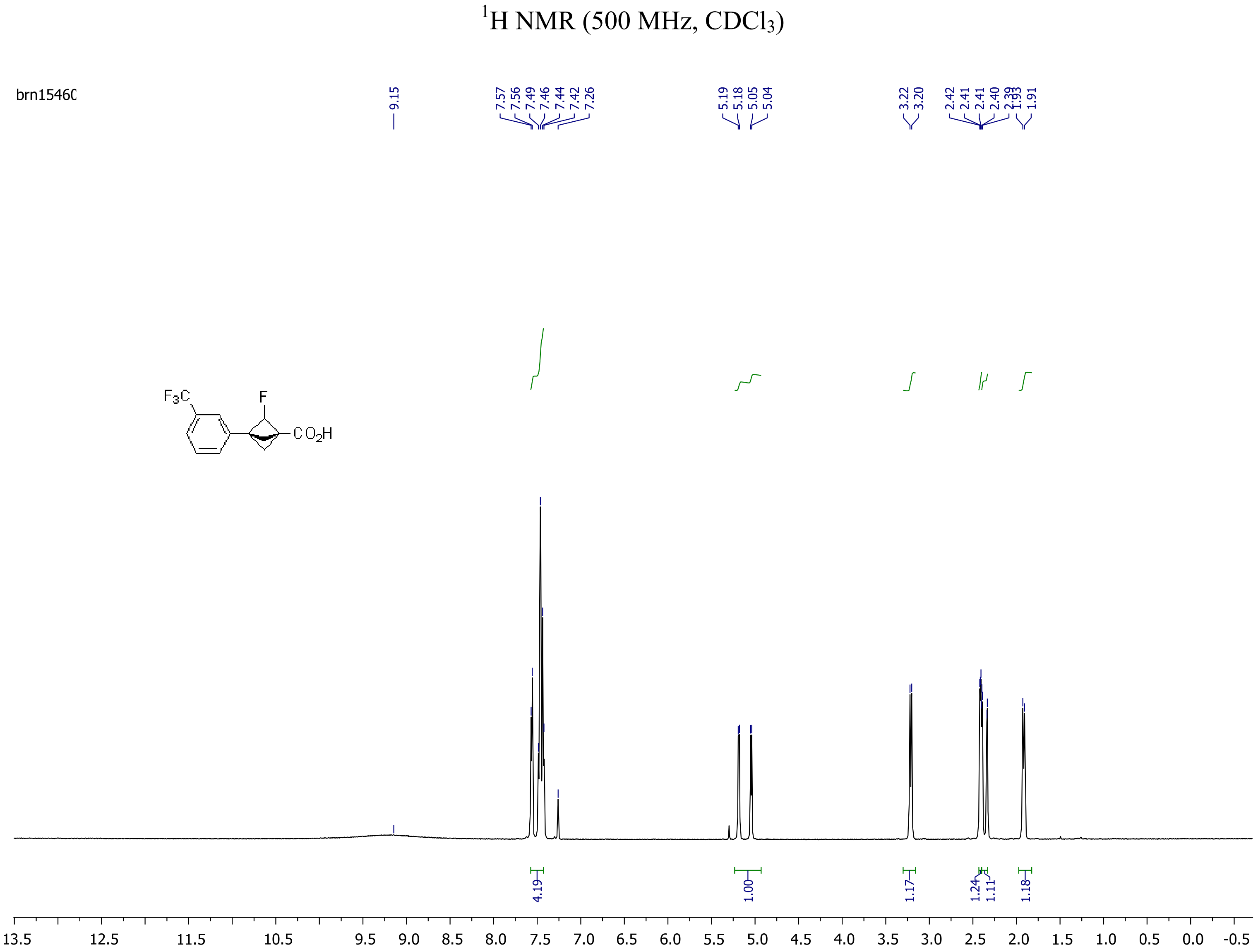

$^{13}C\{^{1}H\}$ NMR (126 MHz, $CDCl_3$)

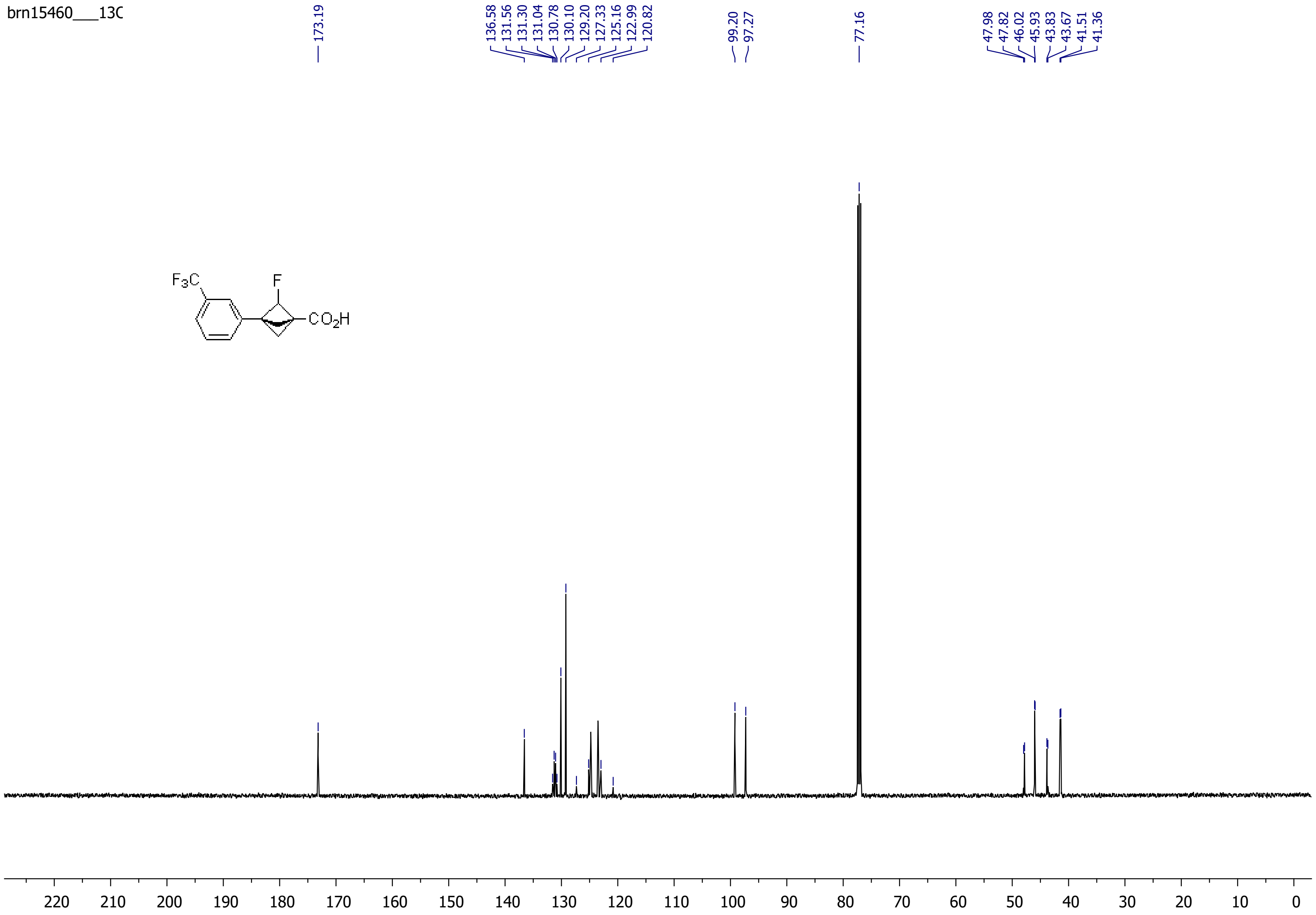

$^{19}F\{^{1}H\}$ NMR (376 MHz, $CDCl_3$)

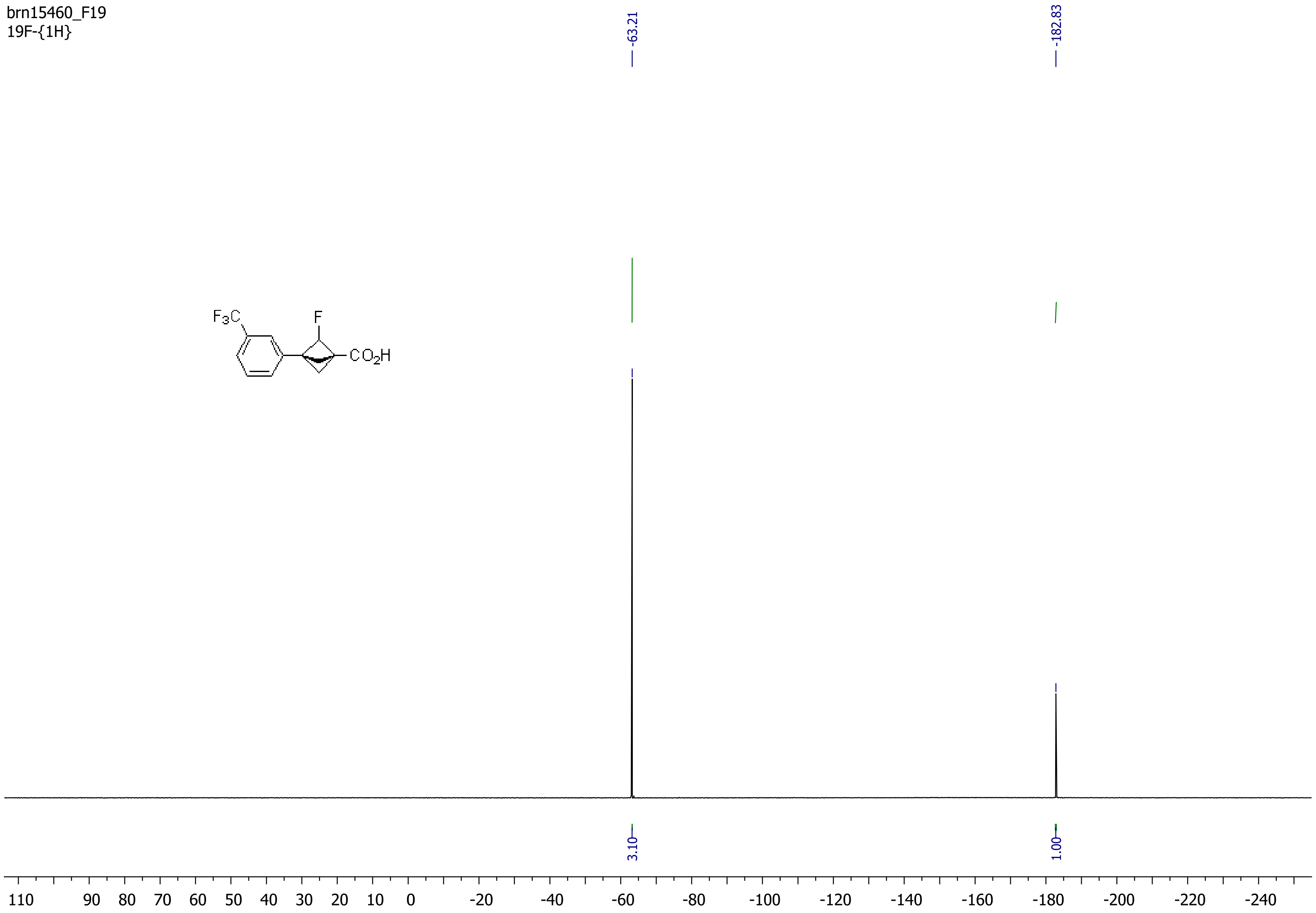

**Compound 19c**

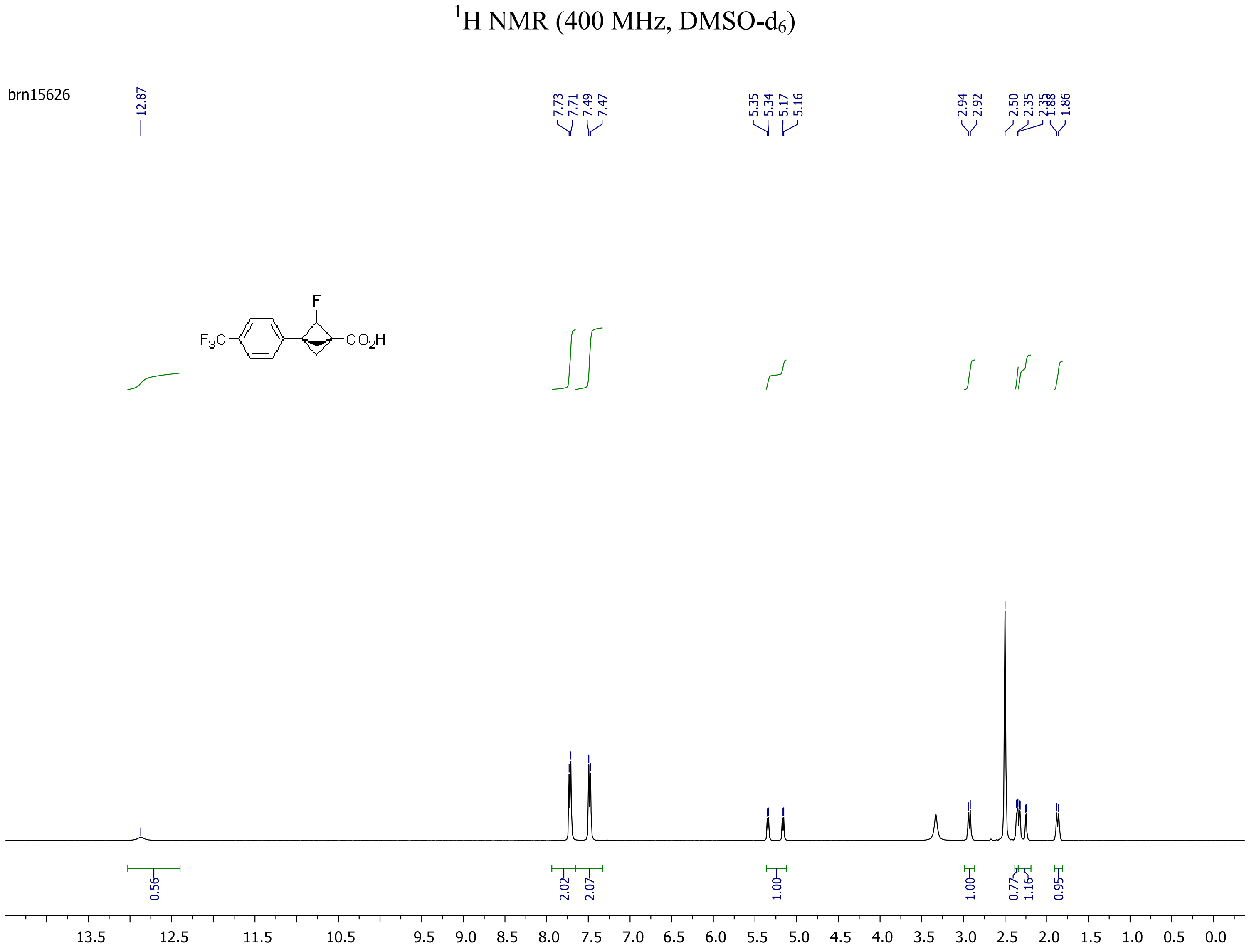

$^{19}F\{^{1}H\}$ NMR (376 MHz, DMSO-$d_6$)

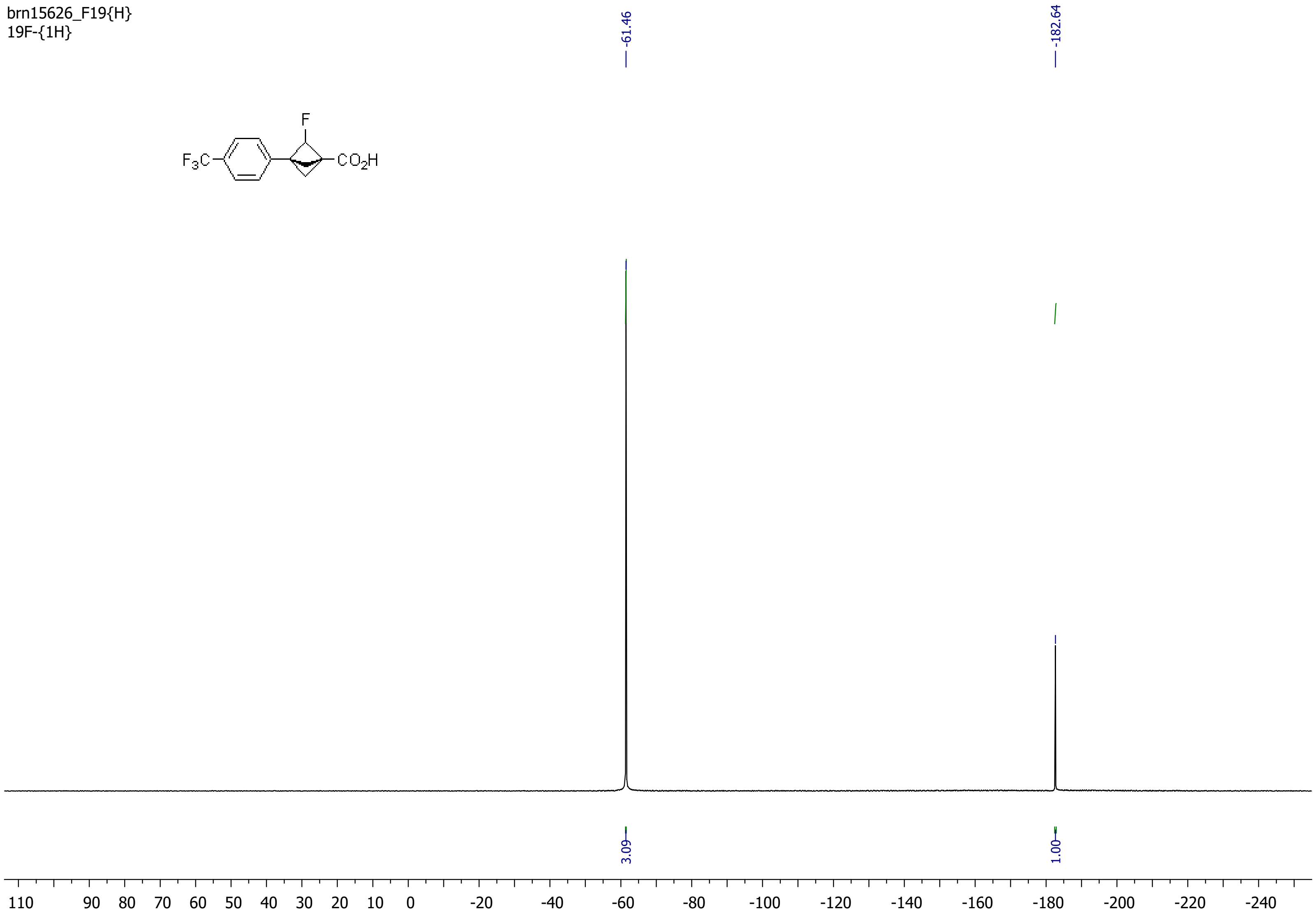

$^{13}C\{^1H\}$ NMR (151 MHz, DMSO-d$_6$)

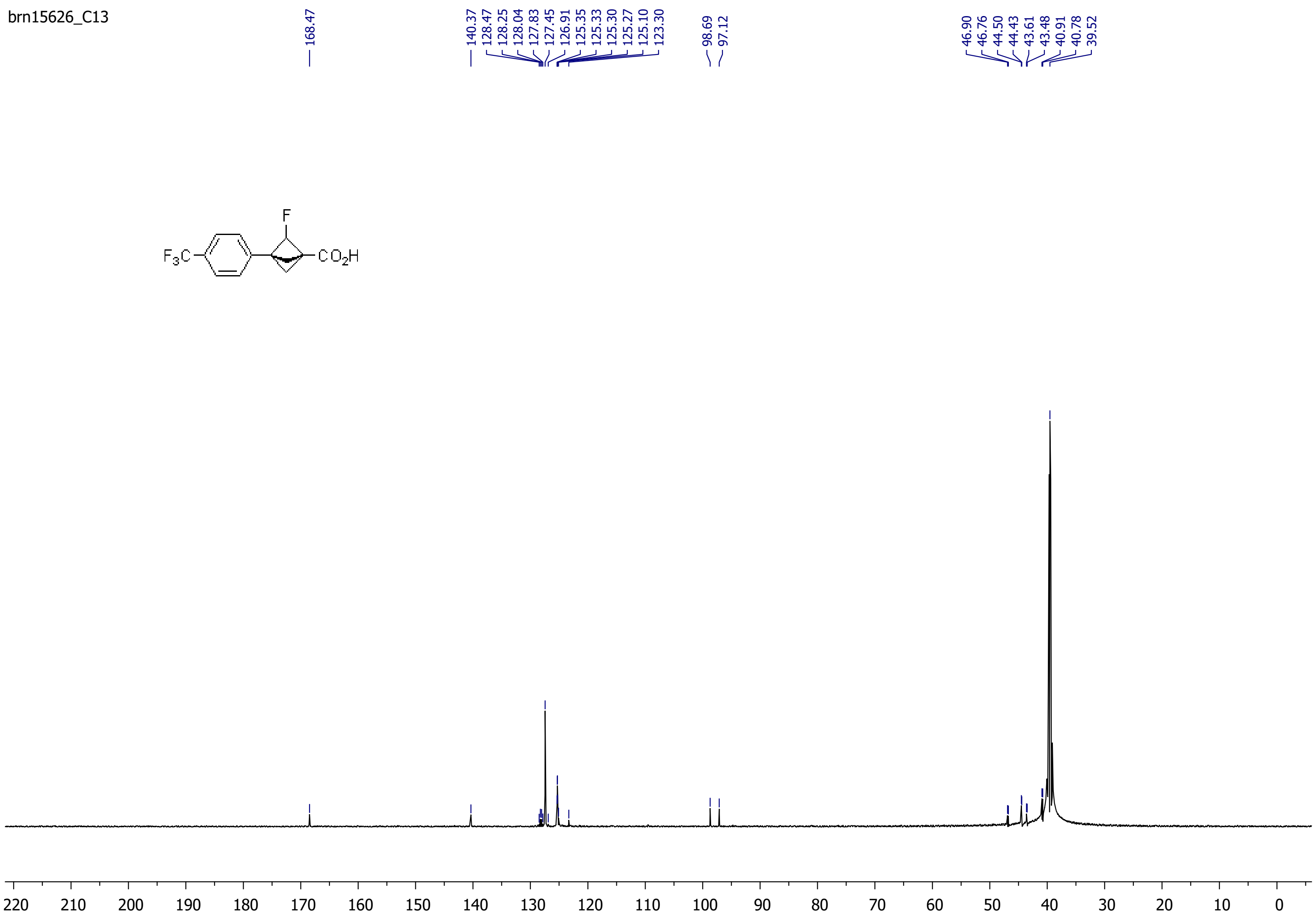

**Compound 23**

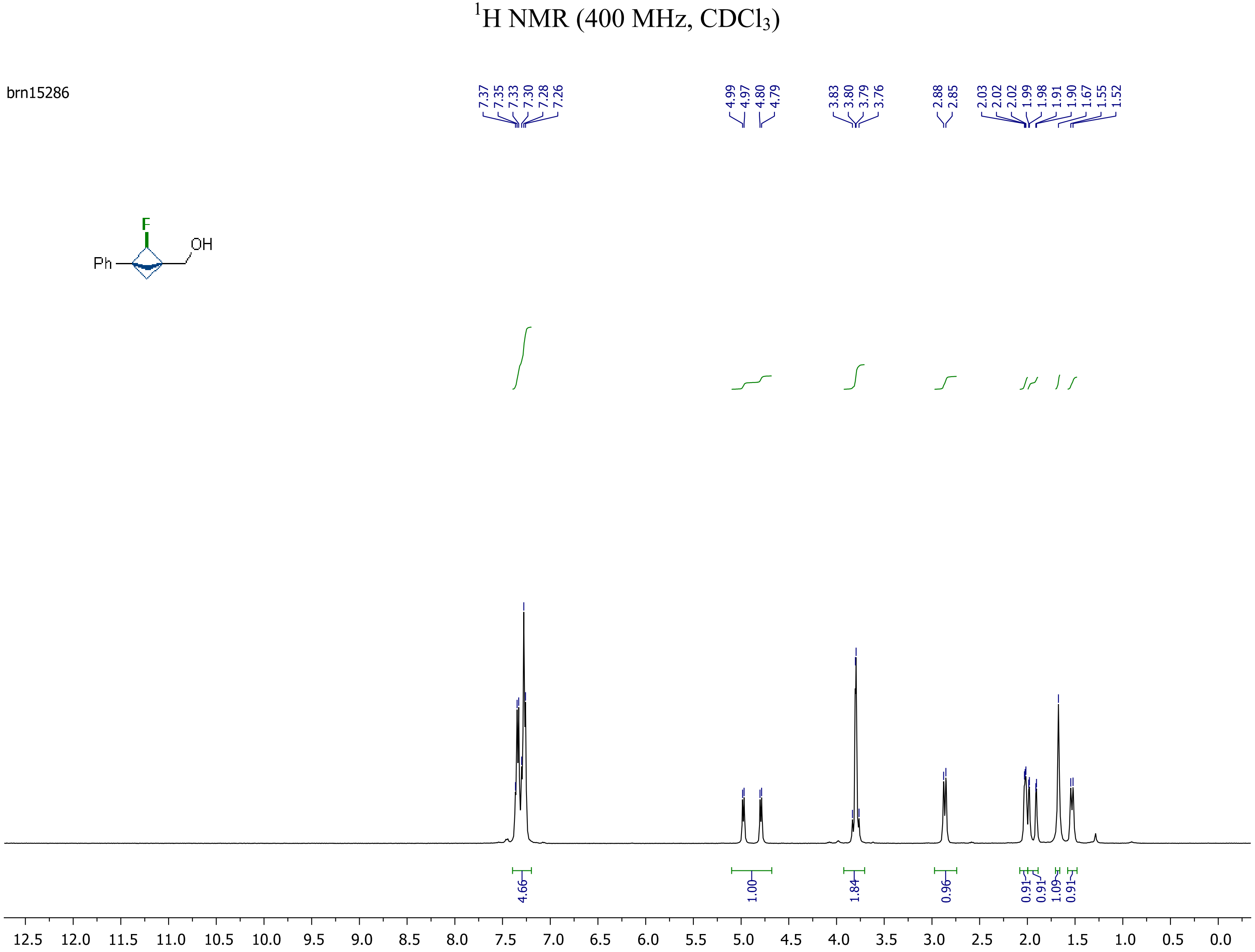
F
OH
Ph
$^1$H NMR (400 MHz, CDCl$_3$)
brn15286
7.37
7.35
7.33
7.30
7.28
7.26
4.99
4.97
4.80
4.79
3.83
3.80
3.79
3.76
2.88
2.85
2.03
2.02
2.02
1.99
1.98
1.91
1.90
1.67
1.55
1.52
4.66
1.00
1.84
0.96
0.91
0.91
1.09
0.91
12.5 12.0 11.5 11.0 10.5 10.0 9.5 9.0 8.5 8.0 7.5 7.0 6.5 6.0 5.5 5.0 4.5 4.0 3.5 3.0 2.5 2.0 1.5 1.0 0.5 0.0

$^{13}C\{^{1}H\}$ NMR (151 MHz, $CDCl_3$)

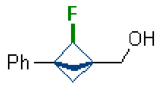

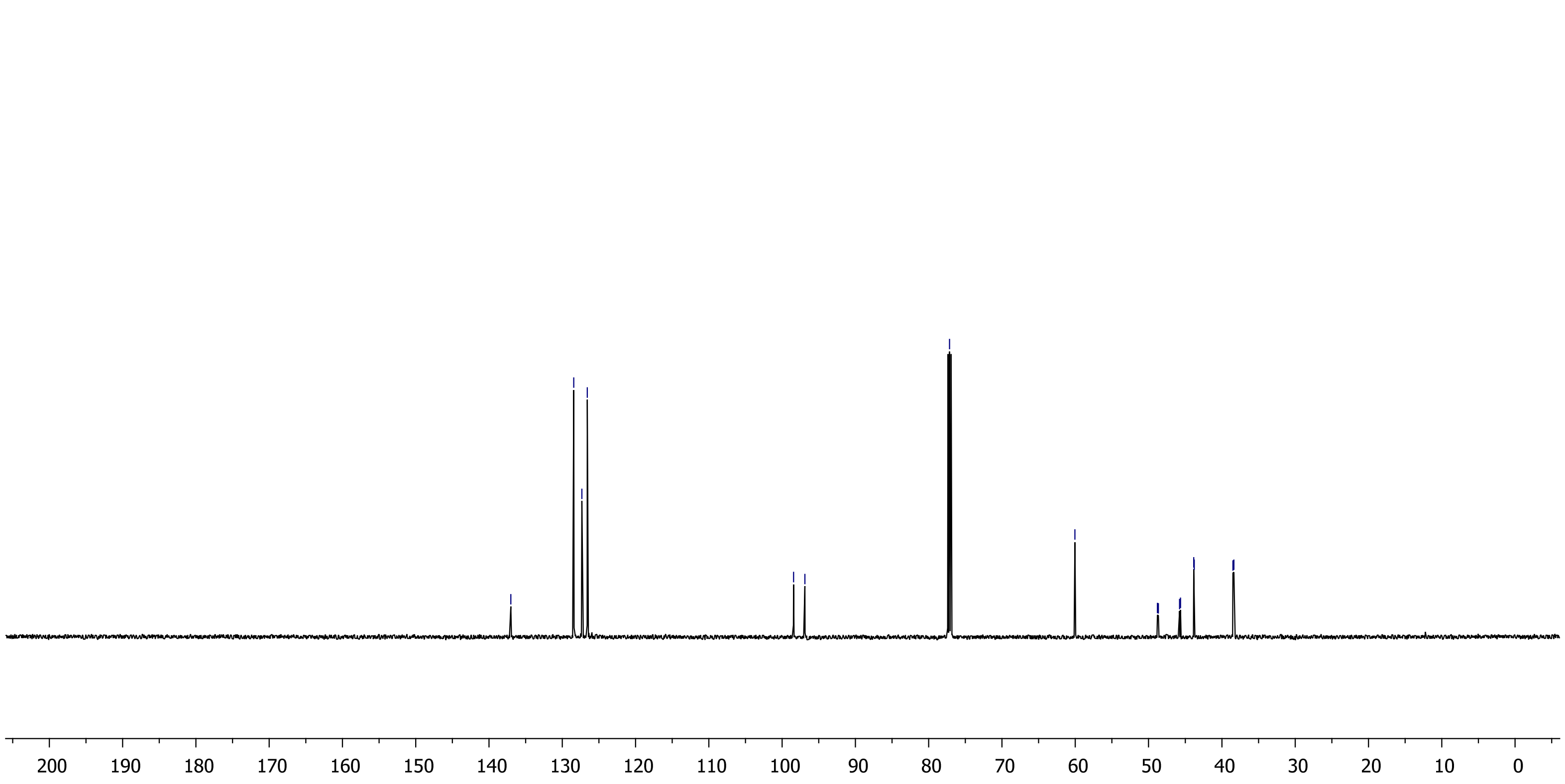

$^{19}F\{^1H\}$ NMR (376 MHz, $CDCl_3$)

brn15286_F19{H}
19F-{1H}

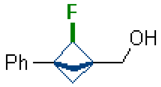


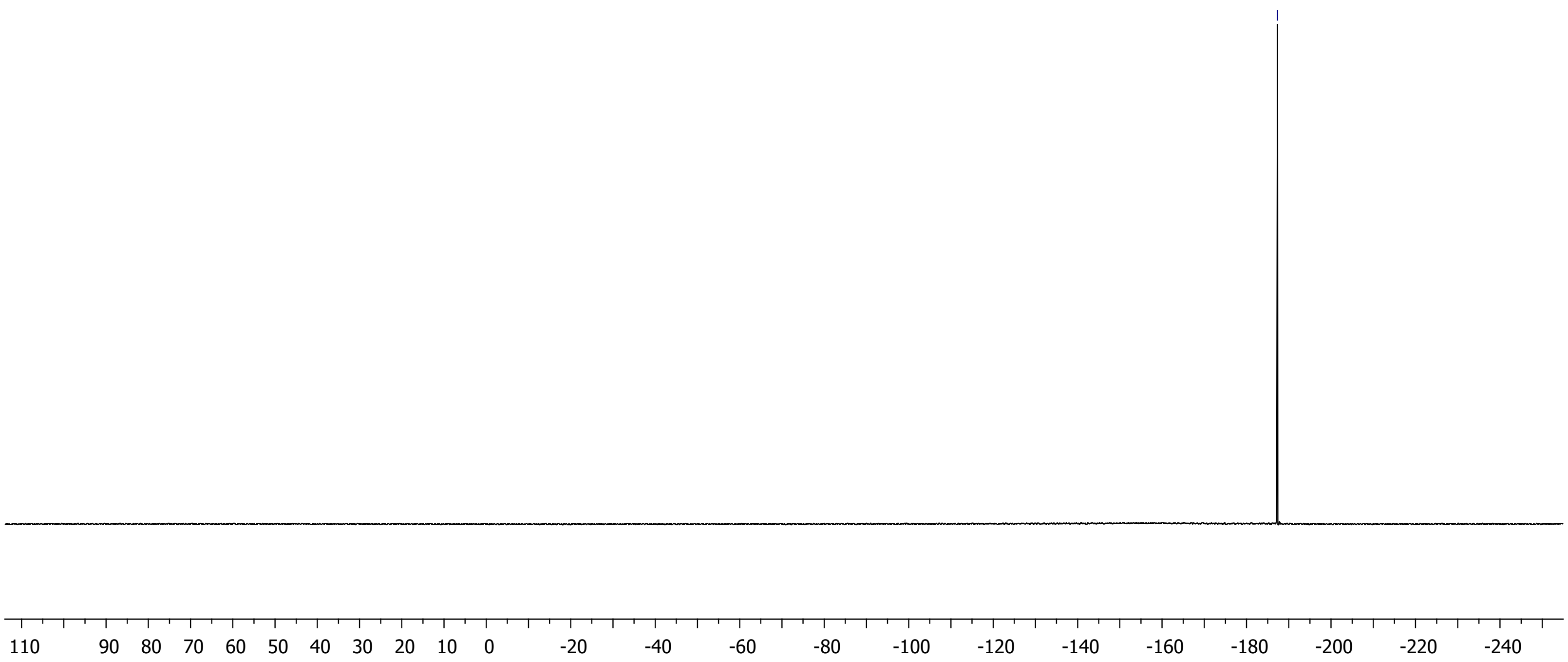

**Compound 24**

$^{1}H$ NMR (500 MHz, $CDCl_3$)

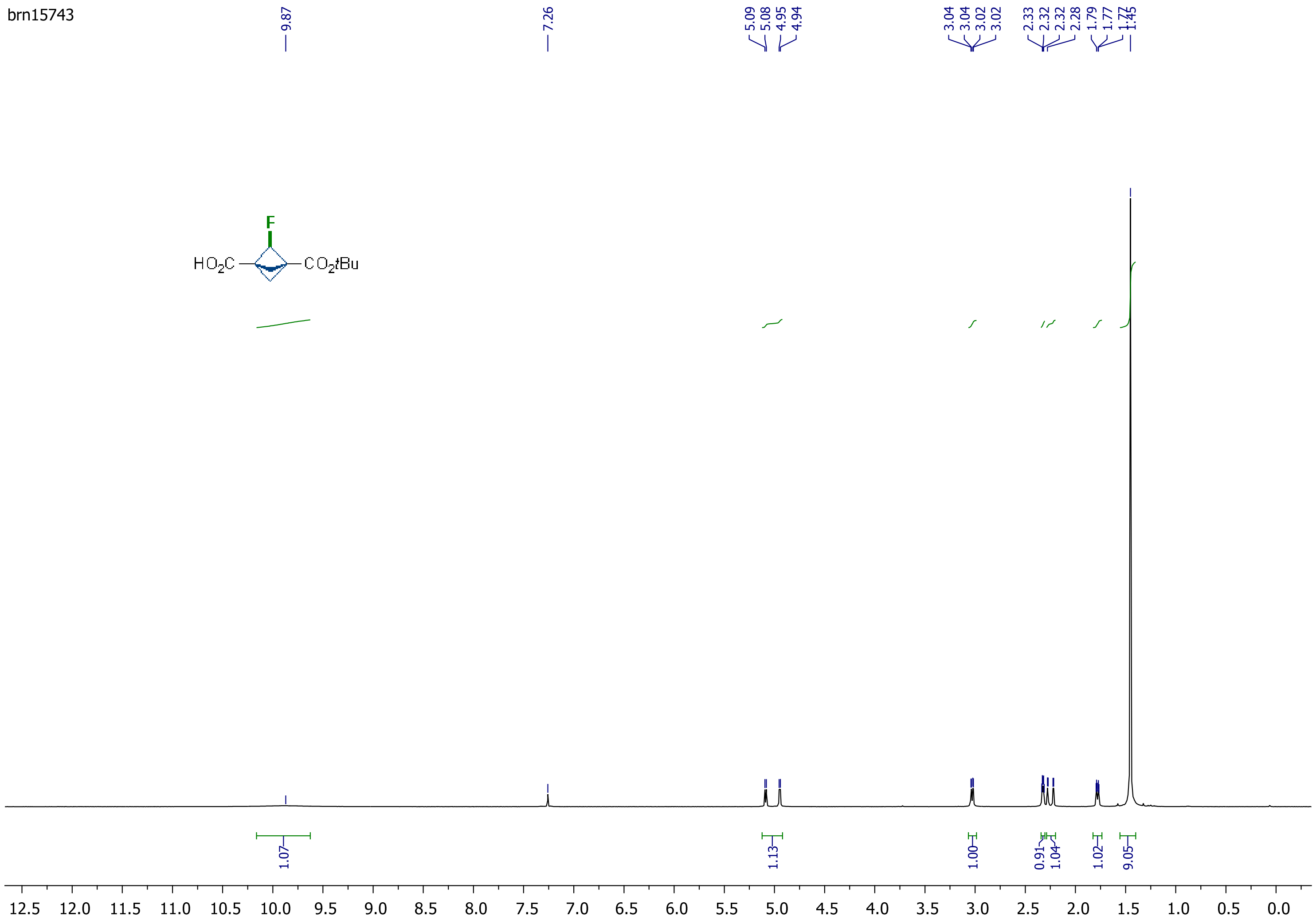

$^{13}C\{^{1}H\}$ NMR (126 MHz, $CDCl_3$)

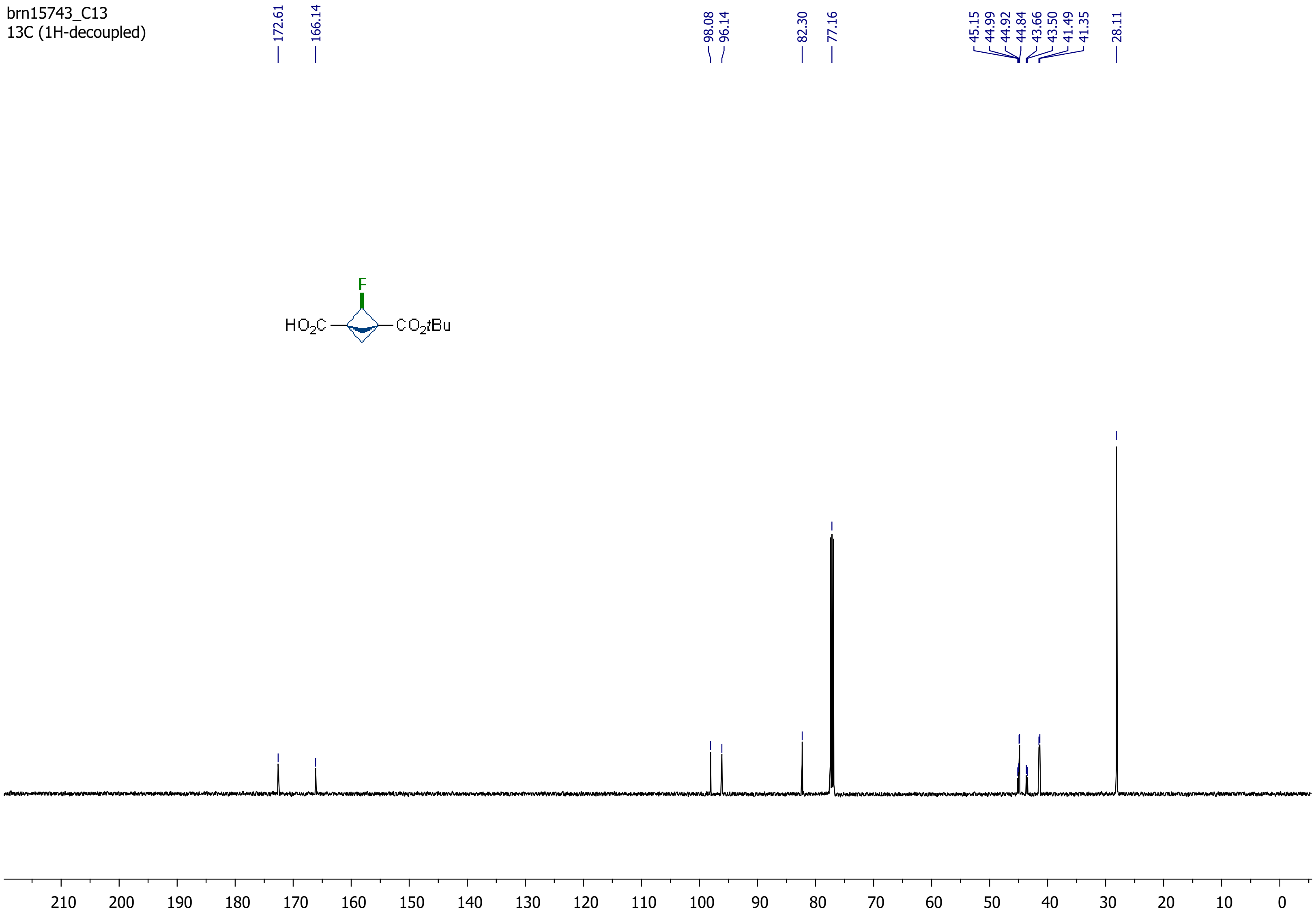

$^{19}F\{^{1}H\}$ NMR (376 MHz, $CDCl_3$)

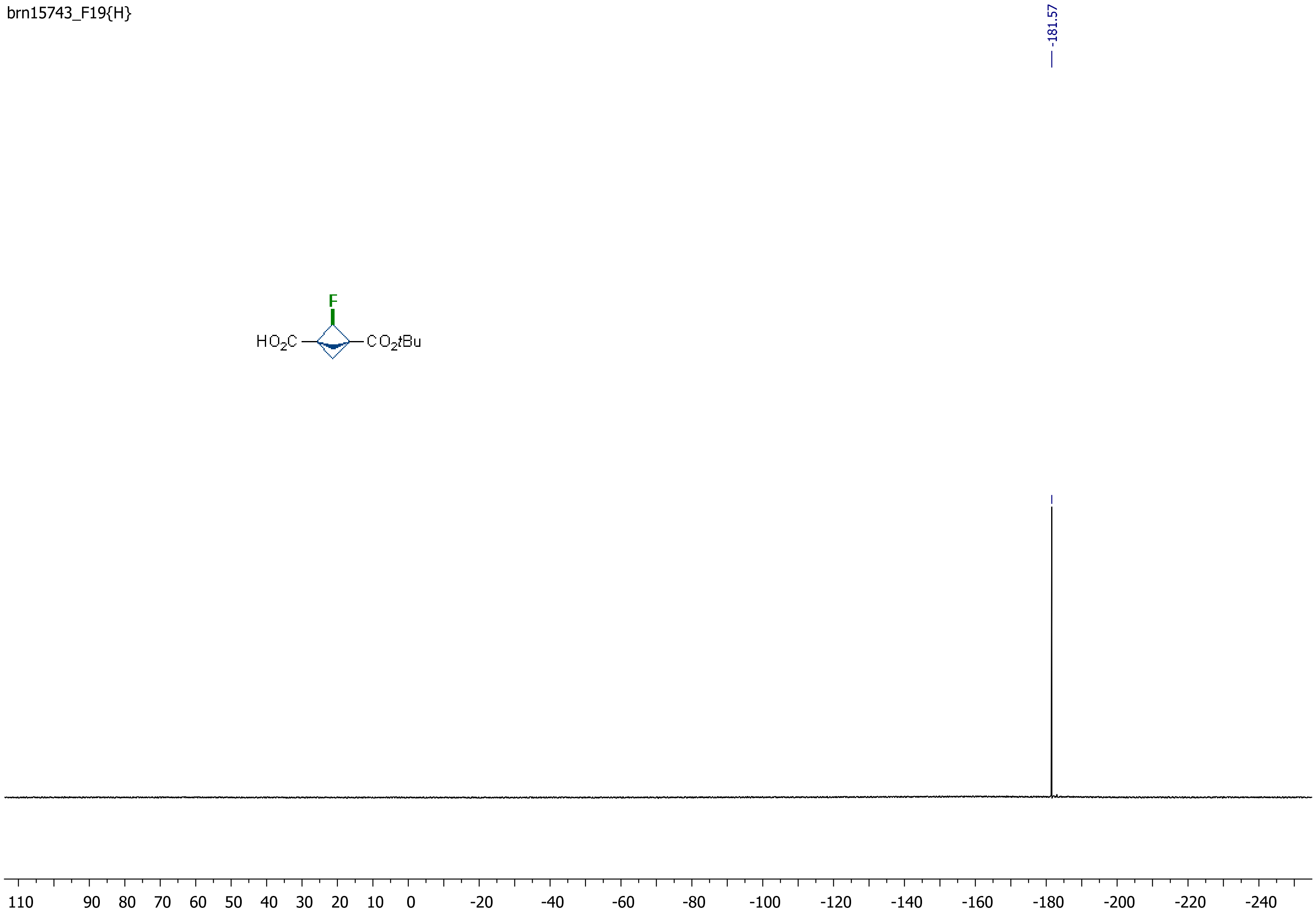

**Compound 25**

$^{1}$H NMR (400 MHz, DMSO-d$_6$)

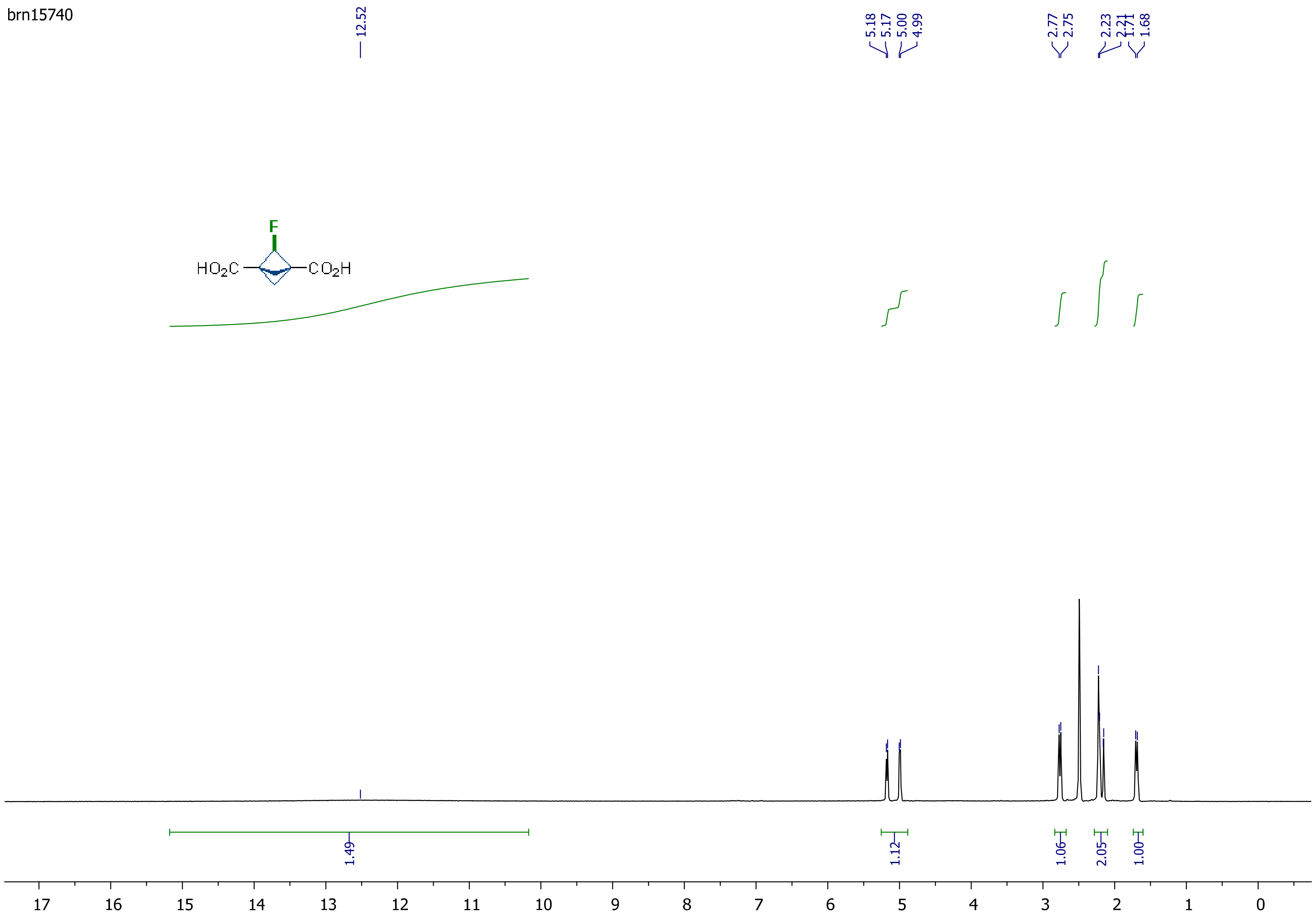

$^{13}C\{^1H\}$ NMR (101 MHz, DMSO-$d_6$)

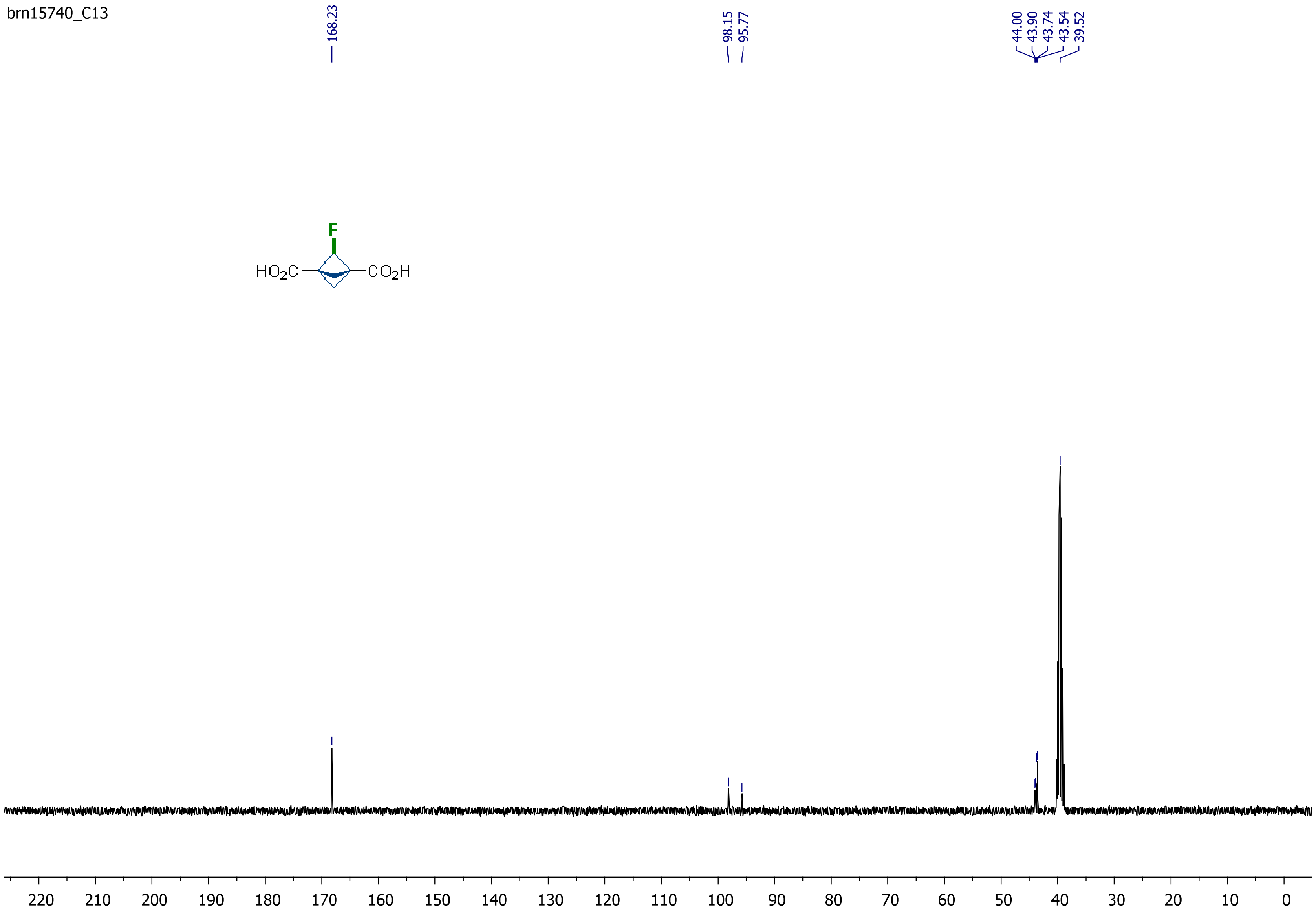

$^{19}F\{^1H\}$ NMR (376 MHz, DMSO-$d_6$)

brn15740_F19{H}

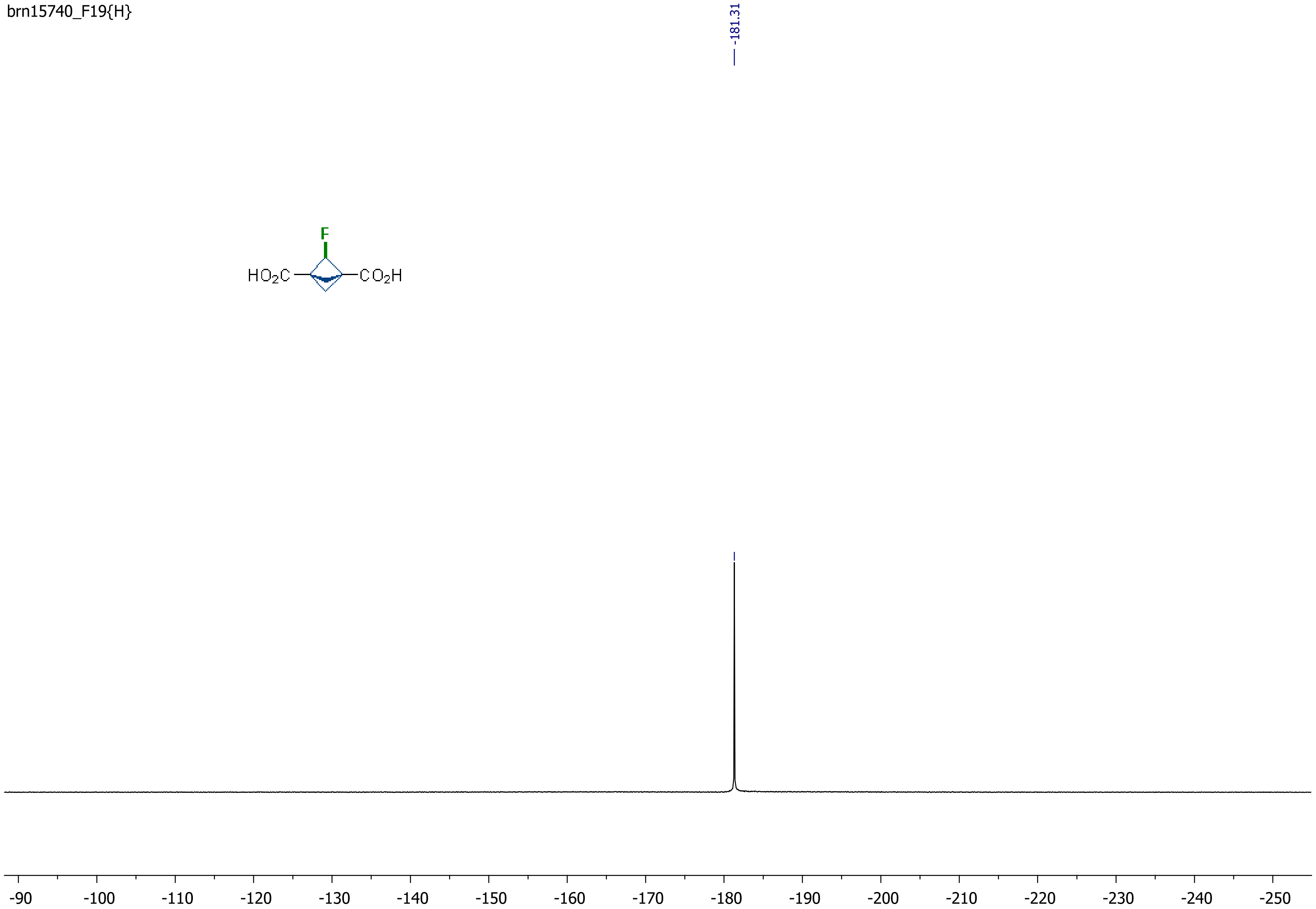

**Tert-butyl (2-fluoro-3-phenylbicyclo[1.1.1]pentan-1-yl)carbamate**

$^{1}$H NMR (500 MHz, DMSO-d$_6$)

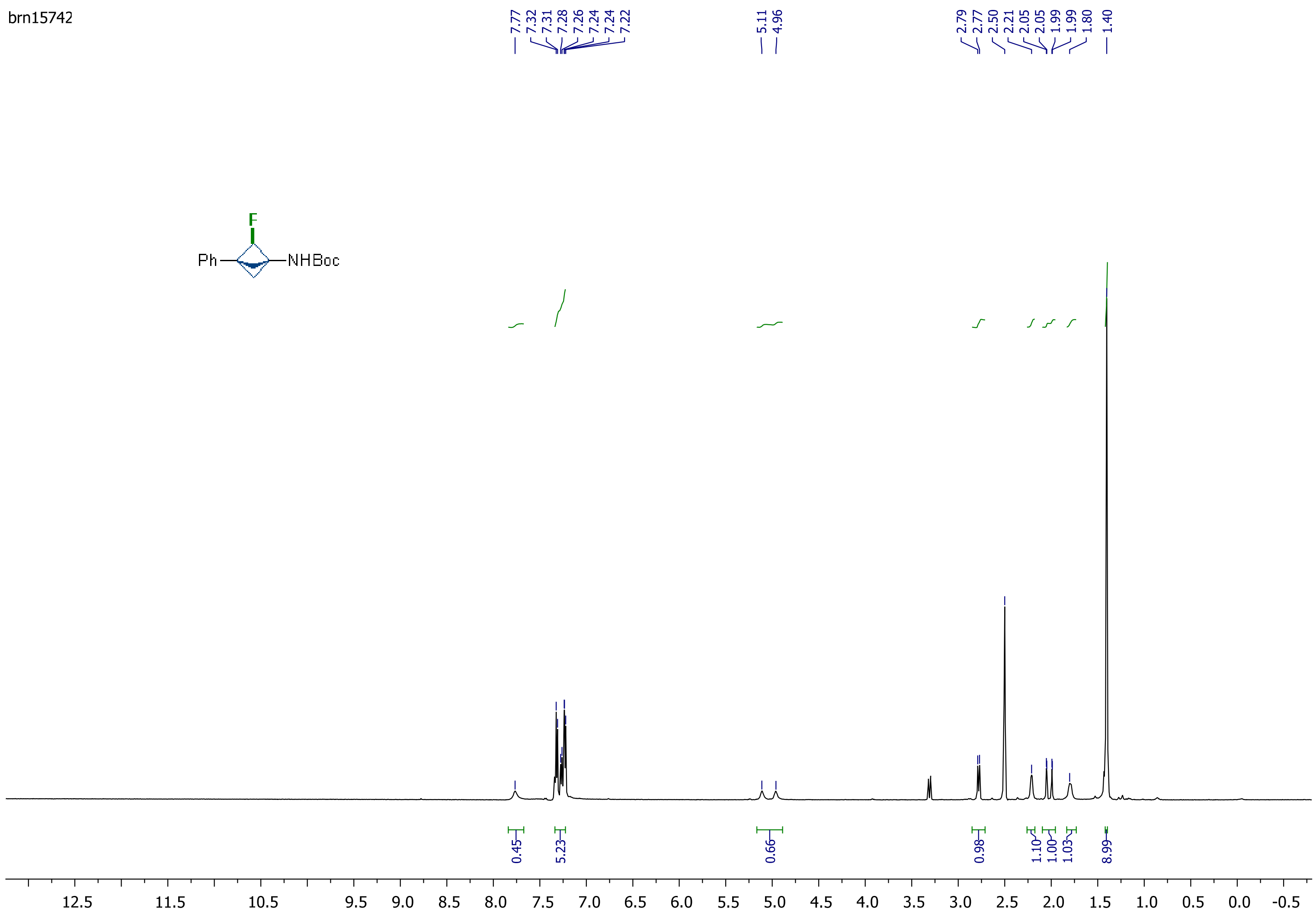

$^{13}C\{^1H\}$ NMR (151 MHz, DMSO-$d_6$)

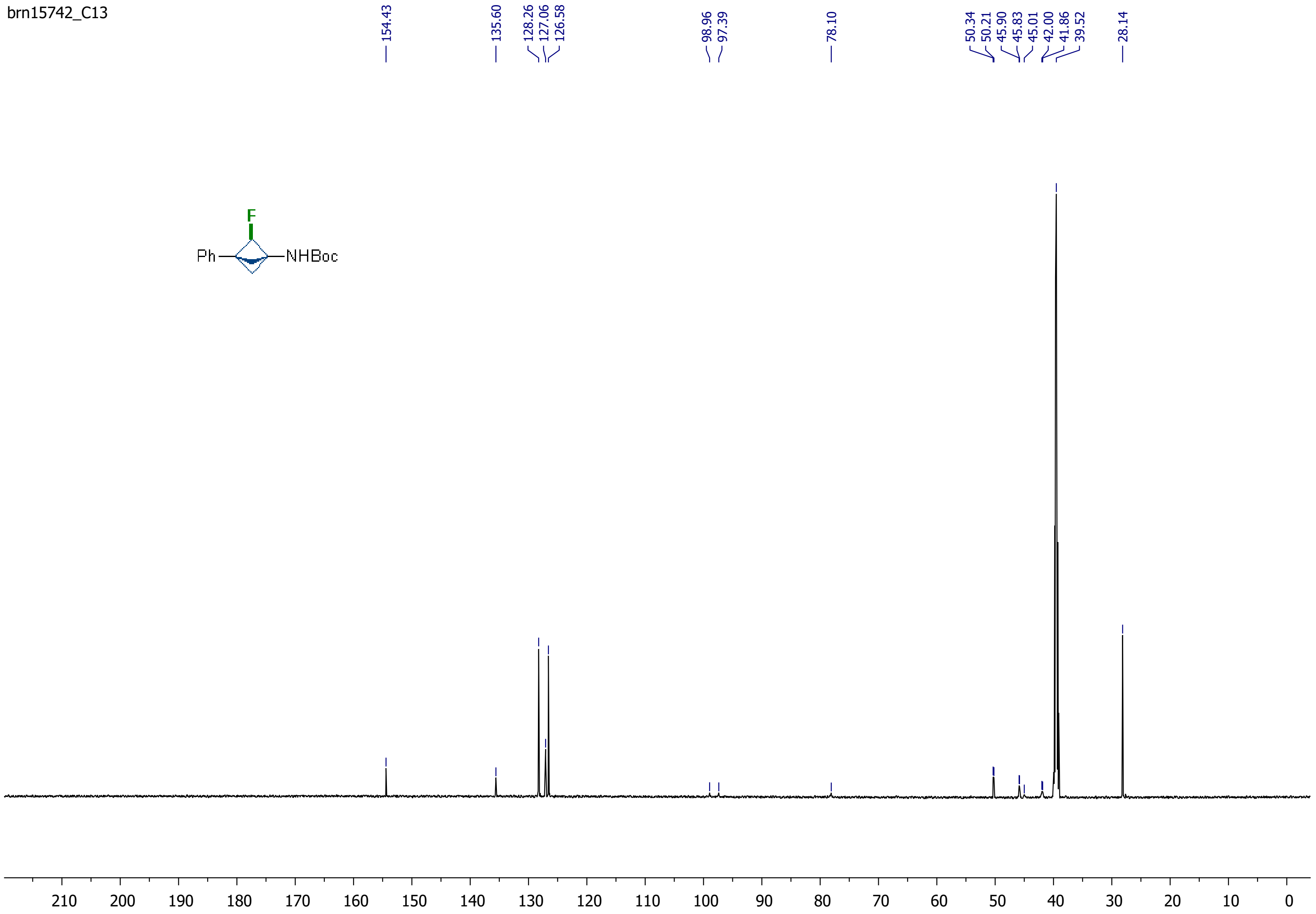

$^{19}F\{^{1}H\}$ NMR (376 MHz, DMSO-$d_6$)

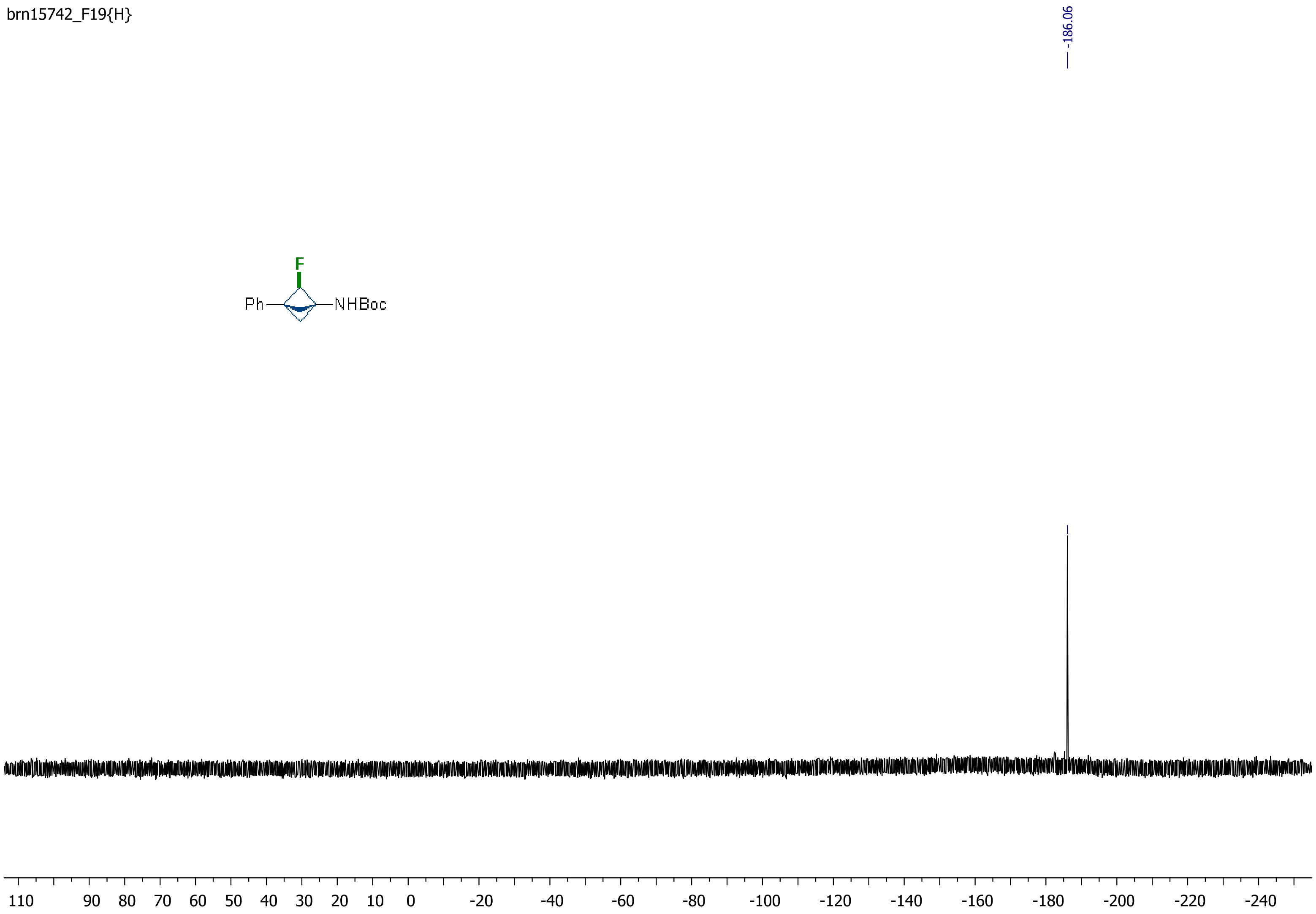

**Compound 27*HCl**

$^{1}$H NMR (500 MHz, DMSO-$d_6$)

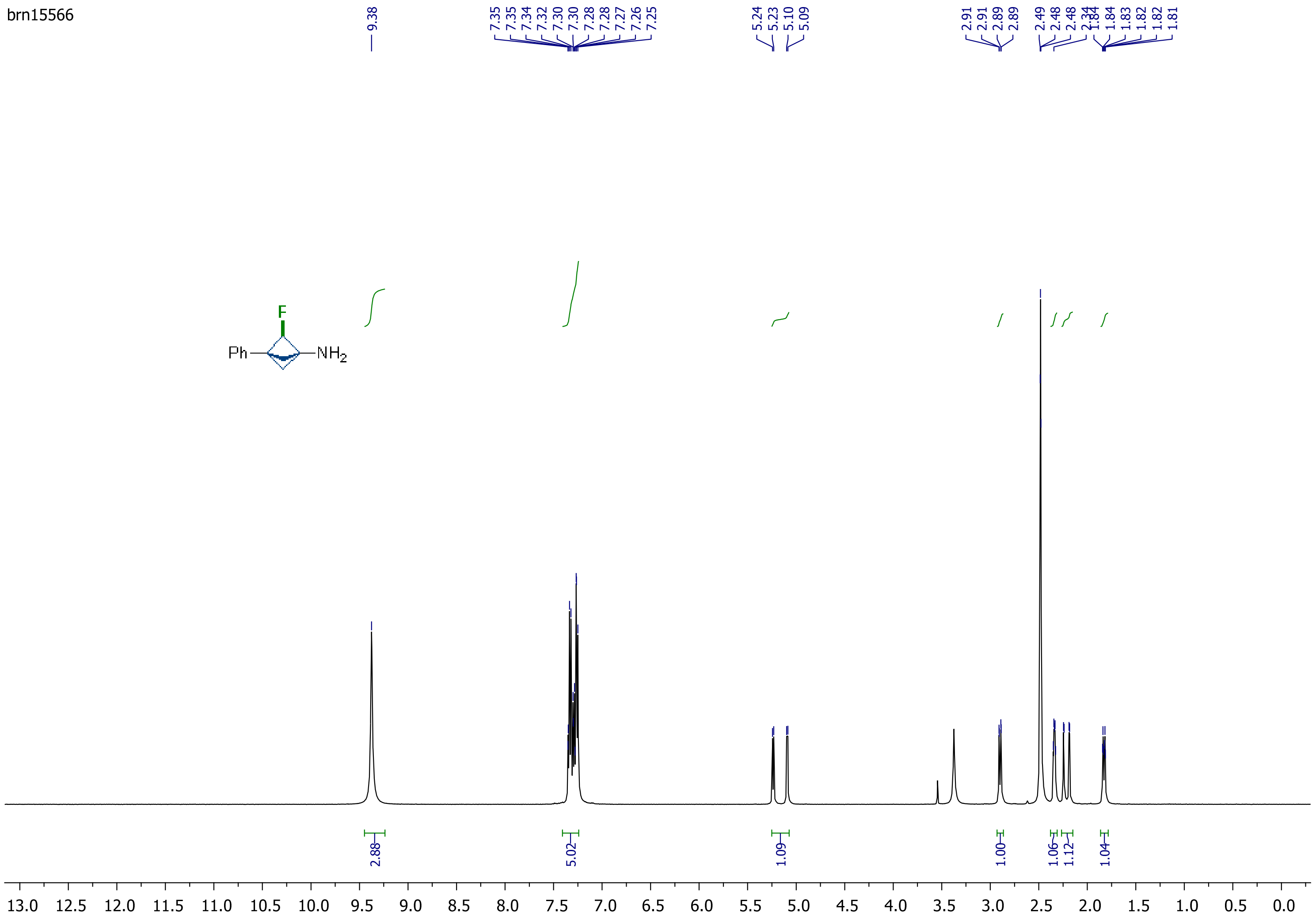

$^{13}C\{^1H\}$ NMR (126 MHz, DMSO-d$_6$)

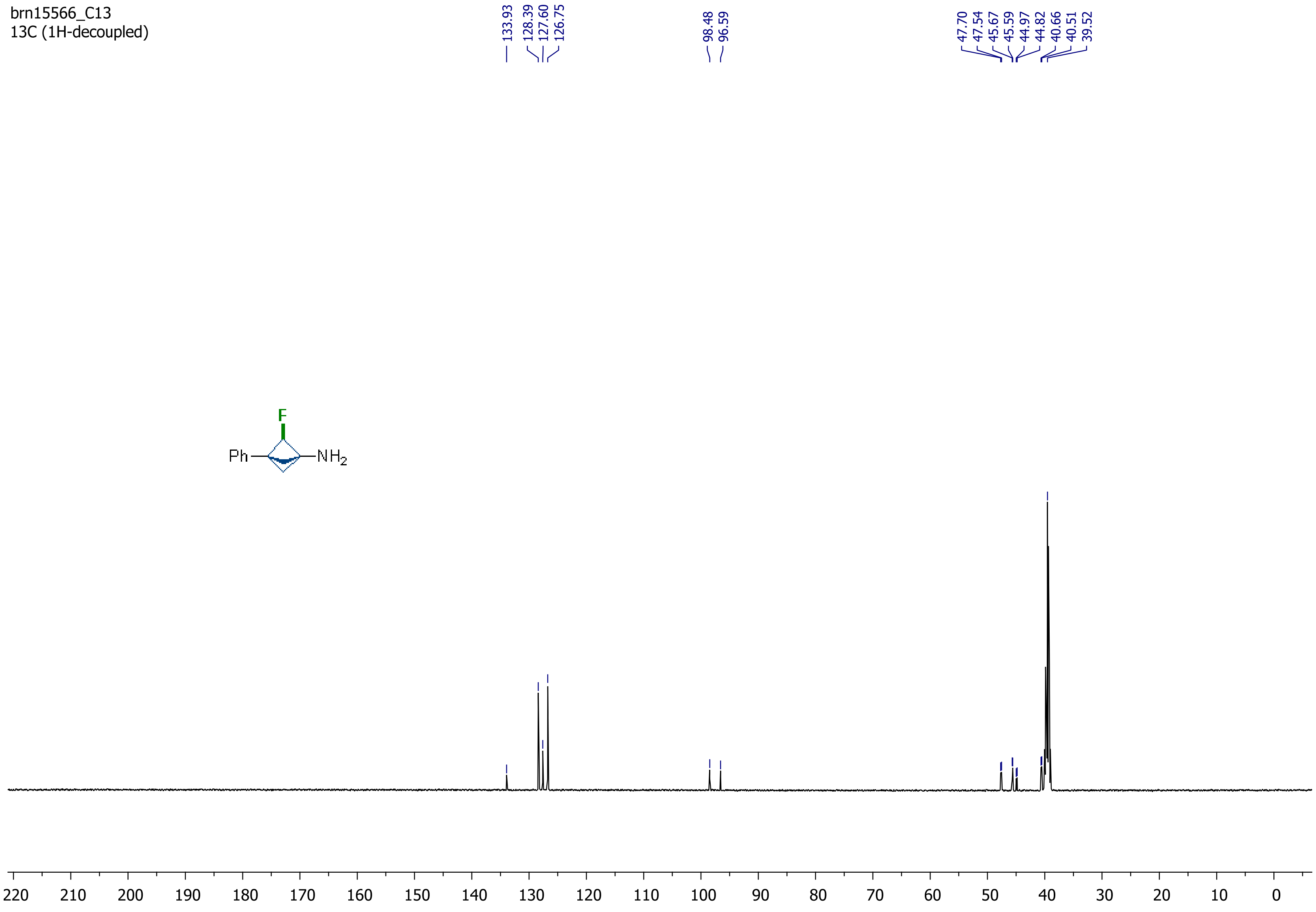

$^{19}F\{^{1}H\}$ NMR (376 MHz, DMSO-$d_6$)

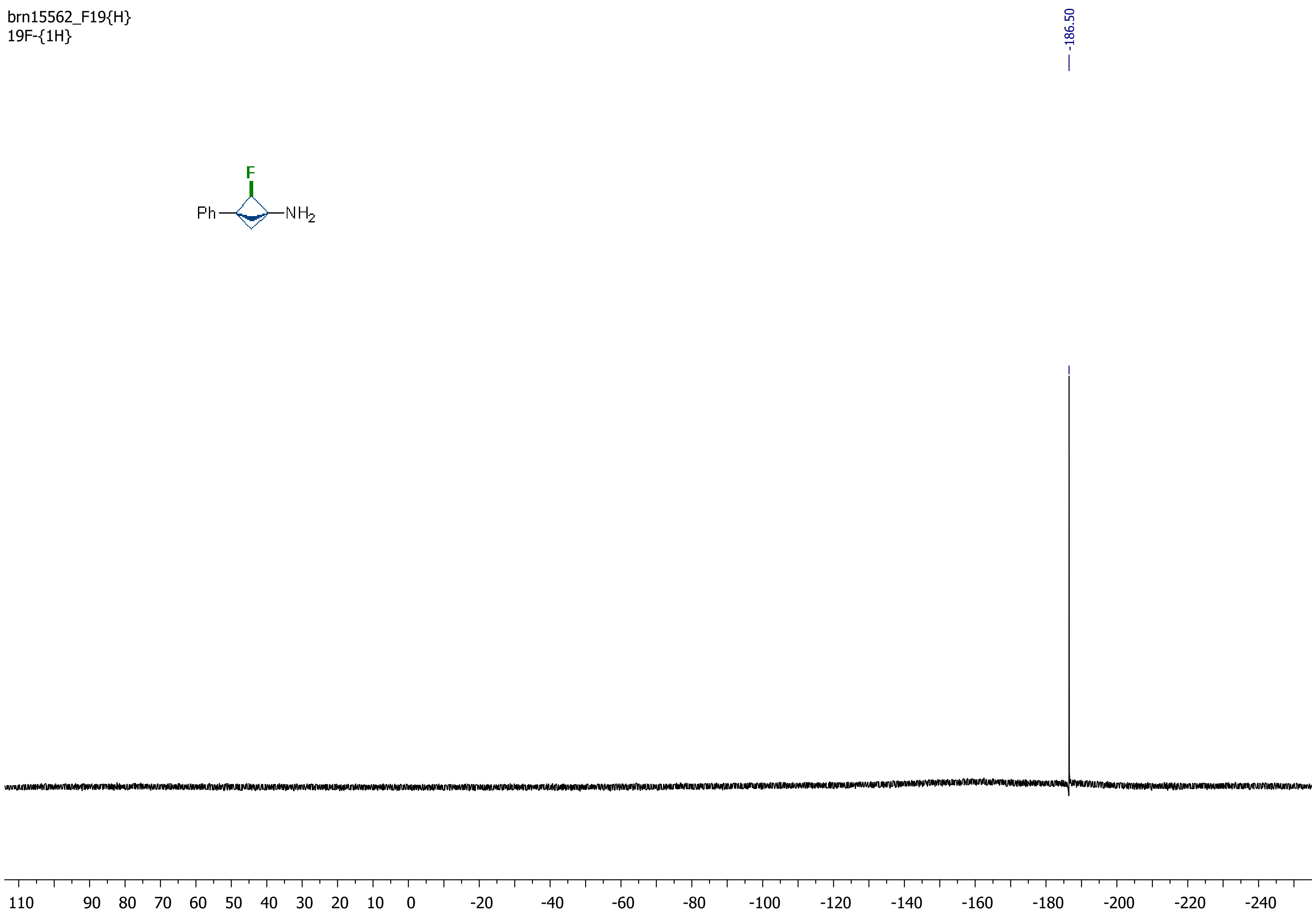

**Compound 33**

$^{1}$H NMR (400 MHz, $CDCl_3$)

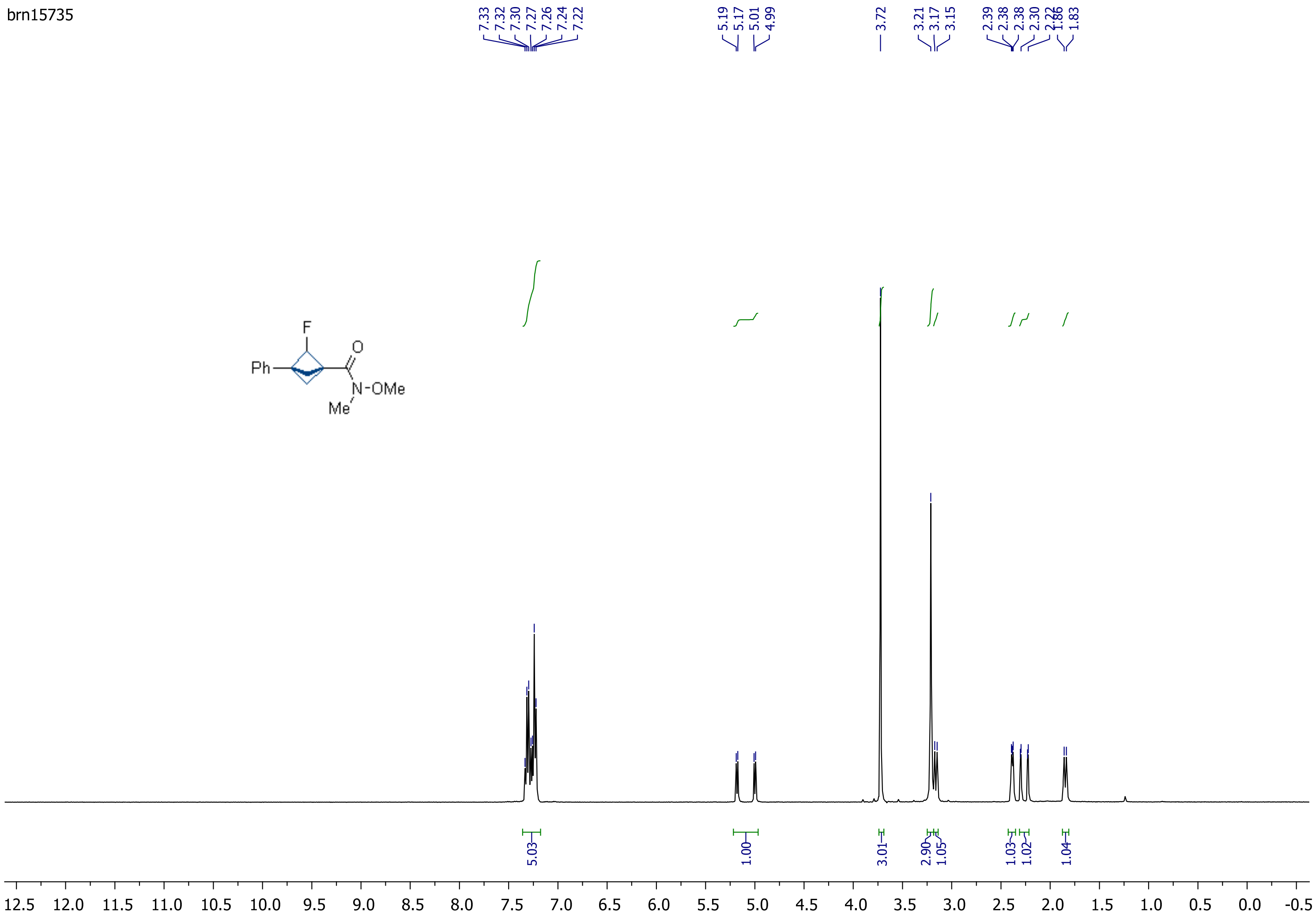

$^{13}C\{^1H\}$ NMR (101 MHz, $CDCl_3$)

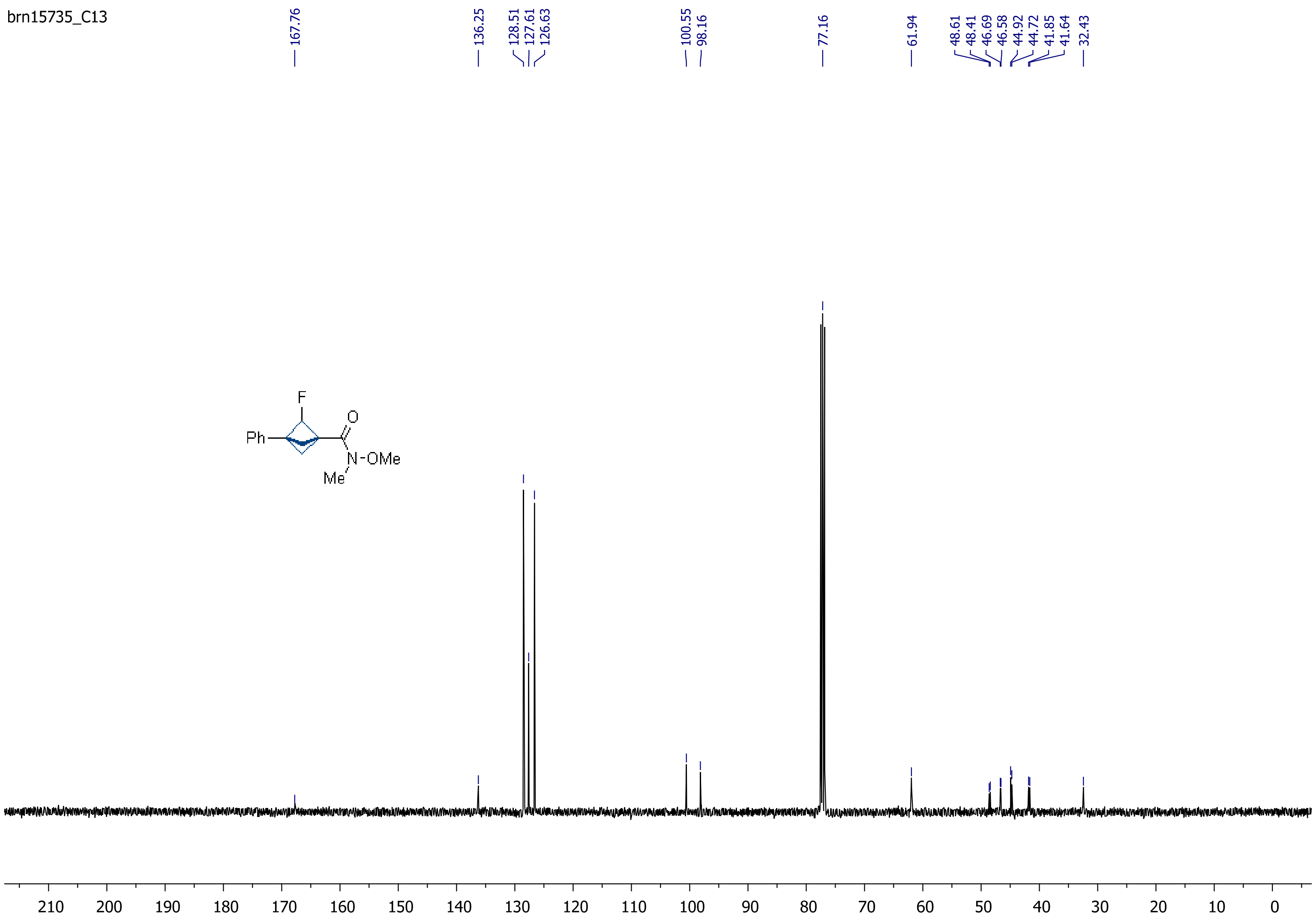

$^{19}F\{^{1}H\}$ NMR (376 MHz, $CDCl_3$)

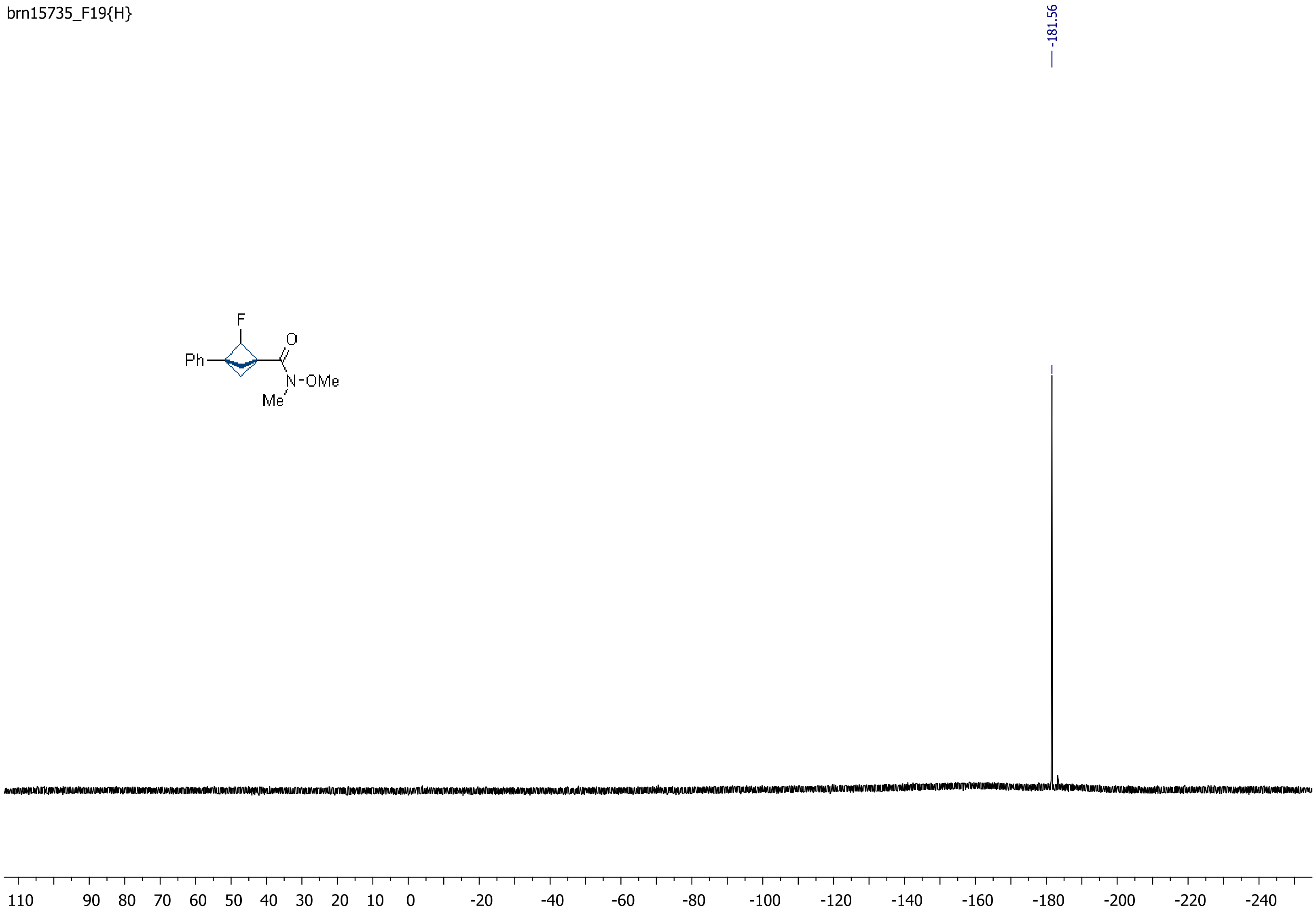

**Compound 34**

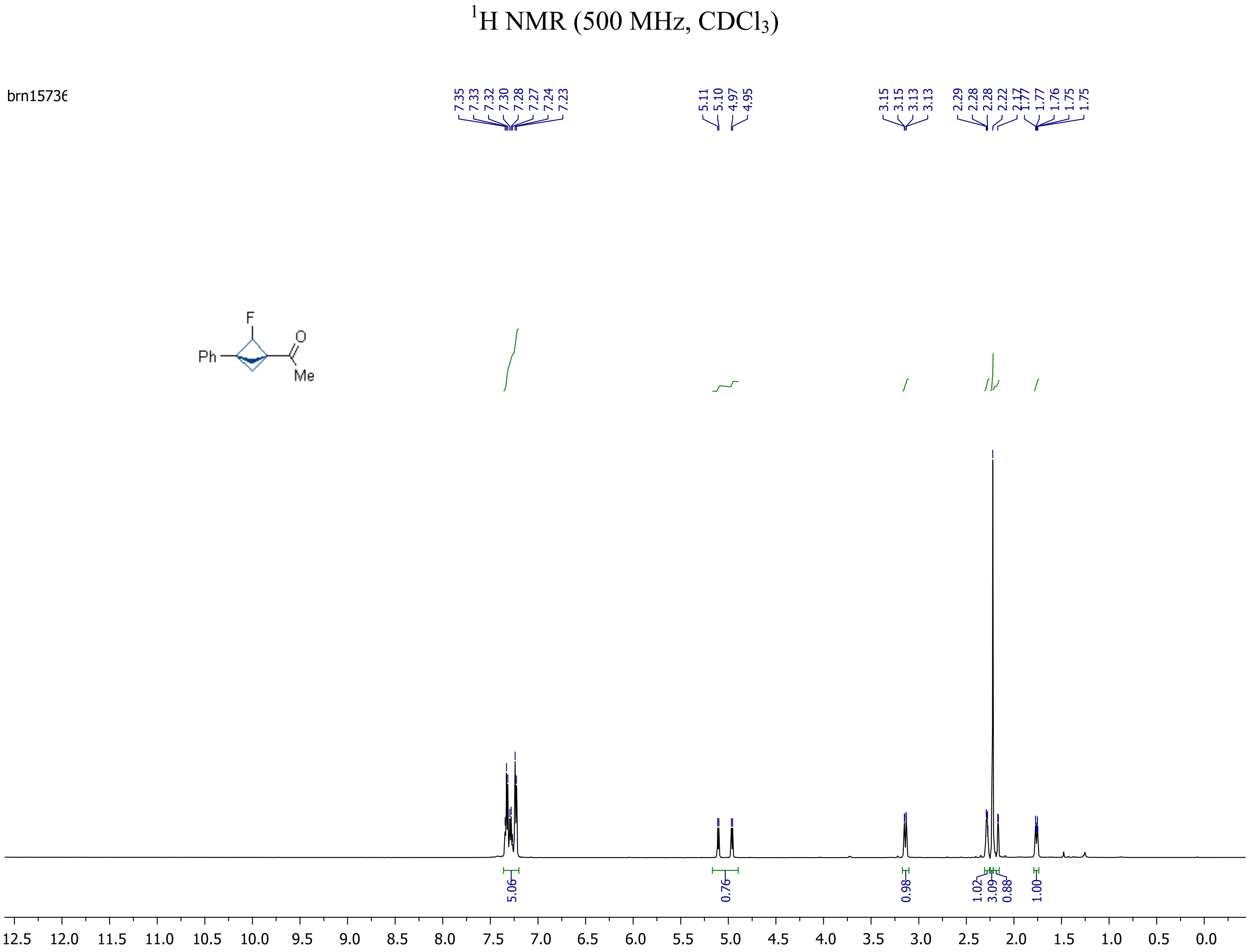

1H NMR (500 MHz, CDCl3)
brn15736
7.35
7.33
7.32
7.30
7.28
7.27
7.24
7.23
5.11
5.10
4.97
4.95
3.15
3.15
3.13
3.13
2.29
2.28
2.28
2.22
2.17
1.77
1.77
1.76
1.75
1.75
F
O
Ph
Me
5.06
0.76
0.98
1.02
3.09
0.88
1.00
12.5 12.0 11.5 11.0 10.5 10.0 9.5 9.0 8.5 8.0 7.5 7.0 6.5 6.0 5.5 5.0 4.5 4.0 3.5 3.0 2.5 2.0 1.5 1.0 0.5 0.0

$^{13}C\{^1H\}$ NMR (126 MHz, $CDCl_3$)

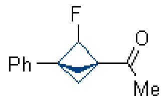


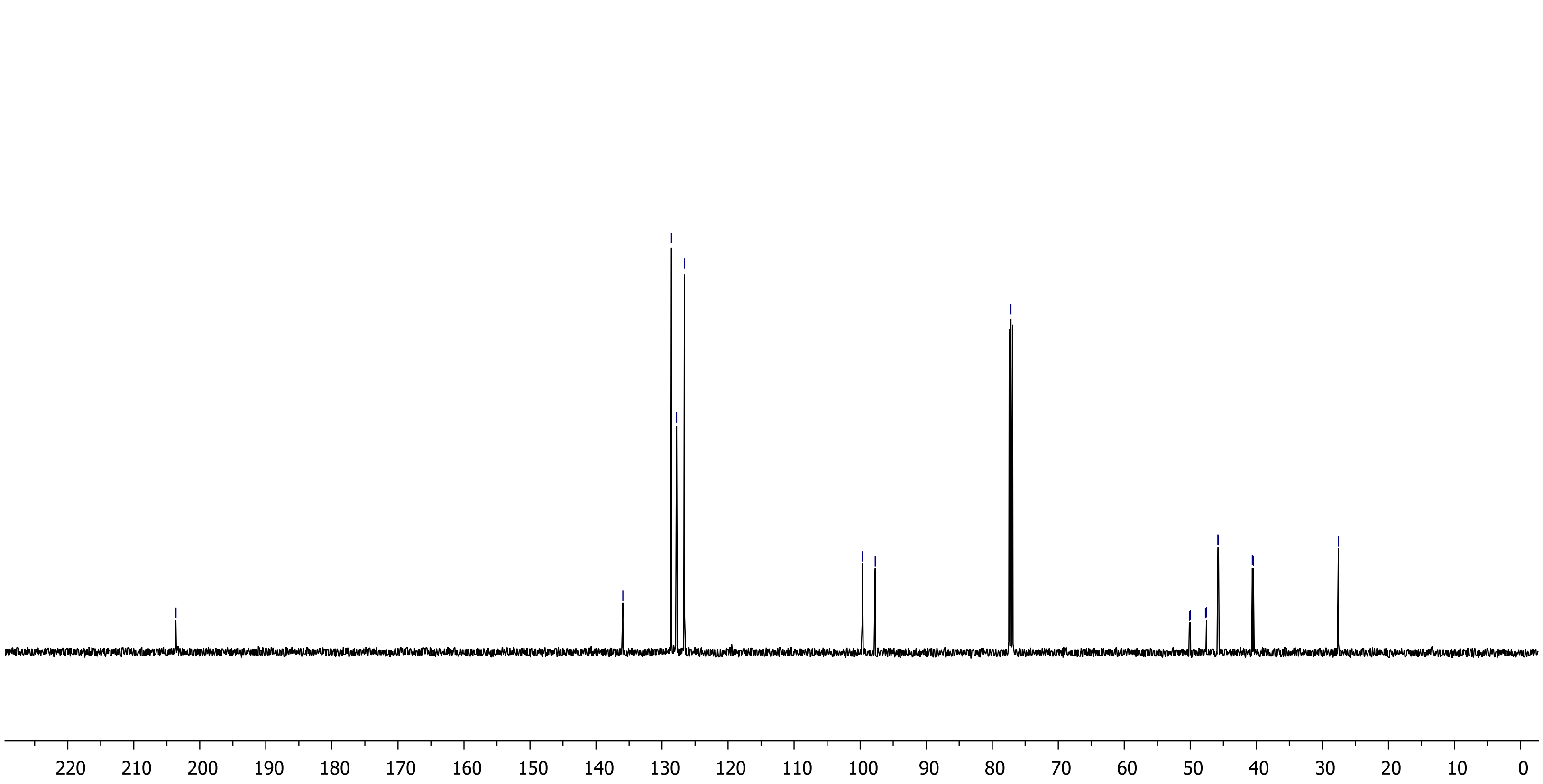

$^{19}F\{^{1}H\}$ NMR (376 MHz, $CDCl_3$)

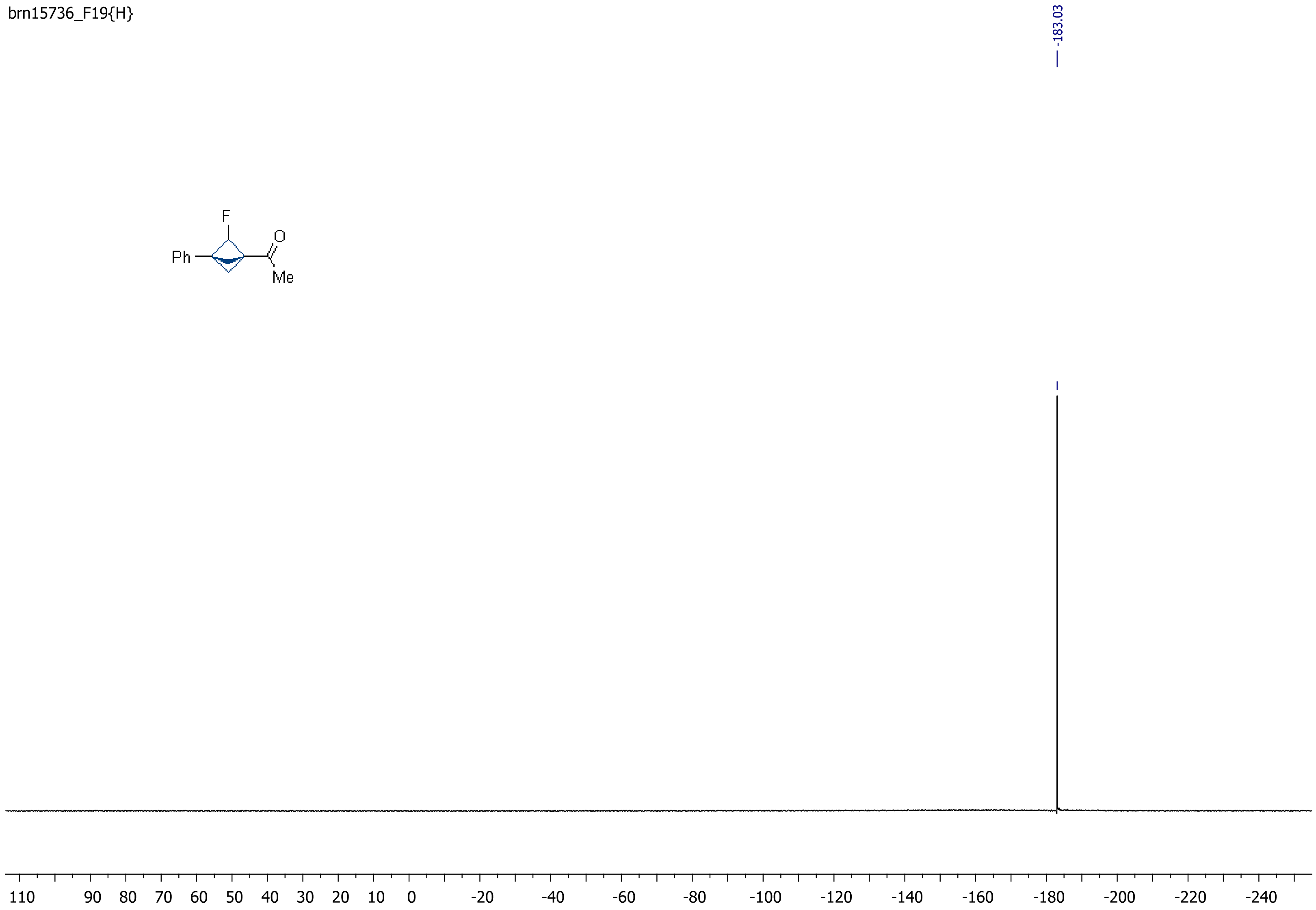

**Compound 35**

$^{1}$H NMR (400 MHz, $CDCl_3$)

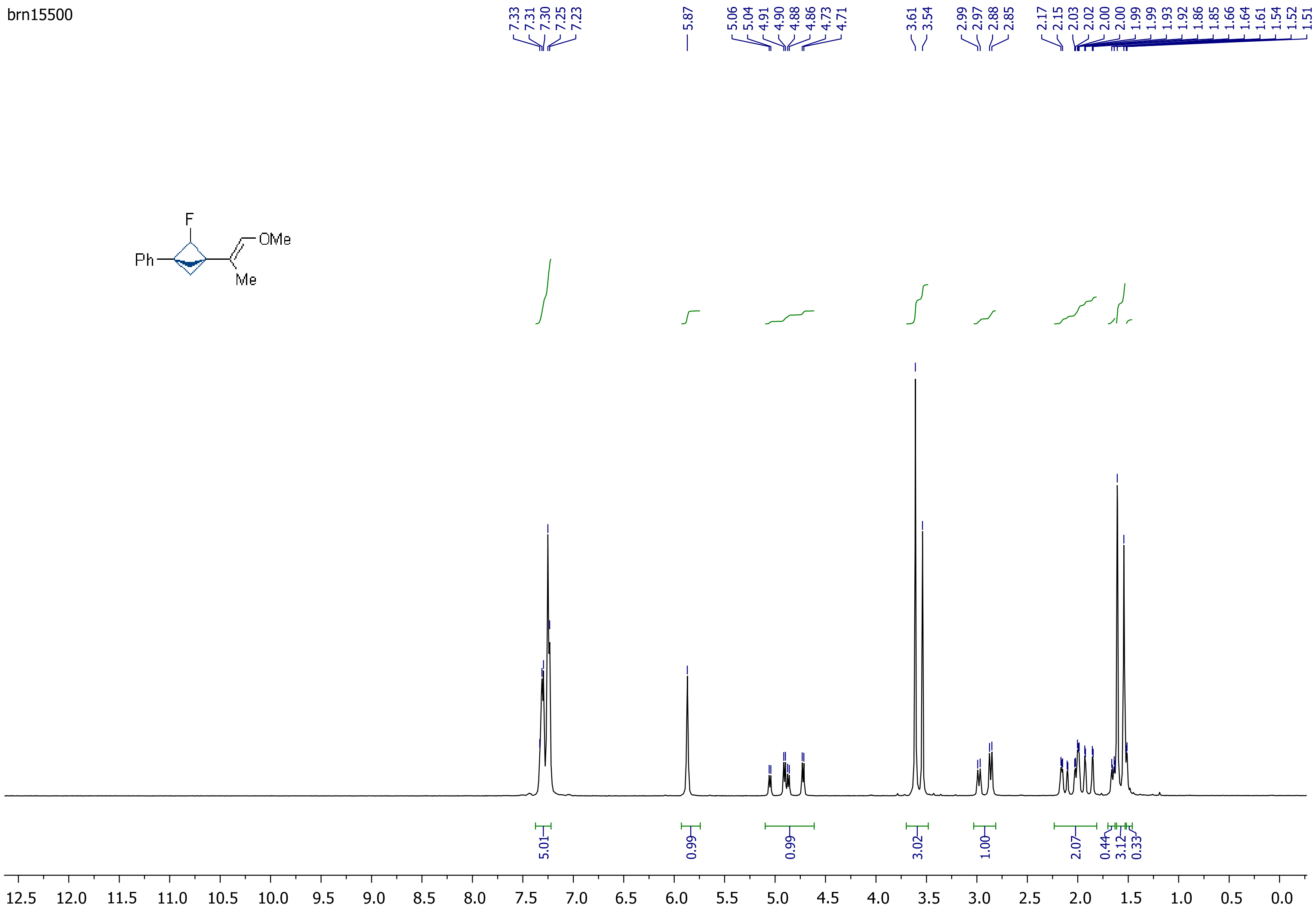

$^{13}C\{^1H\}$ NMR (101 MHz, $CDCl_3$)

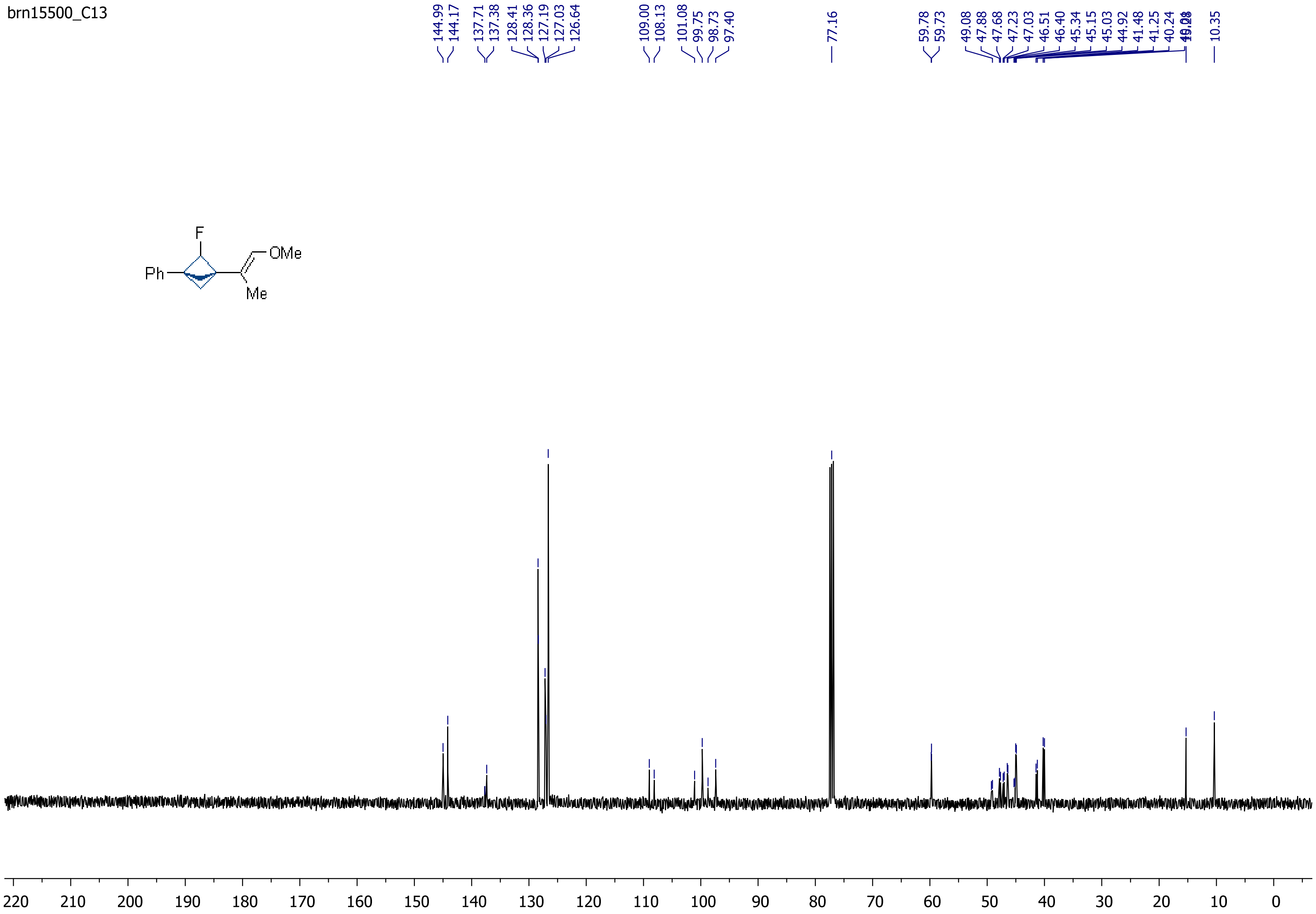

$^{19}F\{^1H\}$ NMR (376 MHz, $CDCl_3$)

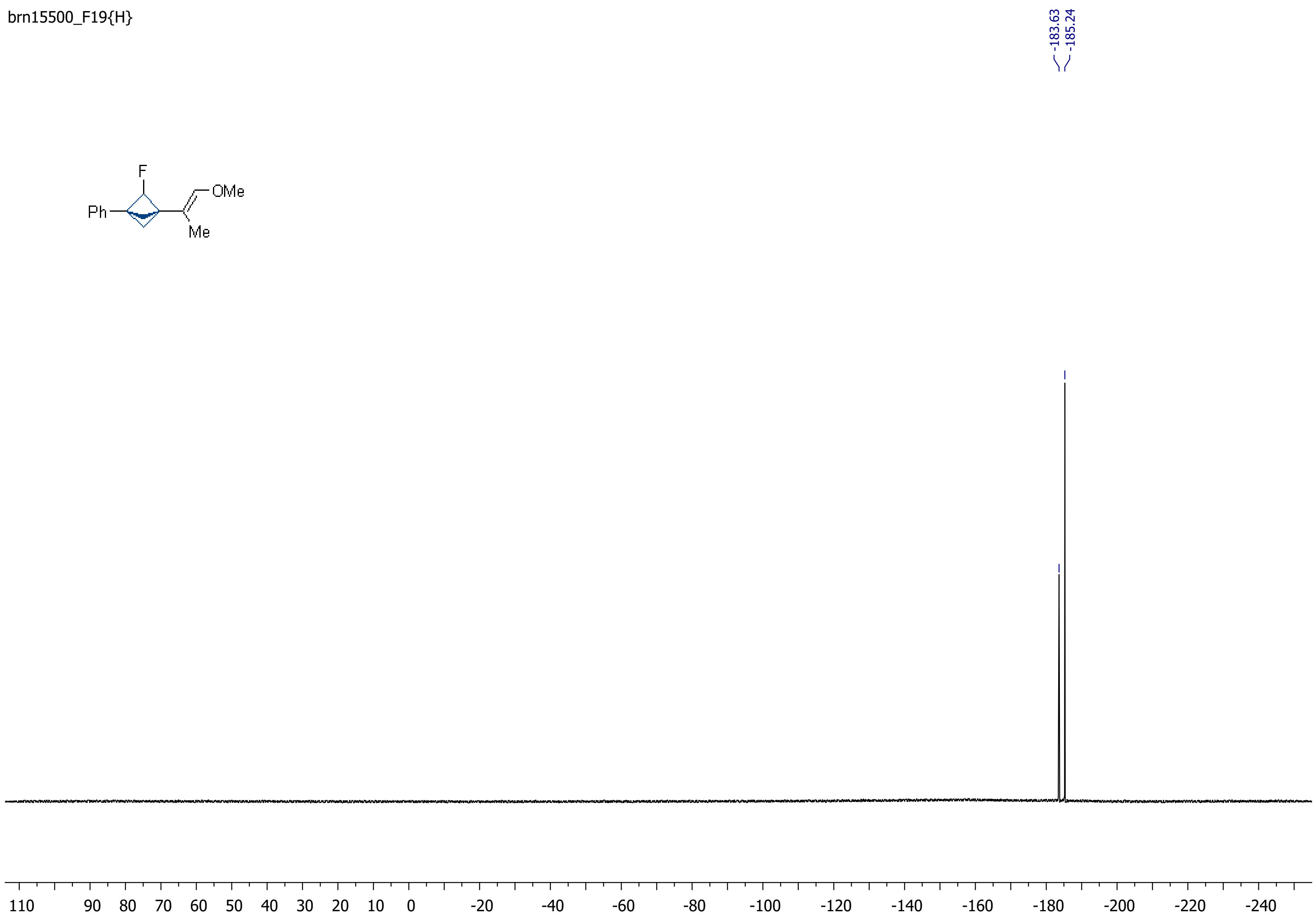

**2-(2-Fluoro-3-phenylbicyclo[1.1.1]pentan-1-yl)propanal**

$^{1}$H NMR (500 MHz, $CDCl_3$)

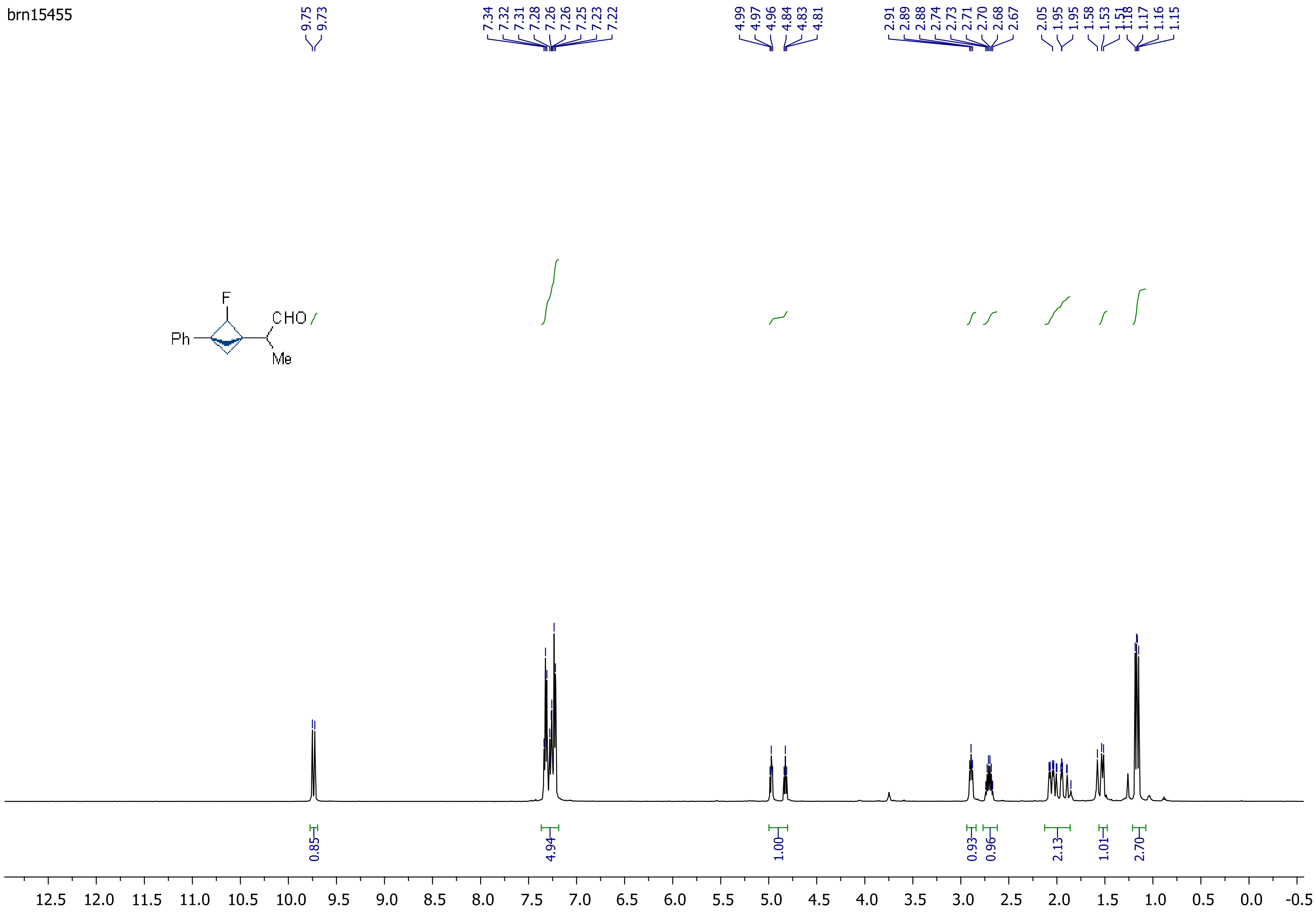

$^{19}F\{^{1}H\}$ NMR (376 MHz, $CDCl_3$)

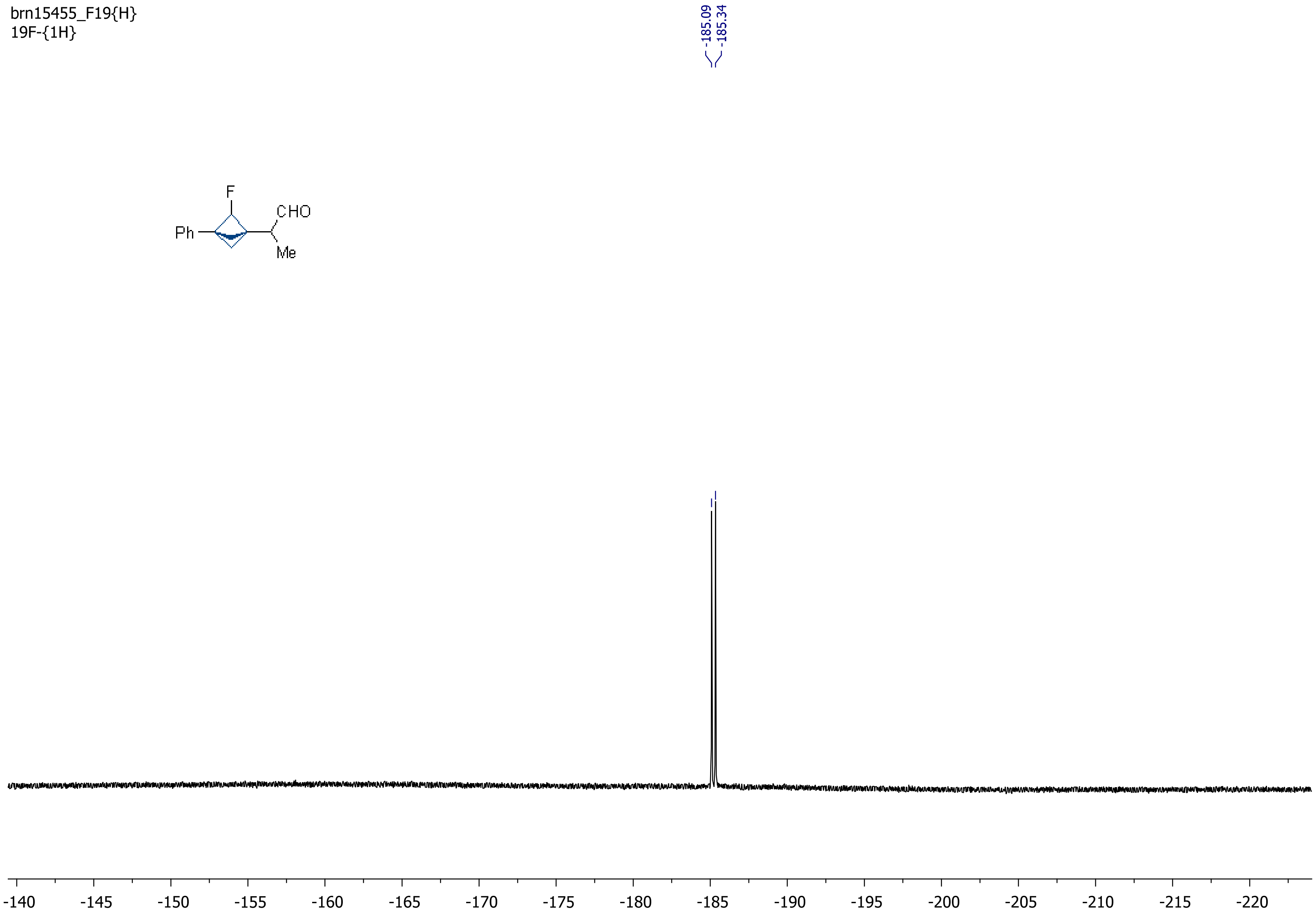

**Compound 37**

$^{1}$H NMR (400 MHz, $CDCl_3$)

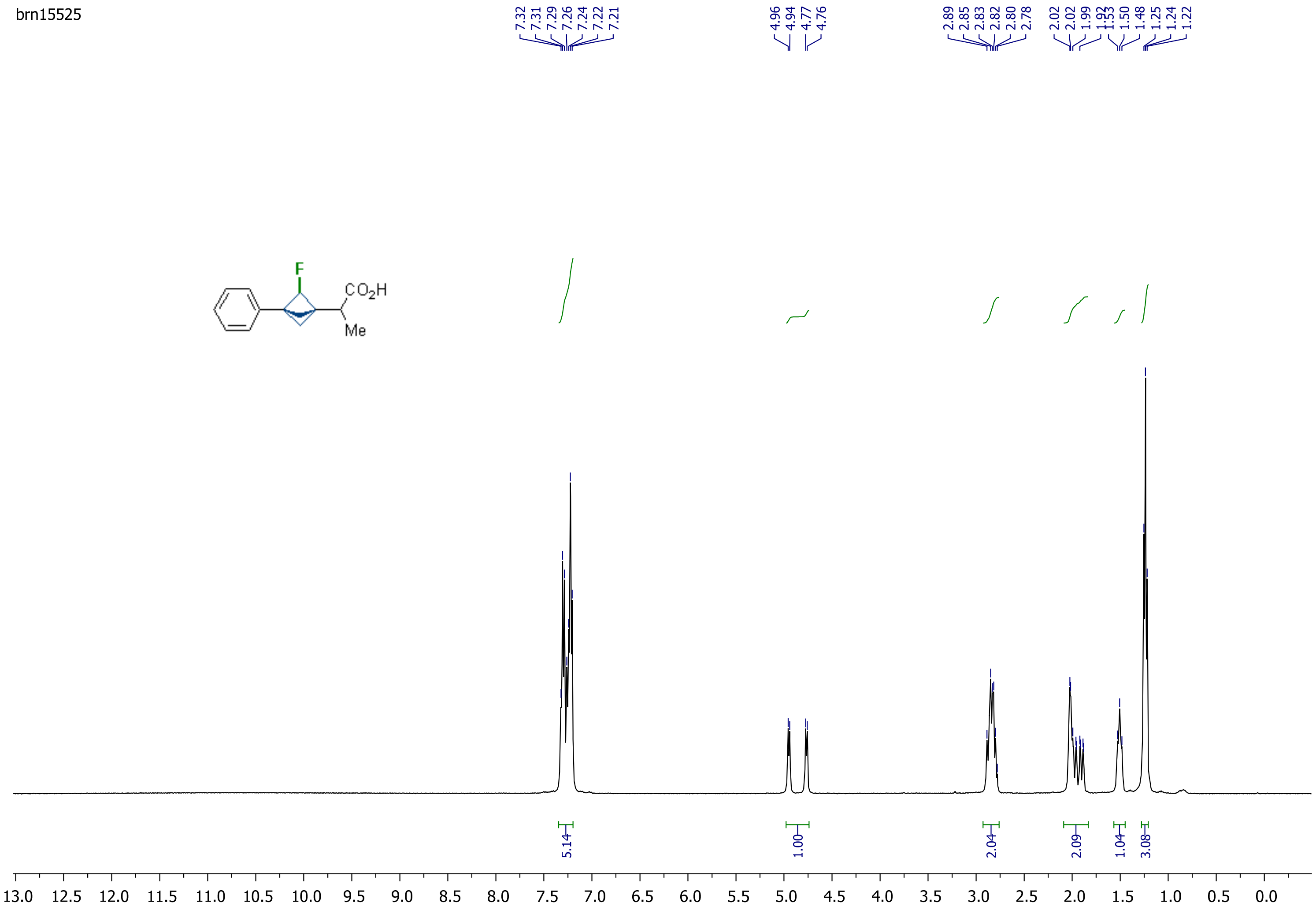

$^{13}C\{^{1}H\}$ NMR (126 MHz, $CDCl_3$)

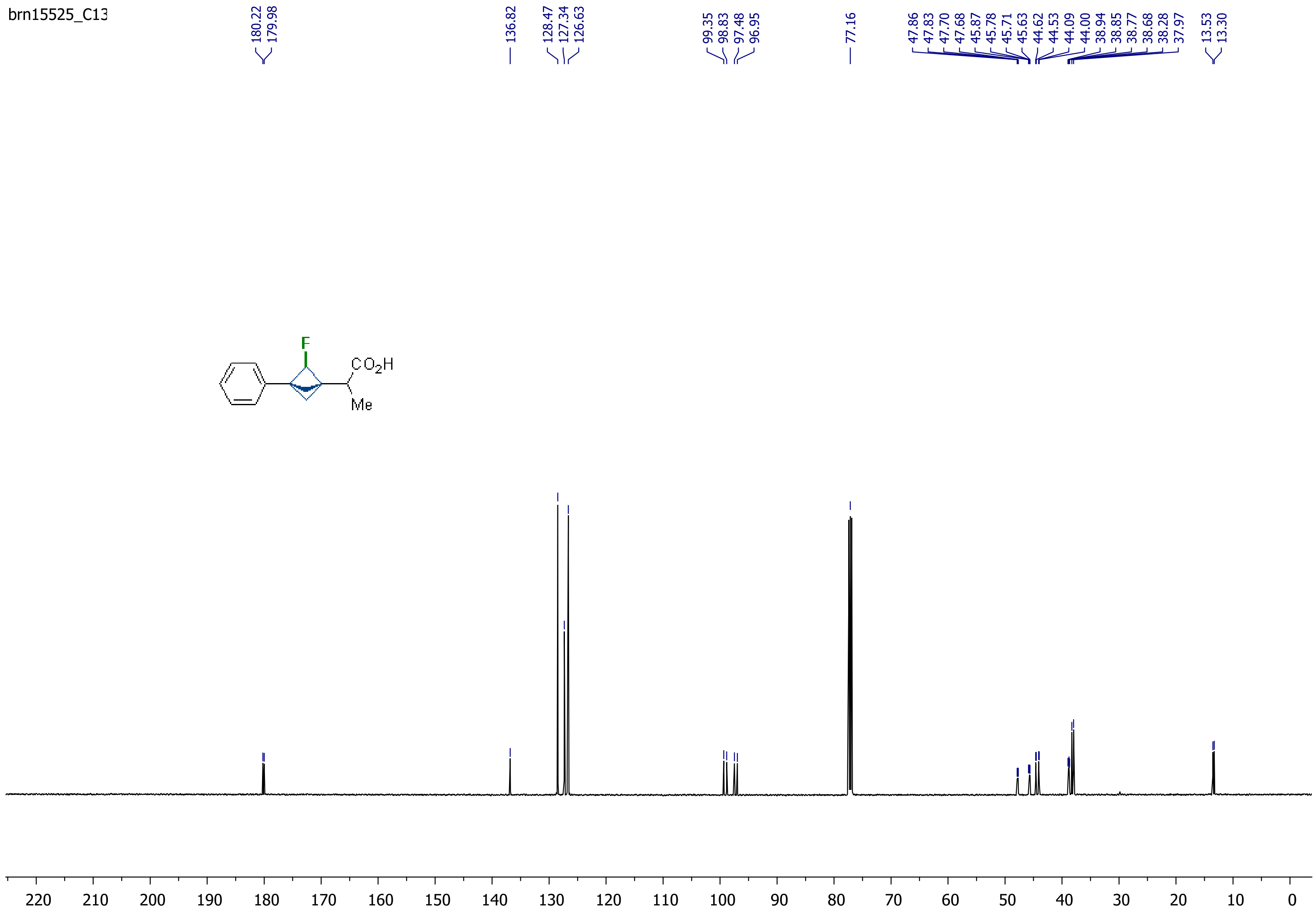

$^{19}F\{^1H\}$ NMR (376 MHz, $CDCl_3$)

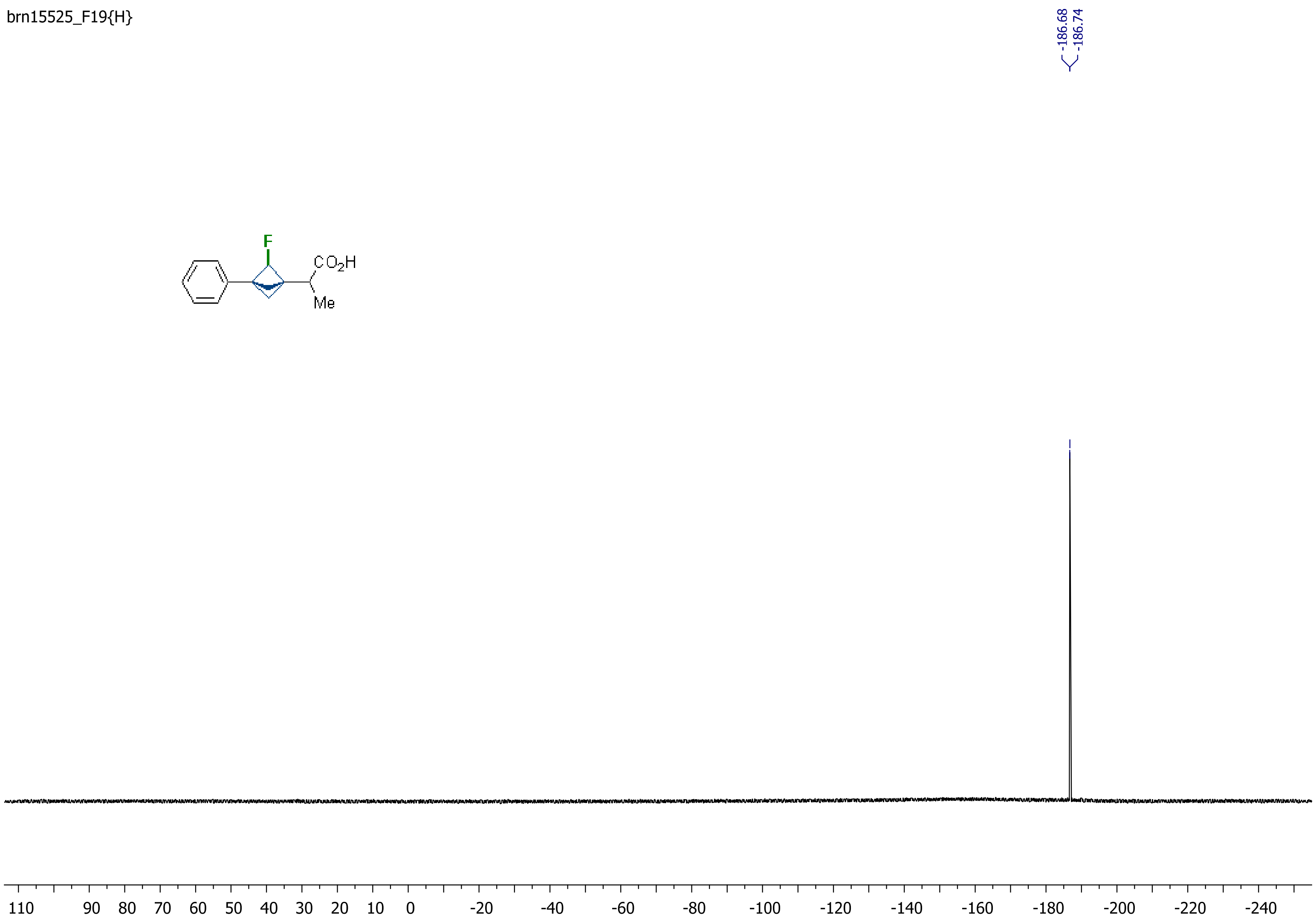

Supplement: Supplementary file 1 — Supporting Information [file ANIE-61-0-s001.pdf]
